# The effect of universal testing and treatment on HIV stigma in 21 communities in Zambia and South Africa

**DOI:** 10.1097/QAD.0000000000002658

**Published:** 2020-08-06

**Authors:** Anne L. Stangl, Triantafyllos Pliakas, Tila Mainga, Mara Steinhaus, Constance Mubekapi-Musadaidzwa, Lario Viljoen, Rory Dunbar, Ab Schaap, Sian Floyd, Nomtha Mandla, Virginia Bond, Graeme Hoddinott, Sarah Fidler, Richard Hayes, Helen Ayles, Peter Bock, Deborah Donnell, James R. Hargreaves

**Affiliations:** a International Center for Research on Women, Washington, DC; b Hera Solutions, Baltimore, Maryland, USA; c London School of Hygiene and Tropical Medicine, London, UK; d Zambart, School of Public Health, University of Zambia, Lusaka, Zambia; e Desmond Tutu TB Centre, Department of Paediatrics and Child Health, Faculty of Medicine and Health Sciences, Stellenbosch University, Cape Town, South Africa; f Department of Infectious Disease, Imperial College, London, UK; g SCHARP, Seattle, Washington, USA.

**Keywords:** antiretroviral therapy, community, healthcare, HIV, people living with HIV, stigma, sub-Saharan Africa

## Abstract

**Objectives:**

To assess the impact of a combination HIV prevention intervention including universal testing and treatment (UTT) on HIV stigma among people living with HIV, and among community members and health workers not living with HIV.

**Design:**

This HIV stigma study was nested in the HPTN 071 (PopART) trial, a three-arm cluster randomised trial conducted between 2013 and 2018 in 21 urban/peri-urban communities (12 in Zambia and nine in South Africa).

**Methods:**

Using an adjusted two-stage cluster-level analysis, controlling for baseline imbalances, we compared multiple domains of stigma between the trial arms at 36 months. Different domains of stigma were measured among three cohorts recruited across all study communities: 4178 randomly sampled adults aged 18–44 who were living with HIV, and 3487 randomly sampled adults and 1224 health workers who did not self-report living with HIV.

**Results:**

Prevalence of any stigma reported by people living with HIV at 36 months was 20.2% in arm A, 26.1% in arm B, and 19.1% in arm C (adjusted prevalence ratio, A vs. C 1.01 95% CI 0.49–2.08, B vs. C 1.34 95% CI 0.65–2.75). There were no significant differences between arms in any other measures of stigma across all three cohorts. All measures of stigma reduced over time (0.2--4.1% reduction between rounds) with most reductions statistically significant.

**Conclusion:**

We found little evidence that UTT either increased or decreased HIV stigma measured among people living with HIV, or among community members or health workers not living with HIV. Stigma reduced over time, but slowly.

**ClinicalTrials.gov number:**

NCT01900977.

## Introduction

Stigma is a barrier to the HIV care continuum, creating gaps across the prevention and treatment cascades and hampering the global goal of ending the AIDS epidemic by 2030 [1]. HIV stigma, a difference distinguished and labelled [2], results from drivers and facilitators including negative and judgmental attitudes towards people living with HIV (PLHIV), shame of HIV-positive status, and social, cultural, and gender norms [3]. Stigmatizing practices and experiences [3] deny PLHIV full social acceptance, consequently reducing their life chances, deterring access to essential services [4], and fuelling social inequalities [5].

HIV stigma is particularly harmful in healthcare settings [6,7], and stigmatizing attitudes and actions among health workers may hinder efforts to control HIV [8,9]. PLHIV perceiving that health workers will not maintain confidentiality [10], or anticipating stigma while accessing services, have been identified as barriers to HIV testing [10,11]. HIV stigma also dissuades timely linkage to care [12]. Fear of ‘being seen’ in the health clinic has been linked to demarcated HIV services, visibility, and distinctive client flow [13]. Internalized stigma, where PLHIV apply negative feelings to themselves, has been linked with refusal of ART [12] and poor ART adherence [14]. In low-income and middle-income countries, PLHIV who perceive high HIV stigma are more likely to delay enrolment in HIV care [15].

It has been hypothesized that universal testing and treatment (UTT) interventions will normalize HIV and reduce stigma through a nondiscriminatory approach that offers community-wide HIV testing to all, irrespective of perceived risk of infection, and provides ART to all PLHIV, irrespective of immune status [16]. This could negate the need for specific stigma mitigation interventions. Although interventions to reduce HIV stigma and discrimination have been tested in multiple contexts [17,18], it is unclear whether population-level biomedical interventions, such as UTT, can reduce stigma. Conversely, large-scale implementation of community-based HIV testing [19] and a shift to treatment as prevention [20] could lead to increased stigma. Burnout among health workers is also a concern, given the increase in clients at health facilities implementing UTT, which could in turn increase HIV stigma [21].

The HPTN 071 (PopART) trial was a three-arm cluster randomized trial conducted between 2013–2018 in 21 urban/peri-urban communities (12 in Zambia and 9 in Western Cape Province, South Africa). The study found that the PopART HIV combination prevention intervention package was successful in reducing HIV incidence by approximately 20% compared with standard of care [22]. In a sub-study, we evaluated the impact of the PopART intervention on HIV stigma.

## Methods

### Trial design

We nested a mixed-method study within the HPTN 071 (PopART) trial to assess the effect of the intervention on HIV stigma and report quantitative results here. Details of the main and sub-study designs have been described previously [16,23] (Supplemental File 1_HPTN 071 Study Protocol:). Briefly, the 21 study communities were arranged in 7 triplets matched on geographical location and estimated HIV prevalence. Communities in each triplet were randomly allocated to three study arms using restricted randomization to ensure balance across study arms on population size, baseline ART coverage and HIV prevalence [16].

### Intervention

Communities in Arm A received the full PopART package [22] including home-based HIV testing and an offer of immediate ART for those testing HIV-positive. Arm B received the full package except that ART initiation followed national guidelines at the time, which were initially based on CD4^+^ cell count but changed to ‘immediate ART’ by 2016, so arms A and B were alike in all respects from that point. In arms A and B, community HIV-care Providers (CHiPs) were employed by the trial to deliver the package at annual household visits. The procedure for the recruitment of CHiPs is described elsewhere [16]. Arm C received standard of care for HIV testing with ART initiation according to national guidelines. In all trial arms, health facility workers and existing community-based health workers received training on HIV care and ART to ensure national guidelines were adhered to. Health workers in arms A and B also received training specific to the PopART intervention.

### Populations of interest

We collected data on HIV stigma from three distinct populations, two of which were sub-sets of the main population cohort of the HPTN 071 (PopART) trial, procedures for which are described in more detail below (Supplemental File 2_HPTN 071a Stigma Protocol:). First was a cohort of adults living with HIV recruited as part of the population cohort, who both self-reported living with HIV and were laboratory confirmed as HIV-positive. We refer to this group as population cohort (PC)-HIV+^SR^. Second was a cohort of adults who were not living with HIV, also recruited as part of the population cohort, and who were HIV-negative in a laboratory test and did not self-report that they were positive. We refer to this group as PC-HIV−. Third was an open cohort of health workers recruited in all communities as part of a separate sub-study. We restricted analyses to health workers who self-reported they were HIV-negative. We refer to this group as health worker (HW)-HIV-.

### Data collection procedures

Population cohort recruitment occurred between December 2013 and March 2015 (PC0), and this closed cohort was followed up after 12, 24 and 36 months (PC12/PC24/PC36). The target sample size was 2500 adults per study community, of whom 15% were expected to be living with HIV. In randomly sampled households, one adult resident aged 18–44 years was selected at random. Participants completed an interviewer-administered questionnaire at their home with data captured on electronic capture devices (ECDs) [16]. Additional participants were enrolled in some study communities at 12 and 24 months, excluding households already sampled, as the target sample size was not reached at PC0 [22]. PC-HIV+^SR^ participants were asked about experienced and internalized stigma. A random sample of 20% of PC-HIV− participants received a series of questions on HIV stigma-related attitudes and perceptions (Supplemental Table 1). The final survey at PC36 reached 72% of eligible participants, with similar retention across the trial groups (73, 73, and 71% in groups A, B, and C, respectively) [22].

Data on attitudes and perceptions of HW-HIV− were collected from a nested open cohort study that sought to recruit all health facility staff in all communities, including doctors, nurses, pharmacists, counsellors, security guards, and other community-based health workers, including CHiPs [23]. These data were collected at health facilities using self-administered surveys on ECDs in three waves, first (R1) between August 2014 to May 2015 (8--18 months after the trial began [24]), and then again between June 2015 to June 2016 (R2), and between January 2017 and February 2018 (R3). There was significant turnover in the eligible population; participation rates at each round were 63.7--75.7% and were slightly higher in South Africa than Zambia (Supplemental Figure 1).

### Outcome evaluation

Using a ‘parallel’ approach [25], we asked the three populations about different domains of stigma from different perspectives, but using similar wording. For example, we asked PC-HIV+^SR^ if they were ‘talked badly about’, PC-HIV− whether they perceived that people living with HIV were ‘talked badly about’, and HW-HIV− whether co-workers had ‘talked badly about’ PLHIV.

Specifically, PC-HIV+^SR^ participants responded to 12 items capturing five stigma domains/outcomes: current internalized stigma (three items), stigma experienced in community (five items) or healthcare settings (three items), and a single item asking about whether those who had experienced stigma had confronted, challenged, or educated someone who was stigmatizing and/or discriminating against them. We used an indicator that combined the first three stigma outcomes to reflect any stigma reported by PC-HIV+^SR^ as our main outcome in this article. PC-HIV− participants responded to 11 items capturing four stigma domains/outcomes: hesitation to test because of fear of other people's reaction if the test was positive (one item), fear and judgement of PLHIV (three items), and perceived stigma in community (five items) or healthcare settings (two items). HW-HIV− participants responded to 14 items capturing three stigma domains/outcomes: fear and judgement of PLHIV (five items), perceived stigma in the community (five items) and perceived co-worker stigma (four items). HW-HIV− participants also responded to items related to job stress using the Maslach Burnout Inventory, which constitutes 22 items in three separate domains: emotional exhaustion, depersonalization, and personal accomplishment [26] (Supplemental Table 1). In this analysis, we used the nine items that measure emotional exhaustion defined as ‘feelings of being emotionally overextended and exhausted by one's work’ to categorize HW-HIV− as having low (0–18) or moderate/high (19/54) levels of emotional exhaustion.

Stigma items were precoded using either a four-item Likert scale ('strongly agree’, ‘agree’, ‘disagree’, ‘strongly disagree’) or response categories capturing the frequency of experiences in the last year. These categories were ‘never’, ‘once’, ‘a few times’, ‘often’, or ‘n/a because no one knows my status’ for the stigma items and ‘never’, ‘few times a year’, ‘once a month or less’, ‘few times a month’, ‘once a week’, ‘few times a week’ or ‘everyday’ for the job stress items. All 13 outcomes were collapsed into binary variables coded as ‘disagree’ vs. ‘agree’ or ‘never’ vs. ‘at least once’. All stigma measures used in the analysis were assessed to be valid and reliable in our study populations [27].

### Statistical analysis

We first described the three cohorts recruited at baseline (PC0 and R1) and assessed the data for any evidence of imbalance across trial arms. At endline, we described missing data exclusions from the cohorts (Supplemental Figure 2), before describing the sociodemographic characteristics of those included in the endline analysis.

For our main analysis, we used endline (PC36 or R3) data from all three populations. We calculated summary statistics in the form of geometric means of the cluster level prevalences for all outcomes at endline and included participants with complete data on stigma outcomes, age (18–24, 25–29, 30–34, 35–39, and 40+ years), sex, marital status (married, never married, divorced, and widowed), education (less than secondary, completed secondary, and further education), and whether ever tested for HIV. In line with the analysis strategy for HIV incidence and viral suppression outcomes of the HPTN 071 (PopART) trial [22], we prespecified running the following comparisons: Arm A vs. arm C, and, arm B vs. arm C. Following the unblinding of the main trial and before we ran this analysis, we decided to also run the following comparison: arms A and B (pooled) vs. arm C, as interventions delivered in arm A and arm B were similar.

For all outcomes, we performed unadjusted and adjusted analyses following a two-stage cluster-level approach recommended for CRTs with less than 15 clusters per arm [28]. In the first stage, we ran logistic regression analyses using individual-level data with the triplet as a factor but not the study arm (unadjusted models). In the adjusted models, we included sex, age, the interaction between sex and age (treated as *a* priori confounders) and a linear term for baseline stigma prevalence at the community level. We calculated the expected number of stigma events for both models. In the second stage, we first calculated the log of the observed divided by the expected number of stigma events (log-ratio residual). We carried out linear regression including terms for triplet and study arm to compute the empirical standard error of the mean difference in the log-ratio residual. We subsequently calculated with exponentiation, the prevalence ratio with a 95% confidence interval using the *t*-distribution. For the main outcome only (any stigma reported by PC-HIV+^SR^), we stratified our analysis by sex, and adjusted for age and community-level baseline stigma prevalence, to examine differences between male and female individuals. In some subgroup analyses, there were communities with at least one eligible participant, but zero events. In these cases, 0.5 was added to the number of events and the number of participants for all communities in that triplet at the first stage of the analysis.

We examined secular trends over time for all stigma outcomes. We used cluster-level data and linear regression including terms for time (four time points, PC0--PC36, for stigma outcomes for PC-HIV+^SR^ and PC-HIV− and three time points, R1--R3, for stigma outcomes for HW-HIV−) and community. We also fitted a time--arm interaction term in these models to assess whether there were differences in secular trends across study arms.

We ran sensitivity analyses restricted to PC-HIV+^SR^ who had data at all four rounds, to assess whether the effects observed may have been attributed to cohort attrition. We also ran sensitivity analyses restricted to PC-HIV+^SR^ participants who were diagnosed prior to the start of the trial in 2014, to check whether effects were similar in this group as internalized stigma may be higher immediately following diagnosis [29] and experiences of stigma would be unlikely to occur prior to serostatus disclosure to others.

### Ethical considerations

Prior ethical approval for all study procedures was obtained from the institutional review boards of the London School of Hygiene and Tropical Medicine (LSHTM), Stellenbosch University, and the University of Zambia. All participants provided written informed consent prior to enrolment.

## Results

### Baseline balance between trial arms

We analysed data from 3825 PC-HIV+^SR^ and 4217 PC-HIV- recruited at PC0, and 851 HW-HIV− at R1. Although the prevalence of stigma among health workers was similar across arms at R1, stigma measures among PC-HIV+^SR^ and PC-HIV− participants was lower in group A compared with groups B and C across most outcomes at PC0 (Supplemental Table 2). At PC0, the geometric mean across clusters of the main outcome of interest, prevalence of any stigma among PC-HIV+^SR^ participants was 24.1% in group A, 35.5% in group B, and 45.3% in group C. Stigma experienced in the healthcare setting by PC-HIV+^SR^ participants was lower across all arms (2.5% in group A; 5.5% in group B; and 8.0 in group C).

### Study population at endline

At endline, we analysed data from 4178 PC-HIV+^SR^ and 3487 PC-HIV− included in PC36 and 1224 HW-HIV− at R3. In all three populations at endline, more participants were from Zambia than South Africa, reflecting the design of the study. More participants were women in all three of the study cohorts. Most PC-HIV+^SR^ and HW-HIV− participants were over 29 years of age, while most PC-HIV− participants were under 29 years of age. Around half of participants in each cohort were married. The HW-HIV− participants were more likely than the population cohort participants to have received further education. There was little suggestion that sociodemographic characteristics differed systematically by study arm at endline (Table 1).

**Table 1:**
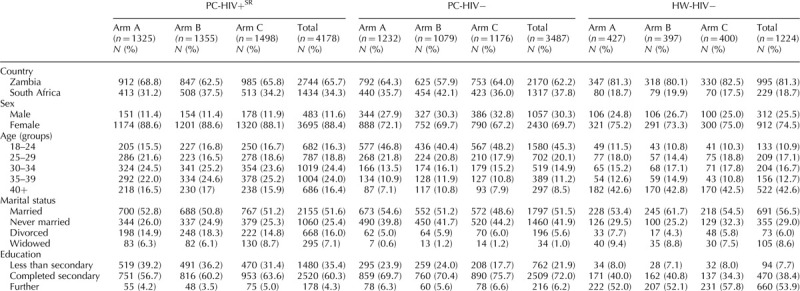
Sociodemographic characteristics of PC-HIV+^SR^, PC-HIV−, and HW-HIV− participants at endline (PC36/R3).

### Effect of the intervention on HIV stigma

At endline, the prevalence of any stigma among PC-HIV+^SR^ participants was 20.2% in group A, 26.1% in group B, and 19.1% in group C. There was no evidence of a difference between groups in either the unadjusted (A vs. C: PR: 1.06, 95% CI: 0.51–2.18; B vs. C: PR: 1.37, 95% CI: 0.49–2.08; A+B vs. C: PR: 1.20, 95% CI: 0.64–2.25) or adjusted models (A vs. C: aPR: 1.01, 95% CI: 0.49–2.08; B vs. C: aPR: 1.20, 95% CI: 0.65–2.75; A+B vs. C: aPR: 1.16, 95% CI: 0.62–2.17). The direction of effect was different (but not statistically significant) in male and female population when comparing impacts on our main outcome (any stigma among PC-HIV+^SR^ participants) between groups A and C but did not differ between groups B and C (Table 2). There was variation between study triplets in the direction and effect of differences in any stigma between the groups (Supplemental Figure 3).

**Table 2:**
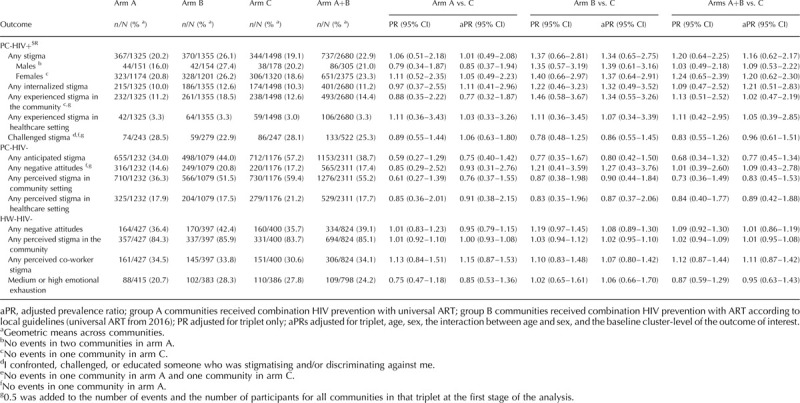
Comparison of intervention effects on stigma and job stress outcomes among women and men receiving combination HIV prevention with universal antiretroviral therapy, the intervention with antiretroviral therapy according to local guidelines (universal since 2016), and a standard care control group in Zambia and South Africa, 2015--2018.

**Fig. 1:**
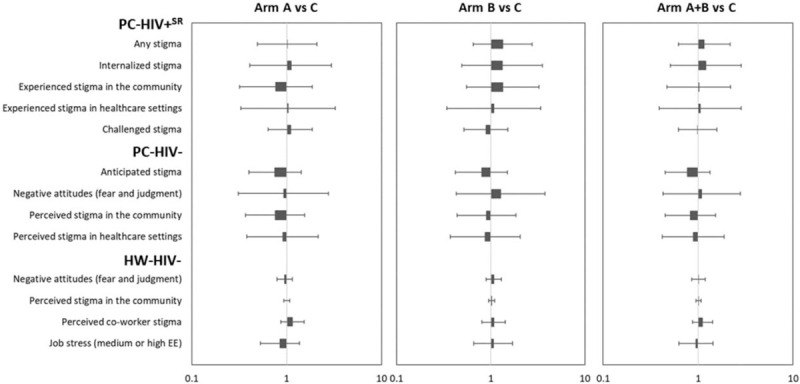
Adjusted prevalence ratios and 95% confidence intervals comparing arms of the trial at endline for all stigma outcomes.

There was no evidence of differences between arms in any other stigma outcome across any of the populations studied (Table 2 and Fig. 1). These findings were replicated in sensitivity analyses (Supplemental Table 3).

At the final round of data collection, 20.7% of HW-HIV− participants in arm A, 28.3% in arm B, and 27.8% in arm C reported medium or high emotional exhaustion. There was no evidence of difference in this outcome by arm in either the unadjusted (A vs. C: PR: 0.75, 95% CI: 0.47–1.18; B vs. C: PR: 1.02, 95% CI: 0.65–1.61; A+B vs. C: PR: 0.87, 95% CI: 0.59–1.29) or adjusted models (A vs. C: aPR: 0.85, 95% CI: 0.53–1.36; B vs. C: aPR: 1.06, 95% CI: 0.66–1.70; A+B vs. C: aPR: 0.95, 95% CI: 0.63–1.43) (Table 2).

### Trends in stigma outcomes over time

**Fig. 2:**
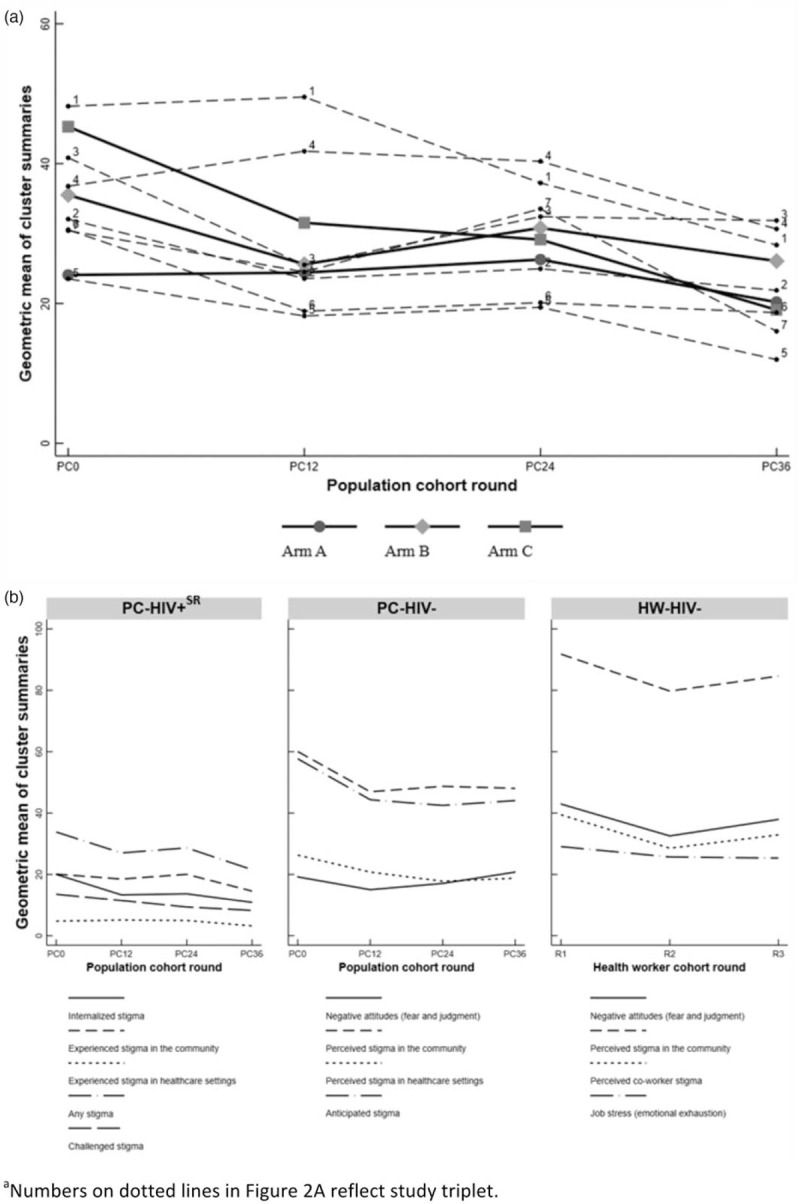
Trends in (a) any stigma reported by PC-HIV+^SR^ over time by study arm and Triplet^a^ and in (b) stigma outcomes and job stress over time by study population.

The prevalence of any stigma declined over the course of the study by −3.3% per year (−5.2% to −1.4%, *P* < 0.01; Table 3 and Fig. 2a), with variation across triplets. The reduction was steepest in arm C, which started with higher levels of stigma, and shallowest in arm A (interaction *P* = 0.011).

**Table 3:**
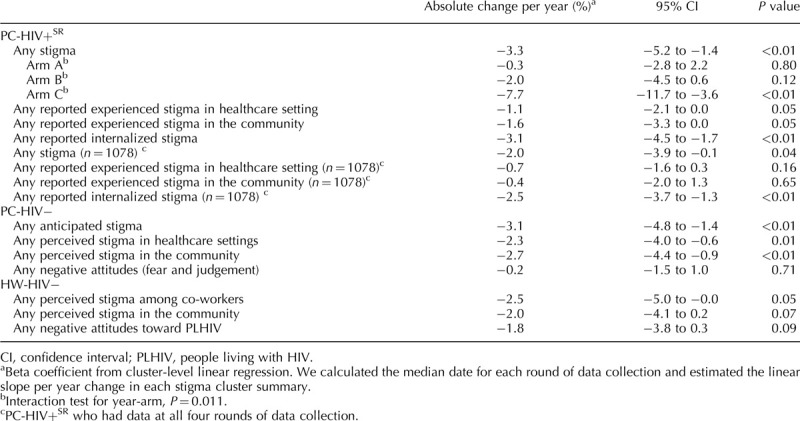
Trends over time in stigma outcomes.

Among all three populations and across all outcomes, stigma declined from the first round of data collection (PC0/R1) to the last (PC36/R3) (Table 3 and Fig. 2b). Perceived stigma in the community was higher among HW-HIV- than PC-HIV− across all time points (Fig. 2b). Among PC-HIV+^SR^, there was strong evidence of a reduction in those reporting internalized stigma (−3.1% (−4.5% to −1.7%), *P* < 0.01) and experienced stigma in the community [−1.6% (−3.3% to −0.0%), *P* = 0.05]. Among PC-HIV−, there was strong evidence of reductions in anticipated stigma, perceived stigma in healthcare settings, and perceived stigma in the community [between −2.3% per year (95% CI −4.0% to −0.6%) to −3.1% (−4.8% to −1.4%), *P* < 0.05]. We observed borderline significant reductions in all stigma outcomes among HW-HIV− (−1.8% to −2.5% per year, all *P* < 0.1) (Table 3).

## Discussion

We found little evidence that the implementation of a combination HIV prevention intervention including universal testing and treatment was associated with higher or lower stigma among PLHIV, or among community members or health workers not living with HIV, compared with standard of care at the end of the HPTN 071 (PopART) trial. Levels of emotional exhaustion among health workers were also similar across arms. All domains of stigma reduced gradually over the course of the trial.

Ours was a large study, nested within a cluster randomized trial, in which we recruited representative samples of populations of interest and deployed best-practice measures of stigma. Nevertheless, a few limitations must be considered. First, the sample size was lower than planned among PC-HIV− participants and inter-cluster variation was high, underlying wide confidence intervals. Second, men were underrepresented in the population cohort [22] and health worker cohorts. However, we did not find gender imbalances between trial arms, and found no difference in the impact of PopART on stigma outcomes between women and men. Third, the stigma measures utilized were valid and based on current stigma theory [27]. However, stigma measurement is complex, and we were only able to include a few items for each stigma domain.

Finally, there was evidence of baseline imbalance in stigma. We accounted for this in our analysis, adjusting for baseline stigma in each community. As PC0 recruitment occurred in a similar time frame to the first round of intervention delivery in arm A, it is possible that the baseline imbalance reflects a short-term effect of the intervention on stigma that then waned over time. It is also plausible that other dynamics, such as reporting biases because of which intervention arm communities were randomly assigned to, may have contributed to this measured imbalance. Interestingly, in a post hoc time trend analysis, stigma reductions were steeper in our main outcome in arm C than arms A and B, taking them from the higher starting point to more similar levels at endline.

We hypothesized that the UTT strategy might reduce HIV stigma through the process of ‘normalization’ of HIV in communities. More people getting on treatment and improving their health, combined with the presence of community health workers providing door-to-door HIV testing, might have reduced negative attitudes and stigmatizing behaviours towards PLHIV. However, we also considered it possible that PopART could increase stigma. For example, we could have seen increases in negative attitudes towards PLHIV among health workers as they experienced heavier workloads from newly diagnosed clients living with HIV, and among community members if access to services was perceived to have reduced. Yet, stigma did not increase in this way, suggesting that a population-level, UTT intervention had limited impact on stigma among PLHIV, community members or health workers.

One possible explanation for the lack of an effect of PopART on HIV stigma was the absence of intensive stigma-reduction components. Interventions to reduce HIV stigma must address the drivers and manifestations of stigma at multiple levels and with multiple populations [7,17,18,30]. Further analysis of qualitative and quantitative data may enhance our understanding of how PopART influenced stigma in our study populations, particularly across communities, and which domains of stigma may be more critical to intervene on.

Other researchers have reported gradual reductions in stigma as HIV normalizes and ART becomes more widely available [31]. In our study, such reductions appeared to be significant but slow, and a high prevalence of stigma was still present at the end of the trial, with one in four PLHIV reporting experience of stigma in the last 12 months. ‘Normalization’ alone may be insufficient to reach the global zero discrimination target [32]. Our finding that a combination HIV prevention intervention with universal testing and treatment did not reduce HIV stigma and discrimination among PLHIV, community members or health workers, alongside a lack of evidence for rapid declines in HIV stigma in any arm of the study, suggests the need for additional strategies to reach the global zero discrimination targets.

## Acknowledgements

The authors wish to thank the research participants in the 21 study communities and the participating health facilities, the members of the community advisory boards, and the study implementing partners including the Ministry of Health in Zambia and Department of Health in South Africa without whom this study would not have been possible. HPTN 071 (PopART) is sponsored by the National Institute of Allergy and Infectious Diseases (NIAID) under Cooperative Agreements UM1-AI068619, UM1-AI068617 and UM1-AI068613, with funding from the US President's Emergency Plan for AIDS Relief (PEPFAR). Additional funding is provided by the International Initiative for Impact Evaluation (3ie) with support from the Bill & Melinda Gates Foundation, as well as by NIAID, the National Institute on Drug Abuse (NIDA) and the National Institute of Mental Health (NIMH), all part of NIH. The stigma ancillary study was funded by NIMH. The content is solely the responsibility of the authors and does not necessarily represent the official views of the NIAID, NIMH, NIDA, PEPFAR, 3ie or the Bill & Melinda Gates Foundation.

J.M.H., A.S., and T.P. were members of the STRIVE consortium, which produced research on the structural drivers of HIV, including stigma. The STRIVE consortium was funded by UKaid from the Department for International Development (http://strive.lshtm.ac.uk/). However, the views expressed do not necessarily reflect the department's official policies. R.H. and S.F. received funding support from the U.K. Medical Research Council and the U.K. Department for International Development (DFID) (MR/R010161/1) under the MRC/DFID Concordat agreement, which is also part of the second program of the European and Developing Countries Clinical Trials Partnership supported by the European Union.

^∗^The HPTN 071 (PopART) Study Team author contributors: A.S., J.H., V.B., and G.H. conceptualized the manuscript. T.P. and M.S. conducted the analysis. T.M., C.M., and T.P. oversaw in-country data collection. N.B-.M., R.D., A.Sc., and D.D. managed the PC data and T.M., C.M., and T.P. managed the healthcare worker data sets. A.S. led the manuscript writing. T.P., T.M., M.S., C.M., L.V., S.F., V.B., G.H., R.H., H.A., P.B., D.D., N.B., and J.H. were involved in drafting the manuscript and provided critical feedback on the full manuscript. All authors read and approved the final manuscript. R.H., P.B., H.A., and S.F. were principal investigators of the HPTN 071 PopART trial within which this study was nested.

### Conflicts of interest

There are no conflicts of interest.

## Supplementary Material

Supplemental Digital Content

## Supplementary Material

Supplemental Digital Content

## Supplementary Material

Supplemental Digital Content

## Supplementary Material

Supplemental Digital Content

## Supplementary Material

Supplemental Digital Content

## Supplementary Material

Supplemental Digital Content

## Supplementary Material

Supplemental Digital Content

## Supplementary Material

Supplemental Digital Content
