# Supplementary material for: The effect of universal testing and treatment on HIV stigma in 21 communities in Zambia and South Africa
Source: AIDS. 2020 Aug 6;34(14):2125–35. doi: 10.1097/QAD.0000000000002658 (PMC8425632; doi:10.1097/QAD.0000000000002658)
Supplement: Supplemental Digital Content [file aids-34-2125-s001.pdf]

Protocol

This trial protocol has been provided by the authors to give readers additional information about their work.

Protocol for: Hayes RJ, Donnell D, Floyd S, et al. Effect of universal testing and treatment on HIV incidence — HPTN 071 (PopART). N Engl J Med 2019;381:207-18. DOI: 10.1056/NEJMoa1814556

HPTN 071
Population Effects of Antiretroviral Therapy to Reduce HIV Transmission (PopART): A cluster-randomized trial of the impact of a combination prevention package on population-level HIV incidence in Zambia and South Africa

This supplement contains the following items:

1. Original protocol (Version 1.0, 26 Oct 2012)
2. Final protocol (Version 3.0, 16 Nov 2015)
3. Summary of protocol changes
4. Original statistical analysis plan (Version 1.0, 14 Apr 2016)
5. Final statistical analysis plan (Version 3.0, 16 Dec 2018; includes a section on pages 396-398 describing the cumulative changes in the SAP)

Date: 16 December 2018

PROTOCOL VERSION 1.0

26 OCT 2012

HPTN 071
Population Effects of Antiretroviral Therapy to Reduce HIV Transmission (PopART): A cluster-randomized trial of the impact of a combination prevention package on population-level HIV incidence in Zambia and South Africa

A Study of the HIV Prevention Trials Network

DAIDS ID:
11865

Sponsored by:
Division of AIDS, National Institute of Allergy and Infectious Diseases
U.S. National Institutes of Health

Funded by:
National Institute of Allergy and Infectious Diseases
National Institute of Mental Health
Office of the United States Global AIDS Coordinator
Bill and Melinda Gates Foundation
U.S. National Institutes of Health

Protocol Chair:
Richard Hayes
London School of Hygiene & Tropical Medicine
London, United Kingdom

Protocol Co-Chair:
Sarah Fidler
Imperial College
London, United Kingdom

Final Version 1.0
26 October 2012

Non-IND Study

HPTN 071
Population Effects of Antiretroviral Therapy to Reduce HIV Transmission (PopART): A cluster-randomized trial of the impact of a combination prevention package on population-level HIV incidence in Zambia and South Africa

TABLE OF CONTENTS

LIST OF TABLES AND FIGURES 6
LIST OF ABBREVIATIONS AND ACRONYMS 7
PROTOCOL TEAM ROSTER 9
INVESTIGATOR SIGNATURE PAGE 13
SCHEMA 14
OVERVIEW OF STUDY DESIGN AND RANDOMIZATION SCHEME 17
1.0 INTRODUCTION 18
1.1 Background and Prior Research 18
1.2 Rationale 18
1.2.1 The HIV Epidemic in Sub-Saharan Africa 19
1.2.2 HIV Prevention Methods 20
1.2.3 Anti-Retroviral Therapy (ART) for HIV Prevention 22
1.2.4 Innovation 23
2.0 STUDY OBJECTIVES AND DESIGN 26
2.1 Primary Objective 26
2.2 Secondary Objectives 26
2.3 Study Design 28
2.4 Timing of Deployment of Intervention and Research Components 30
2.5 Cross-Sectional HIV Incidence Estimation 32
3.0 STUDY INTERVENTION 32
3.1 Implementation Team Experience 32
3.2 Description of CHiP Teams 33
3.3 Universal HIV Testing and Linkage to Care 33
3.4 Male Circumcision 34
3.5 Universal Treatment (Arm A) 34
3.5.1 Choice of ART Regimen (Arm A) 35
3.6 Treatment According to National Guidelines (Arms B & C) 35
3.7 Prevention of Mother-to-Child Transmission 36
3.8 Management of Sexually Transmitted Infections 36
3.9 Provision of PrEP 36
3.10 Standard of Care 36
3.11 Delivery of Intervention 37
3.11.1 Activities with Local Health Centers/Community Institutions 37
3.11.2 Collaborations 38
3.12 Monitoring and Evaluation Plan 40
4.0 STUDY POPULATION 41
4.1 Description/Selection of the 21 Study Communities 41
4.2 Randomization 44

4.3 Community Engagement 44
5.0 RESEARCH PROCEDURES AND ACTIVITIES 45
5.1 *Population Cohort* 46
5.1.1 Sampling/Recruitment of *Population Cohort* 46
5.1.2 Inclusion Criteria *Population Cohort* 46
5.1.3 Exclusion Criteria *Population Cohort* 46
5.1.4 Procedures and Activities 47
5.1.5 Reviewing Health Center Records for *Population Cohort* 47
5.1.6 Retention in *Population Cohort* 48
5.2 *Population Cross-Sectional Survey* (if funded) 49
5.2.1 Sampling/Recruitment of *Population Cross-Sectional Survey* Participants 49
5.2.2 Inclusion Criteria 49
5.2.3 Exclusion Criteria 49
5.2.4 Procedures and Activities 49
5.3 *Case-Control Studies* 50
5.3.1 *Case-Control Study 1* - Uptake of Testing in the First Round of Home-Based Testing Provided by CHiP Teams in Arms A & B 50
5.3.1.1 Sampling/Recruitment of Case-Control Study 1 Participants 51
5.3.1.2 Inclusion Criteria Case-Control Study 1 51
5.3.1.3 Exclusion Criteria Case-Control Study 1 51
5.3.1.4 Procedures and Activities 51
5.3.2 *Case-Control Study 2* - Uptake of Immediate Treatment in Arm A 51
5.3.2.1 Sampling/Recruitment of Case-Control Study 2 Participants 52
5.3.2.2 Inclusion Criteria Case-Control Study 2 52
5.3.2.3 Exclusion Criteria Case-Control Study 2 52
5.3.2.4 Procedures and Activities 52
5.3.3 *Case-Control Study 3* - Uptake of Testing in the Second Round of Home-Based Testing Provided by CHiP Teams in Arms A & B 52
5.3.3.1 Sampling/Recruitment of Case-Control Study 3 Participants 53
5.3.3.2 Inclusion Criteria Case-Control Study 3 53
5.3.3.3 Exclusion Criteria Case-Control Study 3 53
5.3.3.4 Procedures and Activities 53
5.4 *Qualitative Studies* 53
5.4.1 Evaluation of the Acceptability of the Intervention: 54
5.4.2 Qualitative Longitudinal Study in Arms A and B – sub-set of *Case-Control Study 1*. 55
5.4.3 Ethnography of the HIV landscape 55
5.4.4 Graphical Summary of Qualitative Activities 55
5.4.5 Integration of Data from Case-Control and Qualitative Components 56
5.5 Collection of Health Center-Based Data 56
5.5.1 Tuberculosis Case Notification 56
5.5.2 Intervention Effect on Health Center Workload 57
5.5.3 Intervention Effect on Healthcare Costs 57
5.5.4 HIV Disease Progression and Death 57
5.5.5 ART Adherence and ART Toxicity 57
5.5.6 Uptake of Intervention Components 58
5.6 Proposed Additional Surveys 58
5.7 Comparative Table of Study Activities across All Study Arms 60

6.0 SAFETY MONITORING AND SOCIAL HARM REPORTING 61
6.1 Safety Monitoring 61
6.2 Social Harm Reporting 61
6.2.1 Participants in the *Population Cohort* 62
6.2.2 Community at Large 62
6.2.3 Social Harm Monitoring 62
7.0 STATISTICAL CONSIDERATIONS AND DATA ANALYSIS 63
7.1 Sample Size 63
7.1.1 Mathematical Modeling and Sample Size Calculations 63
7.1.2 Primary Endpoint - HIV Incidence Over 36 Months 66
7.1.3 Secondary Endpoints 70
7.2 Random Assignment / Study Arm Assignment 74
7.3 Statistical Analysis 74
7.4 Interim Evaluation 75
7.4.1 HIV incidence 75
7.4.2 Uptake of intervention 76
7.5 Mathematical Modeling 76
7.6 Outcomes for Secondary Objectives 77
7.7 Secondary Objectives for Case-Control Studies 79
7.8 Secondary Objectives for Qualitative Studies 80
7.9 Secondary Objectives Related to Economic Evaluation 80
7.10 Process Measures 85
7.11 Tabular Summary of Outcomes 86
8.0 HUMAN SUBJECTS CONSIDERATIONS 90
8.1 Collaborative Partnerships 90
8.2 Social Value 90
8.3 Scientific Validity 90
8.4 Fair Subject Selection 91
8.5 Risk-Benefit Assessment 91
8.5.1 Community Level 92
8.5.1.1 Benefits 92
8.5.1.2 Risks 92
8.5.1.3 Minimizing Risks to Communities 93
8.5.1.4 Risk-Benefit Assessment at Community Level 93
8.5.2 Individual Level 93
8.5.2.1 Benefits 93
8.5.2.2 Risks 94
8.5.2.3 Minimizing Risks to Individuals 94
8.5.2.4 Risk- Benefit Assessment at Individual level 96
8.6 Informed Consent 96
8.6.1 Approval from Respective Authorities 96
8.6.2 ‘Community’ Consent 96
8.6.3 Individual Consent 97
8.6.4 Waiver of Individual Consent to Access CHiP and Routine Clinic Data 98
8.7 Independent Ethical Review 99
8.8 Respect for Participants and Communities During and After the Study 99
8.8.1 Confidentiality 99
8.8.2 Data and Safety Monitoring Plan 100

8.8.3 Communicable Disease Reporting Requirements 100
8.8.4 Post-Trial Management of Participants Exposed to the Early ARV Intervention . 100
8.8.5 Study Discontinuation 101
9.0 LABORATORY SPECIMENS AND BIOHAZARD CONTAINMENT 101
9.1 Local Laboratory Specimens 101
9.2 Network Laboratory Specimens 101
9.3 Quality Control and Quality Assurance Procedures 102
9.4 Specimen Storage and Possible Future Research Testing 102
9.5 Biohazard Containment 103
10.0 ADMINISTRATIVE PROCEDURES 103
10.1 Protocol Registration 103
10.2 Study Activation 103
10.3 Study Coordination 104
10.4 Study Monitoring 104
10.5 Protocol Compliance 105
10.6 Investigator's Records 105
10.7 Use of Information and Publications 105
11.0 REFERENCES 106
12.0 APPENDICES 112
APPENDIX I - SCHEDULES OF STUDY VISITS AND PROCEDURES 113
APPENDIX II - SAMPLE INFORMED CONSENT FORM – POPULATION COHORT 116
APPENDIX III - SAMPLE INFORMED CONSENT FORM – QUALITATIVE STUDIES PARTICIPANTS 123
APPENDIX IV - SAMPLE INFORMED CONSENT FORM – CASE CONTROL STUDIES PARTICIPANTS 129
APPENDIX V - SAMPLE INFORMED CONSENT FORM – ARM A PARTICIPANTS STARTING ART IMMEDIATELY 134
APPENDIX VI - SAMPLE INFORMED CONSENT FORM – CHIP TEAM ACTIVITIES ... 140
APPENDIX VII – SAMPLE SIZE CALCULATIONS 145
APPENDIX VIII - POPULATION CROSS-SECTIONAL SURVEY 156
APPENDIX IX- TB PREVALENCE SURVEY 160
1.1 Inclusion Criteria 160
1.2 Exclusion Criteria 160
1.3 Visit Procedures for the *TB Prevalence Survey* (Month 36 only) 160
APPENDIX X- PMTCT SURVEY 163
1.1 Inclusion Criteria 163
1.2 Exclusion Criteria 163
1.3 Visit Procedures for the *PMTCT Survey* (Month 36 only) 163

HPTN 071
Population Effects of Antiretroviral Therapy to Reduce HIV Transmission (PopART): A cluster-randomized trial of the impact of a combination prevention package on population-level HIV incidence in Zambia and South Africa

LIST OF TABLES AND FIGURES

List of Tables

Table 1- Summary of Intervention Components ... 38
Table 2- Twenty one proposed study clusters in Zambia and South Africa and relevant background data ... 42
Table 3- Study Activities across All Study Arms ... 60
Table 4- Parameter values assumed for the model of the impact of the intervention for central and optimistic target scenarios, and projected impact on HIV incidence in Arms A and B compared with Arm C, assuming intervention roll-out over a 6-month time period ... 65
Table 5- Parameter values assumed for the model of the impact of the intervention for central and optimistic target scenarios, and projected impact on HIV incidence in Arms A and B compared with Arm C, assuming intervention roll-out over a 12-month time period ... 66
Table 6- Power for comparison of HIV incidence in Arm A or B with Arm C, with 7 communities per arm and Population Cohort of 2500 adults per community (assuming that on average 2125 (85%) will be HIV-uninfected at baseline and that loss to follow-up will be 20% after 2 years and 25% after 3 years) with 5206 person-years per community over 36 months (assuming 1912 person-years 0-12 months; 1700 person-years 12-24 months; 1594 person-years 24-36 months) ... 68
Table 7- Power for comparison of HIV incidence between Arms A and B, with 7 communities per arm and Population Cohort of 2500 adults per community (assuming that on average 2125 (85%) will be HIV-uninfected at baseline and that loss to follow-up will be 20% after 2 years and 25% after 3 years) ... 69
Table 8- Case-control studies of (1) uptake of HIV testing (2) uptake of re-testing for HIV and (3) uptake of immediate ART among HIV-positive individuals ... 73
Table 9- Summary, outcomes for secondary objectives related to the economic analysis ... 83
Table 10- Summary of Study Objectives and Related Outcomes ... 87

List of Figures

Figure 1- Community Randomization Scheme, Zambia and South Africa ... 29
Figure 2- Timing of Deployment of Intervention and Research Components ... 31
Figure 3- Location of 21 clusters in Zambia and South Africa ... 43
Figure 4- Qualitative Activities in HPTN 071 ... 56
Figure 5- Mathematical model of the epidemic and of the PopART interventions ... 64

LIST OF ABBREVIATIONS AND ACRONYMS

AE	Adverse Event
AIDS	Acquired Immunodeficiency Syndrome
ANC	Antenatal Clinic
ART	Anti-Retroviral Therapy
ARV	Anti-Retroviral
CDC	Centers for Disease Control and Prevention
CFR	Code of Federal Regulations
CHiPs	Community HIV-care Providers
CORE	(HPTN) Coordinating and Operations Center
DAIDS	Division of AIDS
DALY	Disability-Adjusted Life-Year
DSMB	Data Safety Monitoring Board
EC	Ethics Committee
EQA	External Quality Assurance
FDA	(United States) Food and Drug Administration
FHCRC	Fred Hutchinson Cancer Research Center
GCLP	Good Clinical Laboratory Practice
GCP	Good Clinical Practice
HCT	HIV Counseling and Testing
HIV	Human Immunodeficiency Virus
HPTN	HIV Prevention Trials Network
HSV-2	Herpes Simplex Virus, Type 2
IATA	International Air Transport Association
ICF	Informed Consent Forms
IRB	Institutional Review Board
LDMS	Laboratory Data Management System
MSM	Men who have Sex with Men
NB	Nota Bene
NIAID	(United States) National Institute of Allergy and Infectious Diseases
NIH	(United States) National Institutes of Health
NL	(HPTN) Network Laboratory
PMTCT	Prevention of Mother to Child Transmission of HIV
PrEP	Pre-Exposure Prophylaxis
PRO	Protocol Registration Office
pSMILE	Patient Safety Monitoring and International Laboratory Evaluation
QA	Quality Assurance
QALY	Quality-Adjusted Life-Year
QC	Quality Control
RE	Regulatory Entity

RNA	Ribonucleic Acid
RR	Rate Ratio
RSC	Regulatory Services Center
SAE	Serious Adverse Event
SANAC	South African National AIDS Council
SDMC	(HPTN) Statistical and Data Management Center
SMC	Study Monitoring Committee
SMS	Short Message Service
START	Selection of Thymidine Analog Regimen Therapy Study
STI	Sexually Transmitted Infection
SSP	Study Specific Procedures
TB	Tuberculosis
UK	United Kingdom
UNAIDS	United Nations Programme on HIV/AIDS
US	United States (of America)
UTT	Universal Testing and Treatment
VCT	Voluntary Counseling and Testing
WHO	World Health Organization
ZAMBART	Zambia AIDS Related Tuberculosis Project
ZAMSTAR	Zambia-South Africa TB and AIDS Reduction Program

HPTN 071
Population Effects of Antiretroviral Therapy to Reduce HIV Transmission (PopART): A cluster-randomized trial of the impact of a combination prevention package on population-level HIV incidence in Zambia and South Africa

PROTOCOL TEAM ROSTER

Helen Ayles
Clinical Scientist/Site Principal Investigator
ZAMBART
University of Zambia
School of Medicine, Ridgeway Campus
Lusaka, Zambia
Dept of Clinical Research
London School of Hygiene and Tropical Medicine
Keppel Street
London WC1E 7HT, UK
Phone: +260 211 254710, 260 211257215
Fax: +260 211 254710
Email: helen@zambart.org.zm

Megan Baldwin
Project Manager
SCHARP-FHCRC
1100 Fairview Avenue North
E3-129
PO Box 19024
Seattle, WA 09109 USA
Phone: 206-667-4378
Email: mmbaldwi@scharp.org

Nulda Beyers
Clinical Scientist/Site Principal Investigator
Desmond Tutu TB Centre
Stellenbosch University
Francie van Zijl Avenue
Clinical Building, K floor, Room 0065
Tygerberg Campus
Western Cape, 7505, South Africa
Phone: +21 938 9114
Fax: + 21 938 9719
Email: nb@sun.ac.za

Peter Bock
Clinical Scientist/SiteLead Investigator
Desmond Tutu TB Centre
Stellenbosch University
Francie van Zijl Avenue
Clinical Building, K floor, Room 0065
Tygerberg Campus
Western Cape, 7505, South Africa
Phone: +27 835 721470
Email: peterb@sun.ac.za

Virginia Bond
Social Scientist
London School of Hygiene & Tropical Medicine
ZAMBART Project, School of Medicine
P.O. Box 50697
Lusaka, Zambia
Phone: +260 211 254710
Email: gbond@zambart.org.zm

David Burns
Prevention Sciences Branch Chief
DAIDS Medical Officer
NIAID/NIH
6700B Rockledge Drive
Bethesda MD 20892, USA
Phone: +001-301-435-8896
Email: burnsda@niaid.nih.gov

Nathaniel Chishinga
Clinical Scientist
ZAMBART Project, School of Medicine
PO Box 50697
Lusaka, Zambia
ZAMBIA
Phone: +260 211 254710
Fax: +260 211 254710
Email: nathaniel@zambart.org.zm

Deborah Donnell
SDMC Biostatistician
SCHARP-FHCRC
1100 Fairview Ave. North, LE-400
PO Box 19024
Seattle, WA 98109, USA
Phone: +001-206-667-5661
Fax: +001-206-667-4812
Email: deborah@scharp.org

Lynda Emel
Research Program Manager
SCHARP-FHCRC
1100 Fairview Ave. North, E-129
PO Box 19024
Seattle, WA 98109, USA
Phone: +001-206-667-5803
Email: lemel@scharp.org

Susan Eshleman
HPTN Network Laboratory
Johns Hopkins University School of Medicine
720 Rutland Ave.
Ross Building, Room 646
Baltimore MD, 21205, USA
Phone: +001-410-614-4734
Fax: +001-410-502-9244
Email: seshlem@jhmi.edu

Sarah Fidler
Protocol Co-Chair
Imperial College London
CSL in Communicable Diseases
St Mary's Campus
HIV Clinical Trials Unit
Winston Churchill Wing
London W2 1NY, UK
Phone: +44 (0) 203 312 6790
Email: s.fidler@imperial.ac.uk

Sian Floyd
Statistician
Department of Infectious Disease Epidemiology
London School of Hygiene & Tropical Medicine
Keppel Street
London, WC1E 7HT, UK
Phone: +44-207-6127888
Fax: +44-207-6368739
Email: sian.floyd@lshtm.ac.uk

Christophe Fraser
Mathematical Modeler
Imperial College London
Department of Infectious Disease Epidemiology
St Mary's Campus
Norfolk Place
London W2 1PG, UK
Phone: +44 (0) 20 75943397
Email: c.fraser@imperial.ac.uk

Peter Godfrey-Faussett
Professor of International Health
Room 303b
London School of Hygiene & Tropical Medicine
Keppel Street
London WC1E 7HT
Phone: +44 020 7958 8127
Fax: +44 020 7612 7860
Email: peter.godfrey-faussett@lshtm.ac.uk

Sam Griffith
Senior Clinical Research Manager
FHI360
2224 E NC Hwy 54
Durham NC 27713, USA
Phone: +001-919-544-7040 ext. 11571
Email: sgriffith@fhi360.org

James Hargreaves
Senior Lecturer in Epidemiology
Department of Infectious Disease Epidemiology
London School of Hygiene & Tropical Medicine
Keppel Street
London WC1E 7HT, UK
Phone: +44 (0)20 7927 2955
Fax: +44 (0)20 7637 4314
Email: james.hargreaves@lshtm.ac.uk

Katharina Hauck
Health Economist
Imperial College Business School
Tanaka Building
South Kensington Campus
London SW7 2AZ, UK
Phone: +44 (0)20 7594 9197
Fax: +44 (0)20 7594 9189
Email: k.hauck@imperial.ac.uk

Richard Hayes
Protocol Chair, Lead Statistician
Department of Infectious Disease Epidemiology
London School of Hygiene & Tropical Medicine
Keppel Street
London WC1E 7HT, UK
Phone: +44 (0)20 7927 2243
Fax: +44 (0)20 7637 4314
Email: richard.hayes@lshtm.ac.uk

Tanette Headen
Research Assistant II
FHI360
2224 E NC Hwy 54
Durham, NC 27713
Phone: +001-919-544-7040 ext. 11297
Email: theaden@fhi360.org

Lyn Horn
Ethicist
Desmond Tutu TB Centre
Division Research Development
Stellenbosch University
Phone: +27 21 808 2670
Email: lhorn@sun.ac.za

Corey Kelly
Project Manager
SCHARP-FHCRC
1100 Fairview Ave. North, E-129
PO Box 19024
Seattle, WA 98109, USA
Phone: 206-667-5170
Fax: 206-667-4812
E-mail: ckelly@scharp.org

Peter Kim
Medical Officer
TB Clinical Research Branch
Division of AIDS, NIAID, NIH
6700B Rockledge Dr.
Room 5222
Bethesda MD 20892, USA
Phone: +001-301-451-2761
Fax: +001-301-480-4582
Email: kimp2@niaid.nih.gov

Estelle Piwowar-Manning
HPTN Network Laboratory
600 North Wolfe St.
Pathology 306
Johns Hopkins University
Baltimore, MD 21287, USA
Phone: +001-410-614-6736
Fax: +001-410-614-0430
Email: epiwowa@jhmi.edu

Ayana Moore
Senior Clinical Research Manager
FHI360
2224 E NC Hwy 54
Durham NC 27713, USA
Phone: +001-919-544-7040 ext. 11244
Email: amoore@fhi360.org

Kalpana Sabapathy
Clinical Epidemiologist
Department of Infectious Disease Epidemiology
London School of Hygiene & Tropical Medicine
304-B, Keppel Street
London WC1E 7HT, UK
Phone: +44 7927 2155
Fax: +44 (0)20 7637 4314**Email:** kalpana.sabapathy@gmail.com

Ab Schaap
Senior Data Manager
ZAMBART Project
London School of Hygiene & Tropical Medicine
Ridgeway Campus
P.O. Box 50697
10101 Lusaka, Zambia
Phone: +260 211 254710, Mobile: +260 977 442965
Fax: : +260-211-254710
Email: *ab@zambart.org.zm*

Kwame Shanaube
PopART Study Manager - Zambia
ZAMBART Project
PO Box 50697
Ridgeway Campus
Lusaka, Zambia
ZAMBIA
Phone : +260-211-254710/+260-211-257215
Fax : +260-211-254710
Email : kshanaube@zambart.org.zm

Peter C. Smith
Health Economist
Imperial College Business School
Centre for Health Policy
Professor of Health Policy
Exhibition Road
London SW7 2AZ, UK
Phone: +44 (0)20 7594 1904
Fax: not available
Email: peter.smith@imperial.ac.uk

Sten Vermund
HPTN Executive Committee Liaison
Vanderbilt University, Institute for Global Health
2525 West End Ave.
Suite 750
Nashville TN 32703, USA
Phone: +001-615-322-9374 (office); +001-615-720-3677 (mobile); +001-615-322-9015 (Asst: Jen St. Clair)
Fax: +001-615-343-7797
Email: sten.vermund@vanderbilt.edu

Deborah Watson-Jones
Clinical Epidemiologist
Department of Clinical Research
London School of Hygiene & Tropical Medicine
Mwanza Intervention Trials Unit
National Institute for Medical Research
P.O. Box 11936
Mwanza, Tanzania
Phone: +255 (0) 28250 0019
Email: deborah.watson-jones@lshtm.ac.uk

Rhonda White
Community Program Manager
FHI360
2224 E NC Hwy 54
Durham NC 27713, USA
Phone: +001-919-544-7040 ext. 11515
Email:* *rwhite@fhi360.org

HPTN 071
Population Effects of Antiretroviral Therapy to Reduce HIV Transmission (PopART): A cluster-randomized trial of the impact of a combination prevention package on population-level HIV incidence in Zambia and South Africa

Final Version 1.0
26 October 2012

INVESTIGATOR SIGNATURE PAGE

A Study of the HIV Prevention Trials Network (HPTN)

Sponsored by:
Division of AIDS, National Institute of Allergy and Infectious Diseases
U.S. National Institutes of Health

Funded by:
National Institute of Allergy and Infectious Diseases
National Institute of Mental Health
Office of the United States Global AIDS Coordinator
Bill and Melinda Gates Foundation
US National Institutes of Health

I, the Investigator of Record, agree to conduct this study in full accordance with the provisions of this protocol. I agree to maintain all study documentation for a minimum of three years after submission of the site's final Financial Status Report to the Division of AIDS (DAIDS), unless otherwise specified by DAIDS or the HIV Prevention Trials Network (HPTN) Coordinating and Operations Center. Publication of the results of this study will be governed by HPTN policies. Any presentation, abstract, or manuscript will be made available by the investigators to the HPTN Manuscript Review Committee and DAIDS for review prior to submission.

I have read and understand the information in this protocol and will ensure that all associates, colleagues, and employees assisting in the conduct of the study are informed about the obligations incurred by their contribution to the study.

____
Name of Investigator of Record

__________________________________ __________________________________
Signature of Investigator of Record Date

HPTN 071
Population Effects of Antiretroviral Therapy to Reduce HIV Transmission (PopART):
A cluster-randomized trial of the impact of a combination prevention package on population-level HIV incidence in Zambia and South Africa

SCHEMA

Purpose: The purpose of this study is to determine the impact of two community-level combination prevention packages, both of which include universal HIV testing and intensified provision of HIV antiretroviral therapy (ART) and care, on population-level HIV incidence.

Design: This is a three-arm, cluster-randomized, longitudinal study to be implemented in 21 clusters (communities).

Study Population: The prevention packages will be implemented throughout the communities randomized to the intervention arms. Main study outcomes will be measured in a randomly-selected group drawn from the adult population of the communities: a *Population Cohort*.

Study Size: The combined population of all 21 clusters is approximately 1.2 million individuals. The interventions will be implemented in 14 of the 21 clusters with a combined population of approximately 800,000 individuals (adults and children) in the intervention arms. The approximate sizes of the randomly-selected groups for main study outcome assessments are:

- *Population Cohort*: 52,500 individuals
- *Case-Control Studies*: 2,400 individuals
- *Qualitative Studies*: about 2,000 individuals
- *Population Cross-Sectional Survey*: 10,500 individuals (if funded)
- *TB Survey*: about 94,500 individuals (if funded)
- *PMTCT Survey*: about 25,200 individuals (if funded)

Study Arms/Interventions:

Arm A - Universal Testing with Immediate ART:

- Combination prevention package including:
  - House-to-house deployment of:
    - Universal HIV counseling and testing
    - Active linkage to care for individuals diagnosed as HIV-infected, with ***immediate eligibility for ART***
    - Promotion of male circumcision and prevention of mother to child transmission (PMTCT) services
    - Provision of condoms
  - Strengthening of HIV testing and services at health facilities and other venues
  - Strengthening of male circumcision and prevention of mother-to-child transmission of HIV services available in the community
  - Treatment of sexually transmitted infections (STIs) and provision of condoms at health units

Arm B - Universal Testing with ART Eligibility According to National Guidelines:

- Combination prevention package including:
  - House-to-house deployment of:
    - Universal HIV counseling and testing
    - Active linkage to care for individuals diagnosed as HIV-infected, with ***ART eligibility according to national guidelines***
    - Promotion of male circumcision and PMTCT services
    - Provision of condoms
  - Strengthening of HIV testing and services at health facilities and other venues
  - Strengthening of male circumcision and PMTCT services available in the community
  - Treatment of STIs and provision of condoms at health units

Arm C - Standard of Care (Control Arm)

- Strengthening of HIV testing and ART services according to national guidelines at health facilities and other venues
- Strengthening of male circumcision and PMTCT services available at health facilities and other venues in the community
- Treatment of STIs and provision of condoms at health facilities and other venues in the community

Study Duration: The planned duration of the entire study will be approximately 6 years, with enrollment and follow-up of communities and delivery of the intervention occurring over 4 years. Assessment of the primary outcome (HIV incidence) in the *Population Cohort* is planned to take place 12, 24, and 36 months after recruitment. Interim evaluation will take place during the first two years of intervention to determine whether to continue with the 36 month follow-up of the *Population Cohort* and the fourth year of intervention.

Primary Objective:

- To measure the impact of the two intervention packages on HIV incidence by enrolling and following a random sample of adults (the *Population Cohort*) in the trial communities for 3 years

Secondary Objectives:

- Measure the impact of the two intervention packages on the following:
  - HIV incidence over the first, second, and third years of follow-up
  - Community viral load (if funding is identified)
  - ART adherence and viral suppression (if funding is identified)
  - ART drug resistance (if funding is identified)
  - HSV-2 incidence
  - Uptake of HIV testing and retesting over the entire study period
  - ART screening and uptake
  - Time between HIV diagnosis and initiation of care
  - Retention in care
  - HIV disease progression and death
  - ART toxicity based on clinic records
  - Sexual risk behavior
  - Case notification rate of tuberculosis
  - HIV-related stigma
  - Uptake of PMTCT
  - Uptake of male circumcision
- Carry out case-control studies to examine factors related to:
  - Uptake of HIV testing during the first round of home-based testing in Arms A and B
  - Uptake of immediate treatment in Arm A

    - Uptake of HIV testing during the second round of home-based testing in Arms A and B
- Use qualitative methods to:
    - Assess popular understanding of HIV testing and treatment at study initiation and during implementation
    - Evaluate the acceptability and functioning of the Community HIV-care Providers (CHiPs) in Arms A & B
    - Evaluate the acceptability of interventions and barriers to access in Arms A & B
    - Document the effect of the interventions on social networks, stigma, sexual behavior, alcohol use, gender-based violence, HIV identity, other HIV prevention options and community morale
    - Evaluate the process and challenges of community consultation and applying ethical principles
- Measure the burden experienced by local health centers due to implementation of the intervention in the community
- Measure the incremental cost of the two intervention packages through systematic recording of costs in intervention and control communities
- Estimate the effectiveness and cost-effectiveness of the intervention packages and alternative packages, both in the chosen study populations and in other populations by fitting mathematical models based on the empirical data from the trial, including data related to cost.

Study Sites: The study is expected to be implemented in the communities identified below.

- The study communities in Zambia are spread across 4 provinces and 5 districts. Each community is the catchment population of a government health facility.
    - Chimwemwe and Ndeke in Kitwe District (Copperbelt Province)
    - Chipulukusu and Chifubu in Ndola District (Copperbelt Province)
    - Makululu and Ngungu in Kabwe District (Central Province)
    - Chawama, Chipata and Kanyama in Lusaka District (Lusaka Province)
    - Maramba and Dambwa in Livingstone District (Southern Province)
    - Shampande in Choma District (Southern Province)

- The study communities in South Africa are located in the Cape Metro District and Cape Winelands District of the Western Cape Province. As above, the communities are defined by the catchment population of a government health facility.
    - Delft South (Metro District)
    - Kuyasa (Metro District)
    - Luvuyo (Metro District)
    - Town II (Metro District)
    - Ikhwezi (Metro District)
    - Bloekombos (Metro District)
    - Dalevale (Cape Winelands District)
    - Wellington (Cape Winelands District)
    - Cloetesville (Cape Winelands District)

HPTN 071
Population Effects of Antiretroviral Therapy to Reduce HIV Transmission (PopART): A cluster-randomized trial of the impact of a combination prevention package on population-level HIV incidence in Zambia and South Africa

OVERVIEW OF STUDY DESIGN AND RANDOMIZATION SCHEME

21 Community Clusters
12 in Zambia / 9 in South Africa
Average of approx. 55,000 individuals in each cluster
Approximately 600,000 total adults across all communities

Randomization

Arm A
Clusters: 4 Zambia / 3 South Africa

Intervention
Combination prevention including:
- universal household-based testing
- active linkage to care
- *immediate* ART eligibility

Research/Evaluation
***Population Cohort* A**
One adult from each of 2,500 randomly-selected households in each cluster

Health Center Data A
Routinely-collected data from Health Centers in the community

CHiPs Data A
Data collected from community members during household visits

Arm B
Clusters: 4 Zambia / 3 South Africa

Intervention
Combination prevention including:
- Universal household-based testing
- active linkage to care
- ART eligibility *according to national guidelines*

Research/Evaluation
***Population Cohort* B**
One adult from each of 2,500 randomly-selected households in each cluster

Health Center Data B
Routinely-collected data from Health Centers in the community

CHiPs Data B
Data collected from community members during household visits

Arm C
Clusters: 4 Zambia / 3 South Africa

Standard of Care
Control Arm:
- Existing prevention & testing services and referral for care
- ART eligibility *according to national guidelines*

Research/Evaluation
***Population Cohort* C**
One adult from each of 2,500 randomly-selected households in each cluster

Health Center Data C
Routinely-collected data from Health Centers in the community

Primary Outcome Measure
- HIV incidence measured over 3 years in ***Population Cohort***

Secondary Outcome Measures
- ***Population Cohort***: HIV incidence measured over 1st, 2nd, and 3rd years, HSV-2 incidence, sexual risk behavior[+], community VL[+]*, viral suppression (ART patients)*[+], drug resistance (ART patients with detectable VL)*[+]
- ***Population Cohort*** and **Health Center Data:** ART Adherence[+], HIV disease progression and death[+], ART toxicity[+], HIV stigma[+]
- **Health Center Data:** TB notification and mortality rates
- ***Population Cohort*, Health Center Data, CHiPs Data:** uptake of PMTCT[+], uptake of male circumcision[+], ART screening and uptake[+], uptake of HIV testing and retesting[+], time between diagnosis and initiation of care[+]

[+] Objectives that will also be addressed by the *Population Cross-Sectional Survey*, if funded
* Pending funding for these assays.

NB: Qualitative and case-control studies that will be undertaken to interpret and inform the results from the objectives above are not included in this diagram for simplicity, but are fully described in subsequent sections.

1.0 INTRODUCTION

1.1 Background and Prior Research

The global health burden associated with human immunodeficiency virus (HIV) infection continues to grow, with an estimated 33 million people living with HIV, including 22.5 million adults and children in sub-Saharan Africa. While several countries have reported reductions in HIV prevalence, prevalence remains extremely high, especially in Southern Africa which continues to experience severe, generalized epidemics with persistently high rates of HIV incidence [1].

While considerable progress has been made in expanding the coverage of antiretroviral treatment (ART) for patients living with advanced disease (CD4 count < 200 cells/µL), a large proportion of HIV-infected individuals who need treatment are not yet receiving it . ART is a lifelong commitment. Therefore, ongoing treatment costs continue to escalate as more patients require ART. There are 2.5 new HIV infections for every HIV-infected patient commencing ART, meaning that there is an ever-expanding pool of patients who will need treatment in the future [1]. Unless the number of new infections can be steeply reduced, it will be increasingly difficult and costly to provide ART for all those who need it [2, 3]. For these reasons, effective HIV prevention has become an even more pressing priority in the era of ART roll-out.

There is increasing recognition that a combination of prevention methods will be needed to bring HIV transmission under effective control in the most severely affected countries, and combination prevention programs are being developed to meet this need [4, 5]. These may involve the provision of proven prevention methods, such as male circumcision[6] and PMTCT [7, 8], a range of behavioral and biomedical interventions specially targeted at those most at risk of infection, and expanded testing and treatment for individuals found to be HIV-infected [9-11]. Early treatment of HIV-infected individuals has been shown to reduce transmission to their sexual partners by 96% [11]. While such strategies are based on sound epidemiological principles, they have not been adequately evaluated in the field [12, 13] and there are no data on their effectiveness or cost-effectiveness in reducing HIV incidence at population level. In particular, identifying specific groups at high risk of HIV infection and providing specially targeted interventions for them is likely to be very difficult to implement on a national scale, and is potentially stigmatizing.

1.2 Rationale

Since the principles of combination HIV prevention were formulated, there has been new interest in the potential impact of universal testing and treatment (UTT) interventions [14]. This concept represents a paradigm shift in HIV prevention, since it focuses on identifying and intervening in HIV-infected individuals in preference to the much larger uninfected population [15]. Even in the high resource environment of the United States, only about a quarter of HIV-infected persons know their status, are linked to care, and are suppressed with ART [16-18]. Mathematical modeling has indicated that if a high proportion of the population can be tested, with those found to be HIV-infected offered immediate ART, HIV infection could be reduced substantially within two years, and potentially eliminated as a public health problem in the longer term [19-26]. While challenging to deliver [27,

28], this approach would nevertheless have major advantages in terms of simplicity and universality, potentially reducing the need for interventions targeting specific groups at high risk of infection, who are often stigmatized, as well as bringing likely clinical benefit to those infected with HIV [29-32].

To guide health policy, data are needed on the population-level impact of different approaches to HIV prevention. We propose to evaluate a combination UTT HIV prevention package that includes universal voluntary HIV testing and counseling, provision of condoms, STI treatment, the offer of male circumcision to men who are HIV uninfected, referral to PMTCT services, and the offer of immediate ART for all those identified as HIV-infected. We will test this package in a cluster-randomized trial in 21 communities in Zambia and South Africa, and measure its impact on HIV incidence in the general population by following a randomly-selected cohort of adults for 3 years. In order to measure the additional impact of offering immediate ART to those who are HIV-infected, the study will have three treatment arms: Arm A will receive the full UTT intervention described above, Arm B will receive the full intervention except that ART will be provided according to current national guidelines, and Arm C will act as a control arm and will receive standard of care services.

Data from the trial will be combined with cost data and mathematical models to estimate the cost-effectiveness of the UTT intervention and alternative intervention approaches in these and other populations.

1.2.1 The HIV Epidemic in Sub-Saharan Africa

Sub-Saharan Africa bears over two-thirds of the worldwide burden of HIV infection [1]. The HIV epidemic in this region has had a devastating effect on morbidity, mortality and national economies, as well as wider societal effects. HIV infection is also a strong risk factor for tuberculosis (TB); people living with HIV who are also infected with TB are about 21–34 times more likely to develop TB disease compared with those who are HIV-negative. Additionally, approximately 24% of global TB deaths are estimated to be HIV-associated, adding to the health burden associated with HIV infection[33] .

While recent declines in HIV prevalence and incidence have been observed in several African countries, HIV prevalence remains extremely high in many parts of the region [1]. In particular, Southern Africa remains severely affected, with an estimated 11.3 million people living with HIV infection, with extensive, generalized HIV epidemics and very high HIV prevalence in most countries. Zambia and South Africa are among the most severely-affected countries with an estimated 980,000 living with HIV in Zambia [34] and an estimated 5,600,000 living with HIV in South Africa [35].

Despite the rapid expansion of access to ART (with an estimated 6.6 million people on ART by the end of 2010), an additional 10 million people are in urgent need of ART in accordance with current World Health Organization (WHO) treatment guidelines[36]. Both countries are making good progress towards achieving the targets set in the new guidelines regarding CD4 count and ART regimens however, there are practical constraints including access to laboratory testing, consistent drug supplies and linkage into care. The HPTN 071 study will work with the local Departments of Health and Ministries

of Health along with the PEPFAR implementing partners, to utilize additional resources to strengthen the health systems.

Globally, it is estimated that there are 2.5 new HIV infections for every patient started on ART. This means that there is an ever-increasing pool of untreated HIV-infected individuals who will need treatment in the next few years in addition to those already on treatment. It is clear that there will be major difficulties in sustaining treatment provision for a continuously expanding number of HIV-infected patients. The expansion of ART services needed in the coming years has increased the urgency for identifying more effective interventions for HIV prevention. Unless HIV incidence can be reduced, an estimated US$35 billion will be needed per year by 2030 to deliver ART to 80% of eligible patients (CD4 <350 cells/µL) in resource-limited settings[37].

1.2.2 HIV Prevention Methods

Very few HIV prevention methods have been shown to be effective in randomized, controlled trials [38, 39]. Behavior change messages have been central to most national acquired immunodeficiency syndrome (AIDS) control programs in Africa, and changes to safer sexual behavior are assumed to have contributed to the reductions in HIV prevalence in Uganda, Zimbabwe, and other countries [40, 41]. However, there is a dearth of evidence from rigorously-designed trials on what specific behavioral interventions bring about the required behavioral changes leading to a reduction in HIV incidence [39]. Similarly, while HIV counseling and testing provide the gateway to key treatment and prevention services, evidence of their effects on behavior and HIV risk is inconclusive [42-44].

In contrast, stronger evidence of effectiveness is available for some biomedical interventions. Male circumcision was shown to reduce HIV incidence by around 60% in three trials in Kenya, South Africa and Uganda [45-47]. Safe services for male circumcision have been recommended for wide-scale roll-out by WHO and the United Nations Programme on HIV/AIDS (UNAIDS), but progress in implementation in many countries has been slow [48, 49]. HIV transmission is known to be facilitated by other sexually transmitted infections (STIs) [50]. One trial in Tanzania showed that improved STI treatment services reduced HIV incidence in the general population [51]. Other trials of a variety of STI interventions in different epidemiological settings have failed to show an impact on HIV incidence [52].

Despite the promising results of the RV144 vaccine trial in Thailand [53], there is general agreement that an effective HIV prophylactic vaccine will not be available for many years [54, 55]. However, the CAPRISA004 trial, reported in 2010, showed that a vaginal gel containing the antiretroviral drug, tenofovir, used periodically, reduced HIV incidence by 39% among women in South Africa [56]. Vaginal microbicides have been shown to be highly acceptable in a wide range of studies, leading to optimism that a product of proven efficacy may achieve substantial coverage [57, 58]. However, further confirmatory and other trials will be needed before this and other microbicides are licensed and available for use in large-scale prevention programs, and it is unclear whether coverage and impact would be sufficient to bring the very high rates of HIV incidence in Southern Africa under control.

Further promising data came from the iPrEx trial in 2010, showing that pre-exposure prophylaxis (PrEP) using a combination of two antiretroviral drugs (emtricitabine and tenofovir) among men who have sex with men (MSM) reduced HIV incidence by 44% in a multi-center study [59]. More recently, two trials of the effects of PrEP on heterosexual transmission among men and women reported a significant protective effect [60, 61], but two trials found no effect [61, 62]. It is currently unclear how the results of these trials will be translated into revised WHO and national guidelines. While such interventions may have a place in HIV prevention programs, particularly for discordant couples, sex workers, and other groups at particularly high risk, the feasibility of wide-scale delivery of PrEP and its population-level impact have been questioned.

Given the limitations in current HIV prevention methods, there is increasing acceptance that effective HIV control in the most severely affected countries in Southern Africa is likely to require the concerted delivery of a combination of partially effective interventions. Combination prevention is therefore becoming the preferred approach to the prevention of HIV infection [4, 5, 9, 63]. Combination prevention packages may consist of different components, including expanded HIV testing and counseling, male circumcision, interventions to promote safer behavior, enhanced PMTCT services, expanded treatment services for HIV-infected patients, and special interventions targeted at groups at increased risk of infection, such as those in HIV-discordant partnerships, injection drug users, commercial sex workers, truck drivers, MSM and others. The emphasis on targeted interventions emerges from the concept of "Know your Epidemic" [64], whereby information on the roles of different modes of transmission and different risk groups in local epidemics helps to guide the most efficient application of limited prevention resources for maximal reduction of HIV transmission.

While the epidemiological basis for combination prevention is strong, there is a need for empirical field studies to evaluate the operational performance of such interventions when applied on a large scale, and to measure their impact on HIV incidence at population level. This would provide valuable data for policy makers who must choose the most appropriate intervention approaches to include in national prevention programs. HPTN 071 will provide such data using a rigorous, cluster-randomized trial design that tests the efficacy of specific combination prevention packages that are strongly supported by epidemiological and modeling data.

One disadvantage of combination prevention strategies that require careful targeting of special groups is that they may be difficult to implement on a wide scale [65]. Optimal implementation of such approaches requires availability of baseline data to define the transmission dynamics and the size and role of different risk groups, development of programs designed specifically for those groups, intensive community liaison work to gain the trust of groups that are often marginalized, stigmatized, or highly mobile, and the management and monitoring of these separate programs. While this may be achievable in demonstration projects in a small number of communities, it may prove very challenging in the context of national roll-out of such programs. In contrast, because the main intervention in HPTN 071 is universal and is offered to the entire community, it will obviate the need for specially-targeted interventions for different risk groups, should help to avoid stigmatization, and should encourage community-wide support for HIV prevention and care.

1.2.3 Anti-Retroviral Therapy (ART) for HIV Prevention

Incident HIV infections necessarily result from transmission of the virus between an HIV-infected index case and an HIV-uninfected individual. This simple observation has led to an increasing interest in interventions focused on HIV-infected persons to prevent transmission to their contacts; this is referred to as positive prevention.

HIV viral load is the key determinant of viral transmission, as demonstrated clearly in observational studies of sexual transmission among HIV-discordant couples; in those studies, no transmission was seen when the index case had a plasma viral load below 1000 copies HIV ribonucleic acid (RNA)/ml [66, 67]. By reducing plasma viral load to undetectable levels (<50 copies HIV RNA/ml), it is assumed that ART will also suppress viral burden in the genital tract to levels at which transmission is unlikely to occur [68, 69]. Although vertical HIV transmission occurs via a different route, proof of concept is provided by trials of PMTCT, which have demonstrated that HIV transmission from mother to child before, during, or after delivery is largely prevented by ART [70-72]. Of even more relevance to sexual transmission, are results of the HPTN 052 trial [73]. In this large, Phase III trial, the effects of early ART on transmission were investigated in 1750 HIV-serodiscordant couples. HPTN 052 was powered to determine the impact of immediate ART initiation for the HIV-infected partner (at CD4 counts >350 cells/µl and <550 cells/µl) on HIV transmission, compared with ART initiation according to standard treatment guidelines [73]. This trial was unblinded early, after demonstrating a 96% reduction in HIV transmission to sexual partners in the early treatment arm, as well as significant reductions in morbidity in HIV-infected index cases [73].

The increasing proportion of HIV-infected patients on ART has likely made some contribution to falling HIV prevalence in some countries. However, ART as currently delivered in resource-poor settings is unlikely to have a substantial effect on HIV transmission because of limited coverage of HIV testing, delays in provision of treatment and – importantly – because much HIV transmission occurs before HIV-infected index cases reach CD4 levels defined by current treatment guidelines [74] . The UTT strategy in this study aims to overcome these limitations by ensuring that all HIV-infected individuals are diagnosed as early as possible, and are provided with ART to lower their viral loads and minimize the risk of transmission. In addition, these interventions will provide important individual-level benefits in terms of reductions in morbidity and mortality among HIV-infected individuals.

Ecological studies have reported promising outcomes of UTT-type interventions at a population level in North America. Among MSM in San Francisco, where 72% uptake of HIV counseling and testing was followed by 95% acceptance of immediate ART for those identified as HIV-infected, an observed reduction in mean and total community viral load was accompanied by a significant decrease in new HIV diagnoses from 798 (in 2004) to 434 (in 2008)[75, 76]. Similarly, among injection drug users in British Columbia, a study of expanded testing and treatment between 1996 and 2009 showed a 52% reduction in estimated HIV incidence [77]. However, the direct relevance of these findings in concentrated epidemics in North America to generalized epidemics in Southern Africa is unclear. While these data are promising, they are subject to many limitations, as they rely on incidence estimates based on diagnosed cases of HIV, and time-trends in HIV

epidemics are notoriously difficult to interpret. No such data are available from sub-Saharan Africa, where the need is greatest.

Interest in the UTT approach to HIV prevention has grown following the publication of mathematical modeling studies suggesting that the approach has the potential to substantially reduce and possibly eliminate HIV transmission at a population level in sub-Saharan Africa. In the much-discussed Granich model [25], ART for all individuals with a CD4 count <350 cells/µL is predicted to reduce population HIV incidence by 30%, based on assumptions about the distributions of plasma viral load and CD4 cell count. In that model, UTT is predicted to reduce the reproduction number to below 1, suggesting that elimination of HIV infection as a public health problem may be possible. However, concerns have been raised about the validity of the assumptions underlying this model, with considerable skepticism about the ability to treat everyone identified as HIV-infected in settings where ART coverage for individuals with CD4 < 200 cells/µL is currently below 50% [78]. The feasibility of the UTT strategy is compromised by weak health systems, insufficient numbers of health care personnel, potential problems with lifelong treatment adherence, drug toxicity, drug resistance and the need for durable second and third-line treatment regimens. The impact of a UTT intervention will also depend on the proportion of transmission events that occur during acute HIV infection, since most patients are not likely to be diagnosed during this highly infectious phase prior to seroconversion [79-81]. In addition, the feasibility and acceptability of regular HIV testing of whole populations, acceptance of immediate ART irrespective of disease stage or symptomatology, and the extent of behavioral risk disinhibition [82] will all be critical determinants of the ultimate success of a UTT intervention. Concerns regarding community acceptability, protection of voluntariness, avoidance of stigma, and preservation of human rights must also be addressed.

Clearly, empirical field studies are needed to test the practical performance of UTT interventions and to measure their impact on HIV transmission. Universal testing is a key component of the UTT strategy and provides the framework for delivering proven preventive interventions to those who are HIV-uninfected at the same time as offering immediate treatment to those who are identified as HIV-infected. Therefore, UTT is fundamentally a combination prevention strategy - this is the approach that will be evaluated in this rigorously-designed, cluster-randomized trial in two severely affected countries in Southern Africa.

Because of the uncertainties regarding the additional impact provided by offering immediate ART (compared to offering ART according to current national guidelines) we propose to carry out a trial with three arms. In this trial, the full combination prevention package including UTT (Arm A) will be compared with a UTT package that includes all components of the intervention except for immediate ART irrespective of CD4 cell count (Arm B); a control arm will allow comparison with a population receiving standard of care (Arm C).

1.2.4 Innovation

The PopART intervention moves the HIV prevention field forward in several important ways.

First, this will be one of the first studies to evaluate the impact of the UTT prevention approach on population-level HIV incidence in sub-Saharan Africa. The concept of UTT for HIV control in Africa is relatively new; the landmark modeling paper of Granich et al appeared only two years ago in 2009 [83]. While the epidemiological rationale for the intervention in HPTN 071 is strong, the approach is controversial. Many question whether it is wise to ask health systems that are struggling to deliver ART even at low treatment thresholds (e.g., at CD4 <200 cells/µL) to provide and supervise a program of immediate ART that goes well beyond the revised WHO guidelines. Nevertheless, there are many arguments in favor of using this approach for HIV prevention:

- Combination prevention incorporating UTT is currently the only strategy that has the potential to eliminate HIV infection in the longer term in the most severely affected countries.
- While initial costs may be high, model estimates suggest that the intervention will be cost-saving in the long run, especially if averted costs of hospital treatment for HIV-related disease are taken into account [26, 84, 85].
- Unless HIV incidence is reduced substantially, ART treatment services will have to meet an ever-increasing case-load and this burden will greatly outweigh the initial costs of implementing UTT.
- Those currently not treated because their CD4 counts do not meet ART eligibility criteria will in any case need to be treated in a short time; meanwhile they are at risk of transmitting the virus to partners, thus increasing the future care burden. Moreover, patients are often lost to follow-up before they have CD4 counts below treatment thresholds.
- There is increasing recognition of the individual clinical benefit of starting treatment earlier. The risks and benefits of immediate treatment have not yet been established for those with CD4 counts > 550 cells/µL. This issue is under investigation in the START trial, but results from that trial will not be available until 2015. However, treatment practice in industrialized countries has already moved a long way in the direction of immediate, universal treatment (see The 2012 US ART guidelines and the 2012 British HIV Association guidelines) and current treatment guidelines in sub-Saharan Africa will rapidly come to be seen as second-class treatment [86-88].
- UTT reduces the complexity of ART delivery, since it avoids the need for CD4 or viral load testing before treatment initiation (which is not always feasible, especially in rural settings). Simplified approaches to testing, treatment and monitoring will partly offset the burden imposed by greater patient numbers.
- Current treatment approaches often lead to severe delays in onset of treatment, so that CD4 counts are often extremely low when ART commences. This leads to greater morbidity, mortality and ongoing transmission, with the associated costs of additional health care for individuals who go on to present with HIV-related illnesses.
- UTT is also projected to have a major impact on the incidence of TB, which often occurs at relatively high CD4 counts, thus reducing morbidity, mortality and the burden on overstretched TB control programs [89, 90].

Second, the UTT intervention will be delivered as part of a combination prevention package that also includes counseling, referral for PMTCT services, and other proven

preventive interventions, including male circumcision. We argue that UTT is by its nature a combination prevention method, in that delivery of universal HIV testing and counseling itself comprises an important prevention package that has been shown to alter reported sexual risk behavior, especially when couples are tested together [91]. In addition, universal testing provides the framework for delivery of prevention services to both HIV-uninfected and HIV-infected individuals. This trial will measure the overall impact of the UTT prevention package on HIV incidence, rather than measuring the impact of any particular component of the intervention. Using a three-arm study design will make it possible to estimate the additional impact provided by offering immediate ART (in addition to the other components of the package). Furthermore, empirical data from the trial on the operational performance of individual intervention components and the measured impact of the two intervention packages (Arms A and B) will be assessed using mathematical models; these models will also be used to explore the projected effects of alternative combinations. The rigorous data generated on the overall effect of the packages, together with these model projections, will be of considerable value to policy makers.

Third, we believe that the proposed intervention package, if successful, will provide a conceptually simple approach that avoids some of the limitations of other combination prevention approaches that emphasize targeting of special interventions to groups at high risk. As we have argued above, while such interventions may still be needed, formulating and delivering locally-appropriate packages on a national scale would be extremely challenging. In contrast, the PopART intervention can potentially be implemented on a wide scale using a relatively uniform and standardized approach, as has been the case for other major public health interventions such as use of impregnated bednets for malaria prevention.

If additional funding can be identified, the following would also be addressed:

Fourth, in addition to measuring the primary endpoint of our cluster-randomized trial design (population-level HIV incidence), we will also measure the impact of the intervention on community viral load if funding for these activities is available. Despite some limitations, community viral load has been proposed as a valuable indicator for assessing the effect of treatment-based HIV interventions, and by making comparisons across our study communities, we hope to investigate how this indicator relates to the HIV incidence measure.

Fifth, the universal test and treat strategy being investigated in the HPTN 071 study is likely to have a significant effect on TB [92, 93]. On an individual level it is well established that TB is increasingly common at lower CD4 counts. However, the risk of developing TB increases rapidly after acquisition of HIV[94]. ART has been shown to reduce the risk of developing TB in individuals by increasing CD4 counts[95]. The effects of ART on TB at community level are not known. If additional funding is available, an additional survey will explore the potential effect of community-wide HIV testing and ART initiation at different CD4 levels on community-level TB burden by testing for TB among a subset of community members at study month 36, as outlined in Appendix IX. Whether or not this component is funded, the study will assess impact of the intervention on TB as determined from health center records. This would be a fifth way by which HPTN 071 would advance the HIV prevention field.

Sixth, the primary strategy to prevent mother to child transmission of HIV is the provision of maternal and neonatal anti-retroviral (ARV) prophylaxis [7]. Scale-up of this intervention has taken place across sub-Saharan Africa. Despite this, there is evidence of on-going transmission to children in the continent, with up to 12% of children whose mothers received some form of PMTCT prophylaxis testing positive for HIV[71, 96, 97]. ART during pregnancy and breastfeeding provides more effective protection against mother-to-child transmission of HIV than standard short course ART regimens[98] which are still being implemented in many African countries. The PopART interventions, through immediate provision of ART, have the potential for a significant impact on HIV-free survival in children through earlier initiation of maternal HIV treatment. They may also lead to an improvement in overall child survival through potential secondary benefits such as improved maternal health, changes in health-seeking behavior, improved care, and increased resources for better nutrition for both HIV infected mothers and their children. If additional funding is available, we will therefore compare the potential effect of the interventions on HIV-free survival amongst HIV-exposed children and overall child survival across the three arms, as described in Appendix X. Whether or not this component is funded, the study will assess the effect of the intervention on uptake of PMTCT services.

Seventh, The development of drug resistant HIV infection will be compared between the three arms (dependent on additional funding) One of the key safety concerns about the population level implementation of a UTT approach is evolution of HIV resistance to ART. This will be examined amongst the HIV+ individuals enrolled into the PC who have detectable viral load measurements whilst on ART, as well as baseline viral genotyping for those who seroconvert through the study period to document the prevalence of transmitted drug resistant infection.

Our preliminary modeling indicates that the intermediate intervention (Arm B) should have a substantial impact on HIV incidence, but that a much larger impact should be seen in Arm A (see Section 2.3). The three-arm study design will allow us to confirm these projections. Detailed data on the costs of these intervention packages, combined with the impact data from the trial, will provide critical policy guidance on the cost-effectiveness of combination prevention strategies and the priority that should be given to earlier treatment. Operational data from the trial will provide valuable information on the practical issues involved in delivering such programs to scale.

2.0 STUDY OBJECTIVES AND DESIGN

2.1 Primary Objective

- The primary objective of this study is to measure the impact of the two intervention packages on HIV incidence by enrolling and following a random sample of adults (the *Population Cohort*) in the trial communities for 3years.

2.2 Secondary Objectives

The secondary objectives of the study are to:

- Measure the impact of the two intervention packages on the following:
  - HIV incidence over the first, second, and third years of follow-up
  - Community viral load (if funding is available)
  - ART adherence and viral suppression (if funding is available)
  - ART drug resistance (if funding is available)
  - HSV-2 incidence
  - HIV disease progression and death
  - ART toxicity
  - Sexual risk behavior
  - Case notification rate of tuberculosis
  - HIV-related stigma
  - Uptake of PMTCT
  - Uptake of male circumcision
  - ART screening and uptake
  - Uptake of HIV testing and retesting
  - Time between HIV diagnosis and initiation of care
  - Retention in care
- Carry out case-control studies to examine factors related to:
  - Uptake of HIV testing during the first round of home-based testing in Arms A and B
  - Uptake of immediate treatment in Arm A
  - Uptake of HIV testing during the second round of home-based testing in Arms A and B
- Use qualitative methods to:
  - Assess popular understanding of HIV testing and treatment at study initiation and during implementation
  - Evaluate the acceptability and functioning of the Community HIV-care Providers (CHiPs) in Arms A & B

  - Evaluate the acceptability of interventions and barriers to access in Arms A & B

  - Document the effect of the interventions on social networks, stigma, sexual behavior, alcohol use, gender-based violence, HIV identity, other HIV prevention options and community morale

  - Evaluate the process and challenges of community consultation and applying ethical principles

- Measure the burden experienced by local health centers due to implementation of the intervention in the community

- Measure the incremental cost of the two intervention packages through systematic recording of costs in intervention and control communities

- Estimate the effectiveness and cost-effectiveness of the intervention packages and alternative packages, both in the chosen study populations and in other populations by fitting mathematical models based on the empirical data from the trial, including data related to cost.

NB – Table 7 provides an overview of the datasets that support the objectives.

2.3 Study Design

The two intervention packages will be implemented and their impact on population-level HIV incidence will be evaluated using a cluster-randomized trial design.

A total of 21 study communities (12 in Zambia and 9 in South Africa) will be selected. The *cluster* or *community* for the purposes of this trial will be defined as the catchment population of a local health unit (through which the intervention will be delivered), and will correspond to a total population of between about 20,000 and 150,000 individuals (average size of approximately 55,000). These 21 communities will be formed into 7 matched triplets, with 4 matched triplets in Zambia and 3 in South Africa. In each matched triplet, one community will be randomly selected to receive the full intervention (Arm A), a second community will receive the full intervention except that ART will be offered according to current national guidelines (Arm B) and the third will act as a control community and will receive standard of care. Within each country, communities will be matched based on the best available estimates of HIV prevalence, as described in Section 4.1, with the aim of minimizing the between-community variance in baseline HIV incidence within matched triplets. In addition, restricted randomization will be used to ensure overall balance in cluster size, ART uptake and mean HIV prevalence across the study arms. The community randomization scheme is represented graphically in Figure 1.

The primary outcome of the study, HIV population-level incidence, will be measured through longitudinal follow-up of a cohort of 2,500 adults consenting to participation, drawn from a randomly selected list of households in each community (the *Population Cohort*).

If funded, at the end of the third year of the intervention, to coincide with the 36 month follow-up visit of the *Population Cohort*, a random sample of houses will be selected (excluding houses in the *Population Cohort*) and visited by field staff to complete a final survey. Because this *Population Cross-Sectional Survey* will be a one-time survey, data obtained from these individuals will be uncontaminated by the potentially biasing effects of longitudinal cohort participation, and will provide additional data on community viral loads and other process measures.

Figure 1- Community Randomization Scheme, Zambia and South Africa

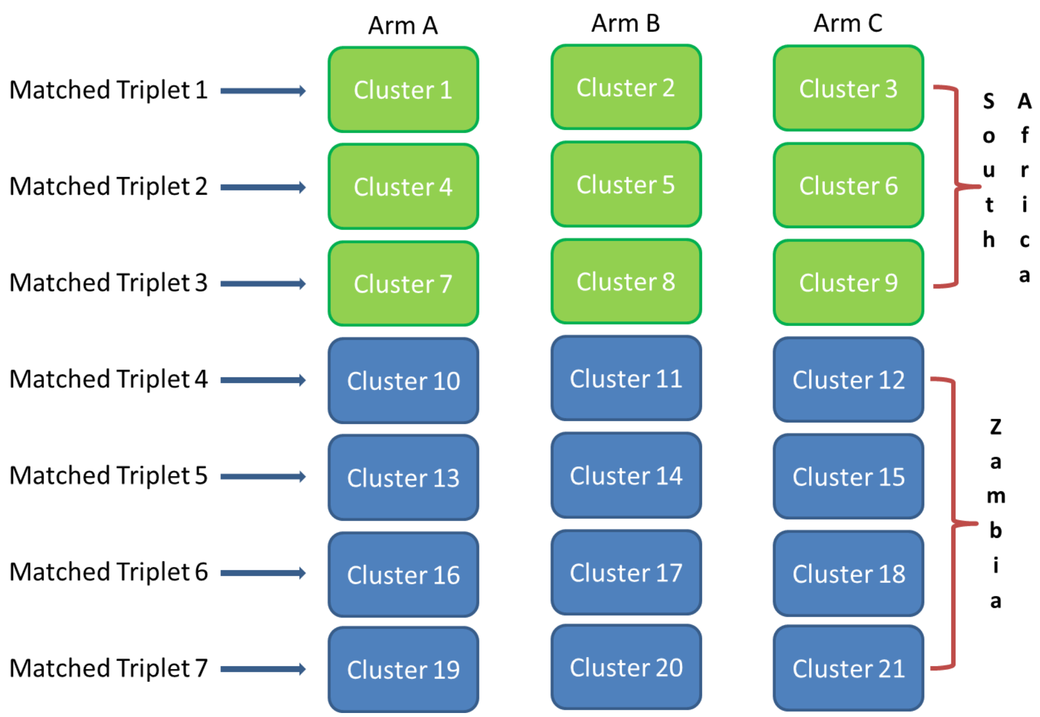

2.4 Timing of Deployment of Intervention and Research Components

The total duration of the study will be approximately 6 years. During the first year, the protocol will be finalized, study procedures defined and manuals of operations developed, plans and procedures developed with community and implementing partners, and study staff trained. Preliminary qualitative work will also be conducted in the communities to prepare for study initiation. Shortly prior to deployment of the intervention, households in each community will be mapped. Early in the second year, the intervention, implemented by the *CHiP teams* (home-based testing and linkage to medical care in the health centers) will be deployed in Arms A and B at the same time that the *Population Cohort* is enrolled by the *research teams* for evaluation in all arms. It is expected that the first round of deployment of the intervention will take approximately six months, as will enrollment of the *Population Cohort*.

CHiP teams will stay engaged in the community throughout the intervention period, but will return to all households to repeat rounds of home-based testing 12, 24, and 36 months after the initial round of testing. Similarly, research teams will conduct evaluation visits to the homes of the *Population Cohort* members at enrollment, 12, 24 and 36 months. The timing of the *Population Cohort* and intervention will be organized as far as possible so that individuals are surveyed for the *Population Cohort* before the corresponding CHiPs household visit. However, for feasibility reasons, this will not always be the case. Whether or not an individual has been seen by a CHiP team will be asked during the *Population Cohort* survey to help interpret the data, particularly with regards to uptake.

During the first two years of intervention, an interim evaluation will take place to determine whether to continue with the 36 month follow-up of the *Population Cohort* and the fourth year of intervention (see Figure 2). The evaluation will consider milestones such as uptake of the intervention and indicators of futility and will be described in the Statistical Analysis Plan.

If funded, the *Population Cross-Sectional Survey* and the *TB* and *PMTCT* surveys, will occur at the same time as the 36-month *Population Cohort* visits. Case-Control studies and qualitative research by the research teams will occur at intervals during the entire follow-up period. Analysis and reporting are expected to take two years after completion of the 36-month follow-up of the *Population Cohort*. This timeline is represented graphically in Figure 2. Analysis and reporting will be on-going following completion of the final survey.

Figure 2- Timing of Deployment of Intervention and Research Components

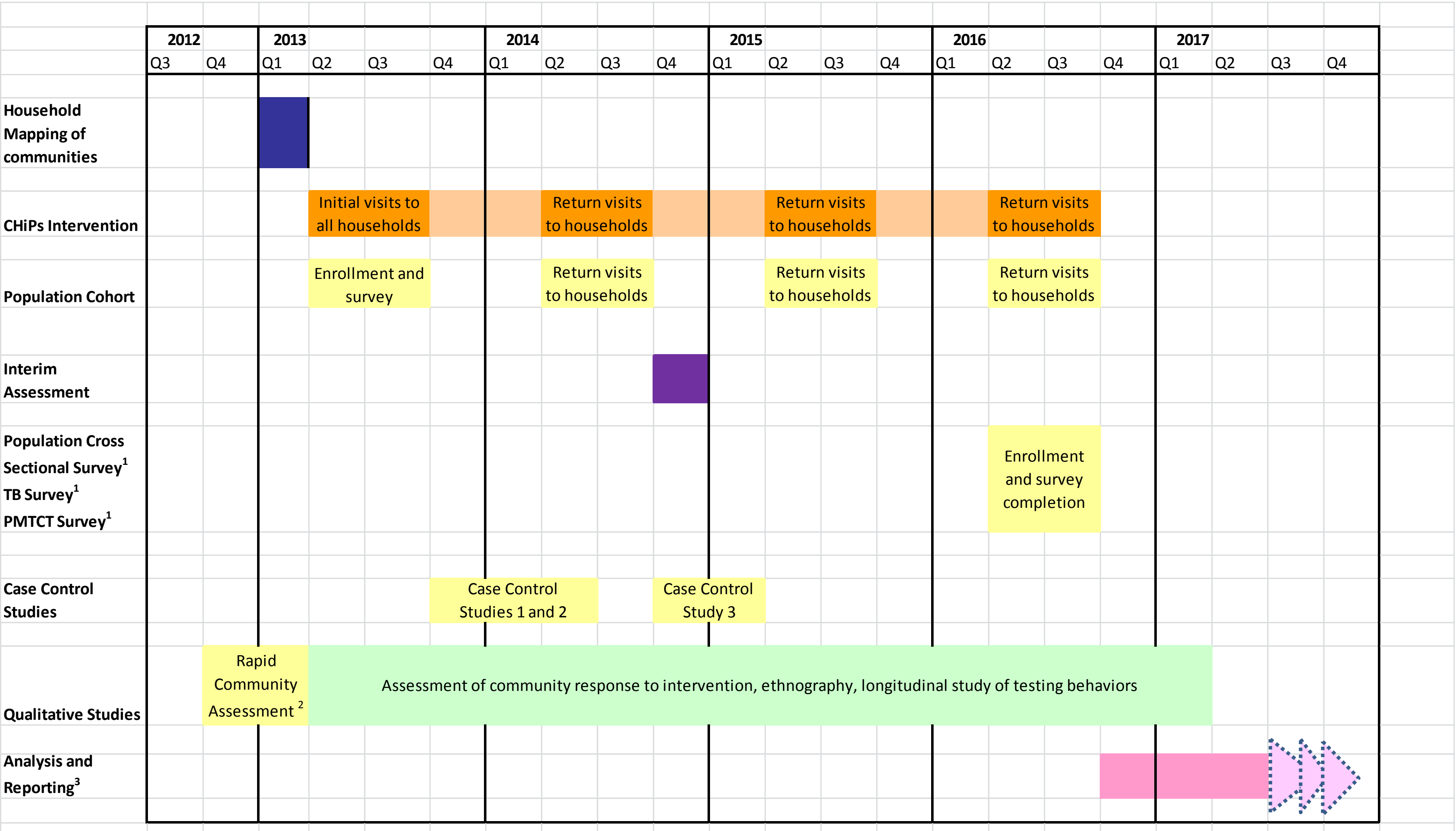

[1] Requires additonal Funding

[2] To be described in and conducted according to an ancillary protocol

[3] Full completion of the analysis is likely to extend beyond Q2 2017

2.5 Cross-Sectional HIV Incidence Estimation

In HPTN 071, HIV incidence estimates will be based on longitudinal assessment of HIV seroconversion. A robust, multi-assay approach for cross-sectional HIV incidence determination was recently validated for subtype B HIV. This algorithm uses a combination of two serologic assays (the BED capture immunoassay [BED-CEIA] and an avidity assay), as well as two non-serologic biomarkers (CD4 cell count and HIV viral load) to identify individuals who are likely to be recently HIV-infected at the time of sample collection. Work is underway to optimize a similar multi-assay algorithm in subtype C HIV.

HPTN 071 provides an opportunity to apply these methods to achieve two objectives: estimation of HIV incidence at baseline (prior to implementation of the study interventions), and comparison of HIV incidence estimates based on longitudinal and cross-sectional assessments. The serologic assays (BED-CEIA and the avidity assay) and HIV viral load can be performed retrospectively using stored plasma samples. In contrast, CD4 cell count must be obtained in real-time (within 7 days of sample collection) from all individuals who have evidence of HIV infection. Because of concerns related to the overall cost of the study, the current version of the trial protocol does not include CD4 cell count testing for the *Population Cohort*. Therefore, cross-sectional HIV incidence testing using the current multi-assay algorithm described above is not possible. The protocol team acknowledges the importance to the HIV prevention research field of developing and validating such assays, and is still considering possible inclusion of cross-sectional HIV incidence measures in the study. If other methods for cross-sectional HIV incidence estimation are developed in the future that are robust and do not require CD4 cell count data, it may be possible to apply those methods to HPTN 071 using stored plasma samples.

3.0 STUDY INTERVENTION

3.1 Implementation Team Experience

The implementing team that will carry out this program has extensive prior experience in conducting community randomized research, including household-level incidence assessment, particularly in the conduct of the Zambia-South Africa TB and AIDS Reduction (ZAMSTAR) trial in the same communities that have been chosen for this study. The leadership and much of the field team from the ZAMSTAR trial remain actively engaged in the communities and will be able to build on their knowledge of and acceptance within these populations when rolling out the community interventions described below.

In addition, extensive effort has been put into developing in-country coordination structures for the trial. In both countries, there have been and will continue to be ongoing dialogues with national, provincial and district Departments of Health, PEPFAR secretariats, USAID and CDC HIV treatment and prevention representatives, other

implementing partners, and community representation organizations. The study team has also developed sustainability plans and community engagement plans.

3.2 Description of CHiP Teams

As described above, seven communities will be randomized to receive the full intervention and seven will be randomized to receive the intervention except with eligibility for ART determined by current national guidelines. In these two types of intervention communities (Arms A and B), delivery of the intervention will be carried out primarily by trained community health workers or 'CHiPs' (Community HIV-care Providers). The CHiPs will provide HIV counseling and testing and active linkage to comprehensive care and prevention services. Each CHiP team will consist of a gender-balanced group of individuals trained in HIV counseling and testing, and other aspects of HIV prevention and care. Each CHiP team will be responsible for implementing the intervention in an assigned subset of households, or "zone". Although CHiP teams are affiliated with this research project (and this will be made clear to all those who interact with them), their role is primarily to deliver what is recognized by the WHO as a 'best practice' public health intervention. Hence for this project we regard 'CHiP teams' as separate from the 'research teams' and believe that the norms and standards governing their activities should largely be those accepted for the implementation of public health interventions rather than those applied to conventional clinical research projects. Regarding all CHiP team activities as research activities would make this complex public health research project logistically impossible to implement.

A cadre of people currently exists in all the study communities who would be appropriate for recruitment as CHiPs. These include ART adherence supporters, TB Treatment Supporters, PMTCT and male circumcision peer educators, home based care volunteers and HIV/AIDS support group members. Most of these people have the necessary skills and have received training in basic HIV counseling, psychosocial counseling, adherence counseling and door-to-door HIV testing. However, successful candidates will be retrained to update their knowledge and harmonize the implementation of the study interventions. Community Advisory Boards (CABs) and other local stakeholders will be consulted in developing job descriptions for CHiPs. All CHiPs will be able to read and write English, and will be conversant with the local geography.

3.3 Universal HIV Testing and Linkage to Care

Door-to-door voluntary HIV testing will be offered to all community members 16 years of age and older in Zambia and 12 years of age or older in South Africa, and any minors younger than these ages who request a test, with the consent of their guardians. The CHiP team will map, visit, and enumerate all households in their zone. Testing will occur during the first 6 months, and will be repeated at annual intervals for those who are HIV-uninfected or who are not tested for any reason during the first round. Household visits will be made at times convenient for community members, with repeat visits arranged for adult household members not present during the first visit. HIV testing will be done on finger-stick samples using two rapid kits in series. Individuals with discordant or inconclusive test results will be further evaluated according to national guidelines.

Following the household visit, the CHiP team will be responsible for ensuring linkage to HIV care at the health center for individuals identified as HIV-infected (defined as attending the health center and being given an “HIV care” patient number), offering male circumcision to men who are HIV-uninfected, facilitating linkage to the male circumcision service, and providing a regular supply of condoms to all households. They will subsequently make periodic visits to all households according to a regular schedule, to check on uptake of services (including male circumcision and ART), encourage HIV testing for those who have not been tested recently, and to provide adherence support to those receiving ART (see below). Essential data on each household member will be captured electronically and this will be linked to the community HIV database at the health center (see below), using a unique identifier.

In addition to the door-to-door service provided by the CHiP team, provision of HIV testing at other venues will be strengthened. This will include opt-out, provider-initiated testing and counseling for all patients presenting to the health center for any reason, testing of all women attending antenatal clinics (ANC), voluntary counseling and testing services provided at the health center or other community venues, and (if appropriate for the community) services provided in occupational settings. Data from venues will be entered into the same community HIV database, to ensure that the CHiP teams are informed on the testing history and service delivery to each community member in their zone and to assist with linkage to care. Information on how confidentiality of data captured into electronic databases will be maintained is provided below in Section 8.8.1.

3.4 Male Circumcision

Services for safe medical male circumcision will be available in all study communities. In most cases, the service will be provided within the health center, but if this exceeds the capacity of the health center, a special service will be set up during the initial phase of the intervention at a convenient community location. Various bloodless devices for male circumcision are currently being evaluated. If these are confirmed to be safe, effective and acceptable, and are approved by the United States (US) Food and Drug Administration (FDA), the study team will explore whether these devices can be used to simplify the delivery and improve the acceptability of this service.

3.5 Universal Treatment (Arm A)

Immediate eligibility for ART, irrespective of CD4 count, will be offered to all individuals attending adult HIV treatment and care services in the health centers. This will include those diagnosed during the door-to-door testing campaign as well as those diagnosed through other testing venues as described above. It will also include HIV-infected patients diagnosed previously who have not yet initiated ART, either because they have not been followed up at the health center or because they are not yet eligible for ART under current national guidelines. In South Africa and in Zambia, there is no requirement to prove residency in order to receive treatment at any clinic. The clinics will however collect locator information so that the study team is aware of how many patients are coming from outside of the catchment area.

Linkage from diagnosis to treatment will be a critical component of the intervention. The CHiP team for each zone will be responsible for ensuring that this linkage takes place.

They will enter details of adults identified as HIV-infected in the electronic database and will provide referral to the health center for initial assessment. They will also offer to accompany patients to the health center.

On presentation at the health center, patients will have baseline blood tests performed in accordance with current standards of care in Zambia and South Africa. CD4 testing will also be performed, but results of CD4 testing will not be used to guide the initiation of ART in this study arm: all patients without contraindications will be immediately eligible for ART regardless of CD4 count. After exclusion of active TB, patients will be offered TB preventive therapy according to national guidelines. Patients will also be offered antibiotic therapy for prophylaxis against opportunistic infections. The project team will endeavor to ensure that drug supplies are maintained without interruption.

Ensuring a high level of adherence to ART is key to the success of the intervention. The CHiP team will be responsible for making regular home visits to patients to provide psycho-social support and to check and support treatment adherence. Because the CHiP team will make regular visits to all households in their zone (see above), this will reduce stigmatization or identification of HIV-infected individuals. Activities of the CHiP team will be supported by automatic updates produced by the electronic database showing which patients have missed clinic appointments or are due for a home visit.

3.5.1 Choice of ART Regimen (Arm A)

To simplify the implementation of the UTT approach, a simple, standard regimen has been chosen that should be safe and effective for most patients. All consenting individuals without contraindications) will be given tenofovir/emtricitabine and efavirenz or an appropriate alternative regimen (see the Study Specific Procedures SSP Manual (SSP) for details) in line with standard national treatment recommendations. This is currently the first line treatment regimen in both Zambia and South Africa, and so the same treatment regimen will be used for patients initiating ART in all three study arms. The small number of individuals for whom this regimen is contraindicated according to national guidelines will be treated using alternative regimens as recommended by national guidelines for ART patients. ART adherence and toxicity monitoring will be managed according to national recommendations. Switch to a second-line treatment regimen and choice of second-line regimens will also be according to standard guidelines. Reported levels of transmitted drug-resistant virus remain low in these settings (< 6%). Most cases of transmitted drug resistance are to nucleoside reverse transcriptase inhibitors or non-nucleoside reverse transcriptase inhibitors, with minimal transmitted drug resistance to protease inhibitors [99].

3.6 Treatment According to National Guidelines (Arms B & C)

Arrangements for linkage to care, treatment and monitoring in Arm B will be similar to those in Arm A, except that ART will be initiated only if the patient is eligible according to current national guidelines (e.g., based on CD4 count or HIV clinical stage). Treatment regimens will follow current national standards of care in all arms. Additional arrangements for linkage to care will not be offered in Arm C.

3.7 Prevention of Mother-to-Child Transmission

In all three study arms, the project team will endeavor to ensure that national policy for PMTCT is delivered effectively at health centers providing antenatal or delivery care, which will usually be the same centers at which ART is delivered.

In Arms A and B, the CHiP team will encourage women who may be pregnant to receive pregnancy testing at the health center. As well CHiPs will encourage pregnant women who are encountered during regular household visits in their zone to attend an ANC. In Arm A, all HIV-infected women should be offered immediate initiation of lifelong ART, and the CHiP team will be responsible for checking this and for assisting with linkage to care, if this has not taken place. For women in Arm B who according to national standard guidelines are not eligible for ART and women in Arm A who decline immediate treatment, the CHiP team will ensure that they are offered standard PMTCT services according to national policy. National policies in Zambia and South Africa may change during the period of this study to initiate lifelong ART among pregnant women with HIV infection. If this occurs, then all pregnant women in both Arms A and B will be linked to care for immediate ART in the country or countries where it is the national policy.

3.8 Management of Sexually Transmitted Infections

Services for STI treatment will be in place in all health centers according to national policies. In all three study arms, the project team will endeavor to ensure that these services are operating effectively, and that drug supplies for STI treatment are maintained without interruption.

3.9 Provision of PrEP

There is growing evidence from randomized clinical trials that use of oral daily PrEP confers significant protection against HIV acquisition, but it is currently unclear how the results of those trials will be incorporated into WHO and national guidelines. New developments will be reviewed during the course of the trial. If the provision of PrEP is incorporated in national guidelines during the course of the trial, the combination prevention package will be adapted appropriately.

3.10 Standard of Care

The primary objective of this trial is to evaluate the impact of an intensive combination prevention intervention program on HIV incidence when compared with current standard of care in Zambia and South Africa. The study team will work with in-country health authorities to ensure to the degree possible that existing services in the seven control communities meet current national guidelines for HIV prevention and care. These activities include endeavoring to ensure that:

- Community members have adequate access to services for voluntary HIV counseling and testing.
- Referral services for male circumcision are available to men who are HIV-uninfected and wish to be circumcised.

- HIV treatment and care are provided according to current national guidelines. The study team will endeavor to ensure that antiretroviral drugs are available to all patients who qualify for treatment, using the current ART drug regimen employed in the government program in each country.
- Adequate services for PMTCT are in place at antenatal and delivery services in the control communities.
- Treatment services for STIs and condoms are available through health units in accordance with national HIV prevention guidelines.

To help interpret the results of the trial, process data from the control communities on HIV testing uptake, ART delivery, male circumcision, and provision of PMTCT services will be collected for comparison with the intervention communities. These data will be used to inform model fitting and projections.

3.11 Delivery of Intervention

3.11.1 Activities with Local Health Centers/Community Institutions

The study team will collaborate with local health centers/community institutions to facilitate the following:

- Establishment of a community-wide HIV testing database in which the name and HIV test results of anyone identified as HIV-infected at any HIV testing location will be entered to enable follow-up (Arms A & B only)
- Promotion of the study at the community level (Arms A & B only)
- Strengthening the provision of HIV services at local health centers and elsewhere in all arms, including
  - ANC care
  - Voluntary Counseling and Testing (VCT) at health centers and other venues
  - PMTCT services
  - STI treatment and referral services
  - Male circumcision services and referral
  - Activities with national/global health entities
  - Occupational venue testing, if appropriate (strengthened in Arms A & B; in Arm C the study will support if already part of local services)
  - Opt-out provider-initiated counseling and testing (strengthened in Arms A & B; in Arm C the study will support if already part of local services)

The home-based HIV testing that is carried out by the CHiP teams will be captured on the CHiPs electronic data collection device and uploaded to the community-wide HIV testing database. This community-wide HIV testing database will be linked to the HIV care database, to estimate the proportion of HIV-infected individuals who register for HIV care following an HIV diagnosis, and the time from HIV diagnosis to HIV care registration. In Arms A and B, but not Arm C, there will be active follow-up of HIV-infected individuals who have been referred for HIV care by CHiP teams, but who have not registered for HIV care. CHiP teams will also provide additional support for retention in HIV care and ART adherence, contributing to active follow-up of individuals who have missed scheduled

visits. If an individual has left the community, they will not be followed up outside the community.

3.11.2 Collaborations

The study team will collaborate with national and global health entities to facilitate the following in all communities:

- Adequate supplies of antiretroviral drugs for all who are prescribed them
- Adequate STI care, including test kits and drugs for STI and treatment
- Adequate supplies of condoms
- Adequate clinic staffing
- Approval of use of health center data
- Adequate clinical supplies for HIV-infected individuals, including
    - TB tests and treatment
    - Blood tests for clinical care
    - Antibiotics for TB and opportunistic infection prophylaxis

Table 1- Summary of Intervention Components

Study Arms	Activity
Arms A & B	Study Start
	• Enumeration of all houses in each community<br>• Division of houses into "zones" and assignment of a CHiP team to each zone<br>• CHiP Team will:<br>o Offer HIV testing with counseling to all household members (all individuals 16+ years old in Zambia and 12+ years old in South Africa and minors with the consent of their guardians) and will record HIV status with name in mobile device<br>o Provide linkage-to-care at local health center for HIV-infected persons<br>o Refer/link men who are uncircumcised to circumcision, if interested, focusing on men who are HIV-uninfected<br>o Identify pregnant women and encourage them to get follow-up at an ANC; encourage HIV-infected pregnant women to initiate ART (Arm A) or PMTCT per national guidelines (Arm B) as part of their care<br>o Provide on-going psycho-social support for ART adherence to those on ART<br>o Encourage STI treatment and provide prevention resources including condoms
	On-going Throughout the Study
	• CHiP team will:<br>o Promote community-based HIV prevention services in their zone<br>o Follow up with all persons in their zone who are identified as HIV-infected (by CHiP team or at other venues) to encourage and facilitate them to access HIV care

	○ Return to houses where residents were not available for testing during original or subsequent visits, to complete testing of all willing residents<br>○ Encourage pregnant women to get follow-up at an ANC; encourage HIV-infected pregnant women to initiate ART (Arm A) or PMTCT per national guidelines (Arm B) as part of their care<br>○ Provide on-going psycho-social support for ART adherence to those on ART<br>○ Encourage STI treatment and provide prevention resources including condoms
	Follow Up Testing at 12-, 24-, and 36-Months
	• CHiP teams will cycle back through their zone at 12, 24, and 36 months to repeat universal testing in each household for those not previously diagnosed as HIV-infected
	Procedures and Tests at the Health Centers
	• Community members who are identified as HIV-infected will receive clinical support and laboratory tests at local health centers, consistent with national guidelines for HIV treatment and care, with immediate eligibility for ART initiation (Arm A) or eligibility for ART according to national guidelines (Arm B)
	By Study Start and Throughout the Study Period
	• Endeavour to ensure that the following resources are available:<br>○ Voluntary HIV counseling and testing<br>○ Male circumcision<br>○ PMTCT<br>○ HIV treatment and care<br>○ STI treatment and prevention resources including condom distribution<br>○ Clinical support and laboratory tests at local health centers for provision of prophylaxis against TB and other opportunistic infections for all HIV infected individuals, consistent with national guidelines for HIV treatment and care
Arm C	By Study Start and Throughout the Study Period
	• Endeavour to ensure that the following standard-of-care resources are available:<br>○ Voluntary HIV counseling and testing<br>○ Male circumcision<br>○ PMTCT<br>○ HIV treatment and care according to national guidelines<br>○ STI treatment and prevention resources including condom distribution<br>○ Clinical support and laboratory tests at local health centers for provision of prophylaxis against TB and other opportunistic infections for all HIV infected individuals, consistent with national guidelines for HIV treatment and care

3.12 Monitoring and Evaluation Plan

The delivery of the intervention will be monitored at frequent intervals from the time of initiation to evaluate the uptake of the intervention. Remedial action will be taken at cluster-level if delivery is behind schedule. Details of these procedures will be set out in the SSP manual.

Briefly, during each round of CHiPs testing, HIV testing uptake is targeted at 90%. In each community, individual CHiP teams will report weekly to the CHiPs supervisors using data from electronic records. Where a team is not meeting the target level of testing uptake, this will be explored in real time and where necessary appropriate intervention, retraining or modification of strategies will take place. Following HIV diagnosis, the target will be for linkage to care and (in Arm A communities) initiation of ART in 80% of cases within 3 months. These targets of 90% uptake and 80% initiation should lead to an overall uptake of 72%, just above our central target of 70% uptake (see Table 4). This will be supported by notification of CHiP teams, based on clinic and CHiPs databases. When patients have not presented within a defined interval, this will trigger repeat home visits for follow-up and support to access care. Data on linkage to care will be reviewed monthly to identify CHiP teams that are not meeting targets and to effect remedial actions as noted above.

As stated in Section 2.4, interim evaluation will take place during the first two years of intervention to determine whether to continue with the 36 month follow-up of the *Population Cohort* and the fourth year of intervention. The evaluation will consider milestones such as uptake of the intervention and indicators of futility.

The study team will also have a continuous presence in each community and will monitor other programs in the community that may affect uptake of the intervention.

4.0 STUDY POPULATION

4.1 Description/Selection of the 21 Study Communities

This study will be carried out in areas of Zambia and South Africa that are known to have high HIV prevalence and incidence and are continuing to experience severe generalized HIV epidemics, with prevalence levels of 15-20% in many areas. National estimates of HIV prevalence in adults aged 15-49 are 13.5% for Zambia and 17.8% for South Africa [34, 35], and incidence estimates are 1.17% and 1.49% respectively.[100]

The specific communities selected for randomization in this trial are largely the communities that were selected for the ZAMSTAR trial. Selection criteria for communities included having a health facility that offered TB and HIV services, a high HIV prevalence, a TB notification rate of at least 400/100,000 per year and a total population of about 20,000 or more. The communities were selected in conjunction with national and local health authorities. All communities were willing to be included in a randomized trial. Extensive work has been done with community representatives to ensure that they understand the fundamentals of research and they were all very supportive during the ZAMSTAR trial.

Additional considerations that informed selection of these sites for the current study included:

- Geographically distinct
- No other major HIV prevention studies planned or ongoing
- Adequate population size to minimize the effects of contamination on outcome measurements (due to contact with other communities or residents of other communities)
- Community willingness to be involved in this current study

The final endpoint measurement of the ZAMSTAR trial involved a community-level survey of 4000 randomly selected individuals from each community and allowed measurement of the uptake of HIV testing, uptake of ART, circumcision and HIV prevalence, which are presented in Table 2. These data would not otherwise be available at this level, as most surveys only provide data at provincial or district level.

Due to differences between the designs of ZAMSTAR and the current study (requiring seven matched triplets) four ZAMSTAR communities from Zambia were excluded from the current study (the most rural communities with the lowest HIV prevalence) and an additional community was added in South Africa. Maps of the locations of the study communities are provided in Figure 3. It should be noted that study sites have not been completely finalized and some substitutions may be necessary due to study requirements or as an outcome of consultations with local authorities. Final site selection is subject to confirmation after completion of the protocol review process.

Table 2- Twenty one proposed study clusters in Zambia and South Africa and relevant background data

Community	Population	Adult HIV prevalence	Know HIV status	HIV-infected on ART	Men circumcised
**ZAMBIA**					
**Dambwa**	31629	26%	65%	24%	14%
**Maramba**	55011	19%	66%	30%	21%
**Chawama**	129221	15%	35%	16%	8%
**Kanyama**	124284	17%	65%	28%	19%
**Shampande**	41615	16%	58%	38%	14%
**Chipata**	166251	15%	59%	24%	8%
**Ngungu**	38081	17%	30%	18%	7%
**Makululu**	34623	20%	52%	30%	8%
**Ndeke**	33297	13%	56%	31%	17%
**Chimwemwe**	42898	15%	51%	25%	16%
**Chifubu**	60222	19%	56%	32%	17%
**Chipulukusu**	45234	18%	52%	19%	12%
**S AFRICA**					
**Delft South**	31423	14%	48%	23%	53%
**Ikhwezi***	N/A	18%	N/A	N/A	N/A
**Bloekombos***	N/A	22%	N/A	N/A	N/A
**Dalevale***	N/A	13%	N/A	N/A	N/A
**Wellington***	N/A	13%	N/A	N/A	N/A
**Cloetesville***	N/A	16%	N/A	N/A	N/A
**Luvuyo***	N/A	19%	N/A	N/A	N/A
**Kuyasa**	39168	19%	53%	22%	87%
**Town II***	N/A	19%	N/A	N/A	N/A

*Seven South African sites – data not available from ZAMSTAR trial; accurate population size estimates will be available upon ethics approval,% HIV-infected on ART not yet available. Estimates of HIV prevalence based on sub-district antenatal clinic HIV prevalence, or (for Luvoyo and Town II) based on ZAMSTAR data for communities in the same sub-district. Estimates of % who know their HIV status, and % men circumcised, not available for 7 communities that were not included in ZAMSTAR trial.

Figure 3- Location of 21 clusters in Zambia and South Africa

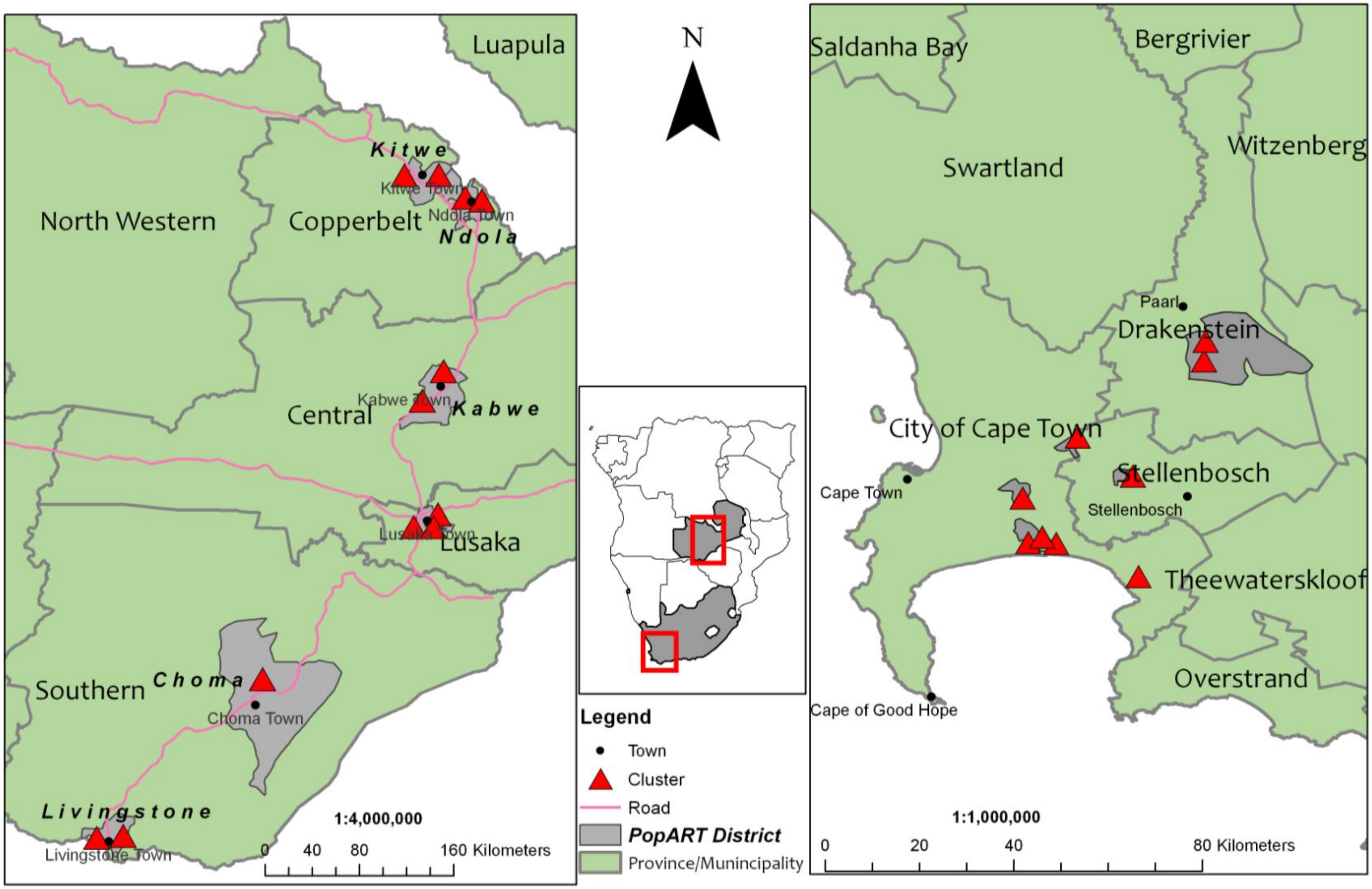

4.2 Randomization

The first step in randomization will be to obtain agreement from communities to take part in the study and to accept the results of the random assignment to a study arm, whatever the outcome. Randomization will then take place in a public ceremony at which the allocation of communities to study arms will be decided using a transparent and fair process. After initiation of the intervention, if any community needs to be removed from the study (for example, if a community should cease to agree to participate in the study) then the study leadership will decide upon the most appropriate course of action, which would likely include replacement of the community.

4.3 Community Engagement

This study will build on the community engagement and community capacity established during the ZAMSTAR trial. To work within a community successfully requires a trusting relationship which requires time to be built, and through the ZAMSTAR trial the research team spent seven years engaging with these communities. Community advisory bodies in all communities were worked with (and developed, where needed), and were trained in research ethics and conduct. These bodies were invaluable during the ZAMSTAR trial to represent community views and to assist the research teams during their work in the communities. The HPTN 071 study will build on these experiences, widening the constituency of these bodies where necessary.

Direct community engagement for this study began early, during the formulation of the research questions, when various community groups (including CABs in former ZAMSTAR study communities), civil society organizations (such as the Cape Metro Health Forum, Treatment Action Campaign and South African National AIDS Council (SANAC)) and government authorities were consulted for their input before the final proposal was submitted, and again after the grant was awarded. Some members of these organizations have provided comments on the protocol and will provide additional input during the preparatory phase of the trial.

A key aspect of the preliminary work during the first year of the study will be a stakeholder analysis, results of which will be used to identify relevant stakeholders to be considered in community engagement as well as membership for CABs. CABs in this study will have broad representation from various community groups and stakeholders such as churches, schools, law enforcement, government structures at community level, health-related committees, and development-related committees. Selection criteria will be arrived at through consultation with the stakeholders. Each study community will have a member of the study team responsible for community engagement activities. One of the main tasks of the staff will be to keep dialogue open and ongoing between researchers and community groups.

Community engagement will be an ongoing process through regular contacts with community groups and CABs. A combination of mechanisms will be utilized, such as community meetings, workshops with key stakeholders, participant meetings, CHIPs meetings and some existing avenues such as health committees, development committees,

civil society groups and local HIV/AIDS coordinating forums such as the District AIDS Task Forces in Zambia and Treatment Action Campaign and SANAC in South Africa. This will enable the study management team to ensure that information about the study is disseminated widely in the communities involved and to keep the community stakeholders updated regarding progress of the study, events that may arise in conduct of the research, and new developments in HIV prevention and treatment. Community engagement will also allow researchers to receive feedback from the community on social harms, individual and community level risks, perceptions about the study in the community, and implementation challenges. All study staff and stakeholders will receive training in Good Clinical Practice (GCP)/research ethics before commencement of intervention implementation.

Community engagement will also be factored into other study processes such as the communication plan, especially the dissemination of study results (preparation of the community). Overall, strong community engagement will allow the establishment of a partnership between communities, participants and researchers to ensure the latter discharge their responsibilities ethically in the study communities**.** A component of the qualitative research will focus on the application of ethical principles in practice as well as documenting and evaluating community engagement.

In both countries, study committees will be formed on which community representatives will serve along with department of health and other stakeholder representatives. These committees will meet periodically for the duration of the trial and these meetings will provide a forum for trial staff to engage with community representatives around the progress of the trial and any relevant issues that may arise.

5.0 RESEARCH PROCEDURES AND ACTIVITIES

The deployment of the interventions among the communities assigned to Arms A and B is expected to lower HIV incidence throughout the communities. Measurement of HIV incidence, however, will occur in a subset of adults enrolled into the *Population Cohort* in each study community and followed longitudinally. Secondary outcomes (among them process measures and qualitative research aims) will also be measured from data provided by this cohort, from routinely-collected health center data, and from data collected by CHiPs during household visits. Other secondary outcomes will be measured from qualitative and case-control studies, and, if funded, from additional one-time surveys. Research activities, including identification and consent of participants, conduct of study procedures, and retention-related activities, will be performed by a trained research team, separate from the CHiP teams that will be responsible for delivering the intervention to the community-at-large. A table summarizing the secondary objectives and outcomes, including the source of outcome data, is provided in Section 7.11.

Descriptions of the *Population Cohort* and surveys are provided below. Detailed instructions to guide and standardize all study procedures across sites will be provided in the SSP Manual.

5.1 *Population Cohort*

5.1.1 Sampling/Recruitment of *Population Cohort*

Prior to study commencement in each community, satellite maps will be used to enumerate and list all the houses in the community. A simple random sample of houses will be selected and visited by field staff who will list all adult residents aged 18-44 years. This age range was chosen because individuals 18 years and older will be able to participate in the cohort without a guardian's consent, and adults under 45 years are believed to be most likely to experience a measurable change in HIV incidence as a result of the intervention. A computer program will be used to select one age-eligible resident from each chosen household at random. The selected individuals will be invited to join the *Population Cohort*, if they meet the other eligibility criteria. A blood sample will be collected and stored for retrospective testing which will include HIV testing and other secondary outcome measures (see Appendix IA). HIV counseling and testing using rapid HIV test kits will be offered to all those who wish to know their test status (participants may refuse an HIV rapid test and still participate in the *Population Cohort*). All HIV-infected individuals (those testing positive on the rapid test as well as those who are already aware of their positive status) will be referred to a health center for further management. All cohort members, irrespective of HIV status, will be followed after 1 and 2 years (interim surveys) and 3 years (final survey) to measure HIV incidence and other outcomes, as described below.

Only one adult will be randomly selected from each household to participate in the *Population Cohort* for outcome evaluation. This is to avoid the distortion of the trial results which might occur if whole households or several members of a household were to be evaluated, since this would in itself constitute a mass testing and counseling intervention. If the person selected for the cohort from a given household is ineligible or refuses participation, the team will move on to the next household on the list. As described in Section 7, the statistical analysis will take into account the different sampling probabilities resulting from the selection of one individual irrespective of household size.

5.1.2 Inclusion Criteria *Population Cohort*

- 18 – 44 years of age
- Able and willing to provide informed consent
- Residing within catchment area of a designated local health unit and intending to remain so for the next three years
- Residing in a randomly selected household

5.1.3 Exclusion Criteria *Population Cohort*

- Current or planned enrollment in another HIV treatment, prevention, or PrEP study
- Current, planned or prior enrollment in an HIV vaccine study
- Anything that, in the opinion of the investigator, would preclude informed consent, make study participation unsafe, complicate interpretation of study outcome data, or otherwise interfere with achieving the study objectives.

5.1.4 Procedures and Activities

***Population Cohort* Creation**

- Generation of random sample of houses in the community for visits
- Research staff visit selected houses and enumerate all adult residents (18-44)
- Selection of one adult at random from the household for invitation to *Population Cohort*
- Complete informed consent

Visit Procedures (Enrollment, 12 months, 24 months, and 36 months)

Administrative, Behavioral, and Regulatory Procedures

- Obtain informed consent for enrollment (study start only)
- Solicit consent to store specimens for future testing and to use participant-identified data from heath center for cohort analyses (study start only)
- Obtain/update locator information
- Complete survey to include socio-demographic, health, social, behavioral, and economic factors
- Perform qualitative interviews covering stigma and discrimination (randomly-selected HIV-infected participants only)

Clinical/Counseling Procedures

- Perform HIV rapid tests, if participant agrees
- Provide pre- and post-test counseling and test results, for those willing to have HIV rapid testing
- Collect blood for laboratory testing and sample storage

Laboratory Procedures

- Plasma storage*

*Stored samples will be used for retrospective, centralized testing. This will include HIV and herpes simplex virus, type-2 (HSV-2) testing (to assess prevalence and incidence), quality assurance (QA) testing for HIV and HSV-2, antiretroviral drug resistance (if funding is identified), and HIV viral load testing (to assess community viral load, ART adherence and viral suppression in a subset of participants, if funding is identified). Other testing that may be performed includes cross-sectional HIV incidence testing and analysis of the linkage of HIV infections. Stored samples may also be used to characterize the HIV virus and the host response to HIV infection. The location of testing for each type of testing will be determined by the HPTN NL. Additional details are provided in Appendix 1A and the SSP Manual.

5.1.5 Reviewing Health Center Records for *Population Cohort*

For HIV-infected *Population Cohort* members who provide consent, the study team will review data from medical records at the health center to measure HIV disease progression and death, ART toxicity, and the time between HIV diagnosis and initiation of HIV care.

5.1.6 Retention in *Population Cohort*

Once a participant is enrolled into the *Population Cohort*, the research team will make every effort to retain him/her for the follow-up surveys at 1, 2, and 3 year time points in order to minimize possible bias associated with loss-to-follow-up. The retention goals for the *Population Cohort* are 90% retained at 12 months, 80% at 24, and 75% at 36 months. Research staff are responsible for developing and implementing local standard operating procedures to reach this goal. Components of such procedures include:

- Thorough explanation of the study visit schedule and procedural requirements during the informed consent process, with re-emphasis at the subsequent 12-monthly study visits.
- Thorough explanation of the importance of their participation to the overall success of the study.
- Collection of detailed locator information at the study Enrollment Visit, and active review and updating of this information at each follow-up visit.
- Immediate and multifaceted follow-up for missed visits.
- Regular communication with the study community at large to increase awareness about HIV/AIDS and explain the purpose of HIV prevention research and the importance of completing research study visits.

In addition to the components described above, which are standard for all HPTN studies, the team will work with local community stakeholders, experienced in-country staff, and participants themselves to identify locally-effective, study-specific strategies for improving participant retention. Such approaches may include use of short message service (SMS) messages to remind participants about upcoming visits, enlisting the assistance of household members to support adherence to study visits and ART adherence, or other methods.

Any member of the *Population Cohort* who leaves the community will be censored regardless of where they move to. If they return during follow-up, they can be included again from the time they first test HIV-negative following their return. For example, if they miss the 12-month follow-up visit, but are present again and still HIV-uninfected at 24-months, they can contribute to HIV incidence data between 24-months and 36-months.

Eligibility criteria for *Population Cohort* enrollment include current residence and intending to remain in the community during follow-up in an attempt to limit loss from the *Population Cohort* due to mobility. Retention rates are broadly in line with experience from previous trials.

Participants may voluntarily withdraw from the study for any reason at any time. The Investigator also may withdraw participants from the study in order to protect their safety and/or if they are unwilling or unable to comply with required study procedures after consultation with the Protocol Chair, Division of AIDS (DAIDS) Medical Officer, Statistical Data Management Center (SDMC) Protocol Statistician, and Coordinating and Operations Center (CORE) Protocol Specialist.

Participants also may be withdrawn if the study sponsor, government or regulatory authorities, or site Institutional Review Board (IRB)/Ethics Committee (EC) terminates the study prior to its planned end date.

5.2 *Population Cross-Sectional Survey* (if funded)

As noted, the *Population Cross-Sectional Survey* described below is currently not funded and is therefore not a part of the current study design. However, the procedures that would be undertaken to implement this activity are described briefly below to illustrate what this work, if funded, would encompass.

5.2.1 Sampling/Recruitment of *Population Cross-Sectional Survey* Participants

A simple random sample of houses will be generated, similar to the method used for the Population Cohort. Research staff will visit the houses in this list in order and all eligible adults in a household will be solicited to participate in the survey. Recruitment will cease when five hundred participants per cluster have been enrolled into the survey.

5.2.2 Inclusion Criteria

- 18 – 44 years of age
- Able and willing to provide informed consent
- Residing within catchment area of a designated local health unit for the three years prior to conduct of the survey
- Residing in a randomly selected household

5.2.3 Exclusion Criteria

- Current enrollment, or enrollment within the prior three years, in another HIV treatment, prevention, or PrEP study
- Current or prior enrollment in an HIV vaccine study
- Anything that, in the opinion of the investigator, would preclude informed consent, make study participation unsafe, complicate interpretation of study outcome data, or otherwise interfere with achieving the study objectives.

5.2.4 Procedures and Activities

***Population Cross-Sectional Survey* Creation**

- Identify a random sample of houses in the community for visits
- Research staff visit selected houses and invite all adult residents (18-44) to participate
- Complete informed consent

Visit Procedures (36 months only)

Administrative, Behavioral, and Regulatory Procedures

- Obtain informed consent for enrollment
- Solicit consent for storage of specimens for future testing
- Obtain/update locator information

- Complete survey to include socio-demographic, health, social, behavioral, and economic factors
- Perform qualitative interviews covering stigma and discrimination (randomly-selected HIV-infected participants only)

Clinical/Counseling Procedures

- Perform HIV rapid tests, if participant agrees
- Provide pre- and post-test counseling and HIV rapid test results, for those willing to receive results
- Collect blood for laboratory testing and sample storage

Laboratory Procedures*

- HIV confirmatory testing (if indicated)
- Plasma storage

*Stored samples will be used for retrospective, centralized testing. This will include HIV testing (to assess HIV prevalence), quality assurance (QA) testing for HIV, antiretroviral drug resistance, and HIV viral load testing (to assess community viral load in a subset of participants). Other testing that may be performed includes: cross-sectional HIV incidence testing and analysis of the linkage of HIV infections. Stored samples may also be used to characterize the HIV virus and the host response to HIV infection. The location of testing for each type of testing will be determined by the HPTN NL. Additional details will be provided in the SSP Manual.

5.3 *Case-Control Studies*

Three *case-control studies* will be undertaken to improve our understanding of participation in three key steps of the intervention, each of which is essential to the success of the trial interventions. These studies will provide information about which factors are associated with non-engagement with particular components of the intervention and will be important for interpreting the findings of the trial, informing mathematical models, and guiding policy.

5.3.1 *Case-Control Study 1* - Uptake of Testing in the First Round of Home-Based Testing Provided by CHiP Teams in Arms A & B

A *case-control study* of refusers (cases) and acceptors (controls) of home-based HIV testing by CHiPs will be undertaken to identify the characteristics of refusers/acceptors and reasons for refusal/acceptance. As this is the first step in the cascade of interventions in Arms A and B, this will be key in interpreting uptake of subsequent steps of the interventions, and will be important for identifying ways to increase testing uptake. The CHiP teams will request permission from individuals declining the intervention to be approached by the research team for potential enrollment into the case-control studies.

5.3.1.1 Sampling/Recruitment of *Case-Control Study 1* Participants

Four hundred cases (refusers of CHiP testing) and 400 randomly selected controls (acceptors) from the communities in Arms A and B will be enrolled. Potential participants will be selected at random and approached by CHiP personnel, who will seek verbal consent for follow-up by a research team. The latter will then obtain the formal informed consent for *case-control study* participation. Recruitment will cease when 400 participants have been enrolled in each of the two groups.

5.3.1.2 Inclusion Criteria *Case-Control Study 1*

- At least 18 years of age
- Able and willing to provide informed consent
- Resident in the cluster during the first round of testing
- Visited by a CHiP team and offered testing during the first round of home-based testing

5.3.1.3 Exclusion Criteria *Case-Control Study 1*

- Individuals belonging to the *Population Cohort* or other case-control studies
- Individuals known to be HIV-infected after testing elsewhere.

5.3.1.4 Procedures and Activities

<u>Administrative, Behavioral, and Regulatory Procedures</u>

- Obtain informed consent for enrollment
- Complete questionnaire of socio-demographic, clinical, and behavioral characteristics

Standardized questionnaires will encompass sexual and health seeking behavior, previous HIV testing, as well as stigma and psycho-social questions. Cases and controls will also have separate sections in the questionnaire, to explore reasons for not testing and motivation to test, respectively. The standardized surveys will be carried out by *case-control study* teams after the end of the first CHiP home-based testing round within a community, at the household or an alternative community location chosen by the participant.

5.3.2 *Case-Control Study 2* - Uptake of Immediate Treatment in Arm A

A *case-control study* of cases (who do not start ART within 3 months of testing positive) and controls (who start ART within 3 months of testing positive) in a random sample of individuals from Arm A communities will be undertaken to identify the characteristics of those who do/do not start ART within 3 months and reasons for starting/not starting. As immediate treatment is the linchpin of the PopART intervention, understanding the barriers to wide-scale uptake (if any) will be crucial in understanding the trial findings.

5.3.2.1 Sampling/Recruitment of *Case-Control Study 2* Participants

Four hundred cases (non-receivers of ART within 3 months after first receiving an HIV positive test from a CHiP, or disclosing previously-diagnosed HIV infection to a CHiP) and 400 randomly selected controls (receivers of ART within this timeframe) from the communities in Arm A will be enrolled. Potential participants will be selected at random and approached by CHiP personnel, who will seek verbal consent for follow-up by a research team. The latter will then obtain the formal informed consent for *Case-Control Study 2* participation. Recruitment will cease when 400 participants have been enrolled in each of the two groups.

5.3.2.2 Inclusion Criteria *Case-Control Study 2*

- At least 18 years of age
- Able and willing to provide informed consent
- Resident in the cluster during the first round of testing
- Tested HIV-infected in CHiP home-based testing, or HIV-infected and disclosed that they were previously diagnosed as HIV-infected to CHiP team

5.3.2.3 Exclusion Criteria *Case-Control Study 2*

- Individuals enrolled in the *Population Cohort* or other *case-control studies*
- HIV-infected individuals already on ART before study commences

5.3.2.4 Procedures and Activities

Administrative, Behavioral, and Regulatory Procedures

- Obtain informed consent for enrollment
- Complete questionnaire of socio-demographic, clinical, process uptake and behavioral factors

Standardized questionnaires will encompass sexual and health seeking behavior, as well as stigma and psycho-social questions. Cases and controls will also have separate sections in the questionnaire, depending on whether: i) they did not attend the health center in the first place (cases), ii) attended but did not initiate immediate treatment (cases), iii) attended and accepted immediate treatment (controls) to explore their reasons for not starting ART within 3 months or motivation to start immediate treatment. The standardized surveys will be carried out by *case-control study* teams approximately 3 months after the end of the first CHiP home-based testing round within a community, at the household or an alternative community location chosen by the participant.

5.3.3 *Case-Control Study 3* - Uptake of Testing in the Second Round of Home-Based Testing Provided by CHiP Teams in Arms A & B

A *case-control study* of refusers (cases) and acceptors (controls) of home-based HIV testing by CHiPs in the second round of testing will be undertaken to identify the characteristics of refusers/accepters and reasons for refusal/acceptance at this stage. Because regular re-testing of individuals who were HIV-uninfected when last tested is a crucial step in the cascade of interventions in Arms A and B, the understanding of reasons

for not accepting CHiP home-based testing in the second round is key for interpreting uptake of subsequent steps of the interventions, and for identifying ways to increase the uptake of re-testing. The CHiP teams will request permission from individuals declining the intervention to be approached by the research team for potential enrollment into the case-control studies.

5.3.3.1 Sampling/Recruitment of *Case-Control Study 3* Participants

Four hundred cases (refusers of CHiP testing) and 400 randomly selected controls (acceptors) from the communities in Arms A and B will be enrolled. Potential participants will be selected at random and approached by CHiP personnel, who will seek verbal consent for follow-up by a research team. The latter will then obtain the formal informed consent for case-control study participation. Recruitment will cease when 400 participants have been enrolled in each of the two groups.

5.3.3.2 Inclusion Criteria *Case-Control Study 3*

- At least 18 years of age
- Able and willing to provide informed consent
- Resident in the cluster during the second round of testing
- Visited by a CHiP team and offered testing during the second round of home-based testing

5.3.3.3 Exclusion Criteria *Case-Control Study 3*

- Known HIV infected from CHiP data.
- Individuals belonging to the *Population Cohort* or other case-control studies

5.3.3.4 Procedures and Activities

<u>Administrative, Behavioral, and Regulatory Procedures</u>

- Obtain informed consent for enrollment
- Complete questionnaire of socio-demographic, clinical, process uptake and behavioral factors

Standardized questionnaires will encompass sexual and health seeking behavior, previous HIV testing, as well as stigma and psycho-social questions. The primary analysis will compare refusers and acceptors of testing at this stage. There will also be sub-group analyses to consider participants who: (i) accepted, tested and were found negative at the first round, (ii) refused testing at the first round, (iii) were absent at the baseline testing round (away from home or newly moved into community). The standardized surveys will be carried out by *case-control study* teams at the end of the second CHiP home-based testing round (i.e. 12 month round) within a community, at the household or an alternative community location chosen by the participant.

5.4 *Qualitative Studies*

Qualitative studies will be conducted in both Zambia and South Africa by an experienced social science team. The research will be conducted in two phases. A first rapid phase

using participatory social research methods carried out in all communities will be described and conducted in an ancillary protocol. A second in-depth phase, with a longitudinal component, related to the different arms of the trial and core questions around uptake and outcomes is described below.

The first phase will identify key features of each community (including social organization and networks) and will involve community mapping of the history of ART, local HIV prevention initiatives, HIV treatment and support services, and key stakeholders (including other HIV research studies). This and initial work on community attitudes to different prevention methods will help to inform the design and delivery of the trial interventions (including the design and content of information/sensitization messages and instruments) and to enable effective stakeholder co-ordination in all communities. In principle, the qualitative studies will work closely with the community engagement process throughout the study.

In the second phase, the qualitative research will have three core components, namely: qualitative research evaluating the acceptability of the intervention including, critically, the acceptability and functioning of the CHiPs and the process of community engagement; a qualitative longitudinal study of representative individuals nested within the first *Case-Control* study described above; and an ethnographic component. These are briefly detailed below.

5.4.1 Evaluation of the Acceptability of the Intervention:

In Arms A and B, social science research will be carried out at community level using a mix of social research methods (including fieldworker structured diaries, in-depth interviews, focus-group discussion, participatory rapid appraisal tools, participant observation, structured observation) to assess over time popular understanding of HIV testing and treatment and how communities actually respond to the combination prevention intervention, including linkage to care and the innovation of immediate HIV treatment. This research component will be carried out throughout the intervention period at intervals linked to the intervention timeline – e.g. at the outset, three months into the intervention, a year into the intervention and towards the end of the intervention.

Building on the rapid formative research, qualitative insights will be collected in a structured diary form throughout the intervention period from all communities using resident fieldworkers who would dedicate a few days a month to document local response. More in-depth work will also be carried out in communities of a certain type. In these communities, roughly 100 participants, including key local stakeholders, CHIP teams and different age and gender groups from the community, will be questioned about the acceptability of the intervention, any problems experienced or foreseen, and suggested solutions to these problems, and findings will be fed back into community engagement and trial practice. Research on the process of community engagement and the application of ethical guidelines will also be embedded within this component. In addition, this component will include any urgent research on significant events at community level (e.g. significant rumors including Satanism accusations, community withdrawal, explicit confrontation with faith healing or other alternative prevention options) which threaten the continuation or practice of the trial and require qualitative investigation.

5.4.2 Qualitative Longitudinal Study in Arms A and B – sub-set of *Case-Control Study 1*

A small number (roughly 12 in each selected community) of representative individuals from *Case-Control Study 1* will be enrolled and seen longitudinally over the intervention period in selected communities across Arms A and B to explore and document the longitudinal trajectory of individual behavior in relation to uptake of HIV testing and treatment, complementing the findings of the case-control studies.

Individuals who have refused testing at baseline and individuals who have accepted testing with different outcomes (tested HIV-uninfected or HIV-infected) from different genders, age groups, and socio-demographic backgrounds will be selected and approached to participate in this longitudinal study. This cohort of individuals would be recruited following their participation in *Case-Control Study 1*– with the first in-depth interview taking place soon after the *Case-Control Study 1*survey, and subsequent in-depth interviews being held at three month intervals until the end of the intervention period. This research will document experiences over time and establish how the micro-level continuum of experiences influence decision making processes related to uptake of HIV testing and treatment services. Additional locator information will be collected and separate informed consent obtained for the study for each visit.

A mix of methods will be used including semi-structured interviews, observations and respondent records of significant events pertaining to individual health and health seeking behavior. In-depth interviews will be conducted by local case-control research assistants supervised by a social scientist.

5.4.3 Ethnography of the HIV landscape

This component aims to provide more contextual and comparative understanding of how communities are experiencing the roll-out of UTT, including immediate HIV treatment. The inquiry will build on and extend current knowledge of the impact of ART on HIV stigma, the long-term realities of ART in low-resource settings, the influence of alternative prevention options, the role of welfare and food insecurity in shaping uptake of ART, popular knowledge of ART, sexual risk disinhibition, alcohol and drug use, gender-based violence, HIV identity, the reproductive health of people living with HIV, the acceptability of and response to male circumcision, the influence of local systems, social networks and community morale, and the role of different stakeholders. This ethnographic research will use a mix of social research methods – with the most key method being the continued presence of a social scientist in a community over a period of 3-6 months, mostly likely 6-18 months into the intervention period. It will be carried out in two communities in each country – with one community selected from Arms A and C – and most of the inquiry will be carried out at household level.

5.4.4 Graphical Summary of Qualitative Activities

A summary of the timing, flow and logic of the qualitative activities is provided in Figure 4.

Figure 4- Qualitative Activities in HPTN 071

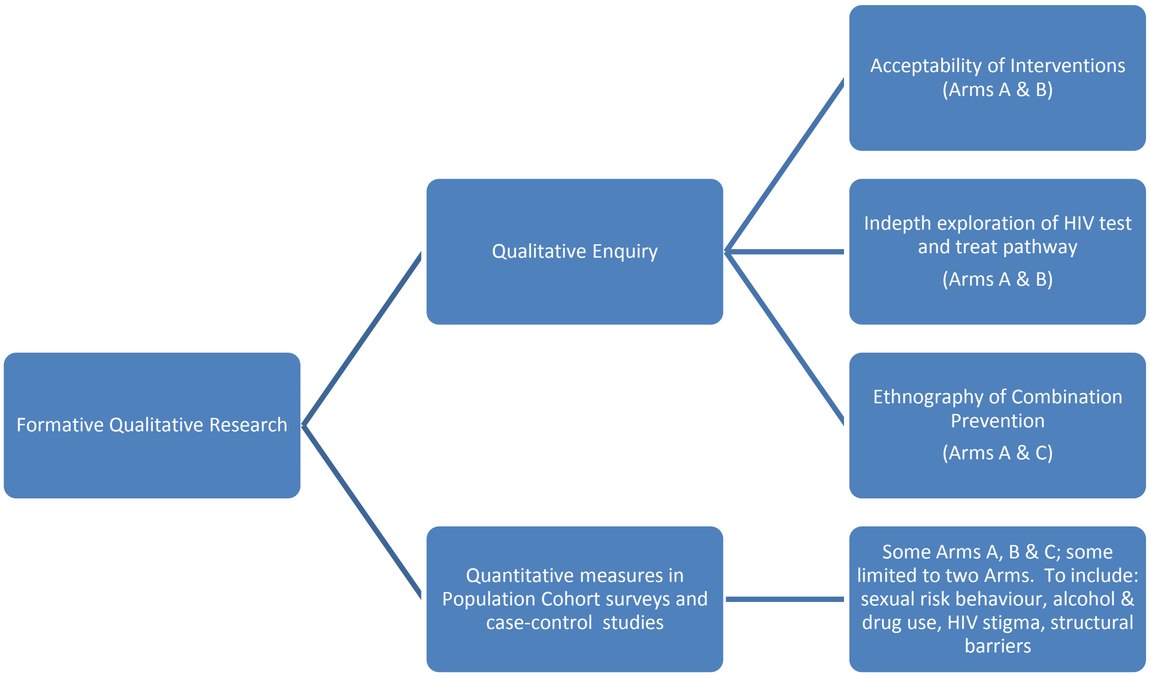

5.4.5 Integration of Data from Case-Control and Qualitative Components

The social science team will be involved in helping to develop the themes and questions for the case-control studies. Within the case-control studies there is a qualitative component that aims to provide a more detailed picture of HIV testing and treatment pathways for a small number of representative individuals. The lead investigator for the case control studies will work closely with a social scientist who will carry out the qualitative component as well as supporting the quantitative measures/data collection. Broader ethnographic enquiry will also explore core themes (all related to secondary outcomes).

5.5 Collection of Health Center-Based Data

In addition to the conduct of specific surveys described in previous sections, routine health center-based data in all study communities will be used to measure several secondary outcomes. To maximize the validity of this information, the research team will work with the health centers to improve the collection and management of these routine data and to the extent possible, harmonize these processes across health centers.

5.5.1 Tuberculosis Case Notification

TB cases in the study communities are routinely diagnosed and treated at the same health centers as those delivering HIV treatment and care. In all of the study communities, the TB notification process will be strengthened by the use of additional diagnostic tests and

enhanced monitoring of the TB case registration system. Data from this system will be compiled at regular intervals during the trial and used to measure the following outcomes:

- Notification rate of bacteriologically confirmed pulmonary tuberculosis
- Mortality rate of bacteriologically confirmed pulmonary tuberculosis

These data will be collected for each time period, and will be classified according to HIV status.

5.5.2 Intervention Effect on Health Center Workload

To address concerns that the intervention may substantially add to the case-load of clinics, data from clinic registers will be compiled at regular intervals to determine the total numbers of outpatient and inpatient attendances. These data will be collected at regular time intervals, and efforts will be made to broadly classify medical reasons for attendance.

5.5.3 Intervention Effect on Healthcare Costs

A multi-step procedure will be used to determine the impact of the intervention on healthcare costs. First, data will be collected on individuals' healthcare utilization by self-report of the members of the *Population Cohort*. Data will be collected on use of outpatient healthcare facilities (number of visits) and secondary and tertiary facilities (number of visits, duration of visit if overnight). Direct costs of providing care to individuals will also be evaluated, including travel costs and reimbursements by third parties (e.g. private insurers). In addition, *Population Cohort* patient records held at the healthcare facilities along with CHiPs data will be reviewed where available to obtain detailed information on healthcare use across treatment arms. We will use both participant self-report and clinic records in order to generate population level estimates of changes in health care utilization, document care sought from providers where linkage to records is not possible, and collect data on patients' cost of seeking care. Second, facility level costs will be collected in all facilities in selected trial communities. Both one-time capital costs (e.g. investments in buildings) and recurrent costs (e.g. salaries) will be collected. Lastly, facility level costs will be apportioned to the visits reported by the cohort members by applying average costs for typical use of healthcare facilities. For members with linked patient records, we will calculate more precise cost estimates of health care use based on actual treatments provided. This final analysis will be based on various assumptions, which will be tested with sensitivity analysis.

5.5.4 HIV Disease Progression and Death

Aggregate data from the health center database for health center attendees on ART such as WHO staging events (including opportunistic infections such as TB), hospitalizations (where documented), CD4 cell counts, and death will be used to monitor effects of the interventions on HIV disease progression and death.

5.5.5 ART Adherence and ART Toxicity

To assess the rates of ART adherence under different intervention conditions, aggregate data will be collected from all health centers about missed follow-up visits among those on ART and missed dispensations of ARV drugs. To assess rates of ART toxicity under

different intervention conditions, aggregate clinical data related to ART related side effects, ART drug interruptions and treatment switches will also be collected from each health center.

5.5.6 Uptake of Intervention Components

Process measures of the uptake of key components of the intervention will be measured in Arms A and B using data from the health centers on the rates of utilization of PMTCT services and medical male circumcision and the proportion of community members initiating HIV care within three months of receiving an HIV diagnosis.

5.6 Proposed Additional Surveys

Three additional surveys have been proposed to support or enhance the research described above. The three surveys have not been described in detail in the body of this protocol because funding is not available for them at the current time. However, each is briefly described below and described in detail in Appendices VIII, VI and X.

Population Cross-Sectional Survey (Appendix VIII)
Because participants in the *Population Cohort* will be followed longitudinally over 3 years, their interactions with the research staff could bias the data they provide for certain outcome measures. The *Population Cross-Sectional Survey* would be a snapshot evaluation to provide unbiased data for comparison on many of the measures evaluated in the *Population Cohort*. Approximately 500 participants per cluster would be recruited from randomly-selected homes for this survey, to be conducted at the end of the second year of the intervention. Procedures would include a questionnaire and blood sampling for HIV testing and sample storage.

TB Prevalence Survey (Appendix IX)
ART treatment has been shown to reduce the risk of developing TB and to reduce mortality from TB at the individual level, but the effects of ART on community-level TB is not known. Mathematical modeling suggests that universal HIV testing and increased access to ART could reduce the incidence of TB rapidly. This study presents an ideal opportunity to measure the effect of such a universal HIV test-and-treat strategy on prevalence of TB in the community. The proposed survey would involve collecting and testing sputum for TB at the end of the follow-up period from 4250 individuals in each community, drawing participants from all adult members of households in which the *Population Cohort* and *Population Cross-Sectional Survey* would be conducted.

PMTCT Survey (Appendix X)
In the current study design, the effect of the PopART intervention on uptake of PMTCT services will be evaluated by self-report and health center data. However, this study presents an ideal opportunity to evaluate the impact of universal testing and immediate eligibility for ART treatment on actual health outcomes in the form of HIV-free survival of infants. In this survey, women who have given birth in the last 36 months and who live in a household in which either the *Population Cohort* or *Population Cross-Sectional Survey* are conducted will be asked at the end of the follow-up period if their child is alive, and to provide a blood sample from themselves for rapid testing. For women who are HIV infected, the team will ask for a blood spot from the child for HIV testing as well.

This would allow comparison of infant HIV-free survival in intervention communities versus control communities.

5.7 Comparative Table of Study Activities across All Study Arms

Table 3- Study Activities across All Study Arms

Study Procedures/ Activity	Arm A	Arm B	Arm C
**Strengthening the provision of HIV services in the community**			
Endeavour to ensure ART service delivery to at least national guidelines	X	X	X
Endeavour to ensure PMTCT services to at least national guidelines	X	X	X
Endeavour to ensure STI treatment to at least national guidelines	X	X	X
Endeavour to ensure male circumcision services to at least national guidelines	X	X	X
Promotion of voluntary counseling and testing at non-HIV clinics and other venues	X	X	X[1]
Opt-out provider-initiated counseling and testing	X	X	X[1]
**Implementation of interventions** including deployment of CHiP teams to all houses in the community			
Offering initial and recurrent HIV testing and counseling to all household members 16 years of age and older (or younger, upon request and with parental consent)	X	X	
Linkage-to-care for HIV-infected persons with			
Immediate eligibility for ART	X		
Eligibility for ART based on national guidelines		X	
Referral of willing HIV-uninfected men for circumcision	X	X	
Referral of pregnant, HIV-infected women to PMTCT services or immediate ART	X	X	
Ongoing promotion of ART adherence and HIV prevention services during the study period	X	X	
**Enrollment and follow-up of *Population Cohort*** by research team			
Informed consent	X	X	X
Offer of HIV rapid test and counseling	X	X	X
Complete survey to include socio-demographic, health, social, behavioral, and economic factors	X	X	X
Blood draw and laboratory-based testing<br>See Sections 5.1 and Appendix IA	X	X	X
**Execution of a *Population Cross-Sectional Survey*** at 36 months (if funded)			
Same procedures as for Population Cohort, but without HSV-2 testing	X	X	X
**Conduct of qualitative studies**			
Informed consent	X	X	X
Completion of qualitative data collection	X	X	X

**Conduct of *Case-Control* studies**			
Informed consent	X	X	
Completion of behavioral questionnaire	X	X	
**Conduct of Additional Surveys (if funded)**			
TB Survey	X	X	X
PMTCT Survey	X	X	X

[1]Where these are already provided locally as standard services.

6.0 SAFETY MONITORING AND SOCIAL HARM REPORTING

6.1 Safety Monitoring

All drugs used in this study for the treatment of HIV have regulatory approval for this purpose in both Zambia and South Africa and are widely used with well-established safety profiles. Community members receiving ART will be seen by the regular staff at the health center for their care and will receive safety assessments according to local standard of care. Data from these tests are not routinely entered into electronic medical records at the health center. The research team therefore will not have access to, nor monitor or report adverse events/serious adverse events (AE/SAEs) for community members on ART. Instead, information about the impact of the different interventions on the health and safety of community members on ART will be assessed through analysis of aggregate, anonymous data from the health centers, and data from *Population Cohort* participants, including measures of HIV disease progression and death, drug adherence, ART toxicity, and, if funded, viral suppression, drug resistance, and community viral load.

6.2 Social Harm Reporting

The HPTN defines social harms as any untoward social occurrences that happen to a **participant as a result of their participation in the study,** with examples including loss of employment, harassment by neighbors, shunned by family, rejection by partner, etc. Because this study is a community-randomized trial of a multi-faceted intervention, the majority of people in the community affected by the implementation of the study will not be participants in the evaluation surveys, and so the definition of social harm for this study will be expanded to also include **any untoward social occurrences that happen to a community, or groups or individuals within a community, as a result of implementation of the study intervention**. Social harms will be monitored throughout the study.

It is important to note that the number of people who live in the communities involved in this study is very large and the number of social harms *unrelated to the study intervention or study participation* that will occur during the trial period is expected to be very high due to social, economic and cultural factors unrelated to the study. Therefore it will be important that study staff are well trained to report only those social harms that they deem to be directly related to the intervention, or participation in the research program.

6.2.1 Participants in the *Population Cohort*

The *Population Cohort* is intended to provide study data representative of the populations from which it is drawn, and this will apply for social harms monitoring as well. Information on social harms experienced by cohort participants - either because of the deployment of the intervention in their community or because of their individual participation as research subjects in the cohort - will be actively solicited from participants at follow-up visits and captured in the study database. When a cohort participant reports a social harm, every effort will be made by the study staff to provide appropriate counseling to the participants, and/or referral to appropriate resources, as needed.

6.2.2 Community at Large

Monitoring of social harms in the community will be accomplished using several approaches. In each household during their annual testing visit, CHiPs will inquire about any social harms due to the implementation of the intervention in their community, and will document qualifying harms in the study database. Because study staff work intimately among, and are often from, the randomized communities, they may become aware on a passive basis of social harms that are occurring within the community. Staff will report these harms as well. The study team will include discussion of social harms as one of the topics regularly covered in work with the community liaison board in each community, and will report on any harms reported in those meetings. Finally, the qualitative research to be conducted includes exploration of social harms in the community.

6.2.3 Social Harm Monitoring

The study management team will review the social harms reports on a quarterly basis, or sooner, if a concerning trend or event is identified. If the management team judges an individual social harm, or a trend in social harms, to be serious or unexpected, they will work together with appropriate bodies (in-country investigator, community liaison board, sponsor, IRB, etc.) to determine if a response is indicated, and if so, what it should be. The nature and frequency of reported social harms will be reviewed by the HPTN Study Monitoring Committee (SMC). Investigators of record will report serious or unexpected social harms to the responsible IRB/EC at least annually, or according to their individual requirements. The study team notes that although most of the activities for detecting social harms listed above will be conducted equally in all three arms of the study, CHiPs will only be deployed in Arms A and B. Therefore a greater number of reported social harms may be seen in these arms, relative to Arm C, due to differential ascertainment, rather than differential incidence of harms, a possibility that will be considered when reviewing trends in social harms.

7.0 STATISTICAL CONSIDERATIONS AND DATA ANALYSIS

7.1 Sample Size

The trial has been powered to detect intervention impact on the primary endpoint, and on key secondary endpoints, as detailed below. All sample size calculations have been carried out using methods for matched cluster-randomized trials.

7.1.1 Mathematical Modeling and Sample Size Calculations

The development of the interventions has been guided by the results of mathematical modeling. Early work on the intervention was based on the papers by Granich et al. Subsequently, the modeling team at Imperial College developed a model fitted to current UNAIDS prevalence data from Zambia and South Africa and used it to predict the impact of the proposed packages of interventions relative to the standard of care arm (Figure 5).

Figure 5- Mathematical model of the epidemic and of the PopART interventions

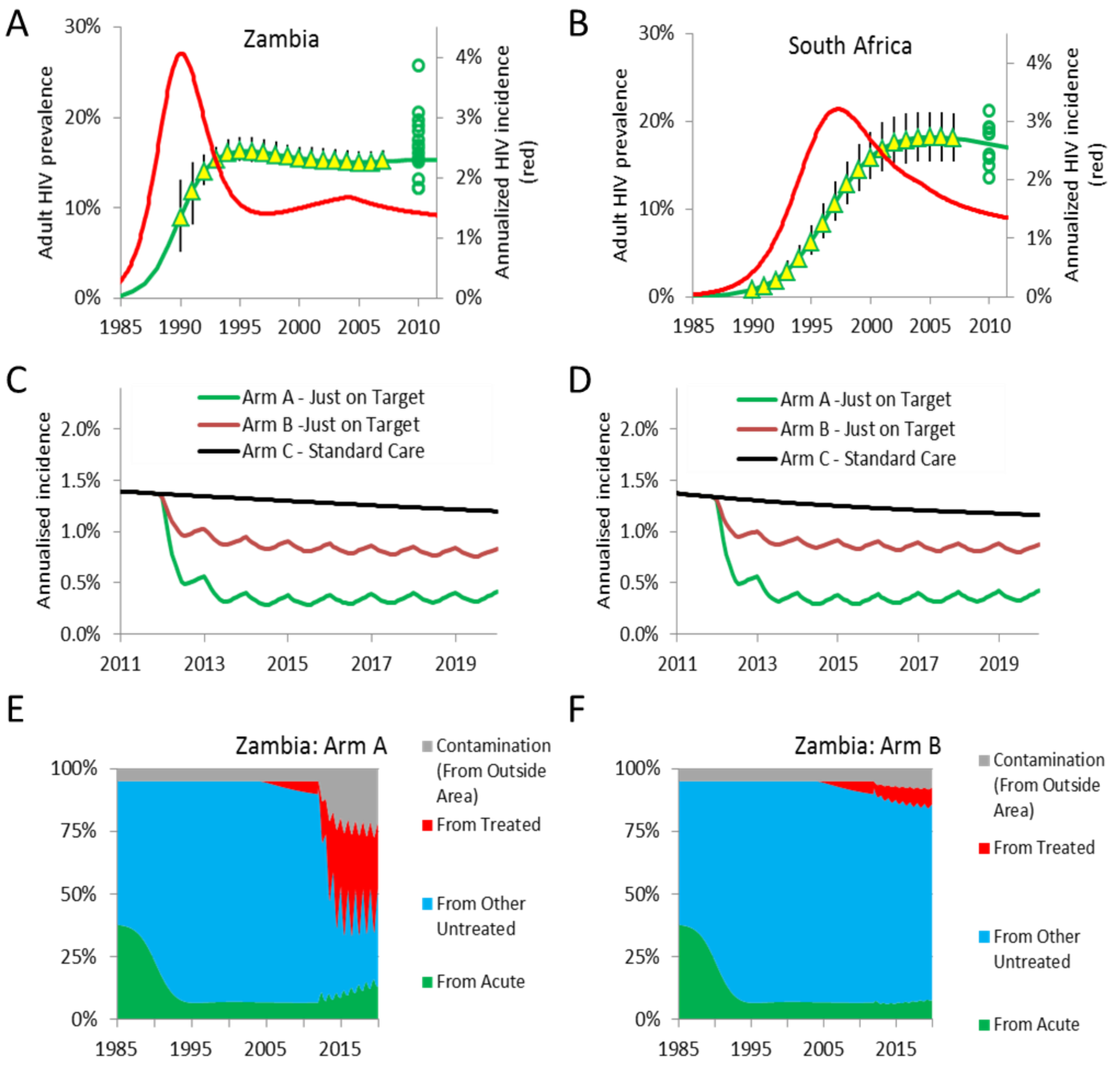

A model was developed to aid development of the trial protocol, and more specifically to develop targets for the process variables (coverage, contamination, etc.), and to provide scenarios for the power calculations. The model is a conventional HIV epidemic model, and has been validated by a recent systematic model comparison exercise (Eaton et al, submitted). The model is calibrated to country-specific UNAIDS data on adult HIV prevalence (green lines in **A** and **B**). Prevalence in the 24 ZAMSTAR communities in 2010 is shown by the green circles, and the predicted incidence curves are shown as red lines. **C** and **D**, starting from 2012, the PopART intervention package is implemented; the packages are implemented in six-monthly cycles, which results in a characteristic 'saw-tooth' pattern in incidence. The Just-on-target scenarios are based on the optimistic scenario (75% annual coverage, 95% treatment efficacy, 5% contamination, 50% uptake of male circumcision, 10% annual drop out and no behavior change). **E**, predicted sources of infection for incident cases for Arm A in Zambia, and **F** as **E** for Arm B.

.

Briefly, the model assumes three sexual activity classes, and the proportions in each class, the assortativity in sexual mixing, rates of partner acquisition and HIV transmissibility are fitted to the prevalence data in each country. The model assumes that male circumcision reduces HIV acquisition by 60%, with rates of male circumcision based on data from the study communities (Table 2- Section 4.1). ART roll-out is assumed to commence in 2004 with coverage amongst those with CD4<200 and CD4<350 matched to ZAMSTAR data.[101] The model includes variable infectivity by stage of infection, matched to data from the Rakai study.[67, 102] To allow for contamination, we assumed that 5% of sexual contacts occur with partners from outside the study community. The fit of the model to the HIV prevalence data is considered to be good.

The model fits assume that interventions commence in 2012, and that during each annual round of testing in Arms A and B, the intervention is delivered over a period of 26 weeks. Figure 5 shows projected HIV incidence over time for Arms A and B compared with the control arm for the optimistic target scenario. Table 4 shows the assumptions made for the central and optimistic target scenarios and the projected impact on cumulative HIV incidence over three years, over the first two years, and also in each year separately, for Arms A and B compared with Arm C. The projections indicate that an impact is expected over three years of 55-65% in Arm A and 20-30% in Arm B. Impact is substantially higher in Years 2 and 3 as expected. As a sensitivity analysis, assuming roll-out takes 12 rather than 6 months, projected impact over three years is 50-60% for Arm A and 20-30% for Arm B (Table 5).

Table 4- Parameter values assumed for the model of the impact of the intervention for central and optimistic target scenarios, and projected impact on HIV incidence in Arms A and B compared with Arm C, assuming intervention roll-out over a 6-month time period

	Parameter	Central Target		Optimistic Target	
	Annual coverage of test and treat campaign	70%		75%	
	Treatment failure & drop-out rate, per year	10%		10%	
	Effectiveness of ART in blocking transmission	90%		95%	
	Take up of male circumcision when offered	50%		50%	
		Arm A	Arm B	Arm A	Arm B
Zambia	Impact on cumulative incidence (3 years)	58%	25%	66%	29%
Zambia	Impact on cumulative incidence (2 first years)	54%	23%	62%	27%
Zambia	Impact on HIV incidence during Year 1	45%	19%	53%	23%
Zambia	Impact on HIV incidence during Year 2	63%	28%	72%	33%
Zambia	Impact on HIV incidence during Year 3	68%	31%	76%	36%
South Africa	Impact on cumulative incidence (3 years)	57%	23%	65%	27%
South Africa	Impact on cumulative incidence (2 first years)	52%	21%	61%	26%
South Africa	Impact on HIV incidence during Year 1	44%	18%	52%	23%
South Africa	Impact on HIV incidence during Year 2	62%	26%	71%	31%
South Africa	Impact on HIV incidence during Year 3	66%	29%	75%	33%

Table 5- Parameter values assumed for the model of the impact of the intervention for central and optimistic target scenarios, and projected impact on HIV incidence in Arms A and B compared with Arm C, assuming intervention roll-out over a 12-month time period

Parameter		Central Target		Optimistic Target	
Annual coverage of test and treat campaign		70%		75%	
Treatment failure & drop-out rate, per year		10%		10%	
Effectiveness of ART in blocking transmission		90%		95%	
Take up of male circumcision when offered		50%		50%	
		Arm A	Arm B	Arm A	Arm B
Zambia	Impact on cumulative incidence (3 years)	54%	23%	62%	27%
	Impact on cumulative incidence (2 first years)	47%	20%	56%	25%
	Impact on HIV incidence during Year 1	34%	14%	42%	19%
	Impact on HIV incidence during Year 2	61%	27%	70%	32%
	Impact on HIV incidence during Year 3	67%	31%	76%	36%
South Africa	Impact on cumulative incidence (3 years)	52%	21%	61%	26%
	Impact on cumulative incidence (2 first years)	46%	19%	54%	23%
	Impact on HIV incidence during Year 1	33%	14%	41%	18%
	Impact on HIV incidence during Year 2	60%	26%	69%	30%
	Impact on HIV incidence during Year 3	66%	29%	75%	33%

The targets appear achievable based on published evaluations of interventions in Africa. Because there is most uncertainty in the effects on behavior change, the model conservatively assumed no effect on behavior when deriving these targets. Process indicators will be monitored as described in Sections 7.1.3 (6) and 7.10, and used to modify and adapt the intervention as necessary.

7.1.2 Primary Endpoint - HIV Incidence Over 36 Months

The incidence of HIV infection among initially HIV-uninfected *Population Cohort* members will be measured during the follow-up period of 36 months. Based on national estimates of HIV incidence and on HIV prevalence in the chosen study areas, it is expected that HIV incidence in the control arm will be in the range 1.0-1.5/100py. With a matched study design, and based on the between-community variation in HIV prevalence observed in the 2010 survey of several thousand adults in each of the trial communities, and the between-community variation in HIV incidence among adults living in the households of TB cases during 2006-2010, it is expected that the between-community coefficient of variation will be in the range 0.15-0.20. Seven communities were chosen per study arm and a *Population Cohort* of 2,500 adults per community to attain adequate power to detect a difference in incidence between Arms A and C (reflecting the full impact of the intervention), as well as the difference in intervention effect between Arms A and B (reflecting the additional effect of immediate HIV treatment compared with current national guidelines). Based on mathematical modeling, the anticipated effect of Arms A and B is to reduce cumulative HIV incidence over a three-year period by 55-65% and 20-30% respectively, compared with Arm C (Figure 5), with a difference in impact

between Arms A and B of about 30-35%. A standard formula for cluster-randomized trials was used for the comparison of incidence rates over 36 months, with matched triplets as the trial design[103].

Table 6 shows that the study will be very well powered to detect an effect of 35% or larger in Arm A compared with Arm C, and moderately well powered to detect an effect of 30% under favorable assumptions. For the direct comparison of Arms A and B, Table 7 shows that the study will be well powered to detect a difference between effects of 60% and 30%, 55% and 25%, and 50% and 20%. Tables 6 and 7 allow for a baseline HIV prevalence of 15% and assume losses to follow-up of 20% over two years, and 25% over three years.

Table 6- Power for comparison of HIV incidence in Arm A or B with Arm C, with 7 communities per arm and Population Cohort of 2500 adults per community (assuming that on average 2125 (85%) will be HIV-uninfected at baseline and that loss to follow-up will be 20% after 2 years and 25% after 3 years) with 5206 person-years per community over 36 months (assuming 1912 person-years 0-12 months; 1700 person-years 12-24 months; 1594 person-years 24-36 months)

HIV incidence rate/ 100py (control arm)	Between-cluster coefficient of variation (k)	Effectiveness (%)	Power (%)
1.0	0.15	25%	57%
1.0	0.15	30%	74%
1.0	0.15	35%	87%
1.0	0.15	40%	95%
1.0	0.15	45%	99%
1.0	0.15	50%	100%
1.0	0.15	55%	100%
1.0	0.15	60%	100%
1.0	0.15	65%	100%
1.0	0.20	25%	44%
1.0	0.20	30%	60%
1.0	0.20	35%	75%
1.0	0.20	40%	87%
1.0	0.20	45%	94%
1.0	0.20	50%	98%
1.0	0.20	55%	99%
1.0	0.20	60%	100%
1.0	0.20	65%	100%
1.5	0.15	25%	64%
1.5	0.15	30%	81%
1.5	0.15	35%	92%
1.5	0.15	40%	98%
1.5	0.15	45%	100%
1.5	0.15	50%	100%
1.5	0.15	55%	100%
1.5	0.15	60%	100%
1.5	0.15	65%	100%
1.5	0.20	25%	48%
1.5	0.20	30%	65%
1.5	0.20	35%	80%
1.5	0.20	40%	91%
1.5	0.20	45%	96%

1.5	0.20	50%	99%
1.5	0.20	55%	100%
1.5	0.20	60%	100%
1.5	0.20	65%	100%

Table 7- Power for comparison of HIV incidence between Arms A and B, with 7 communities per arm and Population Cohort of 2500 adults per community (assuming that on average 2125 (85%) will be HIV-uninfected at baseline and that loss to follow-up will be 20% after 2 years and 25% after 3 years)

HIV incidence rate/ 100py (control arm)	Between-cluster coefficient of variation (k)	Effectiveness (%) Arm A	Effectiveness (%) Arm B	Power (%)
1.0	0.15	50%	20%	89%
1.0	0.15	50%	25%	78%
1.0	0.15	55%	25%	92%
1.0	0.15	55%	30%	82%
1.0	0.15	60%	25%	98%
1.0	0.15	60%	30%	94%
1.0	0.20	50%	20%	78%
1.0	0.20	50%	25%	65%
1.0	0.20	55%	25%	83%
1.0	0.20	55%	30%	71%
1.0	0.20	60%	25%	93%
1.0	0.20	60%	30%	87%
1.5	0.15	50%	20%	94%
1.5	0.15	50%	25%	86%
1.5	0.15	55%	25%	96%
1.5	0.15	55%	30%	90%
1.5	0.15	60%	25%	99%
1.5	0.15	60%	30%	98%
1.5	0.20	50%	20%	84%
1.5	0.20	50%	25%	72%
1.5	0.20	55%	25%	88%
1.5	0.20	55%	30%	78%
1.5	0.20	60%	25%	96%
1.5	0.20	60%	30%	92%

While the study is adequately powered to answer the primary research question, it has not been powered to undertake any stratified analysis by country or to assess difference in impact between countries.

7.1.3 Secondary Endpoints

NB: Tables showing the calculations for secondary endpoints are provided in Appendix VII.

(1) HIV Incidence During Months 12-24, and Months 24-36 from Start of Intervention

Assuming baseline HIV prevalence of 15% and loss to follow-up of around 20% by the end of Year 2 and 25% by the end of Year 3, this incidence estimate will be based on a sample size of approximately 1700 person-years per community in Year 2 and 1594 person-years per community in Year 3.

Our model projections show that under the optimistic scenario the impact on HIV incidence during Year 2 will be 70% and 30-35% in Arms A and B respectively, and for the central target it will be 60% and 25-30% respectively, with a difference in impact between Arms A and B of about 35%. For a comparison of Arm A with Arm C, study power is 96% or higher with the central target of a 60% reduction, with k up to 0.20 and HIV incidence in Arm C of at least 1 per 100 person-years. For a comparison of Arm B with Arm C, study power is 71% with the optimistic target of a 35% reduction, k=0.15 and HIV incidence in Arm C of 1.5 per 100 person-years, but lower than this for the central target, and/or higher k, and/or lower HIV incidence in Arm C. For comparison of Arms A and B, we will have moderate power (around 70% or more) for the central target and 1% HIV incidence, and good power (>80%) for all other conditions.

Our model projections show that under the optimistic scenario the impact on HIV incidence during Year 3 will be approximately 75% and 35% in Arms A and B respectively, and for the central target it will be 65-70% and 30% respectively, with a difference in impact between Arms A and B of about 35%. For a comparison of Arm A with Arm C, study power is 98% or higher with the central target of a 65% reduction, with k up to 0.20 and HIV incidence in Arm C of at least 1 per 100 person-years. For a comparison of Arm B with Arm C, study power is 69% with the optimistic target of a 35% reduction, k=0.15 and HIV incidence in Arm C of 1.5 per 100 person-years, but lower than this for the central target, and/or higher k, and/or lower HIV incidence in Arm C. For comparison of Arms A and B, we will have good power for the central target of 65-70% vs 30% (74%-97% power depending on assumptions).

(2) Community Viral Load 12, 24, and 36 Months after the Start of Intervention

In the *Population Cohort* at 24 months, viral load will be measured in all HIV-infected individuals (irrespective of seroconversion date), estimated to be approximately 300 in each community (subject to funding for HIV viral load testing).

Assuming that the mean of log10(viral load) is 4 in Arm C, that k=0.15 and the standard deviation of viral load within communities is 0.9 on the log10 scale, there is 84% power to show a reduction of 1 in log10 viral load in each of the other two trial arms. Alternatively, comparisons between arms can be made on the basis of what proportion of HIV-infected

individuals have undetectable viral load. Assuming these proportions are 20% in Arm C, 40% in Arm B, and 60% in Arm A, the study is well powered to show a difference between Arms A and B, and very well powered to show a difference between Arms A or B and Arm C.

At 12 and 36 months, viral load will be measured in 75 HIV-infected individuals in each community (subject to funding for HIV viral load testing). Assuming 20% with undetectable viral load in Arm C, 40% in Arm B, and 60% in Arm A, and k=0.20, there is 77% power to show a difference between Arms A and B and 97% power to show a difference between Arm B and Arm C.

(3) HSV-2 Incidence Over 36 Months

This will be measured in the *Population Cohort*. Assuming that baseline HSV-2 prevalence is approximately 70% and that by 24 months the loss to follow-up is 20% and by 36 months it is 25%, the estimate of intervention effect on HSV-2 incidence will be based on 1837 person-years per community. If HSV-2 incidence in Arm C is approximately 5 per 100 person-years, there is >90% power to detect an increase to 7.5 per 100 person-years or a reduction to 3.0 per 100 person-years if k = 0.15, and 80-90% power to detect such effects if k = 0.20.

(4) Retention in HIV Care, and Viral Load Suppression and Drug Resistance Among HIV-Infected Individuals Who Are Taking ART

These outcomes will be measured in HIV-positive participants in the *Population Cohort*.

(i) Retention in care at 12 months after registering for HIV care

This will be measured in HIV-positive participants who present for HIV care for the first time after the start of the intervention period, an estimated 198 per community in Arms A and B, and 99 per community in Arm C. First, assuming that retention in care at 12 months is 85% in Arm C, and that k=0.2, there is 85% power to show a reduction to 75%, and >95% power to show an increase to 95%, in each of Arms A and B. Second, assuming that retention in care is 90% in Arm C, there is 96% power to show a reduction to 80% in each of Arms A and B, and 79% power to show an increase to 95%. Third, assuming retention in Arm C is 80%, there is 71% power to show a reduction to 70% in each of Arms A and B, and 94% power to show an increase to 90%.

(ii) Viral load suppression, and drug resistance, measured among HIV-positive members of the *Population Cohort* at 24 months

Sample size calculations assume that, by the time of the 24-month follow-up in the *Population Cohort*, and among individuals who registered for HIV care for the first time after the start of the intervention period, 67% of patients will have started ART in Arm C, 50% in Arm B and80% in Arm A; and that 80% of patients will participate in the *Population Cohort* survey at 24 months. This gives sample sizes in each community of 141, 88 and 59 patients who start ART and will be available for viral load and drug resistance measurement in Arms A, B, and C respectively.

Assuming 10% are not virally suppressed in Arm C, there is 91% power to show an increase to 20% in Arm A and 63% power to show a reduction to 5%. The corresponding figures for a comparison with Arm B are 86% and 60%.

The percentage of patients with acquired drug resistance will be a subset of those who are not virally suppressed, but the range of scenarios considered above includes plausible values for this endpoint as well.

(5) Case-Control Studies

Three Case-Control studies will be conducted as follows:

(i) Uptake of CHiP home-based HIV testing during Round 1 of intervention in Arms A and B, with cases selected at random from individuals who did not accept testing, and controls selected at random from individuals who accepted testing;

(ii) Acceptance of immediate ART in Arm A, among individuals who were first diagnosed, or self-reported, as HIV-infected as part of CHiP home-based testing in Round 1 of intervention and who were not already on ART. Controls are selected from among individuals who started ART within 3 months of the CHiP household visit in Round 1, and cases from among individuals who did not;

(iii) Uptake of CHiP home-based HIV testing during Round 2 of intervention in Arms A and B, excluding individuals who were diagnosed as HIV-infected in Round 1, and also individuals who self-reported they were HIV-infected in Round 1 or Round 2.

Calculations assume 400 cases and 400 controls, for each of the three case-control studies, assuming an unmatched design. Cases are individuals who refuse HIV testing, re-testing for HIV, or immediate ART for studies (1)-(3) respectively; controls are individuals who accept testing, re-testing for HIV and immediate ART for studies (1)-(3) respectively.

Assuming that the percentage of controls exposed to a particular risk factor is 10%, 15%, or 20%, and that the odds ratio comparing exposed with unexposed individuals is 1.75, the corresponding study power to show an effect of the risk factor is 71%, 85%, and 91% respectively. With an odds ratio of 2, the corresponding figures for study power are 90%, 97%, and 99% respectively. When the proportion of controls exposed to a particular risk factor is 15% or more, the sample size is sufficient for stratified analyses, such as separate analyses by country or by gender. For example, with 200 cases and controls for women, and an odds ratio of 2, then if 15% and 20% of controls respectively are exposed to the risk factor, study power is 75% and 83% respectively.

Table 8- Case-control studies of (1) uptake of HIV testing (2) uptake of re-testing for HIV and (3) uptake of immediate ART among HIV-positive individuals

Percentage of controls with a risk factor	Odds ratio for refusing testing/re-testing/immediate ART, comparing individuals with a risk factor characteristic to those without	Power (%)
10%	1.75	71%
15%	1.75	85%
20%	1.75	91%
10%	2.0	90%
15%	2.0	97%
20%	2.0	99%

(6) Process Measures – Uptake of HIV Testing, ART and Male Circumcision

(a) CHiP Data – Arms A and B

(i) HIV testing uptake

With an average community adult population of 25000, and acceptance of home-based HIV testing in the range 50-80% in each round of testing, in each community the 95% confidence interval for testing uptake will be +/-1-2% of the point estimate.

(ii) Screening for ART eligibility, and uptake of ART, among HIV-infected individuals

If it is assumed that 80% of individuals accept CHiP home-based HIV testing in the first round of testing, that 15% are HIV-infected, and that 25% of HIV-infected individuals are already taking ART, approximately 2250 ART-naïve HIV-infected individuals will be identified through home-based testing in each community. With uptake of immediate ART in Arm A, and screening for ART eligibility in Arm B, in the range 30-80%, in each community the 95% confidence interval for screening/uptake will be +/-2% of the point estimate.

(iii) Male circumcision

With an average community adult male population of 12500, there will be approximately 10625 HIV-uninfected men in each community. In the 2010 ZAMSTAR TB/HIV prevalence survey, in the Western Cape trial communities 77% of men aged 18 or above reported that they were circumcised, and in the Zambian trial communities 13%. So if uptake of CHiP home-based HIV testing in the first round of testing is 80%, an average of 1955 HIV-uninfected men will be eligible for medical male circumcision in each Western Cape trial community, and 7395 in each Zambian trial community. If 50% of these men are circumcised during the first year of trial intervention, the 95% confidence

interval for the percentage who are circumcised will be +/-2% of the point estimate in Western Cape communities and +/-1% of the point estimate in Zambian communities.

(b) Arms A, B, and C – Population Cross-Sectional Survey at 24 Months

As noted above, funding has not been obtained to include a *Population Cross-Sectional Survey* as part of the study. If funded, the survey would provide additional useful data for all of the process measures described above for the *Population Cohort*. The analysis plan for this survey is described in Appendix VIII.

7.2 Random Assignment / Study Arm Assignment

Random assignment to study arms will take place at the cluster level. First, the 21 clusters will be matched into triplets based on best available estimates of HIV prevalence in the general adult population of these clusters, and taking into consideration geographic proximity of the sites to one another. This will be done separately in each country (stratified randomization), with 4 matched triplets in Zambia and 3 matched triplets in South Africa. The matched design will be used with the aim of minimizing the between-community variance in baseline HIV incidence, which is assumed to be correlated with baseline HIV prevalence.

After dividing the 21 clusters into 7 matched triplets, allocation to the three study arms will be carried out using a process of restricted randomization. This procedure will be used to ensure overall balance across study arms on cluster size, current ART uptake and HIV prevalence. There are $(3!)^7$ = 279,936 possible ways of allocating the clusters to the three study arms within matched triplets. These allocations will be evaluated against balance criteria to determine a restricted list of allocations that achieve adequate balance on the three variables defined above. The final allocation will be selected randomly from this restricted list of balanced allocations.

7.3 Statistical Analysis

The primary analysis will be based on a comparison of the incidence of HIV infection during the follow-up period of 3years between Arms A and C, Arms B and C, and Arms A and B. This will be carried out using appropriate analytical methods for cluster-randomized trials.

Because the number of clusters per arm is small, we will use methods based on Student's t-test, which have been shown to be highly robust for small numbers of clusters especially when sample sizes are similar in all clusters as in this study. We will compute the incidence of HIV infection in each cluster, weighted to take account of the sampling design which involves random selection of one adult from each household irrespective of household size. To test the null hypothesis of no impact, the paired t-test will be applied to these summary measures (7 matched pairs for each comparison), with 6df. The effectiveness of the intervention is defined as follows:

Protective effectiveness = 1 – RR

where the rate ratio RR is the ratio of incidence rates in the two study arms under comparison. This will be estimated by taking the geometric mean of the RR observed in each of the matched pairs, and a 95% confidence interval will be obtained using a normal approximation.

Evidence for intervention effect will also be assessed using a non-parametric permutation test, based on the list of all possible allocations of trial arms to communities that met the restricted randomization criteria. For each of these possible allocations, and including the allocation that was randomly selected, incidence rate ratios for intervention effect (comparing Arm A and Arm B, Arm B and Arm C, and Arm A and Arm C) will be calculated as above. The number of allocations (n) for which the incidence rate ratio is as extreme as or more extreme (further away from 1) than the value observed in the trial will be counted, and a 2-sided p-value calculated as n divided by the total number of possible allocations.

A description of how the analysis will control for migration contamination among the communities in the different treatment arms will be given in the statistical analysis plan along with methods used to analyze the secondary outcomes.

7.4 Interim Evaluation

As stated in Section 2.4, interim evaluation will take place during the first two years of intervention to determine whether to continue with the 36 month follow-up of the *Population Cohort* and the fourth year of intervention.

Because decisions on delivery of the intervention need to be taken at least 12 months in advance, to enable sufficient time for planning in the context of the annual PEPFAR funding cycle, the main evaluation will be conducted towards the end of 2014 when the second round of intervention and the 12 month follow-up of the *Population Cohort* should be complete or close to completion (Figure 2).

The main criteria for evaluation will be:

- Observed HIV incidence in the control arm during the first 12 months of follow-up
- Measures of uptake and coverage of the intervention during the first two rounds of intervention

The evaluation will be carried out by the DSMB and detailed criteria for the evaluation will be agreed with the DSMB before the start of the trial.

7.4.1 HIV Incidence

Sample size calculations for the trial were based on an assumed HIV incidence of between 1.0 and 1.5 per 100 person-years in both Zambia and South Africa. While this assumption is consistent with epidemiological data from the study populations, there remains uncertainty about the current and future level of HIV incidence in the 21 study

communities. The study may be under-powered if incidence is substantially below 1.0 per 100 person-years.

Data on estimated HIV incidence in the control arm (Arm C) based on the 12 month follow-up of the *Population Cohort* will be presented to the DSMB. These data will be prepared by a statistician independent of the study team so that they are not inadvertently unblinded to data on the effect size after 12 months. The DSMB will evaluate the implications of this incidence estimate on study power and will consider whether any change in the duration of the study would be appropriate.

Review of estimated effect size (by comparing HIV incidence between study arms) would be of limited value after 12 months of follow-up. If the effect is small, this would be consistent with a projected impact that increases steeply over time. If it is large, there would remain a need to measure the longer-term effects of the intervention including the occurrence of adverse effects.

7.4.2 Uptake of Intervention

Data on the uptake and coverage of the intervention components during the first two years of intervention delivery will be collated and presented to the DSMB. These uptake statistics will be used to populate the mathematical model (Section 7.5) in order to obtain estimates of the projected effect of the interventions in Arms A and B relative to Arm C by time since the start of intervention roll-out. These projected estimates together with the HIV incidence estimates from the control arm will be used to obtain power estimates to guide a recommendation on the duration of follow-up.

The main purpose of the interim evaluation is to assess indicators of futility, suggesting that the trial is unlikely to achieve its aims even if intervention and follow-up are continued. Conversely, if the evaluation suggests a substantial effect of intervention, follow-up for at least three years is likely to be needed to adequately evaluate potential adverse effects of the intervention.

7.5 Mathematical Modeling

A more sophisticated individual-based stochastic model of HIV transmission will be developed during the project and fitted to data from the trial, routine data and published sources to address four main objectives:

- To help interpret the results of the trial: Process data showing the extent of uptake of the intervention compared with similar data from the control arm will be used to obtain model projections of expected impact under these conditions. By examining projected impact under the conditions prevailing in Zambia and South Africa, and in different trial communities, we will be able to examine whether the level of impact and variations in impact are in accordance with expectations.

- To project longer-term impact: Modeling shows that the full impacts of UTT as well as male circumcision are not seen for several years. Impact measured during 3 years of intervention may therefore underestimate the long-term impact of the program. Models

fitted to the impact seen during the first 3 years will be used to project the likely impact over longer time periods.

- To explore likely impact in different settings: If the trial demonstrates impact, it is likely that similar interventions will be implemented in a wide range of settings. The model will be used to explore how impact would be expected to vary depending on epidemiological, demographic and other characteristics of populations, and thus to project likely impact in a range of settings.

- To explore the likely impact of alternative intervention packages: Our study design will provide empirical data on the impact of the specific packages of preventive interventions incorporated in the PopART program. However, the model can be used to explore the effect of adding or removing components. For example, we can project the impact of an intervention in which male circumcision is not promoted, or where the threshold for starting ART is set at different levels.

Like all HIV models, the model will be over-parameterized compared to the amount of data available. The model will thus be fitted to baseline and follow-up data using Bayesian Monte Carlo integration methods. Priors for parameters will be determined by literature review, and a body of informed persons not including the modelers working on this study will pick the prior distributions of parameters so as to avoid bias. Comparison of priors with posteriors will be used to inform the extent to which the trial has improved our estimates of the likely efficacy of the different components of the intervention, and of other epidemiologically relevant parameters.

We acknowledge the importance of the *prevention cascade* in achieving population-level impact. Specifically, achieving high levels of uptake and effectiveness requires guiding individuals through a cascade of individual steps, starting from an initial test, through linkage to care, CD4 testing, circumcision, counseling, and ultimately antiretroviral treatment and adherence counseling for HIV-infected and eligible individuals. The contribution of different levels in the cascade, as well as their contribution to intervention cost, will be explored in our modeling and cost-effectiveness work. Our prior hypothesis based on preliminary modeling is that uptake of testing and prompt initiation of treatment will be critical.

7.6 Outcomes for Secondary Objectives

Multiple secondary objectives for this study are listed in Section 2.2. The majority of these objectives are to measure the effect of the intervention on various outcomes using standard quantitative analyses; the outcome measures for these secondary objectives are listed below and will be measured in all study arms unless otherwise noted. Those secondary objectives that are considered process measures, or that require a different sort of analysis, are described in separate sections below.

- HIV incidence over the first, second, and third years of follow-up
    - HIV diagnosis at 12 months, 24 months, and 36 months among those who were HIV-uninfected at enrollment in the *Population Cohort*

- Community viral load (if funding is available)
    - Viral load in HIV-infected members of the *Population Cohort* (75 per cluster, randomly-selected) at enrollment, 12 months, and 36 months
    - Viral load in HIV-infected members of the *Population Cohort* (all, estimated to be 300 per cluster) at 24 months
- ART adherence and viral suppression
    - HIV viral load at 24 months in HIV-infected members of the *Population Cohort* who initiated HIV care and ART after commencement of the PopART intervention in the community (if funding available)
    - HIV viral loads of health center attendees who initiated HIV care and ART after commencement of the PopART intervention in the community, drawn from routinely-collected data at health centers (if available at a given health center)
    - Self-reported adherence to ART in HIV infected members of the *Population Cohort* who initiated HIV care and ART after commencement of the PopART intervention in the community, measured at 12 months, 24 months, and 36 months
    - Loss-to-follow-up rates and missed dispensations of ARVs among health cent*er attendees who* initiated HIV care and ART after commencement of the PopART intervention in the community (and also in the control community during the same period of time), measured using routine health center data
- ART drug resistance (if funding is available)
    - ART resistance at 24 months in HIV-infected members of the *Population Cohort* who initiated HIV care and ART after commencement of the PopART intervention in the community, among individuals who are not virally suppressed at 24 months (if funding available)
    - ART resistance in HIV-infected members of the *Population Cohort* who initiated HIV care and ART after commencement of the PopART intervention in the community, measured retrospectively on samples collected at enrollment and 12 months, among individuals who are not virally suppressed at 24 months (if funding available)
    - ART resistance, measured at 12 months, 24 months, and 36 months, among participants with incident HIV infection after enrollment in the *Population Cohort* (if funding available)

NB - Viral load/drug resistance testing will be performed at the 24 month visit, as a measure of treatment adherence, among HIV infected members of the *Population Cohort*, rather than delaying to 36 months. If the 24 month data on this indicates a significant number of participants not virally suppressed/with drug resistance, then additional funding may be sought to analyse these data again at 36 months in the Population Cohort and/or the Population Cross Sectional Survey.

- HSV-2 incidence
    - Incident HSV-2 infections at 12 months, 24 months, and 36 months for all individuals in the *Population Cohort* who were HSV-2-uninfected at enrollment
- HIV disease progression, retention in care, and death
    - CD4 counts, WHO staging events, retention in care and death among *Population Cohort* participants initiating ART after commencement of the PopART intervention in the community, measured using routine health center data
    - CD4 counts, WHO staging events, retention in care and death among health center attendees who initiated ART after commencement of the PopART intervention in the community, measured using routine health center data
- ART toxicity
    - ART safety and clinical events among *Population Cohort* participants initiating ART after commencement of the PopART intervention in the community, measured using routine health center data
    - ART safety and clinical events among health center attendees who initiated ART after commencement of the PopART intervention in the community, measured using routine health center data
- Sexual risk behavior
    - Self-reported sexual risk behavior at Enrollment, 12 months, 24 months, and 36 months in the *Population Cohort*
    - HSV-2 incidence, listed above as a separate secondary outcome, serving as a biomarker for sexual risk behavior
- Case notification rate of tuberculosis
    - Case notification rates of bacteriologically-confirmed TB diagnosed among the general population of patients seeking care at health centers as recorded by health centers
    - TB mortality among TB cases in the community as recorded by health centers
- HIV-related stigma
    - Self-reported data on stigma indicators at enrollment, 12 months, 24 months, and 36 months in the *Population Cohort*
    - Qualitative interviews in selected members of the general population in Arms A, B, and C

7.7 Secondary Objectives for Case-Control Studies

- Carry out case-control studies to examine factors related to:

- Uptake of HIV testing during the first round of home-based testing in Arms A and B

- Uptake of immediate treatment in Arm A

- Uptake of HIV testing during the second round of home-based testing in Arms A and B

7.8 Secondary Objectives for Qualitative Studies

- Use qualitative and quantitative methods to:

  - Assess popular understanding of HIV testing and treatment at study initiation and during implementation

  - Evaluate the acceptability and functioning of the CHiPs in Arms A & B

  - Evaluate the acceptability of interventions and the barriers to access in Arms A & B

  - Document the effect of the intervention on social networks, stigma, sexual behavior, alcohol use, gender-based violence, HIV identity, other HIV prevention options and community morale.

  - Evaluate the process and challenges of community consultation and applying ethical principles.

7.9 Secondary Objectives Related to Economic Evaluation

Three secondary objectives of this study are concerned with economic evaluation of the intervention:

- Measure the incremental cost of the two intervention packages through systematic recording of costs in intervention and control communities.

- Estimate the effectiveness and cost-effectiveness of the intervention packages and alternative packages, both in the chosen study populations and in other populations by fitting mathematical models based on the empirical data from the trial, including data related to cost.

- Measure the burden experienced by local health centers due to implementation of the intervention in the community

Economic analysis will seek to assess the incremental health benefits of the intervention in relation to its incremental cost, and will be integrated with the modeling described above in Section 7.5. The main focus will be on costs to the health services, including equipment, materials and personnel. Incremental cost of the intervention will be estimated by comparing health services utilization and associated costs between the three study arms. We will be careful to separate out the costs of the intervention and the costs of the evaluation.

Benefits will be assessed in terms of lifetime change in quality adjusted life years (QALYs) and/or disability adjusted life years (DALYs) brought about by the interventions relative to the control arm. Health related quality of life will be measured with a generic instrument such as SF36 or SF12. Lifetime projections of health and health services utilization will be modeled under a range of assumptions based on current epidemiological and health service evidence. Probabilistic sensitivity analysis will be used to assess uncertainty.

By combining cost data with impact estimates from the trial, we will be able to obtain direct estimates of cost per HIV infection averted. The costing data will be integrated with modeling results and quality of life information to derive cost per QALY and/or DALY using different time horizons. These data will also be used in mathematical models to explore the likely cost-effectiveness of the same intervention in different settings, and of alternative intervention packages in these or other settings.

The 3-arm trial design will allow us to compare the short and long term differences in cost-effectiveness for the two combination prevention packages based on immediate treatment or treatment according to current national guidelines. Additional data on secondary outcomes including TB incidence and other clinical events will provide improved QALY and/or DALY estimates.

The economic evaluation will mainly rely on data from the Population Cohort via a questionnaire, supplemented by health service facilities data. More specifically, we will collect data that will allow us to calculate the following benefits and costs.

We will estimate the benefits of the interventions to individuals, measured by the impact on QALYs/DALYs, and impact on work and home duty productivity (caring for children and seniors), approximated by employment status, occupation and educational status. Another benefit of the interventions is a reduced rate of HIV-related illnesses, with positive health impacts to the individual, and saved health care costs to society.

We will estimate the wider benefits of the interventions to the community. This is mainly the prevention effect of the interventions, in the form of averted secondary HIV infections. We place an economic value on those averted infections. This comprises a) the health benefit (saved QALYs/DALYs) of averted HIV and related illnesses, and b) the averted health care costs (assuming standard care) for the study communities. Further, we consider the indirect benefit to children (both infected and not infected) of mothers receiving the interventions, measured in probability of survival (we do not collect quality of life information for children).

We will generate estimates of the costs to the health care system of providing the interventions, such as clinical assessment, testing and drug provision for ART. This includes costs for treating drug resistance, toxicities, side effects and adverse events. We will consider one-time costs, for example for building and training, and apportion them to the interventions. We will also consider recurrent costs for personnel (wages and related costs, e.g. pensions), the ART drugs, other drugs, laboratory tests, materials, equipment and supplies, transport costs for staff or patients (if covered by the health care system),

and overheads. We will collect aggregate data from health service facilities involved in the trial, and apportion these to individuals based on health service usage information. If we can obtain reliable data from patient records held at health care facilities, through some members of the *Population Cohort* and/or a small scale additional survey of a random sample of patients as they visit health care facilities in selected communities, we will calculate actual costs of treatments; otherwise, we will calculate average costs for typical use of health care facilities. This process requires assumptions, and we will conduct sensitivity analysis to validate our estimates. Further, as the cost estimates rely on health service utilization information collected via questionnaire retrospectively for the past period, we will conduct validation to address potential recall bias.

The program may divert scarce resources from other health programs, and in order to evaluate this indirect effect, we will obtain information via qualitative interviews with senior health care management in selected communities. We will further collect information on the costs to the patients of receiving the interventions, including the costs of adverse events and drug resistance. Costs to individuals may comprise costs directly associated with treatment (frequency, duration and nature of contacts with health services, travel time), user fees, costs of tests, drug costs and other payments related to treatment, time costs (valued by lost earning opportunity) and other costs. Information on those items will be collected from the survey members.

We plan to complement the economic evaluation with additional work looking at broader outcomes, such as the impact on children of improved parental survival, and the impact of improved health status on individual productivity, participation in the labor market, poverty and wider macro-economic effects on the economy. Estimates of the benefits of improved survival and health from other studies can be integrated with the modeling work to obtain estimates of broader societal gains. If the trial shows that interventions are effective, it will also be important to model the projected cost-effectiveness of wider-scale intervention using more streamlined systems of delivery.

Outcomes for secondary objectives related to the economic analysis are summarized in Table 9.

Table 9- Summary, outcomes for secondary objectives related to the economic analysis

Secondary outcomes	Data sources	Subsample	Specific measures	Assumptions	Secondary data sources required?	When collected?
**Health care utilization and costs**	Population Cohort	All	Frequency, types and reason of visits		Yes, lifetime costs of HIV care	Each visit
	Routine patient records	HIV+ and MC patients	Diagnoses and types of treatments	Aggregation into groups with homogenous resource use	Yes, lifetime costs of HIV care from other studies	Throughout trial
	Routine facility data	Selected facilities	Health care utilization aggregated	Facilities are representative	Yes, aggregate data from Dept. of Health	One time, rolling
	Survey of facility costs	Selected facilities	One time and recurrent costs	Facilities are representative;	Yes, aggregate data from Dept. of Health	One time, rolling
**Costs of accessing care to individuals**	Population Cohort	HIV+ and MC patients	User fees, travel costs, time costs	Aggregation into groups of typical travel costs	Yes, private insurance coverage, travel costs	Each visit
**Quality of life**	Population Cohort	All	Generic measure of health	Standard assumptions of generic quality of life instruments	Yes, preference weights	Each visit
**Work and home productivity**	Population Cohort	All	Data on employment occupation	Association between occupation and wage rate	Yes, wage rates for occupations	Each visit

**Burden on care-givers**	Population Cohort	Care-givers of HIV+ and orphans	Data on caring activities	Alternative occupation	Yes, burden of care, wage rates	Each visit
**Child survival**	Population Cohort	All	Mortality information		Yes, official death records	Each visit
	Routine patient records	All	Mortality information		Yes, official death records	Throughout trial

7.10 Process Measures

Several process measures will be recorded in Arms A, B, and C to evaluate the implementation and delivery of the PopART interventions. These measures evaluate processes that are intermediary between the provision of the intervention and achievement of the primary outcome. These measures will be important therefore in understanding how and why the intervention is (or is not) successful in producing that outcome. Further, those data that are collected from CHiP teams or health centers (as opposed to research cohorts) can be reviewed by the study team during the study period and can be used to make real-time adjustments to deployment of the intervention to improve its effectiveness.

- Uptake of PMTCT
  - Self-reported use of services for PMTCT at Enrollment, 12 months, 24 months, and 36 months among HIV-infected women in the *Population Cohort* who had been pregnant in the prior 12 months
  - Uptake of PMTCT services at health centers
  - Uptake of PMTCT as indicated in data collected in households by CHiPs
- Uptake of male circumcision
  - Self-reported circumcision status/uptake at Enrollment, 12 months, 24 months, and 36 months of men in the *Population Cohort*
  - Uptake of circumcision in the community as indicated in health center data
  - Uptake of circumcision as indicated in data collected in households by CHiPs
- ART screening and uptake
  - The proportion of *Population Cohort* members, identified as HIV-infected who screen for ART eligibility, and who subsequently initiate ART
  - Proportion of community members, identified as HIV-infected in data from CHiP teams, who screen for ART eligibility, and who subsequently initiate ART, as indicated in health center data
- HIV testing and retesting
  - Self-reported recent HIV testing at Enrollment, 12 months, 24 months, and 36 months in the *Population Cohort*
  - The number of adults (16 years and older) in the household and the number of HIV tests performed as indicated in data from CHiP teams and health centers
- Time between HIV diagnosis and initiation of care
  - The proportion of *Population Cohort* members initiating HIV care within 3 months of a positive HIV diagnosis
  - The proportion of community members initiating HIV care within 3 months of HIV diagnosis as indicated in data from CHiP teams (provision of HIV positive result) and health center data (date of care initiation)

7.11 Tabular Summary of Outcomes

Table 10 provides a summary of HPTN 071 objectives and outcomes including the source of the outcome data.

Table 10- Summary of Study Objectives and Related Outcomes

PC= *Population Cohort* **PX**= *Population Cross-Sectional Survey* **Ca-Co**= Case-control **CHiPs**= Community HIV-care Providers

**Objectives** and *Outcome Measures*	**Research Participants**							**Community members**	
	**PC at baseline**	**PC at 12m**	**PC at 24m**	**PC at 36m**	**PX at 36m**[a]	**Uptake Ca-Co**	**Qualitative Research**	CHiPs Data (Arm A&B)	Routine Health Center Data
Effect of the interventions on…									
**HIV incidence**									
HIV infection between 0 and 36 months among those testing HIV negative at enrollment (primary objective)	X	X	X	X					
HIV infection between 0 and 12 months in those testing HIV negative at enrollment; HIV infection between 12 and 24 months in those testing HIV negative at 12 months; HIV infection between 24 and 36 months in those testing HIV negative at 24 months (secondary objective)	X	X	X	X					
**Community viral load** [a]									
Viral load in a subset of 75 HIV-infected cohort/survey members per community	X	X		X	X				
Viral load in all HIV-infected cohort members			X						
**ART adherence and viral suppression**									
HIV viral load among cohort/survey members initiating ART after intervention roll-out [a]			X						
HIV viral loads among health center attendees initiating ART after intervention roll-out (if viral load available)									X
Self-reported ART adherence among cohort/survey members initiating ART after intervention roll-out		X	X	X	X				
Loss-to-follow-up rates and missed dispensations of ARVs among health center attendees on ART									X
**ART drug resistance** [a]									
ART resistance at 24 months among cohort/survey members initiating ART after intervention roll-out, who have detectable viral load at 24 months			X						
ART resistance testing may be performed on samples from enrollment, 12 month, and 24 month visits among cohort members initiating ART after intervention roll-out, who have detectable viral load at 36 month (pending funding)	X	X	X	X					
ART resistance at 12 months, 24 months, and 36 months among cohort members who acquire HIV infection during follow-up period		X	X	X					
**HSV-2 incidence**									

Incident HSV-2 among cohort members testing HSV-2 negative at enrollment		X	X	X					
**HIV disease progression, retention in care, and death**									
CD4 counts, WHO staging events, retention in care and death among Population Cohort participants initiating ART after commencement of the PopART intervention in the community, measured using routine health center data [b]									X
CD4 counts, WHO staging events, retention in care and death among health center attendees who initiated ART after commencement of the PopART intervention in the community, measured using routine health center data									X
**ART toxicity**									
ART safety and clinical events among cohort/survey members [b]	X	X	X	X	X				
ART safety and clinical events among health center attendees (based on clinic data)									X
**Sexual risk behavior**									
HSV-2 incidence (independent secondary objective above) serving as a biomarker for sexual risk behavior		X	X	X					
Self-reported sexual risk behavior	X	X	X	X	X	X[c]	X[c]		
**Case notification rate of tuberculosis**									
Case notification rates of bacteriologically-confirmed TB diagnosed among health center attendees as recorded by health centers									X
TB mortality among TB cases in the community as recorded by health centers									X
**HIV-related stigma**									
Self-reported data on stigma indicators collected from cohort members	X	X	X	X	X				
Qualitative interviews conducted with members of general population							X		
Process Measures									
**Uptake of PMTCT services**									
Self-reported use of PMTCT services at among HIV-infected cohort/survey members who were pregnant in the prior 12 months	X	X	X	X	X				
Uptake of PMTCT services at health centers									X
Self-reported uptake of PMTCT in the community								X	
**Uptake of male circumcision**									
Self-reported circumcision status/uptake among cohort/survey participants	X	X	X	X	X				
Uptake of circumcision at health centers									X
Self-reported uptake of circumcision in the community								X	
**ART Screening and uptake**									
The proportion of cohort/survey members identified as HIV-infected who screen for ART eligibility, and who subsequently initiate ART	X	X	X	X	X			X	X

The proportion of community members identified as HIV-infected who screen for ART eligibility, and who subsequently initiate ART								X	X
**Uptake of HIV testing and retesting**									
Self-reported recent HIV testing among cohort/survey	X	X	X	X	X				
Number of adults in households and the number of HIV tests performed in each community								X	X
**Time between HIV diagnosis and initiation of care**									
The proportion of cohort members initiating care within 3 months of HIV diagnosis[b]	X	X	X	X	X			X	X
The proportion of community members initiating HIV care within 3 months of HIV diagnosis								X	X
Using qualitative methods…									
**Assess popular understanding of HIV testing and treatment**							X		
**Evaluate the acceptability and functioning of the CHiPs in Arms A & B**							X		
**Evaluate the acceptability of interventions and barriers to access in Arms A & B**							X		
**Document effect of the interventions on social networks, stigma, sexual behavior, alcohol use, gender-based violence, HIV identity, other HIV prevention options & community morale**							X		
**Evaluate the process and challenges of community consultation and applying ethical principles**							X		
Through mathematical modeling and health economics methods…									
**Measure the incremental cost of the two intervention packages through systematic recording of costs in intervention and control communities** [d]			X						X
**Estimate the effectiveness and cost-effectiveness of the intervention packages and alternative packages** [d]			X						X
**Measure the burden experienced by local health centers due to implementation of the intervention in the community** [d]			X						X

[a] Not currently funded. Will be implemented if funding can be acquired

[b] Consent must be obtained to access health center records for *Population Cohort* members

[c] Explanatory research related to the outcomes/objectives indicated

[d] Cost and effectiveness objectives will be addressed through analysis of deidentified data from health centers regarding costs and clinic use, in addition to specific questions asked of the *Population Cohort*

8.0 HUMAN SUBJECTS CONSIDERATIONS

8.1 Collaborative Partnerships

At all stages of the development of this research protocol community representatives have been involved in the design and have engaged with the research team to finalize the intervention and study questions. The research teams will continue to actively engage with the study communities at various levels (for example with government structures, health care facilities at management and worker level, existing community forums and stakeholder groups) utilizing a range of communication and interaction strategies, as appropriate. In both countries, study committees will be formed with representation from trial staff and in-country stakeholders (from governmental to community representation) to provide guidance and feedback to the study team. As indicated in the earlier section on community engagement, partnerships and CABs worked with/established during the ZAMSTAR study will be reviewed for this study to ensure that all community groups and interests are represented.

8.2 Social Value

As described in Section 1, the worldwide burden of HIV infection continues to grow, with populations in sub-Saharan Africa particularly afflicted with high rates of HIV prevalence and incidence. If this study is able to show that providing a combination prevention intervention including universal testing and treatment is effective in slowing the spread of HIV in communities and is cost-effective, it could provide a path forward to lowering the burden of HIV in sub-Saharan Africa and throughout the world and, importantly, in the communities and countries in which the study will be performed. The value of a highly effective prevention intervention for the economies of countries, communities and persons, the reduction in morbidity and mortality, the value to health infrastructure and even political stability could potentially be large. The multifaceted approach of the intervention, incorporating strengthening and promotion of PMTCT, male circumcision, and universal testing and treatment, offers value to community members whether they are men or women, already infected with HIV or uninfected. The health systems strengthening component of the study, which will be implemented in all three study arms, promises to offer value to all of the communities in the study, even if the intervention cannot be shown to lower HIV incidence.

8.3 Scientific Validity

This study will provide evidence to either support or refute the mathematical model discussed earlier, which has indicated that if a high proportion of the population can be tested, with those found HIV-infected offered immediate ART, HIV infection may be reduced substantially within two years, and potentially eliminated as a public health problem in the longer term. The study has been powered to determine the impact of the interventions on the primary and secondary endpoints. The multi-community cluster-randomized study design chosen for this study has, we believe, the best chance of

providing an answer to the research question: "Can universal HIV testing and intensified provision of HIV treatment and care reduce population-level HIV incidence?"

The study will be conducted according to the most rigorous standards of research and is therefore expected to give definitive answers about the process of implementing the intervention as well as the impact of such an intervention at community level. The study results will be shared throughout the study with national and international policy-makers to ensure that the findings are understood and that lessons from the study are implemented.

8.4 Fair Subject Selection

This study will be carried out in areas of Zambia and South Africa that are known to have a high prevalence and incidence of HIV infection. These areas are continuing to experience severe generalized HIV epidemics with prevalence levels of 15-20% in many areas. Most of the communities chosen for this project are communities that have already been involved in similar community-based research projects such as ZAMSTAR (Zambia and South African TB and AIDS Reduction Study). There are both advantages and disadvantages to involving ‘research experienced’ communities in a new research project such as this. Communities can theoretically become over researched and placed at risk for “research burn-out”- with community members reluctant to become involved in additional research and becoming disillusioned with research related burdens (e.g. time spent, intervention risks, risks to privacy etc.). On the other hand communities who are already accustomed to and well informed about research processes can be considered to be in a more empowered position to engage in a research initiative such as HPTN 071, than "research naïve" communities. Community leaders will be accustomed to engaging with research teams and structures such as community advisory boards (CABs) will already be established and functioning.

The research teams in both Zambia and SA have discussed this issue with the respective government authorities and a joint decision was taken favoring existing research sites and communities over new ones for this very reason. Formative research will be conducted prior to the start of the study to document existing community engagement structures in each community and their level of functioning. Additional action will be taken to improve the functioning of these structures, where necessary.

Care will be taken to ensure that community related research risks and burdens are minimized and that community benefits are maximized (See Section 8.5).

All population members in the intervention communities will be encouraged to participate in the intervention. This will include women and sexually active minors over the age of 16 in Zambia and 12 in South Africa, hard-to-reach populations and “high HIV-risk groups” because this study is directly relevant to the health needs of these groups.

8.5 Risk-Benefit Assessment

This community-based, cluster-randomized study can potentially incur risk of harm at both a community and an individual level. Likewise study-related benefits may accrue at both an individual and a community level.

8.5.1 Community Level

8.5.1.1 Benefits

At the community level, mathematical modeling suggests that the PopART intervention may result in a substantial reduction in HIV incidence and, if sustained over time, to the eventual elimination of HIV as a public health problem, with a wide range of health and socio-economic benefits. Child morbidity and mortality should also be significantly decreased through both the direct effects of the intervention on mother-to-child transmission of HIV and the protection of the health of HIV-infected mothers. In addition to intervention effects on HIV transmission, the universal testing and counseling program is designed to promote acknowledgement and acceptance of HIV as a community-wide health problem potentially resulting in lessening of HIV-related stigma and discrimination. While the control communities will not benefit directly from the intervention, the project will ensure that a standard of care is provided in these communities. In addition, if the trial shows that the intervention is highly effective and cost-effective, leading to wider scale roll-out of the program, efforts will be made to ensure that the control communities are among the first to benefit from this wider implementation.

Networks of stakeholders that will be created through the implementation of the study interventions will not only improve communication between community groups but will also be a catalyst for reinvigorating social connections that have been threatened in the wake of poverty and HIV/AIDS. Previous experience with the ZAMSTAR study suggests that these networks can be useful for advocating for research and improving research literacy.

8.5.1.2 Risks

Any community-based research project may present risks to a community. Communities may feel disempowered by having a research agenda imposed on them or they may be placed at risk of stigmatization by the publication or dissemination of research results. Large community research projects may disrupt intra-community social structures and networks that are not always easily understood by an external research team.
For this particular project at community level, there is the risk of behavioral disinhibition if the wide-scale provision of testing, treatment and male circumcision are assumed to reduce risk and thus encourage unsafe sexual behavior. Extensive counseling at an individual level and HIV prevention education at a broader community level will attempt to minimize this risk, which unfortunately is known to be potentially present with most HIV prevention studies. We will also seek to measure such harms through collection of process data as well as specific sub-studies as outlined in the Research Plan.

An additional potential community related risk involves the possible burden that could be placed on existing health services. Existing health services are already over-burdened due to inadequate resources and overwhelming disease rates. Health-care workers may be recruited into study teams and leave their current positions, worsening the problem.

8.5.1.3 Minimizing Risks to Communities

Communities will not be named in any publication or dissemination of results of the study. Well-functioning community engagement structures (such as CABs) will help to mitigate risks at community level by advising the study team and representing the views of the communities. In addition the research team will actively solicit and report any instances of perceived social harm. We will aim to recruit CHiPs that are residents of their respective study communities and many of the field study staff will have either previously lived or worked in the communities which will give them an advantage in terms of relating with participants and other stakeholders.

Health service burden will be minimized by leveraging additional funding to the Ministry/Department of Health to enable additional staff to be trained and recruited. These staff will work for the duration of the study and it is likely they will continue on as Ministry/Department of Health employees at the end of the study due to natural attrition and increased demand (many services are currently understaffed in respect to the stated staffing establishment). Study teams will work closely with government agencies and will not entice staff away from them by offering differential salary packages.

Communication will be maintained with study communities for the duration of the study and a well-developed exit strategy will be planned with the input of all stakeholders and community engagement structures to ensure that there is a seamless transition from study to routine health services at the end of the study

8.5.1.4 Risk-Benefit Assessment at Community Level

The mathematical models suggest, and we believe, that overall benefits from the proposed intervention program would greatly outweigh any risks or harms at community level. Nevertheless, we acknowledge that some communities and individuals may be placed at increased risk as a result of the intervention. It is therefore important to put appropriate measures in place, as described above, to mitigate these possible harms.

8.5.2 Individual Level

8.5.2.1 Benefits

There is a wide range of benefits at individual level. Knowledge of personal HIV status provides a portal to treatment and care services for HIV-infected patients while HIV-uninfected individuals can be supported in adopting preventive measures. While definitive studies are awaited, early treatment of HIV-infected patients is expected to be of clinical benefit, and the treatment provided (including antibiotic prophylactic therapy) can be confidently expected to reduce the incidence of TB and other infectious diseases as well as protecting the immune system and significantly slowing HIV clinical progression [90, 104-106]. It will also significantly reduce the risk of onward transmission to sexual partners, with a consequent decrease in the anxiety and psychological distress associated with HIV infection. WHO has also recently endorsed this approach in a programmatic update[107]

8.5.2.2 Risks

A key component of the intervention package is an annual HIV testing campaign that aims to encourage all adult community members to undergo HIV testing and counseling. Such approaches involve some risks. These include possible social harm, stigmatization or intimate-partner violence related to intended or unintended disclosure of HIV status, either within or beyond the household; and psychological trauma from learning one's HIV test status.

Community members who report to the health center to undertake HIV care (including ART) or to receive HIV testing will have sensitive data collected in clinic records, some of which will be then be harvested into an electronic research database. Most of these research data will be collected without personal identifiers, but research cohort members may provide consent for use of identified data. Collection and storage of sensitive health information carries with it the risk of unwanted disclosure if there is a breach of data security or incomplete removal of personal identifiers from “anonymous” data sets.

Men who test negative for HIV infection will be offered circumcision through a locally provided service. Circumcision will also be provided to HIV-infected men who request it. While data from randomized trials and routine male circumcision programs in sub-Saharan Africa have shown very low levels of adverse effects, there are some risks of the surgical procedure, including pain, bleeding and infection [49, 108, 109]. There is also a risk of enhanced HIV transmission if men resume sexual activity before the circumcision wound is fully healed [110, 111].

The offer of immediate ART goes beyond current national and international guidelines for HIV care, although treatment practices in the U.S. and some other developed countries are in practice approaching immediate treatment in many cases. Immediate initiation of ART presents some potential risks. These include development of drug resistance if treatment adherence is sub-optimal and consequent limitation of future treatment options, and the inconvenience of having to attend the study clinic and starting on a lifelong course of treatment when still asymptomatic.

There are additional minor risks for the research cohort participants including the taking of specimens, which may include pain or bruising when blood samples are taken. Also, there is the risk that some questions addressed to participants, for example relating to their sexual behavior or HIV infection status may result in discomfort or distress.

The main risks associated with the evaluation research for those individuals who are not directly exposed to the ART intervention are similar to risks relevant to any population-based epidemiological HIV research and primarily involve risks to privacy and confidentiality. These risks are discussed below as well as in Section 8.8.

8.5.2.3 Minimizing Risks to Individuals

To minimize social and other harms relating to the intensive testing program, staff delivering the program will be carefully trained and supervised to ensure that they have the required skills to provide individual or couple counseling according to national and international guidelines. In particular, although couples will be encouraged to undergo

testing and counseling together, individual testing will be provided for those not wishing to take this up. Participants will be given information about community-based organizations providing support and guidance for those dealing with the psychological consequences of HIV infection or suffering from domestic violence. Follow-up counseling will be offered by the community counseling teams as is required by individuals or households according to their wishes.

Data systems and data handling procedures for capturing, transferring, analyzing and storing electronic data obtained from health centers will be developed and tested to verify their ability to preserve participant confidentiality. Electronic systems in which these data are kept will be password protected with access limited to authorized staff. Electronically kept personal identifiers will be stored in separate datasets with password protection only accessible for designated staff (for computers and servers).

Phylogenetic studies may be carried out on stored samples to evaluate the linkage between HIV infections in the community, subject to funding. Any such analyses will only be carried out after removing linkage to personal identifiers. We will ensure that data provided to the team performing any such linkage analyses, and any reports of such analyses, cannot inadvertently identify specific individuals or transmission events.

Male circumcision will be provided by existing service providers through the routine health service and therefore all staff will be carefully trained in line with national guidelines to ensure that the operation is carried out safely, with minimal risk of adverse events. Patients will be seen for a follow-up visit after circumcision according to national guidelines. Staff will be trained to deliver effective counseling about the importance of abstaining from sexual activity until the wound is fully healed and also to explain carefully that the operation is only partially protective against HIV infection and to warn against the hazard of risk disinhibition.

As noted above, the only component of the intervention that goes beyond current guidelines is the offer of immediate ART regardless of CD4 count in Arm A. The team plans to inform all patients seeking ART at health centers in Arm A of the differences between standard treatment guidelines and the UTT strategy being tested in the PopART intervention Arm A in their community, including known risks and benefits. Individuals must provide written informed consent for initiation of ART if not eligible according to current national guidelines. Information provided to patients will have been developed partly in consultation with CABs and have been piloted and translated into vernacular text. The treatment regimen has been chosen carefully to be convenient to take, to be appropriate for the widest possible range of patients, and to minimize the risk of toxicity or side effects. No additional adverse events are expected in patients with intact immune systems. The most significant risks are associated with poor adherence to treatment. To minimize this, community health workers will support patients on treatment, making regular household visits to check on treatment adherence and in particular checking up on patients when they do not attend routine clinic visits. Toxicity associated with antibiotic prophylaxis will also be monitored and treatment modified if necessary.

All project staff will undergo training in GCP and human research protections in accordance with the U.S. National Institutes of Health (NIH) requirements. There will be a

strong emphasis in staff training and supervision on the importance of strict confidentiality of participant information as well as on supportive interviewing skills. Blood collection will be carried out by fully trained staff using appropriate sterile procedures.

8.5.2.4 Risk- Benefit Assessment at Individual level

There are risks associated with this study for the individuals involved. However as indicated above most of these risks are no more than those encountered in everyday life. Individuals exposed to the early ART intervention are likely to encounter a greater than minimal level of risk and some of these risks or burdens may as yet be poorly quantified (e.g. risks associated with extended ARV exposure). However providing ART earlier rather than later is an approach increasingly used in first world clinical settings and recent studies have shown more benefit than risk to this approach[112]. All patients receiving ART at the health centers in all study arms will be monitored for reactions to their ART regimen, in accordance with the local standard of care. Thus we believe that the overall risk-benefit assessment for this study is favorable at both a community and an individual level.

8.6 Informed Consent

In a community-based, cluster-randomized trial such as this one, informed consent needs to take place at several levels ranging from **consent from the government authorities**, to so-called "community consent", and finally to individual consent. However obtaining individual consent from every individual living in every community involved in this study would be unfeasible. As discussed earlier in Section 3.2 the CHiP teams, while an integral part of this research will deliver a community health care package that is recognized as good practice and as such is not a research intervention. Much of the **routine healthcare surveillance data** collected as part of this study, particularly from the control communities will be made available to the research team by the respective public health authorities (who are in full support of the project) and collected without specific individual informed consent. This information will be collected, coded, stored and managed in such a way as to ensure individual identity and privacy are protected at all times.

8.6.1 Approval from Respective Authorities

Approval for this project has been obtained from the respective healthcare authorities in both South Africa and Zambia. Additionally during the planning process of the study approval will be sought from other authorities such as district or local councilors, political leaders and traditional leaders.

8.6.2 ‘Community’ Consent

It is of the nature of a cluster-randomized trial of this kind that entire communities are assigned to one study arm or the other, and individual consent for community allocation is not possible. The term ‘community consent’ can be misleading. True ‘Community Consent’ is only possible if the “community has a legitimate political authority, e.g. a tribal council that has the authority to make binding decisions on behalf of its members.” [113]. If used inappropriately, the concept of ‘community consent’ may result in a false sense of security or mandate. We will seek consent for community participation from

community-level stakeholders who will be defined through the community engagement process (see Research Plan) and who will include local leaders. Following agreement to participate, community representatives will take part in a public randomization ceremony at which the allocation of communities to study arms will be decided using a transparent and fair process.

8.6.3 Individual Consent

Individual consent for Arm A
The main aspect of the intervention that goes beyond current guidelines is the offer of immediate commencement of ART regardless of CD4 count or clinical stage in Arm A. As described above, the study team will obtain consent for research from patients in this arm who are offered immediate treatment that is not considered standard of care according to prevailing national treatment guidelines. Any patients declining this offer will be provided with follow-up and treatment in the same health facilities according to current standard of care.

Individual consent for research studies in all Arms
Written informed consent to participate in research will be required before enrolling individuals in the *Population Cohort and Case-Control* studies. Written informed consent will also be required of individuals participating in qualitative research activities that involve collection of participant-identified responses to interviewer questions (such as interviews and focus groups). However written consent will not be sought for other types of qualitative methods, such as observation of persons who are not participants.

Individual consent for CHiP team activities
Because the proposed CHiP team activities are poised between an established public health intervention (home–based testing and outreach) and a public health research project (data collection and additional follow-up), the team will seek from the appropriate ethics committees, an alteration of consent (verbal consent) for participation in the community intervention. Verbal consent will also permit data collected by CHiPs to be used in aggregate form for research purposes. This verbal consent will be accompanied by a written information leaflet that will be provided to all households. This information leaflet will contain information about the project as a whole, as well as appropriate local research team contact details, and will also describe the option of each household not to engage with the CHiP teams or receive any additional visits.

There is adequate prior research to show that household delivery of HIV testing, and linkage to services by community health workers is safe and effective, such that its deployment in this study could be considered a public health intervention, and therefore not requiring written research consent from each of the approximately 800,000 people to be reached by the CHiPs workers. However there are aspects of the CHiPs intervention that are innovative, and go beyond what would be considered an extension of government health services. For example, it is not routine to have CHiP-performed HIV test results entered into a database which is also populated by health center data, and then have those data prompt CHiPs to return to households to follow up with HIV diagnosed participants who have not reported to the health center, in order to provide linkage to care (e.g. early

ART and circumcision). These aspects will thus be included in the initial verbal consent process.

In summary, an alteration of consent (verbal) will be requested based on the following considerations which comply with the U.S. Code of Federal Regulations (CFR) *45 CFR 46.116 (d)*:

(i) As already described CHiP activities involve delivering community based health care (rather than primarily research activities) and involve minimal risk.
(ii) Requiring full written research consent from all individuals that come into contact with the CHiP teams would make this project logistically unfeasible
(iii) Rights and welfare of individuals will not be adversely affected by a verbal consent process that will be documented by the CHiP teams
(iv) Pertinent written information about the project will be provided to all households visited.

Individual written consent for HIV testing and other interventions such as male circumcision will be obtained using standard procedures as these interventions are considered part of the routine delivery of HIV prevention services and not specifically study related. Thus individuals who undergo these procedures in the study communities will not specifically be asked to participate in a research study, but rather will be asked to consent to these activities as part of their health care.

The study team has considerable experience of designing and implementing suitable models of informed consent for study populations in resource-poor settings in Zambia and South Africa that may have low levels of functional literacy. Care will be taken to ensure that information materials are developed that are appropriate to the study population, with translation into a local language where necessary and back-translation into English to ensure accuracy. Project staff will go through the information sheets with participants and questions will be asked to check their understanding of key points before signed consent is sought. Illiterate participants will be asked to give fingerprint consent witnessed by a literate individual who will sign that the individual has been given sufficient information to allow for an informed decision and has given their full consent voluntarily.

8.6.4 Waiver of Individual Consent to Access CHiP and Routine Clinic Data

A waiver of individual consent will be requested to access CHiP and routine clinic data and record them for research purposes in anonymized form. This request is justified by the following considerations which comply with *45 CFR 46.116 (d)*:

(i) The research involves no more than minimal risk to participants as the data will be de-identified and presented in aggregate form to the research teams that will be analyzing the data for research purposes.
(ii) The rights and welfare of research participants will not be adversely affected in any way by the collection of these data, which will be

stored confidentially by the CHiP team members and shared only in de-identified form with the research teams

(iii) The research could not practicably be carried out without the waiver, as attempting to obtain written informed consent from all community members involved in a study of this scale would not be feasible.

(iv) Household members will be provided with pertinent data about the project and the need to gather and report on the information gathered by the CHiP teams as well as certain routine clinic data.

8.7 Independent Ethical Review

Approval to conduct this study will be obtained from the following IRBs/ECs. In instances where there is disagreement or discordant IRB requirements the condition providing the highest level of human subject protection will be implemented. Approval must be obtained from the local, and national (where relevant) IRBs before the study can be initiated.

Ethical clearance for the trial will be sought from Institutional Review Boards (IRBs) in the United Kingdom (UK), Zambia and South Africa. Adverse events will be reported on a regular basis according to the individual requirements of these IRBs.

8.8 Respect for Participants and Communities During and After the Study

8.8.1 Confidentiality

Strict measures will be in place to safeguard confidentiality of data. All laboratory specimens, reports, study data collection, process, and administrative forms will be identified by coded numbers only to maintain participant confidentiality.
Personal identifiers (name, address, global positioning system coordinates) will only be collected for (1) informed consent and (2) operational and logistical purposes (i.e. to ensure tracing of participants by intervention staff and to locate cohort participants for follow-up visits). Personal identifiers will appear on paper or electronically on appointment books, consent forms, log books, follow up lists and other listings. These listings will NOT include any (sensitive) study information (including laboratory data). A unique study number will be used to link personal identifiers to study information.

Personal identifiers on paper will be stored in a locked cabinet. Electronically kept personal identifiers will be stored in separate datasets with password protection only accessible for designated staff (for computers and servers). Hand-held devices will also be password protected and personal identifiers will be stored in an encrypted format.

Participants' study information will not be released without the written permission of the participant, except as necessary for monitoring by the National Institute of Allergy and Infectious Diseases (NIAID) and/or its contractors, representatives of the HPTN CORE, SDMC, and/or NL, other government and regulatory authorities, and/or site IRBs/ECs. Datasets transferred to locations outside the study sites (e.g. for analyses, progress reports) will be stripped of any personal identifier before transfer.

All electronic data will be stored in password protected database systems. Read and write authorization of data will depend on the designation of the staff member. A second layer of protection is hardware password protection on computers, servers and networks. Thirdly data transfer over wireless or mobile networks will use Virtual Private Networks or router protected dedicated internet protocol addresses.

All collected study data on central computers and servers, remote computers and hand-held devices, will be backed up daily. Backup tapes/discs will be stored separately from the primary electronic storage.

8.8.2 Data and Safety Monitoring Plan

An independent data safety monitoring board (DSMB) will be established according to accepted international norms. The membership of the committee will include expertise in HIV prevention, statistics, cluster-randomized trials and clinical medicine (including antiretroviral therapy). The responsibilities of the DSMB will be to monitor data from the trial and to advise the sponsor and study leadership on any recommended changes to the conduct of the study including early termination for futility on the primary endpoint if appropriate. A formal interim analysis is not anticipated as it is important to measure the effect of the intervention over the full three year follow-up period. However, data from the study communities on operational performance including uptake, retention and adverse events will be reported to the DSMB and reviewed on an ongoing basis.

Data on the uptake of trial interventions - in particular HIV testing and treatment, retention in HIV care and medical male circumcision - will be captured electronically in all trial communities, facilitating timely analysis. We will monitor intervention uptake on a monthly basis. We will use these data for the trial comparison of Arm A (immediate treatment) vs. Arm C (standard-of-care). If study power falls below a pre-specified threshold, then the DSMB will consider whether the trial should be stopped for futility. This pre-specified threshold will be defined in consultation with the DSMB prior to the start of trial interventions.

8.8.3 Communicable Disease Reporting Requirements

Study staff will comply with national requirements to notify tuberculosis identified among study participants to local health authorities. Participants will be made aware of all reporting requirements during the study informed consent process. HIV is not a notifiable disease in either country.

8.8.4 Post-Trial Management of Participants Exposed to the Early ARV Intervention

Any individual started on ART during the trial will continue this therapy after the trial since there is no current guidance to stop ART once it has been started. This treatment will be provided through the national health systems and this has been discussed and is understood by all HIV care implementing agencies in the study communities.

8.8.5 Study Discontinuation

The study also may be discontinued at any time by NIAID, the HPTN, other government or regulatory authorities, and/or site IRBs/ECs.

9.0 LABORATORY SPECIMENS AND BIOHAZARD CONTAINMENT

9.1 Local Laboratory Specimens

"Local Laboratory" in this study refers to regional laboratories and centralized laboratories in each country. Laboratory testing will be performed using stored samples to meet study objectives. In most cases, the results of testing performed using stored samples will not be returned to study sites or participants. The HPTN NL will determine the location of testing. Tests performed by Local Laboratories are described in more detail in Appendix I and the SSP Manual. Local Laboratories performing these tests must demonstrate successful participation in relevant External Quality Assurance (EQA) programs. The EQA results may be monitored by Patient Safety Monitoring and International Laboratory Evaluation (pSMILE) at the discretion of the HPTN NL.

Each study site and Local Laboratory will adhere to standards of Good Clinical Laboratory Practice (GCLP), the laboratory SSP manual, and all activities related to processing, labeling, testing, storage, transport and shipping (to centralized laboratories or to the HPTN NL). Specimen collection and storage at selected Local Laboratories will be documented using the HPTN Laboratory Data Management System (LDMS), as described in the SSP Manual.

All specimens will be shipped in accordance with local shipping regulations as well as International Air Transport Association (IATA) specimen shipping regulations. The HPTN NL will determine which shipments will be documented using the HPTN LDMS, as described in the SSP Manual.

As described in Section 5, the following types of specimens will be collected for testing at the Local Laboratory:

***Population Cohort*:**

- Blood specimen for the following:
  - HIV testing
  - Plasma
  - HSV-2 testing

9.2 Network Laboratory Specimens

Stored samples will be used for retrospective, centralized testing at the HPTN Network Lab (NL). Testing at the HPTN NL will include HIV and HSV-2 testing (to confirm results obtained in country, and for quality assurance (QA), including confirmation of HIV and HSV-2 seroconversion events). If funded, the HPTN NL will also test samples for viral load and antiretroviral drug resistance. Other testing may be performed, including

cross-sectional HIV incidence testing, analysis of the linkage of HIV infection, and testing for antiretroviral drugs; testing may also be performed to detect other substances, such as recreational substances and substances of abuse. Selected samples may also be tested to characterize the HIV virus and the host response to HIV infection. In some cases, testing may be performed at a commercial laboratory or other laboratory designated by the HPTN NL.

It is important to note that the volume of plasma that can be stored at each study visit is limited, due to the very large number of participants in the study. In some cases, testing will be performed at the HPTN NL (rather than at the Local Laboratories) so that specialized methods can be used that require lower plasma volumes, and so that derivatives generated during testing (e.g., plasma supernatant, HIV RNA, polymerase chain reaction amplicons) can be saved and used for other types of testing. This will increase the likelihood that there will be sufficient stored plasma for all of the planned assessments.

9.3 Quality Control and Quality Assurance Procedures

NL staff will conduct periodic visits to each site to assess the implementation of on-site laboratory quality control (QC) procedures, including proper processing, labeling, storage, proper maintenance of laboratory testing equipment and use of appropriate reagents. NL staff will follow up directly with site staff to resolve any QC or QA problems identified through proficiency testing and/or on-site assessments. Throughout the course of the study, the HPTN NL will select a random sample of stored specimens to test for QA purposes. NL staff will follow-up directly with site staff to resolve any QA problems identified through this process.

9.4 Specimen Storage and Possible Future Research Testing

Study site staff will store plasma collected in this study until all protocol-related testing is complete. Note that some testing will be performed retrospectively, after the last participant completes the final study visit. Protocol testing will include QC testing and other testing performed at or coordinated by the HPTN NL. The study site will be informed by the SDMC when shipments to the HPTN NL are required, and will be instructed which samples to ship. Stored samples may also be used to evaluate methods for cross-sectional HIV incidence determination, to evaluate the linkage of HIV infections, to characterize HIV viruses (e.g., HIV genotyping, HIV subtyping, HIV sequencing and phylogenetics, HIV tropism), and to evaluate the host response to HIV infection. Testing may also be performed to evaluate the presence of antiretroviral drugs in study samples.

In addition, study participants will be asked to provide written informed consent for their specimens to be stored after the end of the study for possible future testing (long-term storage). The specimens of participants who do not consent to long-term storage and additional testing will be destroyed at the end of the study, when all protocol-related testing has been completed.

9.5 Biohazard Containment

As the transmission of HIV and other blood-borne pathogens can occur through contact with contaminated needles, blood, and blood products, appropriate blood and secretion precautions will be employed by all personnel in the drawing of blood and shipping and handling of all specimens for this study, as currently recommended by the U.S. Centers for Disease Control and Prevention (CDC). All infectious specimens will be transported in accordance with U.S. regulations [42 Code of Federal Regulations (CFR) 72].

10.0 ADMINISTRATIVE PROCEDURES

10.1 Protocol Registration

Prior to implementation of this protocol, and any subsequent full version amendments, each site must have the protocol and the protocol consent form(s) approved, as appropriate, by their local IRB/EC and any other applicable regulatory entity (RE). Upon receiving final approval, sites will submit all required protocol registration documents to the DAIDS Protocol Registration Office (DAIDS PRO) at the Regulatory Services Center (RSC). The DAIDS PRO will review the submitted protocol registration packet to ensure that all of the required documents have been received.

Site-specific informed consent forms (ICFs) WILL be reviewed and approved by the DAIDS PRO and sites will receive an Initial Registration Notification from the DAIDS PRO that indicates successful completion of the protocol registration process. A copy of the Initial Registration Notification should be retained in the site's regulatory files.

Upon receiving final IRB/EC and any other applicable RE approval(s) for an amendment, sites should implement the amendment immediately. Sites are required to submit an amendment registration packet to the DAIDS PRO at the RSC. The DAIDS PRO will review the submitted protocol registration packet to ensure that all the required documents have been received. Site-specific ICF(s) WILL NOT be reviewed and approved by the DAIDS PRO and sites will receive an Amendment Registration Notification when the DAIDS PRO receives a complete registration packet. A copy of the Amendment Registration Notification should be retained in the site's regulatory files.

For additional information on the protocol registration process and specific documents required for initial and amendment registrations, refer to the current version of the DAIDS Protocol Registration Manual.

10.2 Study Activation

Pending successful protocol registration and submission of all required documents (see Section 10.1 above); CORE staff will “activate” the site to begin study operations. Study implementation may not be initiated until a study activation notice is provided to the site.

10.3 Study Coordination

Study implementation will be directed by this protocol as well as the SSP manual. The SSP manual — which will contain reference copies of the *Requirements for Source Documentation in DAIDS Funded and/or Sponsored Clinical Trials*, as well as the DAIDS Manual for Expedited Reporting of Adverse Events to DAIDS, Version 2.0, dated January 2010 and the DAIDS Toxicity Tables — will outline procedures for conducting study visits; data and forms processing; AE assessment, management and reporting; dispensing study products and documenting product accountability; and other study operations.

Study case report forms, electronic data capture tools, and other study instruments will be developed by the protocol team and HPTN SDMC. The study data from all sources ultimately will be transferred to the HPTN SDMC for storage and analysis. Quality control reports and queries will be generated and distributed to the study sites on a routine schedule for verification and resolution.

Close coordination between protocol team members will be necessary to track study progress, respond to queries about proper study implementation, and address other issues in a timely manner. Rates of accrual, adherence, follow-up, and AE incidence will be monitored closely by the team as well as the HPTN Study Monitoring Committee. The Protocol Chair, DAIDS Medical Officer, Protocol Biostatistician, SDMC Project Manager, and CORE Protocol Specialist will address issues related to study eligibility and AE management and reporting as needed to assure consistent case management, documentation, and information-sharing across sites.

10.4 Study Monitoring

On-site study monitoring will be performed in accordance with DAIDS policies. Study monitors will visit the site to

- Verify compliance with human subjects and other research regulations and guidelines;

- Assess adherence to the study protocol, study-specific procedures manual, and local counseling practices; and

- Confirm the quality and accuracy of information collected at the study site and entered into the study database.

Site investigators will allow study monitors to inspect study facilities and documentation (e.g., informed consent forms, health center and laboratory records, other source documents, case report forms), as well as observe the performance of study procedures. Investigators also will allow inspection of all study-related documentation by authorized representatives of the HPTN CORE, SDMC, NL, NIAID,US and in-country government and regulatory authorities and IRBs/ECs. A site visit log will be maintained at the study site to document all visits.

10.5 Protocol Compliance

The study will be conducted in full compliance with the protocol. The protocol will not be amended without prior written approval by the Protocol Chair and NIAID Medical Officer. All protocol amendments must be submitted to and approved by the relevant IRB(s)/EC(s) and the DAIDS Regulatory Support Center (RSC) prior to implementing the amendment.

10.6 Investigator's Records

The study site investigator will maintain, and store in a secure manner, complete, accurate and current study records throughout the study. The investigator will retain all study records for at least three years after submission of the CTU's final Financial Status Report to DAIDS, which is due within 90 days after the end of the CTU's cooperative agreement with DAIDS, unless otherwise specified by DAIDS or the HPTN CORE. Study records include administrative documentation — including protocol registration documents and all reports and correspondence relating to the study — as well as documentation related to each participant screened for and/or enrolled in the study — including informed consent forms, locator forms, case report forms, notations of all contacts with the participant, and all other source documents.

10.7 Use of Information and Publications

Publication of the results of this study will be governed by the HPTN Manual of Operations and policies. Any presentation, abstract, or manuscript will be submitted by the Investigator to the HPTN Manuscript Review Committee for review prior to submission.

11.0 REFERENCES

1. UNAIDS, *Report on the Global AIDS Epidemic.* 2010.
2. Beck, E.J., X.M. Santas, and P.R. Delay, *Why and how to monitor the cost and evaluate the cost-effectiveness of HIV services in countries.* AIDS, 2008. **22 Suppl 1**: p. S75-85.
3. De Cock, K.M., et al., *Can antiretroviral therapy eliminate HIV transmission?* Lancet, 2009. **373**(9657): p. 7-9.
4. Hankins, C.A. and B.O. de Zalduondo, *Combination prevention: a deeper understanding of effective HIV prevention.* AIDS, 2010. **24 Suppl 4**: p. S70-80.
5. Pirrone, V., et al., *Combinatorial approaches to the prevention and treatment of HIV-1 infection.* Antimicrob Agents Chemother, 2011. **55**(5): p. 1831-42.
6. Templeton, D.J., *Male circumcision to reduce sexual transmission of HIV.* Curr Opin HIV AIDS, 2010. **5**(4): p. 344-9.
7. Volmink, J., et al., *Antiretrovirals for reducing the risk of mother-to-child transmission of HIV infection.* Cochrane Database Syst Rev, 2007(1): p. CD003510.
8. Johri, M. and D. Ako-Arrey, *The cost-effectiveness of preventing mother-to-child transmission of HIV in low- and middle-income countries: systematic review.* Cost Eff Resour Alloc, 2011. **9**: p. 3.
9. Buchbinder, S.P. and A. Liu, *Pre-exposure prophylaxis and the promise of combination prevention approaches.* AIDS Behav, 2011. **15 Suppl 1**: p. S72-9.
10. Granich, R., et al., *Highly active antiretroviral treatment as prevention of HIV transmission: review of scientific evidence and update.* Curr Opin HIV AIDS, 2010. **5**(4): p. 298-304.
11. Cohen, M.S., et al., *Prevention of HIV-1 infection with early antiretroviral therapy.* N Engl J Med, 2011. **365**(6): p. 493-505.
12. Cohen, M.S. and S. Fidler, *HIV prevention 2010: where are we now and where are we going?* Curr Opin HIV AIDS, 2010. **5**(4): p. 265-8.
13. Weber, J., R. Tatoud, and S. Fidler, *Postexposure prophylaxis, preexposure prophylaxis or universal test and treat: the strategic use of antiretroviral drugs to prevent HIV acquisition and transmission.* AIDS, 2010. **24 Suppl 4**: p. S27-39.
14. World Health Organisation, *Consultation on Antiretroviral Treatment for Prevention of HIV Transmission.* Meeting report, 2009.
15. Cohen, M.S. and C.L. Gay, *Treatment to prevent transmission of HIV-1.* Clin Infect Dis, 2010. **50 Suppl 3**: p. S85-95.
16. Centers for Disease Control and Prevention, *Vital signs: HIV prevention through care and treatment--United States.* MMWR Morb Mortal Wkly Rep, 2011. **60**: p. 1618-23.
17. Burns DN, D.C., Vermund SH, *Rethinking Prevention of HIV Type 1 Infection.* Clinical Infectious Diseases, 2010. **51**(6): p. 725-731.
18. Gardner EM, M.M., Steiner JF, Del Rio C, Burman WJ, *The Spectrum of Engagement in HIV Care and Its Relevance to Test-and-Treat Strategies for Prevention of HIV Infection.* Clinical Infectious Diseases, 2011. **52**(6): p. 793-800.
19. Abbas, U.L., R.M. Anderson, and J.W. Mellors, *Potential impact of antiretroviral chemoprophylaxis on HIV-1 transmission in resource-limited settings.* PLoS One, 2007. **2**(9): p. e875.
20. Abbas, U.L., R.M. Anderson, and J.W. Mellors, *Potential impact of antiretroviral therapy on HIV-1 transmission and AIDS mortality in resource-limited settings.* J Acquir Immune Defic Syndr, 2006. **41**(5): p. 632-41.

21. Blower, S., et al., *Predicting the impact of antiretrovirals in resource-poor settings: preventing HIV infections whilst controlling drug resistance.* Curr Drug Targets Infect Disord, 2003. **3**(4): p. 345-53.
22. Castilla, J., et al., *Effectiveness of highly active antiretroviral therapy in reducing heterosexual transmission of HIV.* J Acquir Immune Defic Syndr, 2005. **40**(1): p. 96-101.
23. Velasco-Hernandez, J.X., H.B. Gershengorn, and S.M. Blower, *Could widespread use of combination antiretroviral therapy eradicate HIV epidemics?* Lancet Infect Dis, 2002. **2**(8): p. 487-93.
24. Lima, V.D., et al., *Expanded access to highly active antiretroviral therapy: a potentially powerful strategy to curb the growth of the HIV epidemic.* J Infect Dis, 2008. **198**(1): p. 59-67.
25. Granich, R.M., et al., *Universal voluntary HIV testing with immediate antiretroviral therapy as a strategy for elimination of HIV transmission: a mathematical model.* Lancet, 2009. **373**(9657): p. 48-57.
26. Dodd, P.J., G.P. Garnett, and T.B. Hallett, *Examining the promise of HIV elimination by 'test and treat' in hyperendemic settings.* AIDS, 2010. **24**(5): p. 729-35.
27. Conway, B. and H. Tossonian, *Comprehensive Approaches to the Diagnosis and Treatment of HIV Infection in the Community: Can "Seek and Treat" Really Deliver?* Curr Infect Dis Rep, 2011. **13**(1): p. 68-74.
28. Zachariah, R., et al., *Antiretroviral therapy for HIV prevention: many concerns and challenges, but are there ways forward in sub-Saharan Africa?* Trans R Soc Trop Med Hyg, 2010. **104**(6): p. 387-91.
29. Sterne, J.A., et al., *Timing of initiation of antiretroviral therapy in AIDS-free HIV-1-infected patients: a collaborative analysis of 18 HIV cohort studies.* Lancet, 2009. **373**(9672): p. 1352-63.
30. Ray, M., et al., *The effect of combined antiretroviral therapy on the overall mortality of HIV-infected individuals.* AIDS, 2010. **24**(1): p. 123-37.
31. Kitahata, M.M., et al., *Effect of early versus deferred antiretroviral therapy for HIV on survival.* N Engl J Med, 2009. **360**(18): p. 1815-26.
32. Emery, S., et al., *Major clinical outcomes in antiretroviral therapy (ART)-naive participants and in those not receiving ART at baseline in the SMART study.* J Infect Dis, 2008. **197**(8): p. 1133-44.
33. Organization, W.H., *Global tuberculosis control: WHO report 2011*, 2011.
34. UNAIDS. *Zambia HIV AND AIDS ESTIMATES (2009).* 2009; Available from: http://www.unaids.org/en/regionscountries/countries/zambia/.
35. UNAIDS. *South Africa HIV AND AIDS ESTIMATES (2009).* 2009; Available from: http://www.unaids.org/en/regionscountries/countries/southafrica/.
36. Organization, W.H., *Global HIV/AIDS Response - Epidemic update and health sector progress towards Universal Access*, in *Progress Report 2011*2011.
37. Sloan, C.E., et al., *Newer drugs and earlier treatment: impact on lifetime cost of care for HIV-infected adults.* AIDS, 2012. **26**(1): p. 45-56.
38. Padian, N.S., et al., *Weighing the gold in the gold standard: challenges in HIV prevention research.* AIDS, 2010. **24**(5): p. 621-35.
39. Ross, D.A., *Behavioural interventions to reduce HIV risk: what works?* AIDS, 2010. **24 Suppl 4**: p. S4-14.
40. Cremin, I., et al., *Patterns of self-reported behaviour change associated with receiving voluntary counselling and testing in a longitudinal study from Manicaland, Zimbabwe.* AIDS Behav, 2010. **14**(3): p. 708-15.

41. Bello, G., et al., *Evidence for changes in behaviour leading to reductions in HIV prevalence in urban Malawi.* Sex Transm Infect, 2011. **87**(4): p. 296-300.
42. Coates, T.J., L. Richter, and C. Caceres, *Behavioural strategies to reduce HIV transmission: how to make them work better.* Lancet, 2008. **372**(9639): p. 669-84.
43. Corbett, E.L., et al., *HIV incidence during a cluster-randomized trial of two strategies providing voluntary counselling and testing at the workplace, Zimbabwe.* AIDS, 2007. **21**(4): p. 483-9.
44. Turner, A.N., et al., *Unprotected sex following HIV testing among women in Uganda and Zimbabwe: short- and long-term comparisons with pre-test behaviour.* Int J Epidemiol, 2009. **38**(4): p. 997-1007.
45. Auvert, B., et al., *Randomized, controlled intervention trial of male circumcision for reduction of HIV infection risk: the ANRS 1265 Trial.* PLoS Med, 2005. **2**(11): p. e298.
46. Bailey, R.C., et al., *Male circumcision for HIV prevention in young men in Kisumu, Kenya: a randomised controlled trial.* Lancet, 2007. **369**(9562): p. 643-56.
47. Gray, R.H., et al., *Male circumcision for HIV prevention in men in Rakai, Uganda: a randomised trial.* Lancet, 2007. **369**(9562): p. 657-66.
48. Hallett, T.B., et al., *Understanding the impact of male circumcision interventions on the spread of HIV in southern Africa.* PLoS One, 2008. **3**(5): p. e2212.
49. Lissouba, P., et al., *A model for the roll-out of comprehensive adult male circumcision services in African low-income settings of high HIV incidence: the ANRS 12126 Bophelo Pele Project.* PLoS Med, 2010. **7**(7): p. e1000309.
50. Ward, H. and M. Ronn, *Contribution of sexually transmitted infections to the sexual transmission of HIV.* Curr Opin HIV AIDS, 2010. **5**(4): p. 305-10.
51. Grosskurth, H., et al., *Impact of improved treatment of sexually transmitted diseases on HIV infection in rural Tanzania: randomised controlled trial.* Lancet, 1995. **346**(8974): p. 530-6.
52. Ng, B.E., et al., *Population-based biomedical sexually transmitted infection control interventions for reducing HIV infection.* Cochrane Database Syst Rev, 2011(3): p. CD001220.
53. Rerks-Ngarm, S., et al., *Vaccination with ALVAC and AIDSVAX to prevent HIV-1 infection in Thailand.* N Engl J Med, 2009. **361**(23): p. 2209-20.
54. Willyard, C., *Tiny steps towards an HIV vaccine.* Nature, 2010. **466**(7304): p. S8.
55. Barouch, D.H. and B. Korber, *HIV-1 vaccine development after STEP.* Annu Rev Med, 2010. **61**: p. 153-67.
56. Abdool Karim, Q., et al., *Effectiveness and safety of tenofovir gel, an antiretroviral microbicide, for the prevention of HIV infection in women.* Science, 2010. **329**(5996): p. 1168-74.
57. Greene, E., et al., *Acceptability and adherence of a candidate microbicide gel among high-risk women in Africa and India.* Cult Health Sex, 2010. **12**(7): p. 739-54.
58. Kamali, A., et al., *A randomised placebo-controlled safety and acceptability trial of PRO 2000 vaginal microbicide gel in sexually active women in Uganda.* Sex Transm Infect, 2010. **86**(3): p. 222-6.
59. Grant, R.M., et al., *Preexposure chemoprophylaxis for HIV prevention in men who have sex with men.* N Engl J Med, 2010. **363**(27): p. 2587-99.
60. Baeten, J. and C. Celum. *Antiretroviral pre-exposure prophylaxis for HIV-1 prevention among heterosexual African men and women: the Partners PrEP Study. Abstract No. MOAX0106.* in *6th International AIDS Society Conference on HIV Pathogenesis, Treatment and Prevention*. 2011. Rome, Italy.

61. Thigpen, M., et al. *Daily oral antiretroviral use for the prevention of HIV infection in heterosexually active young adults in Botswana: results from the TDF2 study Abstract No. WELBC01*. in *6th IAS Conference on HIV Pathogeneisis, Treatment and Prevention*. 2011 Rome, Italy
62. Microbicide Trials Network (MTN). *MTN Statement on Decision to Discontinue Use of Tenofovir Gel in VOICE, a Major HIV Prevention Study in Women*. 2011 [cited 2011 December 12]; Available from: http://www.mtnstopshiv.org/studies/70.
63. UNAIDS, *Getting to Zero: 2011-2015 Strategy*. http://www.unaids.org/en/media/unaids/contentassets/documents/unaidspublication/2010/JC2034_UNAIDS_Strategy_en.pdf, 2010.
64. Wilson, D. and D.T. Halperin, *"Know your epidemic, know your response": a useful approach, if we get it right*. Lancet, 2008. **372**(9637): p. 423-6.
65. Kurth, A.E., et al., *Combination HIV prevention: significance, challenges, and opportunities*. Curr HIV/AIDS Rep, 2011. **8**(1): p. 62-72.
66. Attia, S., et al., *Sexual transmission of HIV according to viral load and antiretroviral therapy: systematic review and meta-analysis*. AIDS, 2009. **23**(11): p. 1397-404.
67. Wawer, M.J., et al., *Rates of HIV-1 Transmission per Coital Act, by Stage of HIV-1 Infection, in Rakai, Uganda*. J Infect Dis, 2005. **191**(9): p. 1403-9.
68. Cohen, M.S., et al., *Narrative review: antiretroviral therapy to prevent the sexual transmission of HIV-1*. Ann Intern Med, 2007. **146**(8): p. 591-601.
69. Gay, C.L. and M.S. Cohen, *Antiretrovirals to prevent HIV infection: pre- and postexposure prophylaxis*. Curr Infect Dis Rep, 2008. **10**(4): p. 323-31.
70. Chasela, C.S., et al., *Maternal or infant antiretroviral drugs to reduce HIV-1 transmission*. N Engl J Med, 2010. **362**(24): p. 2271-81.
71. de Vincenzi, I., *Triple antiretroviral compared with zidovudine and single-dose nevirapine prophylaxis during pregnancy and breastfeeding for prevention of mother-to-child transmission of HIV-1 (Kesho Bora study): a randomised controlled trial*. Lancet Infect Dis, 2011. **11**(3): p. 171-80.
72. Shapiro, R.L., et al., *Antiretroviral regimens in pregnancy and breast-feeding in Botswana*. N Engl J Med, 2010. **362**(24): p. 2282-94.
73. Cohen, M.S., et al., *Prevention of HIV-1 Infection with Early Antiretroviral Therapy*. N Engl J Med, 2011.
74. Lawn, S.D., et al., *Early mortality among adults accessing antiretroviral treatment programmes in sub-Saharan Africa*. AIDS, 2008. **22**(15): p. 1897-908.
75. Donnell, D., et al., *Heterosexual HIV-1 transmission after initiation of antiretroviral therapy: a prospective cohort analysis*. The Lancet, 2010. **375**(9731): p. 2092-8.
76. Das, S., *Risk of cardiovascular disease in HIV-infected patients*. J Antimicrob Chemother, 2010. **65**(3): p. 386-9.
77. Montaner, J.S., et al., *Association of highly active antiretroviral therapy coverage, population viral load, and yearly new HIV diagnoses in British Columbia, Canada: a population-based study*. Lancet, 2010. **376**(9740): p. 532-9.
78. Garnett, G.P. and R.F. Baggaley, *Treating our way out of the HIV pandemic: could we, would we, should we?* Lancet, 2009. **373**(9657): p. 9-11.
79. Pinkerton, S.D., *Probability of HIV transmission during acute infection in Rakai, Uganda*. AIDS Behav, 2008. **12**(5): p. 677-84.
80. Brenner, B.G., et al., *High rates of forward transmission events after acute/early HIV-1 infection*. J Infect Dis, 2007. **195**(7): p. 951-9.

81. Hollingsworth, T.D., R.M. Anderson, and C. Fraser, *HIV-1 transmission, by stage of infection.* J Infect Dis, 2008. **198**(5): p. 687-93.
82. Bunnell, R., et al., *Changes in sexual behavior and risk of HIV transmission after antiretroviral therapy and prevention interventions in rural Uganda.* AIDS, 2006. **20**(1): p. 85-92.
83. Granich, R., et al., *Universal voluntary HIV testing with immediate antiretroviral therapy as a strategy for elimination of HIV transmission: a mathematical model.* Lancet, 2009. **373**(9657): p. 48-57.
84. Walensky, R.P., et al., *Test and treat DC: forecasting the impact of a comprehensive HIV strategy in Washington DC.* Clin Infect Dis, 2010. **51**(4): p. 392-400.
85. Johnston, K.M., et al., *Expanding access to HAART: a cost-effective approach for treating and preventing HIV.* AIDS, 2010. **24**(12): p. 1929-35.
86. Panel on Antiretroviral Guidelines for Adults and Adolescents, *Guidelines for the Use of Antiretroviral Agents in HIV-1 Infected for Adults and Adolescents,*
Adults and Adolescents,.
http://www.aidsinfo.nih.gov/ContentFiles/AdultandAdolescentGL.pdf., 2011.
87. Health, N.I.o., *US ART Guidelines* D.o.H.a.H. Services, Editor 2012.
88. *BHIVA guidelines for the treatment of HIV-1 positive adults with antiretroviral therapy,* 2012.
89. Lawn, S.D., K. Kranzer, and R. Wood, *Antiretroviral therapy for control of the HIV-associated tuberculosis epidemic in resource-limited settings.* Clin Chest Med, 2009. **30**(4): p. 685-99, viii.
90. Williams, B.G., et al., *Antiretroviral therapy for tuberculosis control in nine African countries.* Proc Natl Acad Sci U S A, 2010. **107**(45): p. 19485-9.
91. Stephenson, R., et al., *The influence of motivational messages on future planning behaviors among HIV concordant positive and discordant couples in Lusaka, Zambia.* AIDS Care, 2008. **20**(2): p. 150-60.
92. Wood, R. and S.D. Lawn, *Antiretroviral treatment as prevention: impact of the 'test and treat' strategy on the tuberculosis epidemic.* Curr HIV Res, 2011. **9**(6): p. 383-92.
93. Lawn, S.D., et al., *Time to initiation of antiretroviral therapy among patients with HIV-associated tuberculosis in Cape Town, South Africa.* J Acquir Immune Defic Syndr, 2011. **57**(2): p. 136-40.
94. Sonnenberg, P., et al., *How soon after infection with HIV does the risk of tuberculosis start to increase? A retrospective cohort study in South African gold miners.* J Infect Dis, 2005. **191**(2): p. 150-8.
95. Badri, M., D. Wilson, and R. Wood, *Effect of highly active antiretroviral therapy on incidence of tuberculosis in South Africa: a cohort study.* Lancet, 2002. **359**(9323): p. 2059-64.
96. Leroy V, E.D., Becquet R, Viho I, Dequae-Merchadou L, Tonwe-Gold B, et al., *18-month effectiveness of short-course antiretroviral regimens combined with alternatives to breastfeeding to prevent HIV mother-to-child transmission.* PLoS One, 2008. **3**(2): p. 1645.
97. Thomas TK, M.R., Borkowf CB, Ndivo R, Zeh C, Misore A, et al., *Triple-antiretroviral prophylaxis to prevent mother-to-child HIV transmission through breastfeeding - the Kisumu Breastfeeding Study, Kenya: a clinical trial.* PLoS Med, 2011. **8**(3): p. 1001015.
98. Cooper ER, C.M., Mofenson L, Hanson IC, Pitt J, Diaz C, et al., *Combination antiretroviral strategies for the treatment of pregnant HIV-1-infected women and*

prevention of perinatal HIV-1 transmission. J Acquir Immune Defic Syndr, 2002. **29**(5): p. 484-94.

99. Price, M.A., et al., *Transmitted HIV type 1 drug resistance among individuals with recent HIV infection in East and Southern Africa.* AIDS Res Hum Retroviruses, 2011. **27**(1): p. 5-12.
100. UNAIDS. *Report on the Global AIDS Epidemic*. 2010 12 May 2011.
101. Ayles, H., *Personal Communication 11 January 2012*.
102. Hollingsworth TD, A.R., Fraser C, *HIV-1 transmission, by stage of infection.* J Infect Dis, 2008. **198**: p. 687-693.
103. Hayes, R. and L. Moulton, *Cluster Randomised Trials*. Equations 7.132009.
104. Getahun, H., et al., *Development of a standardized screening rule for tuberculosis in people living with HIV in resource-constrained settings: individual participant data meta-analysis of observational studies.* PLoS Med, 2011. **8**(1): p. e1000391.
105. Harries, A.D., et al., *The HIV-associated tuberculosis epidemic--when will we act?* Lancet, 2010. **375**(9729): p. 1906-19.
106. Lawn, S.D., et al., *Antiretrovirals and isoniazid preventive therapy in the prevention of HIV-associated tuberculosis in settings with limited health-care resources.* Lancet Infect Dis, 2010. **10**(7): p. 489-98.
107. Organization, W.H., *Antiretroviral Treatment as Prevention (Ta sP) of HIV and TB*, 2012.
108. Weiss, H., M. Quigley, and R. Hayes, *Male circumcision and risk of HIV infection in sub-Saharan Africa: a systematic review and meta-analysis.* AIDS, 2000. **14**(15): p. 2361-2370.
109. Lissouba, P., et al., *Adult male circumcision as an intervention against HIV: an operational study of uptake in a South African community (ANRS 12126).* BMC Infect Dis, 2011. **11**: p. 253.
110. Wawer, M., G. Kigozi, and D. Swerwadda, *Trial of male circumcision in HIV+ men, Rakai Uganda: effects in HIV+ men and in women partners*, in *15th CROI*2008, CROI: Boston.
111. Mehta, S.D., et al., *Does sex in the early period after circumcision increase HIV-seroconversion risk? Pooled analysis of adult male circumcision clinical trials.* AIDS, 2009. **23**(12): p. 1557-64.
112. Cohen, M.S., et al., *Prevention of HIV-1 Infection with Early Antiretroviral Therapy.* New England Journal of Medicine, 2011. **365**(6): p. 493-505.
113. Weijer, C. and E.J. Emanuel, *Protecting communities in biomedical research.* Science, 2000. **289**: p. 1142-1144.

12.0 APPENDICES

APPENDIX I - SCHEDULES OF STUDY VISITS AND PROCEDURES

APPENDIX IA: POPULATION COHORT- ALL ARMS

PROCEDURES	Enrollment	12 Month Follow-Up	24 Month Follow-Up	36 Month Follow-Up
**ADMINISTRATIVE, BEHAVIORAL, AND REGULATORY PROCEDURES**				
Obtain informed consent for enrollment.<br>Solicit consent for storage of specimens for future testing and for access to data collected at health centers	X			
Obtain/update locator information.	X	X	X	X
Administer survey to include socio-demographic, health, social, behavioral, and economic factors	X	X	X	X
**CLINICAL/COUNSELING PROCEDURES**				
Perform HIV rapid testing[1]	X	X	X	X
Collect blood for laboratory testing and sample storage.	X	X	X	X
Provide HIV pre- and post-test counseling and test results, for those receiving HIV rapid testing and willing to receive results	X	X	X	X
**LABORATORY PROCEDURES**				
Plasma for storage[2]	X	X	X	X
HIV testing using stored samples[3]	X	X	X	X
HIV viral load testing using stored plasma[2,4] (if funding is available)	X	X	X	X
HSV-2 testing using stored plasma[2, 3,5]	X	[X][5]	[X][5]	[X][5]
HIV resistance testing using stored samples[6] (if funding is available)	X	X	X	X
Other testing using stored samples[7]	X	X	X	X

Footnotes for the *Population Cohort*

[1] Rapid testing will be offered at home visits and performed according to in-country guidelines. This testing will not be used to estimate HIV incidence or prevalence; however, the data may be captured along with other data from the home visit. Tie-breaker testing may or may not be performed in the home.

[2] Plasma samples will be stored at an in-country centralized laboratory. Stored samples will be used for testing at one or more centralized laboratories and at the HPTN Network Laboratory in the U.S. Some testing may be performed at other laboratories in the US at the discretion of the HPTN Network Laboratory. Testing performed on stored samples will not be performed in real-time. In most cases, results will not be returned to study sites or participants. Samples will be shipped to the HPTN NL on request.

[3] This testing may be performed at a centralized in-country laboratory designated by the HPTN NL.

[4] HIV viral load testing using stored samples will be performed (if funding is available) to determine community viral load, ART adherence and viral suppression.

[5] HSV-2 testing will be performed for all participants at enrollment; testing will not be performed at 12, 24, or 36 months if the HSV-2 test from a previous visit was positive. The HPTN NL will perform QA testing for HSV-2.

[6] Testing for antiretroviral drug resistance (if funding is available) will be performed on selected samples at a centralized laboratory selected by the HPTN NL.

[7] Additional testing will be performed at the HPTN NL or at another laboratory at the discretion of the HPTN NL. Retrospective HIV testing will be performed for quality assurance and to determine HIV prevalence and HIV incidence. This may include testing related to cross-sectional assessment of HIV incidence. Results from this testing will not be returned to study participants; they will have access to HIV diagnostic testing during the home visit and at the health centers. Quality assurance testing may also be performed to evaluate in-country HSV-2 testing. A subset of samples may also be used to characterize the HIV virus and the host response to HIV infection, to analyze linkage of HIV infections, or to measure/detect the presence of antiretroviral drugs in samples.

APPENDIX 1B- CASE-CONTROL STUDIES 1-3- ARMS A & B

PROCEDURES	Enrollment
**ADMINISTRATIVE, BEHAVIORAL, AND REGULATORY PROCEDURES**	
Obtain informed consent for enrollment	X
Complete questionnaire of socio-demographic, clinical, and behavioral characteristics	X

APPENDIX II - SAMPLE INFORMED CONSENT FORM – POPULATION COHORT

SUBJECT INFORMATION AND CONSENT FORM

**Title of Research Study:**	**Population Effects of Antiretroviral Therapy to Reduce HIV Transmission (PopART): A cluster-randomized trial of the impact of a combination prevention package on population-level HIV incidence in Zambia and South Africa**
**Protocol #:**	HPTN 071, Version 1.0, 26 October 2012<br>DAIDS ID: 11865
**Sponsor:**	National Institute of Allergy and Infectious Diseases<br>National Institute of Mental Health<br>(U.S. National Institutes of Health)<br>Office of the United States Global AIDS Coordinator<br>Bill and Melinda Gates Foundation
**Investigator of Record:**	*(insert name)*
**Research Site Address(es):**	*(insert address)*
**Daytime telephone number(s):**	*(insert number)*
**24-hour contact number(s):**	*(insert number)*

Subject Information and Consent Form
Please ask the study investigator or the study staff to explain any words or procedures that you do not clearly understand.

The purpose of this form is to give you information about the research study you are being asked to join. If you sign this form, you will be giving your permission to take part in the study. The form describes the purpose, procedures, benefits, and risks of the research study. You should take part in the study only if you want to do so. You may choose not to join the research project or withdraw from this study at any time. Choosing not to take part in this research will not in any way affect the health care or benefits that you or your family will receive. Please read this Subject Information and Consent Form and ask as many questions as needed. You should not sign this form if you have any questions that have not been answered to your satisfaction.

This study is being funded by the U.S. National Institutes of Health, the Office of the United States Global AIDS Coordinator, and the Bill and Melinda Gates Foundation

Your participation is voluntary
You do not have to take part in this study. If you decide today to take part in this research project, you may refuse to take part in any portion of the study or stop at any time without reducing or affecting any care that you receive at the health centers in your community.

Purpose of the Research in the Communities
The HPTN 071 or PopART study is testing a program to try to reduce HIV infection in a community like yours. Twenty one communities that include about 600,000 adults are included in this research (about 400,000 adults in twelve Zambian communities and 200,000 adults in nine South African communities).

In some communities, the level of care that people are used to will stay the same, in terms of HIV testing, and care of those who have HIV.

In other communities, to make HIV testing easier, community health care workers will go to all homes and will offer to do an HIV test on each adult (or younger people with permission of guardian) wishing to have a test. For anyone infected with HIV, they will be offered to start taking drugs to treat HIV according to the standard treatment guidelines that are in place for doing so in your country. The health workers will visit every home again once a year for up to three more years to repeat the HIV testing and to refer people to care.

In other communities, health care workers will go to all houses offering HIV testing, as was just described. In these communities if someone tests HIV positive however, they will be offered to start taking medicines to treat HIV right away. The health workers will visit every home again once a year for up to three more years to repeat the HIV testing and to refer people to care.

At the end of the study, the researchers will see if offering HIV tests in each household and offering people the chance to start HIV treatment right away has reduced HIV infection. They will also see if starting ART early has any negative effects on people's health.

Your community is one of the communities participating in this research. If health care workers are visiting homes in your community, you will notice that they provide some other information and services to people, but the most important thing is the testing and HIV treatment they offer.

In each community, around 2,700 people will be asked to participate in additional activities such as completing questionnaires and providing additional samples for laboratory testing. These questionnaires and tests will let the researchers understand how the community feels about the program and if the program is working. You have been selected to be one of the people from your community who we are asking to participate in these additional activities. That is why you are being asked to read this document.

What will happen during this study?
If you participate in this study, you will have up to four study visits: today, in 12 months, in 24 months, and possibly a final visit in 36 months. We will contact you to remind you about your visits. For example, we may call you or send a short text message (SMS). Today's visit will take approximately 2 hours. Future visits may be slightly shorter. Today we will:

- Ask you questions about a number of topics including you and your sexual practices, HIV testing, male circumcision, and how you and others feel about HIV.

- Collect up to 15 mL blood (about 3 teaspoons) for HIV testing and other HIV-related tests as well as herpes simplex-2 testing. Some blood will be stored for study-related testing.

Sometimes at the end of a study, some blood or other specimens are left-over that could be useful for testing in the future. These tests would be for research that is not a part of this study. If you agree to participate in the study, we will also ask if you if are willing to let us keep your left-over samples for future tests.

Some specimens will be shipped and/or securely stored outside of the country for study-related testing, long-term storage, and future testing.

If you agree to participate in the study, we will offer to perform an on-the-spot HIV test at each visit, and will provide counseling if you would like to know the result of your test. If these tests say that you are positive for HIV, we will refer you for care at the local health center. The staff at the health center keep records of all their patients as part of their normal procedures. We would like to look at these medical records for any study participant who is HIV infected. Doing so will help us better understand how the study activities in the community are affecting the health of people diagnosed with HIV. If you agree to participate in this study, we will ask you for your permission to look at your records at the health center. This may include information collected by the community health workers if they are visiting homes in your community.

What are the possible risks or discomforts?
You may become embarrassed, worried or anxious when learning your HIV status and discussing sexual risk behavior and other topics. A trained staff member will help you deal with any feelings or questions you have. You may feel that being part of this study could lead to you feeling stigmatized or separated from our community

It is very unusual to have any problems from having a blood test but you may feel discomfort, dizzy, or even faint when your blood is drawn. Redness, pain, swelling, bruising may occur where the needle goes into your arm but this is rare.

What are the potential benefits?
During the study, you can decide if you would like to learn your HIV status and be provided with information on where to receive treatment and care services if needed. You will also be able to ask questions about your health.

In addition, knowledge gained from this study may help reduce the spread of HIV in the future and promote better health for you and your family as well as helping with acknowledgement and acceptance of HIV as a community-wide health problem.

Are there any alternatives to participation?
If you decide not to participate in this study, we will refer you to other places where you can receive an HIV test. If it is offered in your community, you can also receive testing from a health worker visiting your home during the study period.

How will my confidentiality and privacy be protected?
We cannot guarantee absolute confidentiality. However, we will do everything possible to protect your confidentiality if you join this study. We do this by giving you a study number and

any information will be labeled with this number only, so people working in the health centers and laboratories will only see a number not your name, only the research staff will be able to link this number to your name. Your personal information (name, address, phone number) will be protected by the research staff. This information will not be used in any publication of information about this study.

To protect your privacy, you will meet with the researcher in a private area where others cannot overhear conversations with you.

People who may review your records include: [insert name of site IRB/EC], local regulatory agencies, US National Institutes of Health (NIH), study staff, and study monitors. Institutional Review Boards (IRBs) or Ethics Committees (ECs) are committees that watch over the safety and rights of research participants.

What happens if I am injured by participating in this study?
It is very unlikely that you could be injured as a result of participating in this study. However, if you are injured while participating in this study, you will be given immediate treatment for your injuries. You [will/will not] have to pay for this care. There [is a/is no] program for compensation either through this institution or the United States NIH. You will not be giving up any of your legal rights by signing this Subject Information and Consent Form.

What are some reasons why I may be withdrawn from this activity without my consent?
You may be withdrawn from the study without your consent for the following reasons:

- The research study, or this part of the study, is stopped or canceled
- The study staff feels that completing the study or this part of the study would be harmful to you or others

Persons to Contact for Problems or Questions
If you have any questions about your participation in this research study, your rights as a research subject, or if you feel that you have experienced a research-related injury, contact:

Investigator of Record Name: *(site insert name of the investigator or other study staff)*

Research Site Address(es): *(site insert physical address of above)*

Daytime telephone number(s): *(site insert telephone number)*

24-hour contact number(s): *(site insert telephone number)*

If you have any questions or concerns about your rights as a research subject or want to discuss a problem, get information or offer input, you may contact:

Independent Review Board/Ethics Committee: *(site insert name or title of person on the IRB, EC or other organization appropriate for the site)*

Address of Independent Review Board:*(site insert physical address of above)*

Daytime Telephone Number: *(site insert telephone number of above)*

SUBJECT'S STATEMENT OF CONSENT

Population Effects of Antiretroviral Therapy to Reduce HIV Transmission (PopART): A cluster-randomized trial of the impact of a combination prevention package on population-level HIV incidence in Zambia and South Africa

- I have been given sufficient time to consider whether to take part in this study.
- My taking part in this research study is voluntary. I may decide not to take part or to withdraw from the research study at any time without penalty or loss of benefits or treatment to which I am entitled.
- The research study may be stopped at any time without my consent.
- I have had an opportunity to ask my study investigator questions about this research study. My questions so far have been answered to my satisfaction.
- I have been told how long I may be in the research study.
- I have been informed of the procedures and tests that may be performed during the research study.
- I have been told what the possible risks and benefits are from taking part in this research study. I may not benefit if I take part in this research study.
- I do not give up my legal rights by signing this form.
- I have been told that before any study related procedures being performed, I will be asked to voluntarily sign this Subject Information and Consent Form.
- I will receive a signed and dated copy of this Subject Information and Consent Form.

If you have either read or have heard the information in this Subject Information and Consent Form, if all of your questions have been answered, and if you agree to take part in the study, please print and sign your name and write the date on the line below.

Specimen Storage for Future Testing

_____ My initials indicate that any left-over blood or other specimens may be stored for future testing after the study has ended. I understand that any future research on my specimens may need to be approved by an ethics committee.

_____ I do not agree to allow leftover samples to be saved for long-term storage and future testing after the study has ended.

Access of Data from Heath Center

_____ My initials indicate that I agree to allow my records at the health center to be accessed and used for this study.

_____ I do not agree to allow my health care records to be accessed and used for this study.

I voluntarily agree to take part in this research study.

__________________________	____________________________________
Subject's Name (print)	Subject's Signature and Date

I certify that the information provided was given in a language that was understandable to the subject.

__________________________	____________________________________
Name of Study Staff<br>Conducting Consent Discussion (print)	Study Staff Signature and Date
__________________________	____________________________________
Witness' Name (print)<br>(As appropriate) Date	Witness' Signature and Date

APPENDIX III - SAMPLE INFORMED CONSENT FORM – QUALITATIVE STUDIES PARTICIPANTS

SUBJECT INFORMATION AND CONSENT FORM

**Title of Research Study:**	**Population Effects of Antiretroviral Therapy to Reduce HIV Transmission (PopART): A cluster-randomized trial of the impact of a combination prevention package on population-level HIV incidence in Zambia and South Africa**
**Protocol #:**	HPTN 071, Version 1.0, 26 October 2012<br>DAIDS ID: 11865
**Sponsor:**	National Institute of Allergy and Infectious Diseases<br>National Institute of Mental Health<br>(U.S. National Institutes of Health)<br>Office of the United States Global AIDS Coordinator<br>Bill and Melinda Gates Foundation
**Investigator of Record:**	*(insert name)*
**Research Site Address(es):**	*(insert address)*
**Daytime telephone number(s):**	*(insert number)*
**24-hour contact number(s):**	*(insert number)*

Subject Information and Consent Form
Please ask the study investigator or the study staff to explain any words or procedures that you do not clearly understand.

The purpose of this form is to give you information about the research study you are being asked to join. If you sign this form, you will be giving your permission to take part in the study. The form describes the purpose, procedures, benefits, and risks of the research study. You should take part in the study only if you want to do so. You may choose not to join the research project or withdraw from this study at any time. Choosing not to take part in this research will not in any way affect the health care or benefits that you or your family will receive. Please read this Subject Information and Consent Form and ask as many questions as needed. You should not sign this form if you have any questions that have not been answered to your satisfaction.

This study is being funded by the U.S. National Institutes of Health, the Office of the United States Global AIDS Coordinator, and the Bill and Melinda Gates Foundation

Your participation is voluntary
You do not have to take part in this study. If you decide today to take part in this research project, you may refuse to take part in any portion of the study or stop at any time without reducing or affecting any care that you receive at the health centers in your community.

Purpose of the Research in the Communities
The HPTN 071 or PopART study is testing a program to try to reduce HIV infection in a community like yours. Twenty one communities that include about 600,000 adults are included in this research (about 400,000 adults in twelve Zambian communities and 200,000 adults in nine South African communities).

In some communities, the level of care that people are used to will stay the same, in terms of HIV testing, and care of those who have HIV.

In other communities, to make HIV testing easier, community health care workers will go to all homes and will offer to do an HIV test on each adult (or younger people with permission of guardian) wishing to have a test. For anyone infected with HIV, they will be offered to start taking drugs to treat HIV according to the standard treatment guidelines that are in place for doing so in your country. The health workers will visit every home again once a year for up to three more years to repeat the HIV testing and to refer people to care.

In other communities, health care workers will go to all houses offering HIV testing, as was just described. In these communities if someone tests HIV positive however, they will be offered to start taking medicines to treat HIV right away. The health workers will visit every home again once a year for up to three more years to repeat the HIV testing and to refer people to care.

At the end of the study, the researchers will see if offering HIV tests in each household and offering people the chance to start HIV treatment right away has reduced HIV infection.

Your community is one of the communities participating in this research. If health care workers are visiting homes in your community, you will notice that they provide some other information and services to people, but the most important thing is the testing and HIV treatment they offer.

In each community, around 2,700 people will be asked to participate in additional activities such as completing questionnaires and providing additional samples for laboratory testing. These questionnaires and tests will let the researchers understand how the community feels about the program and if the program is working. You have been selected to be one of the people from your community who we are asking to participate in these additional activities. That is why you are being asked to read this document.

What will happen during this study?

[*For participants providing a single interview*]
If you agree to participate in this study, you will have one interview today. We will ask you questions about the reasons why people in this community chose to test for HIV or not to test for HIV. We will also ask about how people in this community experience HIV treatment and any other HIV prevention methods.

[*For participants being followed longitudinally*]
If you agree to participate in this study, we will interview you every three months until the end of the study. We will ask you questions about the reasons why people in this community chose to test for HIV or not to test for HIV. We will also ask about how people in this community experience HIV treatment and any other HIV prevention methods.

[*For individuals participating in a focus group*]
You have been selected to participate in this group discussion because of either your knowledge of the community or your association with HIV/AIDS related programs and activities in this community. If you agree to participate in this study, you will be a part of a group and questions will be directed towards the group, but you are free to answer any question and comment on the answers of others. In some instances, the facilitator may ask you to elaborate on your answer for the benefit of others but you may choose not to if you are not entirely comfortable with the request. The questions will be broad/ general in nature and will touch on many aspects of the community's experiences with HIV.

What are the possible risks or discomforts?
The risk to you in participating in this study is that some of the questions may be uncomfortable and may make you feel worried or embarrassed. If any of the questions make you feel upset, the interviewer may go to another question or totally stop the interview.

There is also a risk that following up individuals at home may lead to rumors in the community. To minimize this risk we will ask you to propose places where we can talk in private.

What are the potential benefits?
You will not receive any direct benefit from being in this study. You or others may benefit in the future from the information learned in this study.

Are there any alternatives to participation?
If you decide not to participate in this study, you can still receive HIV tests and other services from your local health center.

How will my confidentiality and privacy be protected?
We cannot guarantee absolute confidentiality. However, we will do everything possible to protect your confidentiality if you join this study. We do this by giving you a study number and any information you provide will be labeled with this number only, not your name. Only the research staff will be able to link this number to your name. Your personal information (name, address, phone number) will be protected by the research staff. This information will not be used in any publication of information about this study.

To protect your privacy, you will meet with the researcher in a private area where others cannot overhear conversations with you.

People who may review your records include: [insert name of site IRB/EC], local regulatory agencies, US National Institutes of Health (NIH), study staff, and study monitors. Institutional Review Boards (IRBs) or Ethics Committees (ECs) are committees that watch over the safety and rights of research participants.

What happens if I am injured by participating in this study?
It is very unlikely that you could be injured as a result of participating in this study. However, if you are injured while participating in this study, you will be given immediate treatment for your injuries. You [will/will not] have to pay for this care. There [is a/is no] program for compensation either through this institution or the United States NIH. You will not be giving up any of your legal rights by signing this Subject Information and Consent Form.

What are some reasons why I may be withdrawn from this activity without my consent?
You may be withdrawn from the study without your consent for the following reasons:

- The research study, or this part of the study, is stopped or canceled
- The study staff feels that completing the study or this part of the study would be harmful to you or others

Persons to Contact for Problems or Questions
If you have any questions about your participation in this research study, your rights as a research subject, or if you feel that you have experienced a research-related injury, contact:

Investigator of Record Name: *(site insert name of the investigator or other study staff)*

Research Site Address(es): *(site insert physical address of above)*

Daytime telephone number(s): *(site insert telephone number)*

24-hour contact number(s): *(site insert telephone number)*

If you have any questions or concerns about your rights as a research subject or want to discuss a problem, get information or offer input, you may contact:

Independent Review Board/Ethics Committee: *(site insert name or title of person on the IRB/EC or other organization appropriate for the site)*

Address of Independent Review Board: *(site insert physical address of above)*

Daytime Telephone Number: *(site insert telephone number of above)*

SUBJECT'S STATEMENT OF CONSENT

Population Effects of Antiretroviral Therapy to Reduce HIV Transmission (PopART): A cluster-randomized trial of the impact of a combination prevention package on population-level HIV incidence in Zambia and South Africa

- I have been given sufficient time to consider whether to take part in this study.
- My taking part in this research study is voluntary. I may decide not to take part or to withdraw from the research study at any time without penalty or loss of benefits or treatment to which I am entitled.
- The research study may be stopped at any time without my consent.
- I have had an opportunity to ask my study investigator questions about this research study. My questions so far have been answered to my satisfaction.
- I have been told how long I may be in the research study.
- I have been informed of the procedures and tests that may be performed during the research study.
- I have been told what the possible risks and benefits are from taking part in this research study. I may not benefit if I take part in this research study.
- I do not give up my legal rights by signing this form.
- I have been told that before any study related procedures being performed, I will be asked to voluntarily sign this Subject Information and Consent Form.
- I will receive a signed and dated copy of this Subject Information and Consent Form.

If you have either read or have heard the information in this Subject Information and Consent Form, if all of your questions have been answered, and if you agree to take part in the study, please print and sign and your name and write the date on the line below.

I voluntarily agree to take part in this research study.

______________________________	______________________________
Subject's Name (print)	Subject's Signature and Date

I certify that the information provided was given in a language that was understandable to the subject.

______________________________	______________________________
Name of Study Staff<br>Conducting Consent Discussion (print)	Study Staff Signature and Date

______________________________	______________________________
Witness' Name (print)<br>(As appropriate) Date	Witness' Signature and Date

APPENDIX IV - SAMPLE INFORMED CONSENT FORM – CASE CONTROL STUDIES PARTICIPANTS

SUBJECT INFORMATION AND CONSENT FORM

**Title of Research Study:**	**Population Effects of Antiretroviral Therapy to Reduce HIV Transmission (PopART): A cluster-randomized trial of the impact of a combination prevention package on population-level HIV incidence in Zambia and South Africa**
**Protocol #:**	HPTN 071, Version 1.0, 26 October 2012<br>DAIDS ID: 11865
**Sponsor:**	National Institute of Allergy and Infectious Diseases<br>National Institute of Mental Health<br>(U.S. National Institutes of Health)<br>Office of the United States Global AIDS Coordinator<br>Bill and Melinda Gates Foundation
**Investigator of Record:**	*(insert name)*
**Research Site Address(es):**	*(insert address)*
**Daytime telephone number(s):**	*(insert number)*
**24-hour contact number(s):**	*(insert number)*

Subject Information and Consent Form
Please ask the study investigator or the study staff to explain any words or procedures that you do not clearly understand.

The purpose of this form is to give you information about the research study you are being asked to join. If you sign this form, you will be giving your permission to take part in the study. The form describes the purpose, procedures, benefits, and risks of the research study. You should take part in the study only if you want to do so. You may choose not to join the research project or withdraw from this study at any time. Choosing not to take part in this research will not in any way affect the health care or benefits that you or your family will receive. Please read this Subject Information and Consent Form and ask as many questions as needed. You should not sign this form if you have any questions that have not been answered to your satisfaction.

This study is being funded by the U.S. National Institutes of Health, the Office of the United States Global AIDS Coordinator, and the Bill and Melinda Gates Foundation

Your participation is voluntary
You do not have to take part in this study. If you decide today to take part in this research project, you may refuse to take part in any portion of the study or stop at any time without reducing or affecting any care that you receive at the health centers in your community.

Purpose of the Research in the Communities
The HPTN 071 or PopART study is testing a program to try to reduce HIV infection in a community like yours. Twenty one communities that include about 600,000 adults are included in this research (about 400,000 adults in twelve Zambian communities and 200,000 adults in nine South African communities).

In some communities, the level of care that people are used to will stay the same, in terms of HIV testing, and care of those who have HIV.

In other communities, to make HIV testing easier, community health care workers will go to all homes and will offer to do an HIV test on each adult (or younger people with permission of guardian) wishing to have a test. For anyone infected with HIV, they will be offered to start taking drugs to treat HIV according to the standard treatment guidelines that are in place for doing so in your country. The health workers will visit every home again once a year for up to three more years to repeat the HIV testing and to refer people to care.

In other communities, health care workers will go to all houses offering HIV testing, as was just described. In these communities if someone tests HIV positive however, they will be offered to start taking medicines to treat HIV right away. The health workers will visit every home again once a year for up to three more years to repeat the HIV testing and to refer people to care.

At the end of the study, the researchers will see if offering HIV tests in each household and offering people the chance to start HIV treatment right away has reduced HIV infection.

Your community is one of the communities participating in this research. If health care workers are visiting homes in your community, you will notice that they provide some other information and services to people, but the most important thing is the testing and HIV treatment they offer.

In each community, around 2,700 people will be asked to participate in additional activities such as completing questionnaires and providing additional samples for laboratory testing. These questionnaires and tests will let the researchers understand how the community feels about the program and if the program is working. You have been selected to be one of the people from your community who we are asking to participate in these additional activities. That is why you are being asked to read this document.

What will happen during this study?
You will have one study visit which will occur today. This visit will take approximately 1 hour. During this visit, a researcher will ask you questions about sexual behavior, health services, previous HIV testing, HIV-related stigma and other HIV-related questions.

What are the possible risks or discomforts?
The risk to you in participating in this study is that some of the questions may be uncomfortable and may make you feel worried or embarrassed. If any of the questions make you feel upset, the interviewer may go to another question or totally stop the interview.

What are the potential benefits?
You will not receive any direct benefit from being in this study. You or others may benefit in the future from the information learned in this study.

Are there any alternatives to participation?
If you decide not to participate in this study, you can still receive HIV tests and other services from your local health center.

How will my confidentiality and privacy be protected?
We cannot guarantee absolute confidentiality. However, we will do everything possible to protect your confidentiality if you join this study. We do this by giving you a study number and any information you provide will be labeled with this number only, not your name. Only the research staff will be able to link this number to your name. Your personal information (name, address, phone number) will be protected by the research clinic. This information will not be used in any publication of information about this study.

To protect your privacy, you will meet with the researcher in a private area where others cannot overhear conversations with you.

People who may review your records include: [insert name of site IRB/EC], local regulatory agencies, US National Institutes of Health (NIH), study staff, and study monitors. Institutional Review Boards (IRBs) or Ethics Committees (ECs) are committees that watch over the safety and rights of research participants.

What happens if I am injured by participating in this study?
It is very unlikely that you could be injured as a result of participating in this study. However, if you are injured while participating in this study, you will be given immediate treatment for your injuries. You [will/will not] have to pay for this care. There [is a/is no] program for compensation either through this institution or the United States NIH. You will not be giving up any of your legal rights by signing this Subject Information and Consent Form.

What are some reasons why I may be withdrawn from this activity without my consent?
You may be withdrawn from the study without your consent for the following reasons:

- The research study, or this part of the study, is stopped or canceled
- The study staff feels that completing the study or this part of the study would be harmful to you or others

Persons to Contact for Problems or Questions
If you have any questions about your participation in this research study, your rights as a research subject, or if you feel that you have experienced a research-related injury, contact:

Investigator of Record Name: *(site insert name of the investigator or other study staff)*

Research Site Address(es): *(site insert physical address of above)*

Daytime telephone number(s): *(site insert telephone number)*

24-hour contact number(s): *(site insert telephone number)*

If you have any questions or concerns about your rights as a research subject or want to discuss a problem, get information or offer input, you may contact:

Independent Review Board/Ethics Committee: *(site insert name or title of person on the IRB/EC or other organization appropriate for the site)*

Address of Independent Review Board: *(site insert physical address of above)*

Daytime Telephone Number: *(site insert telephone number of above)*

SUBJECT'S STATEMENT OF CONSENT

Population Effects of Antiretroviral Therapy to Reduce HIV Transmission (PopART): A cluster-randomized trial of the impact of a combination prevention package on population-level HIV incidence in Zambia and South Africa

- I have been given sufficient time to consider whether to take part in this study.
- My taking part in this research study is voluntary. I may decide not to take part or to withdraw from the research study at any time without penalty or loss of benefits or treatment to which I am entitled.
- The research study may be stopped at any time without my consent.
- I have had an opportunity to ask my study investigator questions about this research study. My questions so far have been answered to my satisfaction.
- I have been told how long I may be in the research study.
- I have been informed of the procedures and tests that may be performed during the research study.
- I have been told what the possible risks and benefits are from taking part in this research study. I may not benefit if I take part in this research study.
- I do not give up my legal rights by signing this form.
- I have been told that before any study related procedures being performed, I will be asked to voluntarily sign this Subject Information and Consent Form.
- I will receive a signed and dated copy of this Subject Information and Consent Form.

If you have either read or have heard the information in this Subject Information and Consent Form, if all of your questions have been answered, and if you agree to take part in the study, please print and sign and your name and write the date on the line below.

I voluntarily agree to take part in this research study.

___________________________	____
Subject's Name (print)	Subject's Signature and Date

I certify that the information provided was given in a language that was understandable to the subject.

___________________________	____
Name of Study Staff<br>Conducting Consent Discussion (print)	Study Staff Signature and Date

___________________________	____
Witness' Name (print)<br>(As appropriate) Date	Witness' Signature and Date

APPENDIX V - SAMPLE INFORMED CONSENT FORM – ARM A PARTICIPANTS STARTING ART IMMEDIATELY

SUBJECT INFORMATION AND CONSENT FORM

**Title of Research Study:**	**Population Effects of Antiretroviral Therapy to Reduce HIV Transmission (PopART): A cluster-randomized trial of the impact of a combination prevention package on population-level HIV incidence in Zambia and South Africa**
**Protocol #:**	HPTN 071, Version 1.0, 26 October 2012<br>DAIDS ID: 11865
**Sponsor:**	National Institute of Allergy and Infectious Diseases<br>National Institute of Mental Health<br>(U.S. National Institutes of Health)<br>Office of the United States Global AIDS Coordinator<br>Bill and Melinda Gates Foundation
**Investigator of Record:**	*(insert name)*
**Research Site Address(es):**	*(insert address)*
**Daytime telephone number(s):**	*(insert number)*
**24-hour contact number(s):**	*(insert number)*

Subject Information and Consent Form
Please ask the study investigator or the study staff to explain any words or procedures that you do not clearly understand.

The purpose of this form is to give you information about the research study you are being asked to join. If you sign this form, you will be giving your permission to take part in the study. The form describes the purpose, procedures, benefits, and risks of the research study. You should take part in the study only if you want to do so. You may choose not to join the research project or withdraw from this study at any time. Choosing not to take part in this research will not in any way affect the health care or benefits that you or your family will receive. Please read this Subject Information and Consent Form and ask as many questions as needed. You should not sign this form if you have any questions that have not been answered to your satisfaction.

This study is being funded by the U.S. National Institutes of Health, the Office of the United States Global AIDS Coordinator, and the Bill and Melinda Gates Foundation.

Your participation is voluntary
You do not have to take part in this study. If you decide today to take part in this research project, you may refuse to take part in any portion of the study or stop at any time without reducing or affecting any care that you receive at the clinics in your community.

Purpose of the Research in the Communities
The HPTN 071 or PopART study is testing a program to try to reduce HIV infection in a community like yours. Twenty one communities that include about 600,000 adults are included in this research (about 400,000 adults in twelve Zambian communities and 200,000 adults in nine South African communities).

In some communities, the level of care that people are used to will stay the same, in terms of HIV testing, and care of those who have HIV.

In other communities, to make HIV testing easier, community health care workers will go to all homes and will offer to do an HIV test on each adult (or younger people with permission of guardian) wishing to have a test. For anyone infected with HIV, they will be offered to start taking drugs to treat HIV according to the standard treatment guidelines that are in place for doing so in your country. The health workers will visit every home again once a year for up to three more years to repeat the HIV testing and to refer people to care.

In some communities, **including this community**, health care workers will go to all houses offering HIV testing, as was just described. **In these communities if someone tests HIV positive however, they will be offered to start taking medicines to treat HIV right away**. The health workers will visit every home again once a year for up to three more years to repeat the HIV testing and to refer people to care.

At the end of the study, the researchers will see if offering HIV tests in each household and offering people the chance to start HIV treatment right away has reduced HIV infection.

What will happen during this study?
If you agree to participate in this study, you will start taking anti-HIV drugs immediately. National guidelines suggest starting people on anti-HIV drugs when their immune cells, called CD4 cells, drop below a certain level. We would like to know if starting all HIV positive individuals on anti-HIV drugs as soon as they are diagnosed with HIV helps reduce the spread of HIV in their community.

Your clinic visit schedule and routine health testing will occur according to the local standards at this clinic.

What are the possible risks or discomforts?
Anti-HIV Drugs:
There are many drugs available to treat HIV and AIDS. The doctors in the clinic will determine the best combination of these drugs to treat you. It is possible that the drugs may make you feel sick or will affect your blood tests, in which case the doctors may either switch you to different drugs, or stop them all together. It is very important for you to return to the clinic whenever you feel sick. Feeling sick may be due to the pills or it may be due to a sickness caused by your HIV

infection or it may be caused by something completely different, such as malaria. Either way, you should return to the clinic so that you can be treated.

As with any medication, anti-HIV drugs can cause side effects. Most of the medicines for HIV are very safe and are well tolerated with only very few side effects. Some of these side effects are mild and may go away after you have taken the drugs for a few weeks. Examples of these types of side effects include upset stomach, vomiting, headache, and changes in your mood, sleep, or concentration. Other side effects can be severe but are rare and may require treatment or hospitalization. Examples of these types of side effects include rash, liver problems, severe depression or psychosis, and pancreas problems.

If you take your anti-HIV medicines very regularly they will work and keep the amount of virus in your body low. If for any reason you do not keep taking the medicines every day, the amount of virus in your body can increase and the anti-HIV pills you are taking may stop working against the virus (the virus becomes resistant), and your doctors will have fewer medicines to choose from to try to keep you healthy. If that happens, the doctors will try to give you different drugs that will work.

A doctor will explain all of the possible side-effects of any drugs before you begin taking them.

There is a risk of serious and life-threatening side effects when other drugs are taken with anti-HIV medications. For your safety, you must tell your doctor about all medications you are taking before you start taking anti-HIV medications.

<u>Risks Associated with Early versus Delayed Treatment with Anti-HIV Drugs</u>:
If you agree, you will begin taking anti-HIV drugs immediately. If you begin the drugs immediately, there is a chance that when you start taking the medicines, especially at the beginning, the drugs may make you feel sick. As with any medication some drugs may have side effects so severe that the nurse or doctor may need to take you off that drug and give you another. It is important that the medical teams check that the drugs you start taking are safe for you and change the drugs if they are not.
It is really important that once you start taking anti-HIV medicines, you try to take them every single day and do not miss doses, share tablets with other people or suddenly stop them. If you take the tablets but only very irregularly then there is a chance that they will no longer work against your virus and the virus will becomes ‘resistant’ to the medicine. If this happens it limits the choices for other treatment and if it carries on there may be no medicines that can work to suppress your virus when you become sick.

What are the potential benefits?
At the moment, the national guidelines inform medical teams when it is the best time to start ART and this is decided based on a measure of your immune system (CD4 count). Doctors and researchers are always trying to find better ways to keep people healthy and it is possible that starting ART earlier may be better for your health but at the moment this is uncertain, which is why medical research is being done.

In addition to providing health benefits for you, taking ART to suppress the HIV virus has been shown to reduce the risk of passing on HIV to sexual partners or babies. Also starting treatment early may help prevent Tuberculosis which occurs more often in people who are HIV positive.

There is no cure for HIV and no method is 100% effective in preventing the spread of HIV, except abstinence. ART does not protect you from getting other infections that can be passed on through unprotected sex, so it is important to continue using condoms correctly during every sex act.

Are there any alternatives to participation?
If you do not agree to take anti-HIV drugs at this time, you can still be seen here at the health clinic for HIV care and you will be offered treatment according to the national guidelines.

How will my confidentiality and privacy be protected?
We cannot guarantee absolute confidentiality. However, we will do everything possible to protect your confidentiality if you join this study. We do this by giving you a study number and any information will be labeled with this number only, so people working in the clinics and laboratories will only see a number not your name, only the research staff will be able to link this number to your name. Your personal information (name, address, phone number) will be protected by the research clinic. This information will not be used in any publication of information about this study.

To protect your privacy, you will meet with the researcher in a private area where others cannot overhear conversations with you.

People who may review your records include: [insert name of site IRB/EC], local regulatory agencies, US National Institutes of Health (NIH), study staff, and study monitors. Institutional Review Boards (IRBs) or Ethics Committees (ECs) are committees that watch over the safety and rights of research participants.

What happens if I am injured by participating in this study?
It is very unlikely that you could be injured as a result of participating in this study. However, if you are injured while participating in this study, you will be given immediate treatment for your injuries. You [will/will not] have to pay for this care. There [is a/is no] program for compensation either through this institution or the United States NIH. You will not be giving up any of your legal rights by signing this Subject Information and Consent Form.

What are some reasons why I may be withdrawn from this activity without my consent?
You may be withdrawn from the study without your consent for the following reasons:

- The research study, or this part of the study, is stopped or canceled
- The study staff feels that completing the study or this part of the study would be harmful to you or others

Persons to Contact for Problems or Questions
If you have any questions about your participation in this research study, your rights as a research subject, or if you feel that you have experienced a research-related injury, contact:

Investigator of Record Name: *(site insert name of the investigator or other study staff)*

Research Site Address(es): *(site insert physical address of above)*

Daytime telephone number(s): *(site insert telephone number)*

24-hour contact number(s): *(site insert telephone number)*

If you have any questions or concerns about your rights as a research subject or want to discuss a problem, get information or offer input, you may contact:

Independent Review Board/Ethics Committee: *(site insert name or title of person on the IRB/EC or other organization appropriate for the site)*

Address of Independent Review Board: *(site insert physical address of above)*

Daytime Telephone Number: *(site insert telephone number of above)*

SUBJECT'S STATEMENT OF CONSENT

Population Effects of Antiretroviral Therapy to Reduce HIV Transmission (PopART): A cluster-randomized trial of the impact of a combination prevention package on population-level HIV incidence in Zambia and South Africa

- I have been given sufficient time to consider whether to take part in this study.
- My taking part in this research study is voluntary. I may decide not to take part or to withdraw from the research study at any time without penalty or loss of benefits or treatment to which I am entitled.
- The research study may be stopped at any time without my consent.
- I have had an opportunity to ask my study investigator questions about this research study. My questions so far have been answered to my satisfaction.
- I have been told how long I may be in the research study.
- I have been informed of the procedures and tests that may be performed during the research study.
- I have been told what the possible risks and benefits are from taking part in this research study. I may not benefit if I take part in this research study.
- I do not give up my legal rights by signing this form.
- I have been told that before any study related procedures being performed, I will be asked to voluntarily sign this Subject Information and Consent Form.
- I will receive a signed and dated copy of this Subject Information and Consent Form.

If you have either read or have heard the information in this Subject Information and Consent Form, if all of your questions have been answered, and if you agree to take part in the study, please print and sign and your name and write the date on the line below.

I voluntarily agree to take part in this research study.

___________________________	___________________________
Subject's Name (print)	Subject's Signature and Date

I certify that the information provided was given in a language that was understandable to the subject.

___________________________	___________________________
Name of Study Staff<br>Conducting Consent Discussion (print)	Study Staff Signature and Date
___________________________	___________________________
Witness' Name (print)<br>(As appropriate) Date	Witness' Signature and Date

APPENDIX VI - SAMPLE INFORMED CONSENT FORM – CHIP TEAM ACTIVITIES

SUBJECT INFORMATION SHEET

**Title of Research Study:**	**Population Effects of Antiretroviral Therapy to Reduce HIV Transmission (PopART): A cluster-randomized trial of the impact of a combination prevention package on population-level HIV incidence in Zambia and South Africa**
**Protocol #:**	HPTN 071, Version 1.0, 26 October 2012<br>DAIDS ID: 11865
**Sponsor:**	National Institute of Allergy and Infectious Diseases<br>National Institute of Mental Health<br>(U.S. National Institutes of Health)<br>Office of the United States Global AIDS Coordinator<br>Bill and Melinda Gates Foundation
**Investigator of Record:**	*(insert name)*
**Research Site Address(es):**	*(insert address)*
**Daytime telephone number(s):**	*(insert number)*
**24-hour contact number(s):**	*(insert number)*

What is the PopART study?

- HIV is still a big problem in Zambia and South Africa
- We now have good treatment (called ART) for people living with HIV which is freely available from health facilities.
- There are various methods that are known to help prevent someone from catching HIV such as using condoms, male circumcision and prevention of mother to child transmission (PMTCT) programs
- Getting people who are HIV positive onto treatment earlier may also help to prevent them from infecting their partners.
- It has been suggested that combining these HIV prevention strategies and offering ART to all people who test HIV positive right away might lead to a big reduction in the number of new HIV infections in the community.

The PopART study will try to answer the above question. Its purpose is to find out if **offering HIV tests in each household** and offering people the chance to **start HIV treatment right away** can reduce HIV infection in a community like yours.

Where and who is conducting this study?

This study is being carried out in two countries, Zambia and South Africa, for a period of about 5 years from 2012 to 2017. It will be done in 21 communities, 12 of which are in Zambia and 9 in South Africa. Researchers from the Zambia AIDS Related Tuberculosis (ZAMBART) Project and the Desmond Tutu TB Centre (DTTC) at Stellenbosch University, South Africa, will work closely together with colleagues from different institutions including the Ministry of Health (Zambia) and the Department of Health (South Africa). This study is being funded by the U.S. National Institutes of Health, the Office of the United States Global AIDS Coordinator, and the Bill and Melinda Gates Foundation.

How is the study being carried out?

- The PopART study has 3 Arms (Arms A, B and C). In each arm a package of HIV prevention services will be available including HIV testing, care and treatment, male circumcision, PMTCT and condoms:
    - In Arm C all of these activities will be available at the health facility. In arms A and B, community HIV workers (called CHiPs) will visit each household and offer HIV testing in the home and help people to link to care services at the health facility or in the community.
    - In all arms of the study, people who are HIV-positive will receive ART from the health facility. In Arms B and C this will be for all people who have a CD4 count <350 as per national guidelines. In Arm A, ALL people who are HIV-positive will be offered ART at any CD4 count.
    - In Arms A and B, the CHiP teams will encourage pregnant women who are met during regular household visits, to attend an antenatal clinic in their community.
- The 21 study communities were put in these arms using a process called randomization, which is like a lottery. **Your community was put in Arm [*A/B*].**

The CHiPs worker is your link to all of these services. If you agree, they will visit your house regularly. They can also be contacted at any time if you have specific questions or need help with accessing care.

How will the researchers find out if the program worked?

To find out if the PopART program work in reducing the number of HIV infections, some people in the community will be asked to take part in some special studies. If you are chosen for one of these studies, you will be asked to choose whether or not to take part in that study. But right now, we are only asking if you are willing to take part in the CHiPs program.

What is required of people who agree to take part?

If you agree for the CHiPs to visit your house to provide these services:

- The CHiPs will take down the names and basic information of all household members. This is to ensure that the CHiPs do not miss some members of the household now or in future.

- The CHiPs will ask household members to participate in an education session in their own house. Education sessions may be done individually, for couples or in a group according to your choice.
- The CHiPs will ask individuals to consider having an HIV test. Individuals do not have to have a test unless they wish to do so. Household members aged below 16 can only join the study if their parents or guardians give their consent.
- Individuals who do not want to test for HIV can still choose to receive other services that have been mentioned earlier.
- The CHiPs will encourage and assist people to access care at the health facility and also give information about where male circumcision is provided in this community.
- The information collected by the CHiPs team will link to the health facility information so that it easier to follow up people. The information collected will all be kept confidential but will be used by the CHiPs and health care workers to ensure that you receive the best care.
- Taking part in this CHiPs service is voluntary and therefore individuals are completely free to refuse to take part.

CHiP teams will follow national requirements to notify local health authorities when a TB case is identified.

Risks and Benefits

There are unlikely to be additional risks other than those associated with HIV testing, care and treatment.

Both HIV positive and negative individuals will benefit from the linkages to care provided by the CHiPs program. In addition taking ART reduces the likelihood that HIV will be passed on to a sexual partner or baby.

Persons to Contact for Problems or Questions
If you have any questions about this research study, your rights, or if you feel that you have experienced a research-related injury, contact:

Investigator of Record Name: *(site insert name of the investigator or other study staff)*

Research Site Address(es): *(site insert physical address of above)*

Daytime telephone number(s): *(site insert telephone number)*

24-hour contact number(s): *(site insert telephone number)*

If you have any questions or concerns about your rights or want to discuss a problem, get information or offer input, you may contact:

Independent Review Board/Ethics Committee: *(site insert name or title of person on the IRB/EC or other organization appropriate for the site)*

Address of Independent Review Board: *(site insert physical address of above)*

Daytime Telephone Number: *(site insert telephone number of above)*

Verbal Consent Administered by CHiPs

As you have heard from the information leaflet you have just read/ I have just read to you, I am one of the CHiPs working with the PoPART study in this community. Now I would like to find out if you have understood this information and if you would like to take part in this CHiPs program.

[*CHiP records in a log the decision by the participant(s)*]

APPENDIX VII – SAMPLE SIZE CALCULATIONS

(1) Primary endpoint - HIV incidence over 36 months

N=2500 individuals in population cohort, 85% HIV-negative at baseline, 25% loss to follow-up by 36 months; 5206 person-years per community over 36 months (assuming 1912 person-years 0-12 months; 1700 person-years 12-24 months; 1594 person-years 24-36 months)

(a) Comparison between Arms A or B and Arm C

HIV incidence rate/ 100py (control arm)	Between-cluster coefficient of variation (k)	Effectiveness (%)	Power (%)
1.0	0.15	30%	74%
1.0	0.15	35%	87%
1.0	0.15	40%	95%
1.0	0.15	45%	99%
1.0	0.15	50%	100%
1.0	0.15	55%	100%
1.0	0.15	60%	100%
1.0	0.15	65%	100%
1.0	0.20	30%	60%
1.0	0.20	35%	75%
1.0	0.20	40%	87%
1.0	0.20	45%	94%
1.0	0.20	50%	98%
1.0	0.20	55%	99%
1.0	0.20	60%	100%
1.0	0.20	65%	100%
1.5	0.15	30%	81%
1.5	0.15	35%	92%
1.5	0.15	40%	98%
1.5	0.15	45%	100%
1.5	0.15	50%	100%
1.5	0.15	55%	100%
1.5	0.15	60%	100%
1.5	0.15	65%	100%
1.5	0.20	30%	65%
1.5	0.20	35%	80%
1.5	0.20	40%	91%
1.5	0.20	45%	96%
1.5	0.20	50%	99%
1.5	0.20	55%	100%
1.5	0.20	60%	100%
1.5	0.20	65%	100%

(b) Comparison between Arms A and B

HIV incidence rate/ 100py (control arm)	Between-cluster coefficient of variation (k)	Effectiveness (%) Arm A	Effectiveness (%) Arm B	Power (%)
1.0	0.15	50%	20%	89%
1.0	0.15	50%	25%	78%
1.0	0.15	**55%**	**25%**	**92%**
1.0	0.15	55%	30%	82%
1.0	0.15	**60%**	**25%**	**98%**
1.0	0.15	**60%**	**30%**	**94%**
1.0	0.20	50%	20%	78%
1.0	0.20	50%	25%	65%
1.0	0.20	**55%**	**25%**	**83%**
1.0	0.20	55%	30%	71%
1.0	0.20	**60%**	**25%**	**93%**
1.0	0.20	**60%**	**30%**	**87%**
1.5	0.15	50%	20%	94%
1.5	0.15	50%	25%	86%
1.5	0.15	**55%**	**25%**	**96%**
1.5	0.15	55%	30%	90%
1.5	0.15	**60%**	**25%**	**99%**
1.5	0.15	**60%**	**30%**	**98%**
1.5	0.20	50%	20%	84%
1.5	0.20	50%	25%	72%
1.5	0.20	**55%**	**25%**	**88%**
1.5	0.20	55%	30%	78%
1.5	0.20	**60%**	**25%**	**96%**
1.5	0.20	**60%**	**30%**	**92%**

(2A) HIV incidence during months 12-24 from start of intervention

Number of person-years of follow-up in each community = 1700 during months 12-24 from start of intervention

(a) Comparison between Arms A or B and Arm C

HIV incidence rate/ 100py (control arm)	Between-cluster coefficient of variation (k)	Effectiveness (%)	Power (%)
1.0	0.15	35%	59%
1.0	0.15	40%	72%
1.0	0.15	45%	83%
1.0	0.15	50%	91%
1.0	0.15	55%	96%
1.0	0.15	60%	98%
1.0	0.15	65%	99%
1.0	0.15	70%	100%
1.0	0.20	35%	51%
1.0	0.20	40%	64%
1.0	0.20	45%	76%
1.0	0.20	50%	85%
1.0	0.20	55%	92%
1.0	0.20	60%	96%
1.0	0.20	65%	99%
1.0	0.20	70%	100%
1.5	0.15	35%	71%
1.5	0.15	40%	83%
1.5	0.15	45%	92%
1.5	0.15	50%	97%
1.5	0.15	55%	99%
1.5	0.15	60%	100%
1.5	0.15	65%	100%
1.5	0.15	70%	100%
1.5	0.20	35%	61%
1.5	0.20	40%	74%
1.5	0.20	45%	85%
1.5	0.20	50%	92%
1.5	0.20	55%	97%
1.5	0.20	60%	99%
1.5	0.20	65%	100%
1.5	0.20	70%	100%

(b) Comparison between Arms A and B

HIV incidence rate/ 100py (control arm)	Between-cluster coefficient of variation (k)	Effectiveness (%) Arm A	Effectiveness (%) Arm B	Power (%)
1.0	0.15	60%	25%	77%
1.0	0.15	60%	30%	66%
1.0	0.15	65%	30%	81%
1.0	0.15	65%	35%	71%
1.0	0.15	70%	30%	92%
1.0	0.15	70%	35%	85%
1.0	0.20	60%	25%	71%
1.0	0.20	60%	30%	61%
1.0	0.20	65%	30%	76%
1.0	0.20	65%	35%	66%
1.0	0.20	70%	30%	88%
1.0	0.20	70%	35%	81%
1.5	0.15	60%	25%	89%
1.5	0.15	60%	30%	80%
1.5	0.15	65%	30%	92%
1.5	0.15	65%	35%	84%
1.5	0.15	70%	30%	98%
1.5	0.15	70%	35%	94%
1.5	0.20	60%	25%	82%
1.5	0.20	60%	30%	72%
1.5	0.20	65%	30%	86%
1.5	0.20	65%	35%	78%
1.5	0.20	70%	30%	95%
1.5	0.20	70%	35%	90%

(2B) HIV incidence during months 24-36 from start of intervention

Number of person-years of follow-up in each community = 1594 during months 24-36 from start of intervention

(a) Comparison between Arms A or B and Arm C

HIV incidence rate/ 100py (control arm)	Between-cluster coefficient of variation (k)	Effectiveness (%)	Power (%)
1.0	0.15	35%	57%
1.0	0.15	40%	70%
1.0	0.15	45%	81%
1.0	0.15	50%	89%
1.0	0.15	55%	95%
1.0	0.15	60%	98%
1.0	0.15	65%	99%
1.0	0.15	70%	100%
1.0	0.20	35%	50%
1.0	0.20	40%	62%
1.0	0.20	45%	74%
1.0	0.20	50%	84%
1.0	0.20	55%	91%
1.0	0.20	60%	96%
1.0	0.20	65%	98%
1.0	0.20	70%	99%
1.5	0.15	35%	69%
1.5	0.15	40%	82%
1.5	0.15	45%	91%
1.5	0.15	50%	96%
1.5	0.15	55%	99%
1.5	0.15	60%	100%
1.5	0.15	65%	100%
1.5	0.15	70%	100%
1.5	0.20	35%	60%
1.5	0.20	40%	73%
1.5	0.20	45%	84%
1.5	0.20	50%	92%
1.5	0.20	55%	96%
1.5	0.20	60%	99%
1.5	0.20	65%	100%
1.5	0.20	70%	100%

(b) Comparison between Arms A and B

HIV incidence rate/ 100py (control arm)	Between-cluster coefficient of variation (k)	Effectiveness (%) Arm A	Effectiveness (%) Arm B	Power (%)
1.0	0.15	60%	25%	75%
1.0	0.15	60%	30%	64%
1.0	0.15	65%	30%	79%
1.0	0.15	65%	35%	69%
1.0	0.15	70%	30%	90%
1.0	0.15	70%	35%	84%
1.0	0.20	60%	25%	69%
1.0	0.20	60%	30%	59%
1.0	0.20	65%	30%	74%
1.0	0.20	65%	35%	64%
1.0	0.20	70%	30%	86%
1.0	0.20	70%	35%	79%
1.5	0.15	60%	25%	87%
1.5	0.15	60%	30%	78%
1.5	0.15	65%	30%	90%
1.5	0.15	65%	35%	82%
1.5	0.15	70%	30%	97%
1.5	0.15	70%	35%	93%
1.5	0.20	60%	25%	81%
1.5	0.20	60%	30%	71%
1.5	0.20	65%	30%	85%
1.5	0.20	65%	35%	76%
1.5	0.20	70%	30%	94%
1.5	0.20	70%	35%	89%

(3A) Community viral load 24 months after start of intervention

N=300 HIV-positive individuals in each community

(a) Comparison between Arms A or B and Arm C

Percentage with undetectable viral load (control arm)	Between-cluster coefficient of variation (k)	Percentage with undetectable viral load, Arm A or B	Power (%)
20%	0.15	40%	99%
20%	0.15	60%	99%
20%	0.20	40%	99%
20%	0.20	60%	99%

(b) Comparison between Arms A and B

Between-cluster coefficient of variation (k)	Percentage with undetectable viral load, Arm A	Percentage with undetectable viral load, Arm B	Power (%)
0.15	60%	40%	97%
0.20	60%	40%	85%

(3B) Community viral load 12 and 36 months after start of intervention

N=75 HIV-positive individuals in each community

(a) Comparison between Arms A or B and Arm C

Percentage with undetectable viral load (control arm)	Between-cluster coefficient of variation (k)	Percentage with undetectable viral load, Arm A or B	Power (%)
20%	0.15	40%	99%
20%	0.15	60%	99%
20%	0.20	40%	97%
20%	0.20	60%	99%

(b) Comparison between Arms A and B

Between-cluster coefficient of variation (k)	Percentage with undetectable viral load, Arm A	Percentage with undetectable viral load, Arm B	Power (%)
0.15	60%	40%	91%
0.20	60%	40%	77%

(4) HSV2 incidence over 36 months

Number of person-years of follow-up in each community = 1837 over 36 months

Comparison between Arms A or B and Arm C

HSV2 incidence rate/ 100py (control arm)	Between-cluster coefficient of variation (k)	HSV2 incidence rate / 100py, Arm A or Arm B	Power (%)
5.0	0.15	3.0	98%
5.0	0.15	7.5	94%
5.0	0.20	3.0	92%
5.0	0.20	7.5	81%

(5) Retention in HIV care, and viral load suppression and drug resistance among HIV-positive individuals who are taking ART – measured among HIV-positive members of the Population Cohort

(i) Retention in HIV care 12 months after registering for HIV care

N=198 in each community in Arm A and B; N=99 in each community in Arm C

This assumes: N=375 HIV-positive individuals per community in the population cohort; that 65% of these individuals are not yet registered at the clinic for HIV care (N=244); that among these 244 individuals, in Arms A and B 90% subsequently register at the clinic for HIV care (N=220) and in Arm C 45% subsequently register at the clinic for HIV care (N=110), and that 10% cannot be included in analysis due to migration out of the community, giving N=198 included in analysis in Arms A and B and N=99 included in analysis in Arm C

Comparison between Arm A or Arm B, with Arm C

Percentage retained in care (control arm)	Between-cluster coefficient of variation (k)	Percentage retained in care, Arm A or Arm B	Power (%)
80%	0.20	70%	71%
80%	0.20	90%	94%
85%	0.20	75%	85%
85%	0.20	95%	99%
90%	0.20	80%	96%
90%	0.20	95%	79%

(ii) Viral load suppression and drug resistance, measured among HIV-positive members of the Population Cohort at 24 months

Calculations assume that, among population cohort members, N=220 HIV-positive individuals per community register for HIV care for the first time in Arms A and B and N=110 in Arm C, as above.

It is further assumed that, by the time of the 24-month follow-up in the population cohort, 67% of such patients will have started ART in Arm C, 50% in Arm B, and 80% in Arm A; and that 80% of such patients will participate in the Population Cohort at 24 months (PC24). This gives N=141, N=88, and N=59 per community in Arms A, B, and C respectively, for viral load and drug resistance measurement at PC24.

(a) Comparison between Arm A and Arm C

Percentage with detectable viral load (control arm)	Between-cluster coefficient of variation (k)	Percentage with detectable viral load, Arm A	Power (%)
5%	0.20	10%	72%
5%	0.20	2.5%	39%
10%	0.20	20%	91%
10%	0.20	15%	45%
10%	0.20	5%	63%

(b) Comparison between Arm B and Arm C

Percentage with detectable viral load (control arm)	Between-cluster coefficient of variation (k)	Percentage with detectable viral load, Arm B	Power (%)
5%	0.20	10%	64%
5%	0.20	2.5%	36%
10%	0.20	20%	86%
10%	0.20	15%	40%
10%	0.20	5%	60%

(6) Prevalence of bacteriologically-confirmed pulmonary tuberculosis 36 months after start of intervention

Calculations assume 4250 adults included in TB prevalence survey in each community

Pulmonary TB prevalence (control arm)	Between-cluster coefficient of variation (k)	Effectiveness (%)	Pulmonary TB prevalence (Arm A, or Arm B)	Power (%)
1.00%	0.25	40%	0.60%	73%
1.00%	0.25	45%	0.55%	84%
1.00%	0.25	50%	0.50%	91%
0.80%	0.25	40%	0.48%	69%
0.80%	0.25	45%	0.44%	81%
0.80%	0.25	50%	0.40%	89%

(7) HIV-free child survival among children born during the 36 months of trial intervention

Calculations assume 229 person-years of follow-up on HIV-free child survival, among children born to HIV-positive mothers, in each community

Comparison between Arm A or Arm B, with Arm C

Rate of child mortality and/or HIV infection per 100py, among children born to HIV-positive mother (control arm)	Between-cluster coefficient of variation (k)	Effectiveness (%)	Power (%)
11	0.15	35%	71%
11	0.15	40%	83%
11	0.15	45%	92%
11	0.15	50%	97%
11	0.20	35%	61%
11	0.20	40%	74%
11	0.20	45%	85%
11	0.20	50%	92%
10	0.15	35%	68%
10	0.15	40%	81%
10	0.15	45%	90%
10	0.15	50%	96%
10	0.20	35%	59%
10	0.20	40%	72%
10	0.20	45%	83%
10	0.20	50%	91%
9	0.15	35%	65%
9	0.15	40%	78%
9	0.15	45%	88%
9	0.15	50%	94%
9	0.20	35%	56%
9	0.20	40%	69%
9	0.20	45%	81%
9	0.20	50%	89%

APPENDIX VIII - POPULATION CROSS-SECTIONAL SURVEY

Because participants in the *Population Cohort* will be followed longitudinally over three years, their interactions with the research staff could bias the data they provide for certain outcome measures. The *Population Cross-Sectional Survey*, if funded, would be a snapshot evaluation to provide unbiased data for comparison on many of the measures evaluated in *the Population Cohort*.

Analyses for process measures, secondary outcome measures, and the Schedule of Study Visits and Procedures planned for the Population Cross-Sectional Survey are provided below.

Statistical Analysis of Process Measures in the *Population Cross-Sectional Survey* at 36 Months (Arms A, B, and C)

With a sample size of 500 adults aged 18-44 in the *Population Cross-Sectional Survey* in each community, estimates will be obtained for each trial arm of (i) the percentage of the adult population who have accessed HIV counseling and testing (HCT) services during the 36 months of trial intervention (ii) the percentage of HIV-infected individuals who have been screened for ART eligibility during the 36 months of trial intervention (iii) the percentage of HIV-infected individuals who are on ART at the time of the cross-sectional survey, and (iv) the percentage of initially uncircumcised men who have had medical male circumcision during the 36 months of trial intervention.

(i) HIV testing uptake

Assuming that the uptake of HIV testing during the past 36 months is 50% in Arm C, compared with 70% in each of Arm A and Arm B, and that k=0.2, there is 73% power to show an effect of the CHiP intervention on testing uptake. With higher testing uptake of 80% in each of Arm A and Arm B, there is 94% power to show an effect of the CHiP intervention.

(ii) Screening for ART eligibility, and uptake of ART, among HIV-infected individuals

On average there will be 75 HIV-infected individuals (15% of 500) included in the *Population Cross-Sectional Survey* in each community. Assuming that 25% were already on ART at the start of the trial, on average 56 will have been ART-naïve at the start of the trial. With the percentage screened for ART eligibility 70% or higher in Arm B, and 40% or lower in Arm C, and k=0.2, study power is at least 95% to show an effect of the trial intervention on the uptake of ART eligibility screening. Similarly, with the percentage started on ART 70% or higher in Arm A, and 40% or lower in Arm C, and k=0.2, study power is at least 95% to show an effect of the trial intervention on ART uptake.

(iii) Male circumcision

On average there will be 250 men in the *Population Cross-Sectional Survey*. In the Western Cape trial communities, approximately 49 will be HIV-uninfected and not circumcised prior to the start of the PopART trial and approximately 185 in the Zambian trial communities, giving a harmonic mean of 91 in each community. With the uptake of medical male circumcision 40% or more in each of Arms A and B, but 25% or less in Arm C, and k=0.2, study power is at least 82% to show an effect of the trial intervention.

Outcomes for Secondary Objectives

- Community viral load (if funding is available)
    - Viral load in approximately 75 HIV-infected individuals per cluster at 36 months in the *Population Cross-Sectional Survey*
- ART adherence and viral suppression
    - HIV viral load at 36 months in HIV-infected members of *the Population Cross-Sectional Survey* who initiated HIV care and ART after commencement of the PopART intervention in the community (if funding available)
    - Self-reported adherence to ART in HIV infected members of the *Population Cross-Sectional Survey* who initiated HIV care and ART after commencement of the PopART intervention in the community, measured at 36 months
- ART drug resistance (if funding is available)
    - ART resistance at 36 months in HIV-infected members of the *Population Cross-Sectional Survey* who initiated HIV care and ART after commencement of the PopART intervention in the community, among individuals who are not virally suppressed at 36 months
- HIV disease progression
    - CD4 counts, WHO staging events, and retention in care among members of the *Population Cross-Sectional Survey* initiating ART after commencement of the PopART intervention in the community, measured using routine health center data (consent to use linked routine clinical data required)
- ART toxicity
    - ART safety and clinical events among members of the *Population Cross-Sectional Survey* initiating ART after commencement of the PopART intervention in the community, measured using routine health center and laboratory data (consent to use linked clinical data required)
- Sexual risk behavior
    - Self-reported sexual risk behavior at 36 months in the *Population Cross-Sectional Survey*
- HIV-related stigma

    - Answers to questionnaire evaluating stigma at 36 months in the *Population Cross-Sectional Survey*
- Uptake of PMTCT
    - Self-reported use of services for PMTCT among HIV-infected women in the *Population Cross-Sectional Survey* who had been pregnant in the prior 36 months
- Uptake of male circumcision
    - Self-reported circumcision uptake in the prior 36 months of men in the *Population Cross-Sectional Survey*
- ART screening and uptake
    - The proportion of members of the *Population Cross-Sectional Survey* identified as HIV-infected who have been screened for ART eligibility, and who subsequently initiated ART
- HIV testing and retesting
    - Self-report of prior HIV testing at 36 months in the *Population Cross-Sectional Survey*
- Time between HIV diagnosis and initiation of care
    - The proportion of members of the *Population Cross-Sectional Survey* initiating HIV care within 3 months of a positive HIV diagnosis
- Other testing may be performed using stored samples, as noted in the Schedule of Events

SCHEDULE OF STUDY VISITS AND PROCEDURES: POPULATION CROSS-SECTIONAL SURVEY-ALL ARMS

PROCEDURES	Single Visit at 36 Month Time Point
**ADMINISTRATIVE, BEHAVIORAL, AND REGULATORY PROCEDURES**	
Obtain informed consent for enrollment.<br>Solicit consent for storage of specimens for future testing and for access to data collected at health centers	X
Obtain locator information.	X
Administer survey to include socio-demographic, health, social, behavioral, and economic factors	X
**CLINICAL/COUNSELING PROCEDURES**	
Perform HIV rapid testing[1]	X
Collect blood for laboratory testing and sample storage	X
Provide HIV pre- and post-test counseling and HIV rapid test results, for those willing to receive results.	X
**LABORATORY PROCEDURES**	
Plasma for storage[2]	X
HIV testing using stored samples[3]	X
HIV viral load testing using stored plasma[2,4]	X
HIV resistance testing using stored samples[5]	X
Other testing using stored samples[6]	X

Footnotes for the *Population Cross-Sectional Survey*

[1] Rapid testing will be performed at home visits according to in-country guidelines. This testing will not be used to estimate HIV prevalence; however, the data may be captured along with other data from the home visit. Tie-breaker testing may or may not be performed in the home.

[2] Plasma will be stored at an in-country centralized laboratory. Stored samples will be used for testing at one or more centralized laboratories and at the HPTN Network Laboratory in the US. Some testing may be performed at other laboratories at the discretion of the HPTN Network Laboratory. Testing performed on stored samples will not be performed in real-time. In most cases, results will not be returned to study sites or participants. Samples will be shipped to the HPTN NL on request.

[3] This testing may be performed at a centralized in-country laboratory designated by the HPTN NL.

[4] HIV viral load testing using stored samples will be performed to determine community viral load. This testing will be performed for ~75 HIV-infected participants per community.

[5] Testing for antiretroviral drug resistance will be performed on selected samples at a centralized laboratory selected by the HPTN NL.

[6] Additional testing will be performed at the HPTN NL or at another laboratory at the discretion of the HPTN NL. Retrospective HIV testing will be performed for quality assurance and to determine HIV prevalence. Additional testing related to assess HIV incidence may also be performed. Results from this testing will not be returned to study participants; they will have access to HIV diagnostic testing during the home visit and at the health centers. A subset of samples may also be used to characterize the HIV virus and the host response to HIV infection, to analyze linkage of HIV infections, or to measure/detect the presence of antiretroviral drugs in samples.

APPENDIX IX- TB PREVALENCE SURVEY

The following objectives and procedures describe a community level TB prevalence survey (approximately 4250 individuals in each study community) that, if funded, will be conducted after three years of intervention to assess the effect of the interventions on the burden of undiagnosed TB at community level. All adults residing in the home of a *Population Cohort* member at month 36, and all adults residing in a home participating in the *Population Cross-Sectional Survey* will be asked to give consent to participate in the TB prevalence survey. All participants will be asked a short questionnaire about current and past TB and risk factors for TB. They will be asked to produce a respiratory sample and to have an HIV test done (on-the-spot counseling and rapid testing according to national algorithm). All respiratory samples will be tested for *Mycobacterium tuberculosis* and any individuals who test positive will be re-visited and asked to provide a second confirmatory sample. Any individual found to have undiagnosed TB will be commenced on treatment through the national TB program procedures that are available at every health facility in the study

Secondary Study Objective: To measure the impact of the two intervention packages on the prevalence of TB

Procedures:

1.1 Inclusion Criteria

- Adult aged 18 years and above
- Currently residing in a household included in the *Population Cohort* or *Population Cross-Sectional Survey*
- Residing within catchment area of a designated local health unit for the three years prior to conduct of the survey
- Able and willing to provide informed consent.

1.2 Exclusion Criteria

- Anything that, in the opinion of the investigator, would preclude informed consent, make study participation unsafe, complicate interpretation of study outcome data, or otherwise interfere with achieving the study objectives.

1.3 Visit Procedures for the *TB Prevalence Survey* (Month 36 only)

Administrative, Behavioral, and Regulatory Procedures

- Obtain informed consent for enrollment
- Solicit consent for storage of specimens for future testing
- Obtain locator information
- Administer TB questionnaire

Clinical/Counseling Procedures

- Perform HIV rapid tests, if participant agrees

- Provide pre- and post-test counseling and HIV rapid test results, for those willing to receive results
- Collect respiratory sample for laboratory testing

Laboratory Procedures:

- TB testing and storage of sputum samples

Table 1- Study Activities across All Study Arms

Study Procedures/Activity	**Arm A**	**Arm B**	**Arm C**
TB Survey			
Informed Consent	X	X	X
Completion of TB questionnaire	X	X	X
Blood draw and respiratory sample for TB	X	X	X

Statistical Analysis of Prevalence of Bacteriologically-Confirmed Pulmonary TB 36 Months after Start of Intervention

This will be measured among all adult household members (aged 18 years or older) in both *Population Cohort* and *Population Cross-Sectional Survey* households. Assuming 1875 *Population Cohort* households are still in follow-up at 36 months, and an average of 2 adult participants per household, this gives a total sample size of 4250 adults per community. In 2010, the average prevalence of culture-confirmed pulmonary TB across the trial communities was 1.2%. Using an automated diagnostic test instead of culture, and assuming that the sensitivity of that test in the context of a prevalence survey is of the order of 65%, the observed overall prevalence would be 0.8%. There is 70% power to show a 40% reduction in tuberculosis prevalence to 0.48%, 82% power to show a 45% reduction to 0.44%, and 90% power to show a 50% reduction to 0.40%, with k=0.25. The value of k (within matched triplets) is anticipated to be approximately 0.25, from the findings of the 2010 TB prevalence survey in these communities.

SCHEDULE OF STUDY VISITS AND PROCEDURES: TUBERCULOSIS SURVEY- ALL ARMS

PROCEDURES	Single Visit at 36 Month Time Point
**ADMINISTRATIVE, BEHAVIORAL, AND REGULATORY PROCEDURES**	
Obtain informed consent for enrollment.<br>Solicit consent for storage of specimens for future testing.	X
Obtain locator information.	X
Administer TB questionnaire	X
**CLINICAL/COUNSELING PROCEDURES**	
Perform HIV rapid testing	X
Collect sputum for laboratory testing and sample storage	X
Provide HIV pre- and post-test counseling and HIV rapid test results, for those willing to receive results.	X
**LABORATORY PROCEDURES**	
Sputum testing for Mycobacterium tuberculosis[1]	X

[1]Additional details about TB testing can be found in the SSP manual.

APPENDIX X- PMTCT SURVEY

The following objectives and procedures are focused on determination of the impact of the main study interventions on the prevention of mother to child transmission of HIV (PMTCT) as measured by HIV-free survival of children born during the intervention period. This was not part of the scope of the original protocol, but the team is pursuing additional funding to support this work and has thought carefully about how the work would best be incorporated into the main study.

If the survey is funded, all adult females residing in the home of a *Population Cohort* member at month 36, and all adult females residing in a home participating in the *Population Cross-Sectional Survey* will be asked if they have given birth in the prior 36 months. Those who have given birth during that period will be asked to consent for participation in an interview to record delivery outcome and child survival, and to have a rapid HIV test and blood spot collection via finger stick. If the woman tests HIV positive, she will be asked to have her child tested for HIV, if the child is alive.

Secondary Study Objective:

- To measure the impact of the two intervention packages on HIV-free survival of children born during the intervention period

Procedures:

1.1 Inclusion Criteria

- Female aged 18+, who has given birth in the last 36 months
- Currently residing in a household included in the *Population Cohort* or *Population Cross-Sectional Survey*
- Residing within the catchment area of a designated local health unit for the three years prior to conduct of the survey
- Able and willing to provide informed consent

1.2 Exclusion Criteria

- Anything that, in the opinion of the investigator, would preclude informed consent, make study participation unsafe, complicate interpretation of study outcome data, or otherwise interfere with achieving the study objectives.

1.3 Visit Procedures for the *PMTCT Survey* (Month 36 only)

Administrative, Behavioral, and Regulatory Procedures

- Obtain informed consent for enrollment
- Obtain/update locator information
- Solicit consent for storage of specimens for future testing
- Obtain information on delivery outcome and child survival of all births that occurred within the past 36 months

Clinical/Counseling Procedures

- Perform HIV rapid tests for the mother if participant agrees
- Provide pre- and post-test counseling and HIV rapid test results, for those willing to receive results
- For all mothers with at least one positive HIV rapid test, prepare dry blood spot from mother's finger stick for storage
- Collect heel prick blood sample from all children who are aged 36 months or less and whose mothers are HIV-infected or where the mother does not consent to be tested herself, but does consent to testing of her child
- Prepare dried blood spot from heel prick blood sample for HIV testing and storage

Laboratory Procedures:

- HIV testing of child dried blood spot samples

Table 1- Study Activities across All Study Arms

Study Procedures/Activity	**Arm A**	**Arm B**	**Arm C**
PMTCT Survey			
Informed Consent	X	X	X
Inquiry regarding delivery and child survival	X	X	X
Finger stick (mother) and/or heel stick (child) for HIV testing and sample storage	X	X	X

Statistical Analysis of HIV-Free Survival among Children Born During the 36 Months of Trial Intervention

Assuming 1875 *Population Cohort* households are still in follow-up at 36 months and that an additional 250 households are included in the *Population Cross-Sectional Survey*, and that average household size is 4.7 (based on recent data from trial communities), the total population of included households at 36 months will be 9988 in each trial community. With an estimated birth rate of 40 per 1000 population, approximately 1199 children will be born in these households during the three years of trial intervention, and it is expected that approximately 180 (15%) will be born to HIV-infected mothers. At the 36-month follow-up, the average time since birth will be one and a half years, assuming a constant birth rate. If for 15% of mother-child pairs in which the mother was HIV-infected at the time of delivery, either both the mother and child have died or consent is not given for HIV testing, it will be possible to estimate HIV-free survival for 153 children per community, with 229 person-years of follow-up. Assuming that the rate of the composite outcome of HIV infection/mortality is between 9 and 11 per 100 person-years among children born to HIV-positive mothers in Arm C, and that k=0.15, there is good study power to show a reduction in HIV infection/mortality of 40% or more, and moderate study power to show a reduction of 35%, in Arm A and Arm B. With k=0.2, there is good study power to show a reduction of 45% or more. These estimates of HIV infection/mortality rates among babies born to HIV-positive mothers in Arm C, and possible reductions of 40-50% with timely initiation of HAART in HIV-positive pregnant women, are based on a recent study from Kenya (Kesha Bora study), and unpublished data from a recent large study, the PEARL study, which was conducted in Zambia and other sites in sub-Saharan Africa (Stringer et al).

Laboratory Specimens

The following specimens will be collected to address the PMTCT endpoint for testing at the local laboratory:

- Maternal blood specimen (finger-stick) for
  - HIV rapid testing
  - Preparation of dried blood spots for storage
- Child blood specimen (heel stick) in a subset of children for
  - Preparation of dried blood spots for HIV testing and storage

SCHEDULE OF STUDY VISITS AND PROCEDURES: PMTCT SURVEY- ALL ARMS

	Single Visit at 36 Month Time Point
**PROCEDURES FOR MOTHER**[1]	
**ADMINISTRATIVE, BEHAVIORAL, AND REGULATORY PROCEDURES**	
Obtain informed consent for enrollment.<br>Solicit consent for storage of specimens for future testing.	X
Obtain/update locator information.	X
Obtain information about delivery outcome and child survival of all births in the last 36 months	X
**CLINICAL/COUNSELING PROCEDURES**	
Finger stick for HIV rapid testing and preparation of dried blood spots	X
Perform HIV rapid testing[2]	X
Prepare dried blood spots for storage (for persons with at least one reactive rapid test)	X
Provide HIV pre- and post-test counseling and HIV rapid test results, for those willing to receive results.	X
**PROCEDURES FOR INFANT/CHILD (children < 36 months of age, if mother is HIV-infected (or opts not to have HIV test) and provides consent for testing of the child)**[1]	
**CLINICAL/COUNSELING PROCEDURES**	
Heel-stick and preparation of dried blood spots	X
Prepare dried blood spots for HIV testing and storage	X
**LABORATORY PROCEDURES**	
HIV testing of child dried blood spots[3]	X
Other testing of maternal and child dried blood spots[4]	X

Footnotes for the PMTCT cohort

[1] For all women who have given birth in the prior 36 months who agree to participate

[2] Rapid testing will be performed according to in-country guidelines. This testing will not be used to estimate HIV prevalence; however, the data may be captured along with other data from the home visit. Tie-breaker testing may or may not be performed in the home.

[3] This testing will be centralized at a laboratory determined by the HPTN NL. Results from this testing will be returned to study participants.

[4] Additional testing may be performed at the HPTN NL. This may include quality assurance testing of HIV testing for the mother and child. Other testing may include HIV resistance testing, or other testing to characterize the virus or the host response to viral infection. Results from testing performed at the HPTN NL will not be returned to study sites or participants.

PROTOCOL VERSION 3.0

16 NOV 2015

HPTN 071
Population Effects of Antiretroviral Therapy to Reduce HIV Transmission (PopART): A cluster-randomized trial of the impact of a combination prevention package on population-level HIV incidence in Zambia and South Africa

A Study of the HIV Prevention Trials Network

DAIDS ID:
11865

Sponsored by:
Division of AIDS, National Institute of Allergy and Infectious Diseases
U.S. National Institutes of Health

Funded by:
National Institute of Allergy and Infectious Diseases
National Institute of Mental Health
Office of the United States Global AIDS Coordinator
Bill and Melinda Gates Foundation
U.S. National Institutes of Health

Protocol Chair:
Richard Hayes
London School of Hygiene & Tropical Medicine
London, United Kingdom

Protocol Co-Chair:
Sarah Fidler
Imperial College
London, United Kingdom

Final Version 3.0
16 November 2015

Non-IND Study

HPTN 071
Population Effects of Antiretroviral Therapy to Reduce HIV Transmission (PopART): A cluster-randomized trial of the impact of a combination prevention package on population-level HIV incidence in Zambia and South Africa

TABLE OF CONTENTS

LIST OF TABLES AND FIGURES 6
LIST OF ABBREVIATIONS AND ACRONYMS 7
PROTOCOL TEAM ROSTER 9
INVESTIGATOR SIGNATURE PAGE 15
SCHEMA 16
OVERVIEW OF STUDY DESIGN AND RANDOMIZATION SCHEME 19
1.0 INTRODUCTION 20
1.1 Background and Prior Research 20
1.2 Rationale 20
1.2.1 The HIV Epidemic in Sub-Saharan Africa 21
1.2.2 HIV Prevention Methods 22
1.2.3 Anti-Retroviral Therapy (ART) for HIV Prevention 24
1.2.4 Innovation 26
2.0 STUDY OBJECTIVES AND DESIGN 29
2.1 Primary Objective 29
2.2 Secondary Objectives 29
2.3 Study Design 31
2.4 Timing of Deployment of Intervention and Research Components 33
2.5 Cross-Sectional HIV Incidence Estimation 35
3.0 STUDY INTERVENTION 35
3.1 Implementation Team Experience 35
3.2 Description of CHiP Teams 36
3.3 Universal HIV Testing and Linkage to Care 36
3.4 Male Circumcision 37
3.5 Universal Treatment 37
3.5.1 Choice of ART Regimen 38
3.6 Treatment According to Local Guidelines (Arms B & C) 38
3.7 Prevention of Mother-to-Child Transmission 39
3.8 Management of Sexually Transmitted Infections 39
3.9 Screening and Referral for TB 39
3.10 Provision of PrEP 40
3.11 Standard of Care 40
3.12 Delivery of Intervention 40
3.12.1 Activities with Local Health Centers/Community Institutions 40
3.12.2 Collaborations 41
3.13 Monitoring and Evaluation Plan 43
4.0 STUDY POPULATION 45
4.1 Description/Selection of the 21 Study Communities 45

4.2 Randomization ... 48
4.3 Community Engagement ... 48
5.0 RESEARCH PROCEDURES AND ACTIVITIES ... 49
5.1 Population Cohort ... 50
5.1.1 Sampling/Recruitment of *Population Cohort* ... 50
5.1.2 Inclusion Criteria *Population Cohort* ... 50
5.1.3 Exclusion Criteria *Population Cohort* ... 50
5.1.4 Procedures and Activities ... 51
5.1.5 Reviewing Health Center Records for *Population Cohort* ... 52
5.1.6 Retention in *Population Cohort* ... 52
5.2 Population Cross-Sectional Survey (if funded) ... 53
5.2.1 Sampling/Recruitment of *Population Cross-Sectional Survey* Participants ... 53
5.2.2 Inclusion Criteria ... 53
5.2.3 Exclusion Criteria ... 53
5.2.4 Procedures and Activities ... 54
5.3 Case-Control Studies ... 54
5.3.1 *Case-Control Study 1* - Uptake of Testing in the First Round of Home-Based Testing Provided by CHiP Teams in Arms A & B ... 54
5.3.1.1 Sampling/Recruitment of Case-Control Study 1 Participants ... 55
5.3.1.2 Inclusion Criteria Case-Control Study 1 ... 55
5.3.1.3 Exclusion Criteria Case-Control Study 1 ... 55
5.3.1.4 Procedures and Activities ... 55
5.3.2 *Case-Control Study 2* - Uptake of Immediate Treatment in Arm A ... 55
5.3.2.1 Sampling/Recruitment of Case-Control Study 2 Participants ... 56
5.3.2.2 Inclusion Criteria Case-Control Study 2 ... 56
5.3.2.3 Exclusion Criteria Case-Control Study 2 ... 56
5.3.2.4 Procedures and Activities ... 56
5.3.3 *Case-Control Study 3* - Uptake of Testing in the Second Round of Home-Based Testing Provided by CHiP Teams in Arms A & B ... 57
5.3.3.1 Sampling/Recruitment of Case-Control Study 3 Participants ... 57
5.3.3.2 Inclusion Criteria Case-Control Study 3 ... 57
5.3.3.3 Exclusion Criteria Case-Control Study 3 ... 57
5.3.3.4 Procedures and Activities ... 57
5.4 Qualitative Studies ... 58
5.4.1 Evaluation of the Acceptability of the Intervention: ... 58
5.4.2 Qualitative Longitudinal Study in Arms A and B – sub-set of *Case-Control Study 1* 59
5.4.3 Ethnography of the HIV landscape ... 60
5.4.4 Graphical Summary of Qualitative Activities ... 60
5.4.5 Integration of Data from Case-Control and Qualitative Components ... 61
5.5 Collection of Health Center-Based Data ... 61
5.5.1 Tuberculosis Case Notification ... 61
5.5.2 Intervention Effect on Health Center Workload ... 62
5.5.3 Intervention Effect on Healthcare Costs ... 62
5.5.4 HIV Disease Progression and Death ... 63
5.5.5 ART Adherence and ART Toxicity ... 63
5.5.6 Uptake of Intervention Components ... 63

5.6 Proposed Additional Surveys ... 63
5.7 Comparative Table of Study Activities across All Study Arms ... 64
6.0 SAFETY MONITORING AND SOCIAL HARM REPORTING ... 65
6.1 Safety Monitoring ... 65
6.2 Social Harm Reporting ... 65
6.2.1 Participants in the *Population Cohort* ... 65
6.2.2 Community at Large ... 66
6.2.3 Social Harm Monitoring ... 66
7.0 STATISTICAL CONSIDERATIONS AND DATA ANALYSIS ... 66
7.1 Sample Size ... 66
7.1.1 Mathematical Modeling and Sample Size Calculations ... 66
7.1.2 Primary Endpoint - HIV Incidence Over 36 Months ... 69
7.1.3 Secondary Endpoints ... 73
7.2 Random Assignment / Study Arm Assignment ... 77
7.3 Statistical Analysis ... 78
7.4 Interim Evaluation ... 79
7.4.1 HIV Incidence ... 79
7.4.2 Uptake of Intervention ... 80
7.5 Mathematical Modeling ... 80
7.6 Outcomes for Secondary Objectives ... 81
7.7 Secondary Objectives for Case-Control Studies ... 83
7.8 Secondary Objectives for Qualitative Studies ... 84
7.9 Secondary Objectives Related to Economic Evaluation ... 84
7.10 Process Measures ... 89
7.11 Tabular Summary of Outcomes ... 90
8.0 HUMAN SUBJECTS CONSIDERATIONS ... 94
8.1 Collaborative Partnerships ... 94
8.2 Social Value ... 94
8.3 Scientific Validity ... 94
8.4 Fair Subject Selection ... 95
8.5 Risk-Benefit Assessment ... 96
8.5.1 Community Level ... 96
8.5.1.1 Benefits ... 96
8.5.1.2 Risks ... 96
8.5.1.3 Minimizing Risks to Communities ... 97
8.5.1.4 Risk-Benefit Assessment at Community Level ... 97
8.5.2 Individual Level ... 97
8.5.2.1 Benefits ... 97
8.5.2.2 Risks ... 98
8.5.2.3 Minimizing Risks to Individuals ... 99
8.5.2.4 Risk- Benefit Assessment at Individual level ... 100
8.6 Informed Consent ... 100
8.6.1 Approval from Respective Authorities ... 101
8.6.2 ‘Community’ Consent ... 101
8.6.3 Individual Consent ... 101
8.6.4 Waiver of Individual Consent to Access CHiP and Routine Clinic Data ... 103
8.7 Independent Ethical Review ... 104

8.8 Respect for Participants and Communities During and After the Study 104
8.8.1 Confidentiality 104
8.8.2 Data and Safety Monitoring Plan 105
8.8.3 Communicable Disease Reporting Requirements 105
8.8.4 Post-Trial Management of Participants Exposed to the Early ARV Intervention . 105
8.8.5 Study Discontinuation 105
9.0 LABORATORY SPECIMENS AND BIOHAZARD CONTAINMENT 106
9.1 Local Laboratory Specimens 106
9.2 HPTN Laboratory Center (LC) Specimens 106
9.3 Quality Control and Quality Assurance Procedures 107
9.4 Specimen Storage and Possible Future Research Testing 107
9.4.1 Proposed Phylogenetics Study 107
9.5 Biohazard Containment 108
10.0 ADMINISTRATIVE PROCEDURES 108
10.1 Protocol Registration 108
10.2 Study Activation 109
10.3 Study Coordination 109
10.4 Study Monitoring 109
10.5 Protocol Compliance 110
10.6 Investigator's Records 110
10.7 Use of Information and Publications 110
11.0 REFERENCES 111
12.0 APPENDICES 119
APPENDIX I - SCHEDULES OF STUDY VISITS AND PROCEDURES 120
APPENDIX II - SAMPLE INFORMED CONSENT FORM – POPULATION COHORT 123
APPENDIX III - SAMPLE INFORMED CONSENT FORM – QUALITATIVE STUDIES PARTICIPANTS 130
APPENDIX IV - SAMPLE INFORMED CONSENT FORM – CASE CONTROL STUDIES PARTICIPANTS 136
APPENDIX V - SAMPLE INFORMED CONSENT FORM –PARTICIPANTS STARTING ART OUTSIDE OF LOCAL GUIDELINES 141
APPENDIX VI - SAMPLE INFORMED CONSENT FORM – CHIP TEAM ACTIVITIES ... 147
APPENDIX VII – SAMPLE SIZE CALCULATIONS 152
APPENDIX VIII - PROPOSED POPULATION CROSS-SECTIONAL SURVEY 163

LIST OF TABLES AND FIGURES

List of Tables

Table 1- Summary of Intervention Components ... 42

Table 2- Twenty one proposed study clusters in Zambia and South Africa and relevant background data ... 46

Table 3- Study Activities across All Study Arms ... 64

Table 4- Parameter values assumed for the model of the impact of the intervention for central and optimistic target scenarios, and projected impact on HIV incidence in Arms A and B compared with Arm C, assuming intervention roll-out over a 6-month time period ... 68

Table 5- Parameter values assumed for the model of the impact of the intervention for central and optimistic target scenarios, and projected impact on HIV incidence in Arms A and B compared with Arm C, assuming intervention roll-out over a 12-month time period ... 69

Table 6- Power for comparison of HIV incidence in Arm A or B with Arm C, with 7 communities per arm and Population Cohort of 2500 adults per community (assuming that on average 2125 (85%) will be HIV-uninfected at baseline and that loss to follow-up will be 20% after 2 years and 25% after 3 years) with 5206 person-years per community over 36 months (assuming 1912 person-years 0-12 months; 1700 person-years 12-24 months; 1594 person-years 24-36 months) ... 71

Table 7- Power for comparison of HIV incidence between Arms A and B, with 7 communities per arm and Population Cohort of 2500 adults per community (assuming that on average 2125 (85%) will be HIV-uninfected at baseline and that loss to follow-up will be 20% after 2 years and 25% after 3 years) ... 72

Table 8- Case-control studies of (1) uptake of HIV testing (2) uptake of re-testing for HIV and (3) uptake of immediate ART among HIV-positive individuals ... 76

Table 9- Summary, outcomes for secondary objectives related to the economic analysis ... 87

Table 10- Summary of Study Objectives and Related Outcomes ... 91

List of Figures

Figure 1- Community Randomization Scheme, Zambia and South Africa ... 32

Figure 2- Timing of Deployment of Intervention and Research Component ... 34

Figure 3- Location of 21 clusters in Zambia and South Africa ... 47

Figure 4- Qualitative Activities in HPTN 071 ... 61

Figure 5- Mathematical model of the epidemic and of the PopART interventions ... 67

LIST OF ABBREVIATIONS AND ACRONYMS

AE	Adverse Event
AIDS	Acquired Immunodeficiency Syndrome
ANC	Antenatal Clinic
ART	Anti-Retroviral Therapy
ARV	Anti-Retroviral
CDC	Centers for Disease Control and Prevention
CFR	Code of Federal Regulations
CHiPs	Community HIV-care Providers
CORE	(HPTN) Coordinating and Operations Center
DAIDS	Division of AIDS
DALY	Disability-Adjusted Life-Year
DSMB	Data Safety Monitoring Board
EC	Ethics Committee
EQA	External Quality Assurance
FHCRC	Fred Hutchinson Cancer Research Center
GCLP	Good Clinical Laboratory Practice
GCP	Good Clinical Practice
HCT	HIV Counseling and Testing
HIV	Human Immunodeficiency Virus
HPTN	HIV Prevention Trials Network
HSV-2	Herpes Simplex Virus, Type 2
IATA	International Air Transport Association
ICF	Informed Consent Forms
IRB	Institutional Review Board
LC	(HPTN) Laboratory Center
LDMS	Laboratory Data Management System
MSM	Men who have Sex with Men
NIAID	(United States) National Institute of Allergy and Infectious Diseases
NIH	(United States) National Institutes of Health
PMTCT	Prevention of Mother to Child Transmission of HIV
PrEP	Pre-Exposure Prophylaxis
PRO	Protocol Registration Office
QA	Quality Assurance
QALY	Quality-Adjusted Life-Year
QC	Quality Control
RE	Regulatory Entity
RNA	Ribonucleic Acid
RR	Rate Ratio
RSC	Regulatory Services Center

SAE	Serious Adverse Event
SANAC	South African National AIDS Council
SDMC	(HPTN) Statistical and Data Management Center
SMC	Study Monitoring Committee
SMS	Short Message Service
START	Strategic Timing of Anti-Retroviral Treatment
STI	Sexually Transmitted Infection
SSP	Study Specific Procedures
TB	Tuberculosis
UK	United Kingdom
UNAIDS	United Nations Programme on HIV/AIDS
US	United States (of America)
UTT	Universal Testing and Treatment
VCT	Voluntary Counseling and Testing
WHO	World Health Organization
ZAMBART	Zambia AIDS Related Tuberculosis Project
ZAMSTAR	Zambia-South Africa TB and AIDS Reduction Program

HPTN 071
Population Effects of Antiretroviral Therapy to Reduce HIV Transmission (PopART): A cluster-randomized trial of the impact of a combination prevention package on population-level HIV incidence in Zambia and South Africa

PROTOCOL TEAM ROSTER

Yaw Agyei
International HPTN LC QA/QC Coordinator
Dept. of Pathology
Johns Hopkins Univ. School of Medicine
Pathology Building, Room 313
600 North Wolfe Street
Baltimore, MD 21287, USA
Phone: +27-813766180/+001-410-614-6736
Email: yagyei1@jhmi.edu

Helen Ayles
Clinical Scientist/Site Principal Investigator
ZAMBART
University of Zambia
School of Medicine, Ridgeway Campus
Lusaka, Zambia
Dept of Clinical Research
London School of Hygiene and Tropical Medicine
Keppel Street
London WC1E 7HT, UK
Phone: +260 211 254710, 260 211257215
Fax: +260 211 254710
Email: helen@zambart.org.zm

Mark Barnes
HPTN Ethics Working Group Representative
Partner, Ropes & Gray LLP
Lecturer, Harvard Law School
Prudential Tower, 800 Boylston Street
Boston, MA 02199-3600
Phone: +001-617-951-7827
Email: mark.barnes@ropesgray.com

Nulda Beyers
Clinical Scientist/Site Principal Investigator
Desmond Tutu TB Centre
Stellenbosch University
Francie van Zijl Avenue
Clinical Building, K floor, Room 0065
Tygerberg Campus
Western Cape, 7505, South Africa
Phone: +21-21 938 9114
Fax: + 27-21 938 9719
Email: nb@sun.ac.za

Peter Bock
Site Co- Principal Investigator
Desmond Tutu TB Centre
Stellenbosch University
Francie van Zijl Avenue
Clinical Building, K floor, Room 0065
Tygerberg Campus
Western Cape, 7505, South Africa
Phone: +27 835 721470
Email: peterb@sun.ac.za

Virginia Bond
Social Scientist
London School of Hygiene & Tropical Medicine
ZAMBART Project, School of Medicine
P.O. Box 50697
Lusaka, Zambia
Phone: +260 211 254710
Email: gbond@zambart.org.zm

Lisa Bunts
Lab Data Manager, Lab Data Operations
SCHARP-FHCRC
1100 Fairview Ave. North, LE-400
PO Box 19024
Seattle, WA 98109, USA
Phone: 206-667-3026
Email: lbunts@scharp.org

David Burns
Prevention Sciences Branch Chief
DAIDS Medical Officer
NIAID/NIH
5601 Fishers Lane, Room 8B40
Rockville MD 20852, USA
Phone: +001-301-435-8896
Email: burnsda@niaid.nih.gov

Anne Cori
Mathematical Modeler
Imperial College London
Department of Infectious Disease
Epidemiology
St Mary's Campus
Norfolk Place
London W2 1PG, UK
Phone: +44 (0) 20 75943229
Email: a.cori@imperial.ac.uk

Vanessa Cummings
HPTN LC QA/QC Representative
Dept. of Pathology
Johns Hopkins Univ. School of Medicine
Pathology Building, Room 313
600 North Wolfe Street
Baltimore, MD 21287, USA
Phone: +001-410-614-0479
Fax: +001-410-614-0430
Email: vcummin1@jhmi.edu

Deborah Donnell
SDMC Biostatistician
SCHARP-FHCRC
1100 Fairview Ave. North, LE-400
PO Box 19024
Seattle, WA 98109, USA
Phone: +001-206-667-5661
Fax: +001-206-667-4812
Email: deborah@scharp.org

Lynda Emel
Associate Director, HPTN SDMC
SCHARP-FHCRC
1100 Fairview Ave. North, E3-129
PO Box 19024
Seattle, WA 98109, USA
Phone: +001-206-667-5803
Email: lemel@scharp.org

Susan Eshleman
HPTN Laboratory Center
Johns Hopkins University School of
Medicine
720 Rutland Ave.
Ross Building, Room 646
Baltimore MD, 21205, USA
Phone: +001-410-614-4734
Fax: +001-410-502-9244
Email: seshlem@jhmi.edu

Sarah Fidler
Protocol Co-Chair
Imperial College London
CSL in Communicable Diseases
St Mary's Campus
HIV Clinical Trials Unit
Winston Churchill Wing
London W2 1NY, UK
Phone: +44 (0) 203 312 6790
Email: s.fidler@imperial.ac.uk

Sian Floyd
Statistician
Department of Infectious Disease Epidemiology
London School of Hygiene & Tropical Medicine
Keppel Street
London, WC1E 7HT, UK
Phone: +44-207-6127888
Fax: +44-207-6368739
Email: sian.floyd@lshtm.ac.uk

Christophe Fraser
Mathematical Modeler
Imperial College London
Department of Infectious Disease Epidemiology
St Mary's Campus
Norfolk Place
London W2 1PG, UK
Phone: +44 (0) 20 75943397
Email: c.fraser@imperial.ac.uk

Peter Godfrey-Faussett
Professor of International Health
Room 303b
London School of Hygiene & Tropical Medicine
Keppel Street
London WC1E 7HT
Phone: +44 020 7958 8127
Fax: +44 020 7612 7860
Email: peter.godfrey-faussett@lshtm.ac.uk

Sam Griffith
Senior Clinical Research Manager
FHI 360
359 Blackwell Street, Suite 200
Durham NC 27701, USA
Phone: +001-919-544-7040 ext. 11571
Email: sgriffith@fhi360.org

James Hargreaves
Senior Lecturer in Epidemiology
Department of Infectious Disease Epidemiology
London School of Hygiene & Tropical Medicine
Keppel Street
London WC1E 7HT, UK
Phone: +44 (0)20 7927 2955
Fax: +44 (0)20 7637 4314
Email: james.hargreaves@lshtm.ac.uk

Katharina Hauck
Health Economist
Imperial College Business School
Tanaka Building
South Kensington Campus
London SW7 2AZ, UK
Phone: +44 (0)20 7594 9197
Fax: +44 (0)20 7594 9189
Email: k.hauck@imperial.ac.uk

Richard Hayes
Protocol Chair, Lead Statistician
Department of Infectious Disease Epidemiology
London School of Hygiene & Tropical Medicine
Keppel Street
London WC1E 7HT, UK
Phone: +44 (0)20 7927 2243
Fax: +44 (0)20 7637 4314
Email: richard.hayes@lshtm.ac.uk

Tanette Headen
Research Assistant II
FHI 360
359 Blackwell Street, Suite 200
Durham, NC 277101
Phone: +001-919-544-7040 ext. 11297
Email: theaden@fhi360.org

Graeme Hoddinott
Social Scientist
Desmond Tutu HIV Centre
Stellenbosch University
Francie van Zijl Avenue
Clinical Building, K Floor, Room 0081
Tygerberg Campus
Cape Town, Western Cape 7505
South Africa
Phone: +_27 21 938 9846
Email: graemeh@sun.ac.za

Lyn Horn
Ethicist
Desmond Tutu TB Centre
Division Research Development
Stellenbosch University
Phone: +27 21 808 2670
Email: lhorn@sun.ac.za

Erin Hughes
Project Manager
SCHARP-FHCRC
1100 Fairview Avenue North
E3-129
PO Box 19024
Seattle, WA 09109 USA
Phone: 206-667-7109
Email: *eehughes@scharp.org*

Mohammed Limbada
Clinical scientist
ZAMBART
University of Zambia Ridgeway campus
Lusaka
Zambia
Phone : +260 211254710
Fax: +260 211254710
Email: Mohaamed@zambart.org.zm

Ayana Moore
Scientist
FHI 360
359 Blackwell Street, Suite 200
Durham NC 27701, USA
Phone: +001-919-544-7040 ext. 11244
Email: amoore@fhi360.org

Albert Mwango
ART Coordinator, Ministry of Health, Zambia
ZAMBART
University of Zambia
School of Medicine, Ridgeway Campus
Lusaka, Zambia
Phone: +260 211 254710, 260 211257215
Fax: +260 211 254710
Email: albert.mwango@moh.gov.zm

Alwyn Mwinga
Implemention Coordinator
ZAMBART
University of Zambia
School of Medicine, Ridgeway Campus
Lusaka, Zambia
Phone: +260 211 254710, 260 211257215
Fax: +260 211 254710
Email: Alwyn@Zambart.org.zm

Michael Pickles
Mathematical Modeler
Imperial College London
Department of Infectious Disease
Epidemiology
St Mary's Campus
Norfolk Place
London W2 1PG, UK
Phone: +44 (0) 20 75943266
Email: m.pickles@imperial.ac.uk

Estelle Piwowar-Manning
HPTN Laboratory Center, Department of Pathology
Johns Hopkins Univ. School of Medicine
600 North Wolfe St.
Pathology 306
Baltimore, MD 21287, USA
Phone: +001-410-614-6736
Fax: +001-410-614-0430
Email: epiwowa@jhmi.edu

Kalpana Sabapathy
Clinical Epidemiologist
Department of Infectious Disease Epidemiology
London School of Hygiene & Tropical Medicine
304-B, Keppel Street, Room 112
London WC1E 7HT, UK
Phone: +44 7927 2155
Fax: +44 (0)20 7637 4314
Email: kalpana.sabapathy@lshtm.ac.uk

Ab Schaap
Senior Data Manager
ZAMBART Project
London School of Hygiene & Tropical Medicine
Ridgeway Campus
P.O. Box 50697
10101 Lusaka, Zambia
Phone: +260 211 254710, Mobile: +260 977 442965
Fax: +260-211-254710
Email: ab@zambart.org.zm

Kwame Shanaube
PopART Study Manager - Zambia
ZAMBART Project
PO Box 50697
Ridgeway Campus
Lusaka, Zambia
ZAMBIA
Phone : +260 211 254710,, 260-211-257215
Fax : +260 211 254710
Email : kshanaube@zambart.org.zm

Musonda Simwinga
Social Scientist/Community Coordinator
ZAMBART
University of Zambia
School of Medicine, Ridgeway Campus
Lusaka, Zambia
Phone: +260 211 254710, 260 211257215
Fax: +260 211 254710
Email: Musonda@zambart.org.zm

Peter C. Smith
Emeritus Professor of Health Policy
Imperial College Business School
Centre for Health Policy
Professor of Health Policy
Exhibition Road
London SW7 2AZ, UK
Phone: +44 (0)20 7594 1904
Email: peter.smith@imperial.ac.uk

Sten Vermund
HPTN Executive Committee Liaison
Vanderbilt University, Institute for Global Health
2525 West End Ave.
Suite 750
Nashville TN 32703, USA
Phone: +001-615-322-9374 (office)
Fax: +001-615-343-7797
Email: sten.vermund@vanderbilt.edu

Deborah Watson-Jones
Clinical Epidemiologist
Department of Clinical Research
London School of Hygiene & Tropical Medicine
Mwanza Intervention Trials Unit
National Institute for Medical Research
P.O. Box 11936
Mwanza, Tanzania
Phone: +255 (0) 28250 0019
Email: deborah.watson-jones@lshtm.ac.uk

Rhonda White
Community Program Manager
FHI 360
359 Blackwell Street, Suite 200
Durham NC 27701, USA
Phone: +001-919-544-7040 ext. 11515
Email: *rwhite@fhi360.org*

HPTN 071
Population Effects of Antiretroviral Therapy to Reduce HIV Transmission (PopART): A cluster-randomized trial of the impact of a combination prevention package on population-level HIV incidence in Zambia and South Africa

Final Version 3.0
16 November 2015

INVESTIGATOR SIGNATURE PAGE

A Study of the HIV Prevention Trials Network (HPTN)

Sponsored by:
Division of AIDS, National Institute of Allergy and Infectious Diseases
U.S. National Institutes of Health

Funded by:
National Institute of Allergy and Infectious Diseases
National Institute of Mental Health
Office of the United States Global AIDS Coordinator
Bill and Melinda Gates Foundation
US National Institutes of Health

I, the Investigator of Record, agree to conduct this study in full accordance with the provisions of this protocol. I agree to maintain all study documentation for a minimum of three years after submission of the site's final Financial Status Report to the Division of AIDS (DAIDS), unless otherwise specified by DAIDS or the HIV Prevention Trials Network (HPTN) Coordinating and Operations Center. Publication of the results of this study will be governed by HPTN policies. Any presentation, abstract, or manuscript will be made available by the investigators to the HPTN Manuscript Review Committee and DAIDS for review prior to submission.

I have read and understand the information in this protocol and will ensure that all associates, colleagues, and employees assisting in the conduct of the study are informed about the obligations incurred by their contribution to the study.

____
Name of Investigator of Record

____________________________________ ____________________________________
Signature of Investigator of Record Date

HPTN 071
Population Effects of Antiretroviral Therapy to Reduce HIV Transmission (PopART): A cluster-randomized trial of the impact of a combination prevention package on population-level HIV incidence in Zambia and South Africa

SCHEMA

Purpose: The purpose of this study is to determine the impact of two community-level combination prevention packages, both of which include universal HIV testing and intensified provision of HIV antiretroviral therapy (ART) and care, on population-level HIV incidence.

Design: This is a three-arm, cluster-randomized, longitudinal study to be implemented in 21 clusters (communities).

Study Population: The prevention packages will be implemented throughout the communities randomized to the intervention arms. Main study outcomes will be measured in a randomly-selected group drawn from the adult population of the communities: a *Population Cohort*.

Study Size: The combined population of all 21 clusters is approximately 1.2 million individuals. The interventions will be implemented in 14 of the 21 clusters with a combined population of approximately 800,000 individuals (adults and children) in the intervention arms. The approximate sizes of the randomly-selected groups for main study outcome assessments are:

- *Population Cohort*: 52,500 individuals
- *Case-Control Studies*: 2,400 individuals
- *Qualitative Studies*: about 2,000 individuals
- *Population Cross-Sectional Survey*: 10,500 individuals (if funded)

Note: Final sample sizes for surveys pending funding may change and will be described in separate protocol.

Study Arms/Interventions:

Arm A - Universal Testing with Immediate ART:

- Combination prevention package including:
    - House-to-house deployment of:
        - Universal HIV counseling and testing
        - Active linkage to care for individuals diagnosed as HIV-infected, with ***immediate eligibility for ART***
        - Promotion of male circumcision and prevention of mother to child transmission (PMTCT) services
        - Provision of condoms
    - Strengthening of HIV testing and services at health facilities and other venues
    - Strengthening of male circumcision and PMTCT services available in the community
    - Treatment of sexually transmitted infections (STIs) and provision of condoms at health units

Arm B - Universal Testing with ART Eligibility According to Local Guidelines:

- Combination prevention package including:
  - House-to-house deployment of:
    - Universal HIV counseling and testing
    - Active linkage to care for individuals diagnosed as HIV-infected, with ***ART eligibility according to local guidelines****
    - Promotion of male circumcision and PMTCT services
    - Provision of condoms
  - Strengthening of HIV testing and services at health facilities and other venues
  - Strengthening of male circumcision and PMTCT services available in the community
  - Treatment of STIs and provision of condoms at health units

Arm C - Standard of Care (Control Arm)

- Strengthening of HIV testing and ART services according to local guidelines* at health facilities and other venues
- Strengthening of male circumcision and PMTCT services available at health facilities and other venues in the community
- Treatment of STIs and provision of condoms at health facilities and other venues in the community

*In the initial phases of the study, Arms B and C offered treatment according to local guidelines which, at the time, based treatment eligibility upon CD4 cell count or disease progression. With protocol v.3.0, the study will (once funding is secured) provide immediate eligibility for ART in these arms (after participant consent if offered outside of local guidelines), in anticipation of changes in global standards of care.

Study Duration: The planned duration of the entire study will be approximately 6 years, with enrollment and follow-up of communities and delivery of the intervention occurring over 4 years. Assessment of the primary outcome (HIV incidence) in the *Population Cohort* is planned to take place 12, 24, and 36 months after recruitment. Interim evaluation will take place during the first two years of intervention to determine whether to continue with the 36 month follow-up of the *Population Cohort* and the fourth year of intervention.

Primary Objective:

- To measure the impact of the two intervention packages on HIV incidence by enrolling and following a random sample of adults (the *Population Cohort*) in the trial communities for 3 years

Secondary Objectives:

- Measure the impact of the two intervention packages on the following:
  - HIV incidence over the first, second, and third years of follow-up
  - Community viral load (subject to funding)
  - ART adherence and viral suppression (subject to funding)
  - Anti-Retroviral (ARV) drug resistance (subject to funding)
  - HSV-2 incidence
  - Uptake of HIV testing and retesting over the entire study period
  - ART screening and uptake
  - Time between HIV diagnosis and initiation of care
  - Retention in care
  - HIV disease progression and death
  - ART toxicity based on clinic records
  - Sexual risk behavior
  - Case notification rate of tuberculosis

- HIV-related stigma
  - Uptake of PMTCT
  - Uptake of male circumcision
- Carry out case-control studies to examine factors related to:
  - Uptake of HIV testing during the first round of home-based testing in Arms A and B
  - Uptake of immediate treatment in Arm A
  - Uptake of HIV testing during the second round of home-based testing in Arms A and B
- Use qualitative methods to:
  - Assess popular understanding of HIV testing and treatment at study initiation and during implementation
  - Evaluate the acceptability and functioning of the Community HIV-care Providers (CHiPs) in Arms A & B
  - Evaluate the acceptability of interventions and barriers to access in Arms A & B
  - Document the effect of the interventions on social networks, stigma, sexual behavior, alcohol use, gender-based violence, HIV identity, other HIV prevention options and community morale
  - Evaluate the process and challenges of community consultation and applying ethical principles
- Measure the burden experienced by local health centers due to implementation of the intervention in the community
- Measure the incremental cost of the two intervention packages through systematic recording of costs in intervention and control communities
- Estimate the effectiveness and cost-effectiveness of the intervention packages and alternative packages, both in the chosen study populations and in other populations by fitting mathematical models based on the empirical data from the trial, including data related to cost.

Study Sites: The study is expected to be implemented in the communities identified below.

- The study communities in Zambia are spread across 4 provinces and 6 districts. Each community is the catchment population of a government health facility.
  - Chimwemwe and Ndeke in Kitwe District (Copperbelt Province)
  - Chipulukusu and Chifubu in Ndola District (Copperbelt Province)
  - Makululu and Ngungu in Kabwe District (Central Province)
  - Chawama, Chipata and Kanyama in Lusaka District (Lusaka Province)
  - Maramba and Dambwa in Livingstone District (Southern Province)
  - Shampande in Choma District (Southern Province)

- The study communities in South Africa are located in the Cape Metro District and Cape Winelands District of the Western Cape Province. As above, the communities are defined by the catchment population of a government health facility.
  - Delft South (Metro District)
  - Kuyasa (Metro District)
  - Luvuyo (Metro District)
  - Town II (Metro District)
  - Ikhwezi (Metro District)
  - Bloekombos (Metro District)
  - Dalevale (Cape Winelands District)
  - Wellington (Cape Winelands District)
  - Cloetesville and Idas Valley (Cape Winelands District)

HPTN 071
Population Effects of Antiretroviral Therapy to Reduce HIV Transmission (PopART): A cluster-randomized trial of the impact of a combination prevention package on population-level HIV incidence in Zambia and South Africa

OVERVIEW OF STUDY DESIGN AND RANDOMIZATION SCHEME

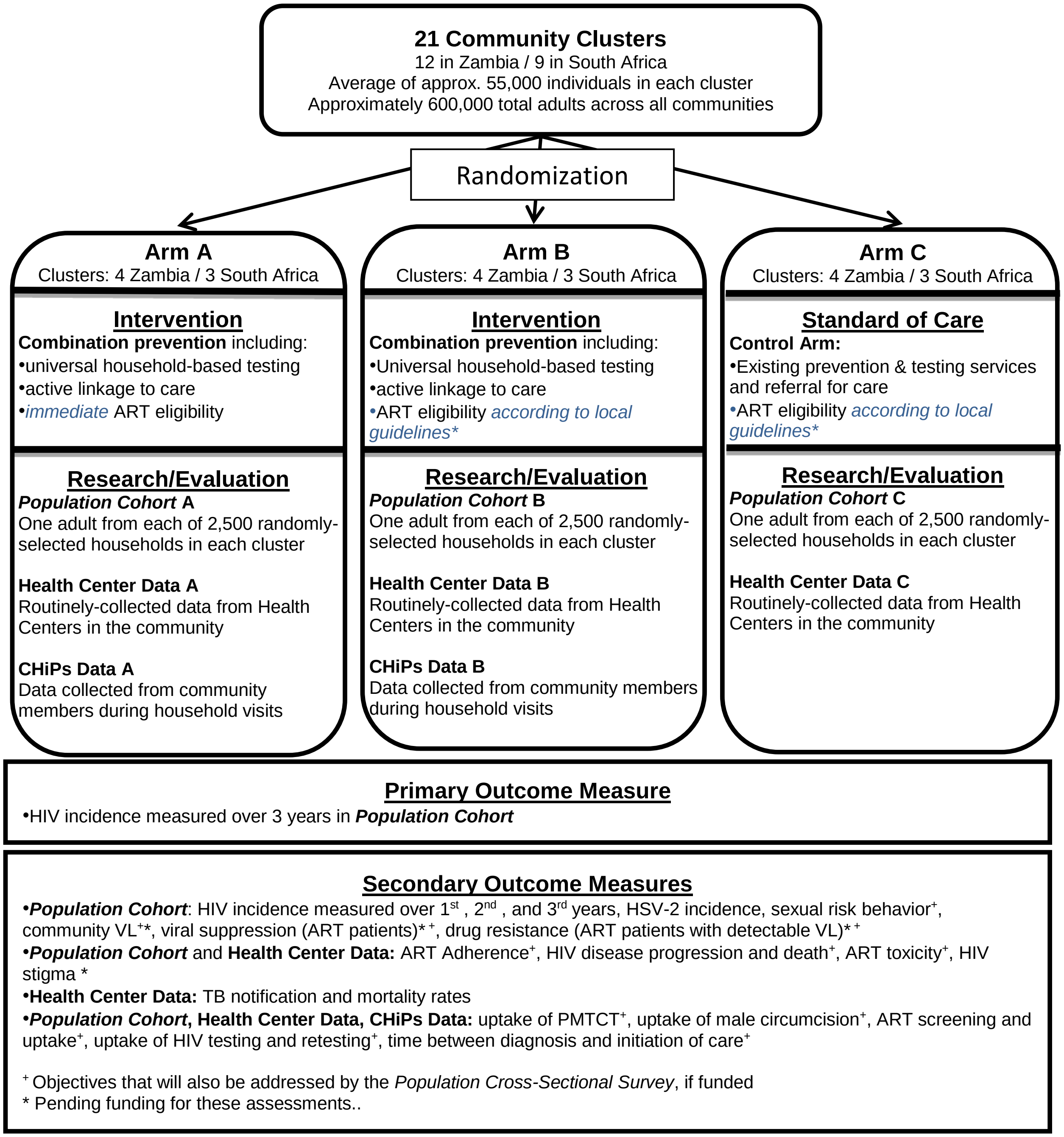

* In initial phases of the study, treatment according to local guidelines; with protocol v.3.0, and once funding is secured, immediate eligibility for ART (after participant consent if offered outside of local guidelines).

Note: Qualitative and case-control studies that will be undertaken to interpret and inform the results from the objectives above are not included in this diagram for simplicity, but are fully described in subsequent sections.

1.0 INTRODUCTION

1.1 Background and Prior Research

The global health burden associated with human immunodeficiency virus (HIV) infection continues to grow, with an estimated 33 million people living with HIV, including 22.5 million adults and children in sub-Saharan Africa. While several countries have reported reductions in HIV prevalence, prevalence remains extremely high, especially in Southern Africa which continues to experience severe, generalized epidemics with persistently high rates of HIV incidence [1].

While considerable progress has been made in expanding the coverage of antiretroviral treatment (ART) for patients living with advanced disease (CD4 cell count < 200 cells/µL), a large proportion of HIV-infected individuals who need treatment are not yet receiving it . ART is a lifelong commitment. Therefore, ongoing treatment costs continue to escalate as more patients require ART. There are 2.5 new HIV infections for every HIV-infected patient commencing ART, meaning that there is an ever-expanding pool of patients who will need treatment in the future [1]. Unless the number of new infections can be steeply reduced, it will be increasingly difficult and costly to provide ART for all those who need it [2, 3]. For these reasons, effective HIV prevention has become an even more pressing priority in the era of ART roll-out.

There is increasing recognition that a combination of prevention methods will be needed to bring HIV transmission under effective control in the most severely affected countries, and combination prevention programs are being developed to meet this need [4, 5]. These may involve the provision of proven prevention methods, such as male circumcision[6] and PMTCT [7, 8], a range of behavioral and biomedical interventions specially targeted at those most at risk of infection, and expanded testing and treatment for individuals found to be HIV-infected [9-11]. Early treatment of HIV-infected individuals has been shown to reduce transmission to their sexual partners by 96% [11] and, more recently, to have significant beneficial effects upon the health of infected individuals themselves [12]. While such strategies are based on sound epidemiological principles, they have not been adequately evaluated in the field [13, 14] and there are no data on their effectiveness or cost-effectiveness in reducing HIV incidence at population level. In particular, identifying specific groups at high risk of HIV infection and providing specially targeted interventions for them is likely to be very difficult to implement on a national scale, and is potentially stigmatizing.

1.2 Rationale

Since the principles of combination HIV prevention were formulated, there has been new interest in the potential impact of universal testing and treatment (UTT) interventions [15]. This concept represents a paradigm shift in HIV prevention, since it focuses on identifying and intervening in HIV-infected individuals in preference to the much larger uninfected population [16]. Even in the high resource environment of the United States, only about a quarter of HIV-infected persons know their status, are linked to care, and are suppressed with ART [17-19]. Mathematical modeling has indicated that if a high proportion of the

population can be tested, with those found to be HIV-infected offered immediate ART, HIV infection could be reduced substantially within two years, and potentially eliminated as a public health problem in the longer term [20-27]. While challenging to deliver [28, 29], this approach would nevertheless have major advantages in terms of simplicity and universality, potentially reducing the need for interventions targeting specific groups at high risk of infection, who are often stigmatized, as well as bringing likely clinical benefit to those infected with HIV [30-33].

To guide health policy, data are needed on the population-level impact of different approaches to HIV prevention. We propose to evaluate a combination UTT HIV prevention package that includes universal voluntary HIV testing and counseling, provision of condoms, STI treatment, the offer of male circumcision to men who are HIV uninfected, referral to PMTCT services, and the offer of immediate ART for all those identified as HIV-infected. We will test this package in a cluster-randomized trial in 21 communities in Zambia and South Africa, and measure its impact on HIV incidence in the general population by following a randomly-selected cohort of adults for 3 years. In order to measure the additional impact of offering immediate ART to those who are HIV-infected, the study commenced with three treatment arms: Arm A receiving the full UTT intervention described above, Arm B receiving the full intervention except that ART was provided according to current local guidelines, and Arm C acting as a control arm receiving standard of care services. With version 3.0 of the protocol and once funding has been secured, Arms B and C will also receive immediate eligibility for ART, either through revision of local guidelines, or as specified by this protocol if local guidelines have not been revised by the time version 3.0 is approved for implementation by local authorities.

Data from the trial will be combined with cost data and mathematical models to estimate the cost-effectiveness of the UTT intervention and alternative intervention approaches in these and other populations.

1.2.1 The HIV Epidemic in Sub-Saharan Africa

Sub-Saharan Africa bears over two-thirds of the worldwide burden of HIV infection [1]. The HIV epidemic in this region has had a devastating effect on morbidity, mortality and national economies, as well as wider societal effects. HIV infection is also a strong risk factor for tuberculosis (TB); people living with HIV who are also infected with TB are about 21–34 times more likely to develop TB disease compared with those who are HIV-negative. Additionally, approximately 24% of global TB deaths are estimated to be HIV-associated, adding to the health burden associated with HIV infection[34] .

While recent declines in HIV prevalence and incidence have been observed in several African countries, HIV prevalence remains extremely high in many parts of the region [1]. In particular, Southern Africa remains severely affected, with an estimated 11.3 million people living with HIV infection, with extensive, generalized HIV epidemics and very high HIV prevalence in most countries. Zambia and South Africa are among the most severely-affected countries with an estimated 980,000 living with HIV in Zambia [35] and an estimated 5,600,000 living with HIV in South Africa [36].

Despite the rapid expansion of access to ART (with an estimated 6.6 million people on ART by the end of 2010), an additional 10 million people are in urgent need of ART in accordance with current World Health Organization (WHO) treatment guidelines[37]. Both countries are making good progress towards achieving the targets set in the new guidelines regarding CD4 cell count and ART regimens however, there are practical constraints including access to laboratory testing, consistent drug supplies and linkage into care. The HPTN 071 study will work with the local Departments of Health and Ministries of Health along with the PEPFAR implementing partners, to utilize additional resources to strengthen the health systems.

Globally, it is estimated that there are 2.5 new HIV infections for every patient started on ART. This means that there is an ever-increasing pool of untreated HIV-infected individuals who will need treatment in the next few years in addition to those already on treatment. It is clear that there will be major difficulties in sustaining treatment provision for a continuously expanding number of HIV-infected patients. The expansion of ART services needed in the coming years has increased the urgency for identifying more effective interventions for HIV prevention. Unless HIV incidence can be reduced, an estimated US$35 billion will be needed per year by 2030 to deliver ART to 80% of eligible patients (CD4 <350 cells/µL) in resource-limited settings[38].

1.2.2 HIV Prevention Methods

Very few HIV prevention methods have been shown to be effective in randomized, controlled trials [39, 40]. Behavior change messages have been central to most national acquired immunodeficiency syndrome (AIDS) control programs in Africa, and changes to safer sexual behavior are assumed to have contributed to the reductions in HIV prevalence in Uganda, Zimbabwe, and other countries [41, 42]. However, there is a dearth of evidence from rigorously-designed trials on what specific behavioral interventions bring about the required behavioral changes leading to a reduction in HIV incidence [40]. Similarly, while HIV counseling and testing provide the gateway to key treatment and prevention services, evidence of their effects on behavior and HIV risk is inconclusive [43-45].

In contrast, stronger evidence of effectiveness is available for some biomedical interventions. Male circumcision was shown to reduce HIV incidence by around 60% in three trials in Kenya, South Africa and Uganda [46-48]. Safe services for male circumcision have been recommended for wide-scale roll-out by WHO and the United Nations Programme on HIV/AIDS (UNAIDS), but progress in implementation in many countries has been slow [49, 50]. HIV transmission is known to be facilitated by other sexually transmitted infections (STIs) [51]. One trial in Tanzania showed that improved STI treatment services reduced HIV incidence in the general population [52]. Other trials of a variety of STI interventions in different epidemiological settings have failed to show an impact on HIV incidence [53].

Despite the promising results of the RV144 vaccine trial in Thailand [54], there is general agreement that an effective HIV prophylactic vaccine will not be available for many years [55, 56]. However, the CAPRISA004 trial, reported in 2010, showed that a vaginal gel containing the antiretroviral drug, tenofovir, used periodically, reduced HIV incidence by

39% among women in South Africa [57]. Vaginal microbicides have been shown to be highly acceptable in a wide range of studies, leading to optimism that a product of proven efficacy may achieve substantial coverage [58, 59]. However, further confirmatory and other trials will be needed before this and other microbicides are licensed and available for use in large-scale prevention programs, and it is unclear whether coverage and impact would be sufficient to bring the very high rates of HIV incidence in Southern Africa under control.

Further promising data came from the iPrEx trial in 2010, showing that pre-exposure prophylaxis (PrEP) using a combination of two antiretroviral drugs (emtricitabine and tenofovir) among men who have sex with men (MSM) reduced HIV incidence by 44% in a multi-center study [60]. More recently, two trials of the effects of PrEP on heterosexual transmission among men and women reported a significant protective effect [61, 62], but two trials found no effect [62, 63]. It is currently unclear how the results of these trials will be translated into revised WHO and local guidelines. While such interventions may have a place in HIV prevention programs, particularly for discordant couples, sex workers, and other groups at particularly high risk, the feasibility of wide-scale delivery of PrEP and its population-level impact have been questioned.

Given the limitations in current HIV prevention methods, there is increasing acceptance that effective HIV control in the most severely affected countries in Southern Africa is likely to require the concerted delivery of a combination of partially effective interventions. Combination prevention is therefore becoming the preferred approach to the prevention of HIV infection [4, 5, 9, 64]. Combination prevention packages may consist of different components, including expanded HIV testing and counseling, male circumcision, interventions to promote safer behavior, enhanced PMTCT services, expanded treatment services for HIV-infected patients, and special interventions targeted at groups at increased risk of infection, such as those in HIV-discordant partnerships, injection drug users, commercial sex workers, truck drivers, MSM and others. The emphasis on targeted interventions emerges from the concept of “Know your Epidemic” [65], whereby information on the roles of different modes of transmission and different risk groups in local epidemics helps to guide the most efficient application of limited prevention resources for maximal reduction of HIV transmission.

While the epidemiological basis for combination prevention is strong, there is a need for empirical field studies to evaluate the operational performance of such interventions when applied on a large scale, and to measure their impact on HIV incidence at population level. This would provide valuable data for policy makers who must choose the most appropriate intervention approaches to include in national prevention programs. HPTN 071 will provide such data using a rigorous, cluster-randomized trial design that tests the efficacy of specific combination prevention packages that are strongly supported by epidemiological and modeling data.

One disadvantage of combination prevention strategies that require careful targeting of special groups is that they may be difficult to implement on a wide scale [66]. Optimal implementation of such approaches requires availability of baseline data to define the transmission dynamics and the size and role of different risk groups, development of programs designed specifically for those groups, intensive community liaison work to gain

the trust of groups that are often marginalized, stigmatized, or highly mobile, and the management and monitoring of these separate programs. While this may be achievable in demonstration projects in a small number of communities, it may prove very challenging in the context of national roll-out of such programs. In contrast, because the main intervention in HPTN 071 is universal and is offered to the entire community, it will obviate the need for specially-targeted interventions for different risk groups, should help to avoid stigmatization, and should encourage community-wide support for HIV prevention and care.

1.2.3 Anti-Retroviral Therapy (ART) for HIV Prevention

Incident HIV infections necessarily result from transmission of the virus between an HIV-infected index case and an HIV-uninfected individual. This simple observation has led to an increasing interest in interventions focused on HIV-infected persons to prevent transmission to their contacts; this is referred to as positive prevention.

HIV viral load is the key determinant of viral transmission, as demonstrated clearly in observational studies of sexual transmission among HIV-discordant couples; in those studies, no transmission was seen when the index case had a plasma viral load below 1000 copies HIV ribonucleic acid (RNA)/ml [67, 68]. By reducing plasma viral load to undetectable levels (<50 copies HIV RNA/ml), it is assumed that ART will also suppress viral burden in the genital tract to levels at which transmission is unlikely to occur [69, 70]. Although vertical HIV transmission occurs via a different route, proof of concept is provided by trials of PMTCT, which have demonstrated that HIV transmission from mother to child before, during, or after delivery is largely prevented by ART [71-73]. Of even more relevance to sexual transmission, are results of the HPTN 052 trial [74]. In this large, Phase III trial, the effects of early ART on transmission were investigated in 1750 HIV-serodiscordant couples. HPTN 052 was powered to determine the impact of immediate ART initiation for the HIV-infected partner (at CD4 cell counts >350 cells/µl and <550 cells/µl) on HIV transmission, compared with ART initiation according to standard treatment guidelines [74]. This trial was unblinded early, after demonstrating a 96% reduction in HIV transmission to sexual partners in the early treatment arm, as well as significant reductions in morbidity in HIV-infected index cases [74].

The increasing proportion of HIV-infected patients on ART has likely made some contribution to falling HIV prevalence in some countries. However, ART as currently delivered in resource-poor settings is unlikely to have a substantial effect on HIV transmission because of limited coverage of HIV testing, delays in provision of treatment and – importantly – because much HIV transmission occurs before HIV-infected index cases reach CD4 levels defined by current treatment guidelines [75] . The UTT strategy in this study aims to overcome these limitations by ensuring that all HIV-infected individuals are diagnosed as early as possible, and are provided with ART to lower their viral loads and minimize the risk of transmission. In addition, these interventions will provide important individual-level benefits in terms of reductions in morbidity and mortality among HIV-infected individuals.

Ecological studies have reported promising outcomes of UTT-type interventions at a population level in North America. Among MSM in San Francisco, where 72% uptake of

HIV counseling and testing was followed by 95% acceptance of immediate ART for those identified as HIV-infected, an observed reduction in mean and total community viral load was accompanied by a significant decrease in new HIV diagnoses from 798 (in 2004) to 434 (in 2008)[76, 77]. Similarly, among injection drug users in British Columbia, a study of expanded testing and treatment between 1996 and 2009 showed a 52% reduction in estimated HIV incidence [78]. However, the direct relevance of these findings in concentrated epidemics in North America to generalized epidemics in Southern Africa is unclear. While these data are promising, they are subject to many limitations, as they rely on incidence estimates based on diagnosed cases of HIV, and time-trends in HIV epidemics are notoriously difficult to interpret. No such data are available from sub-Saharan Africa, where the need is greatest.

Interest in the UTT approach to HIV prevention has grown following the publication of mathematical modeling studies suggesting that the approach has the potential to substantially reduce and possibly eliminate HIV transmission at a population level in sub-Saharan Africa. In the much-discussed Granich model [26], ART for all individuals with a CD4 cell count <350 cells/µL is predicted to reduce population HIV incidence by 30%, based on assumptions about the distributions of plasma viral load and CD4 cell count. In that model, UTT is predicted to reduce the reproduction number to below 1, suggesting that elimination of HIV infection as a public health problem may be possible. However, concerns have been raised about the validity of the assumptions underlying this model, with considerable skepticism about the ability to treat everyone identified as HIV-infected in settings where ART coverage for individuals with CD4 < 200 cells/µL is currently below 50% [79]. The feasibility of the UTT strategy is compromised by weak health systems, insufficient numbers of health care personnel, potential problems with lifelong treatment adherence, drug toxicity, drug resistance and the need for durable second and third-line treatment regimens. The impact of a UTT intervention will also depend on the proportion of transmission events that occur during acute HIV infection, since most patients are not likely to be diagnosed during this highly infectious phase prior to seroconversion [80-82]. In addition, the feasibility and acceptability of regular HIV testing of whole populations, acceptance of immediate ART irrespective of disease stage or symptomatology, and the extent of behavioral risk disinhibition [83] will all be critical determinants of the ultimate success of a UTT intervention. Concerns regarding community acceptability, protection of voluntariness, avoidance of stigma, and preservation of human rights must also be addressed.

Clearly, empirical field studies are needed to test the practical performance of UTT interventions and to measure their impact on HIV transmission. Universal testing is a key component of the UTT strategy and provides the framework for delivering proven preventive interventions to those who are HIV-uninfected at the same time as offering immediate treatment to those who are identified as HIV-infected. Therefore, UTT is fundamentally a combination prevention strategy - this is the approach that will be evaluated in this rigorously-designed, cluster-randomized trial in two severely affected countries in Southern Africa.

Because of uncertainties regarding the additional impact provided by offering immediate ART (compared to offering ART according to current local guidelines) the trial commenced with three arms comparing: a full combination prevention package including

UTT (Arm A), a UTT package including all components of the intervention except for immediate ART irrespective of CD4 cell count (Arm B), and a control arm in which the population received standard of care (Arm C). Research findings published in 2015 [84], showed that immediate ART has substantial clinical benefits for the health of HIV infected persons. WHO guidelines have been set to recommend immediate ART eligibility before the end of the 2015 [12]. Changes to local, country-specific guidelines are expected to follow soon thereafter. These antipated changes in guidelines mean that by the time findings from this trial are available, there will be little global interest for study results comparing immediate vs. delayed eligibility for ART. Because it is not known how long it will take for local guidelines to offer immediate ART, and so to ensure that the study produces findings of the greatest relevance to global public health, and to ensure optimal clinical management of all HIV infected individuals residing within the HPTN 071 (PopART) communities, protocol version 3.0 will offer immediate eligibility for ART to all health center clients in Arms B and C once funding to support this change has been secured. As has been the case in Arm A from the start of the trial, health center clients in Arms B and C will be required to provide consent if the study is making immediate ART available to them outside of local guidelines. If local guidelines change to offer immediate ART as standard care either before or after implementation of version 3.0 of this protocol, then study consent will not be required for anyone to initiate ART from the time the change in guidelines.

1.2.4 Innovation

The PopART intervention moves the HIV prevention field forward in several important ways.

First, this will be one of the first studies to evaluate the impact of the UTT prevention approach on population-level HIV incidence in sub-Saharan Africa. The concept of UTT for HIV control in Africa is relatively new; the landmark modeling paper of Granich et al appeared in 2009 [85]. While the epidemiological rationale for the intervention in HPTN 071 is strong, the approach is controversial. Many question whether it is wise to ask health systems that are struggling to deliver ART even at low treatment thresholds (e.g., at CD4 <200 cells/µL) to provide and supervise a program of immediate ART that goes well beyond the revised WHO guidelines. Nevertheless, there are many arguments in favor of using this approach for HIV prevention:

- Combination prevention incorporating UTT is currently the only strategy that has the potential to eliminate HIV infection in the longer term in the most severely affected countries.
- While initial costs may be high, model estimates suggest that the intervention will be cost-saving in the long run, especially if averted costs of hospital treatment for HIV-related disease are taken into account [27, 86, 87].
- Unless HIV incidence is reduced substantially, ART treatment services will have to meet an ever-increasing case-load and this burden will greatly outweigh the initial costs of implementing UTT.
- Those currently not treated because their CD4 cell counts do not meet ART eligibility criteria will in any case need to be treated in a short time; meanwhile they are at risk of transmitting the virus to partners, thus increasing the future care

burden. Moreover, patients are often lost to follow-up before they have CD4 cell counts below treatment thresholds.

- Compelling research data [12], released in 2015 show that immediate ART confers substantial health benefit for HIV-infected patients irrespective of CD4 count and immediate ART is poised to rapidly become standard of care. In light of these developments, the study team feels it is important to adopt immediate offer of ART for all HIV infected individuals attending health care facilities within all clinics involved in the HPTN071 (PopART) trial. This will be offered ahead of local guidelines if protocol version 3.0 is approved (and funding to support the change can be secured) in advance of a change in local guidelines. Following this protocol change, the trial will continue to address a very important question: how much effect does a UTT intervention (including house-to-house testing, linkage to care and adherence support) have on population-level HIV incidence compared to standard of care.
- UTT reduces the complexity of ART delivery, since all HIV infected patients can be managed through the same clinical pathway. Simplified approaches to testing, treatment and monitoring will partly offset the burden imposed by greater patient numbers.
- Current treatment approaches often lead to severe delays in onset of treatment, so that CD4 cell counts are often extremely low when ART commences. This leads to greater morbidity, mortality and ongoing transmission, with the associated costs of additional health care for individuals who go on to present with HIV-related illnesses.
- UTT is also projected to have a major impact on the incidence of TB, which often occurs at relatively high CD4 cell counts, thus reducing morbidity, mortality and the burden on overstretched TB control programs [88, 89].

Second, the UTT intervention will be delivered as part of a combination prevention package that also includes counseling, referral for PMTCT services, and other proven preventive interventions, including male circumcision. We argue that UTT is by its nature a combination prevention method, in that delivery of universal HIV testing and counseling itself comprises an important prevention package that has been shown to alter reported sexual risk behavior, especially when couples are tested together [90]. In addition, universal testing provides the framework for delivery of prevention services to both HIV-uninfected and HIV-infected individuals. This trial will measure the overall impact of the UTT prevention package on HIV incidence, rather than measuring the impact of any particular component of the intervention. The three-arm study design will make it possible to estimate the additional impact provided by offering immediate ART (in addition to the other components of the package). Despite the change to Arms B and C to offer immediate ART that will occur with protocol version 3.0, analysis of study results is still expected to provide insight into the differential value of immediate ART since approximately two years of the intervention will have been implemented with Arm B and C clinics offering ART based upon CD4 cell count or disease progression. Furthermore, empirical data from the trial on the operational performance of individual intervention components and the measured impact of the two intervention packages (Arms A and B) will be assessed using mathematical models; these models will also be used to explore the projected effects of alternative combinations. The rigorous data generated on the overall

effect of the packages, together with these model projections, will be of considerable value to policy makers. The change to offering immediate ART in all three arms of the trial will be incorporated into the statistical analysis plan as discussed in Section 7.

Third, we believe that the proposed intervention package, if successful, will provide a conceptually simple approach that avoids some of the limitations of other combination prevention approaches that emphasize targeting of special interventions to groups at high risk. As we have argued above, while such interventions may still be needed, formulating and delivering locally-appropriate packages on a national scale would be extremely challenging. In contrast, the PopART intervention can potentially be implemented on a wide scale using a relatively uniform and standardized approach, as has been the case for other major public health interventions such as use of impregnated bednets for malaria prevention.

Fourth, the universal test and treat strategy being investigated in the HPTN 071 study is likely to have a significant effect on TB [91, 92]. On an individual level it is well established that TB is increasingly common at lower CD4 cell counts. However, the risk of developing TB increases rapidly after acquisition of HIV[93]. ART has been shown to reduce the risk of developing TB in individuals by increasing CD4 cell counts[94]. The effects of ART on TB at community level are not known. The study will assess impact of the intervention on TB as determined from health center records and so begin to address this important question.

If additional funding can be identified, the following would also be addressed:

Fifth, in addition to measuring the primary endpoint of our cluster-randomized trial design (population-level HIV incidence), we will also measure the impact of the intervention on community viral load if funding for these activities is available. Despite some limitations, community viral load has been proposed as a valuable indicator for assessing the effect of treatment-based HIV interventions, and by making comparisons across our study communities, we hope to investigate how this indicator relates to the HIV incidence measure.

Sixth, the primary strategy to prevent mother to child transmission of HIV is the provision of maternal and neonatal anti-retroviral (ARV) prophylaxis [7]. Scale-up of this intervention has taken place across sub-Saharan Africa. Despite this, there is evidence of on-going transmission to children in the continent, with up to 12% of children whose mothers received some form of PMTCT prophylaxis testing positive for HIV[72, 95, 96]. ART during pregnancy and breastfeeding provides more effective protection against mother-to-child transmission of HIV than standard short course ART regimens[97] which are still being implemented in many African countries. The PopART interventions, through immediate provision of ART, have the potential for a significant impact on HIV-free survival in children through earlier initiation of maternal HIV treatment. They may also lead to an improvement in overall child survival through potential secondary benefits such as improved maternal health, changes in health-seeking behavior, improved care, and increased resources for better nutrition for both HIV infected mothers and their children.

Seventh, The development of drug resistant HIV infection will be compared between the three arms (dependent on additional funding). One of the key safety concerns about the population level implementation of a UTT approach is evolution of HIV resistance to ART. This will be examined amongst the HIV+ individuals enrolled into the PC who have detectable viral load measurements whilst on ART, as well as baseline viral genotyping for those who seroconvert through the study period to document the prevalence of transmitted drug resistant infection.

Our preliminary modeling indicates that the intermediate intervention (Arm B) should have a substantial impact on HIV incidence, but that a much larger impact should be seen in Arm A (see Section 2.3). It is anticipated that guidelines will change to universal treatment independent of CD4 count during the course of the study, and implementation of version 3.0 of the protocol will ensure that the study offers universal treatment to all arms even if local guidelines have not yet changed. From this stage on, the interventions provided in Arm B and Arm A will be identical. The sooner this change occurs, the smaller the difference in impact on HIV incidence is predicted to be between Arms A and B. The difference between Arms A and C is predicted to remain large due to the impact of household testing and enhanced linkage to care. The three-arm study design will allow us to confirm these projections. Detailed data on the costs of these intervention packages, combined with the impact data from the trial, will provide critical policy guidance on the cost-effectiveness of combination prevention strategies and the priority that should be given to earlier treatment. Operational data from the trial will provide valuable information on the practical issues involved in delivering such programs to scale. The modeling projections will be updated regularly as the trial progresses, so that investigators, reviewers, stakeholders, and the Data Safety and Monitoring Board can assess up-to-date projections. Specifically, modeling projections will be adjusted in accordance with changes in the criteria for ART initiation, either through changes in local guideline or through this protocol amendment.

2.0 STUDY OBJECTIVES AND DESIGN

2.1 Primary Objective

- The primary objective of this study is to measure the impact of the two intervention packages on HIV incidence by enrolling and following a random sample of adults (the *Population Cohort*) in the trial communities for 3years.

2.2 Secondary Objectives

The secondary objectives of the study are to:

- Measure the impact of the two intervention packages on the following:
  - HIV incidence over the first, second, and third years of follow-up
  - Community viral load (subject to funding)

- ART adherence and viral suppression (subject to funding)
- ARV drug resistance (subject to funding)
- HSV-2 incidence
- HIV disease progression and death
- ART toxicity
- Sexual risk behavior
- Case notification rate of tuberculosis
- HIV-related stigma
- Uptake of PMTCT
- Uptake of male circumcision
- ART screening and uptake
- Uptake of HIV testing and retesting
- Time between HIV diagnosis and initiation of care
- Retention in care

- Carry out case-control studies to examine factors related to:
    - Uptake of HIV testing during the first round of home-based testing in Arms A and B
    - Uptake of immediate treatment in Arm A
    - Uptake of HIV testing during the second round of home-based testing in Arms A and B
- Use qualitative methods to:
    - Assess popular understanding of HIV testing and treatment at study initiation and during implementation
    - Evaluate the acceptability and functioning of the Community HIV-care Providers (CHiPs) in Arms A & B
    - Evaluate the acceptability of interventions and barriers to access in Arms A & B

- Document the effect of the interventions on social networks, stigma, sexual behavior, alcohol use, gender-based violence, HIV identity, other HIV prevention options and community morale
- Evaluate the process and challenges of community consultation and applying ethical principles

- Measure the burden experienced by local health centers due to implementation of the intervention in the community
- Measure the incremental cost of the two intervention packages through systematic recording of costs in intervention and control communities
- Estimate the effectiveness and cost-effectiveness of the intervention packages and alternative packages, both in the chosen study populations and in other populations by fitting mathematical models based on the empirical data from the trial, including data related to cost.

2.3 Study Design

The two intervention packages will be implemented in study communities and their impact on population-level HIV incidence will be evaluated using a cluster-randomized trial design.

A total of 21 study communities (12 in Zambia and 9 in South Africa) will be selected. The *cluster* or *community* for the purposes of this trial is defined as the catchment population of a local health unit (through which the intervention is delivered), and corresponds to a total population of between about 20,000 and 150,000 individuals (average size of approximately 55,000). These 21 communities will be formed into 7 matched triplets, with 4 matched triplets in Zambia and 3 in South Africa. In each matched triplet, one community will be randomly selected to receive the full intervention (Arm A), a second community will receive the full intervention except that ART will be offered according to current local guidelines (Arm B) and the third act as a control community receiving standard of care. Within each country, communities will be matched based on the best available estimates of HIV prevalence, as described in Section 4.1, with the aim of minimizing the between-community variance in baseline HIV incidence within matched triplets. In addition, restricted randomization will beused to ensure overall balance in cluster size, ART uptake and mean HIV prevalence across the study arms. The community randomization scheme is represented graphically in Figure 1.

As described above, implementation of the changes in protocol version 3.0 will result in the offer of immediate ART in all three arms.

The primary outcome of the study, HIV population-level incidence, will be measured through longitudinal follow-up of a cohort of approximately 2,500 adults in each community who consent to participation, drawn from a randomly selected list of households (the *Population Cohort*).

At the end of the third year of the intervention, to coincide with the 36 month follow-up visit of the *Population Cohort*, a random sample of houses will be selected (excluding houses in the *Population Cohort*) and visited by field staff to complete a final survey. Because this *Population Cross-Sectional Survey* will be a one-time survey, data obtained from these individuals will be uncontaminated by the potentially biasing effects of longitudinal cohort participation, and will provide additional data on community viral loads and other process measures.

Figure 1- Community Randomization Scheme, Zambia and South Africa

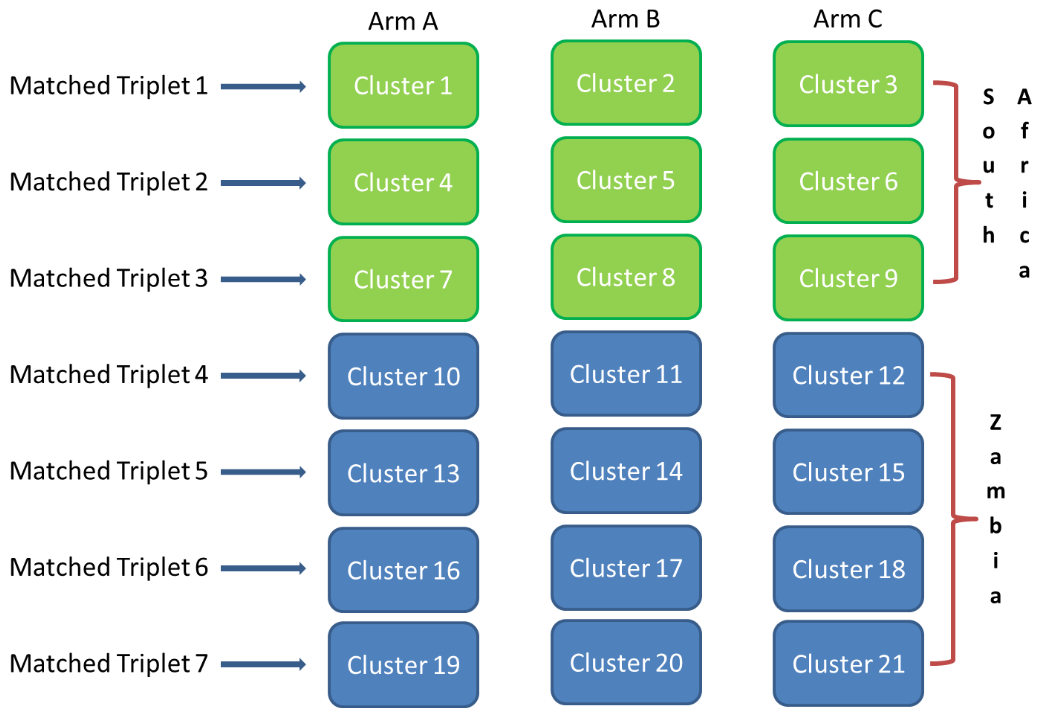

2.4 Timing of Deployment of Intervention and Research Components

The total duration of the study will be approximately 6 years. During the first year, the protocol will be finalized, study procedures defined and manuals of operations developed, plans and procedures developed with community and implementing partners, and study staff trained. Preliminary qualitative work will also be conducted in the communities to prepare for study initiation. Shortly prior to deployment of the intervention, households in each community will be mapped. Early in the second year, the intervention, implemented by the *CHiP teams* (home-based testing and linkage to medical care in the health centers) will be deployed in Arms A and B at the same time that the *Population Cohort* is enrolled by the *research teams* for evaluation in all arms. It is expected that the first round of deployment of the intervention will take approximately one year, as will enrollment of the *Population Cohort*.

CHiP teams will stay engaged in the community throughout the intervention period, but will return to all households to repeat rounds of home-based testing 12, 24, and 36 months after the initial round of testing. Similarly, research teams will conduct evaluation visits to the homes of the *Population Cohort* members at enrollment, 12, 24 and 36 months. Whether or not an individual has been seen by a CHiP team will be asked during the *Population Cohort* survey to help interpret the data, particularly with regards to uptake.

During the first two years of intervention, an interim evaluation will take place to determine whether to continue with the 36 month follow-up of the *Population Cohort* and the fourth year of intervention (see Figure 2). The evaluation will consider milestones such as uptake of the intervention and indicators of futility and will be described in the Statistical Analysis Plan.

If funded, the *Population Cross-Sectional Survey* survey will occur at the same time as the 36-month *Population Cohort* visits. Case-Control studies and qualitative research by the research teams will occur at intervals during the entire follow-up period.

Analysis and reporting of the primary outcome for the main HPTN 071 study (HIV incidence) is not expected to occur for a considerable time after completion of the 36-month follow-up visits for the *Population Cohort.* This is because of the very large sample size of the PC, the need to perform HIV testing both in-country and at the HPTN LC, and the need to complete QA testing, including confirmation of HIV seroconversion, prior to data analysis. This timeline is represented graphically in Figure 2.

Figure 2- Timing of Deployment of Intervention and Research Components

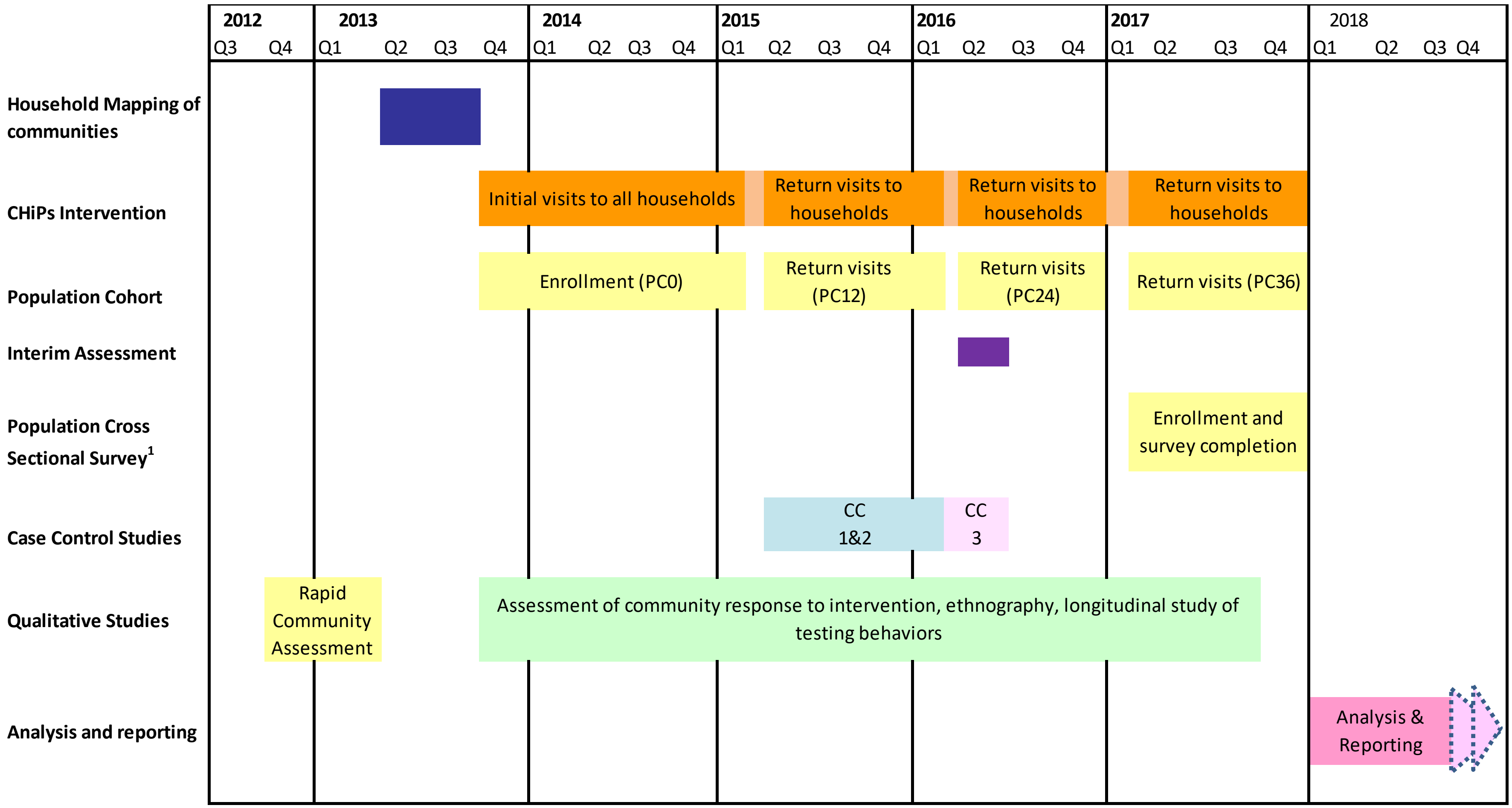

[1] Requires additional funding; to be described in and conducted according to an ancillary protocol

2.5 Cross-Sectional HIV Incidence Estimation

In HPTN 071, HIV incidence estimates will be based on longitudinal assessment of HIV seroconversion. A robust, multi-assay approach for cross-sectional HIV incidence determination was recently validated for subtype B HIV. This algorithm uses a combination of two serologic assays (the limited antigen avidity assay [LAg] and a second antibody avidity assay), as well as two non-serologic biomarkers (CD4 cell count and HIV viral load) to identify individuals who are likely to be recently HIV-infected at the time of sample collection[98]. An alternate multi-assay algorithm has been developed that includes an HIV diversity measure rather than CD4 cell count[99]; an advantage of this alternate algorithm is that it can be performed entirely using stored plasma samples. Work is underway to optimize a similar multi-assay algorithm in subtype C HIV, the prevalent subtype in South Africa and Zambia.

HPTN 071 provides an opportunity to apply these methods to achieve several objectives, including estimation of HIV incidence at baseline (prior to implementation of the study interventions) and comparison of HIV incidence estimates based on longitudinal and cross-sectional assessments. These assessments may be performed using stored plasma samples if an appropriate multi-assay algorithm for subtype C is developed and validated and funding is obtained.

3.0 STUDY INTERVENTION

3.1 Implementation Team Experience

The implementing team that will carry out this program has extensive prior experience in conducting community randomized research, including household-level incidence assessment, particularly in the conduct of the Zambia-South Africa TB and AIDS Reduction (ZAMSTAR) trial in the same communities that have been chosen for this study. The leadership and much of the field team from the ZAMSTAR trial remain actively engaged in the communities and will be able to build on their knowledge of and acceptance within these populations when rolling out the community interventions described below.

In addition, extensive effort has been put into developing in-country coordination structures for the trial. In both countries, there have been and will continue to be ongoing dialogues with national, provincial and district Departments of Health, PEPFAR secretariats, USAID and CDC HIV treatment and prevention representatives, other implementing partners, and community representation organizations. The study team has also developed sustainability plans and community engagement plans.

3.2 Description of CHiP Teams

As described above, seven communities will be randomized to receive the full intervention and seven will be randomized to receive the intervention except with eligibility for ART determined by current local guidelines. In these two types of intervention communities (Arms A and B), delivery of the intervention will be carried out primarily by trained community health workers or ‘CHiPs’ (Community HIV-care Providers). The CHiPs will provide HIV counseling and testing and active linkage to comprehensive care and prevention services. Each CHiP team will consist of a pair of individuals trained in HIV counseling and testing, and other aspects of HIV prevention and care. Each CHiP team will be responsible for implementing the intervention in an assigned subset of households, or “zone”. Although CHiP teams are affiliated with this research project (and this will be made clear to all those who interact with them), their role is primarily to deliver what is recognized by the WHO as a ‘best practice’ public health intervention. Hence for this project we regard ‘CHiP teams’ as separate from the ‘research teams’ and believe that the norms and standards governing their activities should largely be those accepted for the implementation of public health interventions rather than those applied to conventional clinical research projects. Regarding all CHiP team activities as research activities would make this complex public health research project logistically impossible to implement.

A cadre of people currently exists in all the study communities who would be appropriate for recruitment as CHiPs. These include ART adherence supporters, TB Treatment Supporters, PMTCT and male circumcision peer educators, home based care volunteers and HIV/AIDS support group members. Most of these people have the necessary skills and have received training in basic HIV counseling, psychosocial counseling, adherence counseling and door-to-door HIV testing. However, successful candidates will be retrained to update their knowledge and harmonize the implementation of the study interventions. Community Advisory Boards (CABs) and other local stakeholders will be consulted in developing job descriptions for CHiPs. All CHiPs will be able to read and write English, and will be conversant with the local geography.

3.3 Universal HIV Testing and Linkage to Care

Door-to-door voluntary HIV testing will be offered to all community members 16 years of age and older in Zambia and 12 years of age or older in South Africa, and any minors younger than these ages who request a test, with the consent of their guardians. The CHiP team will map, visit, and enumerate all households in their zone. Testing will occur during the first 9-12 months in year 1, and will be repeated at annual intervals for those who are HIV-uninfected or who are not tested for any reason during the first round. Household visits will be made at times convenient for community members, with repeat visits arranged for adult household members not present during the first visit. HIV testing will be done on finger-stick samples using rapid kits following appropriate local testing guidelines. The team may also pilot or adopt other testing approaches- such as oral swabs for self-testing- to achieve the study goal of universal testing annually in intervention communities. Implementing such strategies would be subject to additional funding and agreement amongst the study team. Individuals with discordant or inconclusive test results will be further evaluated according to local guidelines.

Following the household visit, the CHiP team will be responsible for ensuring linkage to HIV care at the health center for individuals identified as HIV-infected (defined as attending the health center and being given an "HIV care" patient number), offering male circumcision to men who are HIV-uninfected, facilitating linkage to the male circumcision service, and providing a regular supply of condoms to all households. They will subsequently make periodic revisits to appropriate households prior to the start of the next annual round to check on uptake of services (including male circumcision and ART), encourage HIV testing for those who have not been tested recently, and to provide adherence support to those receiving ART (see below). Essential data on each household member will be captured electronically and to the degree possible will be used to confirm follow up on CHiPs referrals at the healthcare facility as documented in facilities' patient record systems (see below).

In addition to the door-to-door service provided by the CHiP team, provision of HIV testing at other venues will be strengthened. This will include opt-out, provider-initiated testing and counseling for all patients presenting to the health center for any reason, testing of all women attending antenatal clinics (ANC), voluntary counseling and testing services provided at the health center or other community venues, and (if appropriate for the community) services provided in occupational settings. Information on how confidentiality of data captured into electronic databases will be maintained is provided below in Section 8.8.1.

3.4 Male Circumcision

Services for safe medical male circumcision will be available in all study communities. In most cases, the service will be provided within the health center, but if this exceeds the capacity of the health center, a special service will be set up during the initial phase of the intervention at a convenient community location.

3.5 Universal Treatment

Immediate eligibility for ART, irrespective of CD4 cell count, will be offered to all individuals attending adult HIV treatment and care services in the health centers in Arm A communities. Commencing with implementation of the changes included in version 3.0 of the study protocol, immediate ART will also be offered in Arm B and C communities. This offer will include those diagnosed during the door-to-door testing campaign as well as those diagnosed through other testing venues as described above. It will also include HIV-infected patients diagnosed previously who have not yet initiated ART, either because they have not been followed up at the health center or because they are not yet eligible for ART under current local guidelines. The clinics will however collect locator information so that the study team is aware of how many patients are coming from outside of the catchment area.

Linkage from diagnosis to treatment is a critical component of the intervention in Arms A and B. The CHiP team for each zone will be responsible for ensuring that this linkage takes place. They will enter details of adults identified as HIV-infected in the electronic database and will provide referral to the health center for initial assessment. They will also offer to accompany patients to the health center.

On presentation at the health center, patients in all three arms will have baseline blood tests performed in accordance with current standards of care in Zambia and South Africa. CD4 testing will also be performed, and will be used to determine eligibility for ART in Arms B and C until such time as immediate ART is available in those arms (due to changing guidelines or due to implementation of protocol version 3.0). Results of CD4 testing will not be used to guide the initiation of ART in Arm A, or in Arms B or C after the shift to the offer of immediate ART; all patients without contraindications will be immediately eligible for ART regardless of CD4 cell count (although witten consent will be required if immediate ART is being provided through the study rather than through revised local guidelines). After exclusion of active TB, patients will be offered TB preventive therapy according to local guidelines. Patients will also be offered antibiotic therapy for prophylaxis against opportunistic infections. The project team will endeavor to ensure that drug supplies are maintained without interruption.

Ensuring a high level of adherence to ART is key to the success of the intervention in Arms A and B. The CHiP team will be responsible for making regular home visits to patients to provide psycho-social support and to check and support treatment adherence. Because the CHiP team will make multiple visits to many households in their zone for multiple reasons besides linkage to HIV care (see above), this will reduce stigmatization or identification of HIV-infected individuals. Activities of the CHiP team will be supported by automatic updates produced by the electronic database showing which clients are due for a home visit.

Any patient initiating ART through the HPTN 071 (PopART) site clinics will continue to receive ART as any other clinic client would be. They will not be taken off ART if the study ends early or at the natural end of the study.

3.5.1 Choice of ART Regimen

To simplify the implementation of the UTT approach, the study team will attempt to initiate all consenting individuals (barring contraindications) using the same simple, standard regimen, one that is in line with treatment guidelines in both countries. Regimen details are included in the Study Specific Procedures Manual (SSP). The small number of individuals for whom this regimen is contraindicated according to local guidelines will be treated using alternative regimens as recommended by local guidelines for ART patients. ART adherence and toxicity monitoring will be managed according to national recommendations. Switch to a second-line treatment regimen and choice of second-line regimens will also be according to standard guidelines. Reported levels of transmitted drug-resistant virus remain low in these settings (< 6%). Most cases of transmitted drug resistance are to nucleoside reverse transcriptase inhibitors or non-nucleoside reverse transcriptase inhibitors, with minimal transmitted drug resistance to protease inhibitors [100].

3.6 Treatment According to Local Guidelines (Arms B & C)

Arrangements for linkage to care, treatment and monitoring in Arm B will be similar to those in Arm A, except that until immediate offer of ART is in place (due to change in local guidelines or due implementation of protocol version 3.0), ART will be initiated only

if the patient is eligible according to current local guidelines (e.g., based on CD4 cell count or HIV clinical stage). Treatment regimens will follow current local standards of care in all arms. Additional arrangements for linkage to care will not be offered in Arm C.

With implementation of version 3.0 of the protocol, ART will be offered to all HIV-infected clients at health centers in all three Arms (A, B and C) as described above. Research consent for ART will need to be obtained for all individuals initiating ART outside of local guidelines. Research consent will no longer be needed to initiate ART in any study arm once local treatment guidelines have changed to offer treatment to all infected clients.

The change in protocol version 3.0 to offering universal treatment in Arms B and C will provide a unique and important opportunity to document the process of transition. Lessons learned through this process may thereby inform health policy at a time when many countries are expected to undertake a transition to universal treatment.

3.7 Prevention of Mother-to-Child Transmission

In all three study arms, the project team will endeavor to ensure that local policy for PMTCT is delivered effectively at health centers providing antenatal or delivery care, which will usually be the same centers at which ART is delivered.

In Arms A and B, the CHiP team will encourage women who may be pregnant to receive pregnancy testing at the health center. As well CHiPs will encourage pregnant women who are encountered during regular household visits in their zone to attend an ANC. If CHiPs encounter women who are HIV infected and pregnant or breastfeeding, they will refer them for PMTCT. In both Zambia and the Western Cape of South Africa the "B+" option for PMTCT has been adopted as government policy, promoting lifelong ART for pregnant women with HIV infection. Because of this, the requirement to obtain written informed consent before initiating ART among clients who have CD4 cell count above the ART threshold per local guidelines or are at an early WHO stage, will not apply for HIV infected pregnant or breastfeeding women once "B+" is implemented in the local health care system; such women will be automatically eligible per government policy. The CHiP team will be responsible for assisting these women with linkage to care, if this has not taken place.

3.8 Management of Sexually Transmitted Infections

Services for STI treatment will be in place in all health centers according to national policies. In all three study arms, the project team will endeavor to ensure that these services are operating effectively, and that drug supplies for STI treatment are maintained without interruption.

3.9 Screening and Referral for TB

While performing household visits, CHiPs will assess whether clients have symptoms or exposure to TB using a small number of questions included in the CHiPs' standard information gathering tool (CHiPs register). Clients who are suspected of possibly having

TB will be asked to provide sputa for laboratory testing. CHiPs will follow up to ensure that clients receive their test results and to ensure that those positive for TB are seen at the healthcare facility for treatment.

3.10 Provision of PrEP

There is growing evidence from randomized clinical trials that use of oral daily PrEP confers significant protection against HIV acquisition, but it is currently unclear how the results of those trials will be incorporated into WHO and national guidelines. New developments will be reviewed during the course of the trial. If the provision of PrEP is incorporated in local guidelines during the course of the trial, the combination prevention package will be adapted appropriately.

3.11 Standard of Care

The primary objective of this trial is to evaluate the impact of an intensive combination prevention intervention program on HIV incidence when compared with current standard of care in Zambia and South Africa. The study team will work with in-country health authorities to ensure to the degree possible that existing services in the seven control communities meet current local guidelines for HIV prevention and care. These activities include endeavoring to ensure that:

- Community members have adequate access to services for voluntary HIV counseling and testing.
- Referral services for male circumcision are available to men who are HIV-uninfected and wish to be circumcised.
- HIV treatment and care are provided according to current local guidelines prior to version 3.0 of the protocol, and, upon implementation of version 3.0 of the protocol, will be provided to all HIV-infected clients attending an Arm C health center. The study team will endeavor to ensure that antiretroviral drugs are available to all patients who qualify for treatment, using the current ART drug regimen employed in the government program in each country.
- Adequate services for PMTCT are in place at antenatal and delivery services in the control communities.
- Treatment services for STIs and condoms are available through health units in accordance with local HIV prevention guidelines.

To help interpret the results of the trial, process data from the control communities on HIV testing uptake, ART delivery, male circumcision, and provision of PMTCT services will be collected for comparison with the intervention communities. These data will be used to inform model fitting and projections.

3.12 Delivery of Intervention

3.12.1 Activities with Local Health Centers/Community Institutions

The study team will collaborate with local health centers/community institutions to facilitate the following:

- Establishment of systems that will provide CHiPs teams with information about their clients' follow up on referrals from patient record systems maintained at the healthcare facilities
- Promotion of the study at the community level (Arms A & B only)
- Strengthening the provision of HIV services at local health centers and elsewhere in all arms, including
    - ANC care
    - Voluntary Counseling and Testing (VCT) at health centers and other venues (e.g. occupational venues, community campaigns, etc.)
    - PMTCT services
    - STI treatment and referral services
    - Male circumcision services and referral
    - Activities with national/global health entities
    - Opt-out provider-initiated counseling and testing (strengthened in Arms A & B; in Arm C the study will support if already part of local services)

The home-based HIV testing that is carried out by the CHiP teams will be captured on the CHiPs electronic data collection device. (These data are stored on the device in encrypted form and are accessible only to authorized users after entry of an individual username and password.) To the degree possible, the study team will obtain data from electronic patient record systems at the healthcare facilities on those clients documented as having consented to the CHiPS intervention who are captured in the CHiPs electronic database. This linkage will provide feedback to CHiPs on whether clients need further support to obtain care and will help the team to estimate the proportion of HIV-infected individuals who register for HIV care following an HIV diagnosis, and the time from HIV diagnosis to HIV care registration. In Arms A and B, but not Arm C, there will be active follow-up of HIV-infected individuals who have been referred for HIV care by CHiP teams, but who have not registered for HIV care. CHiP teams will also provide additional support for retention in HIV care and ART adherence, contributing to active follow-up of individuals who have missed scheduled visits. If an individual has left the community, they will not be followed up outside the community.

3.12.2 Collaborations

The study team will collaborate with national and global health entities to facilitate the following in all communities:

- Adequate supplies of antiretroviral drugs for all who are prescribed them
- Adequate STI care, including test kits and drugs for STI and treatment
- Adequate supplies of condoms
- Adequate clinic staffing
- Approval of use of health center data
- Adequate clinical supplies for HIV-infected individuals, including
    - TB tests and treatment
    - Blood tests for clinical care

- Antibiotics for TB and opportunistic infection prophylaxis

Table 1- Summary of Intervention Components

<table>
<tr><th>Study Arms</th><th>Activity</th></tr>
<tr><td rowspan="5">Arms A & B</td><td>Study Start</td></tr>
<tr><td>Enumeration of all houses in each community
Division of houses into “zones” and assignment of a CHiP team to each zone
CHiP Team will:
Offer HIV testing with counseling to all household members (all individuals 16+ years old in Zambia and 12+ years old in South Africa and minors with the consent of their guardians) and will record HIV status with name in mobile device
Provide linkage-to-care at local health center for HIV-infected persons
Refer/link men who are uncircumcised to circumcision, if interested, focusing on men who are HIV-uninfected
Identify pregnant women and encourage them to get follow-up at an ANC; encourage HIV-infected pregnant women to initiate ART and PMTCT per local guidelines as part of their care
Provide on-going psycho-social support for ART adherence to those on ART
Encourage STI treatment and provide prevention resources including condoms
Screen clients for TB and assist in linkage to care for those with positive laboratory screening results.</td></tr>
<tr><td>On-going Throughout the Study</td></tr>
<tr><td>CHiP team will:
Promote community-based HIV prevention services in their zone
Follow up with all persons in their zone who are identified as HIV-infected (by CHiP team or at other venues) to encourage and facilitate them to access HIV care
Return to houses where residents were not available for testing during original or subsequent visits, to complete testing of all willing residents
Encourage pregnant women to get follow-up at an ANC; encourage HIV-infected pregnant women to initiate ART or PMTCT per local guidelines as part of their care
Provide on-going psycho-social support for ART adherence to those on ART
Encourage STI treatment and provide prevention resources including condoms
Screen clients for TB and assist in linkage to care for those with positive laboratory screening results.</td></tr>
<tr><td>Follow Up Testing at 12-, 24-, and 36-Months</td></tr>
</table>

	• CHiP teams will cycle back through their zone at 12, 24, and 36 months to repeat universal testing in each household for those not previously diagnosed as HIV-infected
	Procedures and Tests at the Health Centers
	• Community members who are identified as HIV-infected will receive clinical support and laboratory tests at local health centers, consistent with local guidelines for HIV treatment and care, with immediate eligibility for ART initiation (Arm A) or eligibility for ART according to local guidelines (Arm B). Upon full implementation of protocol version 3.0, Arm B clients will also be offered immediate eligibility for ART as in Arm A.
	By Study Start and Throughout the Study Period
	• Endeavour to ensure that the following resources are available:<br>o Voluntary HIV counseling and testing<br>o Male circumcision<br>o PMTCT<br>o HIV treatment and care<br>o STI treatment and prevention resources including condom distribution<br>o Resources for TB testing and treatment<br>o Clinical support and laboratory tests at local health centers for provision of prophylaxis against TB and other opportunistic infections for all HIV infected individuals, consistent with local guidelines for HIV treatment and care
Arm C	By Study Start and Throughout the Study Period
	• Endeavour to ensure that the following standard-of-care resources are available:<br>o Voluntary HIV counseling and testing<br>o Male circumcision<br>o PMTCT<br>o HIV treatment and care according to local guidelines prior to protocol version 3.0. Upon full implementation of protocol version 3.0, Arm C clients will be offered immediate eligibility for ART.<br>o STI treatment and prevention resources including condom distribution<br>o Resources for TB testing and treatment<br>o Clinical support and laboratory tests at local health centers for provision of prophylaxis against TB and other opportunistic infections for all HIV infected individuals, consistent with local guidelines for HIV treatment and care

3.13 Monitoring and Evaluation Plan

The delivery of the intervention will be monitored at frequent intervals from the time of initiation to evaluate the uptake of the intervention. Remedial action will be taken at cluster-level if delivery is behind schedule. Details of these procedures will be set out in the SSP Manual.

Briefly, during each round of CHiPs testing, HIV testing uptake is targeted at 90%. In each community, individual CHiP teams will report weekly to the CHiPs supervisors using data from electronic records. Where a team is not meeting the target level of testing uptake, this will be explored in real time and where necessary appropriate intervention, retraining or modification of strategies will take place. Following HIV diagnosis, the target will be for linkage to care and (in Arm A communities) initiation of ART in 80% of cases within 3 months. These targets of 90% uptake and 80% initiation should lead to an overall uptake of 72%, just above our central target of 70% uptake (see Table 4). This will be supported by notification of CHiP teams, based on clinic and CHiPs databases. When patients have not presented within a defined interval, this will trigger repeat home visits for follow-up and support to access care. Data on linkage to care will be reviewed monthly to identify CHiP teams that are not meeting targets and to effect remedial actions as noted above.

As stated in Section 2.4, interim evaluation will take place during the first two years of intervention to determine whether to continue with the 36 month follow-up of the *Population Cohort* and the fourth year of intervention. The evaluation will consider milestones such as uptake of the intervention and indicators of futility.

The study team will also have a continuous presence in each community and will monitor other programs in the community that may affect uptake of the intervention.

4.0 STUDY POPULATION

4.1 Description/Selection of the 21 Study Communities

This study will be carried out in areas of Zambia and South Africa that are known to have high HIV prevalence and incidence and are continuing to experience severe generalized HIV epidemics, with prevalence levels of 15-20% in many areas. National estimates of HIV prevalence in adults aged 15-49 are 13.5% for Zambia and 17.8% for South Africa [35, 36], and incidence estimates are 1.17% and 1.49% respectively.[101]

The specific communities selected for randomization in this trial are largely the communities that were selected for the ZAMSTAR trial. Selection criteria for communities included having a health facility that offered TB and HIV services, a high HIV prevalence, a TB notification rate of at least 400/100,000 per year and a total population of about 20,000 or more. The communities were selected in conjunction with national and local health authorities. All communities were willing to be included in a randomized trial. Extensive work has been done with community representatives to ensure that they understand the fundamentals of research and they were all very supportive during the ZAMSTAR trial.

Additional considerations that informed selection of these sites for the current study included:

- Geographically distinct
- No other major HIV prevention studies planned or ongoing
- Adequate population size to minimize the effects of contamination on outcome measurements (due to contact with other communities or residents of other communities)
- Community willingness to be involved in this current study

The final endpoint measurement of the ZAMSTAR trial involved a community-level survey of 4000 randomly selected individuals from each community and allowed measurement of the uptake of HIV testing, uptake of ART, circumcision and HIV prevalence, which are presented in Table 2. These data would not otherwise be available at this level, as most surveys only provide data at provincial or district level.

Due to differences between the designs of ZAMSTAR and the current study (requiring seven matched triplets) four ZAMSTAR communities from Zambia were excluded from the current study (the most rural communities with the lowest HIV prevalence) and an additional community was added in South Africa. Maps of the locations of the study communities are provided in Figure 3.

Table 2- Twenty one proposed study clusters in Zambia and South Africa and relevant background data

Community	Population	Adult HIV prevalence	Know HIV status	HIV-infected on ART	Men circumcised
**ZAMBIA**					
**Dambwa**	31629	26%	65%	24%	14%
**Maramba**	55011	19%	66%	30%	21%
**Chawama**	129221	15%	35%	16%	8%
**Kanyama**	124284	17%	65%	28%	19%
**Shampande**	41615	16%	58%	38%	14%
**Chipata**	166251	15%	59%	24%	8%
**Ngungu**	38081	17%	30%	18%	7%
**Makululu**	34623	20%	52%	30%	8%
**Ndeke**	33297	13%	56%	31%	17%
**Chimwemwe**	42898	15%	51%	25%	16%
**Chifubu**	60222	19%	56%	32%	17%
**Chipulukusu**	45234	18%	52%	19%	12%
**S AFRICA**					
**Delft South**	31423	14%	48%	23%	53%
**Ikhwezi***	N/A	18%	N/A	N/A	N/A
**Bloekombos***	N/A	22%	N/A	N/A	N/A
**Dalevale***	N/A	13%	N/A	N/A	N/A
**Wellington***	N/A	13%	N/A	N/A	N/A
**Cloetesville***	N/A	16%	N/A	N/A	N/A
**Luvuyo***	N/A	19%	N/A	N/A	N/A
**Kuyasa**	39168	19%	53%	22%	87%
**Town II***	N/A	19%	N/A	N/A	N/A

*Seven South African sites – data not available from ZAMSTAR trial; accurate population size estimates will be available upon ethics approval,% HIV-infected on ART not yet available. Estimates of HIV prevalence based on sub-district antenatal clinic HIV prevalence, or (for Luvoyo and Town II) based on ZAMSTAR data for communities in the same sub-district. Estimates of % who know their HIV status, and % men circumcised, not available for 7 communities that were not included in ZAMSTAR trial.

Figure 3- Location of 21 clusters in Zambia and South Africa

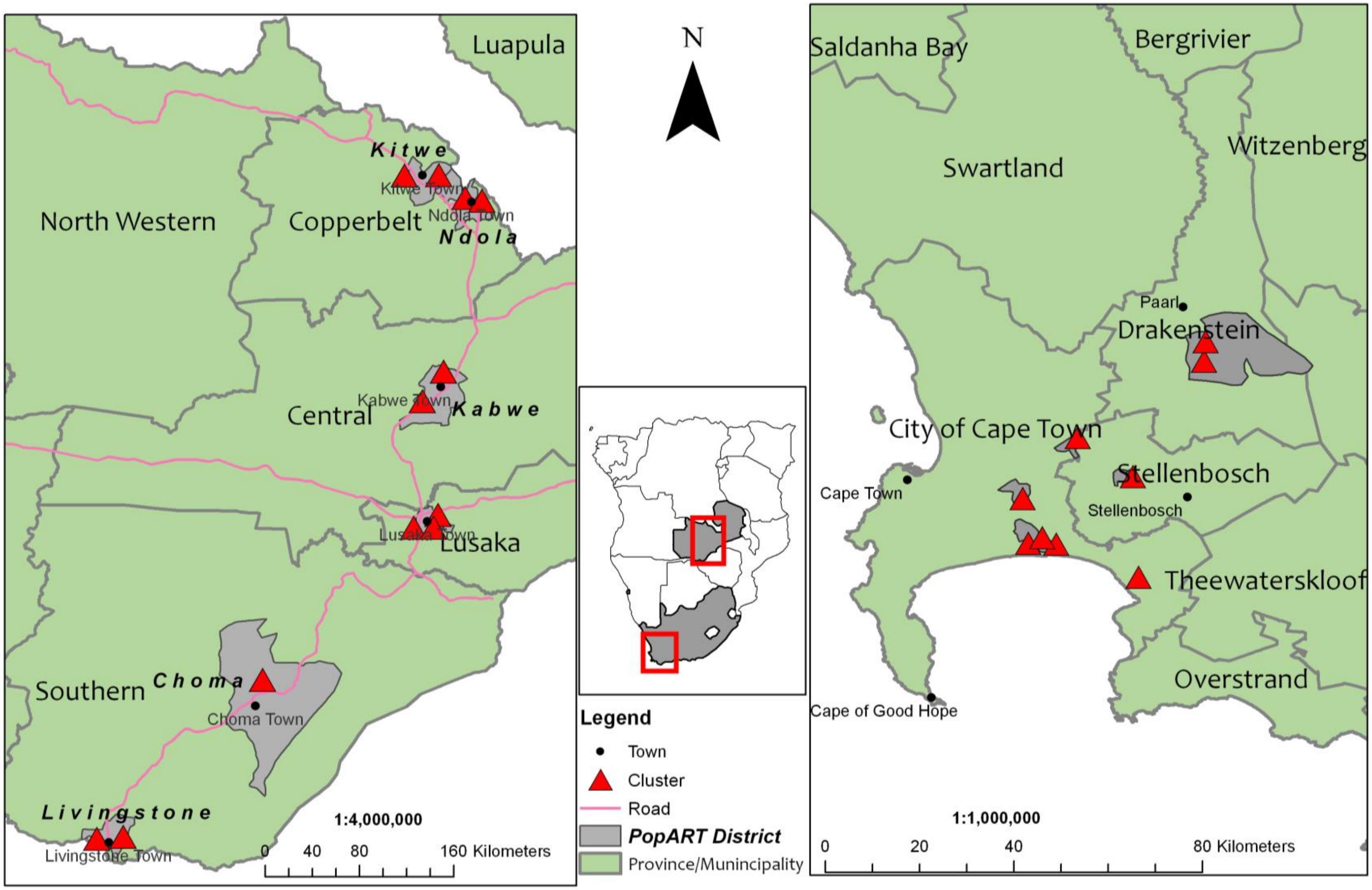

4.2 Randomization

The first step in randomization will be to obtain agreement from communities to take part in the study and to accept the results of the random assignment to a study arm, whatever the outcome. Randomization will then take place in a public ceremony at which the allocation of communities to study arms will be decided using a transparent and fair process. After initiation of the intervention, if any community needs to be removed from the study (for example, if a community should cease to agree to participate in the study) then the study leadership will decide upon the most appropriate course of action, which would likely include replacement of the community.

4.3 Community Engagement

This study will build on the community engagement and community capacity established during the ZAMSTAR trial. To work within a community successfully requires a trusting relationship which requires time to be built, and through the ZAMSTAR trial the research team spent seven years engaging with these communities. Community advisory bodies in all communities were worked with (and developed, where needed), and were trained in research ethics and conduct. These bodies were invaluable during the ZAMSTAR trial to represent community views and to assist the research teams during their work in the communities. The HPTN 071 study will build on these experiences, widening the constituency of these bodies where necessary.

Direct community engagement for this study began early, during the formulation of the research questions, when various community groups (including CABs in former ZAMSTAR study communities), civil society organizations (such as the Cape Metro Health Forum, Treatment Action Campaign and South African National AIDS Council (SANAC)) and government authorities were consulted for their input before the final proposal was submitted, and again after the grant was awarded. Some members of these organizations have provided comments on the protocol and will provide additional input during the preparatory phase of the trial.

A key aspect of the preliminary work during the first year of the study will be a stakeholder analysis, results of which will be used to identify relevant stakeholders to be considered in community engagement as well as membership for CABs. CABs in this study will have broad representation from various community groups and stakeholders such as churches, schools, law enforcement, government structures at community level, health-related committees, and development-related committees. Selection criteria will be arrived at through consultation with the stakeholders. Each study community will have a member of the study team responsible for community engagement activities. One of the main tasks of the staff will be to keep dialogue open and ongoing between researchers and community groups.

Community engagement will be an ongoing process through regular contacts with community groups and CABs. A combination of mechanisms will be utilized, such as community meetings, workshops with key stakeholders, participant meetings, CHIPs

meetings and some existing avenues such as health committees, development committees, civil society groups and local HIV/AIDS coordinating forums such as the District AIDS Task Forces in Zambia and Treatment Action Campaign and SANAC in South Africa. This will enable the study management team to ensure that information about the study is disseminated widely in the communities involved and to keep the community stakeholders updated regarding progress of the study, events that may arise in conduct of the research, and new developments in HIV prevention and treatment. Community engagement will also allow researchers to receive feedback from the community on social harms, individual and community level risks, perceptions about the study in the community, and implementation challenges. All study staff and stakeholders will receive training in Good Clinical Practice (GCP)/research ethics before commencement of intervention implementation.

Community engagement will also be factored into other study processes such as the communication plan, especially the dissemination of study results (preparation of the community). Overall, strong community engagement will allow the establishment of a partnership between communities, participants and researchers to ensure the latter discharge their responsibilities ethically in the study communities**.** A component of the qualitative research will focus on the application of ethical principles in practice as well as documenting and evaluating community engagement.

In both countries, study committees will be formed on which community representatives will serve along with department of health and other stakeholder representatives. These committees will meet periodically for the duration of the trial and these meetings will provide a forum for trial staff to engage with community representatives around the progress of the trial and any relevant issues that may arise.

5.0 RESEARCH PROCEDURES AND ACTIVITIES

The deployment of the interventions among the communities assigned to Arms A and B is expected to lower HIV incidence throughout the communities. Measurement of HIV incidence, however, will occur in a subset of adults enrolled into the *Population Cohort* in each study community and followed longitudinally. Secondary outcomes (among them process measures and qualitative research aims) will also be measured from data provided by this cohort, from routinely-collected health center data, and from data collected by CHiPs during household visits. Other secondary outcomes will be measured from qualitative and case-control studies, and, if funded, from additional one-time surveys. Research activities, including identification and consent of participants, conduct of study procedures, and retention-related activities, will be performed by a trained research team, separate from the CHiP teams that will be responsible for delivering the intervention to the community-at-large. A table summarizing the secondary objectives and outcomes, including the source of outcome data, is provided in Section 7.11.

Descriptions of the *Population Cohort* and surveys are provided below. Detailed instructions to guide and standardize all study procedures across sites will be provided in the SSP Manual.

5.1 Population Cohort

5.1.1 Sampling/Recruitment of *Population Cohort*

Prior to study commencement in each community, satellite maps will be used to enumerate and list all the houses in the community. A simple random sample of houses will be selected and visited by field staff who will list all household residents. One adult per household, aged 18-44 years, will be randomly selected from this list for inclusion in the *Population Cohort*. This age range was chosen because individuals 18 years and older will be able to participate in the cohort without a guardian's consent, and adults under 45 years are believed to be most likely to experience a measurable change in HIV incidence as a result of the intervention. A randomization scheme will be used to select one age-eligible resident from each ramdomly-chosen household. The selected individuals will be invited to join the *Population Cohort*, if they meet the other eligibility criteria. A blood sample will be collected and stored for retrospective testing which will include HIV testing and other secondary outcome measures (see Appendix IA). HIV counseling and testing using rapid HIV test kits will be offered to those who wish to know their test status (participants may refuse an HIV rapid test and still participate in the *Population Cohort*). All HIV-infected individuals (those testing positive on the rapid test as well as those who are already aware of their positive status) will be referred to a health center for further management. All cohort members, irrespective of HIV status, will be followed after 1 and 2 years (interim surveys) and 3 years (final survey) to measure HIV incidence and other outcomes, as described below.

Only one adult will be randomly selected from each randomly selected household to participate in the *Population Cohort* for outcome evaluation. This is to avoid the distortion of the trial results which might occur if whole households or several members of a household were to be evaluated, since this would in itself constitute a mass testing and counseling intervention. To avoid the possibility of coercion or biased study data, field staff will not enumerate a randomly selected household if someone in that household is an employee of ZAMBART or Desmond Tutu TB Centre. If the person selected for the cohort from a given household is ineligible or refuses participation, the team will move on to the next household on the list. As described in Section 7, the statistical analysis will take into account the different sampling probabilities resulting from the selection of one individual irrespective of household size.

5.1.2 Inclusion Criteria *Population Cohort*

- 18 – 44 years of age
- Able and willing to provide informed consent
- Residing within catchment area of a designated local health unit and intending to remain so for the next three years
- Residing in a randomly selected household

5.1.3 Exclusion Criteria *Population Cohort*

- Current enrollment in another HIV treatment, prevention, or PrEP study
- Current, or prior enrollment in an HIV vaccine study

- Anything that, in the opinion of the investigator, would preclude informed consent, make study participation unsafe, complicate interpretation of study outcome data, or otherwise interfere with achieving the study objectives.

5.1.4 Procedures and Activities

***Population Cohort* Creation**

- Generation of random sample of houses in the community for visits
- Research staff visit selected houses and enumerate all adult residents (18-44)
- Selection of one adult at random from the household for invitation to *Population Cohort*
- Complete eligibility assessment
- Complete informed consent

Visit Procedures (Enrollment, 12 months, 24 months, and 36 months)

Administrative, Behavioral, and Regulatory Procedures

- Obtain informed consent for enrollment (study start only)
- Solicit consent to store specimens for future testing and to use participant-identified data from heath center for cohort analyses (study start only)
- Obtain/update locator information
- Complete survey to include topics of stigma and discrimination, and socio-demographic, health, social, behavioral, and economic factors

Clinical/Counseling Procedures

- Perform HIV rapid tests, if participant agrees
- Provide pre- and post-test counseling and test results, for those willing to have HIV rapid testing
- Collect blood for laboratory testing and sample storage

Laboratory Procedures (see Appendix IA)

- HIV testing
- HSV-2 testing*
- Plasma storage**

**HSV-2 testing will be performed at enrollment (PC0) and at 36 months (PC36); HSV-2 testing will not be performed for participants enrolled at PC12.
**Stored samples will be used for retrospective, centralized testing, as described in Section 9. Additional details are provided in Appendix IA, the protocol for the *Phylogenetics Ancillary Study*, and the HPTN 071 SSP Manual.

In the event that accrual falls far below target (greater than a ~20% shortfall in a particular community), additional participants may be enrolled in selected communities during the 12 month follow-up survey. They will then be followed up during the 24 month and 36 month surveys. Participants who are found to be HIV uninfected during this additioinal enrollment period will contribute to the primary outcome evaluation.

Results from laboratory tests are not returned to Population Cohort participants under normal circumstances. However, if the results of in-country laboratory HIV tests at a particular visit differ from HIV rapid test results given to the participant at the same visit, study staff will attempt to contact the participant and encourage the participant to receive additional HIV testing to clarify his/her HIV infection status.

5.1.5 Reviewing Health Center Records for *Population Cohort*

For HIV-infected *Population Cohort* members who provide consent to access their health clinic records, the study team will attempt to link the research data to routine electronic HIV care data that are collected at the health center, to measure HIV disease progression and death, ART toxicity, and the time between HIV diagnosis and initiation of HIV.

5.1.6 Retention in *Population Cohort*

Once a participant is enrolled into the *Population Cohort*, the research team will make every effort to retain him/her for the follow-up surveys at 1, 2, and 3 year time points in order to minimize possible bias associated with loss-to-follow-up. The retention goals for the *Population Cohort* are 90% retained at 12 months, 80% at 24, and 75% at 36 months. Research staff are responsible for developing and implementing local standard operating procedures to reach this goal. Components of such procedures include:

- Thorough explanation of the study visit schedule and procedural requirements during the informed consent process, with re-emphasis at the subsequent 12-monthly study visits.
- Thorough explanation of the importance of their participation to the overall success of the study.
- Collection of detailed locator information at the study Enrollment Visit, and active review and updating of this information at each follow-up visit.
- Regular communication with the study community at large to increase awareness about HIV/AIDS and explain the purpose of HIV prevention research and the importance of completing research study visits.

In addition to the components described above, which are standard for all HPTN studies, the team will work with local community stakeholders, experienced in-country staff, and participants themselves to identify locally-effective, study-specific strategies for improving participant retention. Such approaches may include use of short message service (SMS) messages to remind participants about upcoming visits, enlisting the assistance of household members to support adherence to study visits and ART adherence, or other methods.

Any member of the *Population Cohort* who leaves the community will be censored regardless of where they move to. Individuals who are reported to have moved *within* the community, but cannot be contacted for one follow-up visit, will not be censored. This is because they can still contribute to the study if they are contacted at a later follow-up visit: for example, if they miss the 12-month follow-up visit, they can still contribute to the study if they are contacted at one or both of the 24 and 36 month follow-up visits

Eligibility criteria for *Population Cohort* enrollment include current residence and intending to remain in the community during follow-up in an attempt to limit loss from the *Population Cohort* due to mobility. Retention rates are broadly in line with experience from previous trials.

Participants may voluntarily withdraw from the study for any reason at any time. The Investigator also may withdraw participants from the study in order to protect their safety and/or if they are unwilling or unable to comply with required study procedures after consultation with the Protocol Chair, Division of AIDS (DAIDS) Medical Officer, Statistical and Data Management Center (SDMC) Protocol Statistician, and Coordinating and Operations Center (CORE) Protocol Specialist.

Participants also may be withdrawn if the study sponsor, government or regulatory authorities, or site Institutional Review Board (IRB)/Ethics Committee (EC) terminates the study prior to its planned end date.

5.2 Population Cross-Sectional Survey (if funded)

As noted, the *Population Cross-Sectional Survey* described below is currently not funded and is therefore not a part of the current study design. However, the procedures that would be undertaken to implement this activity are described briefly below to illustrate what this work, if funded, would encompass.

5.2.1 Sampling/Recruitment of *Population Cross-Sectional Survey* Participants

A simple random sample of houses will be generated, similar to the method used for the Population Cohort. Research staff will visit the houses in this list in order and all eligible adults in a household will be solicited to participate in the survey. Recruitment will cease when five hundred participants per cluster have been enrolled into the survey.

5.2.2 Inclusion Criteria

- 18 – 44 years of age
- Able and willing to provide informed consent
- Residing within catchment area of a designated local health unit for the three years prior to conduct of the survey
- Residing in a randomly selected household

5.2.3 Exclusion Criteria

- Current enrollment, or enrollment within the prior three years, in another HIV treatment, prevention, or PrEP study
- Current or prior enrollment in an HIV vaccine study
- Anything that, in the opinion of the investigator, would preclude informed consent, make study participation unsafe, complicate interpretation of study outcome data, or otherwise interfere with achieving the study objectives.

5.2.4 Procedures and Activities

***Population Cross-Sectional Survey* Creation**

- Identify a random sample of houses in the community for visits
- Research staff visit selected houses and invite all adult residents (18-44) to participate
- Complete informed consent

Visit Procedures (36 months only)

Administrative, Behavioral, and Regulatory Procedures

- Obtain informed consent for enrollment
- Solicit consent for storage of specimens for future testing
- Obtain/update locator information
- Complete survey to include socio-demographic, health, social, behavioral, and economic factors
- Perform qualitative interviews covering stigma and discrimination (randomly-selected HIV-infected participants only)

Clinical/Counseling Procedures

- Perform HIV rapid tests, if participant agrees
- Provide pre- and post-test counseling and HIV rapid test results, for those willing to receive results
- Collect blood for laboratory testing and sample storage

Laboratory Procedures

- HIV testing
- Plasma storage*

*Stored samples will be used for retrospective, centralized testing, as described in Section 9. Additional details are provided in Appendix 1A, and the HPTN 071 SSP Manual.

5.3 Case-Control Studies

Three *case-control studies* will be undertaken to improve our understanding of participation in three key steps of the intervention, each of which is essential to the success of the trial interventions. These studies will provide information about which factors are associated with non-engagement with particular components of the intervention and will be important for interpreting the findings of the trial, informing mathematical models, and guiding policy.

5.3.1 *Case-Control Study 1* - Uptake of Testing in the First Round of Home-Based Testing Provided by CHiP Teams in Arms A & B

A *case-control study* of refusers (cases) and acceptors (controls) of home-based HIV testing by CHiPs will be undertaken to identify the characteristics of refusers/acceptors and reasons for refusal/acceptance. As this is the first step in the cascade of interventions

in Arms A and B, this will be key in interpreting uptake of subsequent steps of the interventions, and will be important for identifying ways to increase testing uptake. The CHiP teams will request permission from individuals declining the intervention to be approached by the research team for potential enrollment into the case-control studies.

5.3.1.1 Sampling/Recruitment of *Case-Control Study 1* Participants

Four hundred cases (refusers of CHiP testing) and 400 randomly selected controls (acceptors) from the communities in Arms A and B will be enrolled. Potential participants will be selected at random and approached by CHiP personnel, who will seek verbal consent for follow-up by a research team. The latter will then obtain the formal informed consent for *case-control study* participation. Recruitment will cease when 400 participants have been enrolled in each of the two groups.

5.3.1.2 Inclusion Criteria *Case-Control Study 1*

- At least 18 years of age
- Able and willing to provide informed consent
- Resident in the cluster during the first round of testing
- Visited by a CHiP team and offered testing during the first round of home-based testing

5.3.1.3 Exclusion Criteria *Case-Control Study 1*

- Individuals belonging to the *Population Cohort* or other case-control studies
- Individuals known to be HIV-infected after testing elsewhere
- Individuals working on, or living in the same household as a member of staff working on, the HPTN 071 (PopART) trial

5.3.1.4 Procedures and Activities

<u>Administrative, Behavioral, and Regulatory Procedures</u>

- Obtain informed consent for enrollment
- Complete questionnaire of socio-demographic, clinical, and behavioral characteristics

Standardized questionnaires will encompass sexual and health seeking behavior, previous HIV testing, as well as stigma and psycho-social questions. Cases and controls will also have separate sections in the questionnaire, to explore reasons for not testing and motivation to test, respectively. The standardized surveys will be carried out by *case-control study* teams after the end of the first CHiP home-based testing round within a community, at the household or an alternative community location chosen by the participant.

5.3.2 *Case-Control Study 2* - Uptake of Immediate Treatment in Arm A

A *case-control study* of cases (who do not start ART within 6 months of being identified as HIV-infected and referred for HIV care by CHiPs) and controls (who start ART within

6 months of referral) selected randomly from HIV-infected individuals from Arm A communities, will be undertaken to identify the characteristics of those who do/do not start ART within 6 months and reasons for starting/not starting. As timely treatment is the linchpin of the PopART intervention, understanding the barriers to wide-scale uptake (if any) will be crucial in understanding the trial findings.

5.3.2.1 Sampling/Recruitment of *Case-Control Study 2* Participants

Four hundred cases (non-receivers of ART within 6 months after first receiving an HIV positive test from a CHiP, or disclosing previously-diagnosed HIV infection to a CHiP and being referred to HIV care) and 400 randomly selected controls (HIV-infected initiators of ART within this timeframe) from the communities in Arm A will be enrolled. Potential participants will be selected at random and approached by CHiP personnel, who will seek verbal consent for follow-up by a research team. The latter will then obtain the formal informed consent for *Case-Control Study 2* participation. Recruitment will cease when 400 participants have been enrolled in each of the two groups.

5.3.2.2 Inclusion Criteria *Case-Control Study 2*

- At least 18 years of age
- Able and willing to provide informed consent
- Resident in the cluster during the first round of testing
- Tested HIV-infected in CHiP home-based testing, or HIV-infected and disclosed that they were previously diagnosed as HIV-infected to CHiP team

5.3.2.3 Exclusion Criteria *Case-Control Study 2*

- Individuals enrolled in the *Population Cohort* or other *case-control studies*
- HIV-infected individuals already on ART before study commences
- Individuals working on, or living in the same household as a member of staff working on, the HPTN 071 (PopART) trial

5.3.2.4 Procedures and Activities

<u>Administrative, Behavioral, and Regulatory Procedures</u>

- Obtain informed consent for enrollment
- Complete questionnaire of socio-demographic, clinical, process uptake and behavioral factors

Standardized questionnaires will encompass sexual and health seeking behavior, as well as stigma and psycho-social questions. Cases and controls will also have separate sections in the questionnaire, depending on whether: i) they did not attend the health center in the first place (cases), ii) attended but did not initiate treatment within 6 months (cases), iii) attended and initiated treatment within 6 months (controls) to explore their reasons for not starting ART within 6 months or motivation to start timely treatment. The standardized surveys will be carried out by *case-control study* teams after the end of the first CHiP home-based testing round within a community, at the household or an alternative community location chosen by the participant.

5.3.3 *Case-Control Study 3* - Uptake of Testing in the Second Round of Home-Based Testing Provided by CHiP Teams in Arms A & B

A *case-control study* of refusers (cases) and acceptors (controls) of home-based HIV testing by CHiPs in the second round of testing will be undertaken to identify the characteristics of refusers/accepters and reasons for refusal/acceptance at this stage. Because regular re-testing of individuals who were HIV-uninfected when last tested is a crucial step in the cascade of interventions in Arms A and B, the understanding of reasons for not accepting CHiP home-based testing in the second round is key for interpreting uptake of subsequent steps of the interventions, and for identifying ways to increase the uptake of re-testing. The CHiP teams will request permission from individuals declining the intervention to be approached by the research team for potential enrollment into the case-control studies.

5.3.3.1 Sampling/Recruitment of *Case-Control Study 3* Participants

Four hundred cases (refusers of CHiP testing) and 400 randomly selected controls (acceptors) from the communities in Arms A and B will be enrolled. Potential participants will be selected at random and approached by CHiP personnel, who will seek verbal consent for follow-up by a research team. The latter will then obtain the formal informed consent for case-control study participation. Recruitment will cease when 400 participants have been enrolled in each of the two groups.

5.3.3.2 Inclusion Criteria *Case-Control Study 3*

- At least 18 years of age
- Able and willing to provide informed consent
- Resident in the cluster during the second round of testing
- Visited by a CHiP team and offered testing during the second round of home-based testing

5.3.3.3 Exclusion Criteria *Case-Control Study 3*

- Known HIV infected from CHiP data.
- Individuals belonging to the *Population Cohort* or other case-control studies
- Individuals working on, or living in the same household as a member of staff working on, the HPTN 071 (PopART) trial

5.3.3.4 Procedures and Activities

<u>Administrative, Behavioral, and Regulatory Procedures</u>

- Obtain informed consent for enrollment
- Complete questionnaire of socio-demographic, clinical, process uptake and behavioral factors

Standardized questionnaires will encompass sexual and health seeking behavior, previous HIV testing, as well as stigma and psycho-social questions. The primary analysis will compare refusers and acceptors of testing at this stage. There will also be sub-group

analyses to consider participants who: (i) accepted, tested and were found negative at the first round, (ii) refused testing at the first round, (iii) were absent at the baseline testing round (away from home or newly moved into community). The standardized surveys will be carried out by *case-control study* teams at the end of the second CHiP home-based testing round (i.e. 12 month round) within a community, at the household or an alternative community location chosen by the participant.

5.4 Qualitative Studies

Qualitative studies will be conducted in both Zambia and South Africa by an experienced social science team. The research will be conducted in two phases. A first rapid phase using participatory social research methods carried out in all communities will be described and conducted in an ancillary protocol. A second in-depth phase, with a longitudinal component, related to the different arms of the trial and core questions around uptake and outcomes, is described below.

The first phase will identify key features of each community (including social organization and networks) and will involve community mapping of the history of ART, local HIV prevention initiatives, HIV treatment and support services, and key stakeholders (including other HIV research studies). This and initial work on community attitudes to different prevention methods will help to inform the design and delivery of the trial interventions (including the design and content of information/sensitization messages and instruments) and to enable effective stakeholder co-ordination in all communities. In principle, the qualitative studies will work closely with the community engagement process throughout the study.

In the second phase, the qualitative research will have three core components, namely: qualitative research evaluating the acceptability of the intervention including, critically, the acceptability and functioning of the CHiPs and the process of community engagement; a qualitative longitudinal study of representative individuals nested within the first *Case-Control* study described above; and an ethnographic component. These are briefly detailed below.

5.4.1 Evaluation of the Acceptability of the Intervention:

In Arms A and B, social science research will be carried out at community level using a mix of social research methods (including fieldworker structured diaries, in-depth interviews, focus-group discussion, participatory rapid appraisal tools, participant observation, structured observation) to assess over time popular understanding of HIV testing and treatment and how communities actually respond to the combination prevention intervention, including linkage to care and the innovation of immediate HIV treatment. This research component will be carried out throughout the intervention period at intervals linked to the intervention timeline – e.g. at the outset, three months into the intervention, a year into the intervention and towards the end of the intervention. Comparative research will also be carried out in Arm C – evaluating community response to HIV testing and treatment in the absence of trial interventions. Following full implementation of protocol version 3.0, social science research will be conducted on the

transition to immediate eligibility for ART in Arm B and C communities and the continued response to this change over time.

Building on the rapid formative research, qualitative insights will be collected in a structured diary form throughout the intervention period from all communities using resident fieldworkers who would dedicate a few days a month to document local response. More in-depth work will also be carried out in communities of a certain type. In these communities, roughly 100 participants, including key local stakeholders, CHIP teams and different age and gender groups from the community, will be questioned about the acceptability of the intervention, any problems experienced or foreseen, and suggested solutions to these problems, and findings will be fed back into community engagement and trial practice. Research on the process of community engagement and the application of ethical guidelines will also be embedded within this component. In addition, this component will include any urgent research on significant events at community level (e.g. significant rumors including Satanism accusations, community withdrawal, explicit confrontation with faith healing or other alternative prevention options) which threaten the continuation or practice of the trial and require qualitative investigation.

5.4.2 Qualitative Longitudinal Study in Arms A and B – sub-set of *Case-Control Study 1*

A small number (roughly 12 in each selected community) of representative individuals from *Case-Control Study 1* will be enrolled and seen longitudinally over the intervention period in selected communities across Arms A and B to explore and document the longitudinal trajectory of individual behavior in relation to uptake of HIV testing and treatment, complementing the findings of the case-control studies.

Individuals who have refused testing at baseline and individuals who have accepted testing with different outcomes (tested HIV-uninfected or HIV-infected) from different genders, age groups, and socio-demographic backgrounds will be selected and approached to participate in this longitudinal study. This cohort of individuals would be recruited following their participation in *Case-Control Study 1*– with the first in-depth interview taking place after the *Case-Control Study 1*survey, and subsequent in-depth interviews being held at one to three month intervals until the end of the intervention period. This research will document experiences over time and establish how the micro-level continuum of experiences influence decision making processes related to uptake of HIV testing and treatment services. Additional locator information will be collected and separate informed consent obtained for the study for each visit.

A mix of methods will be used including semi-structured interviews, observations and respondent records of significant events pertaining to individual health and health seeking behavior. In-depth interviews will be conducted by local social science research assistants supervised by a social scientist.

As a result of changes to the protocol in version 3.0, and in addition to the above, a small number of individuals in some Arm C communities representing different decisions concerning HIV testing and treatment (following the shift to immediate eligibility to ART) will also be recruited and followed to the end of the intervention period.

5.4.3 Ethnography of the HIV landscape

This component aims to provide more contextual and comparative understanding of how communities are experiencing the roll-out of UTT, including immediate HIV treatment. The inquiry will build on and extend current knowledge of the impact of ART on HIV stigma, the long-term realities of ART in low-resource settings, the influence of alternative prevention options, the role of welfare and food insecurity in shaping uptake of ART, popular knowledge of ART, sexual risk disinhibition, alcohol and drug use, gender-based violence, HIV identity, the reproductive health of people living with HIV, the acceptability of and response to male circumcision, the influence of local systems, social networks and community morale, and the role of different stakeholders. This ethnographic research will use a mix of social research methods – with the most key method being the continued presence of a social scientist in a community over a period of 3-6 months, mostly likely 6-18 months into the intervention period. It will be carried out in two communities in each country and most of the inquiry will be carried out at household level.

5.4.4 Graphical Summary of Qualitative Activities

A summary of the timing, flow and logic of the qualitative activities is provided in Figure 4.

Figure 4- Qualitative Activities in HPTN 071

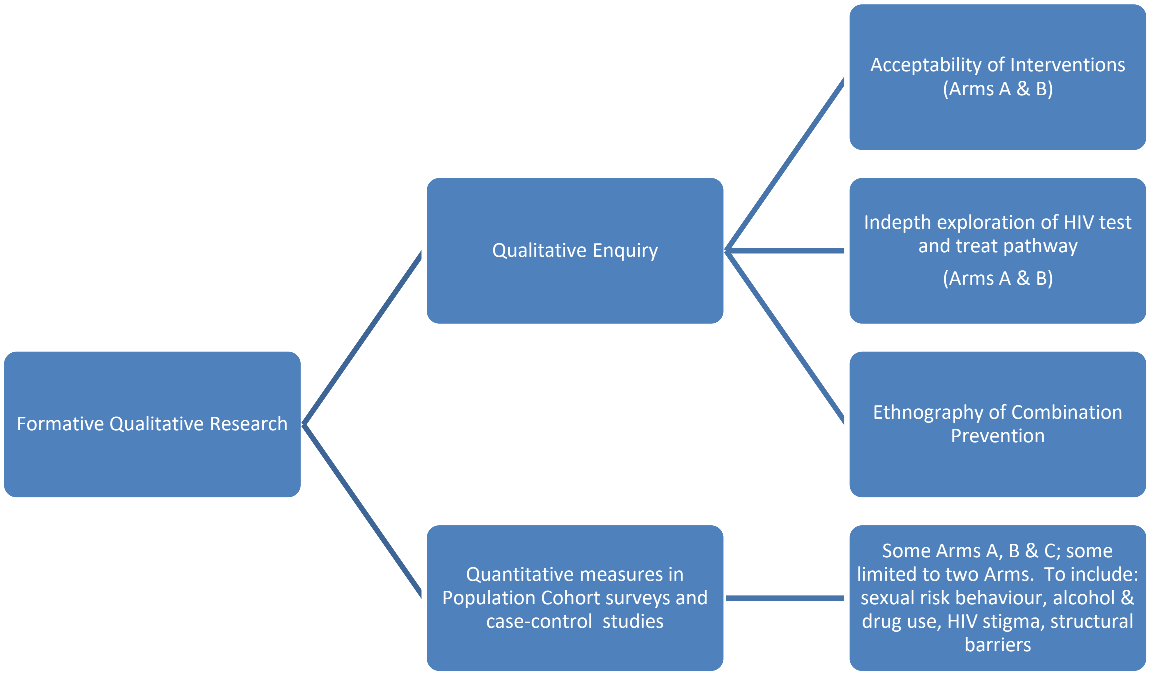

5.4.5 Integration of Data from Case-Control and Qualitative Components

The social science team will be involved in helping to develop the themes and questions for the case-control studies. Within the case-control studies there is a qualitative component that aims to provide a more detailed picture of HIV testing and treatment pathways for a small number of representative individuals. The lead investigator for the case control studies will work closely with a social scientist who will carry out the qualitative component as well as supporting the quantitative measures/data collection. Broader ethnographic enquiry will also explore core themes (all related to secondary outcomes).

5.5 Collection of Health Center-Based Data

In addition to the conduct of specific surveys described in previous sections, routine health center-based data in all study communities will be used to measure several secondary outcomes. To maximize the validity of this information, the research team will work with the health centers to improve the collection and management of these routine data and to the extent possible, harmonize these processes across health centers.

5.5.1 Tuberculosis Case Notification

TB cases in the study communities are routinely diagnosed and treated at the same health centers as those delivering HIV treatment and care. In all of the study communities, the

TB notification process will be strengthened by the use of additional diagnostic tests and enhanced monitoring of the TB case registration system. Data from this system will be compiled at regular intervals during the trial and used to measure the following outcomes:

- Notification rate of bacteriologically confirmed pulmonary tuberculosis
- Mortality rate of bacteriologically confirmed pulmonary tuberculosis

These data will be collected for each time period, and will be classified according to HIV status.

5.5.2 Intervention Effect on Health Center Workload

To address concerns that the intervention may substantially add to the case-load of clinics, data from clinic registers will be compiled at regular intervals to determine the total numbers of outpatient and inpatient attendances. These data will be collected at regular time intervals, and efforts will be made to broadly classify medical reasons for attendance.

5.5.3 Intervention Effect on Healthcare Costs

A multi-step procedure will be used to determine the impact of the intervention on healthcare costs. First, data will be collected on individuals' healthcare utilization by self-report of the members of the *Population Cohort*. Data will be collected on use of outpatient healthcare facilities (number of visits) and secondary and tertiary facilities (number of visits, duration of visit if overnight). Direct costs of providing care to individuals will also be evaluated, including travel costs and reimbursements by third parties (e.g. private insurers). In addition, *Population Cohort* patient records held at the healthcare facilities along with CHiPs data will be reviewed where available to obtain detailed information on healthcare use across treatment arms. We will use both participant self-report and clinic records in order to generate population level estimates of changes in health care utilization, document care sought from providers where linkage to records is not possible, and collect data on patients' cost of seeking care. Second, facility level costs will be collected in all facilities in selected trial communities. Both one-time capital costs (e.g. investments in buildings) and recurrent costs (e.g. salaries) will be collected. Lastly, facility level costs will be apportioned to the visits reported by the cohort members by applying average costs for typical use of healthcare facilities. For members with linked patient records, we will calculate more precise cost estimates of health care use based on actual treatments provided. This final analysis will be based on various assumptions, which will be tested with sensitivity analysis.

5.5.4 HIV Disease Progression and Death

Aggregate data from the health center database for health center attendees on ART such as WHO staging events (including opportunistic infections such as TB), hospitalizations (where documented), CD4 cell counts, and death will be used to monitor effects of the interventions on HIV disease progression and death.

5.5.5 ART Adherence and ART Toxicity

To assess the rates of ART adherence under different intervention conditions, aggregate data will be collected from all health centers about missed follow-up visits among those on ART and missed dispensations of ARV drugs. To assess rates of ART toxicity under different intervention conditions, aggregate clinical data related to ART related side effects, ART drug interruptions and treatment switches will also be collected from each health center.

5.5.6 Uptake of Intervention Components

Process measures of the uptake of key components of the intervention will be measured in Arms A and B using data from the health centers on the rates of utilization of PMTCT services and medical male circumcision and the proportion of community members initiating HIV care within three months of receiving an HIV diagnosis.

5.6 Proposed Additional Surveys

Three additional surveys have been proposed to support or enhance the research described above. The three surveys have not been described in detail in the body of this protocol because funding is not available for them at the current time. However, each is briefly described below and described in detail in Appendices VIII, VI and X.

Population Cross-Sectional Survey (Appendix VIII)
Because participants in the *Population Cohort* will be followed longitudinally over 3 years, their interactions with the research staff could bias the data they provide for certain outcome measures. The *Population Cross-Sectional Survey* would be a snapshot evaluation to provide unbiased data for comparison on many of the measures evaluated in the *Population Cohort*. Approximately 500 participants per cluster would be recruited from randomly-selected homes for this survey, to be conducted at the end of the second year of the intervention. Procedures would include a questionnaire and blood sampling for HIV testing and sample storage.

5.7 Comparative Table of Study Activities across All Study Arms

Table 3- Study Activities across All Study Arms

Study Procedures/ Activity	Arm A	Arm B	Arm C
**Strengthening the provision of HIV services in the community**			
Endeavour to ensure ART service delivery to at least local guidelines	X	X	X
Endeavour to ensure PMTCT services to at least local guidelines	X	X	X
Endeavour to ensure STI treatment to at least local guidelines	X	X	X
Endeavour to ensure male circumcision services to at least local guidelines	X	X	X
Promotion of voluntary counseling and testing at non-HIV clinics and other venues	X	X	X[1]
Opt-out provider-initiated counseling and testing	X	X	X[1]
**Implementation of interventions** including deployment of CHiP teams to all houses in the community			
Offering initial and recurrent HIV testing and counseling to all household members aged 16 years or above in Zambia/12 years or above in South Africa, and younger children with parental consent	X	X	
Linkage-to-care for HIV-infected persons	X	X	
Immediate eligibility for ART	X	X[2]	X[2]
Eligibility for ART based on local guidelines		X[2]	X[2]
Referral of willing HIV-uninfected men for circumcision	X	X	
Referral of pregnant, HIV-infected women to PMTCT services or immediate ART	X	X	
Ongoing promotion of ART adherence and HIV prevention services during the study period	X	X	
**Enrollment and follow-up of *Population Cohort*** by research team			
Informed consent	X	X	X
Offer of HIV rapid test and counseling	X	X	X
Complete survey to include socio-demographic, health, social, behavioral, and economic factors	X	X	X
Blood draw and laboratory-based testing<br>See Sections 5.1 and Appendix IA.	X	X	X
**Execution of a *Population Cross-Sectional Survey*** at 36 months (if funded)			
Same procedures as for Population Cohort, but without HSV-2 testing	X	X	X
**Conduct of qualitative studies**			
Informed consent	X	X	X
Completion of qualitative data collection	X	X	X
**Conduct of *Case-Control* studies**			
Informed consent	X	X	
Completion of behavioral questionnaire	X	X	

[1]Where these are already provided locally as standard services.
[2]ART eligibility according to local guidelines prior to protocol versions 3.0; immediate eligibility after full implementation of protocol version 3.0.

6.0 SAFETY MONITORING AND SOCIAL HARM REPORTING

6.1 Safety Monitoring

All drugs used in this study for the treatment of HIV have regulatory approval for this purpose in both Zambia and South Africa and are widely used with well-established safety profiles. Community members receiving ART will be seen by the regular staff at the health center for their care and will receive safety assessments according to local standard of care. Data from these tests are not routinely entered into electronic medical records at the health center. The research team therefore will not have access to, nor monitor or report adverse events/serious adverse events (AE/SAEs) for community members on ART. Instead, information about the impact of the different interventions on the health and safety of community members on ART will be assessed through analysis of aggregate, anonymous data from the health centers, and data from *Population Cohort* participants, including measures of HIV disease progression and death, self-reported drug adherence, ART toxicity, and, if funded, viral suppression, drug resistance, and community viral load.

6.2 Social Harm Reporting

The HPTN defines social harms as any untoward social occurrences that happen to a **participant as a result of their participation in the study,** with examples including loss of employment, harassment by neighbors, shunned by family, rejection by partner, etc. Because this study is a community-randomized trial of a multi-faceted intervention, the majority of people in the community affected by the implementation of the study will not be participants in the evaluation surveys, and so the definition of social harm for this study will be expanded to also include **any untoward social occurrences that happen to a community, or groups or individuals within a community, as a result of implementation of the study intervention**. Social harms will be monitored throughout the study.

It is important to note that the number of people who live in the communities involved in this study is very large and the number of social harms *unrelated to the study intervention or study participation* that will occur during the trial period is expected to be very high due to social, economic and cultural factors unrelated to the study. Therefore it will be important that study staff are well trained to report only those social harms that they deem to be directly related to the intervention, or participation in the research program.

6.2.1 Participants in the *Population Cohort*

The *Population Cohort* is intended to provide study data representative of the populations from which it is drawn, and this will apply for social harms monitoring as well. Information on social harms experienced by cohort participants - either because of the deployment of the intervention in their community or because of their individual participation as research subjects in the cohort - will be actively solicited from participants at follow-up visits and captured in the study database. When a cohort participant reports a social harm, every effort will be made by the study staff to provide appropriate counseling to the participants, and/or referral to appropriate resources, as needed.

6.2.2 Community at Large

Monitoring of social harms in the community will be accomplished using several approaches. In each household during their annual testing visit, CHiPs will inquire about any social harms due to the implementation of the intervention in their community, and will document qualifying harms in the study database. Because study staff work intimately among, and are often from, the randomized communities, they may become aware on a passive basis of social harms that are occurring within the community. Staff will report these harms as well. The study team will include discussion of social harms as one of the topics regularly covered in work with the community liaison board in each community, and will report on any harms reported in those meetings. Finally, the qualitative research to be conducted includes exploration of social harms in the community.

6.2.3 Social Harm Monitoring

The study management team will review the social harms reports on a quarterly basis, or sooner, if a concerning trend or event is identified. If the management team judges an individual social harm, or a trend in social harms, to be serious or unexpected, they will work together with appropriate bodies (in-country investigator, community liaison board, sponsor, IRB, etc.) to determine if a response is indicated, and if so, what it should be. The nature and frequency of reported social harms will be reviewed by the HPTN Study Monitoring Committee (SMC). Investigators of record will report serious or unexpected social harms to the responsible IRB/EC at least annually, or according to their individual requirements. The study team notes that although most of the activities for detecting social harms listed above will be conducted equally in all three arms of the study, CHiPs will only be deployed in Arms A and B. Therefore a greater number of reported social harms may be seen in these arms, relative to Arm C, due to differential ascertainment, rather than differential incidence of harms, a possibility that will be considered when reviewing trends in social harms.

7.0 STATISTICAL CONSIDERATIONS AND DATA ANALYSIS

7.1 Sample Size

The trial has been powered to detect intervention impact on the primary endpoint, and on key secondary endpoints, as detailed below. All sample size calculations have been carried out using methods for matched cluster-randomized trials.

7.1.1 Mathematical Modeling and Sample Size Calculations

The development of the interventions has been guided by the results of mathematical modeling. Early work on the intervention was based on the papers by Granich et al. Subsequently, the modeling team at Imperial College developed a model fitted to current UNAIDS prevalence data from Zambia and South Africa and used it to predict the impact of the proposed packages of interventions relative to the standard of care arm (Figure 5).

Figure 5- Mathematical model of the epidemic and of the PopART interventions

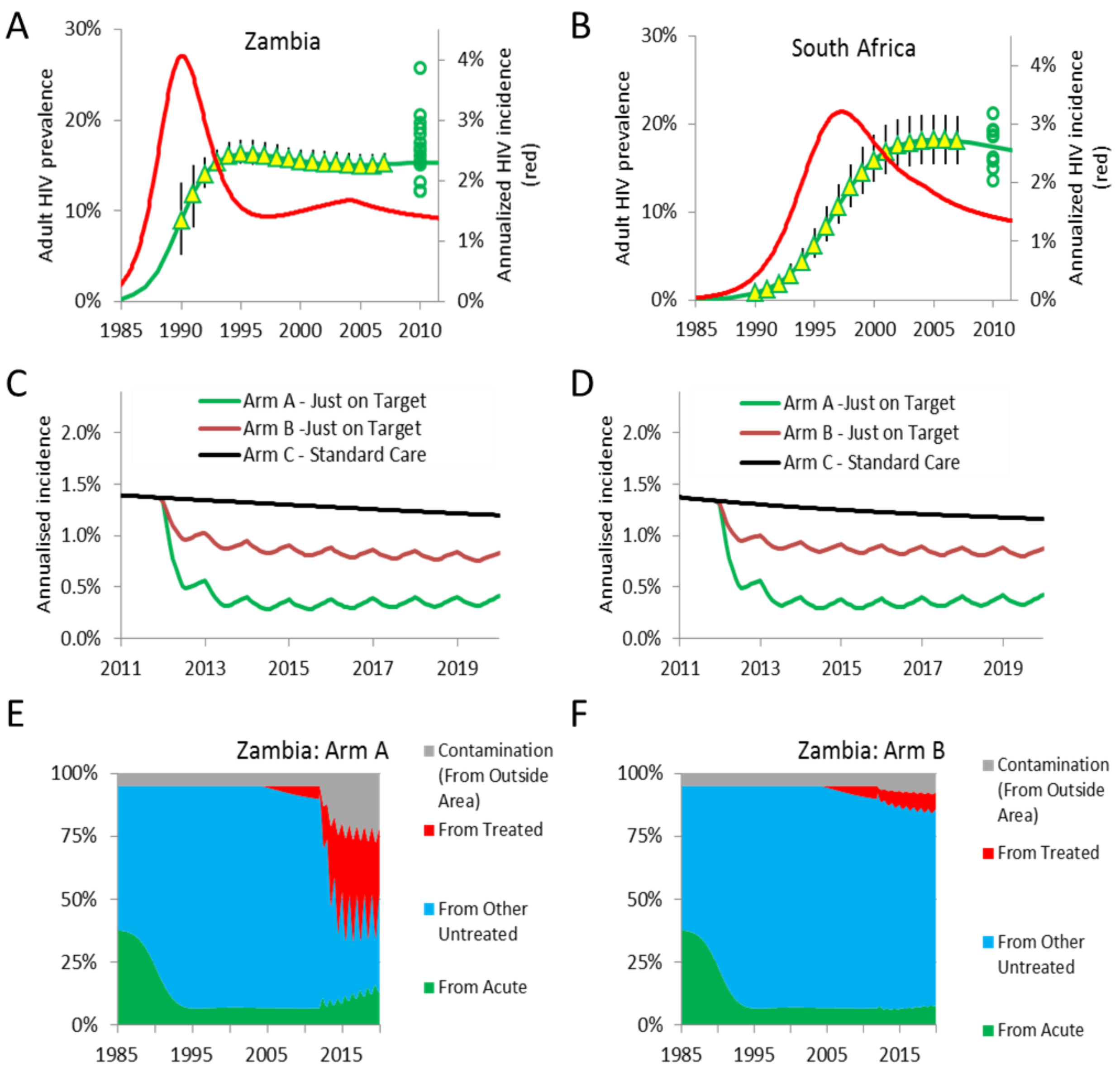

A model was developed to aid development of the trial protocol, and more specifically to develop targets for the process variables (coverage, contamination, etc.), and to provide scenarios for the power calculations. The model is a conventional HIV epidemic model, and has been validated by a recent systematic model comparison exercise (Eaton et al, submitted). The model is calibrated to country-specific UNAIDS data on adult HIV prevalence (green lines in **A** and **B**). Prevalence in the 24 ZAMSTAR communities in 2010 is shown by the green circles, and the predicted incidence curves are shown as red lines. **C** and **D**, starting from 2012, the PopART intervention package is implemented; the packages are implemented in six-monthly cycles, which results in a characteristic 'saw-tooth' pattern in incidence. The Just-on-target scenarios are based on the optimistic scenario (75% annual coverage, 95% treatment efficacy, 5% contamination, 50% uptake of male circumcision, 10% annual drop out and no behavior change). **E**, predicted sources of infection for incident cases for Arm A in Zambia, and **F** as **E** for Arm B.

.

Briefly, the model assumes three sexual activity classes, and the proportions in each class, the assortativity in sexual mixing, rates of partner acquisition and HIV transmissibility are fitted to the prevalence data in each country. The model assumes that male circumcision reduces HIV acquisition by 60%, with rates of male circumcision based on data from the study communities (Table 2- Section 4.1). ART roll-out is assumed to commence in 2004 with coverage amongst those with CD4<200 and CD4<350 matched to ZAMSTAR data.[102] The model includes variable infectivity by stage of infection, matched to data from the Rakai study.[68, 103] To allow for contamination, we assumed that 5% of sexual contacts occur with partners from outside the study community. The fit of the model to the HIV prevalence data is considered to be good.

The model fits assume that interventions commence in 2012, and that during each annual round of testing in Arms A and B, the intervention is delivered over a period of 26 weeks. Figure 5 shows projected HIV incidence over time for Arms A and B compared with the control arm for the optimistic target scenario. Table 4 shows the assumptions made for the central and optimistic target scenarios and the projected impact on cumulative HIV incidence over three years, over the first two years, and also in each year separately, for Arms A and B compared with Arm C. The projections indicate that an impact is expected over three years of 55-65% in Arm A and 20-30% in Arm B. Impact is substantially higher in Years 2 and 3 as expected. As a sensitivity analysis, assuming roll-out takes 12 rather than 6 months, projected impact over three years is 50-60% for Arm A and 20-30% for Arm B (Table 5).

Table 4- Parameter values assumed for the model of the impact of the intervention for central and optimistic target scenarios, and projected impact on HIV incidence in Arms A and B compared with Arm C, assuming intervention roll-out over a 6-month time period

	Parameter	Central Target		Optimistic Target	
	Annual coverage of test and treat campaign	70%		75%	
	Treatment failure & drop-out rate, per year	10%		10%	
	Effectiveness of ART in blocking transmission	90%		95%	
	Take up of male circumcision when offered	50%		50%	
		Arm A	Arm B	Arm A	Arm B
Zambia	Impact on cumulative incidence (3 years)	61%	25%	63%	27%
	Impact on cumulative incidence (2 first years)	58%	24%	61%	25%
	Impact on HIV incidence during Year 1	51%	20%	54%	21%
	Impact on HIV incidence during Year 2	65%	27%	67%	28%
	Impact on HIV incidence during Year 3	67%	29%	68%	30%
South Africa	Impact on cumulative incidence (3 years)	62%	26%	64%	27%
	Impact on cumulative incidence (2 first years)	59%	25%	61%	26%
	Impact on HIV incidence during Year 1	52%	22%	55%	23%
	Impact on HIV incidence during Year 2	65%	28%	67%	29%
	Impact on HIV incidence during Year 3	68%	29%	69%	30%

Table 5- Parameter values assumed for the model of the impact of the intervention for central and optimistic target scenarios, and projected impact on HIV incidence in Arms A and B compared with Arm C, assuming intervention roll-out over a 12-month time period

	Parameter	Central Target		Optimistic Target	
	Annual coverage of test and treat campaign	70%		75%	
	Treatment failure & drop-out rate, per year	10%		10%	
	Effectiveness of ART in blocking transmission	90%		95%	
	Take up of male circumcision when offered	50%		50%	
		Arm A	Arm B	Arm A	Arm B
Zambia	Impact on cumulative incidence (3 years)	58%	24%	60%	25%
	Impact on cumulative incidence (2 first years)	53%	21%	56%	22%
	Impact on HIV incidence during Year 1	42%	16%	45%	17%
	Impact on HIV incidence during Year 2	64%	27%	66%	28%
	Impact on HIV incidence during Year 3	68%	29%	69%	31%
South Africa	Impact on cumulative incidence (3 years)	59%	25%	61%	26%
	Impact on cumulative incidence (2 first years)	54%	23%	57%	24%
	Impact on HIV incidence during Year 1	44%	18%	47%	19%
	Impact on HIV incidence during Year 2	64%	27%	67%	29%
	Impact on HIV incidence during Year 3	68%	30%	70%	31%

The targets appear achievable based on published evaluations of interventions in Africa. Because there is most uncertainty in the effects on behavior change, the model conservatively assumed no effect on behavior when deriving these targets. Process indicators will be monitored as described in Sections 7.1.3 (6) and 7.10, and used to modify and adapt the intervention as necessary.

7.1.2 Primary Endpoint - HIV Incidence Over 36 Months

The incidence of HIV infection among initially HIV-uninfected *Population Cohort* members will be measured during the follow-up period of 36 months. Based on national estimates of HIV incidence and on HIV prevalence in the chosen study areas, it is expected that HIV incidence in the control arm will be in the range 1.0-1.5/100py. With a matched study design, and based on the between-community variation in HIV prevalence observed in the 2010 survey of several thousand adults in each of the trial communities, and the between-community variation in HIV incidence among adults living in the households of TB cases during 2006-2010, it is expected that the between-community coefficient of variation will be in the range 0.15-0.20. Seven communities were chosen per study arm and a *Population Cohort* of 2,500 adults per community to attain adequate power to detect a difference in incidence between Arms A and C (reflecting the full impact of the intervention), as well as the difference in intervention effect between Arms A and B (reflecting the additional effect of immediate HIV treatment compared with current local guidelines). Based on mathematical modeling, the anticipated effect of Arms

A and B is to reduce cumulative HIV incidence over a three-year period by 55-65% and 20-30% respectively, compared with Arm C (Figure 5), with a difference in impact between Arms A and B of about 30-35%. A standard formula for cluster-randomized trials was used for the comparison of incidence rates over 36 months, with matched triplets as the trial design[104].

Table 6 shows that the study will be very well powered to detect an effect of 35% or larger in Arm A compared with Arm C, and moderately well powered to detect an effect of 30% under favorable assumptions. For the direct comparison of Arms A and B, Table 7 shows that the study will be well powered to detect a difference between effects of 60% and 30%, 55% and 25%, and 50% and 20%. Tables 6 and 7 allow for a baseline HIV prevalence of 15% and assume losses to follow-up of 20% over two years, and 25% over three years.

Table 6- Power for comparison of HIV incidence in Arm A or B with Arm C, with 7 communities per arm and Population Cohort of 2500 adults per community (assuming that on average 2125 (85%) will be HIV-uninfected at baseline and that loss to follow-up will be 20% after 2 years and 25% after 3 years) with 5206 person-years per community over 36 months (assuming 1912 person-years 0-12 months; 1700 person-years 12-24 months; 1594 person-years 24-36 months)

HIV incidence rate/ 100py (control arm)	Between-cluster coefficient of variation (k)	Effectiveness (%)	Power (%)
1.0	0.15	25%	57%
1.0	0.15	30%	74%
1.0	0.15	35%	87%
1.0	0.15	40%	95%
1.0	0.15	45%	99%
1.0	0.15	50%	100%
1.0	0.15	55%	100%
1.0	0.15	60%	100%
1.0	0.15	65%	100%
1.0	0.20	25%	44%
1.0	0.20	30%	60%
1.0	0.20	35%	75%
1.0	0.20	40%	87%
1.0	0.20	45%	94%
1.0	0.20	50%	98%
1.0	0.20	55%	99%
1.0	0.20	60%	100%
1.0	0.20	65%	100%
1.5	0.15	25%	64%
1.5	0.15	30%	81%
1.5	0.15	35%	92%
1.5	0.15	40%	98%
1.5	0.15	45%	100%
1.5	0.15	50%	100%
1.5	0.15	55%	100%
1.5	0.15	60%	100%
1.5	0.15	65%	100%
1.5	0.20	25%	48%
1.5	0.20	30%	65%
1.5	0.20	35%	80%
1.5	0.20	40%	91%

1.5	0.20	45%	96%
1.5	0.20	50%	99%
1.5	0.20	55%	100%
1.5	0.20	60%	100%
1.5	0.20	65%	100%

Table 7- Power for comparison of HIV incidence between Arms A and B, with 7 communities per arm and Population Cohort of 2500 adults per community (assuming that on average 2125 (85%) will be HIV-uninfected at baseline and that loss to follow-up will be 20% after 2 years and 25% after 3 years)

HIV incidence rate/ 100py (control arm)	Between-cluster coefficient of variation (k)	Effectiveness (%) Arm A	Effectiveness (%) Arm B	Power (%)
1.0	0.15	50%	20%	89%
1.0	0.15	50%	25%	78%
1.0	0.15	55%	25%	92%
1.0	0.15	55%	30%	82%
1.0	0.15	60%	25%	98%
1.0	0.15	60%	30%	94%
1.0	0.15	65%	25%	99%
1.0	0.15	65%	30%	99%
1.0	0.20	50%	20%	78%
1.0	0.20	50%	25%	65%
1.0	0.20	55%	25%	83%
1.0	0.20	55%	30%	71%
1.0	0.20	60%	25%	93%
1.0	0.20	60%	30%	87%
1.0	0.20	65%	25%	98%
1.0	0.20	65%	30%	96%
1.5	0.15	50%	20%	94%
1.5	0.15	50%	25%	86%
1.5	0.15	55%	25%	96%
1.5	0.15	55%	30%	90%
1.5	0.15	60%	25%	99%
1.5	0.15	60%	30%	98%
1.05	0.2015	65%	25%	99%
1.05	0.2015	65%	30%	99%
1.5	0.20	50%	20%	84%
1.5	0.20	50%	25%	72%
1.5	0.20	55%	25%	88%

1.5	0.20	55%	30%	78%
1.5	0.20	60%	25%	96%
1.5	0.20	60%	30%	92%
1.05	0.20	65%	25%	99%
1.05	0.20	65%	30%	98%

While the study is adequately powered to answer the primary research question, it has not been powered to undertake any stratified analysis by country or to assess difference in impact between countries.

7.1.3 Secondary Endpoints

Note: Tables showing the calculations for secondary endpoints are provided in Appendix VII.

(1) HIV Incidence During Months 12-24, and Months 24-36 from Start of Intervention

Assuming baseline HIV prevalence of 15% and loss to follow-up of around 20% by the end of Year 2 and 25% by the end of Year 3, this incidence estimate will be based on a sample size of approximately 1700 person-years per community in Year 2 and 1594 person-years per community in Year 3.

Our model projections show that under the optimistic scenario the impact on HIV incidence during Year 2 will be 70% and 30-35% in Arms A and B respectively, and for the central target it will be 60% and 25-30% respectively, with a difference in impact between Arms A and B of about 35%. For a comparison of Arm A with Arm C, study power is 96% or higher with the central target of a 60% reduction, with k up to 0.20 and HIV incidence in Arm C of at least 1 per 100 person-years. For a comparison of Arm B with Arm C, study power is 71% with the optimistic target of a 35% reduction, k=0.15 and HIV incidence in Arm C of 1.5 per 100 person-years, but lower than this for the central target, and/or higher k, and/or lower HIV incidence in Arm C. For comparison of Arms A and B, we will have moderate power (around 70% or more) for the central target and 1% HIV incidence, and good power (>80%) for all other conditions.

Our model projections show that under the optimistic scenario the impact on HIV incidence during Year 3 will be approximately 75% and 35% in Arms A and B respectively, and for the central target it will be 65-70% and 30% respectively, with a difference in impact between Arms A and B of about 35%. For a comparison of Arm A with Arm C, study power is 98% or higher with the central target of a 65% reduction, with k up to 0.20 and HIV incidence in Arm C of at least 1 per 100 person-years. For a comparison of Arm B with Arm C, study power is 69% with the optimistic target of a 35% reduction, k=0.15 and HIV incidence in Arm C of 1.5 per 100 person-years, but lower than this for the central target, and/or higher k, and/or lower HIV incidence in Arm C. For

comparison of Arms A and B, we will have good power for the central target of 65-70% vs 30% (74%-97% power depending on assumptions).

(2) Community Viral Load 12, 24, and 36 Months after the Start of Intervention (Subject to Funding)

In the *Population Cohort* at 24 months, viral load will be measured in all HIV-infected individuals (irrespective of seroconversion date), estimated to be approximately 300 in each community (subject to funding for HIV viral load testing).

Assuming that the mean of $\log_{10}$ (viral load) is 4 in Arm C, that k=0.15 and the standard deviation of viral load within communities is 0.9 on the $\log_{10}$ scale, there is 84% power to show a reduction of 1 in $\log_{10}$ viral load in each of the other two trial arms. Alternatively, comparisons between arms can be made on the basis of what proportion of HIV-infected individuals have undetectable viral load. Assuming these proportions are 20% in Arm C, 40% in Arm B, and 60% in Arm A, the study is well powered to show a difference between Arms A and B, and very well powered to show a difference between Arms A or B and Arm C.

At 12 and 36 months, viral load will be measured in approximately 75 HIV-infected individuals in each community (subject to funding for HIV viral load testing). Assuming 20% with undetectable viral load in Arm C, 40% in Arm B, and 60% in Arm A, and k=0.20, there is 77% power to show a difference between Arms A and B and 97% power to show a difference between Arm B and Arm C.

(3) HSV-2 Incidence Over 36 Months

This will be measured in the *Population Cohort*. Assuming that baseline HSV-2 prevalence is approximately 70% and that by 24 months the loss to follow-up is 20% and by 36 months it is 25%, the estimate of intervention effect on HSV-2 incidence will be based on 1837 person-years per community. If HSV-2 incidence in Arm C is approximately 5 per 100 person-years, there is >90% power to detect an increase to 7.5 per 100 person-years or a reduction to 3.0 per 100 person-years if k = 0.15, and 80-90% power to detect such effects if k = 0.20.

(4) Retention in HIV Care, and Viral Load Suppression and Drug Resistance Among HIV-Infected Individuals Who Are Taking ART

These outcomes will be measured in HIV-positive participants in the *Population Cohort*.

(i) Retention in care at 12 months after registering for HIV care

This will be measured in HIV-positive participants who present for HIV care for the first time after the start of the intervention period, an estimated 198 per community in Arms A and B, and 99 per community in Arm C. First, assuming that retention in care at 12 months is 85% in Arm C, and that k=0.2, there is 85% power to show a reduction to 75%, and >95% power to show an increase to 95%, in each of Arms A and B. Second, assuming that retention in care is 90% in Arm

C, there is 96% power to show a reduction to 80% in each of Arms A and B, and 79% power to show an increase to 95%. Third, assuming retention in Arm C is 80%, there is 71% power to show a reduction to 70% in each of Arms A and B, and 94% power to show an increase to 90%.

(ii) Viral load suppression, and drug resistance, measured among HIV-positive members of the *Population Cohort* at 24 months (subject to funding)

Sample size calculations assume that, by the time of the 24-month follow-up in the *Population Cohort*, and among individuals who registered for HIV care for the first time after the start of the intervention period, 67% of patients will have started ART in Arm C, 50% in Arm B and80% in Arm A; and that 80% of patients will participate in the *Population Cohort* survey at 24 months. This gives sample sizes in each community of 141, 88 and 59 patients who start ART and will be available for viral load and drug resistance measurement in Arms A, B, and C respectively.

Assuming 10% are not virally suppressed in Arm C, there is 91% power to show an increase to 20% in Arm A and 63% power to show a reduction to 5%. The corresponding figures for a comparison with Arm B are 86% and 60%.

The percentage of patients with acquired drug resistance will be a subset of those who are not virally suppressed, but the range of scenarios considered above includes plausible values for this endpoint as well.

(5) Case-Control Studies

Three Case-Control studies will be conducted as follows:

(i) Uptake of CHiP home-based HIV testing during Round 1 of intervention in Arms A and B, with cases selected at random from individuals who did not accept testing, and controls selected at random from individuals who accepted testing;

(ii) Acceptance of immediate ART in Arm A, among individuals who were first diagnosed, or self-reported, as HIV-infected as part of CHiP home-based testing in Round 1 of intervention and who were not already on ART. Controls are selected from among individuals who started ART within 6 months of being identified as HIV-infected and referred for HIV care by CHiPs, and cases from among individuals who did not;

(iii) Uptake of CHiP home-based HIV testing during Round 2 of intervention in Arms A and B, excluding individuals who were diagnosed as HIV-infected in Round 1, and also individuals who self-reported they were HIV-infected in Round 1 or Round 2.

Calculations assume 400 cases and 400 controls, for each of the three case-control studies, assuming an unmatched design. Cases are individuals who refuse HIV testing, re-testing for HIV, or immediate ART for studies (1)-(3) respectively; controls are individuals who accept testing, re-testing for HIV and immediate ART for studies (1)-(3) respectively.

Assuming that the percentage of controls exposed to a particular risk factor is 10%, 15%, or 20%, and that the odds ratio comparing exposed with unexposed individuals is 1.75, the corresponding study power to show an effect of the risk factor is 71%, 85%, and 91% respectively. With an odds ratio of 2, the corresponding figures for study power are 90%, 97%, and 99% respectively. When the proportion of controls exposed to a particular risk factor is 15% or more, the sample size is sufficient for stratified analyses, such as separate analyses by country or by gender. For example, with 200 cases and controls for women, and an odds ratio of 2, then if 15% and 20% of controls respectively are exposed to the risk factor, study power is 75% and 83% respectively.

Table 8- Case-control studies of (1) uptake of HIV testing (2) uptake of re-testing for HIV and (3) uptake of immediate ART among HIV-positive individuals

Percentage of controls with a risk factor	Odds ratio for refusing testing/re-testing/immediate ART, comparing individuals with a risk factor characteristic to those without	Power (%)
10%	1.75	71%
15%	1.75	85%
20%	1.75	91%
10%	2.0	90%
15%	2.0	97%
20%	2.0	99%

(6) Process Measures – Uptake of HIV Testing, ART and Male Circumcision

(a) CHiP Data – Arms A and B

(i) HIV testing uptake

With an average community adult population of 25000, and acceptance of home-based HIV testing in the range 50-80% in each round of testing, in each community the 95% confidence interval for testing uptake will be +/-1-2% of the point estimate.

(ii) Screening for ART eligibility, and uptake of ART, among HIV-infected individuals

If it is assumed that 80% of individuals accept CHiP home-based HIV testing in the first round of testing, that 15% are HIV-infected, and that 25% of HIV-infected individuals are already taking ART, approximately 2250 ART-naïve HIV-infected individuals will be identified through home-based testing in each community. With uptake of immediate ART in Arm A, and screening for ART

eligibility in Arm B, in the range 30-80%, in each community the 95% confidence interval for screening/uptake will be +/-2% of the point estimate.

(iii) Male circumcision

With an average community adult male population of 12500, there will be approximately 10625 HIV-uninfected men in each community. In the 2010 ZAMSTAR TB/HIV prevalence survey, in the Western Cape trial communities 77% of men aged 18 or above reported that they were circumcised, and in the Zambian trial communities 13%. So if uptake of CHiP home-based HIV testing in the first round of testing is 80%, an average of 1955 HIV-uninfected men will be eligible for medical male circumcision in each Western Cape trial community, and 7395 in each Zambian trial community. If 50% of these men are circumcised during the first year of trial intervention, the 95% confidence interval for the percentage who are circumcised will be +/-2% of the point estimate in Western Cape communities and +/-1% of the point estimate in Zambian communities.

(b) Arms A, B, and C – Population Cross-Sectional Survey at 24 Months

As noted above, funding has not been obtained to include a *Population Cross-Sectional Survey* as part of the study. If funded, the survey would provide additional useful data for all of the process measures described above for the *Population Cohort*. The analysis plan for this survey is described in Appendix VIII.

7.2 Random Assignment / Study Arm Assignment

Random assignment to study arms will take place at the cluster level. First, the 21 clusters will be matched into triplets based on best available estimates of HIV prevalence in the general adult population of these clusters, and taking into consideration geographic proximity of the sites to one another. This will be done separately in each country (stratified randomization), with 4 matched triplets in Zambia and 3 matched triplets in South Africa. The matched design will be used with the aim of minimizing the between-community variance in baseline HIV incidence, which is assumed to be correlated with baseline HIV prevalence.

After dividing the 21 clusters into 7 matched triplets, allocation to the three study arms will be carried out using a process of restricted randomization. This procedure will be used to ensure overall balance across study arms on cluster size, current ART uptake and HIV prevalence. There are $(3!)^7 = 279{,}936$ possible ways of allocating the clusters to the three study arms within matched triplets. These allocations will be evaluated against balance criteria to determine a restricted list of allocations that achieve adequate balance on the three variables defined above. The final allocation will be selected randomly from this restricted list of balanced allocations.

7.3 Statistical Analysis

The primary analysis will be based on a comparison of the incidence of HIV infection during the follow-up period of 3years between Arms A and C, Arms B and C, and Arms A and B. This will be carried out using appropriate analytical methods for cluster-randomized trials.

Because the number of clusters per arm is small, we will use methods based on Student's t-test, which have been shown to be highly robust for small numbers of clusters especially when sample sizes are similar in all clusters as in this study. We will compute the incidence of HIV infection in each cluster, weighted to take account of the sampling design which involves random selection of one adult from each household irrespective of household size. To test the null hypothesis of no impact, the paired t-test will be applied to these summary measures (7 matched pairs for each comparison), with 6df. The effectiveness of the intervention is defined as follows:

Protective effectiveness = 1 – RR

where the rate ratio (RR) is the ratio of incidence rates in the two study arms under comparison. This will be estimated by taking the geometric mean of the RR observed in each of the matched pairs, and a 95% confidence interval will be obtained using a normal approximation.

Evidence for intervention effect will also be assessed using a non-parametric permutation test, based on the list of all possible allocations of trial arms to communities that met the restricted randomization criteria. For each of these possible allocations, and including the allocation that was randomly selected, incidence rate ratios for intervention effect (comparing Arm A and Arm B, Arm B and Arm C, and Arm A and Arm C) will be calculated as above. The number of allocations (n) for which the incidence rate ratio is as extreme as or more extreme (further away from 1) than the value observed in the trial will be counted, and a 2-sided p-value calculated as n divided by the total number of possible allocations.

A description of how the analysis will control for migration contamination among the communities in the different treatment arms will be given in the Statistical Analysis Plan along with methods used to analyze the secondary outcomes.

Following the full implementation of changes to the protocol in version 3.0 such that Arm B communities will in future receive the full UTT intervention as in Arm A, it is envisaged that data on the primary outcome of HIV incidence will continue to be analysed in the three separate study arms, with separate comparisons between Arms A and C, Arms B and C, and Arms A and B. Effects on incidence will be analysed over the full three years of follow-up of the PC, and also in each individual year of follow-up. The results of these analyses, together with information on the timing of changes to the criteria for ART initiation in Arms B and C, will be used in combination with mathematical model outputs to derive information on the effects of UTT under different conditions of ART delivery. Full details of these analyses will be given in the Statistical Analysis Plan.

7.4 Interim Evaluation

As stated in Section 2.4, interim evaluation will take place during the first two years of intervention to determine whether to continue with the 36 month follow-up of the *Population Cohort* and the fourth year of intervention.

Because decisions on delivery of the intervention need to be taken at least 12 months in advance, to enable sufficient time for planning in the context of the annual PEPFAR funding cycle, the main evaluation is expected to be conducted in 2016 when the second round of intervention and the 12 month follow-up of the *Population Cohort* should be complete or close to completion (Figure 2).

The main criteria for evaluation will be:

- Observed HIV incidence in the control arm during the first 12 months of follow-up
- Measures of uptake and coverage of the intervention during the first two rounds of intervention

The evaluation will be carried out by the DSMB and detailed criteria for the evaluation will be agreed with the DSMB before the start of the trial.

7.4.1 HIV Incidence

Sample size calculations for the trial were based on an assumed HIV incidence of between 1.0 and 1.5 per 100 person-years in both Zambia and South Africa. While this assumption is consistent with epidemiological data from the study populations, there remains uncertainty about the current and future level of HIV incidence in the 21 study communities. The study may be under-powered if incidence is substantially below 1.0 per 100 person-years.

Data on estimated HIV incidence in the control arm (Arm C) based on the 12 month follow-up of the *Population Cohort* will be presented to the DSMB. These data will be prepared by a statistician independent of the study team so that they are not inadvertently unblinded to data on the effect size after 12 months. The DSMB will evaluate the implications of this incidence estimate on study power and will consider whether any change in the duration of the study would be appropriate. Note that results from the 12-month survey may not be available for a considerable time after sample collection is completed. This is because of the very large sample size of the PC, the need to perform HIV testing both in-country and at the HPTN LC, and the need to complete QA testing, including confirmation of HIV seroconversion, prior to data analysis.

Review of estimated effect size (by comparing HIV incidence between study arms) would be of limited value after 12 months of follow-up. If the effect is small, this would be consistent with a projected impact that increases steeply over time. If it is large, there would remain a need to measure the longer-term effects of the intervention including the occurrence of adverse effects.

7.4.2 Uptake of Intervention

Data on the uptake and coverage of the intervention components during the first two years of intervention delivery will be collated and presented to the Data Safety Monitoring Board (DSMB). These uptake statistics will be used to populate the mathematical model (Section 7.5) in order to obtain estimates of the projected effect of the interventions in Arms A and B relative to Arm C by time since the start of intervention roll-out. These projected estimates together with the HIV incidence estimates from the control arm will be used to obtain power estimates to guide a recommendation on the duration of follow-up.

The main purpose of the interim evaluation is to assess indicators of futility, suggesting that the trial is unlikely to achieve its aims even if intervention and follow-up are continued. Conversely, if the evaluation suggests a substantial effect of intervention, follow-up for at least three years is likely to be needed to adequately evaluate potential adverse effects of the intervention.

7.5 Mathematical Modeling

The compartmental model used for this protocol will be updated regularly as the trial progresses to allow investigators, stakeholders and the Data Safety and Monitoring Board to assess revised projections. Revisions to the model will include data from the baseline survey of the population cohort (PC0) on baseline characteristics, especially prevalence and ART uptake, and data from the CHiPS on testing and ART uptake.

Concurrently, a more sophisticated individual-based stochastic model of HIV transmission will be developed during the project and fitted to data from the trial, routine data and published sources to address four main objectives:

- To help interpret the results of the trial: Process data showing the extent of uptake of the intervention compared with similar data from the control arm will be used to obtain model projections of expected impact under these conditions. By examining projected impact under the conditions prevailing in Zambia and South Africa, and in different trial communities, we will be able to examine whether the level of impact and variations in impact are in accordance with expectations.

- To project longer-term impact: Modeling shows that the full impacts of UTT as well as male circumcision are not seen for several years. Impact measured during 3 years of intervention may therefore underestimate the long-term impact of the program. Models fitted to the impact seen during the first 3 years will be used to project the likely impact over longer time periods.

- To explore likely impact in different settings: If the trial demonstrates impact, it is likely that similar interventions will be implemented in a wide range of settings. The model will be used to explore how impact would be expected to vary depending on epidemiological, demographic and other characteristics of populations, and thus to project likely impact in a range of settings.

- To explore the likely impact of alternative intervention packages: Our study design will provide empirical data on the impact of the specific packages of preventive interventions incorporated in the PopART program. However, the model can be used to explore the effect of adding or removing components. For example, we can project the impact of an intervention in which male circumcision is not promoted, or where the threshold for starting ART is set at different levels.

Like all HIV models, the model will be over-parameterized compared to the amount of data available. The model will thus be fitted to baseline and follow-up data using Bayesian Monte Carlo integration methods. Priors for parameters will be determined by literature review, and a body of informed persons not including the modelers working on this study will pick the prior distributions of parameters so as to avoid bias. Comparison of priors with posteriors will be used to inform the extent to which the trial has improved our estimates of the likely efficacy of the different components of the intervention, and of other epidemiologically relevant parameters.

We acknowledge the importance of the *prevention cascade* in achieving population-level impact. Specifically, achieving high levels of uptake and effectiveness requires guiding individuals through a cascade of individual steps, starting from an initial test, through linkage to care, CD4 testing, circumcision, counseling, and ultimately antiretroviral treatment and adherence counseling for HIV-infected and eligible individuals. The contribution of different levels in the cascade, as well as their contribution to intervention cost, will be explored in our modeling and cost-effectiveness work. Our prior hypothesis based on preliminary modeling is that uptake of testing and prompt initiation of treatment will be critical.

7.6 Outcomes for Secondary Objectives

Multiple secondary objectives for this study are listed in Section 2.2. The majority of these objectives are to measure the effect of the intervention on various outcomes using standard quantitative analyses; the outcome measures for these secondary objectives are listed below and will be measured in all study arms unless otherwise noted. Those secondary objectives that are considered process measures, or that require a different sort of analysis, are described in separate sections below.

- HIV incidence over the first, second, and third years of follow-up
  - HIV diagnosis at 12 months, 24 months, and 36 months among those who were HIV-uninfected at enrollment in the *Population Cohort*
- Community viral load (subject to funding)
  - Viral load in HIV-infected members of the *Population Cohort* (approximately 75 per cluster, randomly-selected) at enrollment, 12 months, and 36 months
  - Viral load in HIV-infected members of the *Population Cohort* (all, estimated to be 300 per cluster) at 24 months
- ART adherence and viral suppression (subject to funding)

  - HIV viral load at 24 months in HIV-infected members of the *Population Cohort* who initiated HIV care and ART after commencement of the PopART intervention in the community (subject to funding)
  - HIV viral loads of health center attendees who initiated HIV care and ART after commencement of the PopART intervention in the community, drawn from routinely-collected data at health centers (if available at a given health center)
  - Self-reported adherence to ART in HIV infected members of the *Population Cohort* who initiated HIV care and ART after commencement of the PopART intervention in the community, measured at 12 months, 24 months, and 36 months
  - Loss-to-follow-up rates and missed dispensations of ARVs among health cent*er attendees who* initiated HIV care and ART after commencement of the PopART intervention in the community (and also in the control community during the same period of time), measured using routine health center data
- ARV drug resistance (subject to funding)
  - ARV drug resistance at 24 months in HIV-infected members of the *Population Cohort* who initiated HIV care and ART after commencement of the PopART intervention in the community, among individuals who are not virally suppressed at 24 months (subject to funding)
  - ARV drug resistance in HIV-infected members of the *Population Cohort* who initiated HIV care and ART after commencement of the PopART intervention in the community, measured retrospectively on samples collected at enrollment and 12 months, among individuals who are not virally suppressed at 24 months (subject to funding)
  - ARV drug resistance, measured at 12 months, 24 months, and 36 months, among participants with incident HIV infection after enrollment in the *Population Cohort* (subject to funding)

Note: Viral load/drug resistance testing will be performed at the 24 month visit, as a measure of treatment adherence, among HIV infected members of the *Population Cohort*, rather than delaying to 36 months. If the 24 month data on this indicate a significant number of participants not virally suppressed/with drug resistance, then additional funding may be sought to analyse these data again at 36 months in the *Population Cohort* and/or the *Population Cross-Sectional Survey*. Data from the 24-month visit may not be available until some time after the study ends.

- HSV-2 incidence
  - Incident HSV-2 infections at 36 months for all individuals in the *Population Cohort* who were HSV-2-uninfected at PC0.

- HIV disease progression, retention in care, and death
    - CD4 cell counts, WHO staging events, retention in care and death among *Population Cohort* participants initiating ART after commencement of the PopART intervention in the community, measured using routine health center data
    - CD4 cell counts, WHO staging events, retention in care and death among health center attendees who initiated ART after commencement of the PopART intervention in the community, measured using routine health center data
- ART toxicity
    - ART safety and clinical events among *Population Cohort* participants initiating ART after commencement of the PopART intervention in the community, measured using routine health center data
    - ART safety and clinical events among health center attendees who initiated ART after commencement of the PopART intervention in the community, measured using routine health center data
- Sexual risk behavior
    - Self-reported sexual risk behavior at Enrollment, 12 months, 24 months, and 36 months in the *Population Cohort*
    - HSV-2 incidence, listed above as a separate secondary outcome, serving as a biomarker for sexual risk behavior
- Case notification rate of tuberculosis
    - Case notification rates of bacteriologically-confirmed TB diagnosed among the general population of patients seeking care at health centers as recorded by health centers
    - TB mortality among TB cases in the community as recorded by health centers
- HIV-related stigma
    - Self-reported data on stigma indicators at enrollment, 12 months, 24 months, and 36 months in the *Population Cohort*
    - Qualitative interviews in selected members of the general population in Arms A, B, and C

7.7 Secondary Objectives for Case-Control Studies

- Carry out case-control studies to examine factors related to:
    - Uptake of HIV testing during the first round of home-based testing in Arms A and B
    - Uptake of immediate treatment in Arm A

- Uptake of HIV testing during the second round of home-based testing in Arms A and B

7.8 Secondary Objectives for Qualitative Studies

- Use qualitative and quantitative methods to:
    - Assess popular understanding of HIV testing and treatment at study initiation and during implementation
    - Evaluate the acceptability and functioning of the CHiPs in Arms A & B
    - Evaluate the acceptability of interventions and the barriers to access in Arms A & B
    - Document the effect of the intervention on social networks, stigma, sexual behavior, alcohol use, gender-based violence, HIV identity, other HIV prevention options and community morale.
    - Evaluate the process and challenges of community consultation and applying ethical principles.

7.9 Secondary Objectives Related to Economic Evaluation

Three secondary objectives of this study are concerned with economic evaluation of the intervention:

- Measure the incremental cost of the two intervention packages through systematic recording of costs in intervention and control communities.
- Estimate the effectiveness and cost-effectiveness of the intervention packages and alternative packages, both in the chosen study populations and in other populations by fitting mathematical models based on the empirical data from the trial, including data related to cost.
- Measure the burden experienced by local health centers due to implementation of the intervention in the community

Economic analysis will seek to assess the incremental health benefits of the intervention in relation to its incremental cost, and will be integrated with the modeling described above in Section 7.5. The main focus will be on costs to the health services, including equipment, materials and personnel. Incremental cost of the intervention will be estimated by comparing health services utilization and associated costs between the three study arms. We will be careful to separate out the costs of the intervention and the costs of the evaluation.

Benefits will be assessed in terms of lifetime change in quality adjusted life years (QALYs) and/or disability adjusted life years (DALYs) brought about by the interventions relative to the control arm. Health related quality of life will be measured with a generic instrument such as SF36 or SF12. Lifetime projections of health and health services utilization will be

modeled under a range of assumptions based on current epidemiological and health service evidence. Probabilistic sensitivity analysis will be used to assess uncertainty.

By combining cost data with impact estimates from the trial, we will be able to obtain direct estimates of cost per HIV infection averted. The costing data will be integrated with modeling results and quality of life information to derive cost per QALY and/or DALY using different time horizons. These data will also be used in mathematical models to explore the likely cost-effectiveness of the same intervention in different settings, and of alternative intervention packages in these or other settings.

The 3-arm trial design will allow us to compare the short and long term differences in cost-effectiveness for the two combination prevention packages based on immediate treatment or treatment according to current local guidelines. Additional data on secondary outcomes including TB incidence and other clinical events will provide improved QALY and/or DALY estimates.

The economic evaluation will mainly rely on data from the Population Cohort via a questionnaire, supplemented by health service facilities data. More specifically, we will collect data that will allow us to calculate the following benefits and costs.

We will estimate the benefits of the interventions to individuals, measured by the impact on QALYs/DALYs, and impact on work and home duty productivity (caring for children and seniors), approximated by employment status, occupation and educational status. Another benefit of the interventions is a reduced rate of HIV-related illnesses, with positive health impacts to the individual, and saved health care costs to society.

We will estimate the wider benefits of the interventions to the community. This is mainly the prevention effect of the interventions, in the form of averted secondary HIV infections. We place an economic value on those averted infections. This comprises a) the health benefit (saved QALYs/DALYs) of averted HIV and related illnesses, and b) the averted health care costs (assuming standard care) for the study communities. Further, we consider the indirect benefit to children (both infected and not infected) of mothers receiving the interventions, measured in probability of survival (we do not collect quality of life information for children).

We will generate estimates of the costs to the health care system of providing the interventions, such as clinical assessment, testing and drug provision for ART. This includes costs for treating drug resistance, toxicities, side effects and adverse events. We will consider one-time costs, for example for building and training, and apportion them to the interventions. We will also consider recurrent costs for personnel (wages and related costs, e.g. pensions), the ART drugs, other drugs, laboratory tests, materials, equipment and supplies, transport costs for staff or patients (if covered by the health care system), and overheads. We will collect aggregate data from health service facilities involved in the trial, and apportion these to individuals based on health service usage information. If we can obtain reliable data from patient records held at healthcare facilities, through some members of the *Population Cohort* and/or a small scale additional survey of a random sample of patients as they visit healthcare facilities in selected communities, we will calculate actual

costs of treatments; otherwise, we will calculate average costs for typical use of healthcare facilities. This process requires assumptions, and we will conduct sensitivity analysis to validate our estimates. Further, as the cost estimates rely on health service utilization information collected via questionnaire retrospectively for the past period, we will conduct validation to address potential recall bias.

The program may divert scarce resources from other health programs, and in order to evaluate this indirect effect, we will obtain information via qualitative interviews with senior health care management in selected communities. We will further collect information on the costs to the patients of receiving the interventions, including the costs of adverse events and drug resistance. Costs to individuals may comprise costs directly associated with treatment (frequency, duration and nature of contacts with health services, travel time), user fees, costs of tests, drug costs and other payments related to treatment, time costs (valued by lost earning opportunity) and other costs. Information on those items will be collected from the survey members.

We plan to complement the economic evaluation with additional work looking at broader outcomes, such as the impact on children of improved parental survival, and the impact of improved health status on individual productivity, participation in the labor market, poverty and wider macro-economic effects on the economy. Estimates of the benefits of improved survival and health from other studies can be integrated with the modeling work to obtain estimates of broader societal gains. If the trial shows that interventions are effective, it will also be important to model the projected cost-effectiveness of wider-scale intervention using more streamlined systems of delivery.

Outcomes for secondary objectives related to the economic analysis are summarized in Table 9.

Table 9- Summary, outcomes for secondary objectives related to the economic analysis

Secondary outcomes	Data sources	Subsample	Specific measures	Assumptions	Secondary data sources required?	When collected?
**Health care utilization and costs**	Population Cohort	All	Frequency, types and reason of visits		Yes, lifetime costs of HIV care	Each visit
	Routine patient records	HIV+ and MC patients	Diagnoses and types of treatments	Aggregation into groups with homogenous resource use	Yes, lifetime costs of HIV care from other studies	Throughout trial
	Routine facility data	Selected facilities	Health care utilization aggregated	Facilities are representative	Yes, aggregate data from Dept. of Health	One time, rolling
	Survey of facility costs	Selected facilities	One time and recurrent costs	Facilities are representative;	Yes, aggregate data from Dept. of Health	One time, rolling
**Costs of accessing care to individuals**	Population Cohort	HIV+ and MC patients	User fees, travel costs, time costs	Aggregation into groups of typical travel costs	Yes, private insurance coverage, travel costs	Each visit
**Quality of life**	Population Cohort	All	Generic measure of health	Standard assumptions of generic quality of life instruments	Yes, preference weights	Each visit
**Work and home productivity**	Population Cohort	All	Data on employment occupation	Association between occupation and wage rate	Yes, wage rates for occupations	Each visit

**Burden on care-givers**	Population Cohort	Care-givers of HIV+ and orphans	Data on caring activities	Alternative occupation	Yes, burden of care, wage rates	Each visit
**Child survival**	Population Cohort	All	Mortality information		Yes, official death records	Each visit
	Routine patient records	All	Mortality information		Yes, official death records	Throughout trial

7.10 Process Measures

Several process measures will be recorded in Arms A, B, and C to evaluate the implementation and delivery of the PopART interventions. These measures evaluate processes that are intermediary between the provision of the intervention and achievement of the primary outcome. These measures will be important therefore in understanding how and why the intervention is (or is not) successful in producing that outcome. Further, those data that are collected from CHiP teams or health centers (as opposed to research cohorts) can be reviewed by the study team during the study period and can be used to make real-time adjustments to deployment of the intervention to improve its effectiveness.

- Uptake of PMTCT
    - Self-reported use of services for PMTCT at Enrollment, 12 months, 24 months, and 36 months among HIV-infected women in the *Population Cohort* who had been pregnant in the prior 12 months
    - Uptake of PMTCT services at health centers
    - Uptake of PMTCT as indicated in data collected in households by CHiPs
- Uptake of male circumcision
    - Self-reported circumcision status/uptake at Enrollment, 12 months, 24 months, and 36 months of men in the *Population Cohort*
    - Uptake of circumcision in the community as indicated in health center data
    - Uptake of circumcision as indicated in data collected in households by CHiPs
- ART screening and uptake
    - The proportion of *Population Cohort* members, identified as HIV-infected who screen for ART eligibility, and who subsequently initiate ART
    - Proportion of community members, identified as HIV-infected in data from CHiP teams, who screen for ART eligibility, and who subsequently initiate ART, as indicated in health center data
- HIV testing and retesting
    - Self-reported recent HIV testing at Enrollment, 12 months, 24 months, and 36 months in the *Population Cohort*
    - The number of adults (16 years and older) in the household and the number of HIV tests performed as indicated in data from CHiP teams and health centers
- Time between HIV diagnosis and initiation of care
    - The proportion of *Population Cohort* members initiating HIV care within 3 months of a positive HIV diagnosis

- The proportion of community members initiating HIV care within 3 months of HIV diagnosis as indicated in data from CHiP teams (provision of HIV positive result) and health center data (date of care initiation)

7.11 Tabular Summary of Outcomes

Table 10 provides a summary of HPTN 071 objectives and outcomes including the source of the outcome data.

Table 10- Summary of Study Objectives and Related Outcomes

PC= *Population Cohort* **PX**= *Population Cross-Sectional Survey* **Ca-Co**= Case-control **CHiPs**= Community HIV-care Providers

**Objectives** and *Outcome Measures*	**Research Participants**							**Community members**	
	**PC at baseline**	**PC at 12m**	**PC at 24m**	**PC at 36m**	**PX at 36m[a]**	**Uptake Ca-Co**	**Qualitative Research**	CHiPs Data (Arm A&B)	Routine Health Center Data
Effect of the interventions on…									
**HIV incidence**									
HIV infection between 0 and 36 months among those testing HIV negative at enrollment (primary objective)	X	X	X	X					
HIV infection between 0 and 12 months in those testing HIV negative at enrollment; HIV infection between 12 and 24 months in those testing HIV negative at 12 months; HIV infection between 24 and 36 months in those testing HIV negative at 24 months (secondary objective)	X	X	X	X					
**Community viral load (subject to funding)[a]**									
Viral load in a subset of approximately 75 HIV-infected cohort/survey members per community	X	X		X	X				
Viral load in all HIV-infected cohort members			X						
**ART adherence and viral suppression**									
HIV viral load among cohort/survey members initiating ART after intervention roll-out (if funded)[a]			X						
HIV viral loads among health center attendees initiating ART after intervention roll-out (if viral load available)									X
Self-reported ART adherence among cohort/survey members initiating ART after intervention roll-out		X	X	X	X				
Loss-to-follow-up rates and missed dispensations of ARVs among health center attendees on ART									X
**Antiretroviral drug resistance (subject to funding)** *[a]*									
Drug resistance among a subset of cohort/survey members who are on ART with detectable viral load			X						
Drug resistance testing for cohort/survey members who have resistance at 24 months	X	X							
ART resistance at 12 months, 24 months, and 36 months among cohort members who acquire HIV infection during the follow-up period		X	X	X					
**HSV-2 incidence**									
Incident HSV-2 among cohort members who are HSV-2 negative at PC0 (does not include PC participants enrolled at the PC12 visit)	X			X					

**HIV disease progression, retention in care, and death**									
CD4 cell counts, WHO staging events, retention in care and death among Population Cohort participants initiating ART after commencement of the PopART intervention in the community, measured using routine health center data [b]									X
CD4 cell counts, WHO staging events, retention in care and death among health center attendees who initiated ART after commencement of the PopART intervention in the community, measured using routine health center data									X
**ART toxicity**									
ART safety and clinical events among cohort/survey members [b]	X	X	X	X	X				
ART safety and clinical events among health center attendees (based on clinic data)									X
**Sexual risk behavior**									
HSV-2 incidence (independent secondary objective above) serving as a biomarker for sexual risk behavior		X	X	X					
Self-reported sexual risk behavior	X	X	X	X	X	X[c]	X[c]		
**Case notification rate of tuberculosis**									
Case notification rates of bacteriologically-confirmed TB diagnosed among health center attendees as recorded by health centers									X
TB mortality among TB cases in the community as recorded by health centers									X
**HIV-related stigma**									
Self-reported data on stigma indicators collected from cohort members	X	X	X	X	X				
Qualitative interviews conducted with members of general population							X		
Process Measures									
**Uptake of PMTCT services**									
Self-reported use of PMTCT services at among HIV-infected cohort/survey members who were pregnant in the prior 12 months	X	X	X	X	X				
Uptake of PMTCT services at health centers									X
Self-reported uptake of PMTCT in the community								X	
**Uptake of male circumcision**									
Self-reported circumcision status/uptake among cohort/survey participants	X	X	X	X	X				
Uptake of circumcision at health centers									X
Self-reported uptake of circumcision in the community								X	
**ART Screening and uptake**									
The proportion of cohort/survey members identified as HIV-infected who screen for ART eligibility, and who subsequently initiate ART	X	X	X	X	X			X	X

The proportion of community members identified as HIV-infected who screen for ART eligibility, and who subsequently initiate ART								X	X
**Uptake of HIV testing and retesting**									
Self-reported recent HIV testing among cohort/survey	X	X	X	X	X				
Number of adults in households and the number of HIV tests performed in each community								X	X
**Time between HIV diagnosis and initiation of care**									
The proportion of cohort members initiating care within 3 months of HIV diagnosis[b]	X	X	X	X	X			X	X
The proportion of community members initiating HIV care within 3 months of HIV diagnosis								X	X
Using qualitative methods…									
**Assess popular understanding of HIV testing and treatment**							X		
**Evaluate the acceptability and functioning of the CHiPs in Arms A & B**							X		
**Evaluate the acceptability of interventions and barriers to access in Arms A & B**							X		
**Document effect of the interventions on social networks, stigma, sexual behavior, alcohol use, gender-based violence, HIV identity, other HIV prevention options & community morale**							X		
**Evaluate the process and challenges of community consultation and applying ethical principles**							X		
Through mathematical modeling and health economics methods…									
**Measure the incremental cost of the two intervention packages through systematic recording of costs in intervention and control communities** [d]			X						X
**Estimate the effectiveness and cost-effectiveness of the intervention packages and alternative packages** [d]			X						X
**Measure the burden experienced by local health centers due to implementation of the intervention in the community** [d]			X						X

[a] Not currently funded. Will be implemented if funding can be acquired

[b] Consent must be obtained to access health center records for *Population Cohort* members

[c] Explanatory research related to the outcomes/objectives indicated

[d] Cost and effectiveness objectives will be addressed through analysis of deidentified data from health centers regarding costs and clinic use, in addition to specific questions asked of the *Population Cohort*

8.0 HUMAN SUBJECTS CONSIDERATIONS

8.1 Collaborative Partnerships

At all stages of the development of this research protocol community representatives have been involved in the design and have engaged with the research team to finalize the intervention and study questions. The research teams will continue to actively engage with the study communities at various levels (for example with government structures, healthcare facilities at management and worker level, existing community forums and stakeholder groups) utilizing a range of communication and interaction strategies, as appropriate. In both countries, study committees will be formed with representation from trial staff and in-country stakeholders (from governmental to community representation) to provide guidance and feedback to the study team. As indicated in the earlier section on community engagement, partnerships and CABs worked with/established during the ZAMSTAR study will be reviewed for this study to ensure that all community groups and interests are represented.

8.2 Social Value

As described in Section 1, the worldwide burden of HIV infection continues to grow, with populations in sub-Saharan Africa particularly afflicted with high rates of HIV prevalence and incidence. If this study is able to show that providing a combination prevention intervention including universal testing and treatment is effective in slowing the spread of HIV in communities and is cost-effective, it could provide a path forward to lowering the burden of HIV in sub-Saharan Africa and throughout the world and, importantly, in the communities and countries in which the study will be performed. The value of a highly effective prevention intervention for the economies of countries, communities and persons, the reduction in morbidity and mortality, the value to health infrastructure and even political stability could potentially be large. The multifaceted approach of the intervention, incorporating strengthening and promotion of PMTCT, male circumcision, and universal testing and treatment, offers value to community members whether they are men or women, already infected with HIV or uninfected. The health systems strengthening component of the study, which will be implemented in all three study arms, promises to offer value to all of the communities in the study, even if the intervention cannot be shown to lower HIV incidence.

8.3 Scientific Validity

This study will provide evidence to either support or refute the mathematical model discussed earlier, which has indicated that if a high proportion of the population can be tested, with those found HIV-infected offered immediate ART, HIV infection may be reduced substantially within two years, and potentially eliminated as a public health problem in the longer term. The study has been powered to determine the impact of the interventions on the primary and secondary endpoints. The multi-community cluster-randomized study design chosen for this study has, we believe, the best chance of

providing an answer to the research question: "Can universal HIV testing and intensified provision of HIV treatment and care reduce population-level HIV incidence?"

The study will be conducted according to the most rigorous standards of research and is therefore expected to give definitive answers about the process of implementing the intervention as well as the impact of such an intervention at community level. The study results will be shared throughout the study with national and international policy-makers to ensure that the findings are understood and that lessons from the study are implemented.

8.4 Fair Subject Selection

This study will be carried out in areas of Zambia and South Africa that are known to have a high prevalence and incidence of HIV infection. These areas are continuing to experience severe generalized HIV epidemics with prevalence levels of 15-20% in many areas. Most of the communities chosen for this project are communities that have already been involved in similar community-based research projects such as ZAMSTAR (Zambia and South African TB and AIDS Reduction Study). There are both advantages and disadvantages to involving 'research experienced' communities in a new research project such as this. Communities can theoretically become over researched and placed at risk for "research burn-out"- with community members reluctant to become involved in additional research and becoming disillusioned with research related burdens (e.g. time spent, intervention risks, risks to privacy etc.). On the other hand, communities that are already accustomed to and well informed about research processes can be considered to be in a more empowered position to engage in a research initiative such as HPTN 071, than "research naïve" communities. Community leaders will be accustomed to engaging with research teams and structures such as community advisory boards (CABs) will already be established and functioning.

The research teams in both Zambia and SA have discussed this issue with the respective government authorities and a joint decision was taken favoring existing research sites and communities over new ones for this very reason. Formative research will be conducted prior to the start of the study to document existing community engagement structures in each community and their level of functioning with additional action taken to improve the functioning of these structures, where necessary.

Care will be taken to ensure that community related research risks and burdens are minimized and that community benefits are maximized (See Section 8.5).

All population members in the intervention communities will be encouraged to receive home-based HIV counseling and testing and to receive the health education, symptom screening and referrals that are included in the intervention. The health information of adult household members who provide consent (and information of minors for whom consent has been obtained from parents or guardians) will be recorded in the CHiPs database to allow follow up on referrals and linkage to care by CHiPs. The CHiPs intervention will be offered throughout the community, including to women and minors, hard-to-reach populations and "high HIV-risk groups" because this study is directly relevant to the health needs of these groups. In communities where the immediate ART is

offered outside of local guidelines, those younger than 18 will not be eligible , in accordance with local regulations regarding the age of consent for research. However, to the degree that local guidelines do allow minors to initiate immediate ART, this will be implemented.

8.5 Risk-Benefit Assessment

This community-based, cluster-randomized study can potentially incur risk of harm at both a community and an individual level. Likewise study-related benefits may accrue at both an individual and a community level.

8.5.1 Community Level

8.5.1.1 Benefits

At the community level, mathematical modeling suggests that the PopART intervention may result in a substantial reduction in HIV incidence and, if sustained over time, to the eventual elimination of HIV as a public health problem, with a wide range of health and socio-economic benefits. Child morbidity and mortality should also be significantly decreased through both the direct effects of the intervention on mother-to-child transmission of HIV and the protection of the health of HIV-infected mothers. In addition to intervention effects on HIV transmission, the universal testing and counseling program is designed to promote acknowledgement and acceptance of HIV as a community-wide health problem potentially resulting in lessening of HIV-related stigma and discrimination. While the control communities will not benefit directly from the intervention, at least until such time as immediate eligibility for ART is offered in Arm C communities, the project will ensure that a standard of care is provided in these communities. In addition, if the trial shows that the intervention is highly effective and cost-effective, leading to wider scale roll-out of the program, efforts will be made to ensure that the control communities are among the first to benefit from this wider implementation.

Networks of stakeholders that will be created through the implementation of the study interventions will not only improve communication between community groups but will also be a catalyst for reinvigorating social connections that have been threatened in the wake of poverty and HIV/AIDS. Previous experience with the ZAMSTAR study suggests that these networks can be useful for advocating for research and improving research literacy.

8.5.1.2 Risks

Any community-based research project may present risks to a community. Communities may feel disempowered by having a research agenda imposed on them or they may be placed at risk of stigmatization by the publication or dissemination of research results. Large community research projects may disrupt intra-community social structures and networks that are not always easily understood by an external research team.
For this particular project at community level, there is the risk of behavioral disinhibition if the wide-scale provision of testing, treatment and male circumcision are assumed to reduce risk and thus encourage unsafe sexual behavior. Extensive counseling at an

individual level and HIV prevention education at a broader community level will attempt to minimize this risk, which unfortunately is known to be potentially present with most HIV prevention studies. We will also seek to measure such harms through collection of process data as well as specific sub-studies as outlined in the Research Plan.

An additional potential community related risk involves the possible burden that could be placed on existing health services. Existing health services are already over-burdened due to inadequate resources and overwhelming disease rates. Health-care workers may be recruited into study teams and leave their current positions, worsening the problem.

8.5.1.3 Minimizing Risks to Communities

Communities will not be named in any publication or dissemination of results of the study. Well-functioning community engagement structures (such as CABs) will help to mitigate risks at community level by advising the study team and representing the views of the communities. In addition the research team will actively solicit and report any instances of perceived social harm. We will aim to recruit CHiPs that are residents of their respective study communities and many of the field study staff will have either previously lived or worked in the communities which will give them an advantage in terms of relating with participants and other stakeholders.

Health service burden will be minimized by leveraging additional funding to the Ministry/Department of Health to enable additional staff to be trained and recruited. These staff will work for the duration of the study and it is likely they will continue on as Ministry/Department of Health employees at the end of the study due to natural attrition and increased demand (many services are currently understaffed in respect to the stated staffing establishment). Study teams will work closely with government agencies and will not entice staff away from them by offering differential salary packages.

Communication will be maintained with study communities for the duration of the study and a well-developed exit strategy will be planned with the input of all stakeholders and community engagement structures to ensure that there is a seamless transition from study to routine health services at the end of the study

8.5.1.4 Risk-Benefit Assessment at Community Level

The mathematical models suggest, and we believe, that overall benefits from the proposed intervention program would greatly outweigh any risks or harms at community level. Nevertheless, we acknowledge that some communities and individuals may be placed at increased risk as a result of the intervention. It is therefore important to put appropriate measures in place, as described above, to mitigate these possible harms.

8.5.2 Individual Level

8.5.2.1 Benefits

There is a wide range of benefits at individual level. Knowledge of personal HIV status provides a portal to treatment and care services for HIV-infected patients while HIV-

uninfected individuals can be supported in adopting preventive measures. Early treatment of HIV-infected patients has been shown to be of substantial clinical benefit [12], and the treatment provided (including antibiotic prophylactic therapy) can be confidently expected to reduce the incidence of TB and other infectious diseases as well as protecting the immune system and significantly slowing HIV clinical progression [89, 105-107]. It will also significantly reduce the risk of onward transmission to sexual partners, with a consequent decrease in the anxiety and psychological distress associated with HIV infection. WHO has also recently endorsed this approach in a programmatic update[108]

8.5.2.2 Risks

A key component of the intervention package is an annual HIV testing campaign that aims to encourage all adult community members to undergo HIV testing and counseling. Such approaches involve some risks. These include possible social harm, stigmatization or intimate-partner violence related to intended or unintended disclosure of HIV status, either within or beyond the household; and psychological trauma from learning one's HIV test status.

Community members who report to the health center to undertake HIV care (including ART) or to receive HIV testing will have sensitive data collected in clinic records, some of which will be then be harvested into an electronic research database. Most of these research data will be collected without personal identifiers, but research cohort members may provide consent for use of identified data. Collection and storage of sensitive health information carries with it the risk of unwanted disclosure if there is a breach of data security or incomplete removal of personal identifiers from “anonymous” data sets.

Men who test negative for HIV infection will be offered circumcision through a locally provided service. Circumcision will also be provided to HIV-infected men who request it. While data from randomized trials and routine male circumcision programs in sub-Saharan Africa have shown very low levels of adverse effects, there are some risks of the surgical procedure, including pain, bleeding and infection [50, 109, 110]. There is also a risk of enhanced HIV transmission if men resume sexual activity before the circumcision wound is fully healed [111, 112].

At the time of preparation of version 3.0 of this protocol, the offer of immediate ART goes beyond current national and international guidelines for HIV care, although these treatment guidelines are expected to change rapidly [12]. Immediate initiation of ART presents some potential risks, but data from the START trial has identified that overall, immediate ART initiation confers significant clinical benefit over deferral. The risks of starting ART for any HIV infected individual includes side effects, the development of drug resistance if treatment adherence is sub-optimal and consequent limitation of future treatment options, and the inconvenience of having to attend the study clinic and starting on a lifelong course of treatment when still asymptomatic.

There are additional minor risks for the research cohort participants including the taking of specimens, which may include pain or bruising when blood samples are taken. Also, there is the risk that some questions addressed to participants, for example relating to their sexual behavior or HIV infection status may result in discomfort or distress.

The main risks associated with the evaluation research for those individuals who are not directly exposed to the ART intervention are similar to risks relevant to any population-based epidemiological HIV research and primarily involve risks to privacy and confidentiality. These risks are discussed below as well as in Section 8.8.

8.5.2.3 Minimizing Risks to Individuals

To minimize social and other harms relating to the intensive testing program, staff delivering the program will be carefully trained and supervised to ensure that they have the required skills to provide individual or couple counseling according to national and international guidelines. In particular, although couples will be encouraged to undergo testing and counseling together, individual testing will be provided for those not wishing to take this up. Participants will be given information about community-based organizations providing support and guidance for those dealing with the psychological consequences of HIV infection or suffering from domestic violence. Follow-up counseling will be offered by the community counseling teams as is required by individuals or households according to their wishes.

Data systems and data handling procedures for capturing, transferring, analyzing and storing electronic data obtained from health centers, *Population Cohort* participants and community members contacted by CHiPs will be developed and tested to verify their ability to preserve participant confidentiality. Electronic systems in which these data are kept will be password protected with access limited to authorized staff. Personal identifiers (name, address, plot number, telephone number, GPS coordinates) collected by study staff on mobile devices are stored encrypted. The devices are programmed to time out after a period of disuse and then require re-entry of username and password to log in. Decrypted electronic personal identifiers will only be generated to support specific field operations (e.g. male campaigns) and will be stored temporarily in separate datasets with password protection, accessible only to designated staff (for computers and servers).

Phylogenetic studies are proposed (pending funding) that would be carried out using stored samples to evaluate the phylogenetic relationship of viruses in the community. These studies are described in the *Phylogenetics Ancillary Protocol*. Any such analyses would only be carried out after removing linkage to personal identifiers. We will ensure that data provided to the team performing any such analyses, and any reports of such analyses, cannot inadvertently identify specific individuals or transmission events. A detailed description of human subjects protections specifically related to the phylogenetics study can be found in the *Phylogenetics Ancillary Protocol*.

Male circumcision will be provided by existing service providers through the routine health service and therefore all staff will be carefully trained in line with local guidelines to ensure that the operation is carried out safely, with minimal risk of adverse events. Patients will be seen for a follow-up visit after circumcision according to local guidelines. Staff will be trained to deliver effective counseling about the importance of abstaining from sexual activity until the wound is fully healed and also to explain carefully that the operation is only partially protective against HIV infection and to warn against the hazard of risk disinhibition.

As noted above, the only component of the intervention that goes beyond current guidelines is the offer of immediate ART regardless of CD4 cell count. The team plans to inform all patients seeking ART at health centers in PopART communities where immediate ART is offered of the differences between current and previous treatment guidelines and the strategy being offered by the study in their community, including known risks and benefits. Individuals must provide written informed consent for initiation of ART if not eligible according to current local guidelines. Information provided to patients will have been developed partly in consultation with CABs and have been piloted and translated into vernacular text. The treatment regimen has been chosen carefully to be convenient to take, to be appropriate for the widest possible range of patients, and to minimize the risk of toxicity or side effects. No additional adverse events are expected in patients with intact immune systems. The most significant risks are associated with poor adherence to treatment. To minimize this, community health workers will support patients on treatment, making regular household visits to check on treatment adherence and in particular checking up on patients when they do not attend routine clinic visits. Toxicity associated with antibiotic prophylaxis will also be monitored and treatment modified if necessary.

All project staff will undergo training in GCP and human research protections in accordance with the U.S. National Institutes of Health (NIH) requirements. There will be a strong emphasis in staff training and supervision on the importance of strict confidentiality of participant information as well as on supportive interviewing skills. Blood collection will be carried out by fully trained staff using appropriate sterile procedures.

8.5.2.4 Risk- Benefit Assessment at Individual level

There are risks associated with this study for the individuals involved. However as indicated above, most of these risks are no more than those encountered in everyday life. Individuals exposed to the early ART intervention are likely to encounter a greater than minimal level of risk and some of these risks or burdens may as yet be poorly quantified (e.g. risks associated with extended ARV exposure). However, immediate ART for all individuals with HIV infection irrespective of CD4 count has now been shown to confer clinical benefit on those individuals [12]. All patients receiving ART at the health centers in all study arms will be monitored for reactions to their ART regimen, in accordance with the local standard of care. Thus we believe that the overall risk-benefit assessment for this study is favorable at both a community and an individual level.

8.6 Informed Consent

In a community-based, cluster-randomized trial such as this one, informed consent needs to take place at several levels ranging from **consent from the government authorities**, to so-called "community consent", and finally to individual consent. However obtaining individual consent from every individual living in every community involved in this study would be unfeasible. As discussed earlier in Section 3.2 the CHiP teams, while an integral part of this research will deliver a community health care package that is recognized as good practice and as such is not a research intervention. Much of the **routine healthcare surveillance data** collected as part of this study, particularly from the control

communities will be made available to the research team by the respective public health authorities (who are in full support of the project) and collected without specific individual informed consent. This information will be collected, coded, stored and managed in such a way as to ensure individual identity and privacy are protected at all times.

8.6.1 Approval from Respective Authorities

Approval for this project has been obtained from the respective healthcare authorities in both South Africa and Zambia. Additionally during the planning process of the study approval will be sought from other authorities such as district or local councilors, political leaders and traditional leaders.

8.6.2 'Community' Consent

It is of the nature of a cluster-randomized trial of this kind that entire communities are assigned to one study arm or the other, and individual consent for community allocation is not possible. The term 'community consent' can be misleading. True 'Community Consent' is only possible if the "community has a legitimate political authority, e.g. a tribal council that has the authority to make binding decisions on behalf of its members." [113]. If used inappropriately, the concept of 'community consent' may result in a false sense of security or mandate. We will seek consent for community participation from community-level stakeholders who will be defined through the community engagement process (see Research Plan) and who will include local leaders. Following agreement to participate, community representatives will take part in a public randomization ceremony at which the allocation of communities to study arms will be decided using a transparent and fair process.

8.6.3 Individual Consent

Individual consent for CHiP team activities
Because the proposed CHiP team activities are poised between an established public health intervention (home–based testing and outreach) and a public health research project (data collection and additional follow-up), the team will seek from the appropriate ethics committees, an alteration of consent (verbal consent) for participation in the community intervention. Verbal consent will also permit data collected by CHiPs to be used in aggregate form for research purposes. This verbal consent will be accompanied by a written information leaflet that will be provided to all households. This information leaflet will contain information about the project as a whole, as well as appropriate local research team contact details, and will also describe the option of each household not to engage with the CHiP teams or receive any additional visits.

There is adequate prior research to show that household delivery of HIV testing, and linkage to services by community health workers is safe and effective, such that its deployment in this study could be considered a public health intervention, and therefore not requiring written research consent from each of the approximately 800,000 people to be reached by the CHiPs workers. However there are aspects of the CHiPs intervention that are innovative, and go beyond what would be considered an extension of government health services. For example, it is not routine to have CHiP-performed HIV test results

entered into a database which is also populated by health center data, and then have those data prompt CHiPs to return to households to follow up with HIV diagnosed participants who have not reported to the health center, in order to provide linkage to care (e.g. early ART and circumcision). These aspects will thus be included in the initial verbal consent process.

In summary, an alteration of consent (verbal) will be requested based on the following considerations which comply with the U.S. Code of Federal Regulations (CFR) *45 CFR 46.116 (d)*:

(i) As already described CHiP activities involve delivering community based health care (rather than primarily research activities) and involve minimal risk.
(ii) Requiring full written research consent from all individuals that come into contact with the CHiP teams would make this project logistically unfeasible
(iii) Rights and welfare of individuals will not be adversely affected by a verbal consent process that will be documented by the CHiP teams
(iv) Pertinent written information about the project will be provided to all households visited.

Individual written consent for HIV testing and other interventions such as male circumcision will be obtained using standard procedures as these interventions are considered part of the routine delivery of HIV prevention services and not specifically study related. Thus individuals who undergo these procedures in the study communities will not specifically be asked to participate in a research study, but rather will be asked to consent to these activities as part of their health care.

Individual consent for ART outside of local guidelines
The main aspect of the intervention that goes beyond current guidelines is the offer of immediate commencement of ART regardless of CD4 cell count or clinical stage. As described above, the study team will obtain consent for research from patients in any arm who are offered immediate treatment that is not considered standard of care according to prevailing local treatment guidelines. Any patients declining this offer will be provided with follow-up and treatment in the same health facilities according to current standard of care. Participants consenting to commencement of ART regardless of CD4 or clinical stage will continue to be asked to consent (verbally) to any CHiP team activities and related data collection, as described above. Once local implementation of immediate ART is in place, individual consent will not longer be necessary.

Individual consent for research studies in all Arms
Written informed consent to participate in research will be required before enrolling individuals in the *Population Cohort and Case-Control* studies. Written informed consent will also be required of individuals participating in qualitative research activities that involve collection of participant-identified responses to interviewer questions (such as interviews and focus groups). However written consent will not be sought for other types of qualitative methods, such as observation of persons who are not participants.

The study team has considerable experience of designing and implementing suitable models of informed consent for study populations in resource-poor settings in Zambia and South Africa that may have low levels of functional literacy. Care will be taken to ensure that information materials are developed that are appropriate to the study population, with translation into a local language where necessary and back-translation into English to ensure accuracy. Project staff will go through the information sheets with participants and questions will be asked to check their understanding of key points before signed consent is sought. Illiterate participants will be asked to give fingerprint consent witnessed by a literate individual who will sign that the individual has been given sufficient information to allow for an informed decision and has given their full consent voluntarily.

8.6.4 Waiver of Individual Consent to Access CHiP and Routine Clinic Data

A waiver of individual consent will be requested to access and link CHiP and routine clinic data. The linked data will be used to monitor and facilitate linkage of CHiPs clients to care at health clinics, and so its primary use is to provide benefit to clients. Data so collected will also be used for research purposes in coded form. This request is justified by the following considerations which comply with *45 CFR 46.116 (d)*:

(i) The research involves no more than minimal risk to participants as the data will be de-identified and presented in aggregate form to the research teams that will be analyzing the data for research purposes.
(ii) The rights and welfare of research participants will not be adversely affected in any way by the collection of these data, which will be stored confidentially by the CHiP team members and shared only in de-identified form with the research teams
(iii) The research could not practicably be carried out without the waiver, as attempting to obtain written informed consent from all community members involved in a study of this scale would not be feasible.
(iv) Household members will be provided with pertinent data about the project and the need to gather and report on the information gathered by the CHiP teams as well as certain routine clinic data.

In the CHiPs intervention, health data from consenting individuals will be captured in association with personally-identifying information (such as name, age, and gender) and will be assigned identification numbers unique to the household and to the individual from whom it is obtained. During follow up, CHiP teams will have access to the personally-identified data for the clients in their CHiP zone because they need this information to find individuals and provide individualized care. Data captured by CHiPs in the field, and any client-linked data retrieved from clinics, will only be shared for *research* use after personally identifying information has been removed. Besides the CHiPs (whose access to personally-identified data is limited to clients in their zone) only the study data manager will have access to personally-identified client data, which will be stored in an encrypted

database. The encryption method and encryption key for this database are embedded in the software and can only be accessed by designated data managers.

8.7 Independent Ethical Review

Approval to conduct this study will be obtained from the following IRBs/ECs. In instances where there is disagreement or discordant IRB requirements the condition providing the highest level of human subject protection will be implemented. Approval must be obtained from the local, and national (where relevant) IRBs before the study can be initiated.

Ethical clearance for the trial will be sought from Institutional Review Boards (IRBs) in the United Kingdom (UK), Zambia and South Africa. Adverse events will be reported on a regular basis according to the individual requirements of these IRBs.

8.8 Respect for Participants and Communities During and After the Study

8.8.1 Confidentiality

Strict measures will be in place to safeguard confidentiality of data. All laboratory specimens, reports, study data collection, process, and administrative forms will be identified by coded numbers only to maintain participant confidentiality.
Personal identifiers (name, address, global positioning system coordinates) will only be collected for (1) informed consent and (2) operational and logistical purposes (i.e. to ensure tracing of participants by intervention staff and to locate cohort participants for follow-up visits). Personal identifiers will appear on paper or electronically on appointment books, consent forms, log books, follow up lists and other listings. These listings will NOT include any (sensitive) study information (including laboratory data). A unique study number will be used to link personal identifiers to study information.

Personal identifiers on paper will be stored in a locked cabinet. Electronically kept personal identifiers will be stored in separate datasets with password protection only accessible for designated staff (for computers and servers). Hand-held devices will also be password protected and personal identifiers will be stored in an encrypted format.

Participants' study information will not be released without the written permission of the participant, except as necessary for monitoring by the National Institute of Allergy and Infectious Diseases (NIAID) and/or its contractors, representatives of the HPTN CORE, SDMC, and/or LC, other government and regulatory authorities, and/or site IRBs/ECs. Datasets transferred to locations outside the study sites (e.g. for analyses, progress reports) will be stripped of any personal identifier before transfer.

All electronic data will be stored in password protected database systems. Read and write authorization of data will depend on the designation of the staff member. A second layer of protection is hardware password protection on computers, servers and networks. Thirdly data transfer over wireless or mobile networks will use Virtual Private Networks or router protected dedicated internet protocol addresses.

All collected study data on central computers and servers, remote computers and hand-held devices, will be backed up daily. Backup tapes/discs will be stored separately from the primary electronic storage.

8.8.2 Data and Safety Monitoring Plan

An independent data safety monitoring board (DSMB) will be established according to accepted international norms. The membership of the committee will include expertise in HIV prevention, statistics, cluster-randomized trials and clinical medicine (including antiretroviral therapy). The responsibilities of the DSMB will be to monitor data from the trial and to advise the sponsor and study leadership on any recommended changes to the conduct of the study including early termination for futility on the primary endpoint if appropriate. A formal interim analysis is not anticipated as it is important to measure the effect of the intervention over the full three year follow-up period. However, data from the study communities on operational performance including uptake, retention and adverse events will be reported to the DSMB and reviewed on an ongoing basis.

Data on the uptake of trial interventions - in particular HIV testing and treatment, retention in HIV care and medical male circumcision - will be captured electronically in all trial communities, facilitating timely analysis. We will monitor intervention uptake on a monthly basis. We will use these data for the trial comparison of Arm A (immediate treatment) vs. Arm C (standard-of-care). If study power falls below a pre-specified threshold, then the DSMB will consider whether the trial should be stopped for futility. This pre-specified threshold will be defined in consultation with the DSMB prior to the start of trial interventions.

8.8.3 Communicable Disease Reporting Requirements

Study staff will comply with national requirements to notify tuberculosis identified among study participants to local health authorities. Participants will be made aware of all reporting requirements during the study informed consent process. HIV is not a notifiable disease in either country.

8.8.4 Post-Trial Management of Participants Exposed to the Early ARV Intervention

Any individual started on ART during the trial will continue this therapy after the trial since there is no current guidance to stop ART once it has been started. This treatment will be provided through the national health systems and this has been discussed and is understood by all HIV care implementing agencies in the study communities.

8.8.5 Study Discontinuation

The study also may be discontinued at any time by NIAID, the HPTN, other government or regulatory authorities, and/or site IRBs/ECs.

9.0 LABORATORY SPECIMENS AND BIOHAZARD CONTAINMENT

9.1 Local Laboratory Specimens

"Local Laboratory" in this study refers to regional laboratories and centralized laboratories in each country. Laboratory testing will be performed using stored samples to meet study objectives. The results of testing performed using stored samples will not be returned to study sites or participants. The HPTN LC will determine the location of testing. Tests performed by Local Laboratories are described in more detail in Appendix I and the SSP Manual. Local Laboratories performing these tests will receive External Quality Assurance (EQA) panels for HIV and HSV-2 testing from the HPTN LC.

Each study site and Local Laboratory will adhere to standards of Good Clinical Laboratory Practice (GCLP), the laboratory SSP Manual, and all activities related to processing, labeling, testing, storage, transport and shipping (to centralized laboratories or to the HPTN LC). Specimen collection and storage at selected Local Laboratories will be documented using the HPTN Laboratory Data Management System (LDMS), as described in the SSP Manual.

All specimens will be shipped in accordance with local shipping regulations as well as International Air Transport Association (IATA) specimen shipping regulations. The HPTN LC will determine which shipments will be documented using the HPTN LDMS, as described in the SSP Manual.

As described in Section 5, the following types of specimens will be collected for testing at the Local Laboratory:

***Population Cohort*:**

- Blood specimens for the following:
    - HIV testing
    - HSV-2 testing
    - Plasma storage

9.2 HPTN Laboratory Center (LC) Specimens

Stored samples will be used for retrospective, centralized testing at the HPTN LC. This will include HIV and HSV-2 testing (e.g., to confirm results obtained in country, determine HIV infection status, and for quality assurance (QA), including confirmation of HIV and HSV-2 seroconversion events). Results will not be returned to study sites or participants, unless directed by the HPTN LC for specific cases. If funded, viral load testing and antiretroviral drug resistance testing will also be performed. Other testing may include: cross-sectional HIV incidence testing and testing for antiretroviral drugs and other substances (e.g., other medications, substances of abuse). Selected samples may also be tested to characterize the HIV virus (e.g., HIV subtyping, HIV tropism) and the host response to HIV infection. In some cases, testing may be performed at a commercial laboratory or other laboratory designated by the HPTN LC. If the *Phylogenetics Ancillary*

Study is funded, samples will also be used for that work (see Section 9.4.1). Results from the *Phylogenetics Ancillary Study* will not be returned to study sites or participants.

The study sites will ship samples to the HPTN LC on a routine basis and will ship additional samples as requested by the HPTN LC. Additional information will be provided in the SSP Manual.

It is important to note that the volume of plasma stored at each study visit will be limited, due to the very large number of participants in the study. In some cases, testing will be performed at the HPTN LC (rather than at the Local Laboratories) so that specialized methods can be used that require lower plasma volumes, and so that derivatives generated during testing (e.g., plasma supernatant, HIV RNA, polymerase chain reaction amplicons) can be saved and used for other types of testing. This will increase the likelihood that there will be sufficient stored plasma for all of the planned assessments.

9.3 Quality Control and Quality Assurance Procedures

HPTN LC staff will conduct periodic visits to each site to assess the implementation of on-site laboratory quality control (QC) procedures, including proper processing, labeling, storage, proper maintenance of laboratory testing equipment and use of appropriate reagents. HPTN LC staff will follow up directly with site staff to resolve any QC or QA problems identified through proficiency testing and/or on-site assessments.

Throughout the course of the study, the HPTN LC will work with HPTN SDMC to select a random sample of stored specimens to test for QA purposes. HPTN LC staff will follow-up directly with site staff to resolve any QA problems identified through this process.

9.4 Specimen Storage and Possible Future Research Testing

Study site staff will store plasma collected in this study until the HPTN LC confirms that all protocol testing has been completed. Note that some protocol testing will be performed retrospectively, after the last participant completes the final study visit. Protocol testing will include QC testing and other testing performed at or coordinated by the HPTN LC (see Section 9.2).

In addition to protocol testing (see Sections 9.1 and 9.2), study participants will be asked to provide written informed consent for their specimens to be stored for possible additional, future testing (long-term storage), unless disallowed by local laws or regulations. The specimens of participants who do not consent to long-term storage for future research will be destroyed after the HPTN LC confirms that all protocol-related testing has been completed.

9.4.1 Proposed Phylogenetics Study

If the *Phylogenetics Ancillary Study* is funded, some stored plasma specimens from the *Population Cohort* will be transferred to other laboratories for HIV sequencing and analysis. Samples from the *Population Cohort* will only be made available for the phylogenetics study after all of the primary assessments for the main HPTN 071 study

have been completed (including quality assurance testing, HIV and HSV-2 testing, confirmation of HIV seroconversion events) and after the HPTN LC has determined that sufficient plasma would remain for any secondary assessments (e.g., , viral load testing, resistance testing). Samples from the *Population Cohort* will only be made available for the *Phylogenetics Ancillary Study* if the participant consented to be included in that study.

9.5 Biohazard Containment

As the transmission of HIV and other blood-borne pathogens can occur through contact with contaminated needles, blood, and blood products, appropriate blood and secretion precautions will be employed by all personnel in the drawing of blood and shipping and handling of all specimens for this study, as currently recommended by the U.S. Centers for Disease Control and Prevention (CDC). All infectious specimens will be transported in accordance with U.S. regulations [42 Code of Federal Regulations (CFR) 72].

10.0 ADMINISTRATIVE PROCEDURES

10.1 Protocol Registration

Prior to implementation of this protocol, and any subsequent full version amendments, each site must have the protocol and the protocol consent form(s) approved, as appropriate, by their local IRB/EC and any other applicable regulatory entity (RE). Upon receiving final approval, sites will submit all required protocol registration documents to the DAIDS Protocol Registration Office (DAIDS PRO) at the Regulatory Services Center (RSC). The DAIDS PRO will review the submitted protocol registration packet to ensure that all of the required documents have been received.

Site-specific informed consent forms (ICFs) WILL be reviewed and approved by the DAIDS PRO and sites will receive an Initial Registration Notification from the DAIDS PRO that indicates successful completion of the protocol registration process. A copy of the Initial Registration Notification should be retained in the site's regulatory files.

Upon receiving final IRB/EC and any other applicable RE approval(s) for an amendment, sites should implement the amendment immediately. Sites are required to submit an amendment registration packet to the DAIDS PRO at the RSC. The DAIDS PRO will review the submitted protocol registration packet to ensure that all the required documents have been received. Site-specific ICF(s) WILL NOT be reviewed and approved by the DAIDS PRO and sites will receive an Amendment Registration Notification when the DAIDS PRO receives a complete registration packet. A copy of the Amendment Registration Notification should be retained in the site's regulatory files.

For additional information on the protocol registration process and specific documents required for initial and amendment registrations, refer to the current version of the DAIDS Protocol Registration Manual.

10.2 Study Activation

Pending successful protocol registration and submission of all required documents (see Section 10.1 above); CORE staff will "activate" the site to begin study operations. Study implementation may not be initiated until a study activation notice is provided to the site.

10.3 Study Coordination

Study implementation will be directed by this protocol as well as the SSP Manual. The SSP Manual — which will contain reference copies of the *Requirements for Source Documentation in DAIDS Funded and/or Sponsored Clinical Trials,* as well as the DAIDS Manual for Expedited Reporting of Adverse Events to DAIDS, Version 2.0, dated January 2010 and the DAIDS Toxicity Tables — will outline procedures for conducting study visits; data and forms processing; AE assessment, management and reporting; dispensing study products and documenting product accountability; and other study operations.

Study case report forms, electronic data capture tools, and other study instruments will be developed by the protocol team and HPTN SDMC. The study data from all sources ultimately will be transferred to the HPTN SDMC for storage and analysis. Quality control reports and queries will be generated and distributed to the study sites on a routine schedule for verification and resolution.

Close coordination between protocol team members will be necessary to track study progress, respond to queries about proper study implementation, and address other issues in a timely manner. Rates of accrual, adherence, follow-up, and AE incidence will be monitored closely by the team as well as the HPTN Study Monitoring Committee. The Protocol Chair, DAIDS Medical Officer, Protocol Biostatistician, SDMC Project Manager, and CORE Protocol Specialist will address issues related to study eligibility and AE management and reporting as needed to assure consistent case management, documentation, and information-sharing across sites.

10.4 Study Monitoring

On-site study monitoring will be performed in accordance with DAIDS policies. Study monitors will visit the site to

- Verify compliance with human subjects and other research regulations and guidelines;

- Assess adherence to the study protocol, study-specific procedures manual, and local counseling practices; and

- Confirm the quality and accuracy of information collected at the study site and entered into the study database.

Site investigators will allow study monitors to inspect study facilities and documentation (e.g., informed consent forms, health center and laboratory records, other source documents, case report forms), as well as observe the performance of study procedures.

Investigators also will allow inspection of all study-related documentation by authorized representatives of the HPTN CORE, SDMC, LC, NIAID,US and in-country government and regulatory authorities and IRBs/ECs. A site visit log will be maintained at the study site to document all visits.

10.5 Protocol Compliance

The study will be conducted in full compliance with the protocol. The protocol will not be amended without prior written approval by the Protocol Chair and NIAID Medical Officer. All protocol amendments must be submitted to and approved by the relevant IRB(s)/EC(s) and the DAIDS Regulatory Support Center (RSC) prior to implementing the amendment.

10.6 Investigator's Records

The study site investigator will maintain, and store in a secure manner, complete, accurate and current study records throughout the study. The investigator will retain all study records for at least three years after submission of the CTU's final Financial Status Report to DAIDS, which is due within 90 days after the end of the CTU's cooperative agreement with DAIDS, unless otherwise specified by DAIDS or the HPTN CORE. Study records include administrative documentation — including protocol registration documents and all reports and correspondence relating to the study — as well as documentation related to each participant screened for and/or enrolled in the study — including informed consent forms, locator forms, case report forms, notations of all contacts with the participant, and all other source documents.

10.7 Use of Information and Publications

Publication of the results of this study will be governed by the HPTN Manual of Operations and policies. Any presentation, abstract, or manuscript will be submitted by the Investigator to the HPTN Manuscript Review Committee for review prior to submission.

11.0 REFERENCES

1. UNAIDS, *Report on the Global AIDS Epidemic.* 2010.
2. Beck, E.J., X.M. Santas, and P.R. Delay, *Why and how to monitor the cost and evaluate the cost-effectiveness of HIV services in countries.* AIDS, 2008. **22 Suppl 1**: p. S75-85.
3. De Cock, K.M., et al., *Can antiretroviral therapy eliminate HIV transmission?* Lancet, 2009. **373**(9657): p. 7-9.
4. Hankins, C.A. and B.O. de Zalduondo, *Combination prevention: a deeper understanding of effective HIV prevention.* AIDS, 2010. **24 Suppl 4**: p. S70-80.
5. Pirrone, V., et al., *Combinatorial approaches to the prevention and treatment of HIV-1 infection.* Antimicrob Agents Chemother, 2011. **55**(5): p. 1831-42.
6. Templeton, D.J., *Male circumcision to reduce sexual transmission of HIV.* Curr Opin HIV AIDS, 2010. **5**(4): p. 344-9.
7. Volmink, J., et al., *Antiretrovirals for reducing the risk of mother-to-child transmission of HIV infection.* Cochrane Database Syst Rev, 2007(1): p. CD003510.
8. Johri, M. and D. Ako-Arrey, *The cost-effectiveness of preventing mother-to-child transmission of HIV in low- and middle-income countries: systematic review.* Cost Eff Resour Alloc, 2011. **9**: p. 3.
9. Buchbinder, S.P. and A. Liu, *Pre-exposure prophylaxis and the promise of combination prevention approaches.* AIDS Behav, 2011. **15 Suppl 1**: p. S72-9.
10. Granich, R., et al., *Highly active antiretroviral treatment as prevention of HIV transmission: review of scientific evidence and update.* Curr Opin HIV AIDS, 2010. **5**(4): p. 298-304.
11. Cohen, M.S., et al., *Prevention of HIV-1 infection with early antiretroviral therapy.* N Engl J Med, 2011. **365**(6): p. 493-505.
12. World Health Organization (WHO). *Testing, New Directions in Treatment, and Measuring Impact: New WHO Guidelines*. in *IAS Conference on HIV Pathogenesis, Treatment and Prevention*. 2015. Vancouver, Canada.
13. Cohen, M.S. and S. Fidler, *HIV prevention 2010: where are we now and where are we going?* Curr Opin HIV AIDS, 2010. **5**(4): p. 265-8.
14. Weber, J., R. Tatoud, and S. Fidler, *Postexposure prophylaxis, preexposure prophylaxis or universal test and treat: the strategic use of antiretroviral drugs to prevent HIV acquisition and transmission.* AIDS, 2010. **24 Suppl 4**: p. S27-39.
15. World Health Organisation, *Consultation on Antiretroviral Treatment for Prevention*

of HIV Transmission. Meeting report, 2009.
16. Cohen, M.S. and C.L. Gay, *Treatment to prevent transmission of HIV-1.* Clin Infect Dis, 2010. **50 Suppl 3**: p. S85-95.
17. Centers for Disease Control and Prevention, *Vital signs: HIV prevention through care and treatment--United States.* MMWR Morb Mortal Wkly Rep, 2011. **60**: p. 1618-23.
18. Burns DN, D.C., Vermund SH, *Rethinking Prevention of HIV Type 1 Infection.* Clinical Infectious Diseases, 2010. **51**(6): p. 725-731.
19. Gardner EM, M.M., Steiner JF, Del Rio C, Burman WJ, *The Spectrum of Engagement in HIV Care and Its Relevance to Test-and-Treat Strategies for Prevention of HIV Infection.* Clinical Infectious Diseases, 2011. **52**(6): p. 793-800.
20. Abbas, U.L., R.M. Anderson, and J.W. Mellors, *Potential impact of antiretroviral chemoprophylaxis on HIV-1 transmission in resource-limited settings.* PLoS One, 2007. **2**(9): p. e875.
21. Abbas, U.L., R.M. Anderson, and J.W. Mellors, *Potential impact of antiretroviral therapy on HIV-1 transmission and AIDS mortality in resource-limited settings.* J Acquir Immune Defic Syndr, 2006. **41**(5): p. 632-41.
22. Blower, S., et al., *Predicting the impact of antiretrovirals in resource-poor settings: preventing HIV infections whilst controlling drug resistance.* Curr Drug Targets Infect Disord, 2003. **3**(4): p. 345-53.
23. Castilla, J., et al., *Effectiveness of highly active antiretroviral therapy in reducing heterosexual transmission of HIV.* J Acquir Immune Defic Syndr, 2005. **40**(1): p. 96-101.
24. Velasco-Hernandez, J.X., H.B. Gershengorn, and S.M. Blower, *Could widespread use of combination antiretroviral therapy eradicate HIV epidemics?* Lancet Infect Dis, 2002. **2**(8): p. 487-93.
25. Lima, V.D., et al., *Expanded access to highly active antiretroviral therapy: a potentially powerful strategy to curb the growth of the HIV epidemic.* J Infect Dis, 2008. **198**(1): p. 59-67.
26. Granich, R.M., et al., *Universal voluntary HIV testing with immediate antiretroviral therapy as a strategy for elimination of HIV transmission: a mathematical model.* Lancet, 2009. **373**(9657): p. 48-57.
27. Dodd, P.J., G.P. Garnett, and T.B. Hallett, *Examining the promise of HIV elimination by 'test and treat' in hyperendemic settings.* AIDS, 2010. **24**(5): p. 729-35.
28. Conway, B. and H. Tossonian, *Comprehensive Approaches to the Diagnosis and Treatment of HIV Infection in the Community: Can "Seek and Treat" Really Deliver?* Curr Infect Dis Rep, 2011. **13**(1): p. 68-74.

29. Zachariah, R., et al., *Antiretroviral therapy for HIV prevention: many concerns and challenges, but are there ways forward in sub-Saharan Africa?* Trans R Soc Trop Med Hyg, 2010. **104**(6): p. 387-91.
30. Sterne, J.A., et al., *Timing of initiation of antiretroviral therapy in AIDS-free HIV-1-infected patients: a collaborative analysis of 18 HIV cohort studies.* Lancet, 2009. **373**(9672): p. 1352-63.
31. Ray, M., et al., *The effect of combined antiretroviral therapy on the overall mortality of HIV-infected individuals.* AIDS, 2010. **24**(1): p. 123-37.
32. Kitahata, M.M., et al., *Effect of early versus deferred antiretroviral therapy for HIV on survival.* N Engl J Med, 2009. **360**(18): p. 1815-26.
33. Emery, S., et al., *Major clinical outcomes in antiretroviral therapy (ART)-naive participants and in those not receiving ART at baseline in the SMART study.* J Infect Dis, 2008. **197**(8): p. 1133-44.
34. Organization, W.H., *Global tuberculosis control: WHO report 2011*, 2011.
35. UNAIDS. *Zambia HIV AND AIDS ESTIMATES (2009)*. 2009; Available from: http://www.unaids.org/en/regionscountries/countries/zambia/.
36. UNAIDS. *South Africa HIV AND AIDS ESTIMATES (2009)*. 2009; Available from: http://www.unaids.org/en/regionscountries/countries/southafrica/.
37. Organization, W.H., *Global HIV/AIDS Response - Epidemic update and health sector progress towards Universal Access*, in *Progress Report 2011*2011.
38. Sloan, C.E., et al., *Newer drugs and earlier treatment: impact on lifetime cost of care for HIV-infected adults.* AIDS, 2012. **26**(1): p. 45-56.
39. Padian, N.S., et al., *Weighing the gold in the gold standard: challenges in HIV prevention research.* AIDS, 2010. **24**(5): p. 621-35.
40. Ross, D.A., *Behavioural interventions to reduce HIV risk: what works?* AIDS, 2010. **24 Suppl 4**: p. S4-14.
41. Cremin, I., et al., *Patterns of self-reported behaviour change associated with receiving voluntary counselling and testing in a longitudinal study from Manicaland, Zimbabwe.* AIDS Behav, 2010. **14**(3): p. 708-15.
42. Bello, G., et al., *Evidence for changes in behaviour leading to reductions in HIV prevalence in urban Malawi.* Sex Transm Infect, 2011. **87**(4): p. 296-300.
43. Coates, T.J., L. Richter, and C. Caceres, *Behavioural strategies to reduce HIV transmission: how to make them work better.* Lancet, 2008. **372**(9639): p. 669-84.
44. Corbett, E.L., et al., *HIV incidence during a cluster-randomized trial of two strategies providing voluntary counselling and testing at the workplace, Zimbabwe.* AIDS, 2007. **21**(4): p. 483-9.

45. Turner, A.N., et al., *Unprotected sex following HIV testing among women in Uganda and Zimbabwe: short- and long-term comparisons with pre-test behaviour.* Int J Epidemiol, 2009. **38**(4): p. 997-1007.
46. Auvert, B., et al., *Randomized, controlled intervention trial of male circumcision for reduction of HIV infection risk: the ANRS 1265 Trial.* PLoS Med, 2005. **2**(11): p. e298.
47. Bailey, R.C., et al., *Male circumcision for HIV prevention in young men in Kisumu, Kenya: a randomised controlled trial.* Lancet, 2007. **369**(9562): p. 643-56.
48. Gray, R.H., et al., *Male circumcision for HIV prevention in men in Rakai, Uganda: a randomised trial.* Lancet, 2007. **369**(9562): p. 657-66.
49. Hallett, T.B., et al., *Understanding the impact of male circumcision interventions on the spread of HIV in southern Africa.* PLoS One, 2008. **3**(5): p. e2212.
50. Lissouba, P., et al., *A model for the roll-out of comprehensive adult male circumcision services in African low-income settings of high HIV incidence: the ANRS 12126 Bophelo Pele Project.* PLoS Med, 2010. **7**(7): p. e1000309.
51. Ward, H. and M. Ronn, *Contribution of sexually transmitted infections to the sexual transmission of HIV.* Curr Opin HIV AIDS, 2010. **5**(4): p. 305-10.
52. Grosskurth, H., et al., *Impact of improved treatment of sexually transmitted diseases on HIV infection in rural Tanzania: randomised controlled trial.* Lancet, 1995. **346**(8974): p. 530-6.
53. Ng, B.E., et al., *Population-based biomedical sexually transmitted infection control interventions for reducing HIV infection.* Cochrane Database Syst Rev, 2011(3): p. CD001220.
54. Rerks-Ngarm, S., et al., *Vaccination with ALVAC and AIDSVAX to prevent HIV-1 infection in Thailand.* N Engl J Med, 2009. **361**(23): p. 2209-20.
55. Willyard, C., *Tiny steps towards an HIV vaccine.* Nature, 2010. **466**(7304): p. S8.
56. Barouch, D.H. and B. Korber, *HIV-1 vaccine development after STEP.* Annu Rev Med, 2010. **61**: p. 153-67.
57. Abdool Karim, Q., et al., *Effectiveness and safety of tenofovir gel, an antiretroviral microbicide, for the prevention of HIV infection in women.* Science, 2010. **329**(5996): p. 1168-74.
58. Greene, E., et al., *Acceptability and adherence of a candidate microbicide gel among high-risk women in Africa and India.* Cult Health Sex, 2010. **12**(7): p. 739-54.
59. Kamali, A., et al., *A randomised placebo-controlled safety and acceptability trial of PRO 2000 vaginal microbicide gel in sexually active women in Uganda.* Sex Transm Infect, 2010. **86**(3): p. 222-6.

60. Grant, R.M., et al., *Preexposure chemoprophylaxis for HIV prevention in men who have sex with men.* N Engl J Med, 2010. **363**(27): p. 2587-99.
61. Baeten, J. and C. Celum. *Antiretroviral pre-exposure prophylaxis for HIV-1 prevention among heterosexual African men and women: the Partners PrEP Study. Abstract No. MOAX0106*. in *6th International AIDS Society Conference on HIV Pathogenesis, Treatment and Prevention*. 2011. Rome, Italy.
62. Thigpen, M., et al. *Daily oral antiretroviral use for the prevention of HIV infection in heterosexually active young adults in Botswana: results from the TDF2 study Abstract No. WELBC01*. in *6th IAS Conference on HIV Pathogeneisis, Treatment and Prevention*. 2011 Rome, Italy
63. Microbicide Trials Network (MTN). *MTN Statement on Decision to Discontinue Use of Tenofovir Gel in VOICE, a Major HIV Prevention Study in Women.* 2011 [cited 2011 December 12]; Available from: http://www.mtnstopshiv.org/studies/70.
64. UNAIDS, *Getting to Zero: 2011-2015 Strategy.* http://www.unaids.org/en/media/unaids/contentassets/documents/unaidspublication/2010/JC2034_UNAIDS_Strategy_en.pdf, 2010.
65. Wilson, D. and D.T. Halperin, *"Know your epidemic, know your response": a useful approach, if we get it right.* Lancet, 2008. **372**(9637): p. 423-6.
66. Kurth, A.E., et al., *Combination HIV prevention: significance, challenges, and opportunities.* Curr HIV/AIDS Rep, 2011. **8**(1): p. 62-72.
67. Attia, S., et al., *Sexual transmission of HIV according to viral load and antiretroviral therapy: systematic review and meta-analysis.* AIDS, 2009. **23**(11): p. 1397-404.
68. Wawer, M.J., et al., *Rates of HIV-1 Transmission per Coital Act, by Stage of HIV-1 Infection, in Rakai, Uganda.* J Infect Dis, 2005. **191**(9): p. 1403-9.
69. Cohen, M.S., et al., *Narrative review: antiretroviral therapy to prevent the sexual transmission of HIV-1.* Ann Intern Med, 2007. **146**(8): p. 591-601.
70. Gay, C.L. and M.S. Cohen, *Antiretrovirals to prevent HIV infection: pre- and postexposure prophylaxis.* Curr Infect Dis Rep, 2008. **10**(4): p. 323-31.
71. Chasela, C.S., et al., *Maternal or infant antiretroviral drugs to reduce HIV-1 transmission.* N Engl J Med, 2010. **362**(24): p. 2271-81.
72. de Vincenzi, I., *Triple antiretroviral compared with zidovudine and single-dose nevirapine prophylaxis during pregnancy and breastfeeding for prevention of mother-to-child transmission of HIV-1 (Kesho Bora study): a randomised controlled trial.* Lancet Infect Dis, 2011. **11**(3): p. 171-80.
73. Shapiro, R.L., et al., *Antiretroviral regimens in pregnancy and breast-feeding in Botswana.* N Engl J Med, 2010. **362**(24): p. 2282-94.

74. Cohen, M.S., et al., *Prevention of HIV-1 Infection with Early Antiretroviral Therapy*. N Engl J Med, 2011.
75. Lawn, S.D., et al., *Early mortality among adults accessing antiretroviral treatment programmes in sub-Saharan Africa.* AIDS, 2008. **22**(15): p. 1897-908.
76. Donnell, D., et al., *Heterosexual HIV-1 transmission after initiation of antiretroviral therapy: a prospective cohort analysis.* The Lancet, 2010. **375**(9731): p. 2092-8.
77. Das, S., *Risk of cardiovascular disease in HIV-infected patients.* J Antimicrob Chemother, 2010. **65**(3): p. 386-9.
78. Montaner, J.S., et al., *Association of highly active antiretroviral therapy coverage, population viral load, and yearly new HIV diagnoses in British Columbia, Canada: a population-based study.* Lancet, 2010. **376**(9740): p. 532-9.
79. Garnett, G.P. and R.F. Baggaley, *Treating our way out of the HIV pandemic: could we, would we, should we?* Lancet, 2009. **373**(9657): p. 9-11.
80. Pinkerton, S.D., *Probability of HIV transmission during acute infection in Rakai, Uganda.* AIDS Behav, 2008. **12**(5): p. 677-84.
81. Brenner, B.G., et al., *High rates of forward transmission events after acute/early HIV-1 infection.* J Infect Dis, 2007. **195**(7): p. 951-9.
82. Hollingsworth, T.D., R.M. Anderson, and C. Fraser, *HIV-1 transmission, by stage of infection.* J Infect Dis, 2008. **198**(5): p. 687-93.
83. Bunnell, R., et al., *Changes in sexual behavior and risk of HIV transmission after antiretroviral therapy and prevention interventions in rural Uganda.* AIDS, 2006. **20**(1): p. 85-92.
84. Lundgren, J.D., et al., *Initiation of Antiretroviral Therapy in Early Asymptomatic HIV Infection.* N Engl J Med, 2015. **373**(9): p. 795-807.
85. Granich, R., et al., *Universal voluntary HIV testing with immediate antiretroviral therapy as a strategy for elimination of HIV transmission: a mathematical model.* Lancet, 2009. **373**(9657): p. 48-57.
86. Walensky, R.P., et al., *Test and treat DC: forecasting the impact of a comprehensive HIV strategy in Washington DC.* Clin Infect Dis, 2010. **51**(4): p. 392-400.
87. Johnston, K.M., et al., *Expanding access to HAART: a cost-effective approach for treating and preventing HIV.* AIDS, 2010. **24**(12): p. 1929-35.
88. Lawn, S.D., K. Kranzer, and R. Wood, *Antiretroviral therapy for control of the HIV-associated tuberculosis epidemic in resource-limited settings.* Clin Chest Med, 2009. **30**(4): p. 685-99, viii.
89. Williams, B.G., et al., *Antiretroviral therapy for tuberculosis control in nine African countries.* Proc Natl Acad Sci U S A, 2010. **107**(45): p. 19485-9.

90. Stephenson, R., et al., *The influence of motivational messages on future planning behaviors among HIV concordant positive and discordant couples in Lusaka, Zambia.* AIDS Care, 2008. **20**(2): p. 150-60.
91. Wood, R. and S.D. Lawn, *Antiretroviral treatment as prevention: impact of the 'test and treat' strategy on the tuberculosis epidemic.* Curr HIV Res, 2011. **9**(6): p. 383-92.
92. Lawn, S.D., et al., *Time to initiation of antiretroviral therapy among patients with HIV-associated tuberculosis in Cape Town, South Africa.* J Acquir Immune Defic Syndr, 2011. **57**(2): p. 136-40.
93. Sonnenberg, P., et al., *How soon after infection with HIV does the risk of tuberculosis start to increase? A retrospective cohort study in South African gold miners.* J Infect Dis, 2005. **191**(2): p. 150-8.
94. Badri, M., D. Wilson, and R. Wood, *Effect of highly active antiretroviral therapy on incidence of tuberculosis in South Africa: a cohort study.* Lancet, 2002. **359**(9323): p. 2059-64.
95. Leroy V, E.D., Becquet R, Viho I, Dequae-Merchadou L, Tonwe-Gold B, et al., *18-month effectiveness of short-course antiretroviral regimens combined with alternatives to breastfeeding to prevent HIV mother-to-child transmission.* PLoS One, 2008. **3**(2): p. 1645.
96. Thomas TK, M.R., Borkowf CB, Ndivo R, Zeh C, Misore A, et al., *Triple-antiretroviral prophylaxis to prevent mother-to-child HIV transmission through breastfeeding - the Kisumu Breastfeeding Study, Kenya: a clinical trial.* PLoS Med, 2011. **8**(3): p. 1001015.
97. Cooper ER, C.M., Mofenson L, Hanson IC, Pitt J, Diaz C, et al., *Combination antiretroviral strategies for the treatment of pregnant HIV-1-infected women and prevention of perinatal HIV-1 transmission.* J Acquir Immune Defic Syndr, 2002. **29**(5): p. 484-94.
98. Konikoff, J., et al., *Performance of a Limiting-Antigen Avidity Enzyme Immunoassay for Cross-Sectional Estimation of HIV Incidence in the United States.* PLoS One, 2013. **8**(12): p. e82772.
99. Cousins, M.M., et al., *HIV Diversity as a Biomarker for HIV Incidence Estimation: Including a High-Resolution Melting Diversity Assay in a Multiassay Algorithm.* J Clin Microbiol, 2014. **52**(1): p. 115-21.
100. Price, M.A., et al., *Transmitted HIV type 1 drug resistance among individuals with recent HIV infection in East and Southern Africa.* AIDS Res Hum Retroviruses, 2011. **27**(1): p. 5-12.
101. UNAIDS. *Report on the Global AIDS Epidemic*. 2010 12 May 2011.
102. Ayles, H., *Personal Communication 11 January 2012*.
103. Hollingsworth TD, A.R., Fraser C, *HIV-1 transmission, by stage of infection.* J Infect Dis, 2008. **198**: p. 687-693.

104. Hayes, R. and L. Moulton, *Cluster Randomised Trials*. Equations 7.132009.
105. Getahun, H., et al., *Development of a standardized screening rule for tuberculosis in people living with HIV in resource-constrained settings: individual participant data meta-analysis of observational studies.* PLoS Med, 2011. **8**(1): p. e1000391.
106. Harries, A.D., et al., *The HIV-associated tuberculosis epidemic--when will we act?* Lancet, 2010. **375**(9729): p. 1906-19.
107. Lawn, S.D., et al., *Antiretrovirals and isoniazid preventive therapy in the prevention of HIV-associated tuberculosis in settings with limited health-care resources.* Lancet Infect Dis, 2010. **10**(7): p. 489-98.
108. Organization, W.H., *Antiretroviral Treatment as Prevention (Ta sP) of HIV and TB*, 2012.
109. Weiss, H., M. Quigley, and R. Hayes, *Male circumcision and risk of HIV infection in sub-Saharan Africa: a systematic review and meta-analysis.* AIDS, 2000. **14**(15): p. 2361-2370.
110. Lissouba, P., et al., *Adult male circumcision as an intervention against HIV: an operational study of uptake in a South African community (ANRS 12126).* BMC Infect Dis, 2011. **11**: p. 253.
111. Wawer, M., G. Kigozi, and D. Swerwadda, *Trial of male circumcision in HIV+ men, Rakai Uganda: effects in HIV+ men and in women partners*, in *15th CROI*2008, CROI: Boston.
112. Mehta, S.D., et al., *Does sex in the early period after circumcision increase HIV-seroconversion risk? Pooled analysis of adult male circumcision clinical trials.* AIDS, 2009. **23**(12): p. 1557-64.
113. Weijer, C. and E.J. Emanuel, *Protecting communities in biomedical research.* Science, 2000. **289**: p. 1142-1144.

12.0 APPENDICES

APPENDIX I - SCHEDULES OF STUDY VISITS AND PROCEDURES

APPENDIX IA: POPULATION COHORT- ALL ARMS

PROCEDURES	Enrollment	12 Month Follow-Up	24 Month Follow-Up	36 Month Follow-Up
**ADMINISTRATIVE, BEHAVIORAL, AND REGULATORY PROCEDURES**				
Obtain informed consent for enrollment.<br>Solicit consent for storage of specimens for future testing and for access to data collected at health centers	X			
Obtain/update locator information.	X	X	X	X
Administer survey to include socio-demographic, health, social, behavioral, and economic factors	X	X	X	X
**CLINICAL/COUNSELING PROCEDURES**				
Perform HIV rapid testing[2]	X	X	X	X
Collect blood for laboratory testing and sample storage.	X	X	X	X
Provide HIV pre- and post-test counseling and test results, for those receiving HIV rapid testing and willing to receive results	X	X	X	X
**LABORATORY PROCEDURES**				
HIV testing[3]	X	X	X	X
HSV-2 testing[4]	X			[X][4]
Plasma storage[5]	X	X	X	X

<u>Footnotes for the *Population Cohort*</u>

[1] Consent for the *Phylogenetics Ancillary Study* will be solicited at the 12 month follow-up visit of the *Population Cohort.* If it is determined operationally feasible, consent may also be solicited at the 24 and 36 month follow-up visits for those participants not available to be offered participation at the 12 month follow-up visit. This decision will be subject to review and approval by the study team.

[2] Rapid testing will be offered at home visits and performed according to in-country guidelines. This testing will not be used to estimate HIV incidence or prevalence; however, the data may be captured along with other data from the home visit. Tie-breaker testing may or may not be performed in the home.

[3] Preliminary testing to assess HIV status will be performed in-country at a centralized laboratory. Additional HIV testing will be performed at the HPTN LC to confirm/determine HIV infection status. Results will not be returned to study sites or participants, unless directed by the HPTN LC for specific cases.

[4] Preliminary testing to assess HSV-2 status will be performed in-country at a centralized laboratory. Additional HSV-2 testing will be performed at the HPTN LC. HSV-2 testing will be performed for all participants at PC0; samples from PC36 will be selected for testing at the end of the study based on PC0 test results (see SSP Manual). Some samples may be tested for quality assurance (QA) assessments. Results will not be returned to study sites or participants.

[5] Plasma samples will be stored at in-country centralized laboratories. The study site will ship samples to the HPTN LC on a routine basis, and will ship additional samples as requested by the HPTN LC. Additional information will be provided in the SSP Manual. Information about the use of stored samples is provided in Section 9.2.

APPENDIX 1B- CASE-CONTROL STUDIES 1-3- ARMS A & B

PROCEDURES	Enrollment
**ADMINISTRATIVE, BEHAVIORAL, AND REGULATORY PROCEDURES**	
Obtain informed consent for enrollment	X
Complete questionnaire of socio-demographic, clinical, and behavioral characteristics	X

APPENDIX II - SAMPLE INFORMED CONSENT FORM – POPULATION COHORT

NOTE: Sample informed consent forms are adapted from NIH templates. It is understood that sites will modify these consents to meet the requirements of their setting and of their ethics committees. Modifications made locally to prior versions of the consents that have already been approved for use in-country are expected to be maintained in subsequent site-specific consent versions.

SUBJECT INFORMATION AND CONSENT FORM

**Title of Research Study:**	**Population Effects of Antiretroviral Therapy to Reduce HIV Transmission (PopART): A cluster-randomized trial of the impact of a combination prevention package on population-level HIV incidence in Zambia and South Africa**
**Protocol #:**	HPTN 071, Version 3.0 16 November 2015<br>DAIDS ID: 11865
**Sponsor:**	National Institute of Allergy and Infectious Diseases<br>National Institute of Mental Health<br>(U.S. National Institutes of Health)<br>Office of the United States Global AIDS Coordinator<br>Bill and Melinda Gates Foundation
**Investigator of Record:**	*(insert name)*
**Research Site Address(es):**	*(insert address)*
**Daytime telephone number(s):**	*(insert number)*
**24-hour contact number(s):**	*(insert number)*

Subject Information and Consent Form
Please ask the study investigator or the study staff to explain any words or procedures that you do not clearly understand.

The purpose of this form is to give you information about the research study you are being asked to join. If you sign this form, you will be giving your permission to take part in the study. The form describes the purpose, procedures, benefits, and risks of the research study. You should take part in the study only if you want to do so. You may choose not to join the research project or withdraw from this study at any time. Choosing not to take part in this research will not in any way affect the health care or benefits that you or your family will receive. Please read this

Subject Information and Consent Form and ask as many questions as needed. You should not sign this form if you have any questions that have not been answered to your satisfaction.

This study is being funded by the U.S. National Institutes of Health, the Office of the United States Global AIDS Coordinator, and the Bill and Melinda Gates Foundation

Your participation is voluntary
You do not have to take part in this study. If you decide today to take part in this research project, you may refuse to take part in any portion of the study or stop at any time without reducing or affecting any care that you receive at the health centers in your community.

Purpose of the Research in the Communities
The HPTN 071 or PopART study is testing a program to try to reduce HIV infection in a community like yours. Twenty one communities that include about 600,000 adults are included in this research (about 400,000 adults in twelve Zambian communities and 200,000 adults in nine South African communities).

In some communities, the level of care that people are used to will stay the same, in terms of HIV testing, and care of those who have HIV.

In other communities, to make HIV testing easier, community health care workers will go to all homes and will offer to do an HIV test on each adult (or younger people with permission of guardian) wishing to have a test. For anyone infected with HIV, they will be offered to start taking drugs to treat HIV according to the standard treatment guidelines that are in place for doing so in your country. The health workers will visit every home again once a year for up to three more years to repeat the HIV testing and to refer people to care.

In other communities, health care workers will go to all houses offering HIV testing, as was just described. In these communities if someone tests HIV positive however, they will be offered to start taking medicines to treat HIV right away. The health workers will visit every home again once a year for up to three more years to repeat the HIV testing and to refer people to care.

At the end of the study, the researchers will see if offering HIV tests in each household and offering people the chance to start HIV treatment right away has reduced HIV infection. They will also see if starting ART early has any negative effects on people’s health.

Your community is one of the communities participating in this research. If health care workers are visiting homes in your community, you will notice that they provide some other information and services to people, but the most important thing is the testing and HIV treatment they offer.

In each community, around 2,700 people will be asked to participate in additional activities such as completing questionnaires and providing additional samples for laboratory testing. These questionnaires and tests will let the researchers understand how the community feels about the program and if the program is working. You have been selected to be one of the people from your community who we are asking to participate in these additional activities. That is why you are being asked to read this document.

What will happen during this study?
If you participate in this study, you will have up to four study visits: today, in 12 months, in 24 months, and possibly a final visit in 36 months. We will contact you to remind you about your visits. For example, we may call you or send a short text message (SMS). Today's visit will take approximately 2 hours. Future visits may be slightly shorter. Today we will:

- Ask you questions about a number of topics including you and your sexual practices, HIV testing, male circumcision, and how you and others feel about HIV.
- Collect up to 15 mL blood (about 3 teaspoons) for HIV testing and other HIV-related tests as well as herpes simplex-2 testing. Some blood will be stored for study-related testing.

Sometimes at the end of a study, some blood or other specimens are left-over that could be useful for testing in the future. These tests would be for research that is not a part of this study. If you agree to participate in the study, we will also ask if you if are willing to let us keep your left-over samples for future tests.

Some specimens will be shipped and/or securely stored outside of the country for study-related testing, long-term storage, and future testing.

If you agree to participate in the study, we will offer to perform an on-the-spot HIV test at each visit, and will provide counseling if you would like to know the result of your test. If these tests say that you are positive for HIV, we will refer you for care at the local health center. The staff at the health center keep records of all their patients as part of their normal procedures. We would like to look at these medical records for any study participant who is HIV infected. Doing so will help us better understand how the study activities in the community are affecting the health of people diagnosed with HIV. If you agree to participate in this study, we will ask you for your permission to look at your records at the health center. This may include information collected by the community health workers if they are visiting homes in your community.

What are the possible risks or discomforts?
You may become embarrassed, worried or anxious when learning your HIV status and discussing sexual risk behavior and other topics. A trained staff member will help you deal with any feelings or questions you have. You may feel that being part of this study could lead to you feeling stigmatized or separated from our community

It is very unusual to have any problems from having a blood test but you may feel discomfort, dizzy, or even faint when your blood is drawn. Redness, pain, swelling, bruising may occur where the needle goes into your arm but this is rare.

What are the potential benefits?
During the study, you can decide if you would like to learn your HIV status and be provided with information on where to receive treatment and care services if needed. You will also be able to ask questions about your health.

In addition, knowledge gained from this study may help reduce the spread of HIV in the future and promote better health for you and your family as well as helping with acknowledgement and acceptance of HIV as a community-wide health problem.

Are there any alternatives to participation?
If you decide not to participate in this study, we will refer you to other places where you can receive an HIV test. If it is offered in your community, you can also receive testing from a health worker visiting your home during the study period.

How will my confidentiality and privacy be protected?
We cannot guarantee absolute confidentiality. However, we will do everything possible to protect your confidentiality if you join this study. We do this by giving you a study number and any information will be labeled with this number only, so people working in the health centers and laboratories will only see a number not your name, only the research staff will be able to link this number to your name. Your personal information (name, address, phone number) will be protected by the research staff. This information will not be used in any publication of information about this study.

To protect your privacy, you will meet with the researcher in a private area where others cannot overhear conversations with you.

People who may review your records include: [insert name of site IRB/EC], local regulatory agencies, US National Institutes of Health (NIH), study staff, and study monitors. Institutional Review Boards (IRBs) or Ethics Committees (ECs) are committees that watch over the safety and rights of research participants.

What happens if I am injured by participating in this study?
It is very unlikely that you could be injured as a result of participating in this study. However, if you are injured while participating in this study, you will be given immediate treatment for your injuries. You [will/will not] have to pay for this care. There [is a/is no] program for compensation either through this institution or the United States NIH. You will not be giving up any of your legal rights by signing this Subject Information and Consent Form.

What are some reasons why I may be withdrawn from this activity without my consent?
You may be withdrawn from the study without your consent for the following reasons:

- The research study, or this part of the study, is stopped or canceled
- The study staff feels that completing the study or this part of the study would be harmful to you or others

Persons to Contact for Problems or Questions
If you have any questions about your participation in this research study, your rights as a research subject, or if you feel that you have experienced a research-related injury, contact:

Investigator of Record Name: *(site insert name of the investigator or other study staff)*

Research Site Address(es): *(site insert physical address of above)*

Daytime telephone number(s): *(site insert telephone number)*

24-hour contact number(s): *(site insert telephone number)*

If you have any questions or concerns about your rights as a research subject or want to discuss a problem, get information or offer input, you may contact:

Independent Review Board/Ethics Committee: *(site insert name or title of person on the IRB, EC or other organization appropriate for the site)*

Address of Independent Review Board: *(site insert physical address of above)*

Daytime Telephone Number: *(site insert telephone number of above)*

SUBJECT'S STATEMENT OF CONSENT

Population Effects of Antiretroviral Therapy to Reduce HIV Transmission (PopART): A cluster-randomized trial of the impact of a combination prevention package on population-level HIV incidence in Zambia and South Africa

- I have been given sufficient time to consider whether to take part in this study.
- My taking part in this research study is voluntary. I may decide not to take part or to withdraw from the research study at any time without penalty or loss of benefits or treatment to which I am entitled.
- The research study may be stopped at any time without my consent.
- I have had an opportunity to ask my study investigator questions about this research study. My questions so far have been answered to my satisfaction.
- I have been told how long I may be in the research study.
- I have been informed of the procedures and tests that may be performed during the research study.
- I have been told what the possible risks and benefits are from taking part in this research study. I may not benefit if I take part in this research study.
- I do not give up my legal rights by signing this form.
- I have been told that before any study related procedures being performed, I will be asked to voluntarily sign this Subject Information and Consent Form.
- I will receive a signed and dated copy of this Subject Information and Consent Form.

If you have either read or have heard the information in this Subject Information and Consent Form, if all of your questions have been answered, and if you agree to take part in the study, please print and sign your name and write the date on the line below.

Specimen Storage for Future Testing

_____ My initials indicate that any left-over blood or other specimens may be stored for future testing after the study has ended. I understand that any future research on my specimens may need to be approved by an ethics committee.

_____ I do not agree to allow leftover samples to be saved for long-term storage and future testing after the study has ended.

Access of Data from Heath Center

_____ My initials indicate that I agree to allow my records at the health center to be accessed and used for this study.

_____ I do not agree to allow my health care records to be accessed and used for this study.

I voluntarily agree to take part in this research study.

_________________________	_________________________
Subject's Name (print)	Subject's Signature and Date

I certify that the information provided was given in a language that was understandable to the subject.

_________________________	_________________________
Name of Study Staff<br>Conducting Consent Discussion (print)	Study Staff Signature and Date
_________________________	_________________________
Witness' Name (print)<br>(As appropriate) Date	Witness' Signature and Date

APPENDIX III - SAMPLE INFORMED CONSENT FORM – QUALITATIVE STUDIES PARTICIPANTS

NOTE: Sample informed consent forms are adapted from NIH templates. It is understood that sites will modify these consents to meet the requirements of their setting and of their ethics committees. Modifications made locally to prior versions of the consents that have already been approved for use in-country are expected to be maintained in subsequent site-specific consent versions.

SUBJECT INFORMATION AND CONSENT FORM

**Title of Research Study:**	**Population Effects of Antiretroviral Therapy to Reduce HIV Transmission (PopART): A cluster-randomized trial of the impact of a combination prevention package on population-level HIV incidence in Zambia and South Africa**
**Protocol #:**	HPTN 071, Version 3.0 16 November 2015<br>DAIDS ID: 11865
**Sponsor:**	National Institute of Allergy and Infectious Diseases<br>National Institute of Mental Health<br>(U.S. National Institutes of Health)<br>Office of the United States Global AIDS Coordinator<br>Bill and Melinda Gates Foundation
**Investigator of Record:**	*(insert name)*
**Research Site Address(es):**	*(insert address)*
**Daytime telephone number(s):**	*(insert number)*
**24-hour contact number(s):**	*(insert number)*

Subject Information and Consent Form
Please ask the study investigator or the study staff to explain any words or procedures that you do not clearly understand.

The purpose of this form is to give you information about the research study you are being asked to join. If you sign this form, you will be giving your permission to take part in the study. The form describes the purpose, procedures, benefits, and risks of the research study. You should take part in the study only if you want to do so. You may choose not to join the research project or withdraw from this study at any time. Choosing not to take part in this research will not in any way affect the health care or benefits that you or your family will receive. Please read this Subject Information and Consent Form and ask as many questions as needed. You should not sign this form if you have any questions that have not been answered to your satisfaction.

This study is being funded by the U.S. National Institutes of Health, the Office of the United States Global AIDS Coordinator, and the Bill and Melinda Gates Foundation

Your participation is voluntary
You do not have to take part in this study. If you decide today to take part in this research project, you may refuse to take part in any portion of the study or stop at any time without reducing or affecting any care that you receive at the health centers in your community.

Purpose of the Research in the Communities
The HPTN 071 or PopART study is testing a program to try to reduce HIV infection in a community like yours. Twenty one communities that include about 600,000 adults are included in this research (about 400,000 adults in twelve Zambian communities and 200,000 adults in nine South African communities).

In some communities, the level of care that people are used to will stay the same, in terms of HIV testing, and care of those who have HIV.

In other communities, to make HIV testing easier, community health care workers will go to all homes and will offer to do an HIV test on each adult (or younger people with permission of guardian) wishing to have a test. For anyone infected with HIV, they will be offered to start taking drugs to treat HIV according to the standard treatment guidelines that are in place for doing so in your country. The health workers will visit every home again once a year for up to three more years to repeat the HIV testing and to refer people to care.

In other communities, health care workers will go to all houses offering HIV testing, as was just described. In these communities if someone tests HIV positive however, they will be offered to start taking medicines to treat HIV right away. The health workers will visit every home again once a year for up to three more years to repeat the HIV testing and to refer people to care.

At the end of the study, the researchers will see if offering HIV tests in each household and offering people the chance to start HIV treatment right away has reduced HIV infection.

Your community is one of the communities participating in this research. If health care workers are visiting homes in your community, you will notice that they provide some other information and services to people, but the most important thing is the testing and HIV treatment they offer.

In each community, around 2,700 people will be asked to participate in additional activities such as completing questionnaires and providing additional samples for laboratory testing. These questionnaires and tests will let the researchers understand how the community feels about the program and if the program is working. You have been selected to be one of the people from your community who we are asking to participate in these additional activities. That is why you are being asked to read this document.

What will happen during this study?

[*For participants providing a single interview*]
If you agree to participate in this study, you will have one interview today. We will ask you questions about the reasons why people in this community chose to test for HIV or not to test for HIV. We will also ask about how people in this community experience HIV treatment and any other HIV prevention methods.

[*For participants being followed longitudinally*]
If you agree to participate in this study, we will interview you every three months until the end of the study. We will ask you questions about the reasons why people in this community chose to test for HIV or not to test for HIV. We will also ask about how people in this community experience HIV treatment and any other HIV prevention methods.

[*For individuals participating in a focus group*]
You have been selected to participate in this group discussion because of either your knowledge of the community or your association with HIV/AIDS related programs and activities in this community. If you agree to participate in this study, you will be a part of a group and questions will be directed towards the group, but you are free to answer any question and comment on the answers of others. In some instances, the facilitator may ask you to elaborate on your answer for the benefit of others but you may choose not to if you are not entirely comfortable with the request. The questions will be broad/ general in nature and will touch on many aspects of the community's experiences with HIV.

What are the possible risks or discomforts?
The risk to you in participating in this study is that some of the questions may be uncomfortable and may make you feel worried or embarrassed. If any of the questions make you feel upset, the interviewer may go to another question or totally stop the interview.

There is also a risk that following up individuals at home may lead to rumors in the community. To minimize this risk we will ask you to propose places where we can talk in private.

What are the potential benefits?
You will not receive any direct benefit from being in this study. You or others may benefit in the future from the information learned in this study.

Are there any alternatives to participation?
If you decide not to participate in this study, you can still receive HIV tests and other services from your local health center.

How will my confidentiality and privacy be protected?
We cannot guarantee absolute confidentiality. However, we will do everything possible to protect your confidentiality if you join this study. We do this by giving you a study number and any information you provide will be labeled with this number only, not your name. Only the research staff will be able to link this number to your name. Your personal information (name, address, phone number) will be protected by the research staff. This information will not be used in any publication of information about this study.

To protect your privacy, you will meet with the researcher in a private area where others cannot overhear conversations with you.

People who may review your records include: [insert name of site IRB/EC], local regulatory agencies, US National Institutes of Health (NIH), study staff, and study monitors. Institutional Review Boards (IRBs) or Ethics Committees (ECs) are committees that watch over the safety and rights of research participants.

What happens if I am injured by participating in this study?
It is very unlikely that you could be injured as a result of participating in this study. However, if you are injured while participating in this study, you will be given immediate treatment for your injuries. You [will/will not] have to pay for this care. There [is a/is no] program for compensation either through this institution or the United States NIH. You will not be giving up any of your legal rights by signing this Subject Information and Consent Form.

What are some reasons why I may be withdrawn from this activity without my consent?
You may be withdrawn from the study without your consent for the following reasons:

- The research study, or this part of the study, is stopped or canceled
- The study staff feels that completing the study or this part of the study would be harmful to you or others

Persons to Contact for Problems or Questions
If you have any questions about your participation in this research study, your rights as a research subject, or if you feel that you have experienced a research-related injury, contact:

Investigator of Record Name: *(site insert name of the investigator or other study staff)*

Research Site Address(es): *(site insert physical address of above)*

Daytime telephone number(s): *(site insert telephone number)*

24-hour contact number(s): *(site insert telephone number)*

If you have any questions or concerns about your rights as a research subject or want to discuss a problem, get information or offer input, you may contact:

Independent Review Board/Ethics Committee: *(site insert name or title of person on the IRB/EC or other organization appropriate for the site)*

Address of Independent Review Board: *(site insert physical address of above)*

Daytime Telephone Number: *(site insert telephone number of above)*

SUBJECT'S STATEMENT OF CONSENT

Population Effects of Antiretroviral Therapy to Reduce HIV Transmission (PopART): A cluster-randomized trial of the impact of a combination prevention package on population-level HIV incidence in Zambia and South Africa

- I have been given sufficient time to consider whether to take part in this study.
- My taking part in this research study is voluntary. I may decide not to take part or to withdraw from the research study at any time without penalty or loss of benefits or treatment to which I am entitled.
- The research study may be stopped at any time without my consent.
- I have had an opportunity to ask my study investigator questions about this research study. My questions so far have been answered to my satisfaction.
- I have been told how long I may be in the research study.
- I have been informed of the procedures and tests that may be performed during the research study.
- I have been told what the possible risks and benefits are from taking part in this research study. I may not benefit if I take part in this research study.
- I do not give up my legal rights by signing this form.
- I have been told that before any study related procedures being performed, I will be asked to voluntarily sign this Subject Information and Consent Form.
- I will receive a signed and dated copy of this Subject Information and Consent Form.

If you have either read or have heard the information in this Subject Information and Consent Form, if all of your questions have been answered, and if you agree to take part in the study, please print and sign and your name and write the date on the line below.

I voluntarily agree to take part in this research study.

________________________	________________________
Subject's Name (print)	Subject's Signature and Date

I certify that the information provided was given in a language that was understandable to the subject.

________________________	________________________
Name of Study Staff<br>Conducting Consent Discussion (print)	Study Staff Signature and Date

________________________	________________________
Witness' Name (print)<br>(As appropriate) Date	Witness' Signature and Date

APPENDIX IV - SAMPLE INFORMED CONSENT FORM – CASE CONTROL STUDIES PARTICIPANTS

NOTE: Sample informed consent forms are adapted from NIH templates. It is understood that sites will modify these consents to meet the requirements of their setting and of their ethics committees. Modifications made locally to prior versions of the consents that have already been approved for use in-country are expected to be maintained in subsequent site-specific consent versions.

SUBJECT INFORMATION AND CONSENT FORM

**Title of Research Study:**	**Population Effects of Antiretroviral Therapy to Reduce HIV Transmission (PopART): A cluster-randomized trial of the impact of a combination prevention package on population-level HIV incidence in Zambia and South Africa**
**Protocol #:**	HPTN 071, Version 3.0 16 November 2015<br>DAIDS ID: 11865
**Sponsor:**	National Institute of Allergy and Infectious Diseases<br>National Institute of Mental Health<br>(U.S. National Institutes of Health)<br>Office of the United States Global AIDS Coordinator<br>Bill and Melinda Gates Foundation
**Investigator of Record:**	*(insert name)*
**Research Site Address(es):**	*(insert address)*
**Daytime telephone number(s):**	*(insert number)*
**24-hour contact number(s):**	*(insert number)*

Subject Information and Consent Form
Please ask the study investigator or the study staff to explain any words or procedures that you do not clearly understand.

The purpose of this form is to give you information about the research study you are being asked to join. If you sign this form, you will be giving your permission to take part in the study. The form describes the purpose, procedures, benefits, and risks of the research study. You should take part in the study only if you want to do so. You may choose not to join the research project or withdraw from this study at any time. Choosing not to take part in this research will not in any way affect the health care or benefits that you or your family will receive. Please read this Subject Information and Consent Form and ask as many questions as needed. You should not sign this form if you have any questions that have not been answered to your satisfaction.

This study is being funded by the U.S. National Institutes of Health, the Office of the United States Global AIDS Coordinator, and the Bill and Melinda Gates Foundation

Your participation is voluntary
You do not have to take part in this study. If you decide today to take part in this research project, you may refuse to take part in any portion of the study or stop at any time without reducing or affecting any care that you receive at the health centers in your community.

Purpose of the Research in the Communities
The HPTN 071 or PopART study is testing a program to try to reduce HIV infection in a community like yours. Twenty one communities that include about 600,000 adults are included in this research (about 400,000 adults in twelve Zambian communities and 200,000 adults in nine South African communities).

In some communities, the level of care that people are used to will stay the same, in terms of HIV testing, and care of those who have HIV.

In other communities, to make HIV testing easier, community health care workers will go to all homes and will offer to do an HIV test on each adult (or younger people with permission of guardian) wishing to have a test. For anyone infected with HIV, they will be offered to start taking drugs to treat HIV according to the standard treatment guidelines that are in place for doing so in your country. The health workers will visit every home again once a year for up to three more years to repeat the HIV testing and to refer people to care.

In other communities, health care workers will go to all houses offering HIV testing, as was just described. In these communities if someone tests HIV positive however, they will be offered to start taking medicines to treat HIV right away. The health workers will visit every home again once a year for up to three more years to repeat the HIV testing and to refer people to care.

At the end of the study, the researchers will see if offering HIV tests in each household and offering people the chance to start HIV treatment right away has reduced HIV infection.

Your community is one of the communities participating in this research. If health care workers are visiting homes in your community, you will notice that they provide some other information and services to people, but the most important thing is the testing and HIV treatment they offer.

In each community, around 2,700 people will be asked to participate in additional activities such as completing questionnaires and providing additional samples for laboratory testing. These questionnaires and tests will let the researchers understand how the community feels about the program and if the program is working. You have been selected to be one of the people from your community who we are asking to participate in these additional activities. That is why you are being asked to read this document.

What will happen during this study?
You will have one study visit which will occur today. This visit will take approximately 1 hour. During this visit, a researcher will ask you questions about sexual behavior, health services, previous HIV testing, HIV-related stigma and other HIV-related questions.

What are the possible risks or discomforts?
The risk to you in participating in this study is that some of the questions may be uncomfortable and may make you feel worried or embarrassed. If any of the questions make you feel upset, the interviewer may go to another question or totally stop the interview.

What are the potential benefits?
You will not receive any direct benefit from being in this study. You or others may benefit in the future from the information learned in this study.

Are there any alternatives to participation?
If you decide not to participate in this study, you can still receive HIV tests and other services from your local health center.

How will my confidentiality and privacy be protected?
We cannot guarantee absolute confidentiality. However, we will do everything possible to protect your confidentiality if you join this study. We do this by giving you a study number and any information you provide will be labeled with this number only, not your name. Only the research staff will be able to link this number to your name. Your personal information (name, address, phone number) will be protected by the research clinic. This information will not be used in any publication of information about this study.

To protect your privacy, you will meet with the researcher in a private area where others cannot overhear conversations with you.

People who may review your records include: [insert name of site IRB/EC], local regulatory agencies, US National Institutes of Health (NIH), study staff, and study monitors. Institutional Review Boards (IRBs) or Ethics Committees (ECs) are committees that watch over the safety and rights of research participants.

What happens if I am injured by participating in this study?
It is very unlikely that you could be injured as a result of participating in this study. However, if you are injured while participating in this study, you will be given immediate treatment for your injuries. You [will/will not] have to pay for this care. There [is a/is no] program for compensation either through this institution or the United States NIH. You will not be giving up any of your legal rights by signing this Subject Information and Consent Form.

What are some reasons why I may be withdrawn from this activity without my consent?
You may be withdrawn from the study without your consent for the following reasons:

- The research study, or this part of the study, is stopped or canceled
- The study staff feels that completing the study or this part of the study would be harmful to you or others

Persons to Contact for Problems or Questions
If you have any questions about your participation in this research study, your rights as a research subject, or if you feel that you have experienced a research-related injury, contact:

Investigator of Record Name: *(site insert name of the investigator or other study staff)*

Research Site Address(es): *(site insert physical address of above)*

Daytime telephone number(s): *(site insert telephone number)*

24-hour contact number(s): *(site insert telephone number)*

If you have any questions or concerns about your rights as a research subject or want to discuss a problem, get information or offer input, you may contact:

Independent Review Board/Ethics Committee: *(site insert name or title of person on the IRB/EC or other organization appropriate for the site)*

Address of Independent Review Board:*(site insert physical address of above)*

Daytime Telephone Number: *(site insert telephone number of above)*

SUBJECT'S STATEMENT OF CONSENT

Population Effects of Antiretroviral Therapy to Reduce HIV Transmission (PopART): A cluster-randomized trial of the impact of a combination prevention package on population-level HIV incidence in Zambia and South Africa

- I have been given sufficient time to consider whether to take part in this study.
- My taking part in this research study is voluntary. I may decide not to take part or to withdraw from the research study at any time without penalty or loss of benefits or treatment to which I am entitled.
- The research study may be stopped at any time without my consent.
- I have had an opportunity to ask my study investigator questions about this research study. My questions so far have been answered to my satisfaction.
- I have been told how long I may be in the research study.
- I have been informed of the procedures and tests that may be performed during the research study.
- I have been told what the possible risks and benefits are from taking part in this research study. I may not benefit if I take part in this research study.
- I do not give up my legal rights by signing this form.
- I have been told that before any study related procedures being performed, I will be asked to voluntarily sign this Subject Information and Consent Form.
- I will receive a signed and dated copy of this Subject Information and Consent Form.

If you have either read or have heard the information in this Subject Information and Consent Form, if all of your questions have been answered, and if you agree to take part in the study, please print and sign and your name and write the date on the line below.

I voluntarily agree to take part in this research study.

___________________________	_______________________________________
Subject's Name (print)	Subject's Signature and Date

I certify that the information provided was given in a language that was understandable to the subject.

___________________________	_______________________________________
Name of Study Staff<br>Conducting Consent Discussion (print)	Study Staff Signature and Date

___________________________	_______________________________________
Witness' Name (print)<br>(As appropriate) Date	Witness' Signature and Date

APPENDIX V - SAMPLE INFORMED CONSENT FORM – PARTICIPANTS STARTING ART OUTSIDE OF LOCAL GUIDELINES

> ***NOTE:*** Sample informed consent forms are adapted from NIH templates. It is understood that sites will modify these consents to meet the requirements of their setting and of their ethics committees. Modifications made locally to prior versions of the consents that have already been approved for use in-country are expected to be maintained in subsequent site-specific consent versions.

SUBJECT INFORMATION AND CONSENT FORM

**Title of Research Study:**	**Population Effects of Antiretroviral Therapy to Reduce HIV Transmission (PopART): A cluster-randomized trial of the impact of a combination prevention package on population-level HIV incidence in Zambia and South Africa**
**Protocol #:**	HPTN 071, Version 3.0 16 November 2015<br>DAIDS ID: 11865
**Sponsor:**	National Institute of Allergy and Infectious Diseases<br>National Institute of Mental Health<br>(U.S. National Institutes of Health)<br>Office of the United States Global AIDS Coordinator<br>Bill and Melinda Gates Foundation
**Investigator of Record:**	*(insert name)*
**Research Site Address(es):**	*(insert address)*
**Daytime telephone number(s):**	*(insert number)*
**24-hour contact number(s):**	*(insert number)*

Subject Information and Consent Form
Please ask the study investigator or the study staff to explain any words or procedures that you do not clearly understand.

The purpose of this form is to give you information about the research study you are being asked to join. If you sign this form, you will be giving your permission to take part in the study. The form describes the purpose, procedures, benefits, and risks of the research study. You should take part in the study only if you want to do so. You may choose not to join the research project or withdraw from this study at any time. Choosing not to take part in this research will not in any way affect the health care or benefits that you or your family will receive. Please read this Subject Information and Consent Form and ask as many questions as needed. You should not sign this form if you have any questions that have not been answered to your satisfaction.

This study is being funded by the U.S. National Institutes of Health, the Office of the United States Global AIDS Coordinator, and the Bill and Melinda Gates Foundation.

Your participation is voluntary

You do not have to take part in this study. If you decide today to take part in this research project, you may refuse to take part in any portion of the study or stop at any time without reducing or affecting any care that you receive at the clinics in your community.

Purpose of the Research in the Communities

The HPTN 071 or PopART study is testing a program to try to reduce HIV infection in a community like yours. Twenty one communities that include about 600,000 adults are included in this research (about 400,000 adults in twelve Zambian communities and 200,000 adults in nine South African communities).

In some communities, the level of care that people are used to will stay the same, in terms of HIV testing.

In other communities, to make HIV testing easier, community health care workers will go to all homes and will offer to do an HIV test on each adult (or younger people with permission of guardian) wishing to have a test. The health workers will visit every home again once a year for up to three more years to repeat the HIV testing and to refer people to care.

In all communities in the study, anyone infected with HIV will be offered to start taking medicines to treat HIV right away, even if local treatment guidelines would typically require people to wait before starting treatment. Other studies have shown that if someone with HIV starts treatment right away, they are much less likely to pass HIV to their partner.

At the end of the study, the researchers will see if offering HIV tests in each household and offering people the chance to start HIV treatment right away has reduced HIV infection.

What will happen during this study?

If you agree to participate in this study, you will start taking anti-HIV drugs immediately. Local guidelines suggest starting people on anti-HIV drugs when their immune cells, called CD4 cells, drop below a certain level. So by agreeing to this study you would begin taking anti-HIV drugs sooner than if you waited for treatment under local guidelines.

It is expected that local guidelines will change in the future to allow all HIV-infected patients to start taking anti-HIV drugs immediately. This is because studies show that taking anti-HIV drugs improves the health of a person with HIV and prevents them from passing HIV to their partners. But local guidelines have not changed yet.

Your clinic visit schedule and routine health testing will occur according to the local standards at this clinic.

What are the possible risks or discomforts?

Anti-HIV Drugs:

There are many drugs available to treat HIV and AIDS. The doctors in the clinic will determine the best combination of these drugs to treat you. It is possible that the drugs may make you feel sick or will affect your blood tests, in which case the doctors may either switch you to different drugs, or stop them all together. It is very important for you to return to the clinic whenever you feel sick. Feeling sick may be due to the pills or it may be due to a sickness caused by your HIV infection or it may be caused by something completely different, such as malaria. Either way, you should return to the clinic so that you can be treated.

As with any medication, anti-HIV drugs can cause side effects. Most of the medicines for HIV are very safe and are well tolerated with only very few side effects. Some of these side effects are mild and may go away after you have taken the drugs for a few weeks. Examples of these types of side effects include upset stomach, vomiting, headache, and changes in your mood, sleep, or concentration. Other side effects can be severe but are rare and may require treatment or hospitalization. Examples of these types of side effects include rash, liver problems, severe depression or psychosis, and pancreas problems.

If you take your anti-HIV medicines very regularly they will work and keep the amount of virus in your body low. If for any reason you do not keep taking the medicines every day, the amount of virus in your body can increase and the anti-HIV pills you are taking may stop working against the virus (the virus becomes resistant), and your doctors will have fewer medicines to choose from to try to keep you healthy. If that happens, the doctors will try to give you different drugs that will work.

A doctor will explain all of the possible side-effects of any drugs before you begin taking them.

There is a risk of serious and life-threatening side effects when other drugs are taken with anti-HIV medications. For your safety, you must tell your doctor about all medications you are taking before you start taking anti-HIV medications.

<u>Risks Associated with Early versus Delayed Treatment with Anti-HIV Drugs</u>:
If you agree, you will begin taking anti-HIV drugs immediately. If you begin the drugs immediately, there is a chance that when you start taking the medicines, especially at the beginning, the drugs may make you feel sick. As with any medication some drugs may have side effects so severe that the nurse or doctor may need to take you off that drug and give you another. It is important that the medical teams check that the drugs you start taking are safe for you and change the drugs if they are not.
It is really important that once you start taking anti-HIV medicines, you try to take them every single day and do not miss doses, share tablets with other people or suddenly stop them. If you take the tablets but only very irregularly then there is a chance that they will no longer work against your virus and the virus will becomes ‘resistant’ to the medicine. If this happens it limits the choices for other treatment and if it carries on there may be no medicines that can work to suppress your virus when you become sick.

What are the potential benefits?
At the moment, the national guidelines inform medical teams when to start ART and this is decided based on a measure of your immune system (CD4 cell count). Doctors and researchers

are always trying to find better ways to keep people healthy and new research has shown that starting ART earlier may be better for your health.

In addition to providing health benefits for you, taking ART to suppress the HIV virus has been shown to reduce the risk of passing on HIV to sexual partners or babies. Also, starting treatment early may help prevent Tuberculosis which occurs more often in people who are HIV positive.

There is no cure for HIV and no method is 100% effective in preventing the spread of HIV, except abstinence. ART does not protect you from getting other infections that can be passed on through unprotected sex, so it is important to continue using condoms correctly during every sex act.

Are there any alternatives to participation?
If you do not agree to take anti-HIV drugs at this time, you can still be seen here at the health clinic for HIV care and you will be offered treatment according to the local guidelines.

How will my confidentiality and privacy be protected?
We cannot guarantee absolute confidentiality. However, we will do everything possible to protect your confidentiality if you join this study. We do this by giving you a study number and any information will be labeled with this number only, so people working in the clinics and laboratories will only see a number not your name, only the research staff will be able to link this number to your name. Your personal information (name, address, phone number) will be protected by the research clinic. This information will not be used in any publication of information about this study.

To protect your privacy, you will meet with the researcher in a private area where others cannot overhear conversations with you.

People who may review your records include: [insert name of site IRB/EC], local regulatory agencies, US National Institutes of Health (NIH), study staff, and study monitors. Institutional Review Boards (IRBs) or Ethics Committees (ECs) are committees that watch over the safety and rights of research participants.

What happens if I am injured by participating in this study?
It is very unlikely that you could be injured as a result of participating in this study. However, if you are injured while participating in this study, you will be given immediate treatment for your injuries. You [will/will not] have to pay for this care. There [is a/is no] program for compensation either through this institution or the United States NIH. You will not be giving up any of your legal rights by signing this Subject Information and Consent Form.

What are some reasons why I may be withdrawn from this study without my consent?

If you agree to begin taking anti-HIV drugs at this time, your only study activity will be to start ART earlier than suggested by local guidelines. You will not be taken off ART if the study ends early or at the natural end of the study. Your usual care will continue at the health center, including receiving ART. Your ART will only stop or change if your health care provider decides that it is important to do so for your health.

Persons to Contact for Problems or Questions
If you have any questions about your participation in this research study, your rights as a research subject, or if you feel that you have experienced a research-related injury, contact:

Investigator of Record Name: *(site insert name of the investigator or other study staff)*

Research Site Address(es): *(site insert physical address of above)*

Daytime telephone number(s): *(site insert telephone number)*

24-hour contact number(s): *(site insert telephone number)*

If you have any questions or concerns about your rights as a research subject or want to discuss a problem, get information or offer input, you may contact:

Independent Review Board/Ethics Committee: *(site insert name or title of person on the IRB/EC or other organization appropriate for the site)*

Address of Independent Review Board: *(site insert physical address of above)*

Daytime Telephone Number: *(site insert telephone number of above)*

SUBJECT’S STATEMENT OF CONSENT

Population Effects of Antiretroviral Therapy to Reduce HIV Transmission (PopART): A cluster-randomized trial of the impact of a combination prevention package on population-level HIV incidence in Zambia and South Africa

- I have been given sufficient time to consider whether to take part in this study.
- My taking part in this research study is voluntary. I may decide not to take part or to withdraw from the research study at any time without penalty or loss of benefits or treatment to which I am entitled.
- The research study may be stopped at any time without my consent.
- I have had an opportunity to ask my study investigator questions about this research study. My questions so far have been answered to my satisfaction.
- I have been told how long I may be in the research study.
- I have been informed of the procedures and tests that may be performed during the research study.
- I have been told what the possible risks and benefits are from taking part in this research study. I may not benefit if I take part in this research study.
- I do not give up my legal rights by signing this form.
- I have been told that before any study related procedures being performed, I will be asked to voluntarily sign this Subject Information and Consent Form.
- I will receive a signed and dated copy of this Subject Information and Consent Form.

If you have either read or have heard the information in this Subject Information and Consent Form, if all of your questions have been answered, and if you agree to take part in the study, please print and sign and your name and write the date on the line below.

I voluntarily agree to take part in this research study.

______________________	______________________________
Subject’s Name (print)	Subject’s Signature and Date

I certify that the information provided was given in a language that was understandable to the subject.

______________________	______________________________
Name of Study Staff<br>Conducting Consent Discussion (print)	Study Staff Signature and Date

______________________	______________________________
Witness’ Name (print)<br>(As appropriate) Date	Witness’ Signature and Date

APPENDIX VI - SAMPLE INFORMED CONSENT FORM – CHIP TEAM ACTIVITIES

NOTE: Sample informed consent forms are adapted from NIH templates. It is understood that sites will modify these consents to meet the requirements of their setting and of their ethics committees. Modifications made locally to prior versions of the consents that have already been approved for use in-country are expected to be maintained in subsequent site-specific consent versions.

SUBJECT INFORMATION SHEET

**Title of Research Study:**	**Population Effects of Antiretroviral Therapy to Reduce HIV Transmission (PopART): A cluster-randomized trial of the impact of a combination prevention package on population-level HIV incidence in Zambia and South Africa**
**Protocol #:**	HPTN 071, Version 3.0 16 November 2015<br>DAIDS ID: 11865
**Sponsor:**	National Institute of Allergy and Infectious Diseases<br>National Institute of Mental Health<br>(U.S. National Institutes of Health)<br>Office of the United States Global AIDS Coordinator<br>Bill and Melinda Gates Foundation
**Investigator of Record:**	*(insert name)*
**Research Site Address(es):**	*(insert address)*
**Daytime telephone number(s):**	*(insert number)*
**24-hour contact number(s):**	*(insert number)*

What is the PopART study?

- HIV is still a big problem in Zambia and South Africa
- We now have good treatment (called ART) for people living with HIV which is freely available from health facilities.
- There are various methods that are known to help prevent someone from catching HIV such as using condoms, male circumcision and prevention of mother to child transmission (PMTCT) programs
- Getting people who are HIV positive onto treatment earlier may also help to prevent them from infecting their partners.

- It has been suggested that combining these HIV prevention strategies and offering ART to all people who test HIV positive right away might lead to a big reduction in the number of new HIV infections in the community.

The PopART study will try to answer the above question. Its purpose is to find out if **offering HIV tests in each household** and offering people the chance to **start HIV treatment right away** can reduce HIV infection in a community like yours.

Where and who is conducting this study?

This study is being carried out in two countries, Zambia and South Africa, for a period of about 5 years from 2012 to 2017. It will be done in 21 communities, 12 of which are in Zambia and 9 in South Africa. Researchers from the Zambia AIDS Related Tuberculosis (ZAMBART) Project and the Desmond Tutu TB Centre (DTTC) at Stellenbosch University, South Africa, will work closely together with colleagues from different institutions including the Ministry of Health (Zambia) and the Department of Health (South Africa). This study is being funded by the U.S. National Institutes of Health, the Office of the United States Global AIDS Coordinator, and the Bill and Melinda Gates Foundation.

How is the study being carried out?

- The PopART study has 3 Arms (Arms A, B and C). In each arm a package of HIV prevention services will be available including HIV testing, care and treatment, male circumcision, PMTCT and condoms:
  - In Arm C all of these activities will be available at the health facility. In arms A and B, community HIV workers (called CHiPs) will visit each household and offer HIV testing in the home and help people to link to care services at the health facility or in the community.
  - In all arms of the study, people who are HIV-positive will receive ART from the health facility. In Arms B and C this will be for all people who have a CD4 cell count below the threshold set bylocal guidelines. In Arm A, ALL people who are HIV-positive will be offered ART at any CD4 cell count.
  - In Arms A and B, the CHiP teams will encourage pregnant women who are met during regular household visits, to attend an antenatal clinic in their community.
- The 21 study communities were put in these arms using a process called randomization, which is like a lottery. **Your community was put in <u>Arm [*A/B*]</u>.**

The CHiPs worker is your link to all of these services. If you agree, they will visit your house regularly. They will take down the names and basic information of all household members. This is to ensure that the CHiPs do not miss some members of the household now or in future.

The CHiPs will ask all household members to participate in a health education session in the home. CHiPs will also offer HIV testing to everyone in the home. CHiPs will refer household members to care based upon their health needs. For example, if a person is HIV positive, the CHiP will refer them for care or treatment at the local clinic.CHiPs can also be contacted at any time if you have specific questions or need help with accessing care. Household members may choose not to receive any services recommended by the CHiPs without penalty.

CHiPs will seek permission from each person in the household to ask additional health-related questions and record those answers in an electronic device to help provide better services. This is described more fully below.

How will the researchers find out if the program worked?

To find out if the PopART program works in reducing the number of HIV infections, some people in the community will be asked to take part in some special studies. If you are chosen for one of these studies, you will be asked to choose whether or not to take part in that study. But right now, we are only asking if you will let us collect some health information from you as part of the CHiPs program.

What will happen if I agree to having my health information recorded in the electronic device?

You are being asked for your permission to let the CHiPs ask additional health-related questions and record your health data in an electronic device. For example, we will ask you if you have symptoms of TB or sexually transmitted infections. The additional questions will help us understand your health history better and provide you with better referrals for care. For example if you have symptoms of TB, we will ask you to produce a sputum sample by coughing that can be tested in the laboratory, and if it is positive for TB, we will tell you and refer you to the clinic for care.

Recording your answers in the electronic device will allow us to follow up and make sure you receive care for any referrals we make. The data on the electronic device can only be seen by authorized staff with a secret password. We may follow up by coming back to the house and asking you whether you received care, or we may get this information from the health center. If we get this information from the health center, the information collected will all be kept confidential.

Allowing CHiPs to collect your health information in this way is voluntary and therefore you are completely free to refuse to take part.

CHiP teams will follow national requirements to notify local health authorities when a TB case is identified.

Risks and Benefits

There are unlikely to be additional risks other than those associated with HIV testing, care and treatment.

Both HIV positive and negative individuals will benefit from the linkages to care provided by the CHiPs program. In addition, taking ART reduces the likelihood that HIV will be passed on to a sexual partner or baby.

Persons to Contact for Problems or Questions

If you have any questions about this research study, your rights, or if you feel that you have experienced a research-related injury, contact:

Investigator of Record Name: *(site insert name of the investigator or other study staff)*

Research Site Address(es): *(site insert physical address of above)*

Daytime telephone number(s): *(site insert telephone number)*

24-hour contact number(s): *(site insert telephone number)*

If you have any questions or concerns about your rights or want to discuss a problem, get information or offer input, you may contact:

Independent Review Board/Ethics Committee: *(site insert name or title of person on the IRB/EC or other organization appropriate for the site)*

Address of Independent Review Board: *(site insert physical address of above)*

Daytime Telephone Number: *(site insert telephone number of above)*

Verbal Consent Administered by CHiPs

As you have heard from the information leaflet you have just read/ I have just read to you, I am one of the CHiPs working with the PoPART study in this community. Now I would like to find out if you have understood this information and if you would like to take part in this CHiPs program.

[*CHiP records in a log the decision by the participant(s)*]

Parent or Guardian Verbal Consent for Minors to Participate in the Intervention:
As a parent or guardian, you are being asked if you give your permission for the child in your care to participate in the CHiPs program. The procedures, risks and benefits for your child would be the same as has just been described.

If you do not give permission for your child to participate in the CHiPs program, we will still offer to provide him/her health screening here at the household. However, we would not have information to allow us to check that your child has received the care they need.

Now I will ask you if you have understood this information and whether you consent for your child to participate in the CHiPs program.

Do you give your consent (permission) for your child's participation in the CHiPs program?

APPENDIX VII – SAMPLE SIZE CALCULATIONS

(1) Primary endpoint - HIV incidence over 36 months

N=2500 individuals in population cohort, 85% HIV-negative at baseline, 25% loss to follow-up by 36 months; 5206 person-years per community over 36 months (assuming 1912 person-years 0-12 months; 1700 person-years 12-24 months; 1594 person-years 24-36 months)

(a) Comparison between Arms A or B and Arm C

HIV incidence rate/ 100py (control arm)	Between-cluster coefficient of variation (k)	Effectiveness (%)	Power (%)
1.0	0.15	30%	74%
1.0	0.15	35%	87%
1.0	0.15	40%	95%
1.0	0.15	45%	99%
1.0	0.15	50%	100%
1.0	0.15	55%	100%
1.0	0.15	60%	100%
1.0	0.15	65%	100%
1.0	0.20	30%	60%
1.0	0.20	35%	75%
1.0	0.20	40%	87%
1.0	0.20	45%	94%
1.0	0.20	50%	98%
1.0	0.20	55%	99%
1.0	0.20	60%	100%
1.0	0.20	65%	100%
1.5	0.15	30%	81%
1.5	0.15	35%	92%
1.5	0.15	40%	98%
1.5	0.15	45%	100%
1.5	0.15	50%	100%
1.5	0.15	55%	100%
1.5	0.15	60%	100%
1.5	0.15	65%	100%
1.5	0.20	30%	65%
1.5	0.20	35%	80%
1.5	0.20	40%	91%
1.5	0.20	45%	96%
1.5	0.20	50%	99%
1.5	0.20	55%	100%
1.5	0.20	60%	100%
1.5	0.20	65%	100%

(b) Comparison between Arms A and B

HIV incidence rate/ 100py (control arm)	Between-cluster coefficient of variation (k)	Effectiveness (%) Arm A	Effectiveness (%) Arm B	Power (%)
1.0	0.15	50%	20%	89%
1.0	0.15	50%	25%	78%
1.0	0.15	**55%**	**25%**	**92%**
1.0	0.15	55%	30%	82%
1.0	0.15	**60%**	**25%**	**98%**
1.0	0.15	**60%**	**30%**	**94%**
1.0	0.20	50%	20%	78%
1.0	0.20	50%	25%	65%
1.0	0.20	**55%**	**25%**	**83%**
1.0	0.20	55%	30%	71%
1.0	0.20	**60%**	**25%**	**93%**
1.0	0.20	**60%**	**30%**	**87%**
1.5	0.15	50%	20%	94%
1.5	0.15	50%	25%	86%
1.5	0.15	**55%**	**25%**	**96%**
1.5	0.15	55%	30%	90%
1.5	0.15	**60%**	**25%**	**99%**
1.5	0.15	**60%**	**30%**	**98%**
1.5	0.20	50%	20%	84%
1.5	0.20	50%	25%	72%
1.5	0.20	**55%**	**25%**	**88%**
1.5	0.20	55%	30%	78%
1.5	0.20	**60%**	**25%**	**96%**
1.5	0.20	**60%**	**30%**	**92%**

(2A) HIV incidence during months 12-24 from start of intervention

Number of person-years of follow-up in each community = 1700 during months 12-24 from start of intervention

(a) Comparison between Arms A or B and Arm C

HIV incidence rate/ 100py (control arm)	Between-cluster coefficient of variation (k)	Effectiveness (%)	Power (%)
1.0	0.15	35%	59%
1.0	0.15	40%	72%
1.0	0.15	45%	83%
1.0	0.15	50%	91%
1.0	0.15	55%	96%
1.0	0.15	60%	98%
1.0	0.15	65%	99%
1.0	0.15	70%	100%
1.0	0.20	35%	51%
1.0	0.20	40%	64%
1.0	0.20	45%	76%
1.0	0.20	50%	85%
1.0	0.20	55%	92%
1.0	0.20	60%	96%
1.0	0.20	65%	99%
1.0	0.20	70%	100%
1.5	0.15	35%	71%
1.5	0.15	40%	83%
1.5	0.15	45%	92%
1.5	0.15	50%	97%
1.5	0.15	55%	99%
1.5	0.15	60%	100%
1.5	0.15	65%	100%
1.5	0.15	70%	100%
1.5	0.20	35%	61%
1.5	0.20	40%	74%
1.5	0.20	45%	85%
1.5	0.20	50%	92%
1.5	0.20	55%	97%
1.5	0.20	60%	99%
1.5	0.20	65%	100%
1.5	0.20	70%	100%

(b) Comparison between Arms A and B

HIV incidence rate/ 100py (control arm)	Between-cluster coefficient of variation (k)	Effectiveness (%) Arm A	Effectiveness (%) Arm B	Power (%)
1.0	0.15	60%	25%	77%
1.0	0.15	60%	30%	66%
1.0	0.15	65%	30%	81%
1.0	0.15	65%	35%	71%
1.0	0.15	70%	30%	92%
1.0	0.15	70%	35%	85%
1.0	0.20	60%	25%	71%
1.0	0.20	60%	30%	61%
1.0	0.20	65%	30%	76%
1.0	0.20	65%	35%	66%
1.0	0.20	70%	30%	88%
1.0	0.20	70%	35%	81%
1.5	0.15	60%	25%	89%
1.5	0.15	60%	30%	80%
1.5	0.15	65%	30%	92%
1.5	0.15	65%	35%	84%
1.5	0.15	70%	30%	98%
1.5	0.15	70%	35%	94%
1.5	0.20	60%	25%	82%
1.5	0.20	60%	30%	72%
1.5	0.20	65%	30%	86%
1.5	0.20	65%	35%	78%
1.5	0.20	70%	30%	95%
1.5	0.20	70%	35%	90%

(2B) HIV incidence during months 24-36 from start of intervention

Number of person-years of follow-up in each community = 1594 during months 24-36 from start of intervention

(a) Comparison between Arms A or B and Arm C

HIV incidence rate/ 100py (control arm)	Between-cluster coefficient of variation (k)	Effectiveness (%)	Power (%)
1.0	0.15	35%	57%
1.0	0.15	40%	70%
1.0	0.15	45%	81%
1.0	0.15	50%	89%
1.0	0.15	55%	95%
1.0	0.15	60%	98%
1.0	0.15	65%	99%
1.0	0.15	70%	100%
1.0	0.20	35%	50%
1.0	0.20	40%	62%
1.0	0.20	45%	74%
1.0	0.20	50%	84%
1.0	0.20	55%	91%
1.0	0.20	60%	96%
1.0	0.20	65%	98%
1.0	0.20	70%	99%
1.5	0.15	35%	69%
1.5	0.15	40%	82%
1.5	0.15	45%	91%
1.5	0.15	50%	96%
1.5	0.15	55%	99%
1.5	0.15	60%	100%
1.5	0.15	65%	100%
1.5	0.15	70%	100%
1.5	0.20	35%	60%
1.5	0.20	40%	73%
1.5	0.20	45%	84%
1.5	0.20	50%	92%
1.5	0.20	55%	96%
1.5	0.20	60%	99%
1.5	0.20	65%	100%
1.5	0.20	70%	100%

(b) Comparison between Arms A and B

HIV incidence rate/ 100py (control arm)	Between-cluster coefficient of variation (k)	Effectiveness (%) Arm A	Effectiveness (%) Arm B	Power (%)
1.0	0.15	60%	25%	75%
1.0	0.15	60%	30%	64%
1.0	0.15	65%	30%	79%
1.0	0.15	65%	35%	69%
1.0	0.15	70%	30%	90%
1.0	0.15	70%	35%	84%
1.0	0.20	60%	25%	69%
1.0	0.20	60%	30%	59%
1.0	0.20	65%	30%	74%
1.0	0.20	65%	35%	64%
1.0	0.20	70%	30%	86%
1.0	0.20	70%	35%	79%
1.5	0.15	60%	25%	87%
1.5	0.15	60%	30%	78%
1.5	0.15	65%	30%	90%
1.5	0.15	65%	35%	82%
1.5	0.15	70%	30%	97%
1.5	0.15	70%	35%	93%
1.5	0.20	60%	25%	81%
1.5	0.20	60%	30%	71%
1.5	0.20	65%	30%	85%
1.5	0.20	65%	35%	76%
1.5	0.20	70%	30%	94%
1.5	0.20	70%	35%	89%

(3A) Community viral load 24 months after start of intervention

N=300 HIV-positive individuals in each community

(a) Comparison between Arms A or B and Arm C

Percentage with undetectable viral load (control arm)	Between-cluster coefficient of variation (k)	Percentage with undetectable viral load, Arm A or B	Power (%)
20%	0.15	40%	99%
20%	0.15	60%	99%
20%	0.20	40%	99%
20%	0.20	60%	99%

(b) Comparison between Arms A and B

Between-cluster coefficient of variation (k)	Percentage with undetectable viral load, Arm A	Percentage with undetectable viral load, Arm B	Power (%)
0.15	60%	40%	97%
0.20	60%	40%	85%

(3B) Community viral load 12 and 36 months after start of intervention

N=approximately 75 HIV-positive individuals in each community

(a) Comparison between Arms A or B and Arm C

Percentage with undetectable viral load (control arm)	Between-cluster coefficient of variation (k)	Percentage with undetectable viral load, Arm A or B	Power (%)
20%	0.15	40%	99%
20%	0.15	60%	99%
20%	0.20	40%	97%
20%	0.20	60%	99%

(b) Comparison between Arms A and B

Between-cluster coefficient of variation (k)	Percentage with undetectable viral load, Arm A	Percentage with undetectable viral load, Arm B	Power (%)
0.15	60%	40%	91%

0.20	60%	40%	77%

(4) HSV2 incidence over 36 months

Number of person-years of follow-up in each community = 1837 over 36 months

Comparison between Arms A or B and Arm C

HSV2 incidence rate/ 100py (control arm)	Between-cluster coefficient of variation (k)	HSV2 incidence rate / 100py, Arm A or Arm B	Power (%)
5.0	0.15	3.0	98%
5.0	0.15	7.5	94%
5.0	0.20	3.0	92%
5.0	0.20	7.5	81%

(5) Retention in HIV care, and viral load suppression and drug resistance among HIV-positive individuals who are taking ART – measured among HIV-positive members of the Population Cohort

(i) Retention in HIV care 12 months after registering for HIV care

N=198 in each community in Arm A and B; N=99 in each community in Arm C

This assumes: N=375 HIV-positive individuals per community in the population cohort; that 65% of these individuals are not yet registered at the clinic for HIV care (N=244); that among these 244 individuals, in Arms A and B 90% subsequently register at the clinic for HIV care (N=220) and in Arm C 45% subsequently register at the clinic for HIV care (N=110), and that 10% cannot be included in analysis due to migration out of the community, giving N=198 included in analysis in Arms A and B and N=99 included in analysis in Arm C

Comparison between Arm A or Arm B, with Arm C

Percentage retained in care (control arm)	Between-cluster coefficient of variation (k)	Percentage retained in care, Arm A or Arm B	Power (%)
80%	0.20	70%	71%
80%	0.20	90%	94%
85%	0.20	75%	85%
85%	0.20	95%	99%
90%	0.20	80%	96%
90%	0.20	95%	79%

(ii) Viral load suppression and drug resistance, measured among HIV-positive members of the Population Cohort at 24 months

Calculations assume that, among population cohort members, N=220 HIV-positive individuals per community register for HIV care for the first time in Arms A and B and N=110 in Arm C, as above.

It is further assumed that, by the time of the 24-month follow-up in the population cohort, 67% of such patients will have started ART in Arm C, 50% in Arm B, and 80% in Arm A; and that 80% of such patients will participate in the Population Cohort at 24 months (PC24). This gives N=141, N=88, and N=59 per community in Arms A, B, and C respectively, for viral load and drug resistance measurement at PC24.

(a) Comparison between Arm A and Arm C

Percentage with detectable viral load (control arm)	Between-cluster coefficient of variation (k)	Percentage with detectable viral load, Arm A	Power (%)
5%	0.20	10%	72%
5%	0.20	2.5%	39%
10%	0.20	20%	91%
10%	0.20	15%	45%
10%	0.20	5%	63%

(b) Comparison between Arm B and Arm C

Percentage with detectable viral load (control arm)	Between-cluster coefficient of variation (k)	Percentage with detectable viral load, Arm B	Power (%)
5%	0.20	10%	64%
5%	0.20	2.5%	36%
10%	0.20	20%	86%
10%	0.20	15%	40%
10%	0.20	5%	60%

(6) Prevalence of bacteriologically-confirmed pulmonary tuberculosis 36 months after start of intervention

Calculations assume 4250 adults included in TB prevalence survey in each community

Pulmonary TB prevalence (control arm)	Between-cluster coefficient of variation (k)	Effectiveness (%)	Pulmonary TB prevalence (Arm A, or Arm B)	Power (%)
1.00%	0.25	40%	0.60%	73%
1.00%	0.25	45%	0.55%	84%
1.00%	0.25	50%	0.50%	91%
0.80%	0.25	40%	0.48%	69%
0.80%	0.25	45%	0.44%	81%
0.80%	0.25	50%	0.40%	89%

(7) HIV-free child survival among children born during the 36 months of trial intervention

Calculations assume 229 person-years of follow-up on HIV-free child survival, among children born to HIV-positive mothers, in each community

Comparison between Arm A or Arm B, with Arm C

Rate of child mortality and/or HIV infection per 100py, among children born to HIV-positive mother (control arm)	Between-cluster coefficient of variation (k)	Effectiveness (%)	Power (%)
11	0.15	35%	71%
11	0.15	40%	83%
11	0.15	45%	92%
11	0.15	50%	97%
11	0.20	35%	61%
11	0.20	40%	74%
11	0.20	45%	85%
11	0.20	50%	92%
10	0.15	35%	68%
10	0.15	40%	81%
10	0.15	45%	90%
10	0.15	50%	96%
10	0.20	35%	59%
10	0.20	40%	72%
10	0.20	45%	83%
10	0.20	50%	91%
9	0.15	35%	65%
9	0.15	40%	78%
9	0.15	45%	88%
9	0.15	50%	94%
9	0.20	35%	56%
9	0.20	40%	69%
9	0.20	45%	81%
9	0.20	50%	89%

APPENDIX VIII - PROPOSED POPULATION CROSS-SECTIONAL SURVEY

Because participants in the *Population Cohort* will be followed longitudinally over three years, their interactions with the research staff could bias the data they provide for certain outcome measures. The *Population Cross-Sectional Survey*, if funded, would be a snapshot evaluation to provide unbiased data for comparison on many of the measures evaluated in *the Population Cohort*.

Analyses for process measures, secondary outcome measures, and the Schedule of Study Visits and Procedures planned for the Population Cross-Sectional Survey are provided below.

Statistical Analysis of Process Measures in the *Population Cross-Sectional Survey* at 36 Months (Arms A, B, and C)

With a sample size of 500 adults aged 18-44 in the *Population Cross-Sectional Survey* in each community, estimates will be obtained for each trial arm of (i) the percentage of the adult population who have accessed HIV counseling and testing (HCT) services during the 36 months of trial intervention (ii) the percentage of HIV-infected individuals who have been screened for ART eligibility during the 36 months of trial intervention (iii) the percentage of HIV-infected individuals who are on ART at the time of the cross-sectional survey, and (iv) the percentage of initially uncircumcised men who have had medical male circumcision during the 36 months of trial intervention.

(i) HIV testing uptake

Assuming that the uptake of HIV testing during the past 36 months is 50% in Arm C, compared with 70% in each of Arm A and Arm B, and that k=0.2, there is 73% power to show an effect of the CHiP intervention on testing uptake. With higher testing uptake of 80% in each of Arm A and Arm B, there is 94% power to show an effect of the CHiP intervention.

(ii) Screening for ART eligibility, and uptake of ART, among HIV-infected individuals

On average there will be 75 HIV-infected individuals (15% of 500) included in the *Population Cross-Sectional Survey* in each community. Assuming that 25% were already on ART at the start of the trial, on average 56 will have been ART-naïve at the start of the trial. With the percentage screened for ART eligibility 70% or higher in Arm B, and 40% or lower in Arm C, and k=0.2, study power is at least 95% to show an effect of the trial intervention on the uptake of ART eligibility screening. Similarly, with the percentage started on ART 70% or higher in Arm A, and 40% or lower in Arm C, and k=0.2, study power is at least 95% to show an effect of the trial intervention on ART uptake.

(iii) Male circumcision

On average there will be 250 men in the *Population Cross-Sectional Survey*. In the Western Cape trial communities, approximately 49 will be HIV-uninfected and not circumcised prior to the start of the PopART trial and approximately 185 in the Zambian trial communities, giving a harmonic mean of 91 in each community. With the uptake of medical male circumcision 40% or more in each of Arms A and B, but 25% or less in Arm C, and k=0.2, study power is at least 82% to show an effect of the trial intervention.

Outcomes for Secondary Objectives

- Community viral load (subject to funding)
    - Viral load in approximately 75 HIV-infected individuals per cluster at 36 months in the *Population Cross-Sectional Survey*
- ART adherence and viral suppression
    - HIV viral load at 36 months in HIV-infected members of *the Population Cross-Sectional Survey* who initiated HIV care and ART after commencement of the PopART intervention in the community (subject to funding)
    - Self-reported adherence to ART in HIV infected members of the *Population Cross-Sectional Survey* who initiated HIV care and ART after commencement of the PopART intervention in the community, measured at 36 months
- ARV drug resistance (subject to funding)
    - ARV drug resistance at 36 months in HIV-infected members of the *Population Cross-Sectional Survey* who initiated HIV care and ART after commencement of the PopART intervention in the community, among individuals who are not virally suppressed at 36 months
- HIV disease progression
    - CD4 cell counts, WHO staging events, and retention in care among members of the *Population Cross-Sectional Survey* initiating ART after commencement of the PopART intervention in the community, measured using routine health center data (consent to use linked routine clinical data required)
- ART toxicity
    - ART safety and clinical events among members of the *Population Cross-Sectional Survey* initiating ART after commencement of the PopART intervention in the community, measured using routine health center and laboratory data (consent to use linked clinical data required)

- Sexual risk behavior
    - Self-reported sexual risk behavior at 36 months in the *Population Cross-Sectional Survey*
- HIV-related stigma
    - Answers to questionnaire evaluating stigma at 36 months in the *Population Cross-Sectional Survey*
- Uptake of PMTCT
    - Self-reported use of services for PMTCT among HIV-infected women in the *Population Cross-Sectional Survey* who had been pregnant in the prior 36 months
- Uptake of male circumcision
    - Self-reported circumcision uptake in the prior 36 months of men in the *Population Cross-Sectional Survey*
- ART screening and uptake
    - The proportion of members of the *Population Cross-Sectional Survey* identified as HIV-infected who have been screened for ART eligibility, and who subsequently initiated ART
- HIV testing and retesting
    - Self-report of prior HIV testing at 36 months in the *Population Cross-Sectional Survey*
- Time between HIV diagnosis and initiation of care
    - The proportion of members of the *Population Cross-Sectional Survey* initiating HIV care within 3 months of a positive HIV diagnosis
- Other testing may be performed using stored samples, as noted in the Section 9.

SCHEDULE OF STUDY VISITS AND PROCEDURES: PROPOSED POPULATION CROSS-SECTIONAL SURVEY-ALL ARMS

**PROCEDURES**	**Single Visit at 36 Month Time Point**
**ADMINISTRATIVE, BEHAVIORAL, AND REGULATORY PROCEDURES**	
Obtain informed consent for enrollment.<br>Solicit consent for storage of specimens for future testing and for access to data collected at health centers	X
Obtain locator information.	X
Administer survey to include socio-demographic, health, social, behavioral, and economic factors	X
**CLINICAL/COUNSELING PROCEDURES**	
Perform HIV rapid testing[1]	X
Collect blood for laboratory testing and sample storage	X
Provide HIV pre- and post-test counseling and HIV rapid test results, for those willing to receive results.	X
**LABORATORY PROCEDURES**	
HIV testing[2]	X
Plasma storage[3]	X

<u>Footnotes for the *Population Cross-Sectional Survey*</u>

[1] Rapid testing will be offered at home visits and performed according to in-country guidelines. This testing will not be used to estimate HIV incidence or prevalence; however, the data may be captured along with other data from the home visit. Tie-breaker testing may or may not be performed in the home.

[2] Preliminary testing to assess HIV status will be performed in-country at a centralized laboratory. Additional HIV testing will be performed at the HPTN LC to confirm/determine HIV infection status. Results will not be returned to study sites or participants.

[3] Plasma samples will be stored at in-country centralized laboratories. The study site will ship samples to the HPTN LC on a routine basis, and will ship additional samples as requested by the HPTN LC. Additional information will be provided in the SSP Manual. Information about the use of stored samples is provided in Section 9.

SUMMARY OF PROTOCOL CHANGES

HPTN 071 (PopART)
Summary of Protocol Changes

CM – Clarification Memo
LoA – Letter of Amendment

Document & Version	Date Approved	Summary of Changes
Protocol Version 1.0	26 Oct 2012	Original protocol
CM #1	21 Aug 2013	• Update the protocol team roster<br>• Update the study timelines<br>• Modify Population Cohort exclusion criteria<br>• Clarify laboratory processes, procedures, and timelines<br>• Update statistical tables
CM #2	17 Oct 2013	• Clarify that all HIV infected, pregnant or breastfeeding women are eligible for lifelong ART per government policy in Zambia and the Western Cape of South Africa<br>• Clarify that field staff will not solicit enrollment into the Population Cohort of community members living in the same household as an employee of Zambart or the Desmond Tutu TB Center<br>• Clarify that the age provided in the study information sheet is for consent to HIV testing, rather than the age of consent for participation in the intervention
Protocol Version 2.0	02 June 2015	This protocol amendment includes many revisions to version 1.0 of the protocol, some of which are minor or administrative in nature. The most significant revisions are highlighted in the list below.<br>• Incorporate changes previously approved in Clarification Memos #1 and #2. Such changes are identified below as 'CM#1' or 'CM#2'. Changes first introduced with this amendment are labeled 'Current revision'.<br>• Update the study timeline.<br>• Clarify laboratory nomenclature, processes, procedures and timelines.

Document & Version	Date Approved	Summary of Changes
		• Revise the Human Subjects Considerations section and the sample informed consent form for Arm A participants starting art immediately to address suggestions from the Protection of Participants, Evaluation, and Policy Branch at DAIDS.<br>• Clarify in the CHiPs Information Sheet which activities of the CHiPs intervention are being consented to through individual informed consent.<br>• Allow parents or guardians to consent for the minors in their charge to participate in the intervention activities requiring individual informed consent through the addition of appropriate language in the CHiPs Information Sheet.<br>• Clarify that portions of some samples collected from *Population Cohort (PC)* participants will be used for an ancillary *Phylogenetics Ancillary Study*, if funding for this ancillary study is obtained, if the participant agrees to this use through a separate informed consent process described in the ancillary study protocol, and if uses of the samples for main study objectives are complete.
Protocol Version 3.0	16 Nov 2015	This protocol amendment includes many revisions to version 2.0 of the protocol, some of which are minor or administrative in nature. The principal and most significant revision is the first item in the list below, provision of immediate access to ART.<br>• Describes provision for immediate access to ART in all three study arms, pending funding<br>• Remove reference to potential PMTCT survey due to lack of funding<br>• Version number and dates changed throughout<br>• Updates to protocol team roster
LoA #1	23 Aug 2016	• The protocol is being updated to allow the study team to enroll additional participants into the Population Cohort (PC) at the time of the 24-month survey, if necessary, The prior version of the protocol had already allowed this at the 12-month survey.

Document & Version	Date Approved	Summary of Changes
		• The protocol is being updated to remove the third case-control study.<br>• The title of a sub-section in Section 7 of the protocol is corrected to indicate that a proposed Population Cross-Sectional Survey would take place at the 36-month visit.
LoA #2	06 Dec 2016	• The description of the qualitative studies to be undertaken as part of the protocol has been revised. The sample informed consent for qualitative activities has been modified accordingly and an additional sample informed consent for parental/guardian consent has been added.<br>• The sample informed consent for the Population Cohort (PC) has been revised to reflect the follow-up visit schedule for participants newly enrolling during the first or second round of follow up visits (PC12N and PC24N).<br>• The sample informed consent forms for PC and for the intervention have been modified slightly to reflect the change in local guidelines for initiation of antiretroviral therapy (ART), which has eliminated the differences between the study's two intervention arms.<br>• Administrative revisions were made to the protocol.
LoA #3	06 Jun 2017	• The protocol has been modified to include an Adolescent Cross-Sectional Survey that will collect data from young people in the standard-of-care arm of the study (Arm C).<br>• The protocol has been modified to add two additional secondary objectives measuring the impact of the study intervention on TB outcomes.
LoA #4	27 Sep 2017	• The protocol has been modified to increase the sample size of the Adolescent Cross-Sectional Survey from approximately 1,400 participants to between approximately 1,400 and 2,800 participants.
LoA #5	17 Apr 2018	• To change the assessment period for the primary outcome from PC0–PC36 to PC12–PC36.

STATISTICAL ANALYSIS PLAN VERSION 1.0

14 APR 2016

HPTN 071

Population Effects of Antiretroviral Therapy to Reduce HIV Transmission (PopART): A cluster-randomized trial of the impact of a combination prevention package on population-level HIV incidence in Zambia and South Africa

Statistical Analysis Plan

Deborah Donnell, PhD

Alicia Young, MS

Vaccine and Infectious Disease Division, Fred Hutch, Seattle WA

Sian Floyd, MS

Richard Hayes, DSc

Department of Infectious Disease Epidemiology, London School of Hygiene and Tropical Medicine

Version 1.0, April 14, 2016

Table of Contents

A INTRODUCTION 3
B STUDY OBJECTIVES AND SUMMARY 3
B.1 MODIFICATIONS OF THE PROTOCOL AFFECTING THE STATISTICAL ANALYSIS PLAN 6
C OVERVIEW OF STUDY DESIGN AND RANDOMIZATION SCHEME 7
D ANALYSIS COHORTS OF THE POPULATION COHORT 8
D.1 POPULATION COHORT 8
D.2 HIV UNINFECTED COHORT 8
D.3 HIV-INFECTED COHORT 8
D.4 SELF-REPORTED HIV-INFECTED COHORT 9
E VARIABLE DEFINITIONS 9
F BASELINE TABLES AND STUDY CONDUCT 14
F.1 DESCRIPTION OF TABLES FOR THE PC COHORT 14
F.2 DESCRIPTION OF TABLES FOR THE CHIPS DATA 16
G BLINDING, ACCESS TO DATA AND PUBLICATION DURING THE STUDY 24
H INTERIM MONITORING GUIDELINES 25
H.1 INTERIM MONITORING FOR EFFECT SIZE BASED ON INTERVENTION UPTAKE 25
H.2 INTERIM MONITORING OF HIV INCIDENCE 27
H.3 INTERIM EVALUATION OF VIRAL LOAD SUPPRESSION 27
I STATISTICAL ANALYSES 31
I.1 DESCRIPTIVE ANALYSES 31
I.2 PRIMARY ANALYSIS 32
I.3 INTERIM MONITORING ANALYSIS 35
I.4 SECONDARY OBJECTIVES: POPULATION COHORT 36
I.5 SECONDARY OBJECTIVES: CHIPS DATA 45

A Introduction

This statistical analysis plan (SAP) details the statistical procedures that address the study objectives specified in Protocol version 1.0 of the HPTN071 (PopART) Study: Population Effects of Antiretroviral Therapy to Reduce HIV Transmission (PopART): A cluster-randomized trial of the impact of a combination prevention package on population-level HIV incidence in Zambia and South Africa

New versions of the SAP will be issued to document updates and changes in the plan. Any meaningful changes or additions to this SAP (e.g., in response to protocol amendments or violations of assumptions underlying pre-planned analyses), and the timing of such changes will be documented in Section B.1 and further described in an Appendix of the SAP, as necessary. Analysis plans for sub-studies not addressed in the protocol, and for secondary analyses not anticipated prior to study completion, will be developed as separate documents. Specifically, the following study components are not included in the SAP

1. Mathematical modeling
2. Case-control studies
3. Economic modeling
4. Ancilliary studies

Formal interim monitoring will not be implemented in the study since intervention effects are expected to become stronger with each year of the intervention and reliable assessments of emerging data on the primary endpoint will likely not be available until some months after the completion of each PC round. Plans for interim monitoring of intermediate outcomes are outlined in Section H.

In addition to DSMB reviews, the PopART data team will routinely report on operational metrics (e.g., rates of recruitment and retention, intervention uptake) to the study operations team, which will communicate with sites concerning operational performance based on these metrics. No analysis of HIV incidence or community viral load will be included in these reports, the format and schedule of which are not considered further in this SAP.

B Study Objectives and Summary

This section corresponds to the protocol (Version 1.0).

Purpose: The purpose of this study is to determine the impact of two community-level combination prevention packages, both of which include universal HIV testing and intensified provision of HIV antiretroviral therapy (ART) and care, on population-level HIV incidence.

Design: This is a three-arm, cluster-randomized, longitudinal study to be implemented in 21 clusters (communities).

Study Population: The prevention packages will be implemented throughout the communities randomized to the intervention arms. Main study outcomes will be measured in a randomly-selected group drawn from the adult population of the communities: a *Population Cohort*.

Study Size: The combined population of all 21 clusters is approximately 1.2 million individuals. The interventions will be implemented in 14 of the 21 clusters with a combined population of approximately 800,000 individuals (adults and children) in the intervention arms. The approximate sizes of the randomly-selected groups for main study outcome assessments are:

- *Population Cohort*: 52,500 individuals
- *Case-Control Studies*: 2,400 individuals
- *Qualitative Studies*: about 2,000 individuals
- *Population Cross-Sectional Survey*: 10,500 individuals (if funded)
- *TB Survey*: about 56,000 (Arms A and B) individuals (if funded)
- *CHiPS: about 150,000 households; 300,000 adults.*

Study Arms/Interventions:

Arm A - Universal Testing with Immediate ART:

- Combination prevention package including:
  - House-to-house deployment of:
    - Universal HIV counseling and testing
    - Active linkage to care for individuals diagnosed as HIV-infected, with ***immediate eligibility for ART***
    - Promotion of male circumcision and prevention of mother-to-child transmission (PMTCT) services
    - Provision of condoms
  - Strengthening of HIV testing and services at health facilities and other venues
  - Strengthening of male circumcision and prevention of mother-to-child transmission of HIV services available in the community
  - Treatment of sexually transmitted infections (STIs) and provision of condoms at health units

Arm B - Universal Testing with ART Eligibility According to National Guidelines:

- Combination prevention package including:
  - House-to-house deployment of:
    - Universal HIV counseling and testing
    - Active linkage to care for individuals diagnosed as HIV-infected, with ***ART eligibility according to national guidelines***
    - Promotion of male circumcision and PMTCT services
    - Provision of condoms
  - Strengthening of HIV testing and services at health facilities and other venues
  - Strengthening of male circumcision and PMTCT services available in the community
  - Treatment of STIs and provision of condoms at health units

Arm C - Standard of Care (Control Arm)

  - Strengthening of HIV testing and ART services according to national guidelines at health facilities and other venues
  - Strengthening of male circumcision and PMTCT services available at health facilities and other venues in the community
  - Treatment of STIs and provision of condoms at health facilities and other venues in the community

Study Duration: The planned duration of the entire study will be approximately 6 years, with enrollment and follow-up of communities and delivery of the intervention occurring over 4 years. Assessment of the primary outcome (HIV incidence) in the *Population Cohort* is planned to take place 12, 24, and 36 months after recruitment. Interim evaluation will take place during the first two years of intervention to determine whether to continue with the 36 month follow-up of the *Population Cohort* and the fourth year of intervention.

Primary Objective:

- To measure the impact of the two intervention packages on HIV incidence by enrolling and following a random sample of adults (the *Population Cohort*) in the trial communities for 3 years

Secondary Objectives:

- Measure the impact of the two intervention packages on the following:
    - HIV incidence over the first, second, and third years of follow-up
    - Community viral load (if funding is identified)
    - ART adherence and viral suppression (if funding is identified)
    - ART drug resistance (if funding is identified)
    - HSV-2 incidence
    - Uptake of HIV testing and retesting over the entire study period
    - ART screening and uptake
    - Time between HIV diagnosis and initiation of care
    - Retention in care
    - HIV disease progression and death
    - ART toxicity based on clinic records
    - Sexual risk behavior
    - Case notification rate of tuberculosis
    - HIV-related stigma
    - Uptake of PMTCT
    - Uptake of male circumcision
- Carry out case-control studies to examine factors related to:
    - Uptake of HIV testing during the first round of home-based testing in Arms A and B
    - Uptake of immediate treatment in Arm A
    - Uptake of HIV testing during the second round of home-based testing in Arms A and B
- Use qualitative methods to:
    - Assess popular understanding of HIV testing and treatment at study initiation and during implementation
    - Evaluate the acceptability and functioning of the Community HIV-care Providers (CHiPs) in Arms A & B
    - Evaluate the acceptability of interventions and barriers to access in Arms A & B
    - Document the effect of the interventions on social networks, stigma, sexual behavior, alcohol use, gender-based violence, HIV identity, other HIV prevention options and community morale
    - Evaluate the process and challenges of community consultation and applying ethical principles
- Measure the burden experienced by local health centers due to implementation of the intervention in the community

- Measure the incremental cost of the two intervention packages through systematic recording of costs in intervention and control communities
- Estimate the effectiveness and cost-effectiveness of the intervention packages and alternative packages, both in the chosen study populations and in other populations by fitting mathematical models based on the empirical data from the trial, including data related to cost.

Study Sites: The study is implemented in the communities identified below.

- The study communities in Zambia are spread across 4 provinces and 5 districts. Each community is the catchment population of a government health facility.
    - Chimwemwe and Ndeke in Kitwe District (Copperbelt Province)
    - Chipulukusu and Chifubu in Ndola District (Copperbelt Province)
    - Makululu and Ngungu in Kabwe District (Central Province)
    - Chawama, Chipata and Kanyama in Lusaka District (Lusaka Province)
    - Maramba and Dambwa in Livingstone District (Southern Province)
    - Shampande in Choma District (Southern Province)

- The study communities in South Africa are located in the Cape Metro District and Cape Winelands District of the Western Cape Province. As above, the communities are defined by the catchment population of a government health facility.
    - Delft South (Metro District)
    - Kuyasa (Metro District)
    - Luvuyo (Metro District)
    - Town II (Metro District)
    - Ikhwezi (Metro District)
    - Bloekombos (Metro District)
    - Dalevale (Cape Winelands District)
    - Wellington (Cape Winelands District)
    - Cloetesville (Cape Winelands District)

B.1 Modifications of the Protocol Affecting the Statistical Analysis Plan

B.1.1 Version 3.0:

1. Immediate offer of ART for all HIV infected individuals attending health care facilities will be implemented within all clinics involved in the HPTN071 (PopART) trial. This will be offered ahead of local guidelines once protocol version 3.0 is approved and funding to support the change can be secured.

Change to analysis plan:

The primary analysis for the assessment of the PopART impact on HIV incidence is unchanged, as the implementation of the change did not affect Year 1 of the intervention, and is likely to only partially impact outcomes in Year 2. Then even in Year 3, as for the PopART intervention itself, effects will still take time to accumulate, especially for impact on HIV incidence.

A secondary analysis objective has been added to assess the impact of immediate treatment policies on community HIV incidence. (See Section I.4.15)

C OVERVIEW OF STUDY DESIGN AND RANDOMIZATION SCHEME

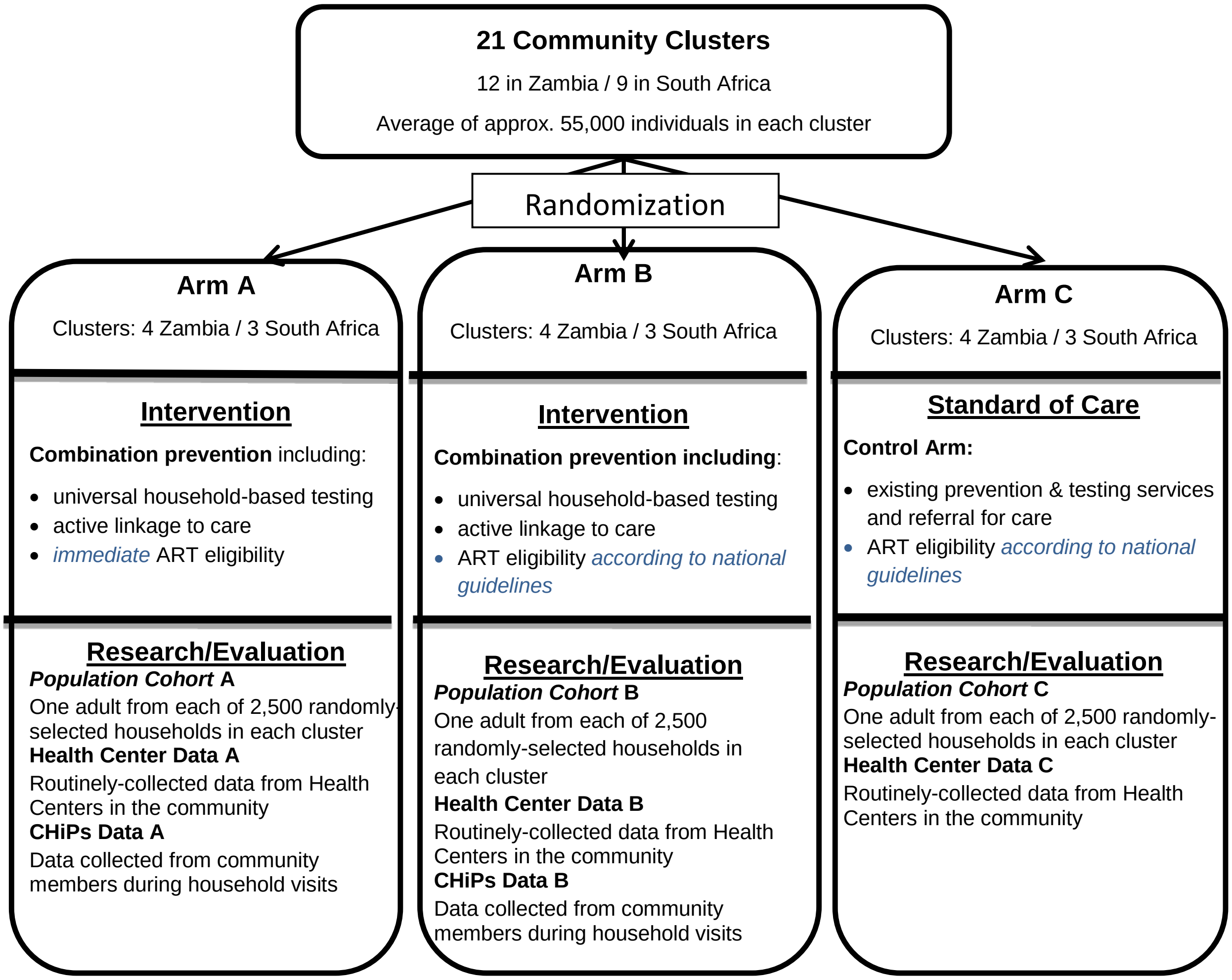

Primary Outcome Measure

•HIV incidence measured over 3 years in ***Population Cohort***

Secondary Outcome Measures

•***Population Cohort***: HIV incidence measured over 1st , 2nd , and 3rd years, HSV-2 incidence, sexual risk behavior+, community VL+*, viral suppression (ART patients)* +, drug resistance (ART patients with detectable VL)* +

•***Population Cohort*** and **Health Center Data:** ART Adherence+, HIV disease progression and death+, ART toxicity+, HIV stigma+

•**Health Center Data:** TB notification and mortality rates

•***Population Cohort*, Health Center Data, CHiPs Data:** uptake of PMTCT+, uptake of male circumcision+, ART screening and uptake+, uptake of HIV testing and retesting+, time between diagnosis and initiation of care+

+ Objectives that will also be addressed by the *Population Cross-Sectional Survey*, if funded
* Pending funding for these assays.

D Analysis Cohorts of the Population Cohort

This section describes the analysis cohorts of the population cohort

D.1 Population Cohort

The population cohort (PC) is all participants enrolled in the PC cohort.

D.1.1 Subgroups of the PC Cohort

An extended group of questions are administered to two subgroups at each PC round. The subgroups are mutually exclusive (so a participant is not asked to complete two extended sets of questions). The subgroups are re-selected each cycle.

- PC0, PC12, PC24 and PC36 Risk subgroups: A 20% randomly selected subgroup is given an extended sexual risk and stigma questionnaire.
- PC0, PC12, PC24 and PC36 Economics subgroups: A 20% randomly selected subgroup is given an extended economic and quality-of-life assessment.

D.2 HIV Uninfected Cohort

The primary endpoint analysis will be conducted using the HIV Uninfected Cohort.

The Primary HIV Uninfected Cohort includes:

- PC participants who were negative at PC0 and/or PC12 and/or PC24 by study testing and who have subsequent HIV test status determined by study testing at PC12 and/or PC24 and/or PC36.
- Determination of HIV status requires completed HIV testing according to the study HIV test algorithm, which generally requires a single, fourth-generation HIV test for HIV-uninfected status and two different fourth-generation tests for HIV-infected status.
- Participants who did not have HIV status determined on at least two different visits are not included in the cohort.

D.2.1 Subgroups of the HIV Uninfected Cohort

HIV incidence cohorts for each year of the PC cohort are defined as follows:

- **PC12**: All PC0 participants who have HIV status HIV-uninfected at PC0, and have HIV status determined in PC12.
- **PC24**: All PC participants who have HIV status HIV-uninfected at PC12, with HIV status determined in PC24.
- **PC36**: All PC0 participants who have HIV status HIV-uninfected at PC24, and have HIV status determined in PC36.

D.3 HIV-Infected Cohort

All PC participants who have HIV-infected status at baseline, PC12, PC24, or PC36. Participant data are only included in the cohort after HIV-infected status occurs.

D.3.1 Subgroups of the HIV-Infected Cohort

- **Seroconverter Cohort**

All PC participants who seroconvert during the HPTN071 protocol. A seroconverter is defined as a participant who has HIV-uninfected status documented at enrollment or during PC cohort follow-up and subsequently has confirmed HIV-infected status.

- **PC0, PC12, PC24, PC36**
  Participants of the HIV-Infected Cohort who are HIV infected at baseline, by study testing, at PC12, PC24, PC36, respectively (each includes those infected at previous visits)
- **Community Viral Load: PC24**
  Viral load is measured in all HIV-infected participants at PC24.
- **Community Viral Load: PC0, PC12, PC36**
  PC0, PC12, PC36: Randomly selected subset of 75 HIV-infected participants in each community in the HIV-infected cohort at PC0, PC12 and PC36. Seroconverters in the PC12 round are excluded from the sampling frame for PC12 cohort in the expedited evaluation of viral load for interim monitoring.

D.4 Self-reported HIV-Infected Cohort

All PC participants who have self-reported they are HIV-infected at PC0, PC12, PC24, or PC36. Participants' data are included in the cohort after they first self-report they are HIV-infected.

D.4.1 Subgroups of the Self-reported HIV-Infected Cohort

- PC0, PC12, PC24, PC36
  Participants of the self-reported HIV-Infected Cohort who first report, or have previously reported, they are HIV-infected at PC0, PC12, PC24, PC36, respectively (each cohort includes those self-reporting HIV infected in previous visits).
- **Newly diagnosed:** all participants who self-report HIV-infected diagnosis after the initiation of PopART.
- **Registered in care since start of PopART:** all HIV-infected participants who did not self-report being registered in care at PC0 who subsequently report being HIV-infected and registered in care.
- **Initiated ART since start of PopART:** All participants who did not self-report current ART treatment at PC0 who subsequently report being HIV-infected and on ART treatment during PC follow-up.

E Variable definitions

Label	Description/Definition	Type	Units/Categories	Use in analysis
**Administrative**				
Household ID	Uniquely identifies each household randomly selected from the sampling frame	Label	8 digit number	Identifier

Participant ID	Uniquely identifies each selected participant	Label	10 digit number	Identifier
Barcode/specimen ID	Uniquely identifies specimens collected for each enrolled participant	Label	10 digit number	Identifier
Enrolled	Selected participant who consented to participate	Binary	Yes/No	Cohort
Triplet	Uniquely identifies each triplet	Label	1-7	Stratification
Randomization arm	Randomization arm for each community	Categorical	A,B,C	Primary covariate
Community number	Identifies community of each participant	Categorical	101-121	Cluster
Visit	Time point for data collection	Categorical	PC0, PC12, PC24, PC36	Study Time
Date of visit	Calendar date of visit	Calendar time	ddMMMyy/PC0, PC12, PC24, PC36	
Duration on study	Duration between HIV tests: e.g. Date of PC12 specimen draw – Date of PC0 specimen draw in years	Duration	Years/ PC12, PC24, PC36	Time on study
**Primary Endpoint**				
Local HIV-1 result	HIV-1 lab result from in-country (local lab) testing, used in calculation of primary endpoint	Categorical	Reactive/Non-reactive PC0, PC12, PC24, PC36	
Central HIV-1 result	HIV-1 lab result from central lab (LC) testing, used in calculation of primary endpoint. All local reactive; 10% local non-reactive.	Categorical	Reactive/Non-reactive PC0, PC12, PC24, PC36	
HIV-1 status	Derived from the local and central HIV-1 results, Uninfected if the local HIV-1 result was non-reactive or if reactive by the local result but determined non-reactive by further testing, Infected if reactive by local testing and confirmed reactive by	Categorical	Infected/Uninfected /Indeterminate PC0, PC12, PC24, PC36	Primary endpoint

	central testing. Or if non-reactive by local testing but confirmed reactive by central testing. Indeterminate if reactive is not confirmed.			
**Demographics/Subgroups**				
Gender	Gender of participant	Categorica l	Male/Female	Subgroups
Age in years, baseline	Age of participant at enrollment: calculated from birthdate and enrollment date, or from age in years if exact birth date was unknown	Numeric	18-44	Subgroups
Marital status, baseline	Marital status	Categorica l	Currently married/living as married,<br>never married,<br>divorced/separated,<br>widowed	Demographic
Education, baseline	Education level	Categorica l	None/Grade 1-2,<br>Grade 3-6,<br>Grade 7-10,<br>Grade 11-12,<br>College/University,<br>Other	Demographic
Sexually active at baseline	Participant report of sexual activity at baseline	Binary	Yes/No	Subgroups
**Secondary Endpoints**				
HSV-2 status	Derived from the local and central HSV-2 results, Uninfected if local HSV-2 result was non-reactive or if reactive by the local result but determined non-reactive by further testing. Infected if reactive by local testing and confirmed reactive by central testing. Indeterminate if reactive/non-reactive is not confirmed.	Categorica l	Infected/Uninfected /Indeterminate.<br>PC0, PC36	Secondary endpoint

Self-reported HIV status	Participant report of HIV status	Categorical	Negative, Positive, Don't Know/Unwilling to disclose<br>PC0, PC12, PC24, PC36	Cohort
Plasma viral load	Number of viral copies per mL in plasma	Numeric or BLQ	Copies/mL or BLQ<br>HIV-infected PC12 (75/community), PC24	Secondary endpoint
Undetectable PVL	Whether PVL is <400 copies/mL	Binary	0/1	Secondary endpoint
Self -reported ART treatment	Participant report of ART treatment uptake, assessed only in those self-reporting HIV-infected status	Binary	Skipped,<br>Yes/No<br>PC0, PC12, PC24, PC36 (HIV-infected)	Secondary endpoint
Registered for HIV care	Participant report of registering for HIV care, assessed only in those self-reporting HIV-infected status	Binary	Skipped,<br>Yes/No<br>PC0, PC12, PC24, PC36 (HIV-infected)	Secondary endpoint
Date of most recent HIV care visit	Participant report of last clinic visit for HIV care	Calendar time	Skipped,<br>MM/YY<br>PC0, PC12, PC24, PC36 (HIV-infected)	Secondary endpoint
Number of sexual partners in prior year	Number of partners in last 12 months	Count	1-999<br>PC0, PC12, PC24, PC36	Secondary endpoint
Number of sex events per month	Participant report of average number of sex events per month, assessed only in the cohort of participants assigned to the extended Risk questionnaire	Count	1-99<br>PC0, PC12, PC24, PC36	Secondary endpoint for Risk subset
How often used condoms	Participant report of condom use, assessed only in the cohort of participants assigned to the extended Risk questionnaire	Categorical	All the time /Sometimes/Never<br>PC0, PC12, PC24, PC36	Secondary endpoint for Risk subset
Exchange of money/drugs/food/shelter for sex	Participant report of exchange of money, drugs, food or shelter for sex, assessed only in the cohort of participants	Categorical	Yes/No<br>PC0, PC12, PC24, PC36	Secondary endpoint for Risk subset

	assigned to the extended Risk questionnaire			
Any casual or one time partners	Participant report of any casual or one time partners	Categorica l	Yes/No PC0, PC12, PC24, PC36	Secondary endpoint
Unprotected sex	Participant report that most recent sex act was unprotected	Categorica l	Yes/No PC0, PC12, PC24, PC36	Secondary endpoint
Concurrent sexual partners	Participant report of concurrent sexual partners, assessed only in the extended Risk questionnaire	Categorica l	Yes/No PC0, PC12, PC24, PC36	Secondary endpoint for Risk subset
Inconsistent condom use	Participant report of any inconsistent condom use, assessed only in the extended Risk questionnaire	Categorica l	Yes/No PC0, PC12, PC24, PC36	Secondary endpoint for Risk subset
HIV discordant relationship	Participant report of any partner with discordant HIV status, assessed only in the extended Risk questionnaire	Categorica l	Yes/No PC0, PC12, PC24, PC36	Secondary endpoint for Risk subset
Stigma score	HIV related stigma score, calculated from 12 questions measured on a 4-point Likert scale, assessed only in the extended Risk questionnaire	Numeric	PC0, PC12, PC24, PC36	Secondary endpoint for Risk subset
Pregnancy during past year	Participant report of pregnancy during the past year	Categorica l	Yes/No PC0, PC12, PC24, PC36 (Female)	Cohort
PMTCT use during pregnancy	Participant report of ART treatment during pregnancy	Categorica l	Yes/No PC0, PC12, PC24, PC36 (Female)	Secondary endpoint
Circumcision in the past year	Participant report of circumcision during the past year	Categorica l	Yes/No PC0, PC12, PC24, PC36 (Male)	Secondary endpoint

F Baseline Tables and Study Conduct

F.1 Description of tables for the PC cohort

This section describes the tables of baseline characteristics of the PC0 cohort. A subset of these will be presented to the DSMB at each meeting. These tables will appear in the open DSMB report. When results are presented by study arm, as this is an unblinded trial, the arms will be labeled Arm A, B and C, corresponding to the trial arms.

F.1.1 Study Accrual

Activation dates, first enrollment dates, target accrual numbers, and the number of participants accrued each month are presented for each country and community.

F.1.2 Baseline Demographic Characteristics

Demographic characteristics summarized include age, gender, education, marital status, employment status, type of employment among those currently employed, nights spent away from home in the last three months, and nights spent outside the community in the last three months. Characteristics are presented by country, community and arm for all participants and for men and women separately by country and community.

F.1.3 Baseline Household Characteristics

Household characteristics summarized include building type, main source of drinking water, and main source of energy used for cooking. Characteristics are summarized by country and arm.

F.1.4 Baseline Risk Characteristics

Risk characteristics summarized include alcohol use, recreational drug use in the last 12 months, sexual activity in the last 12 months, number of sex partners in the last 12 months, partners living outside the community and condom use during the last sex event for all participants. Circumcision status is reported for men. The number of sex partners in the last 12 months is categorized as 0, 1, 2 or 3 or more partners. Characteristics are summarized for all participants by country, community and arm and for men and women separately by country and community.

F.1.5 Baseline Sexual Activity by Age Group

Sexual activity risk characteristics are summarized by age group overall and for men and women separately. Risk characteristics summarized include reported sexual activity in the last 12 months, number of sex partners in the last 12 months, partners living outside the community and condom use during the last sex event.

F.1.6 Baseline HIV Self-Report

Self-reported HIV status and HIV testing history by country, arm and community are summarized for the entire cohort of enrolled participants and for the cohort of those participants who report no prior visits from CHiP teams. Self-reported HIV status is categorized as missing, positive, negative, unknown or unwilling to disclose, and never tested.

F.1.7 Baseline Rapid Test Acceptance

Baseline rapid test acceptance and rapid test results are presented by country, by arm, by arm in Zambia, by arm in South Africa and by community for the entire cohort of enrolled participants and for participants who report no prior visits from CHiP teams. Rapid tests refer to the HIV tests offered to the participant during the PC enrollment visit by the Research Nurse. The percentages of those accepting the rapid test are shown overall, for those self-reporting HIV- infected, for those self-reporting HIV-

negative, and for those never tested. Each rapid test produces a result that is non-reactive, reactive or invalid. Per study procedure, a reactive rapid result is followed by a second rapid test. The "final" rapid result is reported in this table. The result is reported as non-reactive if the first test is non-reactive. The result is reported as reactive if both the first and second rapid tests are reactive. A reactive rapid result followed by an invalid or non-reactive test, or not followed by a repeat test, is considered invalid/inconclusive. If the first rapid test is invalid, then the result is also considered invalid.

F.1.8 Baseline HIV Care and ART Uptake

Baseline HIV care and ART uptake among those self-reporting HIV-infected at baseline are summarized by country, arm and community. The number of self-reported HIV-infected participants who report that a CHiP team has not yet visited the household is shown. Participants who have been visited by a CHiP team and report a recent first positive HIV test may have discovered their HIV status through the study intervention. The year of the first positive HIV test and ever registering for HIV care are shown for all participants self-reporting HIV infected. The year registered for HIV care and ART use are summarized among those reporting registered for HIV care. The primary reason for starting ART, the year starting ART, and current ART use are summarized for those reporting ever using ART. Ever stopping ART in the last 12 months and missing any ART pills in the last seven days are summarized among those reporting current ART use.

F.1.9 Specimen Collection Completeness

The completeness of specimen collection is presented by country, arm and community. Percentages of sample collection for in-country HIV testing, in-country HSV-2 testing, Laboratory Center (LC) testing and storage and in-country storage are summarized. The denominator for these participants includes all enrolled participants. All reactive HIV results by in-country testing will be confirmed at the HPTN Laboratory Center (LC) provided an aliquot for LC testing and storage was collected. Ten percent of the HIV-negative in-country results are selected for QA at the LC. The LC will also perform confirmatory testing for all samples for which the in-country testing did not yield a result. The percentages of the HIV confirmatory testing completeness and results from the LC are summarized.

F.1.10 Baseline HIV-1 and HSV-2 Results

HIV-1 and HSV-2 test results are presented by country, arm and community. HIV status is assessed by a combination of in-country and HPTN Laboratory Center (LC, Johns Hopkins) testing. A first HIV test is performed in-country. If the first test is non-reactive, the HIV test is considered negative (10% of these are retested at the LC for quality assurance). If the first test is reactive or invalid, a second test is conducted at the LC. In the case of discordant or inconclusive testing, the LC conducts further tests to establish HIV status. The percentages of the in-country HIV and HSV-2 test results are shown for those samples that have been tested. No result obtained refers to samples that were to be tested but could not be tested effectively due to machine failure, etc.

F.1.11 Baseline HIV-1 Prevalence

Baseline HIV-1 prevalence is presented for each community and overall. In addition for men and women in each country separately, by age category, and by age category for each of men and women. The denominator for the prevalence calculations is the number with completed determination of HIV-status, including LC HIV confirmatory tests.

F.1.12 Baseline HIV Care and ART Uptake Among HIV+

Baseline HIV care and ART uptake are summarized among those HIV-infected by the in-country lab testing. Ever registered for HIV care is missing if the participant self-reported HIV-infected but did not answer the question, "Have you ever registered for HIV care?" None of those self-reporting something

other than HIV-infected status are considered registered for care. Current ART use is missing if the participant self-reported HIV-infected but did not answer the question, "Have you ever taken any ART?" None of those self-reporting something other than HIV-infected status are considered on ART.

F.2 Description of tables for the CHiPs data

Progress of the intervention on a number of key performance measures on a monthly basis. At each review the progress of the HPTN 071 (PopART) intervention will summarize outcomes for each completed round, and data to date of the current round.

The intervention data tables are ordered so as to follow the "cascade" of care from households being visited, enumeration of all household members, through to HIV care registration and ART initiation, followed by summary tables related to PMTCT, medical male circumcision, and tuberculosis screening and diagnosis and treatment.

Additional information on the time to link to HIV care following referral and the time to start ART following referral, assessed by survival analysis methods that account for lag in assessment of outcomes will be provided.

Clinic data on ART initiation is provided from Arm A trial communities.

Most tables are shown separately for men and women, as well as overall.

The average for each country is calculated in two ways:

1. "Total" row: the denominator is the sum of denominators across all communities in the same country, and similarly for the numerators; the overall percentage is obtained from these 2 values. This gives the same weight to each household or individual, thus communities with a larger population are given more weight.
2. "Average" row: the average of the percentage values from each community. This summary gives the same weight to each community, rather than to each household or individual.

The overall summary by trial arm, within each country, is calculated using method (2), i.e. the average of the percentage values from each community is calculated, separately for Arm A and Arm B.

For completed rounds, households and individuals are included in the analysis each Round if they were first visited by a CHiP team before the end of Round 1. For ongoing rounds, they are included if they were first visited as part of the Round on or before the visit cut date

F.2.1 Households visited, among total households in the community – cumulative summary and by calendar quarter

This table summarizes the number and percentage of households visited by CHiP teams.

- The denominator is the total number of households in the community, estimated from the household census that was conducted in mid-2013.
- The numerator of "visited" households counts all households for which an adult was contacted at home and permission was given for the household to be listed on the electronic household register that is maintained by CHiP teams.
- The summary for each calendar quarter includes in the numerator all households visited up to the end of that quarter.

F.2.2 Households enumerated, among visited households – cumulative summary, and by calendar quarter

- This table summarizes the number and percentage of households that consented to the CHiP teams explaining the intervention and also to listing all household members (adults and children) on the electronic register that is maintained by CHiP teams.
- The cumulative summary includes in the denominator all households that were first visited by CHiP teams on or before the end of the round.
- The summary for each calendar quarter includes in the denominator all households that were first visited during this calendar quarter, and the numerator includes all households that consented to household enumeration on or before the end of the round.

F.2.3 Age distribution of the enumerated adult population, separately for men and women

- This table summarizes the number and percentage of adults according to each of 5 age groups, separately for men and women. All individuals who have been listed on the electronic register as a household member, from the start of the intervention up to the end of the round and were 18 or more years old on the date they were first enumerated, are included.

F.2.4 Individual participation in the intervention, cumulative summary

- This table summarizes the number and percentage of adults that consented to participate in the CHiPs intervention, the number and percentage of adults who refused to participate, the number and percentage of adults who had not yet been contacted by CHiP teams, and the number and percentage of adults contacted by CHiP teams but whose consent was "pending" because they wished to take more time to decide if they will participate or not, all as of the end of the round.
- An "adult" includes any individual who was aged 18 or more years old on the date they were first enumerated as a household member as part of the CHiP team visit to their household during the annual round of household visits.
- The denominator includes all adults who were enumerated as a household member from the start of the intervention up to the end of the round.
- The numerator for "adults who consent to participate" includes all adults who *ever* consented to participate by the end of the round. It includes adults who consented but subsequently withdrew their consent to participate (of whom there are few). It also includes adults who initially refused to participate, but subsequently chose to participate.
- The numerator for "adults who refuse to participate" includes all adults who were offered participation in the intervention, but initially refused and did not subsequently change their mind to choose to participate by the end of the round.
- The numerator for "adults not contacted" includes those who were enumerated but were not engaged by the CHiPs during the annual household visit to accept or decline participation, as of the end of the round.
- The numerator for "pending consent" includes those who were contacted by CHiPs on or prior to the end of the round

F.2.5 Knowledge of HIV status, from a prior HIV-positive test result or acceptance of the offer of HIV testing by CHiP teams, cumulative summary

- The first column of this table summarizes the number and percentage of adults who know their HIV status. An adult is defined here as "knowing their HIV status" if they either report they know

they are HIV-positive from a prior HIV-positive test result, or they accept the offer of HIV testing by CHiP teams.

- The denominator for the first column of this table, on “knows HIV status”, includes all adults who were enumerated as a household member and who also consented to participate in the intervention. Thus the denominator for this table, is the same as the *numerator* for “adults who consent to participate” in Table x.4. The numerator counts adults who either self-reported they were HIV-positive or they accepted the offer of HIV testing from CHiP teams, among all adults in the denominator.
- The information collected on knowledge of HIV status and acceptance of the offer of HIV testing by CHiP teams, among adults who consent to participate in the intervention, is held on the “annual round” electronic register that is maintained by CHiP teams.

F.2.6 Knowledge of HIV status, from a prior HIV-positive test result or acceptance of the offer of HIV testing by CHiP teams, or self-report of a recent HIV-negative test result, cumulative summary

- The first column of Table 1(2).5c summarizes the number and percentage of adults who know their HIV status, with the same definition of “know HIV status” as used in Tables x.5, 5a i
- The second to fourth columns of Table 5c summarize the number and percentage of adults who know their HIV status, including adults as “knowing their HIV status” if they report they have recently tested for HIV and the test result was HIV-negative. The second, third, and fourth columns include in the numerator those individuals who report a prior HIV-negative test result from a test within 3, 6, and 12 months respectively of the date of the annual round household visit.

F.2.7 Adults who are “known” to be HIV-positive following the annual household visit, by age group, cumulative summary

- Table 6a summarizes the number and percentage of men who are known to be HIV-positive following the annual household visit, disaggregated by age group. The denominator includes all men who consented to participate in the intervention, and the numerator counts all men who either self-reported they were HIV-positive or they accepted HCT from the CHiP teams and the test result was HIV-positive.
- Table 6b provides the equivalent summary for women.

F.2.8 HIV diagnosis and history of HIV care and treatment, among adults known to be HIV-positive following the annual household visit, cumulative summary

- The first column in this table summarizes the number and percentage of adults who were known to be HIV-positive following the CHiP team’s visit to their household during the annual round of household visits, among all adults who consented to participate in the intervention.
- Adults are “known to be HIV-positive” if they self-reported they were HIV-positive or they accepted the offer of HIV testing from CHiP teams and the test result was HIV-positive.
- The denominator for column 1 includes all adults who consented to participate in the intervention during the annual round. The numerator counts all adults who self-reported they were HIV-positive or accepted the offer of HIV testing from CHiPs teams and the test result was HIV-positive, among adults in the denominator (and so is the sum of the numerators from columns 2 and 4 of Table 5 (or 5a and 5b for men and women respectively)).
- The table then summarizes the prior experience of HIV diagnosis, care, and treatment among individuals known to be HIV-positive, i.e. the denominator for columns 2 to 6 is all adults who were known to be HIV-positive following the annual household visit.

F.2.9 Referral to HIV care, among adults known to be HIV-positive following the annual household visit, overall and according to prior history of HIV diagnosis and care and treatment, cumulative summary

- The first column in this table summarizes the number and percentage of adults who were given a referral to HIV care, and/or to be screened for eligibility to start ART, by CHiP teams.
- The denominator for this column includes all adults who had consented to participate in the intervention and who either self-reported they were HIV-positive or were newly diagnosed as HIV-positive in testing by the CHiP teams. Thus the denominator for this table, is the same as the *numerator* for "adults who are known to be HIV-positive" in Table 7.

F.2.10 Linkage to HIV care, and ART initiation, by 3 months after referral to HIV care, overall and separately for men and women, cumulative summary

- This table summarizes the number and percentage of adults who were linked to HIV care within 3 months of being referred by CHiP teams, and the number and percentage who started ART within 3 months of being referred by CHiP teams, among adults who were referred for HIV care and/or to be screened for eligibility to start ART by CHiP teams.
- The denominator for "linkage to HIV care" includes all individuals who consented to participate in the intervention on or before four months before the end of the round, who either self-reported they were HIV-positive or were newly diagnosed as HIV-positive in testing by the CHiP teams, but excluding individuals who reported that they were taking ART (in the last 1 month) on the date they were given a referral to HIV care by a CHiP team. The denominator is further restricted to individuals who were first given a referral to HIV care by CHiP teams on or before four months before the end of the round.
- Thus the denominator includes all adults who were included in the *numerator* for column 1 of Table 8 except for individuals who reported they were taking ART at the time of the annual round visit, but then restricted to those who were given a referral to HIV care before a given time point.
- The rationale for restricting the analysis to individuals referred on or before four months before the end of the round is because this is 4 months prior to the latest date for which data are available within the round: this then allows us to measure linkage to care / ART initiation 3 months after referral (target metric) and an additional month to allow time for a subsequent follow-up visit by a CHiP team.
- The denominator for "starting ART within 3 months of referral" is the same as for "linkage to HIV care".
- Adults are included in the denominator irrespective of whether they have received none, one or more follow-up visits from a CHiP team.
- Adults can only be counted in the numerator of "linked to HIV care" or "started ART" if they have received a follow-up visit from a CHiP team within the annual round, thus the estimates of the percentage linked to care and/or started ART are conservative, i.e. they are "minimum" estimates.
- Adults are counted in the numerator of "adults seen for HIV care at the clinic within 3 months of CHiPs referral" if any one of the following is true based on information collected in the electronic register that is maintained by CHiP teams: they reported they have attended the clinic for HIV care after referral by a CHIP team and the date of attendance is within 90 days of the date of referral; they reported they are newly registered for HIV care and the date of HIV care registration is within 90 days after the date of referral; they reported they are newly registered for HIV care and the date of the follow-up visit on which this was reported is within 90 days after the date of referral; they reported they have started ART and the date of starting ART is within 90 days after the date of

referral; they reported they have started ART and the date of the follow-up visit on which this was reported is within 90 days after the date of referral.

- Adults are counted in the numerator of “adults starting ART within 3 months of CHiPs referral” if they were given a referral to HIV care by a CHiP team and any one of the following is true based on information collected in the follow-up electronic register that is maintained by CHiPs teams: (1) they reported they have started ART and the date of first starting ART is within 90 days after the date of referral; or (2) they reported they have “ever” taken ART, the date they reported this is within 90 days after the date of referral, and at the time of the annual round visit they reported they had never previously taken ART; or (3) they reported they have “ever” taken ART, but were not currently taking ART, at the time of the annual round visit, and on a follow-up visit they reported they had not missed any pills in the last 3 days (Round 1) or had taken ART in the previous 1 month (subsequent Rounds) and the date of the follow-up visit on which this was reported is within 90 days after the date of referral. For (3), the requirement of not missing pills in the last 3 days for Round 1 is because the “follow-up” electronic register for Round 1 did not include a direct question on “are you currently taking ART”.
- The number and percentage of HIV-positive adults who were linked to HIV care, and/or started ART, is summarized overall, and also separately for men and women.

F.2.11 Linkage to HIV care, by time since referral to HIV care, from a “survival” analysis, cumulative summary

INT Table x.11: ART initiation, by time since referral to HIV care, from a “survival” analysis, cumulative summary

- The estimates of linkage to HIV care, and ART initiation, by 3 months after CHiPs referral that are summarized in Table 9 are conservative because they include in the denominator individuals who have not received a follow-up visit by a CHiP team. Also, they do not provide information on the time to link to HIV care / start ART other than for the single time point of 3 months after referral. And, they are necessarily restricted to referrals made on or before four months before the end of the round.
- Tables 10 and 11 summarize “survival” analyses (using a Kaplan-Meier analysis) of the time for adults to link to HIV care and/or start ART following a referral by a CHiP team. The denominator for these analyses includes adults who were referred to HIV care on or before two months before the end of the round. This allows more adults to be included in the analysis than in the summaries for Table 9 but still allows at least 2 months after the referral was given for a CHiP team to make a follow-up visit.
- The rules for being counted in the numerator are the same as for Table 9.
- The percentages linked to HIV care / starting ART are calculated based on the number of adults who are linked to care / start ART and the person-years of follow-up. The numbers shown in the table are respectively the number with the outcome (n), the number in the denominator at the start of follow-up i.e. the number who were given a referral by CHiP teams and can be included in the analysis according to the criteria specified above (N), and the *percentage with the outcome based on the survival analysis* (which means that the percentages in the tables do not equate to n/ N, which they do in all other tables).
- The percentage of adults linked to HIV care / starting ART are shown for the time points 1 month, 2 months, 3 months, 6 months, 9 months, and 12 months after the date they were referred to the clinic by CHiP teams for the Round.

- The number and percentage of HIV-positive adults who were linked to HIV care / started ART is summarized overall, not also separately for men and women.

F.2.12 Uptake of HIV care and treatment, at the time of the annual household visit, and by the end of the round (Round 1) or by March 31 2016 (Round 2), cumulative summary

- The first indicator on this table summarizes the number and percentage of adults who were known to be HIV-positive following the CHiP team visit to their household during the annual round of household visits, among all adults who consented to participate in the intervention. It provides the same summary as column 1 of Table 7.
- Among individuals known to be HIV-positive, the table then summarizes whether they were in HIV care at the time of the first CHiP team visit to their household in the annual round of household visits. The numerator for "in HIV care" includes all adults who reported they had previously registered for HIV care, except for adults who reported the date of their last attendance at the clinic for HIV care and this date was more than 12 months prior to the annual round visit date.
- The table next summarizes the number and percentage of adults who were taking ART at the time of the CHiP team visit to their household in the annual round of household visits. The numerator for "taking ART" includes all adults who at the time of the annual round visit reported they had taken ART during the last one month.
- For Round 1, the table next summarizes the number and percentage of adults who are assumed still to be alive and resident in the community as of the end of the round, among all adults identified as HIV-positive following the first annual round visit. The numerator of adults "alive and resident in the community as of the end of Round 1" excludes adults who are reported to have died, moved to a different zone within the community, or moved out of the community between the time of the annual round visit and the end of the round. Adults who have died or moved between the time of the annual round visit and the end of the round, but for whom this information was not captured at a follow-up visit during the Round, are counted in the numerator and so the numerator is an over-estimate.
- The table then summarizes the number and percentage of adults who were in HIV care at the end of the round. Adults are included in the numerator if they were in HIV care at the time of the last visit by a CHiP team within the annual round, i.e. at the time of the last follow-up visit for adults who received at least one follow-up visit from a CHiP team after the annual round visit, and otherwise the annual round visit.
- Lastly, the table summarizes the number and percentage of adults who were taking ART at the end of the round. Adults are included in the numerator if they were taking ART at the time of the most recent visit by a CHiP team. For each Round, this required that at the time of the last follow-up visit within Round 1 they reported they had not missed any pills in the last 3 days, or no follow-up visit was made during Round 1 but the individual reported taking ART in the last 1 month at the time of the first annual round household visit.
- The criterion to be included in the numerator of "on ART" in Round 1 was different for the annual round visit and the follow-up visits because the electronic follow-up register for Round 1 did not record whether ART was taken during the last 1 month but instead asked about adherence to treatment during the previous 3 days. For future Rounds, information on whether ART was taken during the previous 1 month was collected in the follow-up register.

- For the summaries on “currently in HIV care” and “currently taking ART”, the percentages may be under-estimates because some adults will have registered for HIV care and/or started ART after their last visit from the CHiP team but before the end of the round.

F.2.13 Knowledge of HIV status among women who reported they were pregnant at the time of the annual household visit, cumulative summary

- This table first summarizes the number and percentage of women who were, or were not, pregnant at the time of the annual round visit to their household. The denominator includes all women who consented to participate in the CHiPs intervention. This information is held on the electronic register that is maintained by CHiP teams.
- Among women who reported they were pregnant at the time of the annual household visit, the table then summarizes the number and percentage who know their HIV status. Women are counted in the numerator if they “know their HIV status” according to the definition used in Table 5b (either self-reported they were HIV-positive, or they accepted the offer of HIV testing by CHiP teams) and also if, as part of the annual household visit, they reported that they had tested for HIV within the previous 90 days and the result of the HIV test was HIV-negative.

F.2.14 Male circumcision status at the time of the annual household visit, and referral for MMC among uncircumcised men who accepted the offer of HIV testing from CHiP teams and the test result was HIV-negative, and uptake of MMC in each Round among men who were not circumcised at the time of Round 1, cumulative summary

- Table 14 first summarizes the number and percentage of men who reported that they were, or were not, circumcised at the time of the annual round visit to their household. The denominator includes all men who consented to participate in the CHiPs intervention. Men who reported they did not know if they were circumcised or not, are counted as not circumcised in this table. The information on circumcision status is held on the electronic register that is maintained by CHiP teams.
- Among men who reported that they were not circumcised (or did not know if they were circumcised), the table then summarizes the number and percentage who accepted the offer of HIV testing from CHiP teams and tested HIV-negative, the number and percentage who self-reported they were HIV-positive or they tested HIV-positive in testing by CHiPs teams, and the number and percentage who did not self-report they were HIV-positive and also did not accept the offer of HIV testing from CHiP teams and so their HIV status is unknown. Lastly, the table summarizes the number and percentage of uncircumcised, HIV-negative men who accepted from the CHiP team a referral for medical male circumcision.

F.2.15 Adults on TB treatment, symptom screening for TB among adults not on TB treatment, and sputum collection from adults with TB symptoms at the time of the annual household visit; subsequent TB diagnosis and treatment initiation, cumulative summary

- The first column of this table summarizes the number and percentage of adults who self-reported they were currently on TB treatment at the time of the annual household visit, among all adults who consented to participate in the intervention.
- The second column of the table then summarizes the number and percentage of adults who were screened for TB symptoms (cough for 2 or more weeks, night sweats, and weight loss) and/or asked if a household member was currently taking TB treatment. The denominator includes all adults who consented to participate in the intervention except for adults who self-reported they were currently

on TB treatment (adults who self-report they are currently on TB treatment are not screened for TB symptoms). The numerator includes all adults who were screened for TB symptoms and/or asked if a household member was currently taking TB treatment.

- The third column of the table then summarizes the number and percentage of adults who reported TB symptoms, among all who were screened for TB symptoms.
- The fourth column of the table then summarizes the number and percentage who gave one or two sputum samples to the CHiP team (to be taken to the clinic for TB diagnosis), among those with TB symptoms.
- The fifth column summarizes the number and percentage who were known to have received a test result for TB from a sputum sample, among all adults with TB symptoms at the time of the annual round. The denominator includes adults who did not give a sputum sample to a CHiP team, because all adults with TB symptoms were referred to the clinic for TB diagnosis, so they could also provide a sputum sample later at the clinic.
- The sixth column summarizes the number and percentage who were known to have started TB treatment (based on self-reported information), among those who received a positive test result for TB.

F.2.16 Linkage to HIV care, by time since referral to HIV care, from a "survival" analysis, cumulative summary

- This table summarises the same analysis as in 1.10, but extended to include information from subsequent follow-up visits and rounds.
- The analysis is pooled across all trial communities, within each country, and then disaggregated by calendar quarter in which the individual was first referred to HIV care.

F.2.17 ART initiation in Arm A communities, by time since referral to HIV care, from a "survival" analysis, cumulative summary

- This table summarises the same analysis as in 1.11, but restricted to Arm A communities and extended to include information from subsequent follow-up visits and.
- The analysis is pooled across all trial communities, within each country, and then disaggregated by calendar quarter in which the individual was first referred to HIV care.

F.2.18 Retention on ART among adults who (re-) consented to participate and have reported at least once that they have ever taken ART, cumulative summary

- This table summarises retention on ART, among HIV-positive adults who (re-) consented to participate, and who have reported they have "ever" taken ART at one or more of the rounds to date annual household visits or a follow-up visit made at any time in subsequent annual household visits.
- Columns 1-3 of the table summarise the number and percentage of individuals who, in subsequent annual household visits, reported they were currently taking ART and had not missed any pills in the previous 3 days. The denominator is all adults who re consented to participate in subsequent Rounds, and have reported they have "ever" taken ART at any annual household visits or a follow-up visit.
- Columns 4 and 5 summarise estimates of retention on ART 6 months after starting ART, among adults who first started ART between 1 January 2014 (the beginning of trial interventions) and up to 8 months before the data extract date, and have consented to participate in the Round 2 annual household visit.

- Column 4 summarises the number and percentage for whom it is unknown if they were retained on ART at 6 months after starting ART, because there is no information from a follow-up visit during the time window 5-18 months after starting ART.
- Column 5 summarises retention on ART at 6 months after starting ART, among those with information in the time window 5-18 months after starting ART. Among those with information in the time window, information from the time point closest in time to the 6-month time point is used. If this information is from an annual round visit, or a follow-up visit, then an individual is considered "retained on ART" if they reported they had taken ART within the last 1 month. If the information is from a follow-up visit made during Round 1, when information on "are you currently taking ART" was not collected, then an individual is considered "retained on ART" if they reported that they missed no pills in the previous 3 days.

F.2.19 Uptake of ART in Arm A Clinics in Zambia, cumulative summary

- This table summarizes the number of patients seen in the Arm A clinics in Zambia, since the initiation of the study, who were HIV-positive and were assessed by the clinical officer at the clinic (denominator, indicator 1) on or before March 31 2016.
- Indicator 1 then provides the number and percentage of those assessed who were found not to be eligible for ART per national guidelines, e.g. who had a high CD4 cell count (numerator, indicator 1).
- Indicator 2 then provides the number and percentage of those at each Arm A clinic who were not eligible to start ART under national guidelines (denominator) who consented to start ART "early" through the HPTN 071 study (numerator).
- Indicator 3 then provides the number and percentage of those who consented to start ART "early" through HPTN 071 (denominator) who actually did start early (numerator) according to the clinical log book.

F.2.20 Uptake of ART in Arm A Clinics in South Africa, cumulative summary

- This table summarizes the number of clinic patients started on ART at the Arm A clinics since the beginning of the intervention, as of and including March 31 2016. The total numbers are provided and a breakdown by whether the patients were inside or outside of national guidelines (those outside of national guidelines needed to provide written consent to start). The percentage inside/outside of guidelines is also given. The table provides data for each clinic and in total across all three clinics.
- Data recording systems in the Department of Health (DOH) clinics in South Africa are different from those in Zambia. The primary difference is that in South Africa the Arm A consent log sheet is completed retrospectively and implementation was not designed to capture additional indicators including 'the proportion of those outside of national guidelines who are offered and accept 'early' ART initiation'. Introduction of additional parallel data systems into DOH clinics needs to be discussed with DOH. Discussions are ongoing regard introduction of mechanisms to capture these additional data. Therefore the metrics provided at the current time for South African clinic data are those that can be obtained from routine data collection at all three Western Cape clinics and are different than those provided for Zambia.

G Blinding, Access to Data and Publication during the Study

Neither site staff nor participants will be blind to community assignment. It is study policy not to report aggregate data by community name, or have information about community arm assignment in public

documents. And although only the DSMB and statisticians preparing analysis reports will review accumulating interim data by study arm, study sponsors, monitors and non-site staff are likely to become aware of community assignments while the study is ongoing. To the extent possible, however, decisions regarding additions or modifications to planned analyses, determination of inclusion in or exclusion from analysis cohorts or datasets, and rules for handling missing or incomplete data will be made by statisticians, study leadership or other study staff who do not have access to outcome data grouped by arm. Lab assessments will be done blinded to community treatment assignment and the Endpoints Committee will not be provided community assignments when adjudicating HIV endpoint data. The level of blinding maintained when performing these activities will be described in the study report or manuscripts, as relevant.

No data on comparisons between the intervention arms (A and B), nor between the intervention arms (A and B) and the standard of care arm (C), will be presented or published during the study.

The study team will publish and present data from the PC0 (baseline) data during the study. PC0 data will also be accessed by the mathematical modeling group to inform and validate the model.

H Interim Monitoring Guidelines

H.1 Interim monitoring for Effect Size based on Intervention Uptake

The uptake of intervention components will be reported using both data from the CHiPs (available only in arms A and B) and the population cohort (primarily through self-report). Targets have been set for each of the intervention components for CHiPs for Arms A and B. However for interim monitoring, a range of plausible intervention effect sizes will be assessed through mathematical models that allow for the combined effect of the prevention cascade.

H.1.1 Protocol effect size

In the study design, mathematical models used the targets detailed in the Protocol (V1.0). These modelling results are included here for reference.

Table 1- Parameter values assumed for the model of the impact of the intervention for central and optimistic target scenarios, and projected impact on HIV incidence in Arms A and B compared with Arm C, assuming intervention roll-out over a 6-month time period

Parameter		Central Target		Optimistic Target	
Annual coverage of test and treat campaign		70%		75%	
Treatment failure & drop-out rate, per year		10%		10%	
Effectiveness of ART in blocking transmission		90%		95%	
Take up of male circumcision when offered		50%		50%	
		Arm A	Arm B	Arm A	Arm B
Zambia	Impact on cumulative incidence (3 years)	58%	25%	66%	29%
	Impact on cumulative incidence (2 first years)	54%	23%	62%	27%
	Impact on HIV incidence during Year 1	45%	19%	53%	23%
	Impact on HIV incidence during Year 2	63%	28%	72%	33%
	Impact on HIV incidence during Year 3	68%	31%	76%	36%

South Africa	Impact on cumulative incidence (3 years)	57%	23%	65%	27%
	Impact on cumulative incidence (2 first years)	52%	21%	61%	26%
	Impact on HIV incidence during Year 1	44%	18%	52%	23%
	Impact on HIV incidence during Year 2	62%	26%	71%	31%
	Impact on HIV incidence during Year 3	66%	29%	75%	33%

Table 2- Parameter values assumed for the model of the impact of the intervention for central and optimistic target scenarios, and projected impact on HIV incidence in Arms A and B compared with Arm C, assuming intervention roll-out over a 12-month time period

Parameter		Central Target		Optimistic Target	
Annual coverage of test and treat campaign		70%		75%	
Treatment failure & drop-out rate, per year		10%		10%	
Effectiveness of ART in blocking transmission		90%		95%	
Take up of male circumcision when offered		50%		50%	
		**Arm A**	**Arm B**	**Arm A**	**Arm B**
Zambia	Impact on cumulative incidence (3 years)	54%	23%	62%	27%
	Impact on cumulative incidence (2 first years)	47%	20%	56%	25%
	Impact on HIV incidence during Year 1	34%	14%	42%	19%
	Impact on HIV incidence during Year 2	61%	27%	70%	32%
	Impact on HIV incidence during Year 3	67%	31%	76%	36%
South Africa	Impact on cumulative incidence (3 years)	52%	21%	61%	26%
	Impact on cumulative incidence (2 first years)	46%	19%	54%	23%
	Impact on HIV incidence during Year 1	33%	14%	41%	18%
	Impact on HIV incidence during Year 2	60%	26%	69%	30%
	Impact on HIV incidence during Year 3	66%	29%	75%	33%

H.1.2 Interim modelling of effect size.

The complexity of the multiple components of the intervention makes it difficult to estimate the population impact on HIV incidence. Operational futility in the study will use a mathematical model to assess the likely range of effect sizes, based on the intervention uptake data collected in the trial. The description of the mathematical model and the scenarios for uptake are detailed in a separate Modeling Projection Report. This will be used to estimate whether the power of the trial remains high.

The Study Team proposes that if the power for the comparison of Arms A and C under scenarios consistent with the observed intervention uptake becomes less than 50%, given data on HIV incidence in Arm C and HIV prevalence in the PC cohort, the DSMB could recommend early stopping of the study for futility, i.e. when the trial is unlikely to have the power to demonstrate effectiveness.

Under current assumptions of 1% incidence and 22% HIV prevalence, 50% power corresponds to a PopART intervention community effectiveness of approximately 25%, i.e. a recommendation to stop the trial for futility if study power was estimated to be "only" 50% would correspond to stopping the trial if the effectiveness of Arm A relative to Arm C was projected to be smaller than 25%. At each DSMB

meeting, updated projections of intervention effect and power based on updated assumptions and protocol changes will be provided in a separate Modeling Projections Report.

H.2 Interim monitoring of HIV incidence

The study was powered assuming an HIV incidence rate of 1.0-1.5 per 100 person years, and an HIV prevalence of 15% in the population cohort of 2,500 per community, so 85% of the enrolled participants are HIV uninfected and are followed to measure HIV incidence. The between-community coefficient of variation of HIV incidence was assumed to be 0.15-0.20. The Protocol (v1.0) indicated that the study will be very well powered to detect an effect of 35% or larger in Arm A compared with Arm C, and moderately well powered to detect an effect of 30% under favorable assumptions. For the direct comparison of Arms A and B, the study will be well powered to detect a difference between effects of 60% and 30%, 55% and 25%, and 50% and 20%. The power calculations assume losses to follow-up of 20% over two years, and 25% over three years.

During the trial, power estimates will be updated based on information available from the PC0 and the modelled projections of effect size. These details will be provided in separate Modelling Projections document that will be updated for each DSMB review when information changes.

Expected information that will require updating of power assumptions:

- Prevalence: The prevalence in PC0 will be available to assess the prevalence assumptions shortly after PC0 is complete.
- Between community variance of HIV incidence: the between-community variance of HIV prevalence is expected to be a good approximation to check this assumption. This will be available shortly after PC0 is complete.
- Arm C incidence: HIV incidence from the PC12 cohort can be used to assess the incidence assumption and assess the power assumptions of the study.
- Loss to follow-up: 20% over two years, and 25% over three years
- Effectiveness: Interim assessment of effectiveness by comparison of emerging data on HIV incidence is not planned. Because full intervention scale up will not be achieved prior to PC12, HIV incidence comparisons at PC12 are unlikely to reflect a significant change. The HIV incidence from PC24 will be completed too late in the study to be useful as an interim assessment. Instead, the modelling group will estimate potential effectiveness using data from intervention uptake and PC0.

H.3 Interim Evaluation of Viral Load Suppression

The PopART test-and-treat intervention relies for its effectiveness on diagnosing a large proportion of HIV-infected individuals in the community, linking them to care, starting them on ART as soon as possible and maintaining high levels of retention, adherence and viral suppression. All the steps in this cascade are monitored continuously during the delivery of the intervention, primarily using process data. We will also monitor viral suppression in the *Population Cohort* across all study arms at PC12, where viral suppression at PC12 may provide an early warning of any major deficiencies in delivery so that corrective action could be taken at an early stage.

H.3.1 Expedited PC12 viral load testing and assessment of community viral load

Expedited PC12 viral load testing in the Population Cohort will be used as a monitoring tool to inform corrective action in the HPTN 071 (PopART) trial. At the PC12 visit, the majority of HIV-infected patients

starting on ART since the start of the intervention in Arms A and B (including those starting outside national guidelines) will have been on ART for 3-12 months, allowing for the time needed for testing and linkage to take place. Data on viral suppression among these patients will provide an early indication of treatment adequacy. Carrying out the 12 month viral load testing, expedited to inform interim monitoring, will allow early corrective action to be taken if needed, while not requiring the addition of any further testing as it is already planned as a secondary trial outcome,.

The main assessment of viral load suppression will occur at the PC24 follow-up when most patients will have been on ART for 1-2 years. For this reason, the trial protocol sets this time-point as the most relevant for assessing viral suppression and will conduct viral testing in *all* HIV-infected participants. The larger sample size at 24 months will provide greater power and precision. Expediting PC24 month viral load testing for more immediate availability of results is not proposed, since the complete results are unlikely to be available with enough time to deploy corrective action. For example, if viral load results are available within 3 months of the last PC24 specimen being collected, the final round of follow-up (PC36) will already be underway before any corrective action could be taken. However, if the PC12 survey were to indicate reasons for concern, expedited testing at 24 month could be reconsidered.

Note that as specified in the Expedited PC12 viral load testing plan, the protocol team leadership will have full access to the VL data set among the subset of participants who self-reported being on ART at PC0 or PC12.

H.3.2 Biological marker of ARVs

A biomarker for ARVs may be used to define “on ART”. An affordable and efficient assay that detects an array of ARV drugs is available at the LC and we propose using this in combination with self-report if resources are available. Assessments of the correspondence between self-report and detection of ARV in samples will be used to judge the accuracy, and thus the use, of self report in analysis. Funding for this assessment is not yet secured, however options for use of this biomarker are included in the SAP.

H.3.3 PC12 VLS Cohort

A random sample of 100 participants from the PC0 HIV-infected cohort will be selected per community to achieve a target sample size for expedited testing at PC12 of 75 per community, allowing loss to follow-up between PC0 and PC12, inadequate samples, processing errors and other deficiencies.

Analysis cohorts:

1. ***PC0 HIV-infected***
   All sampled P0 HIV-infected participants with VL results. This larger cohort will have greater precision for overall rate of VLS
2. ***PC0 HIV-infected and PC12 self-reported HIV-infected on ARVs at PC0 or PC12 visit.***
   Option 1 (Self-report only): The subset of sampled PC0 HIV-infected participants who self-report current ARV use in PC0 and/or PC12. This cohort will be used to assess ART adherence.
   Option 2 (self report or biomarker): The subset of sampled PC0 HIV-infected participants who either have ARV’s detected in their plasma sample or self-report current ARV use in PC0 and/or PC12.

H.3.4 Blinding to interim viral load results

While the study team will remain blinded to overall community viral load, they will have access to the PC 12 VL data on those on ART, in order that corrective action can be taken if necessary.

H.3.5 Power for comparisons in PC12 expedited VLS

The table shows the expected sample size for viral load testing at each visit in Arm A, by country. It also shows the expected precision of the estimated proportion of patients virally suppressed, assuming an underlying rate of 90%. The table assumes that the main interest is in testing for viral suppression among patients who have been on ART at any time during the follow-up period, including those already on ART at baseline (assumed to be around 25% of HIV-infected on average) as well as those started on ART following the start of the intervention (assumed to be 70% of HIV-infected not already on ART at baseline).

The available sample size will provide a precise measure of PC12 viral suppression overall and for Zambia and S Africa separately. It will also give an early indication of gross under-performance in any one cluster. For example, if viral suppression in one cluster is as low as 60%, the precision on this estimate will be ± 12.6% so we would be able to say reliably that this cluster is not reaching a target of (say) 75% or 80%. The corresponding estimates are provided for Arms B and C. Because numbers on ART in each cluster will be somewhat lower than in Arm A, the precision of the viral suppression estimates will be correspondingly lower. However, we consider that these estimates are sufficiently precise for the purposes of this monitoring exercise.

Estimated sample size and precision for PC12 VLS

**Arm A**			
**Visit**	**HIV positive tested for VL**	**Number on ART[1]**	**Precision of proportion virally suppressed[2]**
12m			
Per triplet	75	58	± 7.7%
Total (7 triplets)	525	406	± 2.9%
Zambia (4 triplets)	300	232	± 3.9%
S Africa (3 triplets)	225	174	± 4.5%
**Arm B**			
12m			
Per triplet	75	44	±8.9 %
Total (7 triplets)	525	308	±3.4 %
Zambia (4 triplets)	300	176	±4.4 %
S Africa (3 triplets)	225	132	±5.1 %
**Arm C**			
12m			
Per triplet	75	36	±9.8 %
Total (7 triplets)	525	252	±3.7 %
Zambia (4 triplets)	300	144	±4.9 %
S Africa (3 triplets)	225	108	±5.7 %

[1] Assumes 25% already on ART at baseline and 70% of those not on ART are diagnosed and started on ART

[2] 95% CI for proportion virally suppressed among those on ART, assuming estimated proportion is 90%. These CI do not account for clustering due to the community randomized design.

H.3.6 Ethical issues and anonymization

The protocol states that results from viral load testing in the *Population Cohort* will not be fed back to participants (e.g. if failure of viral suppression is found). Doing so would provide a different level of service for these PC patients and could distort the findings of the trial, leading to Hawthorne effects in the *Population Cohort*. Also in most cases laboratory results will only be available after a long delay, diminishing the clinical relevance of these results. Local health authorities and implementing partners could still be informed of community trends which could facilitate efforts to explore and implement changes at the community service level, in order to benefit all patients in a given community.

Specimens could be anonymized (specimen identification (ID) number de-linked from PC ID number) before going forward for viral load testing. This would remove the ethical dilemma that would otherwise exist. However, delinking these expedited viral load data from other data on *Population Cohort* participants would preclude further statistical analysis of trends in viral load over time and related socio-demographic and other risk factors, and so we are not proposing this at this time.

I Statistical Analyses

This section describes the statistical analyses of the primary and secondary outcomes that will be conducted on enrolled participants in the population cohort.

I.1 Descriptive Analyses

I.1.1 By community

Within each community, summary statistics (e.g., frequencies, percentages, means, medians, inter-quartile range, minima and maxima) that are appropriate to the measurement scale will be used to describe demographic, behavioral, HIV testing and medical history and ART use data. Continuous variables may be described using categorical levels chosen based on previous experience with similar studies; depending on the final distributions of these variables, and providing it does not meaningfully impact the intent of analyses based on them, these categories may be modified prior to final report to allow better characterization of the relevant distributions.

I.1.2 By arm

Different descriptive summaries will be computed as appropriate to the analysis questions.

1. Participant aggregated: Summary statistics are computed treating the participants as a single group, i.e., for the jth community (*j = 1,2,3*) in the ith triplet (I = 1…7), with N_{ij} individuals (*k = 1… N_{ij}*), where we assume that j indexes the communities in arms A (*j = 1*), B (*j = 2*) and C (*j = 3*) respectively

   $$\bar{y}_i = \frac{1}{\sum_j N_{ij}} \sum_{jk} y_{ijk}$$

   Standard errors and confidence limits will not be provided for participant aggregated summaries.

2. Community aggregated: Summary statistics are computed by summarizing the within community summaries by arm. E.g.

   $$\bar{y}_i = \frac{1}{7}\sum_j \bar{y}_{ij} = \frac{1}{7}\sum_j \frac{1}{N_{ij}} \sum_k y_{ijk}$$

   Standard errors and confidence limits for community aggregate estimates will be computed as using the within group samples variance of cluster means

   $$S_B^2 = \frac{\sum_{j=1}^{7} (\bar{y}_i - \bar{y}_.)^2}{6}$$

Note: Participant and community aggregated summaries are expected to be similar when the number of people in each community (sub) population are similar. By the design of the study it is intended that the overall number of people in each community is similar.

3. Descriptive matched differences across arms: Summary statistics are computed within each community, and appropriate comparative statistics are computed within matched pairs and summarized across the 7 pairs. The computation is repeated for each pair of arms.
   - Difference in means:

For each triplet and arms A (i = 1) and C (i = 3), for example: compute the ith triplet specific difference in means: $d_i^{AC} = \bar{y}_{i1} - \bar{y}_{i3}$; the average difference in means between arms is

$$D^{AC} = \sum_{i=1}^{7} d_i$$

- Risk ratio
  For each triplet/pair and arms A and C, for example: compute the triplet specific relative risk ratio: $r_i^{AC} = \frac{p_{i1}}{p_{13}}$. The summary statistic is the geometric mean of the relative risks:
  $$R^{AC} = \exp(\bar{s}^{AC}) = \exp\left(\sum s_i^{AC} / 7\right)$$
  where $s_i^{AC} = \log(r_i^{AC})$
- Rate ratio
  For each triplet/pair: compute the triplet specific rate ratio: $r_i^{AC} = \frac{r_{i1}}{r_{13}}$ where $\bar{r}_{ij} = \frac{1}{\sum_k t_{ijk}} \sum_k y_{ijk}$ with t_{ijk} = time on study and y_{ijk} = 1 if event is observed, 0 otherwise. The summary statistic is the geometric mean of the relative risks:
  $$R^{AC} = \exp(\bar{s}^{AC}) = \exp\left(\sum s_i^{AC} / 7\right)$$
  where $s_j^{AC} = \log(r_i^{AC})$
- Odds ratios

  For each triplet and arms A and C, for example: compute the triplet specific odds ratio $r_i^{AC} = \frac{p_{i1}(1-p_{i3})}{p_{i3}(1-p_{i1})}$. The summary statistic is the geometric mean of the relative risks:
  $$R^{AC} = \exp(\bar{s}^{AC}) = \exp\left(\sum s_i^{AC} / 7\right)$$
  where $s_i^{AC} = \log(r_i^{AC})$

I.2 Primary Analysis

I.2.1 HIV Incidence

Question: Does the PopART intervention reduce risk of HIV acquisition?
Endpoint: HIV-1 Infection
Cohort: HIV-uninfected Cohort

Details: HIV-1 infection as measured by seroconversion will be assessed approximately annually as each PC round is conducted and will be used as the primary endpoint for the intervention effect. Individuals who drop out of the study and refuse further testing prior to completion of follow-up and individuals who die prior to completion of follow-up will be treated as uninformatively censored as of their last valid HIV test. Visits are only included if HIV status was assessed by study HIV testing.

Person years of follow-up are calculated as:

1. The time between the PC0 visit and the final visit (PC12, PC24 or PC36) for participants who are HIV negative at their last visit.

2. The time between the PC12 visit and the final visit (PC24 or PC36) for participants who did not have HIV status determined at PC0 and are HIV negative at their last visit.
3. The time between the PC24 visit and the final PC36 visit for participants who did not have HIV status determined at PC0 or PC12 and are HIV negative at PC36.
4. The time between PC0 and the estimated seroconversion date for participants who seroconverted. Estimated seroconversion date is defined as the date halfway between the last negative HIV test result and the first HIV infected test result.
5. The time between P12 and the estimated seroconversion date for participants who seroconverted and were not tested at PC0.
6. The time between P24 and the estimated seroconversion date for participants who seroconverted and were not tested at PC0 or PC12.

Seroconverters are participants in the cohort who were HIV uninfected status by study testing at PC0, or PC12 or PC24 and who subsequently are HIV infected by study testing at PC12, PC24 or PC36.

Descriptive analysis: The number of events and total person years, HIV incidence rates and 95% confidence intervals will be presented for each community, with 95% confidence intervals based on the Poisson distribution. The same data will also be presented for each community for men and women separately; by age categories; by age for men and women.

The by arm summary will be computed by combining the incidence data for each community with a log transformation applied to reduce skewness. The geometric mean and associated 95% CI will be calculated for each of the three trial arms, and also separately for each arm by country.

Analysis: A two-stage analysis will be used. In the first stage, we estimate the expected number of events in each triplet and arm for all three arms simultaneously. In the second stage, we conduct a formal statistical comparison for the primary comparison between Arm A and C. A two-sided test with α = .05 will be used. Arm B versus C and Arm A versus B will also be compared, using a two-sided test with α = .05. Analyses will not be weighted to account for the sampling design (i.e. selection of one person per household). No adjustment is planned for multiple comparisons.

Details for two-stage analysis of incidence outcomes.

To compute incidence, the number of seroconversions in each community is divided by the person-years of follow-up for that community.

Stage 1:

1. Poisson regression will be used to adjust for confounding variables at the individual level. The model will be fitted using data from all three arms, prior to comparisons for specific arms under comparison. The regression model will include terms for the covariates of interest and triplet but not trial arm.

$$\log(\mathrm{l}_{ijk}) = \alpha_j + \sum_l \gamma_l \, z_{ijkl}$$

where l_{ijk} is the indicator of incidence for the kth individual (k = 1…N_{ij}) in the ith study triplet (i = 1…7) and j^{th} study arm (j = 1,2,3), and where z_{ijkl} is l^{th} covariate.

Individual level covariates to be used for adjustment are:

a) Age in groups (18-24, 25-29, 30-34, 35-39, 40-44)

b) Sex
c) Age x Sex interaction

Note: No adjustment is currently planned for community level covariates. Inclusion of an adjustment for HIV prevalence will be considered if unexpected level of imbalance in HIV-prevalence or community viral load within triplets is observed at baseline. If one of these covariates is included, the degrees of freedom in the paired t-test will be 7-2.

2. A fitted model will be used to obtain the ratio of observed-to-expected (O/E) events, and a log transformation will be applied to this ratio. The expected number of events in the *j*[th] community of the *i*[th] triplet is calculated as:

$$\hat{e}_{ij} = \sum_k t_{ijk}\hat{\mathrm{l}}_{ijk} = \sum_k t_{ijk} \times \exp(\hat{\alpha}_j + \sum_l \hat{\gamma}_l\, z_{ijkl})$$

Where t_{ijk} is the study time in person years for the individual.

3. The adjusted rate ratio for the *i*[th] triplet for arms j_1 and j_0 is calculated as:

$$\theta_i = \frac{d_{i_{j_1}}/\hat{e}_{ij_1}}{d_{i_{j_0}}/\hat{e}_{ij_0 j}}$$

where d_{ij} is the observed number of events.

Stage 2:

1. Take the logarithm of the rate ratio:

$$h_i = \log(\theta_i)$$

The point estimate of the intervention effect comparing arms j_1 and j_0 is the geometric mean of the rate ratios $R_{j_1 j_0} = \exp(\bar{h}) = \exp\left(\sum h_i/_7\right)$

2. Carry out paired t-test on pairwise differences, where s_m^2 is the empirical variance of those differences.

$$t_m = \frac{\bar{h}}{\sqrt{\frac{s_m^2}{7}}}$$

$$s_m^2 = \frac{1}{7-1}\sum_i (h_i - \bar{h})^2$$

The p-value of t_m is assessed assuming a *t* distribution statistic with 6 degrees of freedom and will be used to assess the strength of evidence against the null hypothesis

3. Confidence interval for the log rate ratio are computed as:

$$\bar{h} \pm t_{6,0.025} \times \frac{s_m}{\sqrt{7}}$$

I.2.2 Subgroup analysis

The analysis of HIV risk will be conducted for the following subgroups:

1. Men and women
2. Younger and older (<25, >25 yo)
3. Younger men, Older men, Younger women, Older women

The analysis approach will follow the primary analysis of HIV risk

I.2.3 Sensitivity analysis

Evidence for the intervention effectiveness will also be assessed using a non-parametric permutation test. The analysis above will be recomputed for all possible randomizations that were computed at the beginning of the trial under the restricted randomization scheme. The number of allocations for which the incidence rate ratio is as extreme as the value observed in the trial will be counted and a 2-sided p-value will be calculated as n divided by the total number of possible allocations.

I.3 Interim Monitoring Analysis

I.3.1 Viral Load Suppression

Question: For HIV-infected participants on ART, is the proportion virally suppressed lower in Arms A and B compared to C?

Question: For HIV-infected participants is the proportion virally suppressed high, and is it higher in Arm A and B compared to C?

Endpoint: HIV plasma viral load is suppressed (PVL < 400 copies/mL)
Cohort:

1. Random subset of P12 selected from the P0 HIV-infected Cohort
2. Subset restricted to HIV-infected on ART treatment by self-report (or lab-based assay, if funding permits)

Descriptive analysis: For each cohort, the number in the cohort, the number and proportion virally suppressed will be reported for each community, and for each arm (community-aggregated), with 95% confidence limits for community aggregated summaries as previously defined.The characteristics of (log) viral load in each community will be presented: mean, median, SE, range

The by arm summary will be computed by combining the individual proportion or mean for each community with a log transformation applied to the plasma viral load values to reduce skewness. The geometric mean and associated 95% CI will be calculated for each of the three trial arms, and also separately for each arm by country.

Analysis: The statistical analysis for both questions compares the proportion virally suppressed using an unadjusted cluster-based analysis of the relevant cohort selected for evaluation.

1. The prevalence ratio for each triplet is calculated as:

$$r_i = \frac{p_{ij_1}}{p_{i_{j0}}}$$

where p_{ij} is the observed prevalence of events in the ith triplet for arms j_1 and j_0.

2. Take the logarithm of the prevalence ratios:

$$s_i = \log(r_i)$$

The point estimate of the intervention effect comparing arms j_1 and j_0 is the ratio of the geometric means of the prevalence ratio $R_{j_1 j_0} = \exp(\bar{s}) = \exp\left(\sum s_i / 7\right)$

3. Compute a confidence interval for the average log prevalence ratio:

$$\bar{s} \pm t_{6,0.025} \times \frac{s_m}{\sqrt{7}}$$

where s_m^2 is the empirical variance of the pairwise (log) differences

$$s_m^2 = \frac{1}{7-1}\sum_i (s_i - \bar{s})^2$$

I.4 Secondary Objectives: Population Cohort

I.4.1 Community Viral Load

Question: Does the PopART intervention reduce community viral load?

Endpoint: HIV plasma viral load is suppressed (PVL < 400 copies/mL)
Cohort: PC24 HIV-infected Cohort

Descriptive analysis: The number in the cohort, and the number and proportion virally suppressed will be reported for each community, and for each arm (participant-aggregated), with 95% confidence limits based on the binomial distribution. The characteristics of (log) viral load in each community will be presented: mean, median, SE, range.

The by arm summary will be computed by combining the proportion or mean for each community with a log transformation applied to reduce skewness. The geometric mean and associated 95% CI will be calculated for each of the three trial arms, and also separately for each arm by country.

Analysis: A two-stage analysis will be used. In the first stage, we estimate the expected proportion with viral suppression in each triplet and arm for all three arms simultaneously. In the second stage, we conduct a formal statistical comparison for the primary comparison between Arm A and C. A two-sided 95% confidence limits will be used to assess the evidence for the null hypothesis. 95% confidence limits will also be presented for Arm B versus C and Arm A versus B.

Details for two-stage analysis of proportions

The proportion virally suppressed in each community is the proportion in the cohort with PVL < 400.

Stage 1:

1. Logistic regression will be used to adjust for confounding variables at the individual level. The regression model will include terms for the covariates of interest and triplet but not trial arm.

$$\text{logit}(\pi_{ijk}) = \alpha_j + \sum_l \gamma_l z_{ijkl}$$

where y_{ijk} is the binary indicator of suppressed viral load, *j* is j^{th} study arm (*j* = 1,2,3), *i* is i^{th} study triplet (i = 1…7), *k* is k^{th} individual (*k* = 1…N_{ij}) and z_{ijkl} is the covariate value for the lth covariate. Individual level covariates to be used for adjustment are:

a) Age in groups (18-24, 25-29, 30-34, 35-39, 40-44)
b) Sex
c) Age x Sex interaction

Note: No adjustment is currently planned for community level covariates. Inclusion of an adjustment for viral suppression amongst those HIV-infected at PC0 will be considered if unexpected level of imbalance in HIV-prevalence within triplets is observed at baseline. If this covariate is included, the degrees of freedom in the paired t-test will be 7-2.

2. A fitted model will be used to obtain the ratio of observed (Q) to expected (P) proportions, and a log transformation will be applied to this relative risk. The expected proportion in the ith triplet of the jth community is calculated as:

$$p_{ij} = \sum_k \hat{\pi}_{ijk} = \sum_k \frac{\exp\left(\hat{\alpha}_j + \sum_l \hat{\gamma}_l z_{ijkl}\right)}{1 + \exp(\hat{\alpha}_j + \sum_l \hat{\gamma}_l z_{ijkl})}$$

3. The ratio-residual for the ith triplet of the jth community is calculated as:

$$R_{ij} = \frac{q_{ij}}{p_{ij}}$$

where q_{ij} is the observed proportion of events in the ith triplet of the jth community . The adjusted relative risk for each triplet for arms j_1 and j_0 is calculated as:

$$\rho_i = \frac{R_{ij_1}}{R_{ij_0}}$$

Stage 2:

4. Take the logarithm of the ratio residuals:

$$d_i = \log(\rho_i)$$

The point estimate of the intervention effect comparing arms j_1 and j_0 is the ratio of the geometric means of the ratio residuals $R_{j_1 j_0} = \exp(\bar{d}) = \exp\left(\sum d_i / 7\right)$

5. Carry out paired t-test on pairwise (log) differences, where s_m^2 is the empirical variance of those differences.

$$t_m = \frac{\bar{d}}{\sqrt{\frac{s_m^2}{7}}}$$

$$s_m^2 = \frac{1}{7-1}\sum_i (d_i - \bar{d})^2$$

6. The computed value of t_m is calculated compared to a t distribution statistic with 6 degrees of freedom.

7. The confidence interval for the average relative proportion is computed as:

$$\bar{d} \pm t_{6,0.025} \times \frac{s_m}{\sqrt{7}}$$

I.4.1.1 Subgroup analyses

The analysis of proportion virally suppressed in the community will be conducted for the following subgroups (for each subgroup, two-stage analysis would no longer be adjusted for that subgroup):

a. Men and women

b. Younger and older (<25, $\geq$25 yo)

c. Younger men, Older men, Younger women, Older women

I.4.2 ART Adherence and Resistance

Question: Does the PopART intervention affect the probability of adherence amongst people who self-report being currently on ART?

Endpoint: Viral load suppressed (<400 copies/mL)

Cohort: Community viral load subset PC24: The subset of visits from the PC24 community viral load cohort, restricted to specimens collected from participants who self-report current ART treatment at 24 months.

Analysis: The analysis approach will be the same as used for the two-stage analysis of proportions (see Community Viral Load objective)

Question: Does the PopART intervention affect the probability of ART resistance amongst people who self-report being currently on ART?

Endpoint: Resistance to drugs used in current first line ART treatment

Cohort: Community viral load subset PC24:

Details: Visits from the PC24 community viral load cohort will be assessed for ART resistance, restricted to participants who are self-report current ART treatment at 24 months and are not virally suppressed.

Analysis: The analysis approach will use the two stage approach for proportions.

Question: Does the PopART intervention affect the probability of ART resistance amongst PC cohort seroconverters?

Endpoint: Resistance to drugs used in current first and second line ART treatment

Cohort: Seroconverter cohort

Analysis: The analysis approach will use the two stage approach for proportions.

I.4.3 HSV2 Incidence

Question: Does the PopART intervention change HSV-2 incidence?

Cohort: PC cohort, restricted to the subset of participants who are not infected with HSV-2 at PC0.

Endpoint: HSV-2 status at PC36

Analysis: As HSV-2 acquisition is only assessed once, at PC36, acquiring HSV2 will be assessed as a binary outcome, rather than as a survival analysis outcome. The analysis approach will use the two-stage analysis for proportions (see the analysis of community viral load). HSV-2 acquisition is considered to be both a marker of sexual activity and a risk factor for HIV acquisition.

Individual covariates included in adjustment:

a. Age in groups (18-24, 25-29, 30-34, 35-39, 40-44)
b. Sex
c. Interaction of Sex and Age

I.4.4 HIV Testing

Question: How much did PopART increase recent knowledge of HIV-Status (within the past year) through HIV testing and retesting in each arm, in each year of the study.

Cohort: PC cohort

Endpoint: Self-reported knowledge of HIV status by recent testing.

Details: All enrolled participants will be included. Self-report of HIV status and recent testing is recorded in PC0, PC12, PC24 and PC36. A known a priori caveat for this analysis is that all participants in the PC are offered testing by the research enumerators at each PC visit, thus uptake of testing may be higher in this group that the general population, particularly in Arm C.

PopART is expected to decrease the proportion of persons with "HIV status unknown vs known". Knowledge of HIV status has four mutually exclusive categories. The binary endpoint for assessing the PopART effect is Known vs Unknown

- Known; Self-reported HIV-infected
- Known; Self-reported HIV uninfected, testing in past year
- Unknown (refused to answer; has never been tested)
- Unknown; Self-reported HIV uninfected, no testing in past year

Descriptive: Number and proportion for knowledge of HIV status will be reported over time in each community and for each arm (participant-aggregated), with 95% confidence limits based on the binomial distribution.

Analysis: Estimates of the relative decrease within a pair of study arms will use the two stage analysis method described for proportions (see Community Viral Load) for each study round.

Question: How much did PopART increase accurate knowledge of HIV-infected status through HIV testing and retesting in each arm, in each year of the study?

Cohort: PC cohort. For each year, only participants assessed as HIV-infected status by study testing that year are included

Endpoint: Accurate knowledge of HIV-infected status.

Details: All enrolled participants who are assessed as HIV-infected included.

Knowledge of true HIV-infected status has 3 mutually exclusive categories

- ?/+: Unknown (refused to answer; has not been tested); HIV-infected by study testing
- +/+: Self reported HIV-infected; HIV-infected by study testing
- -/+: Self-reported HIV uninfected; HIV-infected by study testing

The binary endpoint for assessing the PopART intervention is Accurately known(+/+) and Not accurate or not known (remaining categories). PopART is expected to increase the proportion of persons with "Accurate knowledge of HIV status".

Descriptive: Number and proportions in each of the 3 categories and classified as Accurately known will be reported for each year in each community and for each arm (participant-aggregated), with 95% confidence limits based on the binomial distribution.

Analysis: Estimates of the relative increase within a pair of study arms will use the two stage analysis method described for proportions (see Community Viral Load) for each study round separately.

I.4.5 Retention in care

Question: Does the PopART intervention increase retention in HIV care in each community?

Cohort: Self-reported HIV-infected cohort, restricted to participants who report having registered for care

Endpoint: HIV care visit within the past three months

PopART is expected to increase the proportion of persons retained in care (i.e. with HIV care visit within the past 3 months).

Descriptive: Number and proportion will be reported for each year in each community and for each arm (participant-aggregated), with 95% confidence limits based on the binomial distribution.

Analysis: Estimates of the relative increase within a pair of study arms will use the two stage analysis method described for proportions for each study round separately.

I.4.6 Viral Load Suppression

Question: Does the PopART intervention increase viral load suppression in each community at PC12 and PC36?

Cohort: PC12 and PC36 community viral load

Endpoint: Plasma Viral Load

Details: Viral load is assessed in a random sample of 75 HIV-infected participants in each of PC0, PC12, and PC36. See also Interim Analysis for PC12.

Descriptive: Number and proportion virally suppressed will be presented in each community and each year. The characteristics of (log) viral load in each community will be presented: mean, median, SE, range.

Analysis: A paired t-test on the unadjusted pairwise rate ratios for each year will be used to test for change in proportion virally suppressed in each pair of arms across triplets for PC12 and PC36 separately.

I.4.7 ART screening and uptake

Question: What are the proportions of HIV-infected [with known status] who are on ART in each arm, in each year of the study?

Question: How much does PopART increase ART treatment?

Cohorts:

1) HIV-infected Cohort
2) Self-reported HIV infected cohort

Endpoint: Self-reported ART treatment

PopART is expected to increase the proportion reporting ART treatment amongst HIV infected.

Details: ART treatment is only assessed in participants who self-report HIV-infected status, and can be self-reported and/or verified by ART treatment documents presented by the participant. We will examine the increase in treatment amongst 1) HIV-infected participants identified in study testing and 2) participants who self-identify as HIV-infected. The same approach will be used for retained in care.

Descriptive: Proportions will be tabulated for all communities each year for each arm (participant-aggregated), for each analysis with 95% confidence limits based on the binomial distribution.

Estimates of the relative increase within a pair of study arms each year will use the analysis method described for proportions (see Community Viral Load).

I.4.8 HIV diagnosis and initiation of care

Question: For person newly diagnosed with HIV in the prior year, what proportion are linked to care within 6 months in each arm, in each year of the study?

Question: How much does PopART increase the proportion linked to care within 6 months of HIV diagnosis?

PopART is expected to increase the proportion registered for care within six months amongst HIV infected.

Cohort: Combined subsets of PC0, PC12, PC24, PC36, restricted to persons who self-report first diagnosis of HIV-infection in the prior year.

Endpoint: Registered for HIV Care within 6 months

Details: Registering for HIV care is only assessed in those who self-report HIV-infected status, and can be self-reported and/or verified by documents presented by the participant. Registering for care will be assessed using both the PC visit where diagnosis is reported and the subsequent PC visit. We will examine registered for HIV Care and time to registration amongst participants who self-identify as HIV-infected.

Description: Proportions registered within 6 months will be tabulated for all communities each year.

Analysis: Estimates of the relative increase within a pair of study arms in each Follow-up round will use the analysis method described for proportions (see Community Viral Load) in the follow-up years.

I.4.9 Sexual risk behavior

Question: Does the PopART intervention change sexual behaviors?

The PopART intervention does not directly target change in sexual behavior, although an increase in knowledge of HIV may result in change in sexual risk, for example as a result of risk disinhibition due to availability of treatment, effects due to behavioral counseling by CHiPs and condom promotion, and increased knowledge of HIV status throughout the community (including in discordant partners).

Endpoints: The following sexual behaviors will be examined separately for men and women:

- More than one sexual partner in the prior year
- Any casual/one-time sex partners in the last year
- Unprotected sex the last time.
- In the extended sexual behavior questionnaire:
    - Any concurrent sexual partners
    - Inconsistent condom use with any partners
    - Any sex for money/or gifts
    - Any known HIV discordant relationships

Cohort:

1) PC cohort, by gender
2) Risk subgroups of the PC cohort, by gender

Details: Detailed partner by partner questions about sexual risk behavior are only asked in the 20% Risk subgroup of the PC cohort. Analyses of sexual behavior will all be sex specific.

Descriptive: Proportions reporting each sexual behavior will be tabulated for all communities and by arm (participant aggregated) each year.

Analysis. Estimates of the relative increase within a pair of study arms each year will use the analysis method described for proportions (see Community Viral Load) in the follow-up years.

Subgroups:

- By HIV status at visit as determined by study testing
- By self-reported HIV status at visit
- Age

I.4.10 HIV disease progression and death

Disease progression not assessed in PC cohort. We continue to work to obtain data from clinics to assess HIV disease progression and death.

I.4.11 ART toxicity based on clinic records

ART toxicity not currently assessed in PC cohort. We continue to work to obtain data from clinics to assess ART toxicity.

I.4.12 Case notification rate of tuberculosis

Case notification of tuberculosis not currently assessed in PC cohort. We continue to work to obtain data from clinics to assess TB case notifications, however the primary assessment of changes in TB case notification will be measured using clinic notification data.

I.4.13 HIV-related stigma

Question: Does PopART change HIV Stigma?

Cohort:

1) PC Cohort
2) Self Reported HIV-infected cohort

Endpoint:

1) HIV-related Stigma Score
2) HIV-infected Stigma Score

Details: HIV Stigma is assessed on a randomly selected 20% sample of the PC participants in PC0, PC12, PC24 and PC36 using 12 questions measured using a 4-point Likert scale. Experience of HIV stigma using a different set of 12 questions measured on a Likert Scale is measured in all self-identified HIV-positive participants.

Analysis: Mean stigma scores and binary indicators of stigma will be reported for each community and each year. Estimates of the relative change within a pair of study arms each year will use a two-stage analysis method for comparing adjusted community means and prevalences within each triplet that parallels the analysis methods described for proportions.

Subgroups. HIV Stigma effects will be assessed in the following subgroups

- Sex
- Country
- Age

I.4.14 Male circumcision

Question: What proportion of uncircumcised [self-reported] HIV-uninfected men were circumcised during the PopART intervention by arm and by year?

Question: How much did the PopART intervention increase male circumcision among [self-reported] HIV-uninfected men?

Cohort

1) Self-reported HIV-uninfected men: The PC cohort, restricted to men in the PC cohort who self-report not being circumcised at enrollment and did not self-report HIV-infected status.
2) HIV-uninfected men cohort: All men in the PC cohort who self-report not being circumcised at enrollment and were HIV-uninfected by study testing.

Endpoint: Male circumcision within the previous year, assessed each year.

Analysis: Proportion of recent circumcisions will be reported for each community for each year.

PopART is expected to increase the proportion of HIV uninfected men who are circumcised. Estimates of the relative change in circumcision in each cohort within a pair of study arms each year will use the two-stage method for comparing community proportions, i.e., a t-test for the within triplet adjusted proportions.

It is known that the baseline prevalence of male circumcision is much higher in South Africa than Zambia: If the prevalence of male circumcision is not well balanced in each triplet, this may be used as a community level covariate.

Subgroups:

- Country
- Age

I.4.15 Immediate treatment policy impact on HIV

Question: What is the impact of immediate treatment policies on HIV incidence?

Approach 1: Randomized comparison

Cohort: HIV-uninfected PC0 Cohort for Arms A and B.

Analysis: The analysis time included will be restricted in each community triplet to follow-up time prior to and/or unaffected by the roll out of immediate treatment in Arm B clinics, which will likely be different in each clinic.

This randomized comparison will follow the same procedures as two-stage analysis of incidence outcomes.

Approach 2: A non-randomized epidemiological analysis using before versus after comparisons

Cohort: HIV-uninfected PC cohort for Arms B and C

Analysis: For each community, define the community immediate treatment date as the date implementation of immediate treatment began at the primary clinic defining the community catchment area.

Assume that for all HIV-infected persons in care, HIV Care visits, ART initiation and viral suppression have a substantial probability of occurring within 9 months of the community universal treatment date. Define the community suppression date at 9 months after the community immediate treatment date

Divide the seroincidence time periods observed in each person in the PC cohort into before, during and after immediate treatment initiation. For the set of HIV testing intervals (t1, t2) for an initially HIV-uninfected person (defined by an individual person who is HIV-uninfected at the beginning of the interval)

Before: All testing intervals where t2 is before community suppression date

During: All testing intervals that don’t meet either the Before or After conditions

After: All testing intervals where t1 is after the community suppression date.

Primary comparison will be relative incidence of HIV in the Before and After periods, assuming independence of periods within each community conditional on being from the same community. The analysis will use a matched pair t-test on the 14 matched before/after pairs; adjustment for cohort size, and variables associated with temporal changes in community risk independent of immediate treatment will be considered.

Caveat: Approach 2 clearly has the potential to be confounded by time given the ongoing change in guidelines and differential uptake by communities. Careful consideration would need o be given to the details of adjustment and interpretation of this analysis.

I.5 Secondary Objectives: CHiPs data

The CHiPs data will be used to measure participation of households and individuals in the CHiP intervention, and uptake of key components of the intervention. Data are available for all 7 Arm A and all 7 Arm B communities, but not for Arm C.

We have aligned how uptake of the intervention will be measured using CHiPs data, with how it will be measured using Population Cohort data, as far as possible. However, a fundamental difference between the PC and the CHiPs intervention, is that in the PC all participants consent to give a blood sample for laboratory HIV testing, so that HIV status is known for close to 100% of PC participants. In contrast, in the CHiPs intervention individuals can choose to participate but choose not to accept HIV testing using a rapid HIV test. This means that, among individuals who consent to participate in the CHiPs intervention, HIV status is known for individuals who self-report they are HIV-infected, or accept the offer of rapid HIV testing, or report they have recently tested for HIV and the test result was HIV-negative (for example in the previous 12 months); and HIV status is unknown for all other individuals.

All of the main outcomes will be summarised as proportions. Two examples are:

(1) the proportion of individuals who know their HIV status, among all individuals who consent to participate in the intervention [see I.4.4 for measurement in the PC]

(2) the proportion of HIV-infected individuals who link to HIV care within 6 months of referral to HIV care, among all who were referred to HIV care by CHiP teams and were not on ART at the time of referral, derived from a “time to event” analysis [see I.4.8 for measurement in the PC].

The general approach to data analysis will be to summarise data:

1. separately for each community
2. average value across the 14 intervention communities
3. average values for each country
4. average values for each trial arm
5. average values for each trial arm, within each country

Average values will be calculated as the average of the percentage values from each of the communities.

For almost all intervention components, uptake is expected to be similar in Arms A and B. The exception is ART uptake, because of the difference between the two trial arms for ART eligibility criteria. Thus in summarising uptake of the intervention, the emphasis will be on summaries 1, 2, and 3 above. For ART uptake, time to start ART after referral to HIV care, and retention on ART the emphasis will be on summaries 1, 4, and 5.

There will be 4 rounds (“years”) of delivering the CHiPs intervention. Uptake of all intervention components will be summarised separately for each round.

As well as overall summaries, subgroup analysis will also be presented by gender, age group, and by age group for each gender. Sub-group analyses beyond those by age group and gender are specified separately if applicable.

The analysis for six outcomes is set out below in detail, chosen on the basis that they are among the most important intervention components. The six outcomes are knowledge of HIV status, the time to link to HIV care after referral to HIV care, ART uptake, retention in HIV care, retention on ART, and uptake of MMC. Various other outcomes will also be measured, including for example the proportion of households that participate in the intervention, the proportion of individuals that participate in the intervention, the uptake of re-testing for HIV after a previous HIV-negative test result, and the time to start ART after referral to HIV care in Arm A communities. Additional sub-sections will be added to the SAP to cover these additional outcomes.

I.5.1 Knowledge of HIV status

Question: How much did PopART increase recent knowledge of HIV-Status (within the past year) through HIV testing and retesting, in each round ("year") of the study.

PopART is expected to achieve high levels of knowledge of HIV status.

Cohort: All who are contacted by CHiP teams and consent to participate in the intervention, separately for each round ("year") of the study.

Endpoint: Knowledge of HIV status, self-reported or from accepting the offer of rapid HIV testing from CHiP teams.

Details: All individuals who are contacted by CHiP teams and consent to participate in the intervention are included. Knowledge of HIV status has five mutually exclusive categories. The binary endpoint for assessing the PopART effect is Known vs Unknown

- Known; Self-reported HIV-infected
- Known; Accepted offer of rapid HIV testing from CHiP teams
- Known; Self-reported HIV uninfected, reports recently tested for HIV elsewhere ["recently" tested will be defined in at least 2 ways: testing in past 3 months, and testing in past year (12 months)]
- Unknown (previously tested but did not disclose result of most recent HIV test; has never been tested)
- Unknown; Self-reported HIV uninfected, no testing in past year

Analysis: Number and proportion who know their HIV status will be reported in each community, for each country, for each arm, and overall, with 95% confidence limits based on the binomial distribution when summarising proportions separately for each community. For the other analyses, which report average values across communities, the clustering in the data will be accounted for by calculating standard errors and corresponding confidence intervals based on the between-community variation in the proportion with the outcome (beyond that explained by country and trial arm).

Subgroups

- Pregnant women

I.5.2 HIV diagnosis and initiation of care

Question: For individuals who are identified as HIV-infected by the CHIP teams, either through self-report of HIV-infected status or from accepting a rapid HIV test from the CHIP teams and the test result was HIV-positive, what proportion are linked to care within 6 months in each round ("year") of the study?

PopART is expected to result in faster linkage to HIV care after HIV diagnosis and/or referral to HIV care.

Cohort: All individuals who were contacted, consented to participate, were identified as HIV-infected and not on ART, and were referred to HIV care by the CHiP teams, separately for each round ("year") of the study.

Endpoint: Registered for HIV Care within 6 months of referral to HIV care.

Details: Registering for HIV care is only assessed in those who are identified by the CHiP teams as HIV-positive, and can be self-reported and/or verified by documents presented by the individual.

Analysis: Number and proportion who have registered for HIV care within 6 months of referral to HIV care, among all individuals who were identified as HIV-infected and were referred to HIV care and were not taking ART at the time of referral, derived from a "time-to-event" analysis. Proportions will be reported for each community, for each country, for each arm, and overall, with 95% confidence limits based on the binomial distribution when summarising proportions separately for each community. For the other analyses, which report average values across communities, the clustering in the data will be accounted for by calculating standard errors and corresponding confidence intervals based on the between-community variation in the proportion with the outcome (beyond that explained by country and trial arm).

Subgroups

- Pregnant women

I.5.3 ART uptake among HIV-infected individuals

Question: What are the proportions of HIV-infected individuals who are on ART in each arm, among individuals who have been identified as HIV-infected by the CHiP teams, in each round ("year") of the study?

Question: How much did PopART increase ART uptake?

PopART is expected to increase the proportion reporting ART treatment amongst HIV infected individuals, especially in Arm A.

Cohort: All individuals who are contacted by CHiP teams, consent to participate in the intervention, and are identified by CHiP teams as HIV-infected either because they self-report they are HIV-infected or because they accept the offer of rapid HIV testing from the CHIP teams and the test result is HIV-positive, separately for each round ("year") of the study.

Endpoint 1: Self-reported ART treatment at time of the annual household visit.

Endpoint 2: Self-reported ART treatment at the end of the round ("year"), using the most recently available information from follow-up visits made to individuals who have been identified as HIV-infected

Details: ART treatment is only assessed in individuals who are identified by the CHiP teams as HIV-positive, and can be self-reported and/or verified by ART treatment documents presented by the individual.

Analysis: Number and proportion who are taking ART, among all individuals identified as HIV-infected, will be reported in each community, for each arm, and for each arm within each country, with 95% confidence limits based on the binomial distribution when summarising proportions separately for each community. For the other analyses, which report average values across communities, the clustering in the data will be accounted for by calculating standard errors and corresponding confidence intervals

based on the between-community variation in the proportion with the outcome (beyond that explained by country and trial arm).

Subgroups

- Pregnant women

I.5.4 Retention in HIV care among HIV-infected individuals who are registered for HIV care

Question: Does the PopART intervention achieve high levels of retention in HIV care, in each round ("year") of the study?

PopART is expected to achieve high levels of retention in HIV care among HIV-infected individuals who have ever registered for HIV care.

Cohort: All individuals who are contacted by CHiP teams, consent to participate in the intervention, and are identified by CHiP teams as HIV-infected either because they self-report they are HIV-infected or because they accept the offer of rapid HIV testing from the CHIP teams and the test result is HIV-positive, restricted to those who as part of the annual household visit report they have previously registered for HIV care, separately for each round ("year") of the study.

Endpoint: HIV care visit within the 3 months prior to the annual household visit.

Analysis: Number and proportion who are retained in HIV care, among all individuals identified as HIV-infected who have ever registered for HIV care, will be reported in each community, for each country, for each arm, and overall, with 95% confidence limits based on the binomial distribution when summarising proportions separately for each community. For the other analyses, which report average values across communities, the clustering in the data will be accounted for by calculating standard errors and corresponding confidence intervals based on the between-community variation in the proportion with the outcome (beyond that explained by country and trial arm).

Subgroups

- First registered for HIV care before or after PopART study started (pre- or post-2014)

I.5.5 Retention on ART among HIV-infected individuals who have ever taken ART

Question: Does the PopART intervention achieve high levels of retention on ART, in each round ("year") of the study?

PopART is expected to achieve high levels of retention on ART among HIV-infected individuals who have ever taken ART.

Cohort: All individuals who are contacted by CHiP teams, consent to participate in the intervention, and are identified by CHiP teams as HIV-infected either because they self-report they are HIV-infected or because they accept the offer of rapid HIV testing from the CHIP teams and the test result is HIV-positive, restricted to those who have reported at least once that they have "ever" taken ART, separately for each round ("year") of the study. For example, for round 2 ("year 2") of the study, HIV-infected individuals are included in the analysis if they have reported, at one or more of the Round 1 and Round 2 annual household visits or a follow-up visit made at any time up to the Round 2 annual household visit, that they have "ever" taken ART.

Endpoint: Taken ART within the 1 month prior to the annual household visit, and missed no pills in the previous 3 days.

Analysis: Number and proportion who are retained on ART, among all individuals identified as HIV-infected who have ever taken ART at the time of the annual household visit, will be reported in each community, for each arm, and for each arm within each country, with 95% confidence limits based on the binomial distribution when summarising proportions separately for each community. For the other analyses, which report average values across communities, the clustering in the data will be accounted for by calculating standard errors and corresponding confidence intervals based on the between-community variation in the proportion with the outcome (beyond that explained by country and trial arm).

Subgroups

- First started ART before or after PopART study started (pre- or post-2014)

I.5.6 Uptake of medical male circumcision (MMC) among HIV-uninfected men

Question: What proportion of uncircumcised [self-reported] HIV-uninfected men were circumcised during the PopART intervention, in each round ("year") of the study?

Cohort 1: Men who are contacted by CHiP teams and consent to participate in the intervention, report they are not circumcised or do not know their circumcision status, and accept the offer of HIV testing from CHiP teams and the test result is HIV-negative, and are still resident in the community one year later, separately for each round ("year") of the study.

Endpoint 1: Medical male circumcision within the previous year (self-reported), among men who in the previous round ("year") reported they were not circumcised and tested HIV-negative. This endpoint can be measured from Round 2 onwards.

Cohort 2: Men who are contacted by CHiP teams and consent to participate in the intervention, and accept the offer of HIV testing from CHiP teams and the test result is HIV-negative, separately for each round ("year") of the study

Endpoint 2: Medical male circumcision, at any time prior to the annual household visit

PopART is expected to increase the proportion of HIV uninfected men who are circumcised with MMC. Endpoint 2 measures the uptake of MMC among all HIV-uninfected men, regardless of when MMC was done, and so provides a cumulative measure of MMC uptake.

Analysis: Number and proportion of HIV-uninfected men who are circumcised with MMC will be reported in each community, for each country, for each arm, and overall, with 95% confidence limits based on the binomial distribution when summarising proportions separately for each community. For the other analyses, which report average values across communities, the clustering in the data will be accounted for by calculating standard errors and corresponding confidence intervals based on the between-community variation in the proportion with the outcome (beyond that explained by country and trial arm).

STATISTICAL ANALYSIS PLAN VERSION 3.0

16 DEC 2018

HPTN 071

Population Effects of Antiretroviral Therapy to Reduce HIV Transmission (PopART): A cluster-randomized trial of the impact of a combination prevention package on population-level HIV incidence in Zambia and South Africa

Statistical Analysis Plan

Deborah Donnell, PhD

Vaccine and Infectious Disease Division, Fred Hutch, Seattle WA

Sian Floyd, MS

Richard Hayes, DSc

Department of Infectious Disease Epidemiology, London School of Hygiene and Tropical Medicine

Version 3.0

16 December 2018

Table of Contents

A INTRODUCTION 3
B STUDY OBJECTIVES AND SUMMARY 3
B.1 MODIFICATIONS OF THE PROTOCOL AFFECTING THE STATISTICAL ANALYSIS PLAN 6
B.2 ADDITIONAL MODIFICATIONS TO THE STATISTICAL ANALYSIS PLAN 7
C OVERVIEW OF STUDY DESIGN AND RANDOMIZATION SCHEME 9
D ANALYSIS COHORTS OF THE POPULATION COHORT 10
D.1 POPULATION COHORT 10
D.2 HIV UNINFECTED COHORT 10
D.3 PRIMARY INCIDENCE COHORT 10
D.4 HIV-INFECTED COHORT 11
D.5 SELF-REPORTED HIV-INFECTED COHORT 12
E VARIABLE DEFINITIONS 12
F BASELINE TABLES AND STUDY CONDUCT 17
F.1 DESCRIPTION OF TABLES FOR THE POPULATION COHORT 17
F.2 STUDY CONDUCT MEASURES 19
G BLINDING, ACCESS TO DATA AND PUBLICATION DURING THE STUDY 20
H INTERIM MONITORING GUIDELINES 20
H.1 INTERIM MONITORING FOR EFFECT SIZE BASED ON INTERVENTION UPTAKE 20
H.2 INTERIM MONITORING OF HIV INCIDENCE 22
H.3 INTERIM EVALUATION OF VIRAL LOAD SUPPRESSION 23
I STATISTICAL ANALYSES 26
I.1 DESCRIPTIVE ANALYSES.... 26
I.2 PRIMARY ANALYSIS 27
I.3 INTERIM MONITORING ANALYSIS.... 33
I.4 SECONDARY OBJECTIVES: POPULATION COHORT 33
I.5 INTERVENTION DELIVERY OBJECTIVES: CHIPS DATA 44

A Introduction

This statistical analysis plan (SAP) details the statistical procedures that address the study objectives specified in Protocol version 1.0 of the HPTN071 (PopART) Study: Population Effects of Antiretroviral Therapy to Reduce HIV Transmission (PopART): A cluster-randomized trial of the impact of a combination prevention package on population-level HIV incidence in Zambia and South Africa

New versions of the SAP will be issued to document updates and changes in the plan. Any meaningful changes or additions to this SAP (e.g., in response to protocol amendments or violations of assumptions underlying pre-planned analyses), and the timing of such changes will be documented in Section B.1 and B.2and further described in an Appendix of the SAP, as necessary. Analysis plans for sub-studies not addressed in the protocol, and for secondary analyses not anticipated prior to study completion, will be developed as separate documents. Specifically, the following study components are not included in the SAP

1. Mathematical modeling
2. Case-control studies
3. Economic modeling
4. Ancillary studies

Formal interim monitoring will not be implemented in the study since intervention effects are expected to become stronger with each year of the intervention and reliable assessments of emerging data on the primary endpoint will likely not be available until some months after the completion of each PC round. Plans for interim monitoring of intermediate outcomes are outlined in Section H.

In addition to DSMB reviews, the PopART data team will routinely report on operational metrics (e.g., rates of recruitment and retention, intervention uptake) to the study operations team, which will communicate with sites concerning operational performance based on these metrics. No analysis of HIV incidence or community viral load will be included in these reports, the format and schedule of which are not considered further in this SAP.

B Study Objectives and Summary

This section corresponds to the protocol (Version 1.0). Changes to the protocol that affect the statistical analysis plan will be noted in Section B.1

Purpose: The purpose of this study is to determine the impact of two community-level combination prevention packages, both of which include universal HIV testing and intensified provision of HIV antiretroviral therapy (ART) and care, on population-level HIV incidence.

Design: This is a three-arm, cluster-randomized, longitudinal study to be implemented in 21 clusters (communities).

Study Population: The prevention packages will be implemented throughout the communities randomized to the intervention arms. Main study outcomes will be measured in a randomly-selected group drawn from the adult population of the communities: A *Population Cohort*.

Study Size: The combined population of all 21 clusters is approximately 1.2 million individuals. The interventions will be implemented in 14 of the 21 clusters with a combined population of approximately 800,000 individuals (adults and children) in the intervention arms. The approximate sizes of the randomly-selected groups for main study outcome assessments are:

- *Population Cohort*: 52,500 individuals
- *Case-Control Studies*: 2,400 individuals
- *Qualitative Studies*: about 2,000 individuals
- *Population Cross-Sectional Survey*: 10,500 individuals (if funded)
- *TB Survey*: about 56,000 (Arms A and B) individuals (if funded)
- *CHiPS: about 150,000 households; 300,000 adults.*

Study Arms/Interventions:

Arm A - Universal Testing with Immediate ART:

- Combination prevention package including:
  - House-to-house deployment of:
    - Universal HIV counseling and testing
    - Active linkage to care for individuals diagnosed as HIV-infected, with ***immediate eligibility for ART***
    - Promotion of male circumcision and prevention of mother-to-child transmission (PMTCT) services
    - Provision of condoms
  - Strengthening of HIV testing and services at health facilities and other venues
  - Strengthening of male circumcision and prevention of mother-to-child transmission of HIV services available in the community
  - Treatment of sexually transmitted infections (STIs) and provision of condoms at health units

Arm B - Universal Testing with ART Eligibility According to National Guidelines:

- Combination prevention package including:
  - House-to-house deployment of:
    - Universal HIV counseling and testing
    - Active linkage to care for individuals diagnosed as HIV-infected, with ***ART eligibility according to national guidelines***
    - Promotion of male circumcision and PMTCT services
    - Provision of condoms
  - Strengthening of HIV testing and services at health facilities and other venues
  - Strengthening of male circumcision and PMTCT services available in the community
  - Treatment of STIs and provision of condoms at health units

Arm C - Standard of Care (Control Arm)

- Strengthening of HIV testing and ART services according to national guidelines at health facilities and other venues
- Strengthening of male circumcision and PMTCT services available at health facilities and other venues in the community
- Treatment of STIs and provision of condoms at health facilities and other venues in the community

Study Duration: The planned duration of the entire study will be approximately 6 years, with enrollment and follow-up of communities and delivery of the intervention occurring over 4 years. Assessment of the primary outcome (HIV incidence) in the *Population Cohort* is planned to take place 12, 24, and 36 months after recruitment. Interim evaluation will take place during the first two years of intervention to determine whether to continue with the 36-month follow-up of the *Population Cohort* and the fourth year of intervention.

Primary Objective:

- To measure the impact of the two intervention packages on HIV incidence by enrolling and following a random sample of adults (the *Population Cohort*) in the trial communities for 3 years

Secondary Objectives:

- Measure the impact of the two intervention packages on the following:
    - HIV incidence over the first, second, and third years of follow-up
    - Community viral load (if funding is identified)
    - ART adherence and viral suppression (if funding is identified)
    - ART drug resistance (if funding is identified)
    - HSV-2 incidence
    - Uptake of HIV testing and retesting over the entire study period
    - ART screening and uptake
    - Time between HIV diagnosis and initiation of care
    - Retention in care
    - HIV disease progression and death
    - ART toxicity based on clinic records
    - Sexual risk behavior
    - Case notification rate of tuberculosis
    - HIV-related stigma
    - Uptake of PMTCT
    - Uptake of male circumcision
- Carry out case-control studies to examine factors related to:
    - Uptake of HIV testing during the first round of home-based testing in Arms A and B
    - Uptake of immediate treatment in Arm A
    - Uptake of HIV testing during the second round of home-based testing in Arms A and B
- Use qualitative methods to:
    - Assess popular understanding of HIV testing and treatment at study initiation and during implementation
    - Evaluate the acceptability and functioning of the Community HIV-care Providers (CHiPs) in Arms A & B
    - Evaluate the acceptability of interventions and barriers to access in Arms A & B
    - Document the effect of the interventions on social networks, stigma, sexual behavior, alcohol use, gender-based violence, HIV identity, other HIV prevention options and community morale
    - Evaluate the process and challenges of community consultation and applying ethical principles

- Measure the burden experienced by local health centers due to implementation of the intervention in the community
- Measure the incremental cost of the two intervention packages through systematic recording of costs in intervention and control communities
- Estimate the effectiveness and cost-effectiveness of the intervention packages and alternative packages, both in the chosen study populations and in other populations by fitting mathematical models based on the empirical data from the trial, including data related to cost.

Study Sites: The study is implemented in the communities identified below.

- The study communities in Zambia are spread across 4 provinces and 5 districts. Each community is the catchment population of a government health facility.
    - Chimwemwe and Ndeke in Kitwe District (Copperbelt Province)
    - Chipulukusu and Chifubu in Ndola District (Copperbelt Province)
    - Makululu and Ngungu in Kabwe District (Central Province)
    - Chawama, Chipata and Kanyama in Lusaka District (Lusaka Province)
    - Maramba and Dambwa in Livingstone District (Southern Province)
    - Shampande in Choma District (Southern Province)

- The study communities in South Africa are located in the Cape Metro District and Cape Winelands District of the Western Cape Province. As above, the communities are defined by the catchment population of a government health facility.
    - Delft South (Metro District)
    - Kuyasa (Metro District)
    - Luvuyo (Metro District)
    - Town II (Metro District)
    - Ikhwezi (Metro District)
    - Bloekombos (Metro District)
    - Dalevale (Cape Winelands District)
    - Wellington (Cape Winelands District)
    - Cloetesville and Idasvalei (Cape Winelands District)

B.1 Modifications of the Protocol Affecting the Statistical Analysis Plan

B.1.1 Version 2.0:

Immediate offer of ART for all HIV infected individuals attending health care facilities will be implemented within all clinics involved in the HPTN071 (PopART) trial. This will be offered ahead of local guidelines once protocol version 3.0 is approved and funding to support the change can be secured. Change to analysis plan:

> The primary analysis for the assessment of the PopART impact on HIV incidence is unchanged, as the implementation of immediate offer of ART for all HIV infected individuals attending health care facilities did not affect Year 1 of the intervention and is likely to only partially impact outcomes in Year 2. Even in Year 3, as for the PopART intervention itself, effects will still take time to accumulate, especially for impact on HIV incidence.
>
> A secondary analysis objective has been added to assess the impact of immediate treatment policies on community HIV incidence.

Addition of PC12N participants, enrolled during the second year of the cohort, in communities where fewer than 2000 participants were enrolled in PC0.

Change to analysis plan:

> PC12N are included in the PC and will contribute endpoints as accumulated. In particular, HIV-uninfected PC12N participants will add to seroincidence and person years accumulated at PC24 and PC36.
>
> Adjustments for age at baseline was changed to age at each visit in the primary analysis. The first stage in the primary analysis estimates expected incidence by age and sex. The modification was required because age at enrollment is not equivalent in PC0 and PC12N/PC24N participants.

B.1.2 Version 3.0

Addition of PC24N participants, enrolled during the third year of the cohort in Arm A and C community, where sufficient remaining households exist.

> PC24N are included in the PC and will contribute additional endpoints and person time for the PC24-PC36 period.
>
> The proposed secondary analysis objective to assess the impact of immediate treatment policies on community HIV incidence was changed to exploratory and removed from the SAP.

B.2 Additional modifications to the Statistical Analysis Plan

At the October 2016 DSMB review, the protocol team reviewed the mathematical modeling projections of potential intervention effect from the individual based model, which was based on the current data about intervention uptake. The DSMB approved a change to the primary endpoint of the trial from assessing the impact of the intervention over the three years of PC follow-up to considering only the second and third year of the PC. i.e., differences in seroincidence rate as measured at PC24 and PC36 only.

The approach to estimation of the standard error for the two-step analysis approach was modified to utilize the three arm design. In the previous version, different estimates of the test statistic standard error were used for Arm A vs Arm C and Arm B vs Arm C, each based on the communities in those arms, resulting in a 6 df t-test. However, it is more efficient to use the MSE from a two-way ANOVA to estimate the common standard error, resulting in the same SE for both comparisons and a t test with 12 df.

Prior versions of the SAP noted the possible use of a community level covariate adjustment for the two-stage analysis, which reduces the degrees of freedom for the primary comparison by 1. The protocol chair decided for the primary HIV incidence outcome to adjust the analysis for Baseline HIV Prevalence based on the following considerations:

- Overall balance in baseline HIV prevalence across arms is good, but there are some triplets where it is less good (notably Triplet 7)
- This might result in a large value for s (based on the between triplet variation in differences between study arms) which then reduces power
- If baseline prevalence is a good predictor of incidence, then adjustment for baseline should help to reduce s

- The critical points for t with 11 and 12 df are very similar (2.18 and 2.20)

Version 3.0

- Clarification of the HIV testing included in defining "HIV status by study testing"
- Clarification of HIV seroconverter to include participants who are determined to be HIV-infected with acute infection
- Age adjustment for HIV incidence analysis will use the Lexis expansion method, to appropriately attribute partial person years to the correct age category in each year of participation.
- An additional subgroup was added for PC24 viral load suppression: Participants self-reporting current ART use at PC24
- Treatment of zeros was added to accommodate this potential in subgroup analyses of HIV incidence.
- The community HIV prevalence covariate used to adjust the Step 1 HIV incidence analysis was further clarified to be a country-specific age and sex standardized HIV prevalence, using the age and sex distributions available from the Arm A and B intervention communities in Zambia and South Africa
- Clarification that Age will be computed from self-report or imputed birthdate, where birthday is imputed at 6 months prior to the visit date where age is reported.
- Addition of clarification that participants with a "sero-reversion" in their sequence of HIV status determinations will have all HIV visit status set to missing.
- Addition of unadjusted relative risk definition.
- Addition of analysis of the endpoint Viral Burden, as an indirect marker of the probability of exposure to an HIV-infected person with unsuppressed viral load.
- Change to include imputation in the primary analysis for missing data at PC12 visit for those enrolled and HIV-uninfected at PC0. This followed completion of blinded endpoint adjudication, after which it was determined that 11% of person time was omitted if the formerly proposed complete case approach was used. The complete cases analysis, excluding all person time with missing HIV status at PC12, will also be presented as a sensitivity analysis.
- Clarification that for seroconverters, if the first HIV-infected visit is an acute infection, the estimated time of infection will be the time of the visit with acute infection.

C OVERVIEW OF STUDY DESIGN AND RANDOMIZATION SCHEME

This section corresponds to the protocol (Version 1.0).

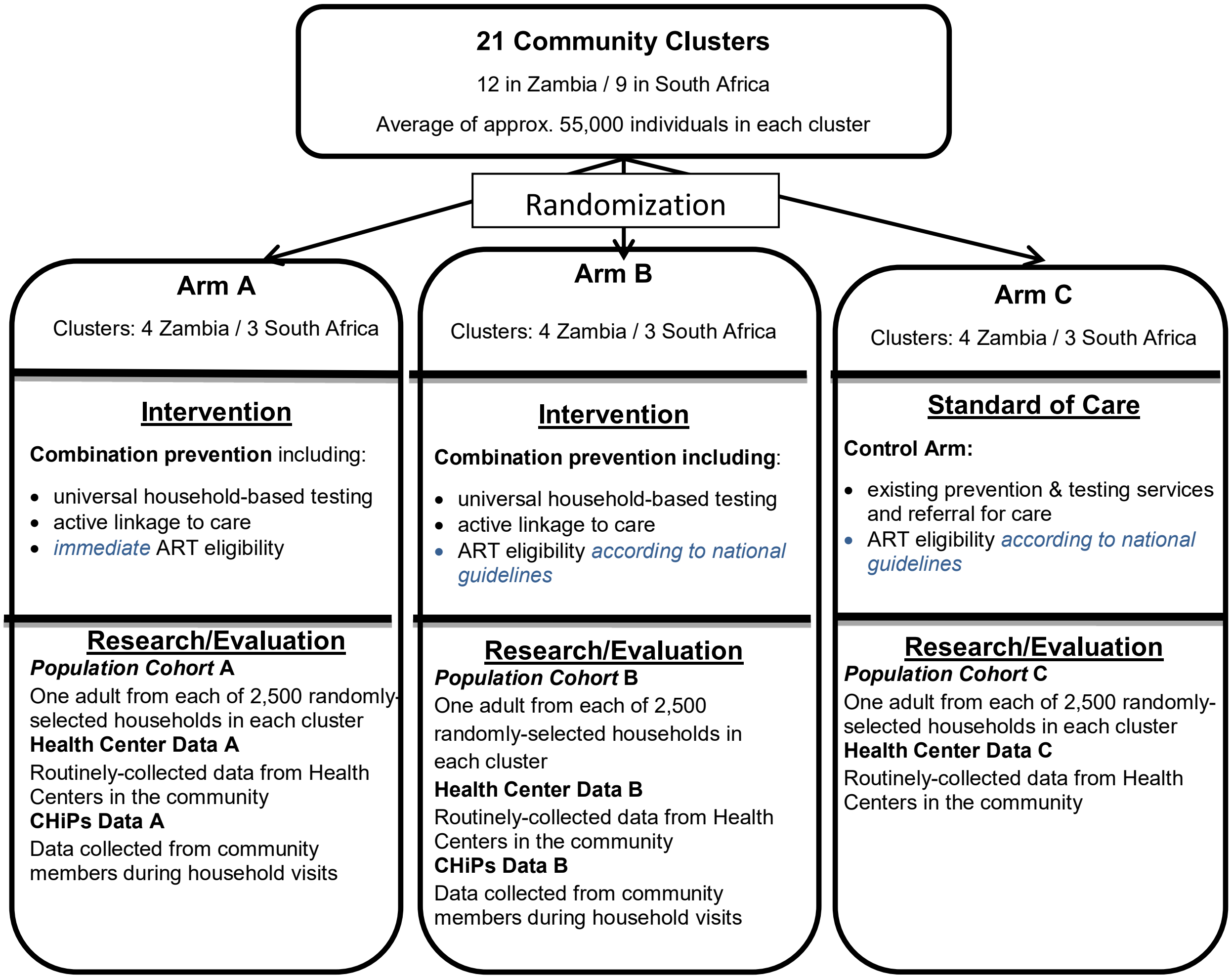

Primary Outcome Measure

•HIV incidence measured over 3 years in ***Population Cohort***

Secondary Outcome Measures

•***Population Cohort***: HIV incidence measured over 1st , 2nd , and 3rd years, HSV-2 incidence, sexual risk behavior[+], community VL[+]*, viral suppression (ART patients)*[+], drug resistance (ART patients with detectable VL)*[+]

•***Population Cohort*** and **Health Center Data:** ART Adherence[+], HIV disease progression and death[+], ART toxicity[+], HIV stigma[+]

•**Health Center Data:** TB notification and mortality rates

•***Population Cohort*, Health Center Data, CHiPs Data:** uptake of PMTCT[+], uptake of male circumcision[+], ART screening and uptake[+], uptake of HIV testing and retesting[+], time between diagnosis and initiation of care[+]

[+] Objectives that will also be addressed by the *Population Cross-Sectional Survey*, if funded
* Pending funding for these assays.

D Analysis Cohorts of the Population Cohort

This section describes the analysis cohorts of the population cohort. For the purposes of analysis in the Population Cohort the beginning of the PopART intervention is defined as January 1, 2014.

Throughout, HIV status ***by study testing*** refers to the HIV testing conducted at site and central labs using the blood samples collected at the PC visits by the study nurse. It does not include HIV testing conducted by the CHiPs, or rapid HIV tests offered to PC participants during their PC visits.

D.1 Population Cohort

The population cohort (PC) is all participants enrolled in the PC, including PC12N participants, enrolled in the second year at the PC12 visit and PC24 N participants enrolled in the third year at the PC24 visit.

D.1.1 Subgroups of the Population Cohort

An extended group of questions are administered to two subgroups at each PC round. The subgroups are mutually exclusive (so a participant is not asked to complete two extended sets of questions). The subgroups are re-selected each cycle.

- PC0, PC12, PC24 and PC36 Risk subgroups: A 20% randomly selected subgroup is given an extended sexual risk and stigma questionnaire.
- PC0, PC12, PC24 and PC36 Economics subgroups: A 20% randomly selected subgroup is given an extended economic and quality-of-life assessment.

D.2 HIV Uninfected Cohort

The HIV Uninfected Cohort includes:

- PC participants who were negative at PC0 and/or PC12 and/or PC24 by study testing. Participants who are HIV-uninfected and enrolled at PC12N and PC24N are included.
- Determination of HIV status requires completed HIV testing according to the study HIV test algorithm, which generally requires a single, fourth-generation HIV test for HIV-uninfected status and two different fourth-generation tests for HIV-infected status. HIV status can also be determined by review of all available lab testing in consultation with the HPTN 071 endpoints committee.
- Participants who did not have HIV status determined as HIV-negative on at least one visit are not included in the cohort.

D.2.1 Subgroups of the HIV Uninfected Cohort

HIV uninfected cohorts for each year of the PC are defined as follows:

- **PC0, PC12, PC24, PC36**: All participants who have HIV status HIV-uninfected at the corresponding PC visit. These will include participants enrolled in PC12N, and PC24N.

D.3 Primary Incidence Cohort

The primary endpoint analysis of HIV incidence will include HIV incidence evaluated in the second and third full year of the intervention as measured by the incidence at PC24 and PC36 visits amongst HIV uninfected PC0, PC12/PC12N and PC24/PC24N participants. Among these participants, the Primary Incidence Cohort includes the subset who meet the following criteria:

- PC0 participants who were negative at PC12 or, if PC12 HIV status is unknown, have subsequent HIV test status determined by study testing at PC24 and/or PC36.
- Participants who were HIV-uninfected when enrolled at PC12N and PC24N are included. Time on study will be from the earliest of PC12 or PC24 visit
- Determination of HIV status follows the description in Section D.2.
- HIV status was determined on at least two different visits, one of which was PC24 or PC36.
- Imputation will be used to include data from HIV negative PC0 participants with PC12 HIV status unknown who have HIV assessed at PC24 and/or PC36.

D.3.1 Subgroups of the Primary Incidence Cohort

HIV incidence cohorts for each year of the PC are defined as follows:

- **PC12**: All PC participants who have HIV status HIV-uninfected at PC12, including PC12N, and have HIV status determined in PC24. Imputation will be used for unknown HIV status at PC12, as above.
- **PC24**: All PC participants who have HIV status HIV-uninfected at PC24/PC24N, including P24N, with HIV status determined in PC36. Imputation will be used for unknown HIV status at PC24, as above.

D.3.2 Full Incidence Cohort

The full incidence cohort includes HIV incidence evaluated in all years of follow-up of the PC as measured by the incidence at PC12, PC24 and PC36 visits amongst HIV uninfected PC0, PC12N and PC24N participants.

The full endpoint cohort follows the definition above, with the addition of

- PC0 participants who were negative at PC0 and had their last HIV status measured at PC12.

D.4 HIV-Infected Cohort

All PC participants who have HIV-infected status at PC0, PC12, PC24, or PC36. Participant data are only included in the cohort from the date of the first HIV-infected visit.

D.4.1 Subgroups of the HIV-Infected Cohort

- **Seroconverter Cohort**
  All PC participants who acquire HIV infection during the HPTN071 protocol. A seroconverter is defined as a participant who has HIV-uninfected status documented at enrollment or during PC follow-up and subsequently has confirmed HIV-infected status. This includes participants who have acute HIV infection at the last study visit, without evidence of seroconversion (i.e., without detectable anti-HIV antibodies).

- **PC0, PC12, PC24, PC36**
  Participants of the HIV-Infected Cohort who are HIV infected at PC0, by study testing, at PC12, PC24, PC36, respectively
- **Community Viral Load: PC24**
  Viral load is measured in all HIV-infected participants at PC24, thus this eligible cohort is the same as HIV-infected PC24, above, minus those where viral load was not tested.
  PC0, PC12 and PC36 Community Viral Load Random Sample

Randomly selected subset of ~75 HIV-infected participants in each community in the HIV-infected cohort at PC0, PC12 and PC36. Seroconverters in the PC12 round are excluded from the sampling frame for PC12 cohort in the expedited evaluation of viral load for interim monitoring.

D.5 Self-reported HIV-Infected Cohort

All PC participants who have self-reported they are HIV-infected at PC0, PC12, PC24, or PC36. Participants' data are included in the cohort from the visitthey first self-report they are HIV-infected. In the case where participants did not consistently self-report HIV-infection and data are not assessed (e.g. current ART use is collected only when a participant self-reports HIV-infected), participants will be excluded from analyses requiring such data.

D.5.1 Subgroups of the Self-reported HIV-Infected Cohort

- PC0, PC12, PC24, PC36
  Participants of the self-reported HIV-Infected Cohort who first report, or have previously reported, they are HIV-infected at PC0, PC12/PC12N, PC24/PC24N, PC36, respectively (each cohort includes those self-reporting HIV infected in previous visits).
- **Newly diagnosed after start of PopART:** all participants who self-report HIV-infected diagnosis after the initiation of PopART intervention (January 1, 2014).
- **Registered in care since start of PopART:** all HIV-infected participants who did not self-report being registered in care before the initiation of the PopART intervention who subsequently report being HIV-infected and registered in care.
- **Initiated ART since start of PopART:** All participants who did not self-report current ART before the initiation of the PopART intervention who subsequently report being HIV-infected and on ART during PC follow-up.

E Variable definitions

Label	Description/Definition	Type	Units/Categories	Use in analysis
**Administrative**				
Household ID	Uniquely identifies each household randomly selected from the sampling frame	Label	8 digit number	Identifier
Participant ID	Uniquely identifies each selected participant	Label	10 digit number	Identifier
Barcode/specimen ID	Uniquely identifies specimens collected for each enrolled participant	Label	10-digit number	Identifier
Enrolled	Selected participant who consented to participate	Binary	Yes/No	Cohort
Triplet	Uniquely identifies each triplet	Label	1-7	Stratification
Randomization arm	Randomization arm for each community	Categorical	A,B,C	Primary covariate

Community number	Identifies community of each participant	Categorical	101-121	Cluster
Visit	Time point for data collection	Categorical	PC0, PC12, PC24, PC36	Study Time
Date of visit	Calendar date of visit	Calendar time	ddMMMyy/PC0, PC12, PC24, PC36	
Duration on study	Duration between HIV tests: e.g. Date of PC12 specimen draw – Date of PC0 specimen draw in years	Duration	Years/ PC12, PC24, PC36	Time on study
**Laboratory Assessments**				
Site HIV Combo assay (HCA)	Singleton Architect HIV Ag/Ab Combo test (Abbott) HIV result from in-country (local lab) testing	Categorical	Reactive/Non-reactive PC0, PC12, PC24, PC36	Part of primary endpoint
LC HCA	Architect HIV Ag/Ab Combo test HIV result from LC. Performed as QC for 10% of site HCA nonreactive, and when site HCA is not done	Categorical	Reactive/Non-reactive PC0, PC12, PC24, PC36	Part of primary endpoint
LC Biorad HIV result	GS HIV Combo Ag/Ab EIA (BioRad) test HIV result from lab (LC) testing. Performed for all Site HCA reactive	Categorical	Reactive/Non-reactive PC0, PC12, PC24, PC36	Part of primary endpoint
LC within visit discrepancy result	Result of additional testing conducted by the LC when Reactive/non-reactive HIV discrepancy on two HIV tests from the same visit	Categorical	Negative/Positive/Inconclusive PC0, PC12, PC24, PC36	Part of primary endpoint
HIV confirmed status	Result determined by Site HCA, LC HCA, LC Biorad and any additional testing conducted by the LC. Additional testing is conducted at relevant visits for all participants who change HIV status between visits i.e. are potential seroconverters or who are observed to "sero-revert"	Categorical	Negative, Positive, Missing. PC0, PC12, PC24, PC36	Used to determine primary endpoint

Acute infection	Determination whether the first HIV-infected visit for a seroconverter was acute or not. Acute infections are characterized as confirmed infections without detection of antibodies	Categorical	Yes, No<br>PC0, PC12, PC24, PC36	Used to characterize acute infection. Date of infection is estimated as acute visit date.
ARV drug testing	An objective, biomedical measure that detects an array of ARV drugs, if resources are available.	Yes/No	Presence/absence for each of an array of ARV drugs. Most probably assessed in HIV-infected PC24 cohort.	Objective marker of use of ART
**Demographics/Subgroups**				
Gender	Gender of participant	Categorical	Male/Female	Subgroups
Age in years at each visit	Age of participant at time of visit: calculated from birthdate and visit date. If exact birth date was unknown, is imputed as 6 months prior to the visit date where age in years was given.	Numeric	18 or older	Subgroups
Marital status, enrollment	Marital status at enrollment	Categorical	Currently married/living as married,<br>never married,<br>divorced/separated,<br>widowed	Demographic
Education, enrollment	Education level at enrollment	Categorical	None/Grade 1-2,<br>Grade 3-6,<br>Grade 7-10,<br>Grade 11-12,<br>College/University,<br>Other	Demographic
Sexually active at enrollment	Participant report of sexual activity at enrollment	Binary	Yes/No	Subgroups
HIV status at enrollment	HIV confirmed status at enrollment visit, PC0, PC12N or PC24N.	Categorical	Negative, Positive, Missing. Missing if no enrollment sample taken, or unable to determine	Subgroup

			HIV status at enrollment	
**Secondary Endpoints**				
HSV-2 status	Derived from the site and central HSV-2 results, Uninfected if site HSV-2 result was non-reactive. Infected if site testing was reactive. If local testing was gray zone or indeterminate, reactive or non-reactive as determined by central testing. Indeterminate if local gray zone and central test indeterminate i.	Categorical	Infected/Uninfected /Indeterminate.<br>PC0, PC36	Secondary endpoint
Self-reported HIV status	Participant report of HIV status	Categorical	Skipped, Negative, Positive, Don't Know/Unwilling to disclose<br>PC0, PC12, PC24, PC36.	Cohort
Plasma viral load	Number of viral copies per mL in plasma	Numeric or BLQ	Copies/mL or BLQ<br>Subset of HIV-infected PC12, PC36 (75/community);PC2 4	Secondary endpoint
Undetectable PVL	Whether PVL is <400 copies/mL	Binary	0/1, subset of HIV-infected PC12, PC36 (75/community); PC24	Secondary endpoint
Self -reported ART uptake	Participant report of ART uptake, assessed only in those self-reporting HIV-infected status	Binary	Skipped,<br>Yes/No<br>PC0, PC12, PC24, PC36 (HIV-infected)	Secondary endpoint
Registered for HIV care	Participant report of registering for HIV care, assessed only in those self-reporting HIV-infected status	Binary	Skipped,<br>Yes/No<br>PC0, PC12, PC24, PC36 (HIV-infected)	Secondary endpoint
Date of most recent HIV care visit	Participant report of last clinic visit for HIV care	Calendar time	Skipped,<br>MM/YY<br>PC0, PC12, PC24, PC36 (HIV-infected)	Secondary endpoint

Number of sexual partners in prior year	Number of partners in last 12 months. 0 if no sexual partners in last 12 months	Count	0-999<br>PC0, PC12, PC24, PC36	Secondary endpoint
Number of sex events per month	Participant report of average number of sex events per month, assessed only in the cohort of participants assigned to the extended Risk questionnaire. 0 if no sexual partners in last 12 months	Count	0-99<br>PC0, PC12, PC24, PC36	Secondary endpoint for Risk subset
How often used condoms	Participant report of condom use, assessed only in the cohort of participants assigned to the extended Risk questionnaire	Categorical	Skipped, All the time /Sometimes/Never<br>PC0, PC12, PC24, PC36	Secondary endpoint for Risk subset
Exchange of money/drugs/food/shelter for sex	Participant report of exchange of money, drugs, food or shelter for sex, assessed only in the cohort of participants assigned to the extended Risk questionnaire	Categorical	Yes/No<br>PC0, PC12, PC24, PC36	Secondary endpoint for Risk subset
Any casual or one-time partners	Participant report of any casual or one-time partners. No if no sex partners	Categorical	Yes/No<br>PC0, PC12, PC24, PC36	Secondary endpoint
Unprotected sex	Participant report that most recent sex act was unprotected. No if no sex partners	Categorical	Yes/No<br>PC0, PC12, PC24, PC36	Secondary endpoint
Concurrent sexual partners	Participant report of concurrent sexual partners, assessed only in the extended Risk questionnaire. . No if no sex partners	Categorical	Yes/No<br>PC0, PC12, PC24, PC36	Secondary endpoint for Risk subset
Inconsistent condom use	Participant report of any inconsistent condom use, assessed only in the extended Risk questionnaire	Categorical	Yes/No<br>PC0, PC12, PC24, PC36	Secondary endpoint for Risk subset

HIV discordant relationship	Participant report of any partner with discordant HIV status, assessed only in the extended Risk questionnaire	Categorical	Yes/No<br>PC0, PC12, PC24, PC36	Secondary endpoint for Risk subset
Stigma scores	HIV related stigma scores, calculated from 12 questions measured on a 4-point Likert scale, assessed only in the extended Risk questionnaire	Numeric	PC0, PC12, PC24, PC36	Secondary endpoint for Risk subset
Pregnancy during past year	Participant report of pregnancy during the past year	Categorical	Yes/No<br>PC0, PC12, PC24, PC36 (Female)	Cohort
Use of ART for PMTCT during pregnancy	Participant report of ART use during pregnancy	Categorical	Yes/No<br>PC0, PC12, PC24, PC36 (Female)	Secondary endpoint
Circumcision in the past year	Participant report of circumcision during the past year	Categorical	Yes/No<br>PC0, PC12, PC24, PC36 (Male)	Secondary endpoint
Community level covariates				
HIV Prevalence at PC0	Proportion of tested cohort in each community who are HIV-infected	Numeric percentage	PC0	Subgroup
Age x gender x country standardized HIV prevalence at PC0	Estimate of proportion HIV-infected in each community, based on intervention data of 18—44 age and gender distribution in each country.	Numeric percentage	PC0	Primary adjustment variable

F Baseline Tables and Study Conduct

F.1 Description of tables for the Population Cohort

This section describes the tables of baseline characteristics of the PC0 cohort. A subset of these will be presented to the DSMB at each meeting. These tables will appear in the open DSMB report. When results are presented by study arm, as this is an unblinded trial, the arms will be labeled Arm A, B and C, corresponding to the trial arms.

Similar tables will describe the enrollment characteristics of the PC12N and PC24N cohorts. However, the PC0 cohort will comprise the primary description of the baseline characteristics of the communities prior to intervention.

F.1.1 Study Accrual

Activation dates, first enrollment dates, target accrual numbers, and the number of participants accrued each month are presented for each country and community.

F.1.2 Baseline Demographic Characteristics

Demographic characteristics summarized include age during PC0 recruitment, gender, education, marital status, employment status, type of employment among those currently employed, nights spent away from home in the last three months, and nights spent outside the community in the last three months. Characteristics are presented by country, community and arm for all participants and for men and women separately by country and community.

F.1.3 Baseline Household Characteristics

Household characteristics summarized include building type, main source of drinking water, and main source of energy used for cooking. Characteristics are summarized by country and arm.

F.1.4 Baseline Risk Characteristics

Risk characteristics summarized include alcohol use, recreational drug use in the last 12 months, sexual activity in the last 12 months, number of sex partners in the last 12 months, partners living outside the community and condom use during the last sex event for all participants. Circumcision status is reported for men. The number of sex partners in the last 12 months is categorized as 0, 1, 2 or 3 or more partners. Characteristics are summarized for all participants by country, community and arm and for men and women separately by country and community.

F.1.5 Baseline Sexual Activity by Age Group

Sexual activity risk characteristics are summarized by age group overall and for men and women separately. Risk characteristics summarized include reported sexual activity in the last 12 months, number of sex partners in the last 12 months, partners living outside the community and condom use during the last sex event.

F.1.6 Baseline HIV Self-Report

Self-reported HIV status and HIV testing history by country, arm and community are summarized for the entire cohort of enrolled participants and for the cohort of those participants who report no prior visits from CHiP teams on the date of enrolment to PC0. Self-reported HIV status is categorized as missing, positive, negative, unknown or unwilling to disclose, and never tested.

F.1.7 Baseline Rapid Test Acceptance

Baseline rapid test acceptance and rapid test results are presented by country, by arm, by arm in Zambia, by arm in South Africa and by community for the entire cohort of enrolled participants and for participants who report no prior visits from CHiP teams. Rapid tests refer to the HIV tests offered to the participant during the PC enrollment visit by the Research Nurse. The percentages of those accepting the rapid test are shown overall, for those self-reporting HIV- infected, for those self-reporting HIV-negative, and for those never tested. Each rapid test produces a result that is non-reactive, reactive or invalid. Per study procedure, a reactive rapid result is followed by a second rapid test. The “final” rapid result is reported in this table. The result is reported as non-reactive if the first test is non-reactive. The result is reported as reactive if both the first and second rapid tests are reactive. A reactive rapid result

followed by an invalid or non-reactive test, or not followed by a repeat test, is considered invalid/inconclusive. If the first rapid test is invalid, then the result is also considered invalid.

F.1.8 Baseline HIV Care and ART Uptake

Baseline HIV care and ART uptake among those self-reporting HIV-infected at baseline are summarized by country, arm and community. The number of self-reported HIV-infected participants who report that a CHiP team has not yet visited the household is shown. Participants who have been visited by a CHiP team and report a recent first positive HIV test may have discovered their HIV status through the study intervention. The year of the first positive HIV test and ever registering for HIV care are shown for all participants self-reporting HIV infected. The year registered for HIV care and ART use are summarized among those reporting registered for HIV care. The primary reason for starting ART, the year starting ART, and current ART use are summarized for those reporting ever using ART. Ever stopping ART in the last 12 months and missing any ART pills in the last seven days are summarized among those reporting current ART use.

F.1.9 Baseline HIV and HSV-2 Results

HIV and HSV-2 test results are presented by country, arm and community. HIV status is assessed by a combination of in-country and HPTN Laboratory Center (LC, Johns Hopkins) testing. A first HIV test is performed in-country. If the first test is non-reactive, the HIV test is considered negative (10% of these are retested at the LC for quality assurance). If the first test is reactive or invalid, a second test is conducted at the LC. In the case of discordant or inconclusive testing, the LC conducts further tests to establish HIV status. The percentages of the in-country HIV and HSV-2 test results are shown for those samples that have been tested. No result obtained refers to samples that were to be tested but could not be tested effectively due to machine failure, etc.

F.1.10 Baseline HIV Prevalence

Baseline HIV prevalence is presented for each community and overall. In addition, for men and women in each country separately, by age category, and by age category for each of men and women. The denominator for the prevalence calculations is the number with completed determination of HIV-status, including LC HIV confirmatory tests.

F.1.11 Baseline HIV Care and ART Uptake Among HIV+

Baseline HIV care and ART uptake are summarized among those HIV-infected by the in-country lab testing. Ever registered for HIV care is missing if the participant self-reported HIV-infected but did not answer the question, “Have you ever registered for HIV care?” None of those self-reporting something other than HIV-infected status are considered registered for care. Current ART use is missing if the participant self-reported HIV-infected but did not answer the question, “Have you ever taken any ART?” None of those self-reporting something other than HIV-infected status are considered on ART.

F.2 Study conduct measures

F.2.1 Consort Diagram

A consort diagram will report for each arm both at the level of individual and community, following Consort diagram best practices for community randomized trials. The consort diagram will include reporting of new participants enrolled in PC12N and PC24N, and the number of participants retained in each PC round.

F.2.2 Retention and Enrollment at PC12N and PC24N

In each PC round, retention of participants among those eligible for each PC round will be reported as completed, missed or terminated (or outcome not yet known, during the round). All those not terminated are eligible for the next PC round. The number of participants enrolled in PC12N and PC24N are added to those eligible for the next round.

F.2.3 Completeness of Specimen Collection

In each PC round the completeness of specimen collection is presented by country, arm and community. Percentages of sample collection for in-country HIV testing, in-country HSV-2 testing, Laboratory Center (LC) testing and storage and in-country storage are summarized. The denominator for these participants includes all enrolled participants. Completeness of HIV testing

All specimens are tested for HIV using a singleton Architect run. All reactive HIV results by in-country testing will be confirmed at the HPTN Laboratory Center (LC) provided an aliquot for LC testing and storage was collected. Ten percent of the HIV non-reactive in-country results are selected for QA at the LC. The LC will also perform confirmatory testing for all samples for which the in-country testing did not yield a result. The percentages of the HIV confirmatory testing completeness and results from the LC are summarized.

G Blinding, Access to Data and Publication during the Study

Neither site staff nor participants will be blind to community assignment. It is study policy not to report aggregate data by community name or have information about community arm assignment in public documents. And although only the DSMB and statisticians preparing analysis reports will review accumulating interim data by study arm, study sponsors, monitors and non-site staff are likely to become aware of community assignments while the study is ongoing. To the extent possible, however, decisions regarding additions or modifications to planned analyses, determination of inclusion in or exclusion from analysis cohorts or datasets, and rules for handling missing or incomplete data will be made by statisticians, study leadership or other study staff who do not have access to outcome data grouped by arm. Lab assessments will be done blinded to community treatment assignment and the Endpoints Committee will not be provided community assignments when adjudicating HIV endpoint data. The level of blinding maintained when performing these activities will be described in the study report or manuscripts, as relevant.

No data on comparisons between the intervention arms (A and B), nor between the intervention arms (A and B) and the standard of care arm (C), will be presented or published during the study.

The study team will publish and present data from the PC0 (baseline) data during the study. PC0 data will also be accessed by the mathematical modelling group to inform and validate the model.

H Interim Monitoring Guidelines

H.1 Interim monitoring for Effect Size based on Intervention Uptake

The uptake of intervention components will be reported using both data from the CHiPs (available only in arms A and B) and the population cohort (primarily through self-report). Targets have been set for each of the intervention components for CHiPs for Arms A and B. However, for interim monitoring, a

range of plausible intervention effect sizes will be assessed through mathematical models that allow for the combined effect of the prevention cascade.

H.1.1 Protocol effect size

In the study design, mathematical models used the targets detailed in the Protocol (V1.0). These modelling results are included here for reference.

Table 1- Parameter values assumed for the model of the impact of the intervention for central and optimistic target scenarios, and projected impact on HIV incidence in Arms A and B compared with Arm C, assuming intervention roll-out over a 6-month time period

	Parameter	Central Target		Optimistic Target	
	Annual coverage of test and treat campaign	70%		75%	
	Treatment failure & drop-out rate, per year	10%		10%	
	Effectiveness of ART in blocking transmission	90%		95%	
	Take up of male circumcision when offered	50%		50%	
		Arm A	Arm B	Arm A	Arm B
Zambia	Impact on cumulative incidence (3 years)	58%	25%	66%	29%
	Impact on cumulative incidence (2 first years)	54%	23%	62%	27%
	Impact on HIV incidence during Year 1	45%	19%	53%	23%
	Impact on HIV incidence during Year 2	63%	28%	72%	33%
	Impact on HIV incidence during Year 3	68%	31%	76%	36%
South Africa	Impact on cumulative incidence (3 years)	57%	23%	65%	27%
	Impact on cumulative incidence (2 first years)	52%	21%	61%	26%
	Impact on HIV incidence during Year 1	44%	18%	52%	23%
	Impact on HIV incidence during Year 2	62%	26%	71%	31%
	Impact on HIV incidence during Year 3	66%	29%	75%	33%

Table 2- Parameter values assumed for the model of the impact of the intervention for central and optimistic target scenarios, and projected impact on HIV incidence in Arms A and B compared with Arm C, assuming intervention roll-out over a 12-month time period

	Parameter	Central Target		Optimistic Target	
	Annual coverage of test and treat campaign	70%		75%	
	Treatment failure & drop-out rate, per year	10%		10%	
	Effectiveness of ART in blocking transmission	90%		95%	
	Take up of male circumcision when offered	50%		50%	
		Arm A	Arm B	Arm A	Arm B
Zambia	Impact on cumulative incidence (3 years)	54%	23%	62%	27%
	Impact on cumulative incidence (2 first years)	47%	20%	56%	25%
	Impact on HIV incidence during Year 1	34%	14%	42%	19%
	Impact on HIV incidence during Year 2	61%	27%	70%	32%
	Impact on HIV incidence during Year 3	67%	31%	76%	36%

South Africa	Impact on cumulative incidence (3 years)	52%	21%	61%	26%
	Impact on cumulative incidence (2 first years)	46%	19%	54%	23%
	Impact on HIV incidence during Year 1	33%	14%	41%	18%
	Impact on HIV incidence during Year 2	60%	26%	69%	30%
	Impact on HIV incidence during Year 3	66%	29%	75%	33%

H.1.2 Interim modelling of effect size.

The complexity of the multiple components of the intervention makes it difficult to estimate the population impact on HIV incidence. Operational futility in the study will use a mathematical model to assess the likely range of effect sizes, based on the intervention uptake data collected in the trial. The description of the mathematical model and the scenarios for uptake are detailed in a separate Modeling Projection Report. This will be used to estimate whether the power of the trial remains high.

The Study Team proposes that if the power for the comparison of Arms A and C under scenarios consistent with the observed intervention uptake becomes less than 50%, given data on HIV incidence in Arm C and HIV prevalence in the PC, the DSMB could recommend early stopping of the study for futility, i.e. when the trial is unlikely to have the power to demonstrate effectiveness.

Under current assumptions of 1% incidence and 22% HIV prevalence, 50% power corresponds to a PopART intervention community effectiveness of approximately 25%, i.e. a recommendation to stop the trial for futility if study power was estimated to be "only" 50% would correspond to stopping the trial if the effectiveness of Arm A relative to Arm C was projected to be smaller than 25%. At each DSMB meeting, updated projections of intervention effect and power based on updated assumptions and protocol changes will be provided in a separate Modeling Projections Report.

H.2 Interim monitoring of HIV incidence

The study was powered assuming an HIV incidence rate of 1.0-1.5 per 100-person years, and an HIV prevalence of 15% in the population cohort of 2,500 per community, so 85% of the enrolled participants are HIV uninfected and are followed to measure HIV incidence. The between-community coefficient of variation of HIV incidence was assumed to be 0.15-0.20. The Protocol (v1.0) indicated that the study will be very well powered to detect an effect of 35% or larger in Arm A compared with Arm C, and moderately well powered to detect an effect of 30% under favorable assumptions. For the direct comparison of Arms A and B, the study will be well powered to detect a difference between effects of 60% and 30%, 55% and 25%, and 50% and 20%. The power calculations assume losses to follow-up of 20% over two years, and 25% over three years.

During the trial, power estimates will be updated based on information available from the PC0 and the modelled projections of effect size. These details will be provided in document that will be updated for each DSMB review when information changes.

Expected information that will require updating of power assumptions:

- Prevalence: The prevalence in PC0 will be available to assess the prevalence assumptions shortly after PC0 is complete.
- Between community variance of HIV incidence: the between-community variance of HIV prevalence is expected to be a good approximation to check this assumption. This will be available shortly after PC0 is complete.

- Arm C incidence: HIV incidence from the PC12 cohort can be used to assess the incidence assumption and assess the power assumptions of the study.
- Loss to follow-up: 20% over two years, and 25% over three years
- Effectiveness: Interim assessment of effectiveness by comparison of emerging data on HIV incidence is not planned. Because full intervention scale-up will not be achieved prior to PC12, HIV incidence comparisons at PC12 are unlikely to reflect a significant change. The HIV incidence from PC24 will be completed too late in the study to be useful as an interim assessment. Instead, the modelling group will estimate potential effectiveness using data from intervention uptake and PC0.

H.3 Interim Evaluation of Viral Load Suppression

The PopART test-and-treat intervention relies for its effectiveness on diagnosing a large proportion of HIV-infected individuals in the community, linking them to care, starting them on ART as soon as possible and maintaining high levels of retention, adherence and viral suppression. All the steps in this cascade are monitored continuously during the delivery of the intervention, primarily using process data. We will also monitor viral suppression in the *Population Cohort* across all study arms at PC12, where viral suppression at PC12 may provide an early warning of any major deficiencies in delivery so that corrective action could be taken at an early stage.

H.3.1 Expedited PC12 viral load testing and assessment of community viral load

Expedited PC12 viral load testing in the Population Cohort will be used as a monitoring tool to inform corrective action in the HPTN 071 (PopART) trial. At the PC12 visit, the majority of HIV-infected patients starting on ART since the start of the intervention in Arms A and B (including those starting outside national guidelines) will have been on ART for 3-12 months, allowing for the time needed for testing and linkage to take place. Data on viral suppression among these patients will provide an early indication of treatment adequacy. Carrying out the 12-month viral load testing, expedited to inform interim monitoring, will allow early corrective action to be taken if needed, while not requiring the addition of any further testing as it is already planned as a secondary trial outcome.

The main assessment of viral load suppression will occur at the PC24 follow-up when most patients will have been on ART for 1-2 years. For this reason, the trial protocol sets this time-point as the most relevant for assessing viral suppression and will conduct viral testing in *all* HIV-infected participants. The larger sample size at 24 months will provide greater power and precision. Expediting PC24 month viral load testing for more immediate availability of results is not proposed, since the complete results are unlikely to be available with enough time to deploy corrective action. For example, if viral load results are available within 3 months of the last PC24 specimen being collected, the final round of follow-up (PC36) will already be underway before any corrective action could be taken. However, if the PC12 survey were to indicate reasons for concern, expedited testing at 24 months could be reconsidered.

Note that as specified in the Expedited PC12 viral load testing plan, the protocol team leadership will have full access to the VL data set among the subset of participants who self-reported being on ART at PC0 or PC12.

H.3.2 ARV drug testing

An objective, biomedical measure of ARV drug use may be used to define “on ART”. An affordable and efficient assay that detects an array of ARV drugs is available at the LC and we propose using this in combination with self-report if resources are available. Assessments of the correspondence between self-report and detection of ARV in samples will be used to judge the accuracy, and thus the use, of self-report in analysis. Funding for this assessment is not yet secured, however options for use of this

biomarker are included in the SAP. If ARV testing is performed, it would likely be performed for participants who are HIV-infected at PC24, using samples from the PC24 visit.

H.3.3 PC12 VLS Cohort

A random sample of 100 participants from the PC0 HIV-infected cohort will be selected per community to achieve a target sample size for expedited testing at PC12 of 75 per community, allowing loss to follow-up between PC0 and PC12, inadequate samples, processing errors and other deficiencies.

Analysis cohorts:

1. ***PC0 HIV-infected***
   All sampled P0 HIV-infected participants with VL results. This larger cohort will have greater precision for overall rate of VLS
2. ***PC0 HIV-infected and PC12 self-reported HIV-infected on ARVs at PC0 or PC12 visit.***
   Option 1 (Self-report only): The subset of sampled PC0 HIV-infected participants who self-report current ARV use in PC0 and/or PC12. This cohort will be used to assess ART adherence.
   Option 2 (Self report or biomarker): The subset of sampled PC0 HIV-infected participants who either have ARV's detected in their plasma sample or self-report current ARV use in PC0 and/or PC12.

H.3.4 Blinding to interim viral load results

While the study team will remain blinded to overall community viral load, they will have access to the PC 12 VL data on those on ART, in order that corrective action can be taken if necessary.

H.3.5 Power for comparisons in PC12 expedited VLS

The table shows the expected sample size for viral load testing at each visit in Arm A, by country. It also shows the expected precision of the estimated proportion of patients virally suppressed, assuming an underlying rate of 90%. The table assumes that the main interest is in testing for viral suppression among patients who have been on ART at any time during the follow-up period, including those already on ART at baseline (assumed to be around 25% of HIV-infected on average) as well as those started on ART following the start of the intervention (assumed to be 70% of HIV-infected not already on ART at baseline).

The available sample size will provide a precise measure of PC12 viral suppression overall and for Zambia and S Africa separately. It will also give an early indication of gross under-performance in any one cluster. For example, if viral suppression in one cluster is as low as 60%, the precision on this estimate will be ± 12.6% so we would be able to say reliably that this cluster is not reaching a target of (say) 75% or 80%. The corresponding estimates are provided for Arms B and C. Because numbers on ART in each cluster will be somewhat lower than in Arm A, the precision of the viral suppression estimates will be correspondingly lower. However, we consider that these estimates are sufficiently precise for the purposes of this monitoring exercise.

Estimated sample size and precision for PC12 VLS

**Arm A**			
**Visit**	**HIV positive tested for VL**	**Number on ART**[1]	**Precision of proportion virally suppressed**[2]
12m<br>Per triplet<br>Total (7 triplets)	 75<br>525	 58<br>406	 ± 7.7%<br>± 2.9%

Zambia (4 triplets)<br>S Africa (3 triplets)	300<br>225	232<br>174	± 3.9%<br>± 4.5%
**Arm B**			
12m<br>Per triplet<br>Total (7 triplets)<br>Zambia (4 triplets)<br>S Africa (3 triplets)	 75<br>525<br>300<br>225	 44<br>308<br>176<br>132	 ±8.9 %<br>±3.4 %<br>±4.4 %<br>±5.1 %
**Arm C**			
12m<br>Per triplet<br>Total (7 triplets)<br>Zambia (4 triplets)<br>S Africa (3 triplets)	 75<br>525<br>300<br>225	 36<br>252<br>144<br>108	 ±9.8 %<br>±3.7 %<br>±4.9 %<br>±5.7 %

[1] Assumes 25% already on ART at baseline and 70% of those not on ART are diagnosed and started on ART

[2] 95% CI for proportion virally suppressed among those on ART, assuming estimated proportion is 90%. These CI do not account for clustering due to the community randomized design.

H.3.6 Ethical issues and anonymization

The protocol states that results from viral load testing in the *Population Cohort* will not be fed back to participants (e.g. if failure of viral suppression is found). Doing so would provide a different level of service for these PC patients and could distort the findings of the trial, leading to Hawthorne effects in the *Population Cohort*. Also, in most cases laboratory results will only be available after a long delay, diminishing the clinical relevance of these results. Local health authorities and implementing partners could still be informed of community trends which could facilitate efforts to explore and implement changes at the community service level, in order to benefit all patients in a given community.

Specimens could be anonymized (specimen identification (ID) number de-linked from PC ID number) before going forward for viral load testing. This would remove the ethical dilemma that would otherwise exist. However, delinking these expedited viral load data from other data on *Population Cohort* participants would preclude further statistical analysis of trends in viral load over time and related socio-demographic and other risk factors, and so we are not proposing this at this time.

I Statistical Analyses

This section describes the statistical analyses of the primary and secondary outcomes that will be conducted on enrolled participants in the population cohort and the analysis of the intervention data from the CHiPs

PC data is collected in all communities; thus, analyses include all three arms of the trial. CHiPs data is collected only in the interventions communities, thus analyses include only Arms A and B.

I.1 Descriptive Analyses

I.1.1 By community

Within each community, summary statistics (e.g., frequencies, percentages, means, medians, inter-quartile range, minima and maxima) that are appropriate to the measurement scale will be used to describe demographic, behavioral, HIV testing and medical history and ART use data. Continuous variables may be described using categorical levels chosen based on previous experience with similar studies; depending on the final distributions of these variables and providing it does not meaningfully impact the intent of analyses based on them, these categories may be modified prior to final report to allow better characterization of the relevant distributions.

I.1.2 By arm

Different descriptive summaries will be computed as appropriate to the analysis questions.

1. Participant aggregated: Summary statistics are computed treating the participants as a single group, i.e., for the ith community ($i = 1,2,3$) in the jth triplet (j = 1…7), with N_{ij} individuals ($k = 1 \ldots N_{ij}$), where we assume that i indexes the communities in arms A ($i = 1$), B ($i = 2$) and C ($i = 3$) respectively

$$\bar{y}_i = \frac{1}{\sum_j N_{ij}} \sum_{jk} y_{ijk}$$

   Standard errors and confidence limits will not be provided for participant aggregated summaries.

2. Community aggregated: Summary statistics are computed by summarizing the within community summaries by arm. E.g.

$$\bar{y}_i = \frac{1}{7} \sum_j \bar{y}_{ij} = \frac{1}{7} \sum_j \frac{1}{N_{ij}} \sum_k y_{ijk}$$

   Standard errors and confidence limits for community aggregate estimates will be computed using the within group sample variance of cluster means

$$S_i^2 = \frac{\sum_{j=1}^{7} \left(\bar{y}_{ij} - \bar{y}_{i.}\right)^2}{6}$$

Note: Participant and community aggregated summaries are expected to be similar when the number of people in each community (sub) population are similar. By the design of the study it is intended that the overall number of people in each community is similar.

3. Descriptive matched differences across arms: Summary statistics are computed within each community, and appropriate comparative statistics are computed within matched pairs and summarized across the 7 pairs. The computation is repeated for each pair of arms.

- Difference in means:

For each triplet and arms A (i = 1) and C (i = 3), for example: compute the jth triplet specific difference in means: $d_j^{AC} = \bar{y}_{1j} - \bar{y}_{3j}$; the average difference in means between arms is

$$D^{AC} = \frac{1}{7}\sum_{j=1}^{7} d_j$$

- Risk ratio
  For each triplet and arms A and C, for example: compute the triplet specific relative risk ratio: $r_j^{AC} = \frac{p_{1j}}{p_{3j}}$. The summary statistic is the geometric mean of the relative risks:

$$R^{AC} = \exp(\bar{s}^{AC}) = \exp\left(\sum s_j^{AC} / 7\right)$$

  where $s_j^{AC} = \log(r_j^{AC})$
- Rate ratio
  For each triplet: compute the triplet specific rate ratio: $r_j^{AC} = \frac{r_{1j}}{r_{3j}}$ where $\bar{r}_{ij} = \frac{1}{\sum_k t_{ijk}} \sum_k y_{ijk}$ with t_{ijk} = time on study and y_{ijk} = 1 if event is observed, 0 otherwise. The summary statistic is the geometric mean of the relative risks:

$$R^{AC} = \exp(\bar{s}^{AC}) = \exp\left(\sum s_j^{AC} / 7\right)$$

  where $s_j^{AC} = \log(r_j^{AC})$
- Odds ratios

  For each triplet and arms A and C, for example: compute the triplet specific odds ratio $r_j^{AC} = \frac{p_{1j}(1-p_{3j})}{p_{3j}(1-p_{1j})}$. The summary statistic is the geometric mean of the relative risks:

$$R^{AC} = \exp(\bar{s}^{AC}) = \exp\left(\sum s_j^{AC} / 7\right)$$

  where $s_j^{AC} = \log(r_j^{AC})$

I.2 Primary Analysis

I.2.1 HIV Incidence

Question: Does the PopART intervention reduce risk of HIV acquisition?
Endpoint: Incident HIV infection
Cohort: Primary Incidence Cohort
Secondary cohort: Full Incidence Cohort

Details:
Determination of incident HIV infections will be determined using site and LC HIV testing, with an algorithm followed for additional testing to confirm HIV infection status, as described in the ***Procedures for HPTN 071 primary endpoint review.*** Any cases with non-conforming test results will be adjudicated by the Virology Endpoint Adjudication Committee, using the ***HPTN 071 Adjudication plan***.
Incident HIV infection will be assessed approximately annually as each PC round is conducted and will be used as the primary endpoint for the intervention effect. Individuals who drop out of the study and

refuse further testing prior to completion of follow-up and individuals who die prior to completion of follow-up will be treated as uninformatively censored as of their last valid HIV status determination. Visits are only included if HIV status was assessed by study HIV testing. Participants enrolled in PC12N and PC24N, after the onset of the intervention, are assumed to contribute to assessment of intervention effectiveness in the same way as those enrolled in PC0.

Person years of follow-up are calculated as:

1. The time between each PC visit and the next subsequent visit (i.e. PC0 to PC12, PC12/PC12N to PC24 and PC24/PC24N to PC36) for participants who are HIV negative at their initial visit. Only visits where HIV status was determined are included.
2. The time between last HIV-negative visit and the estimated seroconversion date for participants who seroconverted. Estimated seroconversion date is defined as the date halfway between the last negative HIV test result and the first HIV infected test result where the first infected visit is not acute. If the first infected visit is acute, the estimated seroconversion date is defined as the observed visit date at which the acute infection was detected.
3. Each participant contributes person years for each pair of visits with HIV testing.

Seroconverters are participants in the cohort who were HIV uninfected by study testing at PC0, PC12, PC12N, PC24 or PC24N and who subsequently are HIV infected by study testing at PC12, PC24 or PC36. Any participant who has a known sample mix-up, evidenced by a visit with confirmed HIV-infection followed by a visit with confirmed HIV-negative status, is omitted from the analysis.

Age and Sex: Sex is assigned to each person as most recently reported during study follow-up. Age group is assigned to each record using a Lexis expansion for age-at-risk adjustment (i.e. age reassessed during each portion of an observed time period) , based on the reported or estimated birthdate, and the visit dates in each round for each participant.

Descriptive analysis: The number of events and total person years, HIV incidence rates and 95% confidence intervals will be presented for each community, with 95% confidence intervals based on the Normal approximation to the Poisson distribution. The same data will also be presented for each community for men and women separately; by age categories; by age for men and women.

The by arm summary will be computed by combining the incidence data for each community with a log transformation applied to reduce skewness. The geometric mean and associated 95% CI will be calculated for each of the three trial arms, and also separately for each arm by country. Unadjusted relative risk ratios, prevalence ratios, and rate ratios will be reported as the ratios of unadjusted geometric means across arms; confidence limits will be based on the residual MSE of an ANOVA with triplet and arm, on the log-transformed risks/prevalences/rates, using a *t* distribution statistic with 12 degrees of freedom. (Note: this is identical to the Step 2 analysis below, conducted on the community incidence rates.)

Analysis: A two-stage analysis will be used. In the first stage, we estimate the expected number of events in each community based on age, gender and triplet for all three arms simultaneously. In the second stage, we conduct a formal statistical comparison for differences in observed versus expected incidence by arm for the primary comparison between Arm A and C. A two-sided test with α = .05 will be used. Arm B versus C and Arm A versus B will also be compared, using a two-sided test with α = .05.

Analyses will not be weighted to account for the sampling design or the lost to follow-up (i.e. selection of one person per household and non-response). No adjustment is planned for multiple comparisons.

Details for two-stage analysis of incidence outcomes.

To compute incidence, the number of seroconversions in each community is divided by the person-years of follow-up for that community.

Stage 1:

1. Poisson regression will be used to adjust for confounding variables at the individual level. The model will be fitted using data from all three arms, prior to comparisons for specific arms under comparison. The regression model will include terms for the covariates of interest and triplet but not trial arm.

$$\log(\mathrm{I}_{ijk_m}) = \alpha_j + \beta h_{ij} + \sum_l \gamma_l z_{ijk_ml}$$

   where I_{ijk_m} is the indicator of incidence for the kth individual's m^{th} (partial) year (k = 1…N_{ij}) in the jth study triplet (j = 1…7) and i^{th} study arm (i = 1,2,3), h_{ij} is age and sex standardized baseline HIV prevalence in the i,j^{th} community and z_{ijk_ml} is l^{th} covariate.
   Individual level covariates to be used for adjustment are:
   a) Age during each portion of an observed time period in groups (18-24, 25-29, 30-34, 35-39, 40+) – Age category can change during each year of follow-up
   b) Sex
   c) Age x Sex interaction

   Community-level covariate to be used for adjustment is age and sex standardized HIV prevalence at baseline. The degrees of freedom in the paired t-test will be reduced by 1 to account for this covariate.

2. A fitted model will be used to obtain the ratio of observed-to-expected (O/E) events, and a log transformation will be applied to this ratio (the log ratio-residual). The expected number of events in the ith arm of the jth triplet is calculated as:

$$e_{ij} = \sum_{k_m} t_{ijk_m}\hat{\mathrm{I}}_{ijk_m} = \sum_{k_m} t_{ijk_m} \times \exp\left(\widehat{\alpha_j} + \hat{\beta} h_{ij} + \sum_l \widehat{\gamma_l}\, z_{ijk_ml}\right)$$

   Where t_{ijk_m} is the person years contributed in the mth (partial) year for the kth individual.

3. The adjusted rate ratio for the jth triplet for arms i_0 and i_1 is calculated as:

$$\widehat{\theta_j}(i_0, i_1) = \frac{d_{i_0j}/e_{i_0j}}{d_{i_1j}/e_{i_1j}}$$

   where d_{ij} is the observed number of events.

Stage 2:

1. Take the logarithm of the adjusted rate ratio:

$$h_j(i_0, i_1) = \log\left(\widehat{\theta_j}(i_0, i_1)\right)$$

   Take the mean over triplets to get the log intervention effect (the mean log adjusted rate ratio):

$$\bar{h}(i_0, i_1) = \sum h_j(i_0, i_1) / 7$$

The point estimate of the intervention effect comparing arms i_0 and i_1 is the geometric mean of the adjusted rate ratios $R_{i_0 i_1} = \exp\left(\bar{h}(i_0, i_1)\right)$

2. Conduct a two-way ANOVA on the log ratio-residuals, using triplet and arm, which is the three-arm extension of a paired t-test: The residual MSE, which has 11 degrees of freedom, is used to compute the empirical standard error of the log intervention effect (mean pairwise difference in log ratio-residuals) between arms

$$s_d = \sqrt{\frac{2 \times \text{MSE}}{7}}$$

3. Carry out paired t-test on pairwise log adjusted rate ratios

$$t(i_0, i_1) = \frac{\bar{h}(i_0, i_1)}{s_d}$$

   The p-value of $t(i_0, i_1)$ is assessed assuming a *t* distribution statistic with 11 degrees of freedom and will be used to assess the strength of evidence against the null hypothesis

4. Confidence interval for the log adjusted rate ratio are computed as:

$$\bar{h}(i_0, i_1) \pm t_{11,\ 0.025} \times s_d$$

Imputation of HIV infection assessment at PC12

A substantial proportion of the person years between PC12 and PC36 would be omitted because of the number of persons who do not have HIV status assessed at PC12 (either because of missed visits or because a specimen was not collected) but have known HIV status at a later visit (PC24 and PC36). Imputation methods will be used to include these person years and endpoints in the primary analysis. For participants who change serostatus, i.e. HIV-negative at PC0, missing results at PC12 and HIV-positive at PC24 (or also missing PC24 and HIV-positive at PC36), PC12 **HIV status** is imputed for the PC12 visit only. For HIV-negative participants, i.e. HIV-negative at PC0, missing PC12, HIV-negative at PC24 (or missing PC24 and HIV-negative at PC36), HIV serostatus is known to be HIV-negative. It is not necessary for the primary analysis to impute missing PC24 data, since having HIV status at PC12 and PC36 is sufficient to be included in the primary analysis. **Specimen collection date** for PC12 will be estimated as the mean relative proportion of time between visits for all persons from the same community with visits at PC0, PC12 and PC24.

For example, the specimen collection date, $t_{ijn}^{(12)}$ for the nth participant in arm i, triplet j is estimated as:

$$t_{ijn}^{(12)} = t_{ijn}^{(0)} + \frac{\bar{t}_{ij.}^{(12)} - \bar{t}_{ij.}^{(0)}}{\bar{t}_{ij.}^{(24)} - \bar{t}_{ij.}^{(0)}}\left(t_{ijn}^{(24)} - t_{ijn}^{(0)}\right)$$

where $\bar{\mathrm{t}}_{\mathrm{ij.}}^{(\mathrm{k})}$ is the mean specimen collection date for all such participants in arm i, triplet j and visit k.

These are the patterns where imputation will occur:

a) Seroconverters

PC0	PC12	PC24	PC36
N	.	P	P

N	.	P	.
N	.	.	P

b) Negatives

N	.	N	N
N	.	N	P
N	.	N	.
N	.	.	N

The sero-status imputation method is an "adjustment cells hot-deck" approach. For any participant needing sero-status imputation, we select a "donor" at random with replacement from the adjustment pool, defined as all participants from a) the same community (therefore the same arm) and sex b) not missing PC12 HIV status and c) with testing that matches the observed test results of the participant, excepting the missed PC12 visit. HIV status at PC12 is assigned using the donor value. Visit time is a cell mean imputation, computed in two steps: 1) the mean fractional time between visits is computed for the corresponding community 2) visit time for PC12 is computed using that mean fraction time between the observed participant visits.

Primary analysis, with imputation of missing PC12 data

Twenty imputation data sets will be constructed. The two-step primary analysis will be repeated for each dataset, producing 20 estimates of the logarithm of the adjusted rate ratios for each pair of arms and the corresponding ANOVA based MSE. The primary analysis estimates will be the average of the m = 1...20 estimates over the imputation datasets:

$$\hat{h}_{\text{imp}}(i_0, i_1) = \frac{1}{M}\sum_{m=1}^{M} \hat{h}_{\text{m}}(i_0, i_1)$$

Where $\hat{h}_{\text{m}}(i_0, i_1)$ is the log intervention effect (of Arm i_1 compared to Arm i_0) for the mth imputation. The variance, $\hat{\text{V}}_{\text{imp}}$, for the primary analysis test statistic will combine the variance for each of the 20 imputed datasets, and the variation in the adjusted rate ratio across the 20 imputed datasets, using standard imputation methods. That is, for M imputations

$$\hat{\text{V}}_{\text{imp}} = \hat{U}_M + \frac{M+1}{M}\hat{B}_M$$

where

$\hat{U}_M = \frac{1}{M}\sum_{m=1}^{M} \hat{U}_m$ = within-imputation variance of the pairwise comparisons across arms, where $\hat{U}_m = \frac{2}{7}MSE_m$ for the mth imputation, and

$$\hat{B}_M = \frac{1}{(M-1)(3-1)}\sum_{m=1}^{M}\Big(h_m(1,2) - h_{.}(1,2) - h_m(.,.) + h_{.}(.,.)\Big)^2 + \Big(h_m(1,3) - h_{.}(1,3) - h_m(.,.) + h_{.}(.,.)\Big)^2 + \Big(h_m(2,3) - h_{.}(2,3) - h_m(.,.) + h_{.}(.,.)\Big)^2$$

= between imputation variability in the estimates of intervention effects. (Notation: "." Implies average over that index, so $h_{.}(1,2)$) is an average over all the imputation estimates of the intervention effect for arms 1 and 2, and $h_m(.,.)$is an average over the three pairwise estimates for imputation m.) This is essentially the MSE of a two-way ANOVA of M imputations with factors imputation and pairwise comparison.

Following the primary analysis above, the paired t-test on log adjusted rate ratios for arms $i_0, i_1$is:

$$t_{imp}(i_0, i_1) = \frac{\hat{h}_{\text{imp}}(i_0, i_1)}{\sqrt{\hat{V}_{\text{imp}}}}$$

The p-value of $t_{imp}(i_0, i_1)$ is assessed assuming a *t* distribution statistic with 11 degrees of freedom and will be used to assess the strength of evidence against the null hypothesis.

I.2.2 Subgroup analysis

The analysis of HIV risk will be conducted for the following subgroups:

1. Men and women
2. Person years for younger and older (<25, ≥25 yo)
3. Person years from Younger men, Older men, Younger women, Older women

The analysis approach will follow the primary analysis of HIV risk.

Treatment of zeros

If in any subgroup analysis there is a community with 0 eligible participants, the triplet with that community will be omitted from the analysis.

If there is a community with at least one eligible participant, but 0 events, 0.5 will be added to the number of events and the number of participants for all communities in that triplet at the first stage of the analysis.

I.2.3 Supportive analyses

1. **Entire Intervention Period**

   The primary analysis will be repeated using all the follow-up time in the Full Incidence Cohort (i.e. including the first year of the PC follow-up).

2. **Intervention effect by Year**

   Intervention effectiveness will be assessed for each year of PC follow-up. Imputation will be used at both PC12 and PC24 for participants with a prior HIV-negative assessment, who did not have an HIV status assessed at PC12 and/or PC24.

3. **Complete case analysis, excluding participants not assessed for HIV in PC12**

   A complete case analysis will be reported, that excludes all person years and events that occur with no direct assessment of HIV status at PC12, that is, without the use of imputation.

4. **Permutation test**

   Evidence for the intervention effectiveness will also be assessed using a non-parametric permutation test. The analysis above will be recomputed for all possible randomizations that were computed at the beginning of the trial under the restricted randomization scheme. The number of allocations n for which the incidence rate ratio is as extreme as the value observed in the trial will be counted and a 2-sided p-value will be calculated as n divided by the total number of possible allocations.

I.3 Interim Monitoring Analysis

I.3.1 Viral Load Suppression

Question: For HIV-infected participants on ART, is the proportion virally suppressed lower in Arms A and B compared to C?

Question: For HIV-infected participants is the proportion virally suppressed high, and is it higher in Arm A and B compared to C?

Endpoint: HIV plasma viral load is suppressed (PVL < 400 copies/mL)
Cohort:

1. Random subset of PC12 samples selected from the PC0 HIV-infected Cohort
2. Subset of above restricted to HIV-infected on ART by self-report at either PC0 or PC12 (or lab-based assay, if funding permits)

Descriptive analysis: For each cohort, the number in the cohort, the number and proportion virally suppressed will be reported for each community, and for each arm (community-aggregated), with 95% confidence limits for community aggregated summaries as previously defined. The characteristics of (log) viral load in each community will be presented: mean, median, SE, range

The by arm summary will be computed by combining the individual proportion or mean for each community with a log transformation applied to the plasma viral load values to reduce skewness. The geometric mean and associated 95% CI will be calculated for each of the three trial arms, and also separately for each arm by country.

Analysis: The statistical analysis for both questions compares the proportion virally suppressed using an unadjusted cluster-based analysis of the relevant cohort selected for evaluation.

1. The prevalence ratio for each triplet is calculated as:
$$r_j(i_0, i_1) = \frac{p_{i_0 j}}{p_{i_1 j}}$$
where p_{ij} is the observed prevalence of events in the jth triplet for arm i.
2. Take the logarithm of the prevalence ratios:
$$s_j(i_0, i_1) = log\left(r_j(i_0, i_1)\right)$$

The point estimate of the intervention effect comparing arms i_0 and i_1 is the geometric mean of the prevalence ratios $R_{i_0 i_1} = \exp\left(\bar{s}(i_0, i_1)\right) = \exp\left(\sum s_j\,(i_0, i_1)/_7\right)$

3. Compute a confidence interval for the mean log prevalence ratio:
$$\bar{s}(i_0, i_1) \pm t_{12,\,0.025} \times s_d$$
where s_d is the empirical standard error for mean pairwise difference based on the MSE of the two-way ANOVA of the (log) prevalence ratios from the 21 communities fitted by triplet and arm.

I.4 Secondary Objectives: Population Cohort

I.4.1 Community Viral Load at PC24

Question: Does the PopART intervention reduce community viral load?

Endpoint: HIV plasma viral load is suppressed (PVL < 400 copies/mL)
Cohort: PC24 HIV-infected Cohort

Descriptive analysis: The number in the cohort, and the number and proportion virally suppressed will be reported for each community, and for each arm (participant-aggregated), with 95% confidence limits based on the binomial distribution. The characteristics of (log) viral load in each community will be presented: mean, median, SE, range.

The by arm summary will be computed by combining the proportion or mean for each community with a log transformation applied to reduce skewness. The geometric mean and associated 95% CI will be calculated for each of the three trial arms, and also separately for each arm by country.

Analysis: A two-stage analysis will be used. In the first stage, we estimate the expected proportion with viral suppression in each triplet and arm for all three arms simultaneously. In the second stage, we conduct a formal statistical comparison for the primary comparison between Arm A and C. A two-sided significance test, and the corresponding 95% confidence interval will be used to assess the evidence against the null hypothesis. Significance tests and 95% confidence limits will also be presented for Arm B versus C and Arm A versus B.

Details for two-stage analysis of proportions

The proportion virally suppressed in each community is the proportion in the cohort with PVL < 400.

Stage 1:

1. Logistic regression will be used to adjust for confounding variables at the individual level. The regression model will include terms for the covariates of interest and triplet but not trial arm.

$$\mathrm{logit}(y_{ijk}) = \alpha_j + \sum_l \gamma_l \, z_{ijkl}$$

   where y_{ijk} is the binary indicator of suppressed viral load, *i* is i^{th} study arm (*i* = 1,2,3), *j* is j^{th} study triplet (j= 1…7), *k* is k^{th} individual (*k* = 1…N_{ij}) and z_{ijkl} is the covariate value for the lth covariate. Individual level covariates to be used for adjustment are:
   a) Age at PC24 visit in groups (18-24, 25-29, 30-34, 35-39, 40+)
   b) Sex
   c) Age x Sex interaction
2. A fitted model will be used to obtain the ratio of observed (O) to expected (P) proportions, and a log transformation will be applied to this ratio-residual. The expected proportion in the ith triplet of the jth study arm is calculated as:

$$p_{ij} = \sum_k \hat{y}_{ijk} = \sum_k \frac{\exp\left(\hat{\alpha}_j + \sum_l \hat{\gamma}_l \, z_{ijkl}\right)}{1 + \exp\left(\hat{\alpha}_j + \sum_l \hat{\gamma}_l \, z_{ijkl}\right)}$$

3. The ratio-residual for the jth triplet of the ith study arm is calculated as:

$$R_{ij} = \frac{o_{ij}}{p_{ij}}$$

   where o_{ij} is the observed proportion of events in the ith arm of the jth triplet. The adjusted risk ratio for each triplet for arms i_0 and i_1 is calculated as:

$$\rho_j(i_0, i_1) = \frac{R_{i_0 j}}{R_{i_1 j}}$$

Stage 2:

4. Take the logarithm of the adjusted risk ratios:

$$d_j(i_0, i_1) = \log\left(\rho_j(i_0, i_1)\right)$$

The point estimate of the intervention risk ratio comparing arms i_0 and i_1 is the geometric mean of the adjusted risk ratios $R_{i_0 i_1} = \exp\left(\bar{d}(i_0, i_1)\right) = \exp\left(\sum d_j(i_0, i_1) / 7\right)$

5. Conduct a two-way ANOVA on the log ratio-residuals, using triplet and arm, which is the three-arm extension of a paired t-test: The residual MSE, which has 12 degrees of freedom, is used to compute the empirical standard error of the log intervention effect (mean pairwise difference in log ratio-residuals)

$$s_d = \sqrt{\frac{2 \times \mathrm{MSE}}{7}}$$

6. Carry out paired t-test on differences in mean log adjusted risk ratio,

$$t(i_0, i_1) = \frac{\bar{d}(i_0, i_1)}{s_d}$$

The computed value of $t(i_0, i_1)$ is compared to a *t* distribution statistic with 12 degrees of freedom.

7. The confidence interval for the mean log adjusted risk ratio is computed as:

$$\bar{d}(i_0, i_1) \pm t_{12,\,0.025} \times s_d$$

This will be back transformed and reported as an adjusted risk ratio.

I.4.1.1 <u>Subgroup analyses</u>

The analysis of proportion virally suppressed in the community will be conducted for the following subgroups (for each subgroup, two-stage analysis would no longer be adjusted for that subgroup):

a. Men and women
b. Younger and older (<25, ≥25 yo)
c. Younger men, Older men, Younger women, Older women
d. Amongst persons who do/do not self-report HIV+, and those who SR HIV+ and current ART use at PC24.

I.4.2 Viral suppression and Resistance

Question: Does the PopART intervention affect the probability of viral suppression amongst people who self-report being currently on ART or ever being on ART?

<u>Endpoint</u>: Viral load suppressed (<400 copies/mL)

<u>Cohort</u>: Community viral load subset PC24: The subset of visits from the PC24 community viral load cohort, restricted to specimens collected from participants who self-report current ART at 24 months. A second analysis will examine viral load suppression in participants who ever report ART use up through PC24.

Analysis: The analysis approach will be the same as used for the two-stage analysis of proportions (see Community Viral Load objective)

Question: Does the PopART intervention affect the probability of ART resistance at 24 months amongst people who initiate ART after the commencement of PopART, among those not virally suppressed at 24 months?

Endpoint: Resistance to drugs used in current first or second line ARV treatment regimens

Cohort: Subset of community viral load assessed at PC24, restricted to those with VL > 400:

Details: A subset of visits from the PC24 community viral load cohort where VL exceeds 400 copies/ml will be assessed for ARV resistance, restricted to participants who self-report any initiation of ART up through PC24 after commencement of PopART.

Analysis: The analysis approach will use the two-stage approach for proportions.

Question: Does the PopART intervention affect the probability of ART resistance amongst PC seroconverters?

Endpoint: Resistance to drugs used in current first and second line ARV treatment regimens

Cohort: Seroconverter cohort

Analysis: The analysis approach will use the two-stage approach for proportions.

I.4.3 Viral Burden in the community

Question: Does the PopART intervention decrease the viral burden in the community?

It has been established that persons with undetectable viral load are not infectious, thus HIV incidence is likely predicted by the proportion of the community with detectable viral load.

Endpoint: Viral burden is defined as

= 0 if HIV-uninfected, or if HIV-infected with VL < 400 copies/ml

= 1 If HIV-infected with unsuppressed viral load (>=400 copies/mL)

Cohort: PC24

Analysis: The analysis approach will be the same as used for the two-stage analysis of proportions (see Community Viral Load objective)

Details: All persons with HIV status evaluated at PC24 visit are included.

I.4.4 HSV2 Incidence

Question: Does the PopART intervention change HSV-2 incidence?

Cohort: PC, restricted to the subset of participants who are not infected with HSV-2 at PC0.

Endpoint: HSV-2 status at PC36

Analysis: As HSV-2 acquisition is only assessed once, at PC36, HSV2 acquisition will be assessed as a binary outcome, rather than as a survival analysis outcome. The analysis approach will use the two-stage analysis for proportions (see the analysis of community viral load). HSV-2 acquisition is considered to be both a marker of sexual activity and a risk factor for HIV acquisition.

Individual covariates included in adjustment:

a. Age in groups (18-24, 25-29, 30-34, 35-39, 40+)
b. Sex
c. Interaction of Sex and Age

I.4.5 HIV Testing

Question: How much did PopART increase recent knowledge of HIV-Status (within the past year) through HIV testing and retesting in each arm, in each year of the study.

Cohort: PC

Endpoint: Self-reported knowledge of HIV status by recent testing.

Details: All enrolled participants with study assessed HIV status will be included. Analysis will also evaluate knowledge separately for HIV infected (see below).

Self-report of HIV status and recent testing is recorded in PC0, PC12, PC24 and PC36. A known a priori caveat for this analysis is that all participants in the PC are offered rapid HIV testing by the research enumerators at each PC visit, thus uptake of testing may be higher in the PC participants than the general population, particularly in Arm C.

PopART is expected to increase the proportion of persons with recent, accurate knowledge of HIV status. Accurate knowledge of HIV status has four mutually exclusive categories, described below. The binary endpoint for assessing the PopART effect is Known vs Unknown

Among HIV-infected:

- Known; Self-reported HIV-infected
- Unknown (refused to answer; has never been tested; did not self-report HIV-infected)

Among HIV uninfected

- Known; Self-reported HIV uninfected, testing in past year
- Unknown; No testing in past year, refused to answer; has never been tested.

Descriptive: Number and proportion for knowledge of HIV status will be reported over time in each community and for each arm (participant-aggregated), with 95% confidence limits based on the binomial distribution, adjusted for within community correlation.

Analysis: Estimates of the relative decrease (i.e. risk ratio) within a pair of study arms will use the two-stage analysis method described for proportions (see Community Viral Load) for each study round.

1. Logistic regression will be used to adjust for confounding variables at the individual level. The regression model will include terms for the covariates of interest and triplet but not trial arm.

$$\text{logit}(y_{ijk_m}) = \alpha_j + \sum_{l,m} \gamma_l \, z_{ijk_m l}$$

where y_{ijk} is the binary indicator of self-reported HIV-positive status for the kth (k = 1…N_{ij}) individual's m^{th} year, i is i^{th} study arm (i = 1,2,3), j is j^{th} study triplet (j= 1…7), and $z_{ijk_m l}$ is the covariate value for the lth covariate.

Individual level covariates to be used for adjustment are:

a) Age at visit in groups (18-24, 25-29, 30-34, 35-39, 40+)
b) Sex
c) Age x Sex interaction

Note: No adjustment is currently planned for community level covariates. If adjustment for a community level covariate is included, the degrees of freedom in the paired t-test will be reduced by 1.

2. A fitted model will be used to obtain the ratio of observed (O) to expected (P) proportions, and a log transformation will be applied to this ratio-residual. The expected proportion in the *j*th triplet of the *i*th study arm in the *m*th year is calculated as:

$$p_{ij}{}^{m} = \sum_{k} \hat{y}_{ijk_m} = \sum_{k} \frac{\exp\left(\hat{\alpha}_j + \sum_l \hat{\gamma}_l\, z_{ijk_m l}\right)}{1 + \exp\left(\hat{\alpha}_j + \sum_l \hat{\gamma}_l\, z_{ijk_m l}\right)}$$

3. The ratio-residual for the *j*th triplet of the *i*th study arm in the *m*th year is calculated as:

$$R_{ij}{}^{m} = \frac{o_{ij}{}^{m}}{p_{ij}{}^{m}}$$

where $o_{ij}{}^{m}$ is the observed proportion of events in the *i*th arm of the *j*th triplet in the *m*th year. The adjusted risk ratio for each triplet for arms i_0 and i_1 (omitting the year subscript) is calculated as:

$$\rho_j(i_0, i_1) = \frac{R_{i_0 j}}{R_{i_1 j}}$$

Stage 2:

4. Take the logarithm of the adjusted risk ratios:

$$d_j(i_0, i_1) = \log\left(\rho_j(i_0, i_1)\right)$$

The point estimate of the intervention risk ratio comparing arms i_0 and i_1 is the geometric mean of the adjusted risk ratios $R_{i_0 i_1} = \exp\left(\bar{d}(i_0, i_1)\right) = \exp\left(\sum d_j(i_0, i_1) / 7\right)$

5. Conduct a two-way ANOVA on the log ratio-residuals using triplet and arm, which is the three-arm extension of a paired t-test: The residual MSE, which has 12 degrees of freedom, is used to compute the empirical standard error of the log intervention risk ratio (mean pairwise difference in log ratio residuals between arms)

$$s_d = \sqrt{\frac{2 \times \mathrm{MSE}}{7}}$$

6. Carry out paired t-test on log adjusted risk ratio ,

$$t(i_0, i_1) = \frac{\bar{d}(i_0, i_1)}{s_d}$$

The computed value of $t(i_0, i_1)$ is compared to a *t* distribution statistic with 12 degrees of freedom.

7. The confidence interval for the log adjusted risk ratio is computed as:

$$\bar{d}(i_0, i_1) \pm t_{12,\,0.025} \times s_d$$

This will be back transformed and reported as an adjusted risk ratio.

Question: How much did PopART increase accurate knowledge of HIV-infected status through HIV testing and retesting in each arm, in each year of the study? (First 90)

Cohort: PC. For each year, all participants assessed with HIV-infected status by study testing that year are included

Endpoint: Accurate knowledge of HIV-infected status.

Details: All enrolled participants who are assessed as HIV-infected are included.

Knowledge of true HIV-infected status has 3 mutually exclusive categories

- Self-reported HIV-infected
- Tested in last year, did not SR HIV+
- Did not test in last year, did not SR HIV+

The binary endpoint for assessing the PopART intervention is Accurate knowledge of HIV-infected status, i.e. HIV+ who SR-HIV+ and Not SR-HIV+ (remaining categories). PopART is expected to increase the proportion of persons with "Accurate knowledge of HIV-infected status".

Descriptive: Number and proportions in each of the 3 categories and classified as Accurately known will be reported for each year in each community and for each arm (participant-aggregated), with 95% confidence limits based on the binomial distribution, adjusted for within community correlation.

Analysis: Estimates of the relative increase within a pair of study arms will use the two-stage analysis method described for proportions (see Community Viral Load) for each study round separately.

I.4.6 Retention in care

Question: Does the PopART intervention increase retention in HIV care in each community?

Cohort: Self-reported HIV-infected cohort, restricted to participants who report having never registered for care

Endpoint: HIV care visit within the past three months

PopART is expected to increase the proportion of persons retained in care (i.e. with HIV care visit within the past 3 months).

Descriptive: Number and proportion will be reported for each year in each community and for each arm (participant-aggregated), with 95% confidence limits based on the binomial distribution, adjusted for within community correlation.

Analysis: Estimates of the relative increase within a pair of study arms will use the two-stage analysis method described for proportions for each study round separately.

I.4.7 Viral Load Suppression

Question: Does the PopART intervention increase viral load suppression in each community at PC12 and PC36?

Cohort: PC12 and PC36 community viral load

Endpoint: Plasma Viral Load

Details: Viral load is assessed in a random sample of 75 HIV-infected participants in each of PC0, PC12, and PC36. See also Interim Analysis for PC12.

Descriptive: Number and proportion virally suppressed will be presented in each community and each year. The characteristics of (log) viral load in each community will be presented: mean, median, SE, range.

Analysis: A paired t-test on the unadjusted pairwise rate ratios for each year will be used to test for change in proportion virally suppressed in each pair of arms across triplets for PC12 and PC36 separately.

I.4.8 ART screening and uptake (Second 90)

Question: What are the proportions of HIV-infected [with known status] on ART in each arm, in each year of the study?

Question: How much does PopART increase ARV treatment?

Cohorts:

1) HIV-infected Cohort (PC0, PC12, PC24, PC36)
2) Self-reported HIV infected cohort

Endpoint: Self-reported ART

PopART is expected to increase the proportion reporting ART amongst HIV infected.

Details: ART is only assessed in participants who self-report HIV-infected status and can be self-reported and/or verified by ART documents presented by the participant. We will examine the increase in treatment amongst 1) HIV-infected participants identified in study testing and 2) participants who self-identify as HIV-infected. The same analysis approach will be used as for retained in care.

Descriptive: Proportions will be tabulated for all communities each year for each arm (participant-aggregated), for each analysis with 95% confidence limits based on the binomial distribution.

Estimates of the relative increase within a pair of study arms each year will use the analysis method described for proportions (see Community Viral Load).

I.4.9 HIV diagnosis and initiation of care

Question: For person newly diagnosed with HIV in the prior year, what proportion are linked to care within 6 months in each arm, in each year of the study?

Cohort: Combined subsets of PC0, PC12, PC24, PC36, restricted to persons who self-report first diagnosis of HIV-infection in the prior year.

Endpoint: Registered for HIV Care within 6 months

Details: Registering for HIV care is only assessed in those who self-report HIV-infected status and can be self-reported and/or verified by documents presented by the participant. Registering for care will be assessed using both the PC visit where diagnosis is reported and the subsequent PC visit. We will examine registered for HIV Care and time to registration amongst participants who self-identify as HIV-infected.

Description: Proportions registered within 6 months will be tabulated for all communities each year.

Analysis: Estimates of the relative increase within a pair of study arms in each Follow-up round will use the analysis method described for proportions (see Community Viral Load) in the follow-up years.

I.4.10 Sexual risk behavior

Question: Does the PopART intervention change sexual behaviors?

The PopART intervention does not directly target change in sexual behavior, although an increase in knowledge of HIV may result in change in sexual risk, for example as a result of risk disinhibition due to availability of treatment, effects due to behavioral counseling by CHiPs and condom promotion, and increased knowledge of HIV status throughout the community (including in discordant partners).

Endpoints: The following sexual behaviors will be examined separately for men and women:

- More than one sexual partner in the prior year

- Any casual/one-time sex partners in the last year
- Unprotected sex the last time.
- In the extended sexual behavior questionnaire:
    - Any concurrent sexual partners
    - Inconsistent or no condom use with any partners
    - Any sex for money/or gifts
    - Any known HIV discordant relationships

Cohort:

1) PC, by gender
2) Risk subgroups of the PC, by gender

Details: Detailed partner by partner questions about sexual risk behavior are only asked in the 20% Risk subgroup of the PC. Analyses of sexual behavior will all be sex specific.

Descriptive: Proportions reporting each sexual behavior will be tabulated for all communities and by arm (participant aggregated) each year.

Analysis. Estimates of the relative increase within a pair of study arms each year will use the analysis method described for proportions (see Community Viral Load) in the follow-up years.

Subgroups:

- By HIV status at visit as determined by study testing
- By self-reported HIV status at visit
- Age group

I.4.11 HIV disease progression and death

Disease progression not assessed in PC. A detailed plan to analyze a limited set of clinical data assessing HIV disease progression and death for PC participants who consented to clinical data access will be developed at a later date in a separate document.

I.4.12 ART toxicity based on clinic records

ART toxicity not currently assessed in PC. We continue to work to obtain data from clinics to assess ART toxicity.

I.4.13 Case notification rate of tuberculosis

Case notification of tuberculosis not currently assessed in PC. We continue to work to obtain data from clinics to assess TB case notifications, however the primary assessment of changes in TB case notification will be measured using clinic notification data, and based on linkage of PC data to clinic data for individuals who self-report they have taken treatment for TB in the previous 12 months (with linked clinic data being used to determine if an individual had bacteriologically confirmed TB).

I.4.14 HIV-related stigma

Question: Does PopART change HIV Stigma?

Cohort:

1) PC stigma subset

2) Self-Reported HIV-infected cohort

Endpoint:

1) HIV-related Stigma Scores
2) HIV-infected Stigma Scores

Details: HIV Stigma is assessed on a randomly selected 20% sample of the PC participants in PC0, PC12, PC24 and PC36 using 12 questions measured using a 4-point Likert scale. Experience of HIV stigma using a different set of 12 questions measured on a Likert Scale is measured in all self-identified HIV-positive participants.

Analysis: Mean stigma scores and binary indicators of stigma, developed by the Stigma Working Group, will be reported for each community and each year. Estimates of the relative change within a pair of study arms each year will use the two-stage analysis method for comparing adjusted community means and prevalences within each triplet described below

Subgroups. HIV Stigma effects will be assessed in the following subgroups

- Sex
- Country
- Age

Analysis: Estimates of the mean change within a pair of study arms will use the two-stage analysis method described for means for each study round.

Stage 1.

1. Linear regression will be used to adjust for confounding variables at the individual level. The regression model will include terms for the covariates of interest and triplet but not trial arm.

$$y_{ijk_m} = \alpha_j + \sum_{l,m} \gamma_l \, z_{ijk_m l}$$

where y_{ijk} is the stigma score for the *k*th (k = 1…N_{ij}) individual's *m*th year, *i* is *i*th study arm (*i* = 1,2,3), *j* is *j*th study triplet (j= 1…7), and $z_{ijk_m l}$ is the covariate value for the *l*th covariate.

Individual level covariates to be used for adjustment are:

a) Age at visit in groups (18-24, 25-29, 30-34, 35-39, 40+)
b) Sex
c) Age x Sex interaction

Note: No adjustment is currently planned for community level covariates. If adjustment for a community level covariate is included, the degrees of freedom in the paired t-test will be reduced by 1.

2. A fitted model will be used to obtain the difference of observed (O) to expected (P) means. The expected mean in the *j*th triplet of the *i*th study arm in the *m*th year is calculated as:

$$p_{ij}{}^{m} = \sum_k \hat{y}_{ijk_m} = \sum_k \hat{\alpha}_j + \sum_l \hat{\gamma}_l \, z_{ijk_m l}$$

3. The difference residual for the *j*th triplet of the *i*th study arm in the *m*th year is calculated as:

$$R_{ij}{}^{m} = o_{ij}{}^{m} - p_{ij}{}^{m}$$

where $o_{ij}{}^{m}$ is the observed means in the *i*th arm of the *j*th triplet in the *m*th year.

Stage 2:

4. The point estimate of the intervention effect is the mean adjusted difference for each triplet for arms i_0 and i_1 (omitting the year subscript), calculated as:

$$\bar{d}_j(i_0, i_1) = {R_{i_0 j}}^m - {R_{i_1 j}}^m$$

5. Conduct a two-way ANOVA on the difference residuals, using triplet and arm, which is the three-arm extension of a paired t-test: The residual MSE, which has 12 degrees of freedom, is used to compute the empirical standard error of the intervention effect (mean pairwise difference in different residuals between arms)

$$s_d = \sqrt{\frac{2 \times \text{MSE}}{7}}$$

6. Carry out paired t-test on mean pairwise differences,

$$t(i_0, i_1) = \frac{\bar{d}(i_0, i_1)}{s_d}$$

The computed value of $t(i_0, i_1)$ is compared to a *t* distribution statistic with 12 degrees of freedom.

7. The confidence interval for the mean adjusted difference is computed as:

$$\bar{d}(i_0, i_1) \pm t_{12,\,0.025} \times s_d$$

I.4.15 Medical male circumcision (MMC)

Question: What proportion of uncircumcised [self-reported] HIV-uninfected men received MMC during the PopART intervention by arm overall and by year?

Question: How much did the PopART intervention increase MMC among [self-reported] HIV-uninfected men?

Cohort

1) Self-reported HIV-uninfected men: The PC, restricted to men in the PC who self-report not having had MMC at enrollment and did not self-report HIV-infected status.
2) HIV-uninfected men cohort: All men in the PC who self-report not having had MMC at enrollment and were HIV-uninfected by study testing.

Endpoint: Overall: Proportion of the cohort with MMC occurring after the initiation of the PopART intervention. Each year: the proportion reporting MMC within the previous year, assessed each year.

Analysis: Proportion of new MMC will be reported for each community for each year.

PopART is expected to increase the proportion of HIV uninfected men who receive MMC. Estimates of the relative change in MMC in within a pair of study arms each year will use the two-stage method for comparing community proportions, i.e., a t-test for the within triplet adjusted proportions.

It is known that the baseline prevalence of traditional male circumcision is much higher in South Africa than Zambia: If the baseline prevalence of male circumcision is not well balanced in each triplet, this may be used as a community level covariate.

Subgroups:

- Country
- Age

I.5 Intervention Delivery Objectives: CHiPs data

The CHiPs data will be used to measure participation of households and individuals in the CHiP intervention, and uptake of key components of the intervention. Data are available for all 7 Arm A and all 7 Arm B communities, but not for Arm C.

We have aligned how uptake of the intervention will be measured using CHiPs data, with how it will be measured using Population Cohort data, as far as possible. However, a fundamental difference between the PC and the CHiPs intervention, is that in the PC all participants consent to give a blood sample for laboratory HIV testing, so that HIV status is known for close to 100% of PC participants. In contrast, in the CHiPs intervention individuals can choose to participate but choose not to accept HIV testing using a rapid HIV test. This means that, among individuals who consent to participate in the CHiPs intervention, HIV status is known for individuals who self-report they are HIV-infected, or accept the offer of rapid HIV testing, or report they have recently tested for HIV and the test result was HIV-negative (for example in the previous 12 months); and HIV status is unknown for all other individuals.

All of the main outcomes will be summarised as proportions. Two examples are:

(1) the proportion of individuals who know their HIV status, among all individuals who consent to participate in the intervention [see I.4.4 for measurement in the PC]
(2) the proportion of HIV-infected individuals who link to HIV care within 6 months of referral to HIV care, among all who were referred to HIV care by CHiP teams and were not on ART at the time of referral, derived from a "time to event" analysis [see I.4.8 for measurement in the PC].

The general approach to data analysis will be to summarise data:

1. separately for each community
2. average value across the 14 intervention communities
3. average values for each country
4. average values for each trial arm
5. average values for each trial arm, within each country

Average values will be calculated as the average of the percentage values from each of the communities, as well as average values based on individual-level data (giving each individual the same weight in the analysis, rather than giving each community the same weight).

For almost all intervention components, uptake is expected to be similar in Arms A and B. The exception is ART uptake, because of the difference between the two trial arms for ART eligibility criteria up to the date when all Arm B communities transition to offering universal treatment. Thus in summarizing uptake of the intervention, the emphasis will be on summaries 1, 2, 3, 4 and 5 above. For ART uptake, time to start ART after referral to HIV care, and retention on ART the emphasis will be on summaries 1, 4, and 5.

There will be 3 rounds ("years") of delivering the CHiPs intervention. Uptake of all intervention components will be summarized separately for each round.

As well as overall summaries, subgroup analysis will also be presented by gender, age group, by age group for each gender, and according to participation and residency in earlier rounds of the

intervention. Sub-group analyses beyond those by age group, gender, and prior participation and residency, are specified separately if applicable.

The analysis for six outcomes that are measured directly using the CHiP data is set out below in detail, chosen on the basis that they are among the most important intervention components. The six outcomes are knowledge of HIV status, the time to link to HIV care after referral to HIV care, ART uptake, retention in HIV care, retention on ART, and uptake of MMC. Various other outcomes will also be measured, including for example the proportion of households that participate in the intervention, the proportion of individuals that participate in the intervention, the uptake of re-testing for HIV after a previous HIV-negative test result, and the time to start ART after referral to HIV care in Arm A communities. In addition, we set out our approach to estimating coverage against the UNAIDS "90-90" targets that 90% of HIV-positive individuals know their HIV-positive status, and that 90% are on ART among those who know their HIV-positive status.

As well as the descriptive summaries outlined above, we will formally compare key outcomes between Arm A and Arm B, using the same 2-stage method of analysis as has been outlined above for the analysis of PC data. In the case of the intervention data, all outcomes are measured as a binary outcome (yes/no) i.e. as a proportion.

I.5.1 Knowledge of HIV status

Question: How much did PopART increase recent knowledge of HIV-Status (within the past year) through HIV testing and retesting, in each round ("year") of the study.

PopART is expected to achieve high levels of knowledge of HIV status.

<u>Cohort</u>: All who are contacted by CHiP teams and consent to participate in the intervention, separately for each round ("year") of the study.

<u>Endpoint</u>: Knowledge of HIV status, self-reported or from accepting the offer of rapid HIV testing from CHiP teams.

Details: All individuals who are contacted by CHiP teams and consent to participate in the intervention are included. Knowledge of HIV status has five mutually exclusive categories. The binary endpoint for assessing the PopART effect is Known vs Unknown

- Known; Self-reported HIV-infected
- Known; Accepted offer of rapid HIV testing from CHiP teams
- Known; Self-reported HIV uninfected, reports recently tested for HIV elsewhere ["recently" tested will be defined in at least 2 ways: testing in past 3 months, and testing in past year (12 months)]
- Unknown (previously tested but did not disclose result of most recent HIV test; has never been tested)
- Unknown; Self-reported HIV uninfected, no testing in past year

Analysis: Number and proportion who know their HIV status will be reported in each community, for each country, for each arm, and overall, with 95% confidence limits based on the binomial distribution when summarizing proportions separately for each community. For the other analyses, which report average values across communities, the clustering in the data will be accounted for by calculating standard errors and corresponding confidence intervals based on the between-community variation in the proportion with the outcome (beyond that explained by country and trial arm).

Subgroups

- Pregnant women

I.5.2 HIV diagnosis and initiation of care

Question: For individuals who are identified as HIV-infected by the CHIP teams, either through self-report of HIV-infected status or from accepting a rapid HIV test from the CHIP teams and the test result was HIV-positive, what proportion are linked to care within 6 months in each round ("year") of the study?

PopART is expected to result in faster linkage to HIV care after HIV diagnosis and/or referral to HIV care.

<u>Cohort</u>: All individuals who were contacted, consented to participate, were identified as HIV-infected and not on ART, and were referred to HIV care by the CHiP teams, separately for each round ("year") of the study.

<u>Endpoint</u>: Registered for HIV Care within 6 months of referral to HIV care.

Details: Registering for HIV care is only assessed in those who are identified by the CHiP teams as HIV-positive and can be self-reported and/or verified by documents presented by the individual.

Analysis: Number and proportion who have registered for HIV care within 6 months of referral to HIV care, among all individuals who were identified as HIV-infected and were referred to HIV care and were not taking ART at the time of referral, derived from a "time-to-event" analysis. Proportions will be reported for each community, for each country, for each arm, and overall, with 95% confidence limits based on the "time-to-event" (Kaplan-Meier) analysis when summarizing proportions separately for each community. For the other analyses, which report average values across communities, the clustering in the data will be accounted for by calculating standard errors and corresponding confidence intervals based on the between-community variation in the proportion with the outcome (beyond that explained by country and trial arm).

Subgroups

- Pregnant women
- Newly diagnosed HIV-positive; self-reported HIV-positive and never previously registered for HIV care

I.5.3 ART uptake among HIV-infected individuals

Question: What are the proportions of HIV-infected individuals who are on ART in each arm, among individuals who have been identified as HIV-infected by the CHiP teams, in each round ("year") of the study?

Question: How much did PopART increase ART uptake?

PopART is expected to increase the proportion reporting ART amongst HIV infected individuals, especially in Arm A.

<u>Cohort</u>: All individuals who are contacted by CHiP teams, consent to participate in the intervention, and are identified by CHiP teams as HIV-infected either because they self-report they are HIV-infected or because they accept the offer of rapid HIV testing from the CHIP teams and the test result is HIV-positive, separately for each round ("year") of the study.

<u>Endpoint 1</u>: Self-reported ART at time of the annual household visit.

Endpoint 2: Self-reported ART at the end of the round ("year"), using the most recently available information from follow-up visits made to individuals who have been identified as HIV-infected, with the denominator restricted to individuals who were still resident in the same area of the community at the end of the round according to the last information collected through CHiP follow-up visits during the round.

Details: ART is only assessed in individuals who are identified by the CHiP teams as HIV-positive and can be self-reported and/or verified by ART documents presented by the individual.

Analysis: Number and proportion who are taking ART, among all individuals identified as HIV-infected, will be reported in each community, for each arm, and for each arm within each country, with 95% confidence limits based on the binomial distribution when summarizing proportions separately for each community. For the other analyses, which report average values across communities, the clustering in the data will be accounted for by calculating standard errors and corresponding confidence intervals based on the between-community variation in the proportion with the outcome (beyond that explained by country and trial arm).

Subgroups

- Pregnant women

I.5.4 Retention in HIV care among HIV-infected individuals who are registered for HIV care

Question: Does the PopART intervention achieve high levels of retention in HIV care, in each round ("year") of the study?

PopART is expected to achieve high levels of retention in HIV care among HIV-infected individuals who have ever registered for HIV care.

Cohort: All individuals who are contacted by CHiP teams, consent to participate in the intervention, and are identified by CHiP teams as HIV-infected either because they self-report they are HIV-infected or because they accept the offer of rapid HIV testing from the CHIP teams and the test result is HIV-positive, restricted to those who as part of the annual household visit report they have previously registered for HIV care, separately for each round ("year") of the study.

Endpoint: HIV care visit within the 3 months prior to the annual household visit.

Analysis: Number and proportion who are retained in HIV care, among all individuals identified as HIV-infected who have ever registered for HIV care, will be reported in each community, for each country, for each arm, and overall, with 95% confidence limits based on the binomial distribution when summarizing proportions separately for each community. For the other analyses, which report average values across communities, the clustering in the data will be accounted for by calculating standard errors and corresponding confidence intervals based on the between-community variation in the proportion with the outcome (beyond that explained by country and trial arm).

Subgroups

- First registered for HIV care before or after PopART study started (pre- or post-2014)

I.5.5 Retention on ART among HIV-infected individuals who have ever taken ART

Question: Does the PopART intervention achieve high levels of retention on ART, in each round ("year") of the study?

PopART is expected to achieve high levels of retention on ART among HIV-infected individuals who have ever taken ART.

Cohort: All individuals who are contacted by CHiP teams, consent to participate in the intervention, and are identified by CHiP teams as HIV-infected either because they self-report they are HIV-infected or because they accept the offer of rapid HIV testing from the CHIP teams and the test result is HIV-positive, restricted to those who have reported at least once that they have "ever" taken ART, separately for each round ("year") of the study. For example, for round 2 ("year 2") of the study, HIV-infected individuals are included in the analysis if they have reported, at one or more of the Round 1 and Round 2 annual household visits or a follow-up visit made at any time up to the Round 2 annual household visit, that they have "ever" taken ART.

Endpoint: Taken ART within the 1 month prior to the annual household visit and missed no pills in the previous 3 days.

Analysis: Number and proportion who are retained on ART, among all individuals identified as HIV-infected who have ever taken ART at the time of the annual household visit, will be reported in each community, for each arm, and for each arm within each country, with 95% confidence limits based on the binomial distribution when summarizing proportions separately for each community. For the other analyses, which report average values across communities, the clustering in the data will be accounted for by calculating standard errors and corresponding confidence intervals based on the between-community variation in the proportion with the outcome (beyond that explained by country and trial arm).

Subgroups

- First started ART before or after PopART study started (pre- or post-2014)

I.5.6 Uptake of MMC among HIV-uninfected men

Question: What proportion of uncircumcised [self-reported] HIV-uninfected men were circumcised during the PopART intervention, in each round ("year") of the study?

Cohort 1: Men who are contacted by CHiP teams and consent to participate in the intervention, report they are not circumcised or do not know their circumcision status, and accept the offer of HIV testing from CHiP teams and the test result is HIV-negative, and are still resident in the community one year later, separately for each round ("year") of the study.

Endpoint 1: MMC within the previous year (self-reported), among men who in the previous round ("year") reported they were not circumcised and tested HIV-negative. This endpoint can be measured from Round 2 onwards.

Cohort 2: Men who are contacted by CHiP teams and consent to participate in the intervention, and accept the offer of HIV testing from CHiP teams and the test result is HIV-negative, separately for each round ("year") of the study

Endpoint 2: MMC, at any time prior to the annual household visit

Cohort 3: Men who are contacted by CHiP teams and consent to participate in the intervention and accept the offer of HIV testing from CHiP teams and the test result is HIV-negative, and report they are not circumcised or do not know their circumcision status, separately for Rounds ("years") 2 and 3 of the study.

Endpoint 3: MMC (self-reported), among men who accepted a referral for MMC [in Rounds 2 and 3, the protocol was for CHiPs to conduct one follow-up visit to men they had referred for MMC, >1 month after the referral date].

PopART is expected to increase the proportion of HIV uninfected men who are circumcised with MMC. Endpoint 2 measures the uptake of MMC among all HIV-uninfected men, regardless of when MMC was done, and so provides a cumulative measure of MMC uptake.

Analysis: Number and proportion of HIV-uninfected men who are circumcised with MMC will be reported in each community, for each country, for each arm, and overall, with 95% confidence limits based on the binomial distribution when summarizing proportions separately for each community. For the other analyses, which report average values across communities, the clustering in the data will be accounted for by calculating standard errors and corresponding confidence intervals based on the between-community variation in the proportion with the outcome (beyond that explained by country and trial arm).

I.5.7 Estimates of coverage against the first two of the UNAIDS 90-90-90 targets

Question: How much did PopART increase coverage against the first 90 target (90% of HIV-positive individuals know their HIV-positive status) and the second 90 target (among HIV-positive individuals who know their HIV-positive status, 90% are on ART), in each round ("year") of the study.

PopART is expected to achieve high levels of coverage against the 90-90 targets.

I.5.7.1 Estimates of coverage against the first 90 target

<u>Cohort 1</u>: All who are contacted by CHiP teams and consent to participate in the intervention, and are HIV-positive, separately for each round ("year") of the study. The number of HIV-positive individuals among all who participated in the round is calculated as the sum of: (1) the number who were known by the CHiPs to be HIV-positive, because they self-reported they were HIV-positive to the CHiPs or they were newly diagnosed HIV-positive by the CHiPs based on a rapid HIV test; and (2) an *estimated* number among those whose HIV status was not known to CHiPs. For (2), our "central" estimate is that HIV prevalence among individuals whose HIV status is not known to the CHiPs is the same as among those who accepted the offer of HIV testing, stratified on all combinations of community, gender, age group, and participation and residency in the previous round. For (2), sensitivity analysis is done assuming that HIV prevalence among individuals whose HIV status is not known to the CHiPs is 2-3 times higher than among those who accepted the offer of HIV testing.

<u>Endpoint 1a</u>: "Central estimate"

(i) <u>At start of the round</u>: Knowledge of HIV-positive status is defined as self-reported HIV-positive (this implicitly assumes that among the HIV-positive individuals whose HIV-positive status is not known to the CHiPs, none know their HIV-positive status).

(ii) <u>By the end of the round</u>: Knowledge of HIV-positive status is defined as self-reported HIV-positive or newly diagnosed HIV-positive by the CHiPs (this implicitly assumes that among the HIV-positive individuals whose HIV-positive status is not known to the CHiPs, none know their HIV-positive status).

<u>Endpoint 1b</u>: "Sensitivity analysis estimate"

(i) <u>At start of the round</u>: Knowledge of HIV-positive status is defined as self-reported HIV-positive, plus a *proportion* of those who are HIV-positive, but their HIV-positive status is not known to the CHiPs. Among those who are HIV-positive but their HIV-positive status is not known to CHiPs, three alternative assumptions are made for the percentage who know their HIV-positive status (i) the percentage who know their HIV-positive status is the *same* as the "at start of round" value for adults whose HIV status is

known to CHiPs i.e. equal to (self-reported HIV-positive to CHiPs / total known by the CHiPs to be HIV-positive by the end of the round) (ii) the percentage who know their HIV-positive status is *half* of the "at start of round" value among adults whose HIV-positive status is known to CHiPs by the end of the round (iii) the percentage who know their HIV-positive status is 0%.

(ii) By the end of the round: - Knowledge of HIV-positive status is defined as self-reported HIV-positive or newly diagnosed HIV-positive by the CHiPs, plus a *proportion* of those who are HIV-positive, but their HIV-positive status is not known to the CHiPs. Among those who are HIV-positive but their HIV-positive status is not known to CHiPs, three alternative assumptions are made for the percentage who know their HIV-positive status (i) the percentage who know their HIV-positive status is the *same* as the "at start of round" value for adults whose HIV status is known to CHiPs i.e. equal to (self-reported HIV-positive to CHiPs / total known by the CHiPs to be HIV-positive by the end of the round) (ii) the percentage who know their HIV-positive status is *half* of the "at start of round" value among adults whose HIV-positive status is known to CHiPs by the end of the round (iii) the percentage who know their HIV-positive status is 0%.

Details: All individuals who are contacted by CHiP teams and consent to participate in the intervention and are HIV-positive are included.

Cohort 2: All who are a household member in the community during the round - i.e. both individuals who are contacted and consent to participate in the intervention (equal to Cohort 1) and also those who are not contacted by CHiPs and those who are contacted but do not consent to participate in the intervention - and are HIV-positive, separately for each round ("year") of the study. The number of HIV-positive individuals among all who participated in the round is calculated as for Cohort 1, for both "central" and "sensitivity analysis" estimates. The number of HIV-positive individuals among all who did not participate in the round is calculated with the assumption that HIV prevalence is the same among non-participants as among participants, stratified on all combinations of community, gender, age group, and participation and residency in the previous round; in sensitivity analysis HIV prevalence among non-participants is varied in the range 80%-125% of the "central" estimate.

Endpoint 2a: "Central estimate".

(i) At start of the round: For individuals who participated in the round, knowledge of HIV-positive status is defined as for Endpoint 1a, i.e. as self-reported HIV-positive to CHiPs. Among all who did not participate in the round, the number of HIV-positive individuals who know their HIV-positive status is calculated with the assumption that the proportion who know their HIV-positive status is the same as among participants *at the start of the round*, stratified on all combinations of community, gender, age group, and participation and residency in the previous round.

(ii) By the end of the round: For individuals who participated in the round, knowledge of HIV-positive status is defined as for Endpoint 1a, i.e. as self-reported HIV-positive to CHiPs or newly diagnosed HIV-positive by CHiPs. Among all who did not participate in the round, the number of HIV-positive individuals who know their HIV-positive status is calculated with the assumption that the proportion who know their HIV-positive status by the end of the round is the same as among participants *at the start of the round*, stratified on all combinations of community, gender, age group, and participation and residency in the previous round – i.e. the assumption is that their knowledge of HIV-positive status does not change during the round, equivalent to assuming (conservatively) that non-participants do not receive HIV testing services during the round.

Endpoint 2b: "Sensitivity analysis estimate".

(i) At start of the round: For individuals who participated in the round, knowledge of HIV-positive status is defined as for Endpoint 1b. Among all who did not participate in the round, the proportion who know their HIV-positive status is varied in the range 80%-125% of the estimated proportion among participants *at the start of the round*.

(ii) By the end of the round: For individuals who participated in the round, knowledge of HIV-positive status is defined as for Endpoint 1b. Among all who did not participate in the round, the proportion who know their HIV-positive status is varied in the range 80%-125% of the estimated proportion among participants *at the start of the round*.

Details: All individuals who are a household member during the round, and are HIV-positive, are included.

Analysis: Number and proportion who know their HIV-positive status will be reported in each community, for each country, for each arm, and overall, with uncertainty intervals based on the sensitivity analyses. For the other analyses, which report average values across communities, the clustering in the data will be accounted for by calculating standard errors and corresponding confidence intervals based on the between-community variation in the proportion with the outcome (beyond that explained by country and trial arm); this will be done both for "central" estimates and for "sensitivity analysis" estimates.

I.5.8 Estimates of coverage against the second 90 target

Cohort 1A: All who are contacted by CHiP teams and consent to participate in the intervention and know their HIV-positive status following the annual round visit, separately for each round ("year") of the study. The "central estimate" of the total who know their HIV-positive status, is the total whose HIV-positive status is known to the CHiPs following the annual round visit – as described in I.5.7.1 for Endpoint 1a. In sensitivity analysis, the total who know their HIV-positive status is varied as described in I.5.7.1 for Endpoint 1b.

Endpoint 1Aa: "Central estimate"

(i) At start of the round: On ART (based on self-report) on the date of the annual round visit.

Endpoint 1Ab: "Sensitivity analysis" estimate

(i) At start of the round: On ART (based on self-report) on the date of the annual round visit, among those who are known by the CHiPs to be HIV-positive. Among the estimated number of individuals who know their HIV-positive status, but their HIV-positive status is not known to CHiPs, ART uptake is varied in the range 50%-100% of the value at the start of the round among those who *self-reported they were HIV-positive* to the CHiPs.

Cohort 1B: All who are contacted by CHiP teams and consent to participate in the intervention, and know their HIV-positive status following the annual round visit, *and are still resident in the same area of the community at the end of the round according to the last information collected from CHiP follow-up visits during the round*, separately for each round ("year") of the study. The "central estimate" and "sensitivity analysis" estimates are calculated in the same way as for Endpoint 1Aa and Endpoint 1Ab respectively, with the additional assumption that the percentage who remain resident in the same area of the community at the end of the round among those who know their HIV-positive status but did not self-report HIV-positive to CHiPs is the *same as among those whose HIV-positive status is known to CHiPs*.

Endpoint 1Ba: "Central estimate"

(ii) <u>By the end of the round</u>: On ART (based on self-report) by the end of the round, according to the last follow-up information collected by CHiPs during the round.

<u>Endpoint 1Bb</u>: "Sensitivity analysis" estimate

(ii) <u>By the end of the round</u>: On ART (based on self-report) by the end of the round, according to the last follow-up information collected by CHiPs during the round, among those who are known by the CHiPs to be HIV-positive. Among the estimated number of individuals who know their HIV-positive status but their HIV-positive status is not known to CHiPs, ART uptake is varied in the range 50%-100% of the value *at the start of the round* among those who self-reported they were HIV-positive to the CHiPs – i.e. it is assumed that ART uptake does not increase during the round among individuals who know their HIV-positive status but their HIV-positive status is not known to CHiPs.

Details: All individuals who are contacted by CHiP teams and consent to participate in the intervention and are known by the CHiPs to be HIV-positive, plus an estimated number who participated in the round and know their HIV-positive status but their HIV-positive status is not known to the CHiPs following the annual round visit, are included.

<u>Cohort 2A</u>: All who were a household member in the community during the round, were HIV-positive, and knew their HIV-positive status. Thus, this cohort includes both (a) individuals who were contacted and consented to participate in the intervention (equal to Cohort 1A) and also (b) those who were not contacted by CHiPs, and those who were contacted but did not consent to participate in the intervention, separately for each round ("year") of the study. For (b) the number who know their HIV-positive status is estimated as described in section I.5.7.1 for Endpoints 2a and 2b.

<u>Endpoint 2Aa</u>: "Central estimate"

(i) <u>At start of the round</u>: On ART (based on self-report) on the date of the annual round visit, among individuals who participated and are known by the CHiPs to be HIV-positive. Among individuals who did not participate in the round but know their HIV-positive status, the percentage on ART is assumed to be the same as *at the start of the round* among individuals who self-reported they were HIV-positive to the CHiPs.

<u>Endpoint 2Ab</u>: "Sensitivity analysis" estimate

(i) <u>At start of the round</u>: On ART (based on self-report) on the date of the annual round visit, among those who are known by the CHiPs to be HIV-positive. Among the estimated number of individuals who participated in the intervention and know their HIV-positive status, but their HIV-positive status is not known to CHiPs, the percentage on ART is varied in the range 50%-100% of the value *at the start of the round* among those who self-reported they were HIV-positive to the CHiPs. Among the estimated number who know their HIV-positive status but did not participate in the round, the percentage on ART is varied in the range 80%-125% of the estimated percentage *among participants at the start of the round [participants who either (a) self-reported they were HIV-positive to the CHiPs or (b) were estimated to know their HIV-positive status at the start of the round but did not disclose this to CHiPs]*.

<u>Cohort 2B</u>: All who were a household member in the community during the round, were HIV-positive, and knew their HIV-positive status, *and were still resident in the same area of the community at the end of the round,* separately for each round ("year") of the study. Thus this cohort is a subset of cohort 2A; and it is assumed that, among those who know their HIV-positive status but their HIV-positive status is not known to CHiPs, the percentage who remain resident in the same area of the community at the end of the round is the same as among those whose HIV-positive status *is* known to the CHiPs.

<u>Endpoint 2Ba</u>: "Central estimate"

(ii) By the end of the round: On ART (based on self-report) by the end of the round, according to the last follow-up information collected by CHiPs during the round, among those whose HIV-positive status is known to the CHiPs. Among individuals who did not participate but know their HIV-positive status, the percentage on ART is assumed to be the same as the value *at the start of the round* among those who self-reported they were HIV-positive to the CHiPs.

Endpoint 2Bb: "Sensitivity analysis" estimate

(ii) By the end of the round: On ART (based on self-report) by the end of the round, according to the last follow-up information collected by CHiPs during the round, among those who are known by the CHiPs to be HIV-positive. Among the estimated number of individuals who participated and know their HIV-positive status but their HIV-positive status is not known to CHiPs, the percentage on ART is varied in the range 50%-100% of the value *at the start of the round* among those who self-reported they were HIV-positive to the CHiPs – i.e. it is assumed that ART uptake does not increase during the round among individuals who participated and know their HIV-positive status but their HIV-positive status is not known to CHiPs. Among the estimated number who know their HIV-positive status but did not participate in the round, the percentage on ART is varied in the range 80%-125% of the estimated percentage *among participants at the start of the round [participants who either (a) self-reported they were HIV-positive to the CHiPs or (b) were estimated to know their HIV-positive status at the start of the round but did not disclose this to CHiPs]*.

Details: All individuals who are a household member during the round, and are HIV-positive, and know their HIV-positive status, are included.

Analysis: Number and proportion who are on ART will be reported in each community, for each country, for each arm, and overall, with uncertainty intervals based on the sensitivity analyses. For the other analyses, which report average values across communities, the clustering in the data will be accounted for by calculating standard errors and corresponding confidence intervals based on the between-community variation in the proportion with the outcome (beyond that explained by country and trial arm); this will be done both for "central" estimates and for "sensitivity analysis" estimates.
