# Supplementary material for: The effect of universal testing and treatment on HIV stigma in 21 communities in Zambia and South Africa
Source: AIDS. 2020 Aug 6;34(14):2125–35. doi: 10.1097/QAD.0000000000002658 (PMC8425632; doi:10.1097/QAD.0000000000002658)
Supplement: Supplemental Digital Content [file aids-34-2125-s002.doc]

**The Role of HIV-related Stigma in the Delivery of Universal Test and Treatment for HIV prevention**

**A Study of the HIV Prevention Trials Network**

**Sponsored by:**

Division of AIDS, U.S. National Institute of Allergy and Infectious Diseases

U.S. National Institute of Mental Health

U.S. National Institutes of Health

**Protocol Chair:**

James Hargreaves

**Version 2.0**

**06 June 2015**

Contents

[LIST OF ABBREVIATIONS AND ACRONYMS 4](#__RefHeading___Toc376807904)

[PROTOCOL TEAM ROSTER 5](#__RefHeading___Toc376807905)

[Investigator Signature Page 8](#__RefHeading___Toc376807906)

[*1.0* Introduction 9](#__RefHeading___Toc376807907)

[*1.1* Background 9](#__RefHeading___Toc376807908)

[*1.2* Rationale for study 10](#__RefHeading___Toc376807909)

[Box 1: The PopART intervention 10](../../../../C:/amoore/Desktop/Temp%20071/Stigma/HPTN%20071a%20Stigma%20Protocol%20Ver%201.0_06%20Jan%202014.doc#__RefHeading___Toc376807910)

[*2.0* STUDY OBJECTIVES AND DESIGN 11](#__RefHeading___Toc376807911)

[*2.1* Objectives 11](#__RefHeading___Toc376807912)

[*2.2* Study Design 12](#__RefHeading___Toc376807913)

[*2.3* Timing of Deployment of Research Components 12](#__RefHeading___Toc376807914)

[*3.0* STUDY INTERVENTIONS 13](#__RefHeading___Toc376807915)

[*4.0* STUDY SETTING 13](#__RefHeading___Toc376807916)

[*4.1* CHiPs 13](#__RefHeading___Toc376807917)

[*4.1.1* Health facility staff 14](#__RefHeading___Toc376807918)

[*5.0* Research PROCEDURES and activities 14](#__RefHeading___Toc376807919)

[*5.1* Cohort study of Health facility staff and CHiPs 14](#__RefHeading___Toc376807920)

[*5.1.1* Inclusion Criteria 14](#__RefHeading___Toc376807921)

[*5.1.2* Exclusion Criteria 14](#__RefHeading___Toc376807922)

[*5.1.3* Selection of Study Participants 15](#__RefHeading___Toc376807923)

[*5.1.4* Negotiating access to potential participants 15](#__RefHeading___Toc376807924)

[*5.1.5* Data Collection 16](#__RefHeading___Toc376807925)

[*5.1.6* Data management 17](#__RefHeading___Toc376807926)

[*5.1.7* Retention 18](#__RefHeading___Toc376807927)

[*5.2* Qualitative research 19](#__RefHeading___Toc376807928)

[*5.2.1* Aim 19](#__RefHeading___Toc376807929)

[*5.2.2* In-situ key informant interviews and observation 19](#__RefHeading___Toc376807930)

[*5.2.3* Complementary research approaches to document context 20](#__RefHeading___Toc376807931)

[*5.2.4* Data management for qualitative data 21](#__RefHeading___Toc376807932)

[*6.0* Statistical Considerations and Data Analysis 22](#__RefHeading___Toc376807933)

[*6.1* Sample size for cohort study 22](#__RefHeading___Toc376807934)

[*6.1.1* Justification of sample size 22](#__RefHeading___Toc376807935)

[*6.2* Data Analysis 23](#__RefHeading___Toc376807936)

[*6.2.1* Objective 1 23](#__RefHeading___Toc376807937)

[*6.2.2* Objective 2: does the PopART intervention reduce HIV-related stigma among health-facility staff? 23](#__RefHeading___Toc376807938)

[*6.2.3* Objective 3: are higher levels of HIV-related stigma among CHiPs/HFS associated with less successful delivery of the PopART intervention? 24](#__RefHeading___Toc376807939)

[*6.3* Objective 4: Analysis of Qualitative data 25](#__RefHeading___Toc376807940)

[*7.0* SAFETY MONITORING AND SOCIAL HARM/Adverse EVENT REPORTING 26](#__RefHeading___Toc376807941)

[*7.1* Safety Reporting 26](#__RefHeading___Toc376807942)

[*8.0* HUMAN SUBJECTS CONSIDERATIONS 26](#__RefHeading___Toc376807943)

[*8.1* Benefits 27](#__RefHeading___Toc376807944)

[*8.2* Risks 28](#__RefHeading___Toc376807945)

[*8.3* Informed Consent 29](#__RefHeading___Toc376807946)

[*8.3.1* Confidentiality 30](#__RefHeading___Toc376807947)

[*8.3.2* Referral to independent counseling services 31](#__RefHeading___Toc376807948)

[*8.3.3* Data not to be used for M&E purposes 31](#__RefHeading___Toc376807949)

[*8.3.4* Monitor experiences of participation 31](#__RefHeading___Toc376807950)

[*8.3.5* Independent Ethical Review 31](#__RefHeading___Toc376807951)

[*8.3.6* Study Discontinuation 31](#__RefHeading___Toc376807952)

[*9.0* ADMINISTRATIVE PROCEDURES 32](#__RefHeading___Toc376807953)

[*9.1* Protocol Registration 32](#__RefHeading___Toc376807954)

[*9.2* Study Activation 32](#__RefHeading___Toc376807955)

[*9.3* Study Coordination 32](#__RefHeading___Toc376807956)

[*9.4* Study Monitoring 33](#__RefHeading___Toc376807957)

[*9.5* Protocol Compliance 33](#__RefHeading___Toc376807958)

[*9.6* Investigator's Records 34](#__RefHeading___Toc376807959)

[*9.7* Use of Information and Publications 34](#__RefHeading___Toc376807960)

[*10.0* References 35](#__RefHeading___Toc376807961)

[*11.0* Appendices 39](#__RefHeading___Toc376807962)

[Appendix I: Sample Informed Consent Form – CHIPS/HFS cohort 40](#__RefHeading___Toc376807963)

[Appendix II: Sample Informed Consent Form – Qualitative study participants 47](#__RefHeading___Toc376807964)

# **LIST OF ABBREVIATIONS AND ACRONYMS**

AE Adverse Event

AIDS Acquired Immunodeficiency Syndrome

ART Anti-Retroviral Therapy

ARV Anti-Retroviral

CHiPs Community HIV-Care Providers

CORE (HPTN) Coordinating and Operations Center

CTU Clinical Trials Unit

DAIDS Division of AIDS

DAIDS PRO DAIDS Protocol Registration Office

DSMB Data Safety Monitoring Board

DTTC Desmond Tutu Tuberculosis Centre

EC Ethics Committee

ENA Enrolled Nurse Assistant

FSW Female Sex Worker

HCT HIV Counselling and Testing

HFS Health Facility Staff

HIV Human Immunodeficiency Virus

HPTN HIV Prevention Trials Network

ICF Informed Consent Form

IRB Institutional Review Board

LSHTM London School of Hygiene and Tropical Medicine

MMC Medical Male Circumcision

MSM Men who have Sex with Men

NGO Non-governmental Organization

NIAID (United States) National Institute of Allergy and Infectious

NL (HPTN) Network Laboratory

PDA Persoal Digital Assistant

PLWH Persons Living with HIV

PMTCT Prevention of Mother to Child Transmission

PopART Population Effects of Antiretroviral Therapy to Reduce HIV Transmission

RE Regulatory Entity

RSC Regulatory Support Center

SDMC (HPTN) Statistical and Data Management Center

SMC Study Monitoring Committee

STI Sexually Transmitted Infection

TB Tuberculosis

UK United Kingdom

US United States

UTT Universal Testing and Treatment

WHO World Health Organization

ZAMBART Zambia AIDS Related Tuberculosis Project

# **PROTOCOL TEAM ROSTER**

**Ab Schaap**

Senior Data Manager

ZAMBART Project

London School of Hygiene & Tropical Medicine

Ridgeway Campus

P.O. Box 50697

10101 Lusaka, Zambia

**Phone:** +260 211 254710, Mobile: +260 977 442965

**Fax:** **:** +260 211 254710

**Email:** *ab@zambart.org.zm*

**Anne Stangl**

Senior Behavioral Scientist

International Center for Research on Women

1120 20th St. NW, Suite 500N

Washington, DC 20036

**Phone**: +1 202 742 1242

**Email**: [*astangl@icrw.org*](mailto:astangl@icrw.org)

**Ayana Moore**

Scientist

FHI360

359 Blackwell St. Suite 200

Durham NC 27701, USA

**Phone:** +1 919 544 7040 ext. 11244

**Email:** [*amoore@fhi360.org*](mailto:amoore@fhi360.org)

**Cyndi Grossman**

Chief, Secondary Prevention Program

National Institute of Mental Health

6001 Executive Boulevard

Bethesda, MD 20892-9663
**Phone**: +1 301 443 8962

**Email**: [*grossmanc@mail.nih.gov*](mailto:grossmanc@mail.nih.gov)

**David Burns**

Prevention Sciences Branch Chief

DAIDS Medical Officer

NIAID/NIH

6700B Rockledge Drive

Bethesda MD 20892, USA

**Phone:** +001-301-435-8896

**Email:** [*burnsda@niaid.nih.gov*](mailto:burnsda@niaid.nih.gov)

**Graeme Hoddinott**

Social Science Lead

Desmond Tutu TB Centre

Department of Paediatrics and ChildHealth

University of Stellenbosch

P.O. Box 19063

Tygerberg, South Africa, 7505

**Phone:** [*+27 21 938 9846*](tel:%2B27-21-938-9846)

**Email:** [*graemeh@sun.ac.za*](mailto:graemeh@sun.ac.za)

**Helen Ayles**

Clinical Scientist/Site Principal Investigator

ZAMBART

University of Zambia

School of Medicine, Ridgeway Campus

Lusaka, Zambia

Dept of Clinical Research

London School of Hygiene and Tropical Medicine

Keppel Street

London WC1E 7HT, UK

**Phone:** +260 211 254710, 260 211257215

**Fax:** +260 211 254710

**Email:** [*helen@zambart.org.zm*](mailto:helen@zambart.org.zm)

**James Hargreaves**

*Senior Lecturer in Epidemiology*

Department of Infectious Disease Epidemiology

London School of Hygiene & Tropical Medicine

Keppel Street

London WC1E 7HT, UK

**Phone**: +44 (0)20 7927 2955

**Fax**: +44 (0)20 7637 4314

**Email:** [*james.hargreaves@lshtm.ac.uk*](mailto:james.hargreaves@lshtm.ac.uk)

**Kirsty M. Sievwright**

*Program Associate*

International Center for Research on Women (ICRW)

1120 20th Street, NW

Suite 500 North

Washington, DC 20036

**Phone:**  +1 202 742  1248

**Email:** [ksievwright@icrw.org](mailto:ksievwright@icrw.org)

**Kwame Shanaube**

PopART Study Manager - Zambia

ZAMBART Project

PO Box 50697

Ridgeway Campus

Lusaka, Zambia

ZAMBIA

**Phone :** +260-211-254710/+260-211-257215

**Fax :** +260-211-254710

**Email :** [*kshanaube@zambart.org.zm*](mailto:kshanaube@zambart.org.zm)

**Lario Viljoen**

*Social Scientist*

Desmond Tutu TB Centre

Tygerberg Campus

Stellenbosch University

Cape Town, South Africa, 7505

**Phone**: [(+27)21 938 9846](tel:(%2B27)21 938 9846)

**Email** : [lario@sun.ac.za](mailto:lario@sun.ac.za)

**Nulda Beyers**

Desmond Tutu TB Centre

Department of Paediatrics and ChildHealth

University of Stellenbosch

P.O. Box 19063

Tygerberg, South Africa, 7505

**Phone:** [*+27 21 938 9846*](tel:%2B27-21-938-9846)

**Email:nb@sun.ac.za**

**Pamela Lilleston, PhD, MHS**

*Monitoring and Evaluation Specialist*

International Center for Research on Women (ICRW)

1120 20th Street, NW

Suite 500 North

Washington, DC 20036

**Phone:** +1 202 742 1249

**E-mail:** [*plilleston@icrw.org*](mailto:plilleston@icrw.org)

**Richard Hayes**

Protocol Chair, Lead Statistician

Department of Infectious Disease Epidemiology

London School of Hygiene & Tropical Medicine

Keppel Street

London WC1E 7HT, UK

**Phone**: +44 (0)20 7927 2243

**Fax**: +44 (0)20 7637 4314

**Email:** [*richard.hayes@lshtm.ac.uk*](mailto:richard.hayes@lshtm.ac.uk)

**Rory Dunbar**

Desmond Tutu TB Centre

Department of Paediatrics and ChildHealth

University of Stellenbosch

P.O. Box 19063

Tygerberg, South Africa, 7505

**Phone:** [*+27 21 938 9846*](tel:%2B27-21-938-9846)

**Email:rory@sun.ac.za**

**Shari Krishnaratne**

Research Fellow

Department Public Health Policy

London School of Hygiene & Tropical Medicine

Keppel Street

London WC1E 7HT, UK

**Phone**: +44 (0)20 7927 2656

**Fax**: +44 (0)20 7637 4314

**Email:** shari.krishnaratne@lshtm.ac.uk

**Tanette Headen**

Research Assistant II

FHI360

359 Blackwell St. Suite 200

Durham, NC 27701

**Phone:** +1 919 544 7040 ext. 11297

**Email:** [*theaden@fhi360.org*](mailto:theaden@fhi360.org)

**Virginia Bond**

Social Scientist

London School of Hygiene & Tropical Medicine

ZAMBART Project, School of Medicine

P.O. Box 50697

Lusaka, Zambia

**Phone**: *+*260 211 254710

**Email:** [*gbond@zambart.org.zm*](mailto:gbond@zambart.org.zm)

**HPTN 071a**

**______________**

**Version 1.0**

**06 January 2014**

**______________**

# Investigator Signature Page

**A Study of the HIV Prevention Trials Network (HPTN)**

**Sponsored by:**

Division of AIDS, U.S. National Institute of Allergy and Infectious Diseases

U.S. National Institute of Mental Health

U.S. National Insitutes of Health

I, the Investigator of Record, agree to conduct this study in full accordance with the provisions of this protocol. I agree to maintain all study documentation for a minimum of three years after submission of the site’s final Financial Status Report to the Division of AIDS (DAIDS), unless otherwise specified by DAIDS or the HIV Prevention Trials Network (HPTN) Coordinating and Operations Center. Publication of the results of this study will be governed by HPTN policies. Any presentation, abstract, or manuscript will be made available by the investigators to the HPTN Manuscript Review Committee and DAIDS for review prior to submission.

I have read and understand the information in this protocol and will ensure that all associates, colleagues, and employees assisting in the conduct of the study are informed about the obligations incurred by their contribution to the study.

__________________________________

Name of Investigator of Record

__________________________________ _________________________________

Signature of Investigator of Record Date

**PREFACE**

This document describes part of an ancillary study nested within the HPTN071 trial, funded by NIMH. It includes a cohort study of health workers involved in the delivery of PopART and data collection with potential clients of HIV-related services. Data collection focuses on understanding HIV-related stigma in the health system, including overlapping stigma in relation to groups such as female sex workers, MSM and others. It forms one part of a set of work known as **“the stigma ancillary”** which also includes guidance to collection and analysis of data on HIV-related stigma within the HPTN071 PC and Case-Control Studies. The work is linked to the broader social science and social epidemiology agenda within HPTN071 which includes research on a range of structural drivers and social inequalities and HIV infection within the context of the PopART trial

# Introduction

## Background

Zambia and South Africa are among the most severely human immunodeficiency virus (HIV)-affected countries in sub-Saharan Africa with an estimated 980,000 people in Zambia and an estimated 5,600,000 in South Africa living with HIV . Research trials have identified partially efficacious HIV prevention approaches, including those that use HIV antiretroviral treatments among those infected to prevent onward transmission. The challenge now is to develop health system approaches that ensure that these interventions can be effective in practice. The ‘Population Effects of Antiretroviral Therapy to Reduce HIV Transmission’ (PopART/HPTN 071) trial will evaluate the effectiveness of a combination HIV prevention approach that incorporates a “universal test and treat” (UTT) strategy on community-level HIV incidence in a cluster randomised trial in Zambia and South Africa.

It is an ambitious trial. HIV-related stigma in communities and health–settings may pose challenges to the acceptability of regular HIV testing, early anti-retroviral therapy (ART) initiation and lifelong treatment adherence in entire populations, particularly for populations who are hard-to-reach . Weak health systems may also struggle to deliver the strategy without significant additional resources. The extent of behavioral risk disinhibition , levels of drug toxicity, identifying durable second and third line treatment regimens and the proportion of transmission events that occur during acute HIV infection will also be critical determinants of the success of a UTT intervention. However, if the approach is successful it may reduce HIV-related stigma and relieve the burden on health workers by removing the need for targeted interventions, simplifying clinical decisions around HIV care and demonstrating HIV to be preventable and treatable.

This protocol describes a cohort study, and linked qualitative and participatory research, nested within the 071 trial. It will investigate HIV-related stigma and other factors among Community Based Health Workers and Health Facility Staff delivering UTT. The community health workers in PopART are referred to as Community HIV Care Providers (CHiPs). The study gives special attention to interactions between health workers and potential clients of HIV-related services, including key populations, who may experience particular barriers to accessing UTT and combination prevention interventions, including women engaged in exchanging sex for money or resources and men who have sex with men.

## Rationale for study

Combination HIV prevention including universal test and treat (UTT), as in the PopART study, is a complex multi-component intervention package (see Box 1 below).

Box 1: The PopART intervention

Key components of combination HIV prevention in sub-Saharan Africa include the following:

- - Strengthening of HIV testing and services at health facilities and other venues
  - Strengthening of male circumcision and prevention of mother-to-child transmission of HIV services available in the community
  - Treatment of sexually transmitted infections (STIs) and provision of condoms at health units

PopART is built on a Universal Test and Treat model which includes house-to-house deployment of community-based HIV-care providers (CHiPs) to provide:

- Universal HIV counseling and testing
- Active linkage to care for individuals diagnosed as HIV-infected, with, in Arm A of the trial, ***immediate eligibility for ART,*** and in Arm B, ***eligibility according to national guidelines***
- Promotion of male circumcision and prevention of mother to child transmission (PMTCT) services
- Referral to care for syndromic STIs
- Provision of condoms

There is much cause for optimism that PopART can be succesful. It builds on the HPTN052 research trial showing that as many as 96% of potential infections among sexual partners of people living with HIV (PLWH) could be prevented through early ART . To be effective in practice, PopART will require testing and treatment to be rapidly scaled up. HIV testing has already expanded over recent years following the widespread introduction of provider initiated testing and counselling approaches in many settings . Home-based testing programmes often delivered by community health workers have demonstrated high levels of acceptance. There has been rapid expansion of access to ART with an estimated 6.6 million people on ART by the end of 2010 . Health workers have overseen ART and testing scale-up in communities characterized by weak health systems, high levels of poverty and HIV-related stigma. These experiences suggest that PopART CHiPs and health facility staff should be able to deliver universal HIV testing treatment for PLWH. The universal approach may also successfully reach vulnerable groups for whom stigma and socioeconomic factors might otherwise be significant barriers to access.

However, there are also concerns about the viability of PopART. HIV-related stigma has contributed to the spread of the epidemic in Sub-Saharan Africa since the late 1980s . The role of stigma in potentially undermining a combination HIV prevention approach is therefore of great significance. Anticipated and internalized HIV stigma can impede uptake of HIV testing, sero-status disclosure to sexual partners and initiation and adherence to ART . Unfortuately, health facilities are often themselves a setting for HIV-related stigma. Stigmatising practices across a range of settings include neglect of patients, differential treatment, disclosure without consent, gossip, abuse and physically separating HIV-positive patients . In addition, HIV-stigma can often be multi-layered since some groups are associated with socially unacceptable behaviours and this deepens the perceived culpability that accompanies HIV infection and these groups can become a repository of blame and be at risk of exclusion from services. Further, much of sub-Saharan Africa is characterized by weak, underfunded health systems. In a recent Zambian study, some 51% of health workers self-classified as suffering from work-related burnout . These stresses can exacerbate existing problems, amplifying HIV-related stigma within health systems. The success of the PopART strategy will to a significant degree rest on the efforts of health workers, both those based in facilities and workers in the community, and other health facility staff (HFS). Stigmatising attitudes, job-stress and burnout and other factors amongst this cadre of health workers, and, conversely any stigma reduction efforts they are part of, could impact the core of the PopART intervention.

# STUDY OBJECTIVES AND DESIGN

The HPTN 071 trial aims, as a secondary outcome, to “measure the impact of the two intervention packages on HIV-related stigma”, and to use qualitative research to “document the effect of the interventions on stigma” (*Section 2.2*). The HPTN071 protocol hypothesizes that “because the main intervention in HPTN 071 is universal and is offered to the entire community, it will obviate the need for specially-targeted interventions for different risk groups, should help to avoid stigmatization, and should encourage community-wide support for HIV prevention and care” (*Section 1.2.2*).

The purpose of the work described in this protocol is to strengthen the capacity of the trial to meet these aims. We will conduct data collection complementary to, and data analysis integrated with, the main trial data (population cohort, case-control studies and wualitative research). Specifically, we will formally document HIV-related stigma in the health system and will assess how HIV-related stigma influences, and is influenced by, the delivery and uptake of the HIV-related interventions that comprise PopART.

## Objectives

1. Describe, develop scales to measure, and explore determinants of HIV-related stigma among health facility staff and CHiPs involved in the delivery of PopART, and how these change over time
2. Evaluate whether health facility staff randomized to the different arms of the trial report different levels of HIV-related stigma after three years.
3. Evaluate whether higher levels of HIV-related stigma among health-workers are associated with less successful uptake of UTT.
4. Contextualise findings through longitudinal qualitative and participatory research with a focus on understanding interactions between CHiPs, health facility staff and potential clients of HIV-related services, especially key populations.

## Study Design

This is a mixed-methods study comprising quantitative and qualitative data collection covering all 21 PopART communities over the duration of the PopART trial (three years). Quantitative data collection occurs in the form of an open cohort study of CHiPs (Arms A and B only) and health facility staff, including lay volunteers (Arms A, B and C). The cohort will be “open” in that it will continue to recruit individuals who newly meet the inclusion criteria over time. Interviews with cohort members will occur at baseline (during the first year of PopART delivery, or upon entry to the cohort), in the second year of PopART delivery, andduring the final year of the trial. Exit interviews will be undertaken with consenting individuals who leave employment with the CHiPS or relevant health facility if feasible. Longitudinal qualitative and participatory data collection uses the following methods: semi-structured key informant interviews; methods to assess context such as rapid appraisal methodologies; document analysis; non-participant observation; and other qualitative tools asappropriate. The key informants for this longitudinal, qualitative research are a total of 40 health facility staff (including volunteers) and CHiPs selected from a range of site across the three Arms. In addition, a smaller number of selected individuals who belong to marginal social groups (for example, sex workers or Men who have Sex with Men) most often excluded from HIV services will also be interviewed as key informants.

## Timing of Deployment of Research Components

# STUDY INTERVENTIONS

There are no new interventions to be delivered for this study. The PopART intervention package is described in full in the HPTN071/PopART study protocol. Training for CHiPs will include some specific training on addressing stigma within families and stigma associated with condom use and ART adherence and will ask CHiPs to reflect on their own stigma experiences. It is not a primary aim of the study to investigate the effectiveness of these stigma reduction components of the PopART intervention. There are no other specific stigma reduction intervention components within PopART.

# STUDY SETTING

A description of the 21 study communities can be found in the to HPTN071 Protocol. The selection of participants for this cohort study is described below.

This study will recruit community based health workers and health facility staff involved in the delivery of the PopART intervention package. As such, it seeks to reflect the importance of CHiPs and HFS in underpinning the success of public health interventions and HIV prevention strategies by reflecting their perspectives and experiences.

## CHiPs

In intervention communities in Arms A and B of the HPTN071 parent study, delivery of the PopART intervention will be carried out primarily by trained community health workers or ‘**CHiPs’ (Community HIV-care Providers)**. The CHiPs will provide HIV counseling and testing and active linkage to comprehensive care and prevention services to community members 16 years of age and older in Zambia and 12 years of age or older in South Africa, and any minors younger than these ages who request a test, with the consent of their guardians. The CHiPs team will approach each and every household that is accessing a government health facility, with pairs of CHiPs workers being responsible for implementing the intervention in an assigned subset of households or ‘zone’. The household will be asked to participate in home-based HIV counseling and testing and consider other HIV prevention initiatives, including medical male circumcision (MMC) for HIV-negative men and prevention of mother to child transmission treatment (PMTCT) for pregnant women who test positive for HIV. Each CHiPs team will consist of a gender-balanced group of individuals trained in HIV counseling and testing, and other aspects of HIV prevention and care. During the trial CHiPs will be employed by the two research organizations in Zambia (Zambia AIDS Related Tuberculosis Project [ZAMBART]) and South Africa (the Desmond Tutu Tuberculosis Centre [DTTC]).

### Health facility staff

In all Arms (A, B and C) of the HPTN071 trial, staff working in the main health facility which defines each cluster will be recruited to the study. **HFS (Health Facility Staff)** in all arms will be those potentially involved in the delivery or support of HIV testing and ARV treatment services, or individuals with whom potential clients may interact in accessing these services. Unlike CHiPs, these staff will be employed by other implementing partners including government and key non-governmental organizations (NGOs).

# Research PROCEDURES and activities

## Cohort study of Health facility staff and CHiPs

### Inclusion Criteria

- At least 18 years of age
- Able to provide informed consent for participation
- In Arm A and B only, CHiPs workers delivering the PopART intervention
- In all Arms (A, B and C), health facility staff who come into direct contact with patients. Health facility staff will include staff who are always based within health facilities as well as those who primarily work in the field but are part of the local community health services mechanism.
- Provide informed consent.

### Exclusion Criteria

Any condition that, in the opinion of the Investigator of Record or designess, would make participation in the study unsafe, complicate interpretation of study outcome data, or otherwise interfere with achieving the study objectives. This might include instances where a respondent would have to travel in unsafe conditions to complete the survey or if the risk of social harm due to a respondent’s participation in the survey is particularly high.

### Selection of Study Participants

#### CHiPs

CHiPs workers will be automatically considered eligible for the study upon recruitment to the PopART intervention team.

#### Health facility staff

The study coordinator will liaise with the community engagement office and intervention coordinator to enumerate site-specific groups of HFS. Interviews with health facility managers and a document review process will enumerate health workers and screen against the inclusion criteria in each facility. Examples of staff roles expected to be included in the study include:

- - Doctor
  - Clinical Officer
  - Clinical Nurse Practitioner
  - Senior Professional Nurse
  - Professional Nurse, including TB professional nurses
  - Enrolled Nurse
  - Enrolled Nurse Assistant, including TB ENA
  - Midwives
  - Lay Counsellors
  - Facility Manager
  - ARV Clerk
  - TB Clerk
  - Clerk / Admin Clerk
  - Counsellor
  - General and TB Assistant
  - Cleaner
  - Pharmacist
  - Assistant Pharmacist
  - Psychologist

A list of identifier numbers for all eligible health workers in each cluster will be compiled in an appropriate computer-based format for use in keeping records of study completion rates.

### Negotiating access to potential participants

#### CHiPs

In each country, the study coordinator will liaise with the intervention coordinator(s) to draft a schedule for data collection. In principle, the schedule of study activities should minimise impact on CHiPs implementation of the PopART intervention.Some flexibility between country, site and CHiPs team differences is expected. Once the schedule is agreed, the study coordinator will liaise with CHiPs teams’ leaders to confirm dates, times, and venues to complete study activities. A brief description of the study will be provided to the CHiPs teams’ leaders which they will convey to the team members. Team members will decide if they would like to participate in the study.

#### Health Facility Staff

In each country, the study coordinator will follow appropriate, site and group specific institutional channels to approach HFS. This may include enlisting the assistance of Department/Ministry of Health and NGO staff in recruiting potential participants to meetings; a brief description of this study will be provided to facilitate this assistance. The schedule should minimise impact on HFS routine work. HFS will decide if they would like to participate in the study.

All individuals must sign a written informed consent form before any study procedures can begin. Participants will retain one copy of the form (if they wish) and the other copy will be filed with the site’s study documents.

### Data Collection

Where possible the intervention site office space should be used as a venue for CHiPs interviews if this can be done without compromising participants’ experience of confidentiality. Similarly, space convenient to the HFS should be used, for example at the facility for clinic staff where appropriate. Every effort will be made to ensure that study data remains confidential while on site at the site office. If a respondent feels particularly vulnerable completing the survey at the study site, we will make arrangements for that respondent to complete the survey at an alternate location.

The field researcher will access the appropriate version of the stigma survey on a personal digital assistant (PDA) and then hand the PDA to the participant to complete the survey. The field researcher will remain close by to assist participants with issues of clarity.

A data collection tool will be designed for facilitated self-delivery using PDAs [see Section 5.1.6 – Data Management]. HFS and CHiPs will receive similar questionnaires, however, some questions may be limited to one group or the other, given the different environments in which they work. These PDAs will be password protected and will only be available to authorized staff. Electronically kept personal identifiers will be stored in separate datasets with password protection only accessible for designated staff (for computers and servers). We anticipate that in some cases, participants might prefer to answer a paper version of the questionnaire. In such cases, paper questionnaires will be available. Research Assistants will be on hand to answer any questions and ensure confidentiality while the participant is completing the survey. The responses will later be entered by a member of research staff. Paper questionnaires will be signed and dated by the participant and will be stored in a locked cabinet, only accessible to study staff.

In some circumstances, if a participant is unable to use the EDC (i.e. if a participant is illiterate) or if a participant is not comfortable doing the self-administered survey as programmed on the EDC, a Research Assistant may administer the questionnaire to the participant, and fill in the information on the EDC.

At baseline, the tool will cover a range of topics including the following:

Sociodemographics

Employment detail

Knowledge and opinions about HIV transmission and prevention, ARVS, barriers to testing and treatment

Job-stress and stigma: Exhaustion, accomplishment and depersonalization

Personal experience of HIV testing & own HIV status

Facility practice with management of PLWH

Facility practice with regard adolescents

Observation of stigma occurring in own health setting

My own practice treating PLWH

Personal opinions associated with risk of HIV

Delivering HIV testing in antenatal care and PMTCT

Providing services to high-risk women and female sex workers

Providing services to men who have sex with men

Providing services to sexually active adolescent

Experience of Stigma by association because I am involved in PLWH care

HCWS who are also PLWH: responses to testing positive, experience of stigma and discrimination

Perceptions of Community support for UTT

The questionnaire includes items from best practice tools, and will be designed to complement data collection from community members and PLWH that are to be collected within the PopART Population Cohort, reflecting a “parallel” approach to data collection that will capture the perspectives of multiple actors. The interview is anticipated to last no more than one hour.

Throughout the data collection process, particular care will be taken by the fieldwork staff to explain that some of the questions asked are of a sensitive nature and that while the PDA device is being used to help ensure privacy and comfort in answering such questions the field staff are more than willing to assist at any point. Further, participants will be encouraged to take as long as they need to answer questions honestly and to ask for clarification from the field staff at every point at which this is helpful. During the written informed consent process the fieldwork staff will verbally articulate the section headings to the questionnaire. The fieldwork staff’s sensitive delivery of the data collection process is a key aspect of training and QC managed by the in-country coordinator with the assistance of the PopART social science leadership.

### Data management

Data collected on the devices will be downloaded within one day of each interview to password protected secure computers under the management of the PopART data team. Data will be checked and cleaned centrally. The interview process and survey progress will be frequently de-briefed and closely monitored by an in-country researcher. Data will be stored securely at a location away from the study sites. Respondents will be identified by a unique identifier in the database.

### Retention

In order to accurately document any shifts over the duration of the study, data collection within the cohort will occur for each individual during the first year and then after 1 and 3 years of PopART delivery. At each year, individuals who have left the cohort (through death, migration or no longer meeting the study inclusion criteria) will be documented and replaced in the cohort by individuals newly meeting the study inclusion criteria.

Once a participant is enrolled, the research team will make every effort to retain him/her for the follow-up surveys in order to minimize possible bias associated with loss-to-follow-up. Research staff are responsible for developing and implementing local standard operating procedures to reach this goal. Components of such procedures include:

- Thorough explanation of the study visit schedule and procedural requirements during the informed consent process, with re-emphasis at subsequent visits.
- Thorough explanation of the importance of their participation to the overall success of the study.
- Immediate and multifaceted follow-up for missed visits.
- Regular communication with CHiPs/HFS at large to increase awareness about the purpose of HIV prevention research and this anciallary protocol and the importance of completing research study visits.

In addition to the components described above, which are standard for all HPTN studies, the team will work with local stakeholders, experienced in-country staff, and participants themselves to identify locally-effective, study-specific strategies for improving participant retention. Such approaches may include use of short message service (SMS) messages to remind participants about upcoming visits.

Any member of the cohort who leaves their role in delivering the UTT intervention, thereby no longer meeting the inclusion criteria, will be censored. Exit interviews will be conducted close to the date of censoring if feasible.

Participants may voluntarily withdraw from the study for any reason at any time. The Investigator also may withdraw participants from the study in order to protect their safety and/or if they are unwilling or unable to comply with required study procedures after consultation with the Protocol Chair, Division of AIDS (DAIDS) Medical Officer, Statistical Data Management Center (SDMC) Protocol Statistician, and Coordinating and Operations Center (CORE) Protocol Specialist.

Participants also may be withdrawn if the study sponsor, government or regulatory authorities, or site Institutional Review Board (IRB)/Ethics Committee (EC) terminates the study prior to its planned end date.

Efforts will be made to document participants reasons for leaving the study since HIV-related stigma surrounding the study is one of the reasons that a participant may choose to withdraw. It is therefore important that this information is captured and recorded.

## Qualitative research

### Aim

To provide contextual explanations of how interactions between CHiPs/HFS and clients are influenced by HIV-related stigma and other factors such as workload, modes of delivery of the PopART interventions, job-stress, burnout and exposure to stigma-related training.

### In-situ key informant interviews and observation

Approximately 40 key informant health workers (15 CHiPs, 15 HFS and 10 supervisors or managers of this group) will be interacted with on an individual basis each year over four years of PopART implementation. Included in these interactions will be a semi-structured interview as well as observation of practice and spaces and use of participatory research tools to facilitate discussion. These interactions will serve as a starting point for describing participants’ experience of implementing healthcare (generally) and PopART (specifically) in relation to HIV-related stigma. Each participant will be followed further according to ethonographic research principles and as appropriate. The analytic aim is to document perspectives on experiences, events and contextual factors relevant to our overall objectives and how these change over time as PopART is rolled out.

In addition to the 40 key informant health workers (above), in the first year, a series of life history/illness narrative interviews will be undertaken with approximately two ‘key informant’ members per a diversity of CARE groups (including, but not limited to, groups such as sex-workers and MSM). These interviews will take place over four study visits, spread evenly over 3-4 weeks. The interviews will last approximately 30-45 minutes, depending on the respondent’s availability. If there are additional things that the respondent would like to tell us about his or her experience, it is possible that we may spend more time with the respondent as needed. These interviews will form the basis for iterative, multi-method research in subsequent years in those CARE groups assessed to be highest priority for understanding the importance of HIV-related stigma in the delivery of the PopART intervention (specifically) and UTT (in general).

Participant selection

The health workers will be purposively sampled for diversity (in age, gender, time working as a health worker, cultural group, trial Arm and site community type). It should be noted that not all PopART sites will be involved in this qualitative longitudinal component. We will select participants from a smaller number of sites within countries across the Arms of the trial that represent different local contexts. See Bond et al, 2013, Technical Worki ng Report for a description of similarities and differences across site communities. The sample is not intended to describe a representative of either ‘most’ or ‘an average of’ health workers opinions nor to be comparative of this between study communities or between triplets. Rathar it is intended to access as broad a range of narratives about HIV-related stigma and PopART implementation as possible to contextualize this in its plurality. In other words, the aim is to select a group who are qualitatively representative of a range of local contexts and a range of opinions. Recruitment will continue until the point at which new participants are not revealing anything new at a conceptual level, ie saturation in HIV-related stigma narratives is achieved .

The CARE groups will be site-specific and identified in the qualitative cohort of health workers and on-going social science in the PopART trial. The key informants will selected on the basis of ‘critical-case sampling’. Critical case sampling is a form of purposive sampling where participants are recruited on the basis of prior expectation that they are knowledgeable or have much experience of the phenomena or topic being studied.

Potential participants will be identified in collaboration with the in-country PopART trial intervention working groups, the social science working groups and the community engagement working group. HFS/CHiPs will be approached with the consent of their supervising body (that is, the Ministry/Department of Health for HFS and Zambart/DTTC for CHiPs), but the identity which individual participants are approached, and which of those approached consented or declined to participate will be protected from these supervising bodies; this is to protect HFS and CHiPs from coercion into the stigma study. Further, all participants will participate only on a voluntary basis and in accordance with all principles of good clinical practice.

Procedures

Research assistants will conduct interviews guided by a semi-structured interview guide; interview guides will have predefined main themes, but these guides are meant to be dynamic and will allow for new topics to emerge during the course of the interview. The interviews will focus on how the work of the participants contributes to the accessibility, acceptability and equity of UTT package interventions to and in particular how HIV-related stigma, health worker burnout, exposure to stigma-reduction activities and other factors influence these. There will also be guides for the observation component of the study.

Data Collection Tools

Interviews will semi-structured and will be digitally recorded and transcribed. Where appropriate to the data collection process, and again with the consent of participants, still photography and video will be used to complement the audio data.

### Complementary research approaches to document context

1. Field researchers wil be encouraged to keep notebooks as they implement the study and we will regularly undertake structured documentation, observation and routine debriefing of field staff.
2. Where feasible, we will observe public or study-specific events such as stigma-reduction trainings relevant to the aims of this study, taking field notes and conducting rapid debriefs of the experiences of people involved
3. Study documentation, media articles and other documentation relevant to the aims of this study will be collected by the research team, including manuals and field notes which will be collected and indexed on a weekly basis into an archive so as to be available for narrative analysis.
4. Other methodologies as appropriate.

### Data management for qualitative data

All qualitative data (including audio, video, and photographic recordings, structured and unstructed field notes, and reflexive notes) as well as all formats of this as it is processed, including recording files, transcriptions, translations, and scanned copies will be maintained by in-country social science data management staff. Efforts will be made to ensure confidentiality throughout the data recording process. All such data will be stored in triplicate in password protected storage devices (when electronic) and in locked, secure cabinets with restricted access (when in hard forms) in order to ensure confidentiality of data. All such data will be handed over to the social science data management staff on a routine basis for indexing and storage. At this hand over, all electronic data will be deleted off the memory cards of devices such as voice recorders and cameras. Data processing for analysis (for example scanning, or transcription) is delegated to appropriate social science staff and will be managed, including quality control and quality assurance) by ZAMBART and DTTC. 10% of all data entry (including transcription) will be audited by senior social scientific staff prior to analysis.

We will ensure that data systems and data handling procedures for capturing, transferring, analyzing and storing electronic data obtained from health centers are developed in such a way that will preserve participant confidentiality. Electronic systems in which these data are kept will be password protected and will only be available to authorized staff. Electronically kept personal identifiers will be stored in separate datasets with password protection only accessible for designated staff (for computers and servers). This will ensure that even if electronic devices are lost or stolen, no one without a specific password will be able to access the data.

# Statistical Considerations and Data Analysis

## Sample size for cohort study

We will include all CHIPS workers and all health facility staff in all 21 study clusters that meet the inclusion criteria described in Section 5.1.1 in the study. At baseline, we estimate that over 2000 individuals will be eligible to participate.

We expect the total number of CHiPs workers across all communities to be approximately 736, with some variability by cluster (estimated range 40-80) reflecting variation in the size of the clusters. We expect the total number of HFS to be approximately 1300 reflecting an average of 60 individuals per cluster again with some variability linked to cluster size (estimated range 15-40).

### Justification of sample size

We considered both statistical and pragmatic / feasibility issues in deciding on sample size. The primary justification for including all individuals meeting the inclusion criteria was as follows:

- The number of clusters was pre-set by the HPTN071 study (n=21)The data from the cohort study will be used both descriptively and to explore a range of research questions
- Including all potentially eligible participants in the study (CHiPs and HFS) will give the most precise estimates for descriptive aims and the maximum possible power to test hypotheses
- Including all CHiPs/HFS gives high statistical power to test key hypotheses under plausible scenarios. However, power will be low in other scenarios. (Details in Appendix). Consequently, we decided that we would only include fewer than the maximum possible sample size would only be considered if including all CHIPS/HFS was not feasible.
- Including all CHiPs /HFS meeting the inclusion criteria is feasible. In initial budgeting work and consultation with the members of the PopART intervention team in both South Africa and Zambia on the enumeration and process of interview of CHiPs /HFS, the strongly expressed view was that it would be feasible to interview all of the CHiPs workers in Arms A and B, and the health facility staff in all Arms (A, B and C) meeting our inclusion criteria. The intervention teams felt that arranging a one-hour PDA-based facilitated self interview with these individuals each year would be relatively simple to achieve, and expressed the view that there would be some feasibility advantages to doing this with the whole group rather than a sample.

## Data Analysis

A full statistical analysis plan wil be developed and documented after the baseline surveys.

### Objective 1

The first stage of analysis after both the baseline and subsequent surveys will be to describe the study population and individual responses in detail. Responses to individual items will be tabulated and grouped where appropriate. The population of CHiPs/HFS will be described in terms of key characteristics such as sex, age, roles and educational background. Variability in characteristics between types of participants (CHiPs vs HFS), countries, study clusters and changes over time will be explored. Using data on the estimated dates on which individual cohort members leave active service following recruitment to the cohort and are replaced by other individuals, we will describe the turnover and rates of attrition of healthworkers and how this varies by key sociodemographic characteristics, including by type of participant, country, cluster and over time. Responses to individual items in the questionnaires across a range of domains will be tabulated. Variation by sociodemographic factors, type of participant, country, study cluster and, in later analyses, over time will be explored and described statistically.

We will develop scales to measure HIV-related stigma and specify primary and secondary indicator variables for our final analysis prior to follow-up analyses. The choice of primary and secondary variables for analysis will be influenced by:

- - qualitative research conducted over the first year of the study
  - the distribution of responses to individual items at baseline
  - selection of an appropriate scale development approach using baseline data

In the following sections, to illustrate how the analysis will be approached, consider a potential outcome variable: the proportion of participants who report judgment of PLHA (e.g. that they agree with any of: HIV is punishment for bad behaviour,People with HIV should be ashamed of themselves, HIV is a punishment from God.) In a study in Tanzania, 21% of health workers were classified as having “judging” attitudes (Nyblade) on the basis of this indicator .

### Objective 2: does the PopART intervention reduce HIV-related stigma among health-facility staff?

A successful UTT strategy may reduce HIV-related stigma in communities and health settings. We will analyse the data to explore whether health facility staff working in clusters randomised to the different arms differ at the end of the study in terms of HIV-related stigma (and other key variables). This analysis will not include data from the CHiPS since these individuals are not present in Arm C.

Because the number of clusters per arm is small, we will use methods based on Student's t-test, which have been shown to be highly robust for small numbers of clusters. We will compute cluster summaries of HIV-related stigma based on the scale development approaches described above. To test the null hypothesis of no impact, the paired t-test will be applied to these summary measures (7 matched pairs for each comparison), with 6df

Evidence for intervention effect will also be assessed using a non-parametric permutation test, based on the list of all possible allocations of trial arms to communities that met the restricted randomization criteria. For each of these possible allocations, and including the allocation that was randomly selected, measures of effect (comparing Arm A and Arm B, Arm B and Arm C, and Arm A and Arm C) will be calculated as above. The number of allocations (n) for which the measure of effect is as or more extreme than the value observed in the trial will be counted, and a 2-sided p-value calculated as n divided by the total number of possible allocations.

### Objective 3: are higher levels of HIV-related stigma among CHiPs/HFS associated with less successful delivery of the PopART intervention?

HIV-related stigma in health settings might act as a barrier to the effectiveness of PopART. To operationalise an approach to addressing this hypothesis will require an analysis strategy that breaks the PopART randomisation. We will exploit naturally occurring variation in levels of HIV-related stigma among health workers and explore whether this variation is associated with uptake of the PopART interventions after adjusting for potentially confounding factors. Our analysis strategy is described below.

For this analysis:

- Indicators of HIV-related stigma as developed for Objective 1 will be treated as exposure variables
- The key “outcome” variables will be measures of the uptake of key components of the PopART intervention package over years 1-3, requiring outcome data drawn from the CHiPs database and population cohort (eg HIV testing, linkage to care, initiation of ART and adherence to ART). The operationalisation of these outcome variables will be finalized in line with plans for the main trial analysis since several of these variables are specified as secondary outcomes.
- We will use cluster, clinic, CHiPs zone and individual-healthworker-level analysis strategies that link the responses of health workers on HIV-related stigma to the uptake of the interventions they offer, promote and support in the zones and clusters in which they work.
- Since the approach will break the randomisation it will be necessary to adjust for potential confounding factors that may also influence the uptake of PopART interventions, including trial arm, country and sociodemographic factors among both CHiPs/HFS and the target populations

## Objective 4: Analysis of Qualitative data

Qualitative analysis will be on-going throughout. Content, thematic and discourse analytic methods will be used, as appropriate. Qualitative data analysis will follow analytic goals determined by iterative engagement with the research communities. All analytic outcomes will be processed and checked centrally, and reviewed by at least two members of the research team.

Following transcription of interviews, research assistants will enter interview transcripts and text from memos into a text organizing program that facilitates content analysis. We will identify core consistencies and meanings in the data through careful repeated reading of interview texts. Sections of text will be labeled based on themes and particular domains of interest related to the influence of stigma, health system and other factors to key trial outcomes. The first 5 interviews / documents analysed annually will be chosen for “open coding,” a procedure using an inductive approach to elucidate patterns and themes in the data to generate codes for subsequent analysis. New themes can be generated from the analysis in addition to those established a priori. Early coding will be followed by a more deductive process whereby relevant text from the remaining interviews is coded. The coded text will then be reviewed and synthesized through development of hierarchical codes, matrices to structure the main themes, and use of the constant comparative method. Quotes representing each theme will be reviewed again within the context of the full text of the interview to contextualize and confirm interpretation of the survey items. Results will be summarized by main themes and reviewed by investigators continually throughout the study, with the goal of refining the quantitative tools to ensure new stigma manifestations that may arise in the context of UTT are being captured as well as understanding how stigmatizing attitudes are changing over time.

Key areas of analytic focus will include

- Which factors inhibit and/or facilitate HIV related stigma at community level?
- How are events relevant to stigma contextualised in terms of HIV services and community life?
- What is the ‘information to action cascade’ from the intention of an event, what participants understood of this, how this understanding was internalised, and what action they took subsequently?
- How are members of key populations and their interests represented by, and represented in events relevant to stigma?
- How is stigma embodied in the clinic space? How is it challenged?
- How is stigma embodied in HIV clinic practice? How is it challenged?
- How does stigma experienced by CHiPs and HFS who are themselves living with HIV impact on stigma?
- How is the extension of health services into the community impacting on the clinic/community space and the experiences of people ‘wearing both hats’?
- How do CHiPs and HFS engage with members of key populations in their professional and private capacities?
- How do members of key populations experience the stigma in their relationships with CHiPs and HFS and as clients of this system?
- How do members of key populations experiences of HIV prevention, treatment and care relate to (reinforce, redefine, or escape) their perceived social position? How does it manifest in their movement in the clinic and community spaces?

# SAFETY MONITORING AND SOCIAL HARM/Adverse EVENT REPORTING

## Safety Reporting

Because this study includes no biomedical intervention or study product, standard adverse event reporting will not be undertaken and no adverse event data will be collected on case report forms for entry into the study database. However, in accordance with 45 CFR 46, unanticipated problems or serious adverse events that are judged to be related or possibly related to study participation will be documented and reported to the IRB/ECs according to their individual requirements and to the DAIDS Medical Officer and the Office of Human Research Protections (OHRP). This reporting will be performed according to the timelines and definitions included in pre-established written procedures, such as the study procedures manual and SOPs, and the guidelines provided at http://www.hhs.gov/ohrp/policy/advevntguid.html. Serious adverse events will not be reported to the DAIDS Regulatory Compliance Center (RSC).

In order to prevent adverse social events related to study participation, social harms will be monitored throughout the study. Social harms are any untoward social occurrences that happen to a participant as a result of their participation in the study. Examples include loss of employment, harassment by neighbors, shunned by family, rejection by partner, etc. Although social harms due to this study are expected to be negligible, they will be monitored closely throughout.  Information on social harms will be actively solicited from participants at follow-up visits and recorded on case report forms and captured in the study database. Participants will also be encouraged to report any social harm on an ad hoc basis when it occurs.  In the event that a participant reports social harm, every effort will be made by study staff to provide appropriate care and counseling to the participant, and/or referral to appropriate resources for the safety of the participant as needed. Social harms that are judged by the Investigator of Record to be serious or unexpected will be reported to the responsible site’s IRB/EC at least annually, or according to their individual requirements.  The nature and frequency of these social impact reports will be monitored by the protocol team on a regular basis.

# HUMAN SUBJECTS CONSIDERATIONS

This is an observational study and participation is completely voluntary. The information gained from this study may help inform future community-wide HIV prevention strategies. Community HIV Care Providers (CHiPs) and health facility staff are the critical groups delivering the PopART strategy. Any population health benefits achieved through a UTT approach will depend on the capacity of health systems to recruit, train and retain workers to promote and deliver the interventions in a way that is accessible and acceptable to the target populations and achieves high uptake. It is therefore of critical social value to understand the characteristics and experiences (such as stigma, job-stress and burnout) of workers supporting the delivery of these interventions, and assess how these change over time and whether they are associated with its success.

The perspectives of those delivering interventions are often poorly documented in research trials which can cause limitations both in the interpretation of trial results or in the recommendations made from such studies. However, studies of intervention delivery are increasing they are recommended as part of process evaluation with trials of complex interventions and, to date, include several studies of HIV-related stigma and burnout within health-care settings .

All study procedures will be approved by the local and other appropriate IRBs before implementation. All study staff will receive the appropriate human subjects and good clinical practice training before they participate in any of the human subjects research aspects of the project. Any definitely, probably, or possibly related serious adverse events will be reported to the DAIDS Medical Officer and to the site’s IRBs/ ECs, per their requirements and pre-established written procedures.

## Benefits

HIV-related stigma in communities and health-settings poses a significant challenge to the success of HIV prevention and treatment efforts in sub-Saharan Africa. This study comprises one critical component of a portfolio of work nested within the HPTN071 (PopART) trial that seeks to explore how HIV-related stigma affects and is affected by the interventions under study. As such, just as the HPTN071 (PopART) trial will provide evidence to support the UNAIDS goals of “Zero new infections” and “Zero AIDS related deaths”, so the inclusion of the work focused on HIV-related stigma will provide evidence to support UNAIDS goal of “Zero Stigma and Discrimination” .

The results of the study should provide important information about the characteristics and experiences of health workers supporting the delivery of the HPTN 071 (PopART) interventions. In this respect the study is innovative and provides potential benefit to an often over-looked group who participate in research studies and for whom the results of such studies have important implications: those who deliver public health interventions. The perspectives of this group will therefore be able to inform recommendations made by the study, with a view to ensuring that any future rollout will consider the effects on health staff of their participation in delivery of the intervention.

At the individual level, interviews conducted for this study will provide an opportunity for reflection on the process of promoting and delivering components of the UTT strategy. As we describe below, particular concerns raised by participants in these interviews can be provided to the study coordinating team for consideration (through systems for monitoring social harms), which has the potential to improve the working lives of this group during the study period.

## Risks

Since the study involves no new interventions, the risks to participation are both likely to be non-severe and have a low probability of occurring.

However, studies of intervention delivery do pose particular risks, which we outline here followed by our proposed approaches to minimize these risks.

One set of risks arises from the organizational set-up within which the proposed research will occur. The research team, forming part of the Social Science research team for HPTN 071 (PopART), will seek informed consent for participation in the research from both CHiPs workers and health facility workers delivering the HPTN 071 (PopART) interventions. In some, though not all, cases the same institution employs both the researchers and the proposed participants. This organizational arrangement requires that our systems:

(i) Ensure that participants do not feel coerced into participation. Potential participants may be vulnerable in this respect for at least three reasons:

- Juridic vulnerability – that is they may feel liable to the authority of others who may have an independent interest in their participation
- Deferential vulnerability – that is they may be used to acting in a subordinate manner as part of a hierarchical system
- ‘Pay check vulnerability’ – that is they may believe that their position as employees could be under threat if they refuse participation) .

(ii) Ensure the confidentiality of responses. Participants may also be concerned that the answers they give to questionnaires may not be kept truly confidential. In particular they may worry that responses given in the questionnaires or interviews will find their way back to individuals with the power to alter the terms of their employment.

A second set of risks relates to the impacts of participation. It is possible that gossip between participants may include discussion of the ways in which individuals or groups of individuals, for example staff members of a particular clinic, responded to the questions in the surveys. If there are any breaches of confidentiality, or if rumours gather that certain individuals may have responded in certain, undesirable ways to questions on the surveys, this may cause unpleasant gossip for participants. In addition, participation itself (including answering questions about potentially challenging work situations) may encourage participants’ reflection on the difficulty of their work environment or their experiences of stigma.

#### Minimizing Risks

We will minimize the risks identified above through:

- Robust and transparent procedures for fully-informed consent
- Maintaining as much confidentiality as possible of individual responses at all stages
- Referral of participants-in-need to support or counseling services
- Not using data acquired through this study for M&E purposes
- Monitoring participants’ experiences of participation
- Independent Ethical Review

## Informed Consent

Informed consent will be sought and obtained using a standardised process that will emphasise both to proposed participants and their colleagues that those approached for informed consent are fully at liberty to participate or not participate in the study. Details of any individual’s participation/non-participation will be kept confidential, and if information about participation/non-participation does become known or suspected this will have no implications for any aspect of their employment status.

Written informed consent will be obtained from each study participant. Each study site will be responsible for developing a study-specific informed consent form for local use, based on the template in Appendix I, that describe the purpose of the study, the procedures to be followed, and the risks and benefits of participation, in accordance with all applicable regulations. The HPTN CORE will work closely with the study site to ensure that the informed consent forms are translated into local languages, and that the accuracy of the translation is verified by performing an independent back-translation.

Literate participants will document their provision of informed consent by signing their informed consent forms. Given the study population, we do not expect many participants to be non-literate. In the event of non-literate eligible participants being identified, these individuals will be asked to document their informed consent by marking their informed consent forms (e.g., with an X, thumbprint, or other mark) in the presence of a literate third party, impartial witness. (Further details regarding DAIDS requirements for documenting the informed consent process with both literate and non-literate participants are provided in the *Requirements for Source Documentation in DAIDS Funded and/or Sponsored Clinical Trials*.) Any other local IRB/EC requirements for obtaining informed consent from non-literate persons also will be followed.

Participants will be provided with a copy of their informed consent forms if they are willing to receive them.

### Confidentiality

Strict measures will be in place to safeguard confidentiality of data.

This starts with the approach used for data collection. Participants will be trained in the use an electronic data collection device (EDC) to enter responses to questions in a “self-interview”. Consequently participants will not need to share their responses to any questions with any other individual when using the EDC. EDC data collection will not be visible at either the point of entry or prior to data upload to those facilitating self-interview sessions, except in some circumstances described below.

In some cases, participants might prefer to answer a paper version of the questionnaire. In such cases, paper questionnaires will be available. Research Assistants will be on hand to answer any questions and ensure confidentiality while the participant is completing the survey. The responses will later be entered by a member of research staff. Paper questionnaires will be signed and dated by the participant and will be stored in a locked cabinet, only accessible to study staff.

In other circumstances, if a participant is unable to use the EDC (i.e. if a participant is illiterate) or if a participant is not comfortable doing the self-administered survey as programmed on the EDC, a Research Assistant may administer the questionnaire to the participant, and fill in the information on the EDC.

All study data collection, process, and administrative forms will be identified by coded numbers only to maintain participant confidentiality.

Personal identifiers (name, address, global positioning system coordinates) will only be collected for (1) informed consent and (2) operational and logistical purposes (i.e. to ensure tracing of participants by intervention staff and to locate cohort participants for follow-up visits). Personal identifiers will appear on paper or electronically on appointment books, consent forms, log books, follow up lists and other listings. These listings will not include any (sensitive) study information .A unique study number will be used to link personal identifiers to study information.

Personal identifiers on paper will be stored in a locked cabinet. Electronically kept personal identifiers will be stored in separate datasets with password protection only accessible for designated staff (for computers and servers). Hand-held devices will also be password protected and personal identifiers will be stored in an encrypted format.

Participants’ study information will not be released without the written permission of the participant, except as necessary for monitoring by the National Institute of Allergy and Infectious Diseases (NIAID) and/or its contractors, representatives of the HPTN CORE (Coordinating Operations Center), other government and regulatory authorities, and/or site IRBs/ECs. Datasets transferred to locations outside the study sites (e.g. for analyses, progress reports) will be stripped of any personal identifier before transfer.

All electronic data will be stored in password protected database systems. Read and write authorization of data will depend on the designation of the staff member. A second layer of protection is hardware password protection on computers, servers and networks. Thirdly data transfer over wireless or mobile networks will use Virtual Private Networks or router protected dedicated internet protocol addresses.

All collected study data on central computers and servers, remote computers and hand-held devices, will be backed up daily. Backup tapes/discs will be stored separately from the primary electronic storage.

### Referral to independent counseling services

Every effort will be made by the study staff to provide appropriate counseling to the participants, and/or referral to appropriate independent resources as required.

### Data not to be used for M&E purposes

Data from the study will not be used for monitoring and evaluation purposes, nor for the identification of individuals, health-settings or communities with particular characteristics.

### Monitor experiences of participation

Given the potential concerns about coercion and potential harms arising from participation in this study, the research team will compile information including the following and make this available for review.

- Consent and data management procedures
- Data on response rates and reasons for non-participation
- Data from participants on the experience of participation
- Dissemination plans

### Independent Ethical Review

Approval to conduct this study will be obtained from the following IRBs/ECs. In instances where there is disagreement or discordant IRB requirements the condition providing the highest level of human subject protection will be implemented. Approval must be obtained from the local, and national (where relevant) IRBs before the study can be initiated.

Ethical clearance for the trial will be sought from IRBs in the United Kingdom (UK), Zambia, South Africa and the United States (U.S.). Adverse events will be reported on a regular basis according to the individual requirements of these IRBs.

### Study Discontinuation

The study may be discontinued at any time by NIAID, the HPTN, other government or regulatory authorities, and/or site IRBs/ECs.

# ADMINISTRATIVE PROCEDURES

## Protocol Registration

Prior to implementation of this protocol, and any subsequent full version amendments, each site must have the protocol and the protocol consent form(s) approved, as appropriate, by their local IRB/EC and any other applicable regulatory entity (RE). Upon receiving final approval, sites will submit all required protocol registration documents to the DAIDS Protocol Registration Office (DAIDS PRO) at the Regulatory Services Center (RSC). The DAIDS PRO will review the submitted protocol registration packet to ensure that all of the required documents have been received.

Site-specific informed consents forms (ICFs) WILL NOT be reviewed or approved by the DAIDS PRO, and sites will receive an Initial Registration Notification when the DAIDS PRO receives a complete registration packet. Receipt of an Initial Registration Notification indicates successful completion of the protocol registration process. Sites will not receive any additional notifications from the DAIDS PRO for the initial protocol registration. A copy of the Initial Registration Notification should be retained in the site’s regulatory files.

Upon receiving final IRB/EC and any other applicable RE approval(s) for an amendment, sites should implement the amendment immediately. Sites are required to submit an amendment registration packet to the DAIDS PRO at the RSC. The DAIDS PRO will review the submitted protocol registration packet to ensure that all the required documents have been received. Site-specific ICF(s) WILL NOT be reviewed and approved by the DAIDS PRO and sites will receive an Amendment Registration Notification when the DAIDS PRO receives a complete registration packet. A copy of the Amendment Registration Notification should be retained in the site's regulatory files.

For additional information on the protocol registration process and specific documents required for initial and amendment registrations, refer to the current version of the DAIDS Protocol Registration Manual.

## Study Activation

Pending successful protocol registration and submission of all required documents CORE staff will “activate” the site to begin study operations. Study implementation may not be initiated until a study activation notice is provided to the site.

## Study Coordination

Study implementation will be directed by this protocol as well as the SSP manual. The SSP manual — which will contain reference copies of the *Requirements for Source Documentation in DAIDS Funded and/or Sponsored Clinical Trials*, as well as the DAIDS Manual for Expedited Reporting of Adverse Events to DAIDS, Version 2.0, dated January 2010 and the DAIDS Toxicity Tables — will outline procedures for conducting study visits; data and forms processing; AE assessment, management and reporting; dispensing study products and documenting product accountability; and other study operations.

Study case report forms, electronic data capture tools, and other study instruments will be developed by the protocol team and HPTN SDMC. The study data from all sources ultimately will be transferred to the HPTN SDMC for storage and analysis. Quality control reports and queries will be generated and distributed to the study sites on a routine schedule for verification and resolution.

Close coordination between protocol team members will be necessary to track study progress, respond to queries about proper study implementation, and address other issues in a timely manner. Rates of accrual, adherence, follow-up, and AE incidence will be monitored closely by the team as well as the HPTN Study Monitoring Committee. The Protocol Chair, DAIDS Medical Officer, Protocol Biostatistician, SDMC Project Manager, and CORE Protocol Specialist will address issues related to study eligibility and AE management and reporting as needed to assure consistent case management, documentation, and information-sharing across sites.

## Study Monitoring

On-site study monitoring will be performed in accordance with DAIDS policies. Study monitors will visit the site to

- Verify compliance with human subjects and other research regulations and guidelines;
- Assess adherence to the study protocol, study-specific procedures manual, and local counseling practices; and
- Confirm the quality and accuracy of information collected at the study site and entered into the study database.

Site investigators will allow study monitors to inspect study facilities and documentation (e.g., informed consent forms, health center and laboratory records, other source documents, case report forms), as well as observe the performance of study procedures. Investigators also will allow inspection of all study-related documentation by authorized representatives of the HPTN CORE, SDMC, NL, NIAID,US and in-country government and regulatory authorities and IRBs/ECs. A site visit log will be maintained at the study site to document all visits.

## Protocol Compliance

The study will be conducted in full compliance with the protocol. The protocol will not be amended without prior written approval by the Protocol Chair and NIAID Medical Officer. All protocol amendments must be submitted to and approved by the relevant IRB(s)/EC(s) and the DAIDS RSC prior to implementing the amendment.

## Investigator's Records

The study site investigator will maintain, and store in a secure manner, complete, accurate and current study records throughout the study. The investigator will retain all study records for at least three years after submission of the Clinical Trial Unit’s (CTU) final Financial Status Report to DAIDS, which is due within 90 days after the end of the CTU’s cooperative agreement with DAIDS, unless otherwise specified by DAIDS or the HPTN CORE. Study records include administrative documentation — including protocol registration documents and all reports and correspondence relating to the study — as well as documentation related to each participant screened for and/or enrolled in the study — including informed consent forms, locator forms, case report forms, notations of all contacts with the participant, and all other source documents.

## Use of Information and Publications

Publication of the results of this study will be governed by the HPTN Manual of Operations and policies. Any presentation, abstract, or manuscript will be submitted by the Investigator to the HPTN Manuscript Review Committee for review prior to submission.

# References

# Appendices

# Appendix I: Sample Informed Consent Form – CHIPS/HFS cohort

**PARTICIPANT’S STATEMENT OF CONSENT**

**Title of Research Study:** **The Role of HIV-related Stigma in the Delivery of Universal Test and Treatment for HIVprevention**

**Protocol #:** HPTN 071a, Version 1.0, 06 January 2014

**Sponsor:** National Institute of Allergy and Infectious Diseases

National Institute of Mental Health

(U.S. National Institutes of Health)

Office of the United States Global AIDS Coordinator

Bill and Melinda Gates Foundation

**Investigator of Record:** *(insert name)*

**Research Site Address(es):** *(insert address)*

**Daytime telephone number(s):** *(insert number)*

**24-hour contact number(s):** *(insert number)*

**Participant Information and Consent Form**

You are being asked to take part in this research study because you are a health care worker or health facility staff member who took part in the PopART trial. Before you decide if you want to join this study, we want you to know about the study. This Screening/Enrollment consent form gives you information about this study. PopART Stigma staff will talk with you about the study and answer any questions you may have. Please ask the study investigator or the study staff to explain any words or procedures that you do not clearly understand.

The purpose of this form is to give you information about the research study you are being asked to join. If you sign this form, you will be giving your permission to take part in the study. The form describes the purpose, procedures, benefits, and risks of the research study. You should take part in the study only if you want to do so. You may choose not to join the research project or withdraw from this study at any time. Choosing not to take part in this research will not in any way affect your job, the health care or benefits that you or your family will receive. Please read this Participant Information and Consent Form and ask as many questions as needed. You should not sign this form if you have any questions that have not been answered to your satisfaction.

This study is being funded by the U.S. National Institutes of Health, the Office of the United States Global AIDS Coordinator, and the Bill and Melinda Gates Foundation

**Your participation is voluntary**

You do not have to take part in this study. If you decide today to take part in this research project, you may refuse to take part in any portion of the study or stop at any time without reducing or affecting your job or any care that you receive at the health centers in your community. If you do decide to leave the study early, you will be given an exit interview.

**Purpose of the Research in the Communities**

The main goal of this study is to investigate the working conditions, attitudes and experiences of lay and trained health care workers/staff delivering “universal test and treat” (UTT) within the PopART trial. Data collected will be integrated with PopART trial data, including stigma and socioeconomic conditions within communities, to strengthen the interpretation of the main trial analysis. The focus will be on understanding how HIV-related stigma, alongside health system factors and socioeconomic conditions are affected by PopART and the role they play in determining the success or failure of the approach.

**What will happen during this study?**

There will be 3 study visits for this study (including this one) and there will be approximately one year between each visit. This first visit will take approximately 75-90 minutes. Each subsequent visit will be approximately 30-60 minutes. The stigma ancillary field team will hand one electronic data collection device (EDC) to you; these devices will be similar to those used throughout the PopART intervention. On the EDC, you will find a data collection tool/survey. The stigma ancillary field team will explain how to access the appropriate version of the stigma survey on the EDC. You will then complete the survey at your leisure, and the stigma ancillary field team will remain in the room to assist you with issues of clarity. When you complete the survey, you will hand the EDC back and can resume your regular duties for the day. The survey will ask questions related to 1) *your work environment* (e.g. number and type of staff in your health facility, client load, availability of HIV testing and treatment services, workplace conditions); 2) *your personal characteristics* (e.g. sociodemographic characteristics, work experience, attitudes towards and interactions with marginalized populations, stigma in your workplace, your own experiences of HIV testing and treatment). A total of 1240 individuals are expected to participate in the study.

**What are the possible risks or discomforts?**

Some of the questions that you will be asked will be of a sensitive nature. You may become embarrassed, worried or anxious when asked questions about HIV, sexual risk behavior and other topics. A trained staff member will help you deal with any feelings or questions you have. You may feel that being part of this study could lead to you feeling stigmatized or separated from your community. You may also be worried that your participation in this survey may lead to gossip among your peers.

Another possible risk of this study is loss of confidentiality of the information you give. In particular, you may worry that responses given in the questionnaires or interviews will find its way back to individuals with the power to alter the terms of you employment. Every effort will be made to protect your confidential information, but this cannot be guaranteed. To reduce this risk, data collected on the EDCs will be downloaded within one day of each interview to password protected secure computers under the management of the PopART data team. Data will be checked and cleaned centrally. The interview process and survey progress will be frequently de-briefed and closely monitored by an in-country researcher. Data will be stored securely at a location away from the study sites. Respondents will be identified by a unique identifier in the database and not by name or other identifying information.

**What are the potential benefits?**

There are no direct benefits to participating in this study. However, the information you provide may help researchers improve counseling materials about medication use and sexual behavior, and may help identify ways to improve reporting these behaviors in future studies.

**Are there any alternatives to participation?**

There may be other studies going on here or in the community that you may be eligible for. If you wish, we will tell you about other studies we know about.

**How will my confidentiality and privacy be protected?**

We cannot guarantee absolute confidentiality. However, we will do everything possible to protect your confidentiality if you join this study. We do this by giving you a study number and any information will be labeled with this number only. Your personal information (name, address, phone number) will be protected by the research staff. This information will not be used in any publication of information about this study.

People who may review your records include: [insert name of site IRB/EC], local regulatory agencies, government authorities, (United States) National Institute of Allergy and Infectious (NIAID) contractors, US National Institutes of Health (NIH), study staff, and study monitors. Institutional Review Boards (IRBs) or Ethics Committees (ECs) are committees that watch over the safety and rights of research participants.

**What happens if I am injured by participating in this study?**

It is very unlikely that you could be injured as a result of participating in this study. Although social harms due to this study are expected to be negligible, they will be monitored closely throughout.  We encourage you to report any social harm that you experience, as and when they occur.  If you do experience negative social harms such as negative impacts on your employment or harassment by your neighbors, friends, or family, every effort will be made by study staff to provide appropriate care and counseling to you, and/or to refer you to appropriate resources and care. You [will/will not] have to pay for this care. There is no program for compensation either through this intuition or the United States NIH. You will not be giving up any of your legal rights by signing this Participant Information and Consent Form.

**Costs to you**

There is no cost to you for being in this study.

**What are some reasons why I may be withdrawn from this activity without my consent?**

Participants may be terminated from the study if they are unwilling or unable to comply with required study procedures. You may also be withdrawn from the study without your consent for the following reasons:

- The research study, or this part of the study, is stopped or canceled
- The study staff feels that completing the study or this part of the study would be harmful to you or others

**Persons to Contact for Problems or Questions**

If you have any questions about your participation in this research study, your rights as a research Participant, or if you feel that you have experienced a research-related injury, contact:

**Investigator of Record Name:** *(site insert name of the investigator or other study staff)*

**Research Site Address(es):** *(site insert physical address of above)*

**Daytime telephone number(s):** *(site insert telephone number)*

**24-hour contact number(s):** *(site insert telephone number)*

If you have any questions or concerns about your rights as a research Participant or want to discuss a problem, get information or offer input, you may contact:

**Independent Review Board/Ethics Committee:** *(site insert name or title of person on the IRB, EC or other organization appropriate for the site)*

**Address of Independent Review Board:***(site insert physical address of above)*

**Daytime Telephone Number:** *(site insert telephone number of above)*

**PARTICIPANT’S STATEMENT OF CONSENT**

***HPTN 071a:* The Role of HIV-related Stigma in the Delivery of Universal Test and Treatment for HIVprevention**

- I have been given sufficient time to consider whether to take part in this study.
- My taking part in this research study is voluntary. I may decide not to take part or to withdraw from the research study at any time without penalty or loss of benefits or treatment to which I am entitled.
- The research study may be stopped at any time without my consent.
- I have had an opportunity to ask my study investigator questions about this research study. My questions so far have been answered to my satisfaction.
- I have been told how long I may be in the research study.
- I have been informed of the procedures and tests that may be performed during the research study.
- I have been told what the possible risks and benefits are from taking part in this research study. I may not benefit if I take part in this research study.
- I do not give up my legal rights by signing this form.
- I have been told that before any study related procedures being performed, I will be asked to voluntarily sign this Participant Information and Consent Form.
- I will receive a signed and dated copy of this Participant Information and Consent Form.

If you have either read or have heard the information in this Participant Information and Consent Form, if all of your questions have been answered, and if you agree to take part in the study, please print and sign your name and write the date on the line below.

I voluntarily agree to take part in this research study.

_______________________ ________________________________________

Participant’s Name (print) Participant’s Signature and Date

I certify that the information provided was given in a language that was understandable to the Participant.

_______________________ ________________________________________

Name of Study Staff Study Staff Signature and Date

Conducting Consent Discussion (print)

_______________________ ________________________________________

Witness’ Name (print) Witness’ Signature and Date

(As appropriate) Date

# Appendix II: Sample Informed Consent Form – Qualitative study participants

**PARTICIPANT INFORMATION AND CONSENT FORM**

**Title of Research Study:** **The Role of HIV-related Stigma in the Delivery of Universal Test and Treatment for HIVprevention**

**Protocol #:** HPTN 071 a, Version 1.0, 06 January 2014

**Sponsor:** National Institute of Allergy and Infectious Diseases

National Institute of Mental Health

(U.S. National Institutes of Health)

Office of the United States Global AIDS Coordinator

Bill and Melinda Gates Foundation

**Investigator of Record:** *(insert name)*

**Research Site Address(es):** *(insert address)*

**Daytime telephone number(s):** *(insert number)*

**24-hour contact number(s):** *(insert number)*

**Participant Information and Consent Form**

Please ask the study investigator or the study staff to explain any words or procedures that you do not clearly understand.

The purpose of this form is to give you information about the research study you are being asked to join. If you sign this form, you will be giving your permission to take part in the study. The form describes the purpose, procedures, benefits, and risks of the research study. You should take part in the study only if you want to do so. You may choose not to join the research project or withdraw from this study at any time. Choosing not to take part in this research will not in any way affect the health care or benefits that you or your family will receive. Please read this Participant Information and Consent Form and ask as many questions as needed. You should not sign this form if you have any questions that have not been answered to your satisfaction.

This study is being funded by the U.S. National Institutes of Health, the Office of the United States Global AIDS Coordinator, and the Bill and Melinda Gates Foundation

**Your participation is voluntary**

You do not have to take part in this study. If you decide today to take part in this research project, you may refuse to take part in any portion of the study or stop at any time without reducing or affecting any care that you receive at the health centers in your community.

**Purpose of the Research in the Communities**

The main goal of this study is to investigate the working conditions, attitudes and experiences of lay and trained health care workers and staff delivering “universal test and treat” (UTT) within the PopART trial. Data collected will be integrated with HPTN071 trial data, including on stigma and socioeconomic conditions within communities, to strengthen the interpretation of the main trial analysis. The focus will be on understanding how HIV-related stigma, alongside health system factors and socioeconomic conditions are affected by PopART and the role they play in determining the success or failure of the approach.

**What will happen during this study?**

[*This section will vary depending on the study population.*]

**Community Health Workers and Health Facility Staff:** We are implementing this research to better understand your experiences as a HFS member/CHiP-worker who is involved in HIV service-delivery. If you decide to participate in this study, you will have one study visit, including today; there may be an additional 3-4 visits if you feel that they would be helpful for us in understanding your work. For HFS only: please know that choosing not to take part in this research will not in any way affect your job. To better understand your experiences, we would like to interact with you in different ways over the course of a day. We will meet you in the morning before you start working, to ask you some interview questions about your experiences. This may last about 30-45 minutes. We would like to spend the remainder of the day with you, seeing how you interact with clients and other people. This process is not meant to ‘check-up’ or judge you, but to better understand what it is like to do your work. While we spend the day with you, please take the time to explain what is happening, and we may ask further questions to better understand what is happening. Also, if you feel like there are more things that we need to know to understand your work, we are available to talk and interact with you and your colleagues, friends and family as you think best. These additional interactions will depend on what you feel comfortable sharing with us and what you feel is important for us to know. Typically, this additional time will occur in the days after the initial interview/observation day but the time period is flexible according to your wishes and circumstances. Where appropriate to the data collection process, and with your consent, still photography and video will be used to complement the audio data. Photographs and recordings will be stored in a locked cabinet in a secure facility and will only be accessible to study staff. If you do not wish to be photographed or recorded, please let us know. You can still participate in the survey without being recorded or photographed. Approximately 40 health workers will participate in this part of the study.

**Clients at Risk of Exclusion:** We are implementing this research to better understand the relationship between health services and different groups of potential clients. You have been asked to participate in this research because we were told that you are well informed about the experiences of [insert relevant CARE group] in relation to health services. If you decide to participate, you will have 4 study visits, including your visit today. During your first visit (today), we would like to ask you to tell us three types of stories or experiences, so that we can get to know you (where you are from, how you came to live here, etc.), and learn more about your experiences in health services. For each of the three stories, we would like to conduct an interview with you, lasting approximately 30-45mins. We will conduct a separate interview for each story, on a different day and will try to plan for all three interviews will be completed over the course of 3-4 weeks, depending on your availability. It is also possible for us to spend more time with you if you feel that there are other things that we need to see or hear to better understand your experiences. Where appropriate to the data collection process, and with your consent, still photography and video will be used to complement the audio data. Photographs and recordings will be stored in a locked cabinet in a secure facility and will only be accessible to study staff. If you do not wish to be photographed or recorded, please let us know. You can still participate in the survey without being recorded or photographed.

**What are the possible risks or discomforts?**

Some of the questions that we will ask you will be of a sensitive nature. You may become embarrassed, worried or anxious when asked questions about HIV, sexual risk behavior and other topics. A trained staff member will help you deal with any feelings or questions you have. You may feel that being part of this study could lead to you feeling stigmatized or separated from your community. You may also be worried that your participation in this survey may lead to gossip among your peers.

Another possible risk of this study is loss of confidentiality of the information you give. In particular, you may worry that responses given in the questionnaires or interviews will find its way back to individuals with the power to alter the terms of you employment. Every effort will be made to protect your confidential information, but this cannot be guaranteed. To reduce this risk, data collected on the EDCs will be downloaded within one day of each interview to password protected secure computers under the management of the PopART data team.

**What are the potential benefits?**

There are no direct benefits to participating in this study. However, the information you provide may help researchers improve counseling materials about medication use and sexual behavior, and may help identify ways to improve reporting these behaviors in future studies.

**Are there any alternatives to participation?**

There may be other studies going on here or in the community that you may be eligible for. If

you wish, we will tell you about other studies we know about.

**How will my confidentiality and privacy be protected?**

We cannot guarantee absolute confidentiality. However, we will do everything possible to protect your confidentiality if you join this study. We do this by giving you a study number and any information will be labeled with this number only. Your personal information (name, address, phone number) will be protected by the research staff. This information will not be used in any publication of information about this study. All electronic data will be deleted off the memory cards of devices such as voice recorders and cameras.

People who may review your records include: [insert name of site IRB/EC], local regulatory agencies, government authorities, (United States) National Institute of Allergy and Infectious (NIAID) contractors, US National Institutes of Health (NIH), study staff, and study monitors. Institutional Review Boards (IRBs) or Ethics Committees (ECs) are committees that watch over the safety and rights of research participants.

**What happens if I am injured by participating in this study?**

It is very unlikely that you could be injured as a result of participating in this study. Although social harms due to this study are expected to be negligible, they will be monitored closely throughout.  We encourage you to report any social harm that you experience, as and when they occur.  If you do experience negative social harms such as negative impacts on your employment or harassment by your neighbors, friends, or family, every effort will be made by study staff to provide appropriate care and counseling to you, and/or to refer you to appropriate resources and care. You [will/will not] have to pay for this care. There is no program for compensation either through this intuition or the United States NIH. You will not be giving up any of your legal rights by signing this Participant Information and Consent Form.

**Costs to you**

There is no cost to you for being in this study.

**What are some reasons why I may be withdrawn from this activity without my consent?**

Participants may be terminated from the study if they are unwilling or unable to comply with required study procedures. You may also be withdrawn from the study without your consent for the following reasons:

- The research study, or this part of the study, is stopped or canceled
- The study staff feels that completing the study or this part of the study would be harmful to you or others

**Persons to Contact for Problems or Questions**

If you have any questions about your participation in this research study, your rights as a research Participant, or if you feel that you have experienced a research-related injury, contact:

**Investigator of Record Name:** *(site insert name of the investigator or other study staff)*

**Research Site Address(es):** *(site insert physical address of above)*

**Daytime telephone number(s):** *(site insert telephone number)*

**24-hour contact number(s):** *(site insert telephone number)*

If you have any questions or concerns about your rights as a research Participant or want to discuss a problem, get information or offer input, you may contact:

**Independent Review Board/Ethics Committee:** *(site insert name or title of person on the IRB/EC or other organization appropriate for the site)*

**Address of Independent Review Board:** *(site insert physical address of above)*

**Daytime Telephone Number:** *(site insert telephone number of above)*

**PARTICIPANT’S STATEMENT OF CONSENT**

***HPTN 071a:* The Role of HIV-related Stigma in the Delivery of Universal Test and Treatment for HIVprevention**

- I have been given sufficient time to consider whether to take part in this study.
- My taking part in this research study is voluntary. I may decide not to take part or to withdraw from the research study at any time without penalty or loss of benefits or treatment to which I am entitled.
- The research study may be stopped at any time without my consent.
- I have had an opportunity to ask my study investigator questions about this research study. My questions so far have been answered to my satisfaction.
- I have been told how long I may be in the research study.
- I have been informed of the procedures and tests that may be performed during the research study.
- I have been told what the possible risks and benefits are from taking part in this research study. I may not benefit if I take part in this research study.
- I do not give up my legal rights by signing this form.
- I have been told that before any study related procedures being performed, I will be asked to voluntarily sign this Participant Information and Consent Form.
- I will receive a signed and dated copy of this Participant Information and Consent Form.

If you have either read or have heard the information in this Participant Information and Consent Form, if all of your questions have been answered, and if you agree to take part in the study, please print and sign and your name and write the date on the line below.

I voluntarily agree to take part in this research study.

_______________________ _____________________________________

Participant’s Name (print) Participant’s Signature and Date

I certify that the information provided was given in a language that was understandable to the Participant.

I consent to having photography and video of my interview being used to compliment audio data (optional).

_______________________ _____________________________________

Participant’s Name (print) Participant’s Signature and Date

_______________________ ______________________________________

Name of Study Staff Study Staff Signature and Date

Conducting Consent Discussion (print)

_______________________ _______________________________________

Witness’ Name (print) Witness’ Signature and Date

(As appropriate) Date
