# Supplementary material for: The effect of universal testing and treatment on HIV stigma in 21 communities in Zambia and South Africa
Source: AIDS. 2020 Aug 6;34(14):2125–35. doi: 10.1097/QAD.0000000000002658 (PMC8425632; doi:10.1097/QAD.0000000000002658)
Supplement: Supplemental Digital Content [file aids-34-2125-s003.docx]

Supplemental Figure 3. Estimates of any stigma prevalence among PC-HIV+^SR^ participants and log ratio residuals for the seven triplets (three communities each).


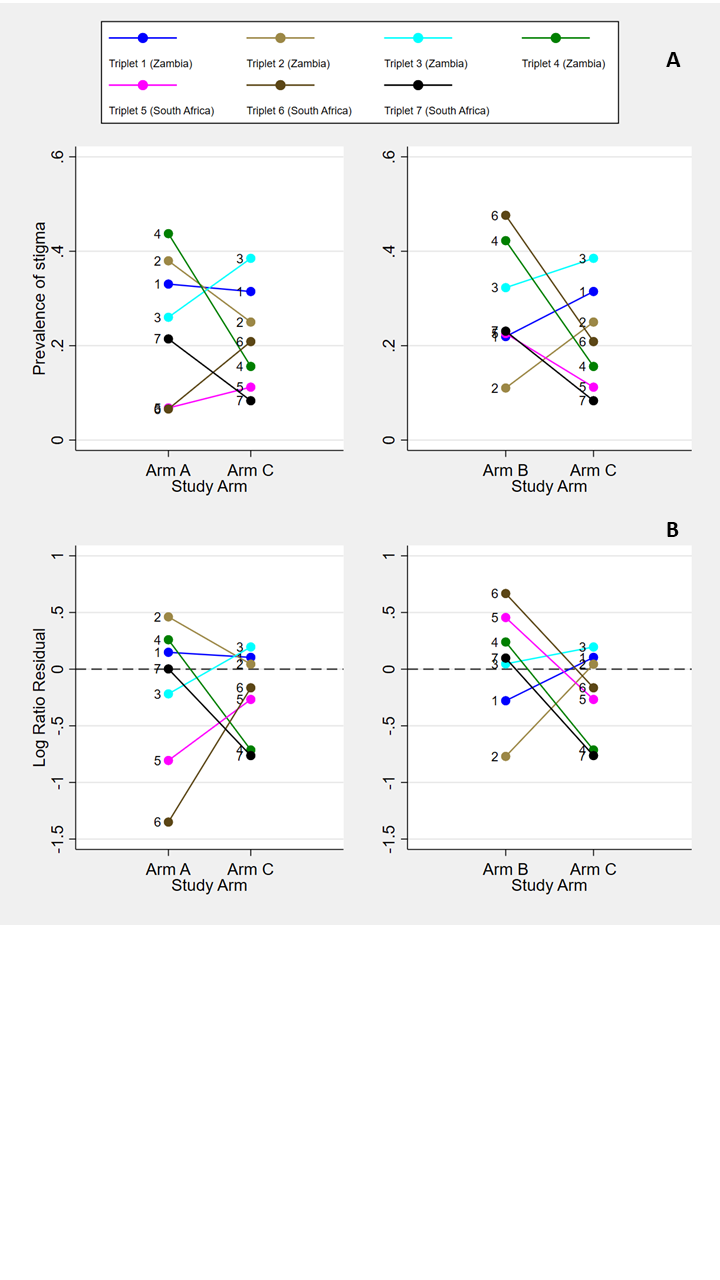


The plots show estimates of any stigma prevalence among PC-HIV+^SR^ participants at PC36 (Panel A) and log ratio residuals (ratio of the observed divided by the expected number of stigma events with adjustment for age, sex, and baseline any HIV sigma prevalence) (Panel B) for group A as compared with group C and for group B as compared with group C. Colored lines represent each of the seven triplets (numbered 1 to 7).
