# Supplementary figures and images for: The effect of universal testing and treatment on HIV stigma in 21 communities in Zambia and South Africa
Source: AIDS. 2020 Aug 6;34(14):2125–35. doi: 10.1097/QAD.0000000000002658 (PMC8425632; doi:10.1097/QAD.0000000000002658)

Supplemental Figure 2. Flow Charts


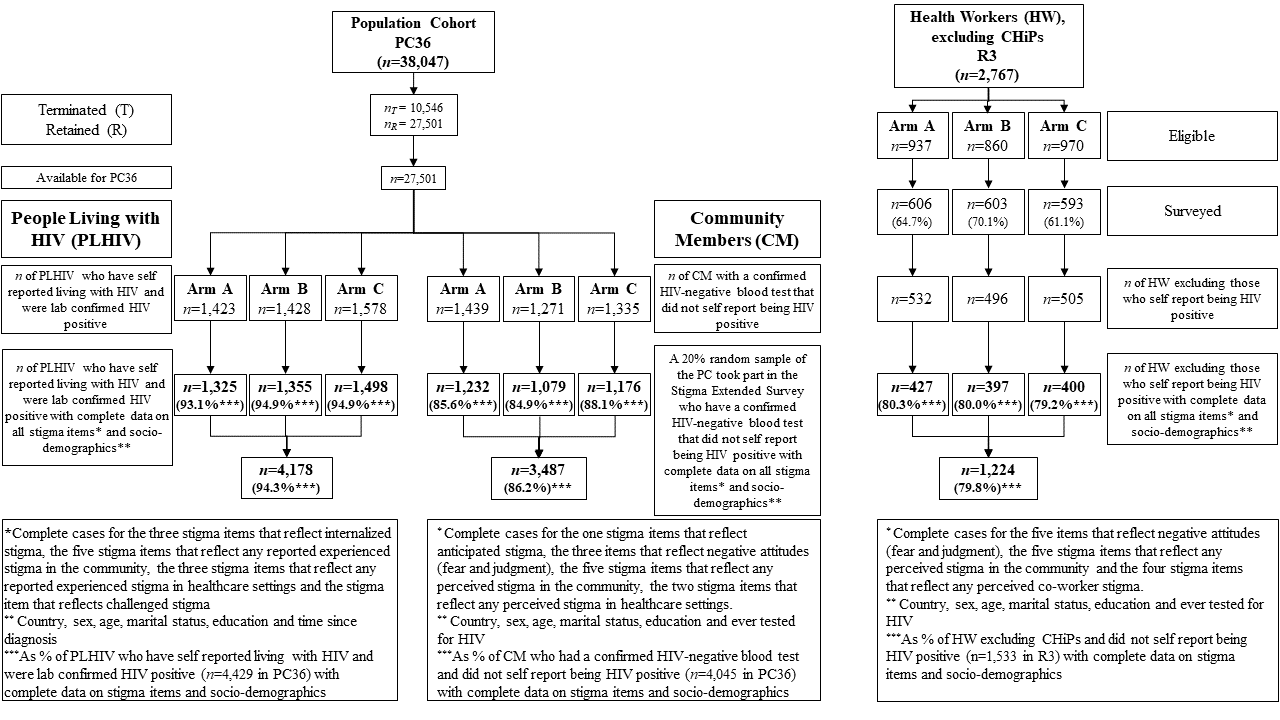

Supplement: Supplemental Digital Content [file aids-34-2125-s004.docx]
