# Supplementary material for: The effect of universal testing and treatment on HIV stigma in 21 communities in Zambia and South Africa
Source: AIDS. 2020 Aug 6;34(14):2125–35. doi: 10.1097/QAD.0000000000002658 (PMC8425632; doi:10.1097/QAD.0000000000002658)
Supplement: Supplemental Digital Content [file aids-34-2125-s005.docx]

Supplemental Table 2. Baseline differences between arms in stigma outcomes

| **Stigma outcomes** | **Arm A** | **Arm B** | **Arm C** |
| --- | --- | --- | --- |
|  | **n/N (% ^a^)** | **n/N (% ^a^)** | **n/N (% ^a^)** |
| **PC-HIV+^SR^** |  |  |  |
| Any stigma (11 items **^b^**) | 284/1138 (24.1) | 550/1420 (35.5) | 515/1267 (45.3) |
| Any reported internalized stigma (3 items **^b^**) | 160/1138 (11.2) | 362/1420 (23.3) | 332/1267 (30.7) |
| Any reported experienced stigma in the community (5 items **^b^**) | 162/1138 (13.5) | 343/1420 (20.2) | 334/1267 (29.8) |
| Any reported experienced stigma in healthcare setting (3 items **^b^**) | 44/1138 (2.5) | 135/1420 (5.5) | 99/1267 (8.0) |
| Challenged stigma **^c, d^** | 51/172 (23.5) | 174/359 (34.9) | 152/352 (44.3) |
| **PC-HIV-** |  |  |  |
| People are hesitant to take an HIV test due to fear of other people's reaction if the test is positive for HIV | 832/1594 (46.8) | 853/1370 (62.7) | 844/1253 (65.4) |
| Any negative attitudes (fear and judgment) (3 items **^b^**) | 229/1594 (12.5) | 325/1370 (22.2) | 379/1253 (25.5) |
| Any perceived stigma in community setting (5 items **^b^**) | 801/1594 (45.9) | 939/1370 (65.2) | 930/1253 (72.3) |
| Any perceived stigma in healthcare setting (2 items **^b^**) | 293/1594 (17.4) | 417/1370 (26.8) | 516/1253 (38.8) |
| **HW-HIV-** |  |  |  |
| Negative attitudes (fear and judgment) (5 items **^b^**) | 121/276(43.9) | 160/314 (49.7) | 108/261 (36.3) |
| Any perceived stigma in the community (5 items **^b^**) | 255/276 (92.3) | 292/314 (93.4) | 232/261 (89.7) |
| Any perceived co-worker stigma (4 items **^b^**) | 122/276 (36.1) | 150/314 (42.7) | 115/261 (39.8) |
| Job stress (emotional exhaustion) (9 items **^b, e^**) | 72/260 (25.8) | 96/298 (28.9) | 87/242 (32.9) |

^a^ Geometric means across communities

^b^ See Appendix 1 for wording of items included in multi-item indicators

^c^ “I confronted, challenged, or educated someone who was stigmatising and/or discriminating against me” asked among those who reported experiencing any form of stigma in the community or healthcare settings

^d^ No events in one community in Arm B, 0.5 was added to the number of events and the number of participants for all communities in that triplet at the first stage of the analysis

^e^ Smaller sample size due to missing data
