# Supplementary material for: The effect of universal testing and treatment on HIV stigma in 21 communities in Zambia and South Africa
Source: AIDS. 2020 Aug 6;34(14):2125–35. doi: 10.1097/QAD.0000000000002658 (PMC8425632; doi:10.1097/QAD.0000000000002658)
Supplement: Supplemental Digital Content [file aids-34-2125-s006.docx]

Supplemental Table 1. Stigma and emotional exhaustion outcomes among people living with HIV, community members and health workers.

| **Outcome** | **Items*** | **Treated in analysis** |
| --- | --- | --- |
| ***PC-HIV+^SR^ (Five outcomes)*** | | |
| Any internalized stigma  (3 items) | - I have lost respect or standing in the community because of my HIV status - I think less of myself because of my HIV status - I have felt ashamed because of my HIV status | 4-item Likert scale (Strongly agree to strongly disagree). |
| Any experienced stigma in the community  (5 items) | - People have talked badly about me because of my HIV status - Someone else disclosed my HIV status without my permission - I have been verbally insulted, harassed and/or threatened because of my HIV status - I have been physically assaulted because of my HIV status - I have felt that people have not wanted to sit next to me, for example, on public transport, at church or in a waiting room because of my HIV status | Frequency of experiences (Never, once, a few times, often). |
| Any experienced stigma in healthcare settings  (3 items) | - I have been denied health services because of my HIV status - Healthcare workers talked badly about me because of my HIV status - A health worker disclosed my HIV status without my permission | Frequency of experiences (Never, once, a few times, often). |
| Any stigma  (11 items) | - Includes stigma items from any internalized stigma (3 items), any experienced stigma in the community (5 items) and healthcare settings (3 items) | Treated as main outcome measure in overall summaries of the findings. |
| Challenged stigma  (1 item) | - I confronted, challenged, or educated someone who was stigmatising and/or discriminating against me (PC0 and P36) | Single item asked at all data rounds; frequency of experiences (Never, once, a few times, often) |
| ***PC-HIV- (Four outcomes)*** | | |
| Any anticipated stigma  (1 item) | - People are hesitant to take an HIV test due to fear of other people's reaction if the test is positive for HIV | 4-item Likert scale (Strongly agree to strongly disagree). |
| Any negative attitudes  (3 items) | - I fear that I could contract HIV if I come into contact with the saliva of a person living with HIV - I would not like to sit close to someone living with HIV, for example on public transport, at church or in a waiting room - I would be ashamed if someone in my family had HIV | 4-item Likert scale (Strongly agree to strongly disagree). |
| Any perceived stigma in community setting (5 items) | - People thought to be living with HIV are sometimes physically assaulted - People sometimes talk badly about PLHIV to others - People thought to be living with HIV lose respect or standing - People thought to be living with HIV are verbally insulted, harassed and/or threatened - People sometimes disclose that other people are HIV positive without their permission | 4-item Likert scale (Strongly agree to strongly disagree). |
| Any perceived stigma in healthcare setting (2 items) | - Health workers sometimes talk badly about people living with or thought to be living with HIV to others - Health workers sometimes disclose that other people are HIV positive with their permission | 4-item Likert scale (Strongly agree to strongly disagree). |
| ***HW-HIV- (Four outcomes)*** | | |
| Any negative attitudes  (5 items) | - I fear that I could contract HIV if I come into contact with the saliva of a person living with HIV - I avoid physical contact with clients living with HIV - HIV is punishment from God - Other people deserve access to health services more than PLHIV - I would be ashamed if someone in my family had HIV | 4-item Likert scale (Strongly agree to strongly disagree). |
| Any perceived stigma in the community perceptions  (5 items) | - People thought to be living with HIV are sometimes physically assaulted - People sometimes talk badly about PLHIV to others - People thought to be living with HIV lose respect or standing - People thought to be living with HIV are verbally insulted, harassed, or threatened - People hesitate to start ARV drugs because they are afraid others will learn they are living with HIV | 4-item Likert scale (Strongly agree to strongly disagree) |
| Any perceived co-worker stigma  (4 items) | - My co-workers sometimes talk badly about people thought to be living with HIV - My co-workers sometimes gossip about clients' HIV test results - My co-workers sometimes treat people living with HIV poorly when providing them with health services - My co-workers sometimes verbally insult clients living with HIV | 4-item Likert scale (Strongly agree to strongly disagree) |
| Emotional exhaustion  (9 items) | - I feel emotionally drained from my work - I feel used up at the end of the workday - I feel tired when I get up in the morning and have to face another day on the job - Working with people all day is really hard/difficult for me - I feel burned out from my work - I feel frustrated by my job - I feel I'm working too hard on my job - Working with people directly puts too much stress on me - I feel like I have reached the end | Frequency of experiences (Never, few times a year, once a month or less, few times a month, once a week, few times a week, or everyday) |

*Respondents were asked whether they felt, thought or experienced each statement in the past 12 months; PC: Population cohort
