# Supplementary material for: The effect of universal testing and treatment on HIV stigma in 21 communities in Zambia and South Africa
Source: AIDS. 2020 Aug 6;34(14):2125–35. doi: 10.1097/QAD.0000000000002658 (PMC8425632; doi:10.1097/QAD.0000000000002658)
Supplement: Supplemental Digital Content [file aids-34-2125-s007.docx]

Supplemental Table 3. Sensitivity analyses conducted for PLHIV outcome models.

| **Outcome** | **Arm A** | **Arm B** | **Arm C** | **Arm A+B** | **Arm A vs C** | | **Arm B vs C** | | **Arm A+B vs C** | |
| --- | --- | --- | --- | --- | --- | --- | --- | --- | --- | --- |
|  | **n/N (% ^a^)** | **n/N (% ^a^)** | **n/N (% ^a^)** | **n/N (% ^a^)** | **PR (95% CI)** | **aPR ^b^ (95% CI)** | **PR (95% CI)** | **aPR ^b^ (95% CI)** | **PR (95% CI)** | **aPR ^b^ (95% CI)** |
| **PC-HIV+^SR^ with data at all four rounds (n=1,078) ^c^** |  |  |  |  |  |  |  |  |  |  |
| Any stigma ^d, h^ | 96/309  (18.6) | 104/419  (25.4) | 74/350  (19.5) | 200/728  (21.8) | 1.00  (0.37–2.71) | 1.05  (0.39–2.79) | 1.32  (0.49–3.60) | 1.33  (0.50–3.57) | 1.15  (0.48–2.73) | 1.18  (0.50–2.77) |
| Any internalized stigma ^d, h^ | 51/309  (9.7) | 54/419  (9.8) | 36/350  (6.2) | 105/728  (9.7) | 1.61  (0.46–5.66) | 2.12  (0.68–6.60) | 1.66  (0.47–5.86) | 1.68  (0.54–5.22) | 1.64  (0.55–4.87) | 1.89  (0.71–5.04) |
| Any experienced stigma in the community ^d, h^ | 64/309  (13.9) | 69/419  (17.9) | 55/350  (15.3) | 133/728  (15.8) | 0.94  (0.40–2.20) | 1.02  (0.46–2.29) | 1.15  (0.49–2.69) | 1.20  (0.54–2.68) | 1.04  (0.50–2.17) | 1.11  (0.55–2.22) |
| Any experienced stigma in healthcare setting ^, h e^ | 6/309  (2.5) | 16/419  (2.8) | 8/350  (2.6) | 22/728  (2.6) | 0.92  (0.22–3.83) | 1.09  (0.25–4.73) | 1.28  (0.31–5.34) | 1.42  (0.33–6.19) | 1.09  (0.32–3.74) | 1.24  (0.35–4.44) |
| **PC-HIV+^SR^ diagnosed before the start of the trial**  **(PopART) in 2014 (n=2,140)** |  |  |  |  |  |  |  |  |  |  |
| Any stigma ^f, h^ | 162/620  (18.6) | 214/751  (26.8) | 188/769  (22.3) | 376/1371  (22.3) | 0.82  (0.40–1.67) | 0.87  (0.42–1.82) | 1.18  (0.58–2.42) | 1.24  (0.60–2.58) | 0.98  (0.53–1.83) | 1.04  (0.55–1.96) |
| Any internalized stigma ^f, h^ | 93/620  (8.8) | 107/751  (13.0) | 87/769  (10.3) | 200/1371  (10.7) | 0.82  (0.30–2.24) | 0.89  (0.32–2.44) | 1.22  (0.45–3.31) | 1.29  (0.47–3.54) | 1.00  (0.42–2.38) | 1.07  (0.44–2.57) |
| Any experienced stigma in the community ^f, h^ | 105/620  (10.0) | 153/751  (19.1) | 145/769  (17.6) | 258/1371  (13.8) | 0.54  (0.23–1.28) | 0.64  (0.27–1.55) | 1.03  (0.44–2.44) | 1.15  (0.48–2.77) | 0.75  (0.35–1.57) | 0.86  (0.40–1.84) |
| Any experienced stigma in healthcare setting ^g, h^ | 15/620  (2.6) | 37/751  (3.3) | 26/769  (2.3) | 52/1371  (3.0) | 1.05  (0.28–3.91) | 1.11  (0.30–4.08) | 1.33  (0.36–4.95) | 1.37  (0.37–5.05) | 1.18  (0.38–3.69) | 1.23  (0.40–3.81) |

^a^ Geometric means across communities;

^b^ Adjusted prevalence ratio (aPR) adjusted for triplet, age, sex, the interaction between age and sex, and the baseline cluster-level of the outcome of interest;

^c^ No challenging stigma sensitivity analysis was possible because the sample of PLHIV who experienced any stigma in community or healthcare settings in the past 12 months and had data at all four rounds was too small (n=284) with the majority of communities having zero events;

^d^ No events in one community in Arm A;

^e^ No events in three communities in Arm A, two communities in Arm B and two communities in Arm C;

^f^ No events in one community in Arm C;

^g^ No events in one community in Arm B and three communities in Arm C;

^h^ 0.5 was added to the number of events and the number of participants for all communities in that triplet at the first stage of the analysis
